# Supplementary material for: Research on the development of an automated system for psychology questionnaire generation based on large language models
Source: PLoS One. 2026 Apr 24;21(4):e0345117. doi: 10.1371/journal.pone.0345117 (PMC13108753; doi:10.1371/journal.pone.0345117)
Supplement: S4 Data — (ZIP) [file pone.0345117.s004.zip › S5_Code (Model & Training Configuration)/psychological-questionnaire-label.en_only.docx]

{"questionnaire_title": "GWB (Chinese version)", "questionnaire_category": "Emotion and affect", "item_count": 96, "emotion_shift_overall": "From negative to positive", "language_style_overall": "Tactful and warm", "items": [{"content": "Are you troubled by illness, physical discomfort, pain or fear of disease?", "word_count": 24, "label": "", "sentence_structure": "Subject鈥搗erb鈥搊bject", "subjective_objective": {"answer": "Objective", "rationale": "The fact that disease, physical discomfort, pain or fear of disease are objective facts."}, "sentence_sentiment": {"answer": "Negative", "rationale": "Because the word \"fear\" in the entry is negative emotional, implying an unhealthy emotional state. And \"disease, physical discomfort, pain, or fear of disease\" is also negative."}, "language_style": {"answer": "Tactful and warm", "rationale": "This questionnaire item is softer and uses some expressions of concern and understanding, such as \"fear,\" \"discomfort,\" \"incommodity,\" \"injury.\" These words imply that researchers are concerned and respectful of the person being investigated, not just asking an objective fact. Moreover, the questionnaire is more euphemismal and friendly, and does not have a too direct or too soft language. This language style helps to ease the tension of the person surveyed and makes them more willing to participate in the questionnaire."}, "time_dimension": {"answer": "Past", "rationale": ""}, "sentence_type": "Interrogative sentence", "pos": {"nouns": {"Diseases": 1, "Body": 2, "I'm not well": 1, "Pain": 1, "Fear": 1, "Trouble": 1}, "verbs": {"Is it possible": 1, "Because": 1, "Sick": 1, "And": 1, "Trouble": 1}, "numerals": {"1": 2}, "pronouns": {"you": 1}}}, {"content": "Are you tired, overtired, powerless or exhausted?", "word_count": 16, "label": "", "sentence_structure": "Subject鈥搗erb鈥搊bject", "subjective_objective": {"answer": "Subjective", "rationale": "Because fatigue, overtiredness, incompetence or exhaustion are subjective feelings of the patient."}, "sentence_sentiment": {"answer": "Negative", "rationale": "Because the words \"tired, overtired, weak or exhausted\" in the entry are negative."}, "language_style": {"answer": "Calm and direct", "rationale": "The language style of the entry is more straightforward, and there is no excessive rhetorical or emotional rendering. It asks the other person in an objective manner whether he or she feels physical fatigue, overtired, weak or exhausted, and is relatively flat."}, "time_dimension": {"answer": "Present", "rationale": ""}, "sentence_type": "Interrogative sentence", "pos": {"nouns": {"Tired": 1, "I'm tired": 1, "- I'm weak": 1, "I'm exhausted": 1}, "verbs": {"Sensor": 1, "Present": 1}}}, {"content": "Are you awake with a clear mind and energy?", "word_count": 18, "label": "", "sentence_structure": "Subject鈥搗erb鈥搊bject", "subjective_objective": {"answer": "Subjective", "rationale": "Because \"Whether you feel clear and energetic after you wake up\" is the patient's subjective feeling."}, "sentence_sentiment": {"answer": "Positive", "rationale": "Because \"clear and energetic\" is a positive description."}, "language_style": {"answer": "Tactful and warm", "rationale": "The language style of the entry is more euphemistic and gentle, using the form of \"Do you feel after awaken\" in a way that is relatively friendly and friendly and that does not make people feel too direct or stabbing. This tone may make respondents more willing to cooperate with the questionnaire and to obtain more effective feedback."}, "time_dimension": {"answer": "Present", "rationale": "The entry asks if the person tested has a feeling or state in the current situation (i.e. \"after you wake up\"), or \"clear and energetic\" and thus judges it now."}, "sentence_type": "Interrogative sentence", "pos": {"nouns": {"you": 1, "Wake up": 1, "Brain": 1, "- The energy": 1}, "verbs": {"I feel": 1, "鈾?Full of 鈾?: 1}, "adjectives": {"Wake up": 1, "- I'm very energetic": 1}, "numerals": {"3": 1}}}, {"content": "Four, are you sleeping hard?", "word_count": 7, "label": "", "sentence_structure": "Subject鈥搗erb鈥揷omplement", "subjective_objective": {"answer": "Objective", "rationale": "Because \" sleep difficulties\" are objective facts that can be observed and measured."}, "sentence_sentiment": {"answer": "Negative", "rationale": "Because \"sleeping is difficult\" is a negative phenomenon."}, "language_style": {"answer": "Tactful and warm", "rationale": "The entry is more apt and acceptable when it is spoken in a gentle, inquisitive way, using a question-and-answer form, without a direct statement or command. At the same time, \"sleep difficulty\" is a relatively sensitive issue, and asking questions in this way reduces the stress and discomfort of the other."}, "time_dimension": {"answer": "Present", "rationale": "The entry asks whether the person tested currently has sleep difficulties, so the time dimension is now judged."}, "sentence_type": "Interrogative sentence", "pos": {"nouns": {"Difficulties": 1, "Sleep": 1}, "verbs": {"have": 1, "- You're not": 1}, "numerals": {"4": 1}}}, {"content": "How do you feel in general?", "word_count": 8, "label": "", "sentence_structure": "Subject鈥損redicate", "subjective_objective": {"answer": "Subjective", "rationale": "Because \"feel\" is usually the result of a subjective experience."}, "sentence_sentiment": {"answer": "Neutral", "rationale": ""}, "language_style": {"answer": "Tactful and warm", "rationale": "The language style of the entry is more euphemistic, it is expressed in a more proximate language, and it is not too formal or rigid, and it is more likely to resonate and respond to the respondents."}, "time_dimension": {"answer": "Present", "rationale": ""}, "sentence_type": "Interrogative sentence", "pos": {"nouns": {"you": 1, "Total": 1, "Body": 1, "Feel": 1, "Sample": 1}, "verbs": {"Here": 1, "Out": 1}, "numerals": {"5": 1}}}, {"content": "Are you happy?", "word_count": 6, "label": "", "sentence_structure": "Subject鈥損redicate", "subjective_objective": {"answer": "Subjective", "rationale": "Because feeling happy is a subjective emotional experience."}, "sentence_sentiment": {"answer": "Positive", "rationale": "Because \"favouring happiness\" in the entry is a positive emotional experience."}, "language_style": {"answer": "Tactful and warm", "rationale": ""}, "time_dimension": {"answer": "Present", "rationale": ""}, "sentence_type": "Interrogative sentence", "pos": {"nouns": {"Questionnaire Entry": 1, "Happiness": 1}, "verbs": {"I feel": 1, "Give": 0}, "adverbs": {"- You're not": 1}}}, {"content": "7. Are you worried, worried or disturbed?", "word_count": 12, "label": "", "sentence_structure": "Subject鈥搗erb鈥搊bject", "subjective_objective": {"answer": "Subjective", "rationale": "The patient's subjective emotional experience is the result of anxiety, anxiety or anxiety."}, "sentence_sentiment": {"answer": "Negative", "rationale": "Because \"anxiety, fear or unease\" in the entry is a negative emotional experience."}, "language_style": {"answer": "Calm and direct", "rationale": "The entry directly asks whether the other party is anxious, worried or uneasy, is not using excessive rhetoric or emotional rendering, and is more objective in tone."}, "time_dimension": {"answer": "Present", "rationale": ""}, "sentence_type": "Interrogative sentence", "pos": {"nouns": {"Anxiety": 1, "I'm worried": 1, "Unsettled": 1}, "verbs": {"I feel": 1, "or": 1}, "numerals": {"7": 1}}}, {"content": "Do you have a lot of things to be interested in in your daily life?", "word_count": 20, "label": "", "sentence_structure": "Subject鈥搗erb鈥搊bject", "subjective_objective": {"answer": "Subjective", "rationale": "Because the question is about the patient ' s perception and feelings of life, it is subjective."}, "sentence_sentiment": {"answer": "Neutral", "rationale": "Since the entry is an objective one, it does not involve emotional colour."}, "language_style": {"answer": "Tactful and warm", "rationale": "The words are more euphemistic, first to lead each other to questions in a question-and-answer manner, and then to use the word \"filled\" to express positive meaning, so that respondents feel that they are being cared for and encouraged. At the same time, the whole sentence is more gentle and friendly, without any imposition or criticism, and is more closely related to the way in which human beings communicate."}, "time_dimension": {"answer": "Present", "rationale": "The entry asks about the current status of the testee, and the keywords \"whether\" and \"day\" and \"life full\" indicate that this is a description and evaluation of current life. So the time dimension is now."}, "sentence_type": "Interrogative sentence", "pos": {"nouns": {"Life": 1, "Things": 1}, "verbs": {"Fill": 1, "鈾?And I'm gonna be 鈾?: 1, "Interested": 1}, "adjectives": {"Chile": 1}, "numerals": {"8": 1}}}, {"content": "- What's the experience in your life?", "word_count": 11, "label": "", "sentence_structure": "Subject鈥損redicate", "subjective_objective": {"answer": "Subjective", "rationale": "Because \"good experience\" is a description of a subjective feeling or view, it is a subjective perspective."}, "sentence_sentiment": {"answer": "Positive", "rationale": "Because the entry asks about the good experiences of life, it is a positive way of asking questions."}, "language_style": {"answer": "Tactful and warm", "rationale": "The language style of the entry is more euphemistic and warm, and dialogue is conducted in a question-and-answer tone, which gives the interviewee a sense of respect and attention, while avoiding too direct or imposed attitudes."}, "time_dimension": {"answer": "Present", "rationale": "The entry asks about the current situation in the life of the person being tested, and the time dimension is now judged."}, "sentence_type": "Interrogative sentence", "pos": {"nouns": {"Life": 1, "Experience": 1}, "verbs": {"have": 1}, "numerals": {"9": 1}}}, {"content": "10. Are you happy, happy or happy?", "word_count": 13, "label": "", "sentence_structure": "Subject鈥搗erb鈥搊bject", "subjective_objective": {"answer": "Subjective", "rationale": "Because the question is about patients ' subjective feelings and assessments, they examine their subjective perception of their own happiness, satisfaction or pleasure in their own lives."}, "sentence_sentiment": {"answer": "Positive", "rationale": "Because the entry describes subjective perceptions of quality of life and positive emotions and attitudes."}, "language_style": {"answer": "Tactful and warm", "rationale": "The language style of the entry is relatively euphemistic, gentle and encouraging. It does not directly ask the interviewee about happiness or satisfaction, but rather \"whether or not\" to guide the interviewee to think about it and answer it. Moreover, the word \"happy\" gives people a positive and positive feeling. Such expression makes it easier for the interviewee to relax and to share his real feelings."}, "time_dimension": {"answer": "Present", "rationale": "The keywords are \"you\" and \"now,\" both of which point to the period under investigation, the current state. So the entry asks about the current living conditions of the testee, and the time dimension is now."}, "sentence_type": "Interrogative sentence", "pos": {"nouns": {"Life": 1, "Happiness": 1, "Satisfied": 1, "Have a nice day": 1}, "verbs": {"be": 2, "Is it possible": 1}, "adjectives": {"you": 1}, "numerals": {"10": 1}}}, {"content": "11: Your satisfaction with school attendance.", "word_count": 10, "label": "", "sentence_structure": "Subject鈥搗erb鈥搊bject", "subjective_objective": {"answer": "Subjective", "rationale": "Because satisfaction or dissatisfaction is a subjective feeling of the patient."}, "sentence_sentiment": {"answer": "Neutral", "rationale": "Because the entry does not have any emotional color."}, "language_style": {"answer": "Tactful and warm", "rationale": "The language style of the entry is euphemism. It uses the form of questioning, and does not directly evaluate the good or bad of the school, but rather allows the interviewee to express his satisfaction with the school. This way allows the interviewee to be more willing to share his or her views and feelings, and to reflect them more objectively."}, "time_dimension": {"answer": "Present", "rationale": ""}, "sentence_type": "Interrogative sentence", "pos": {"nouns": {"School": 1, "Satisfaction": 1}, "verbs": {"Studying": 1}, "numerals": {"11": 1}, "classifiers": {"individual": 1}}}, {"content": "- You're satisfied with your choice of profession?", "word_count": 12, "label": "", "sentence_structure": "Subject鈥損redicate", "subjective_objective": {"answer": "Subjective", "rationale": "Because satisfaction is the patient's subjective feeling and attitude."}, "sentence_sentiment": {"answer": "Neutral", "rationale": "Because the entry does not have any emotional color."}, "language_style": {"answer": "Tactful and warm", "rationale": "The entry uses more euphemistic language, avoiding asking the other directly whether the other party is satisfied with his or her profession, but instead leads the other to think and express his or her attitude and opinion about the profession."}, "time_dimension": {"answer": "Present", "rationale": "The keyword \"satisfaction with the profession of his choice\" represents the current mindset and attitude of the testee, and the entry is therefore the current description."}, "sentence_type": "Interrogative sentence", "pos": {"nouns": {"Professional": 1, "Satisfied": 1}, "verbs": {"Selection": 1}, "adverbs": {"self": 1}, "numerals": {"12": 1}, "classifiers": {"be": 1}}}, {"content": "Are you satisfied with your academic achievement?", "word_count": 12, "label": "", "sentence_structure": "Subject鈥損redicate", "subjective_objective": {"answer": "Subjective", "rationale": "Since satisfaction is a subjective feeling and assessment of one person, the degree of satisfaction of the same achievement may vary from one person to another."}, "sentence_sentiment": {"answer": "Positive", "rationale": "As the issue of entries is one of individual satisfaction, satisfaction with learning achievement is usually a positive attitude."}, "language_style": {"answer": "Calm and direct", "rationale": "The entry is in the form of a direct question asking the other party whether they are satisfied with their academic performance, without any qualifier language or emotional colour, and with a more balanced and objective tone."}, "time_dimension": {"answer": "Present", "rationale": "The entry asks the testee about his satisfaction with his current learning achievement, and the time dimension is now judged."}, "sentence_type": "Interrogative sentence", "pos": {"nouns": {"Learning achievement": 1, "Satisfied": 1}, "verbs": {"to": 1, "self": 1}, "adverbs": {"not": 1}, "numerals": {"13": 1}}}, {"content": "Are you satisfied with the respect you have received?", "word_count": 10, "label": "", "sentence_structure": "Subject鈥搗erb鈥搊bject", "subjective_objective": {"answer": "Subjective", "rationale": "The basis for the judgement: \"Are you satisfied with the respect of others?\" is a subjective question, because it requires the respondent to provide their personal views and feelings. The question is examined by the subjective attitude and emotional reaction of the person under investigation, not just by information about an objective fact."}, "sentence_sentiment": {"answer": "Neutral", "rationale": "Because the entry is merely asking the other party whether it is satisfied with the respect of others, it does not involve any positive or negative emotional colour."}, "language_style": {"answer": "Tactful and warm", "rationale": "The entry is more adept and interactive when it is spoken in a gentle and inviting manner, rather than by direct statements or orders."}, "time_dimension": {"answer": "Present", "rationale": "The entry asks about the current feelings and attitudes of the testee, the keyword is \"satisfaction\", which is a vocabulary describing the current state."}, "sentence_type": "Interrogative sentence", "pos": {"nouns": {"Respect": 1, "person": 1, "Satisfied": 1}, "verbs": {"to": 1, "Respect": 1, "Satisfied": 1}, "numerals": {"14": 1}, "classifiers": {"you": 1}}}, {"content": "Are you satisfied with the understanding of others?", "word_count": 10, "label": "", "sentence_structure": "Subject鈥損redicate", "subjective_objective": {"answer": "Subjective", "rationale": "The understanding of others is that the patient has subjective feelings and views and is therefore subjective."}, "sentence_sentiment": {"answer": "Positive", "rationale": "Because the word \"other\" in the entry is understood as a description of the emotions and attitudes associated with it, satisfaction is usually understood as a positive emotional experience. Thus, the purpose of the article tends to be positive."}, "language_style": {"answer": "Tactful and warm", "rationale": "The entry is more intimate and interactive when it is dialogueable in a query manner, rather than directly speaking or ordering. At the same time, the entry is worded in a more moderate manner, without overly subjective or emotional rendering, and therefore in a soft and warm language style."}, "time_dimension": {"answer": "Present", "rationale": "The entry is for the current feelings and perceptions of the testee, and the time dimension is now judged."}, "sentence_type": "Interrogative sentence", "pos": {"nouns": {"others": 1, "Understood": 1}, "verbs": {"Satisfied": 1, "Here": 0}, "adverbs": {"to": 1}, "numerals": {"15": 1}, "pronouns": {"you": 1}}}, {"content": "16. Have you always had firm control over your behaviour, thinking, emotions or feelings?", "word_count": 23, "label": "", "sentence_structure": "Subject鈥損redicate", "subjective_objective": {"answer": "Subjective", "rationale": "Because the problem involves personal feelings and self-awareness, it is a subjective view of the patient."}, "sentence_sentiment": {"answer": "Negative", "rationale": "Because the word \"contain\" in the entry gives people a sense of restraint and depression, which is a term for negative emotions."}, "language_style": {"answer": "Tactful and warm", "rationale": "The language style of the entry is euphemism. It does not directly blame or order the interviewee, but rather engages in dialogue in a gentle and inviting tone that gives the interviewee a sense of respect and understanding, which is more personal and interactive."}, "time_dimension": {"answer": "Present", "rationale": "The use of the general current \"always\" in the entry means that the testee is asked about the current state of conduct, and the judgement is \"now\"."}, "sentence_type": "Interrogative sentence", "pos": {"nouns": {"Questionnaire": 1, "Entry": 1, "Behaviour": 1, "Thinking": 1, "Emotion": 1, "Feel": 1}, "verbs": {"Is it possible": 1, "It's firmly controlled": 1}, "adverbs": {"Always": 1}, "numerals": {"16": 1}}}, {"content": "17. Do you doubt that there is anything worth doing because of grief, loss of confidence, disappointment or many problems?", "word_count": 32, "label": "", "sentence_structure": "Subject鈥搗erb鈥搊bject", "subjective_objective": {"answer": "Subjective", "rationale": "Because it's the feeling and the thought that people feel inside, it's a subjective view."}, "sentence_sentiment": {"answer": "Negative", "rationale": "Because the attitude in the entry, \"Suspect anything worth doing,\" is negative."}, "language_style": {"answer": "Tactful and warm", "rationale": "The entry uses a caring and understanding tone to ask if the respondents have ever been lost by negative emotions, and attempts to create a safe and comfortable atmosphere of dialogue that allows the respondents to express their thoughts and feelings more easily. At the same time, the language does not direct orders or criticism of the emotional state of the respondents, but rather guides them to think and answer questions in a balanced and caring manner."}, "time_dimension": {"answer": "Present", "rationale": "The temporal dimension of the article is therefore now judged."}, "sentence_type": "Interrogative sentence", "pos": {"nouns": {"Sad": 1, "Confidence": 1, "Disappointment": 1, "Trouble": 1, "Yes": 1}, "verbs": {"Do it": 1, "Doubt": 1}, "adjectives": {"Many": 1, "Any": 1}, "adverbs": {"And": 1}, "numerals": {"17": 1}, "pronouns": {"you": 1}}}, {"content": "18. Are you being or ever being subjected to any constraint, stimulus or pressure?", "word_count": 21, "label": "", "sentence_structure": "Subject鈥搗erb鈥搊bject", "subjective_objective": {"answer": "Subjective", "rationale": "The question is whether the patient has a subjective feeling of being restrained, irritated or under pressure, either currently or in the past."}, "sentence_sentiment": {"answer": "Negative", "rationale": "Because the entries \"constrained,\" \"stimulated\" and \"pressure\" have negative emotional aspects."}, "language_style": {"answer": "Calm and direct", "rationale": "The language style of the entry is calm and straightforward. It asks questions in a concise and straightforward manner, without any rhetorical or emotional rendering, directly asking the interviewee whether he is subjected to any constraints, irritation or pressure, and is neutral in tone. This style is suitable for use in rigorous scientific research and questionnaires, ensuring the accuracy and effectiveness of the problem."}, "time_dimension": {"answer": "Present", "rationale": "\"Accepting\" and \"already\" are current or past acts, and the subject therefore asks about the general state of the present and the general past, i.e. whether there are present or past situations of constraint, stimulation or pressure."}, "sentence_type": "Interrogative sentence", "pos": {"nouns": {"Questionnaire": 1, "Entry": 1, "Constraints": 1, "Exciting": 1, "Pressure": 1}, "verbs": {"Received": 2, "In the process of": 1, "Once": 1}, "adverbs": {"Any": 1}, "numerals": {"18": 1}}}, {"content": "Do you have reason to suspect that you have lost your mind or control over behaviour, conversation, thinking or memory?", "word_count": 31, "label": "", "sentence_structure": "Subject鈥搗erb鈥搊bject", "subjective_objective": {"answer": "Objective", "rationale": "This is because it is an objective experience and behaviour of the patient that is being examined for whether he has ever had circumstances beyond his control."}, "sentence_sentiment": {"answer": "Negative", "rationale": "Because the description of \"unsane\" and \"unsustained behaviour, conversation, thought or memory\" in this entry is usually associated with negative emotional and psychological conditions, and thus expresses negative emotional colours."}, "language_style": {"answer": "Calm and direct", "rationale": "The language style of the entry is relatively calm and objective, and it allows respondents to think and answer the question directly, without too much emotional overturning or euphemism. This language style avoids causing the interviewee to be emotionally volatile and is more conducive to obtaining real and accurate answers."}, "time_dimension": {"answer": "Past", "rationale": "The entry asks whether the person tested had in the past been mentally disinherited or had lost control over his behaviour, conversation, mind or memory, and therefore judged to be the past."}, "sentence_type": "Interrogative sentence", "pos": {"nouns": {"Grandpa": 1, "Questionnaire": 1, "Entry": 1, "Reason": 1, "Behaviour": 1, "Talk": 1, "Thinking": 1, "Memory": 1, "Control": 1}, "verbs": {"have": 1, "Doubt": 1, "Lose": 2, "I'm losing control": 1, "Reason, or loss of behavior, conversation, thought or memory": 1, "Reason or loss of behavior, conversation, thought or memory": 1}, "adverbs": {"Once": 1, "I'm losing control": 1}, "numerals": {"19": 1}, "pronouns": {"you": 1}}}, {"content": "How was your attention during class?", "word_count": 10, "label": "", "sentence_structure": "Subject鈥搗erb鈥揷omplement", "subjective_objective": {"answer": "Subjective", "rationale": "Because \"focus\" is the patient's own experience and feelings, and it's a subjective assessment."}, "sentence_sentiment": {"answer": "Neutral", "rationale": "Because the entry does not have any emotional color."}, "language_style": {"answer": "Tactful and warm", "rationale": "The language style of the entry is more euphemistic, using a query-type speech structure, without direct criticism or evaluation, making it easier for the respondents to accept the problem and give real feedback. At the same time, by using the phrase \"how to focus\", the entry avoids being too direct to question the learning attitude and abilities of the other party, but instead focuses more on understanding their actual situation."}, "time_dimension": {"answer": "Present", "rationale": "The keyword is \"in class\" and it indicates the person's behaviour or state during a certain period of time. And the verb \"how to focus\" also indicates that it is an assessment of the current situation, so the entry is used to assess the current focus of the person being tested."}, "sentence_type": "Interrogative sentence", "pos": {"nouns": {"Questionnaire": 1, "Entry": 1, "During class": 1, "Attention": 1}, "verbs": {"you": 1, "Attention": 1}, "numerals": {"20": 1}}}, {"content": "How's your memory coming along?", "word_count": 9, "label": "", "sentence_structure": "Subject鈥搗erb鈥揷omplement", "subjective_objective": {"answer": "Subjective", "rationale": "Because memory is a subjective feeling and experience of one鈥檚 own, different people may judge their own memory differently. For example, a person with a strong memory may feel good about his recent memory, while a person with a weaker memory may feel that his recent memory has decreased."}, "sentence_sentiment": {"answer": "Neutral", "rationale": "Since the entry does not address any specific behaviour, attitude, emotion, etc., it merely asks the person under investigation about his recent memory and therefore does not have a clear positive or negative emotional profile."}, "language_style": {"answer": "Tactful and warm", "rationale": ""}, "time_dimension": {"answer": "Past", "rationale": ""}, "sentence_type": "Interrogative sentence", "pos": {"nouns": {"Memory": 1, "Recent": 1}, "verbs": {"How's that": 1, "Give": 1}}}, {"content": "22, what's your overall academic achievement compared to your class?", "word_count": 18, "label": "", "sentence_structure": "Subject鈥搗erb鈥搊bject", "subjective_objective": {"answer": "Subjective", "rationale": "The examination is based on the patient ' s evaluation and perception of his or her academic performance and is therefore subjective."}, "sentence_sentiment": {"answer": "Neutral", "rationale": "Because the entry is merely a question of learning and does not involve positive or negative emotional aspects."}, "language_style": {"answer": "Calm and direct", "rationale": "The language style of the entry is more direct, with no over-modification or emotional expression, and the interviewer is primarily asked to answer his/her academic performance, which is an objective, neutral language style."}, "time_dimension": {"answer": "Present", "rationale": "The entry asks the testee about his current performance relative to his peers, so the time dimension is now judged."}, "sentence_type": "Interrogative sentence", "pos": {"nouns": {"Classmate": 1, "Learning achievement": 1, "Situation": 1}, "verbs": {"Compare": 1, "be": 1}, "adverbs": {"and": 1}, "numerals": {"22": 1}}}, {"content": "23, I can learn even if it's hard to do.", "word_count": 11, "label": "", "sentence_structure": "Subject鈥搗erb鈥搊bject", "subjective_objective": {"answer": "Subjective", "rationale": "Because \"I can learn to be an expression of attitude and conviction, even if it is hard to do my homework,\" which reflects patients' sense of self-evaluation and self-effectiveness, it is a subjective perspective."}, "sentence_sentiment": {"answer": "Positive", "rationale": "Because the behavior in the entry \"can learn\" is positive, expressing a man's attitude of perseverance and determination."}, "language_style": {"answer": "Tactful and warm", "rationale": "The entry uses an encouraging and positive language style, expresses confidence and recognition of the capacity of the person surveyed and raises questions in a moderate manner, making it easier for the person interviewed to open up and share real ideas."}, "time_dimension": {"answer": "Present", "rationale": "The entry describes a learning attitude or behaviour of the person tested, and in \"even if it's hard to do\" the time key word also indicates that it is a continuous, customary behaviour and therefore judgmental of the moment."}, "sentence_type": "Interrogative sentence", "pos": {"nouns": {"Work": 1}, "verbs": {"Yes": 2, "Learn": 1}, "adjectives": {"Hard": 1}, "adverbs": {"Yeah": 1, "Both": 0}, "numerals": {"23": 1}, "pronouns": {"I": 1}}}, {"content": "I can solve most of the difficulties that I usually encounter in learning.", "word_count": 19, "label": "", "sentence_structure": "Subject鈥搗erb鈥搊bject", "subjective_objective": {"answer": "Subjective", "rationale": "The subject is a question of the patient鈥檚 own abilities, emphasizing the attitude and beliefs of the individual."}, "sentence_sentiment": {"answer": "Positive", "rationale": "Because the behaviour in the entry \"solves the problem\" is positive, while the degree of description is also positive ( \"most\")."}, "language_style": {"answer": "Calm and direct", "rationale": "The language style of the entry is calm and straightforward, because it is simple, not using too much accommodative or emotional expression. It directly asks whether the person surveyed can solve the usual learning difficulties, and is an objective, factual and question-and-answerer."}, "time_dimension": {"answer": "Present", "rationale": "The keyword \"can solve\" indicates that this is a description of the behaviour in the general current, and that \"the vast majority of the difficulties encountered in learning\" suggests that this is a process or behaviour that is currently under way. Thus, the entry falls within the category of the current act or state."}, "sentence_type": "Interrogative sentence", "pos": {"nouns": {"I": 1, "Problem": 1, "Difficulty": 1}, "verbs": {"Yes": 1, "Resolve": 1, "I met him": 1}, "adjectives": {"Usually": 1, "Most": 1}, "numerals": {"24": 1, "Most of them": 1}}}, {"content": "I can learn carefully even if I have other interesting things to do.", "word_count": 21, "label": "", "sentence_structure": "Subject鈥損redicate", "subjective_objective": {"answer": "Subjective", "rationale": "This is because of the patient ' s personal attitudes and views and is a subjective examination."}, "sentence_sentiment": {"answer": "Positive", "rationale": "Because the \"scientific learning\" behavior in the entries is positive."}, "language_style": {"answer": "Tactful and warm", "rationale": "The entry is a dialogue that is conducted in a spirit of encouragement and support, expressing the determination and perseverance to learn even when faced with other attractive things. This language style can both enhance each other鈥檚 motivation and confidence and create a good atmosphere of communication."}, "time_dimension": {"answer": "Present", "rationale": "The entry indicates the ability or tendency of the testee in the current state to learn with a focus on other interesting things, even if they are available. The time dimension is therefore judged as now."}, "sentence_type": "Affirmative declarative sentence", "pos": {"nouns": {"Things": 1, "Learning": 1}, "verbs": {"Yes": 1, "Do it": 1, "Seriously": 1, "Learning": 1}, "adjectives": {"Interesting": 1}, "adverbs": {"Yeah": 1}, "numerals": {"25": 1}, "pronouns": {"I": 1}}}, {"content": "For me, it is easy to adhere to ideals and to achieve goals.", "word_count": 19, "label": "", "sentence_structure": "Subject鈥揷opula鈥損redicative", "subjective_objective": {"answer": "Subjective", "rationale": "Because the word \"I\" in the sentence refers to the person interviewed, indicating that it is self-evaluation and attitude of the person interviewed."}, "sentence_sentiment": {"answer": "Positive", "rationale": "Because the expression \"to uphold the ideals and achieve the goals\" and \"to achieve the goals\" in the entries are positive emotional expressions of individual confidence and positive attitudes towards their abilities and objectives."}, "language_style": {"answer": "Calm and direct", "rationale": "The entry is more direct in terms of asking the other side whether it considers it easy to adhere to the ideals and to achieve the goals."}, "time_dimension": {"answer": "Present", "rationale": "The entry indicates the attitude and perception of the speaker towards adherence to the ideals and achievement of the goals, and this attitude is the current state. The time dimension therefore is now judged."}, "sentence_type": "Affirmative declarative sentence", "pos": {"nouns": {"Ideal": 1, "Objective": 1}, "verbs": {"Hold on": 1, "Achieved": 1, "be": 1, "Easy": 1}, "adjectives": {"Easy": 1}, "numerals": {"26": 1}, "pronouns": {"I": 1}}}, {"content": "27 I can make a good arrangement for a place to study and be undisturbed.", "word_count": 19, "label": "", "sentence_structure": "Subject鈥搗erb鈥揷omplement", "subjective_objective": {"answer": "Subjective", "rationale": "Because the title refers to \"I can do well,\" which is the patient's own perception and assessment, and is therefore subjective."}, "sentence_sentiment": {"answer": "Positive", "rationale": "Because the \"can be a good place to learn\" behavior and purpose in the entry are positive."}, "language_style": {"answer": "Tactful and warm", "rationale": "This expression is more sensitive and respectful, and helps to increase the participation of the respondents and the effectiveness of the questionnaire."}, "time_dimension": {"answer": "Present", "rationale": "The entry describes a capability that indicates that the person tested can effectively learn from a place and is not disturbed."}, "sentence_type": "Affirmative declarative sentence", "pos": {"nouns": {"Local": 1, "Learning": 1, "Excuse me": 1}, "verbs": {"Yes": 1, "Arrangements": 1}, "adjectives": {"Okay": 1}, "numerals": {"27": 1}, "pronouns": {"I": 1}}}, {"content": "Do you have faith in yourself?", "word_count": 8, "label": "", "sentence_structure": "Subject鈥損redicate", "subjective_objective": {"answer": "Subjective", "rationale": "Because confidence is the idea and feeling within the individual, it is subjective."}, "sentence_sentiment": {"answer": "Positive", "rationale": "Because \"confidence\" in the entry shows the positive emotional color of subjective attitudes."}, "language_style": {"answer": "Tactful and warm", "rationale": "The entry begins with an inquiry, expressing concern for the inside of the interviewee and using the word \"confidence\" to stimulate positive thinking and a more intimate and inspiring overall language style."}, "time_dimension": {"answer": "Present", "rationale": ""}, "sentence_type": "Interrogative sentence", "pos": {"nouns": {"Confidence": 1, "self": 1}, "verbs": {"have": 1, "Here": 1}, "adverbs": {"not": 1}, "numerals": {"28": 1}, "pronouns": {"you": 1}}}, {"content": "Are you depressed by your appearance?", "word_count": 11, "label": "", "sentence_structure": "Subject鈥搗erb鈥搊bject", "subjective_objective": {"answer": "Subjective", "rationale": "Because the problem involves the patient ' s perception and feelings of his or her own appearance, it is the patient ' s subjective attitude."}, "sentence_sentiment": {"answer": "Negative", "rationale": "Because the word \"pressure\" in the entries has a negative emotional connotation."}, "language_style": {"answer": "Tactful and warm", "rationale": "The language style of the entry is more euphemistic, and asks if the other side is disturbed or negative, expressed in a more friendly and non-intrusive manner."}, "time_dimension": {"answer": "Present", "rationale": ""}, "sentence_type": "Interrogative sentence", "pos": {"nouns": {"Looks": 1, "Repression": 1}, "verbs": {"鈾?And I'm gonna be 鈾?: 1, "I feel": 1, "Repression": 1}, "adverbs": {"- You're not": 1}, "numerals": {"29": 1}}}, {"content": "30, do you have any uncomfortable parts on your face?", "word_count": 16, "label": "", "sentence_structure": "Subject鈥搗erb鈥搊bject", "subjective_objective": {"answer": "Subjective", "rationale": "Because feeling uncomfortable is a subjective feeling and emotional experience."}, "sentence_sentiment": {"answer": "Negative", "rationale": "Because the \"part of the line that makes you uncomfortable\" expresses a negative mood and attitude."}, "language_style": {"answer": "Calm and direct", "rationale": "Although it is a question of whether the other party has physical appearance, it also provides respect for the privacy of the individual. The question does not contain any language that may cause discomfort or insecurity, but rather is raised in an open and understanding manner. At the same time, the question does not imply or force the other party to answer, but rather gives the other party a free choice."}, "time_dimension": {"answer": "Present", "rationale": "The words \"if\" indicate that this is a question of choice, \"the appearance,\" \"the uncomfortable part,\" point to the current situation and feelings of the testee. So the entry to the questionnaire is to ask the testee what the testee is now, and the temporal dimension is now."}, "sentence_type": "Interrogative sentence", "pos": {"nouns": {"Questionnaire": 1, "Part": 1}, "verbs": {"Did you": 1}, "adjectives": {"Look at it": 1, "It's not comfortable": 1}, "numerals": {"30": 1}}}, {"content": "Are you satisfied with your body?", "word_count": 10, "label": "", "sentence_structure": "Subject鈥搗erb鈥搊bject", "subjective_objective": {"answer": "Subjective", "rationale": "This is because patients have subjective perceptions and evaluations of their own body."}, "sentence_sentiment": {"answer": "Neutral", "rationale": "Because the entry asks about individual satisfaction with their body size, it does not express positive or negative emotional sentiments."}, "language_style": {"answer": "Tactful and warm", "rationale": "Warm and warm."}, "time_dimension": {"answer": "Present", "rationale": ""}, "sentence_type": "Interrogative sentence", "pos": {"nouns": {"self": 1, "Body": 1}, "verbs": {"Satisfied": 1}, "numerals": {"31": 1}}}, {"content": "32 - Are you depressed and depressed?", "word_count": 10, "label": "", "sentence_structure": "Subject鈥損redicate", "subjective_objective": {"answer": "Subjective", "rationale": "Because feeling frustration and depression is a subjective feeling or experience."}, "sentence_sentiment": {"answer": "Negative", "rationale": "Because \"depression and depression\" in the entry is negative."}, "language_style": {"answer": "Calm and direct", "rationale": "The entry directly asked the interviewees whether they were feeling depressed and depressed, were not over-modified or implied and were more straightforward."}, "time_dimension": {"answer": "Present", "rationale": "The keywords are \"are you depressed or depressed\" and they all point to the state of the mood."}, "sentence_type": "Interrogative sentence", "pos": {"nouns": {"Frustration": 1, "Blue": 1}, "verbs": {"I feel": 1}, "numerals": {"32": 1}}}, {"content": "1. How are you able to act?", "word_count": 8, "label": "", "sentence_structure": "Subject鈥損redicate", "subjective_objective": {"answer": "Subjective", "rationale": "According to the description of the subject, \"How do you act?\" is a subjective self-evaluation of the patient, and the entry is therefore viewed from the patient's subjective perspective."}, "sentence_sentiment": {"answer": "Neutral", "rationale": "Because the entry does not have any emotional color."}, "language_style": {"answer": "Calm and direct", "rationale": "This language style is more objective and concise, and it is easy for the respondents to understand and answer questions."}, "time_dimension": {"answer": "Present", "rationale": "The entry asks the testee about his current mobility, which is a description and assessment of the current situation, and therefore the temporal dimension is now judged."}, "sentence_type": "Interrogative sentence", "pos": {"nouns": {"Operational capacity": 1, "you": 1}, "verbs": {"How's that": 1, "be": 1}, "pronouns": {"Here": 1}}}, {"content": "Are you satisfied with your ability to act?", "word_count": 11, "label": "", "sentence_structure": "Subject鈥搗erb鈥搊bject", "subjective_objective": {"answer": "Subjective", "rationale": "Since satisfaction is the patient ' s subjective attitude, the problem is examined by the patient ' s subjective assessment of his or her ability to act."}, "sentence_sentiment": {"answer": "Neutral", "rationale": "Because the entry merely asks the respondents about their satisfaction with their ability to act, it does not involve positive or negative emotional attitudes."}, "language_style": {"answer": "Calm and direct", "rationale": "The language style of the entry is more direct, with no excessive adoration or emotional colour, but rather with a brief and concise presentation of the questions, which appears to be more objective and calm."}, "time_dimension": {"answer": "Present", "rationale": "The entry asks the testee about his current ability to act, without reference to the past or future time dimension. \"Do you have the ability to act?\" In \"Do you have the ability to act?\", \"Do you have the satisfaction?\", it implies that this is an assessment of and feeling for current behaviour. So, the judgement is based on \"now.\""}, "sentence_type": "Interrogative sentence", "pos": {"nouns": {"Operational capacity": 1, "Satisfied": 1}, "verbs": {"Jean": 1}, "adjectives": {"Yours": 1}, "adverbs": {"You're": 1}, "numerals": {"2": 1}, "pronouns": {"you": 1}}}, {"content": "Is it trouble to be in trouble?", "word_count": 9, "label": "", "sentence_structure": "Subject鈥搗erb鈥搊bject", "subjective_objective": {"answer": "Subjective", "rationale": "Because \"making you upset\" is the patient's subjective feeling."}, "sentence_sentiment": {"answer": "Negative", "rationale": "Because \"actional difficulties\" in the entry are negative, and \"fear you\" is negative."}, "language_style": {"answer": "Tactful and warm", "rationale": "The entry is more intimate and interactive when it is spoken in a gentle and inviting manner, rather than in direct statements or orders. At the same time, the use of the word \"fear\" is more euphemismal and avoids the pain of touching the other."}, "time_dimension": {"answer": "Past", "rationale": "The entry asks whether the person tested had problems or problems arising from operational difficulties, which may be past experiences or current situations and therefore are judged to be past."}, "sentence_type": "Interrogative sentence", "pos": {"nouns": {"Operational difficulties": 1, "Trouble": 1}, "verbs": {"鈾?And I'm gonna be 鈾?: 1, "Tired": 1, "Hard": 1, "I'm upset": 1}, "classifiers": {"How many": 1}}}, {"content": "- Does the difficulty of movement affect your lifestyle?", "word_count": 13, "label": "", "sentence_structure": "Subject鈥搗erb鈥搊bject", "subjective_objective": {"answer": "Subjective", "rationale": "Because the entry asks patients about their subjective feelings and perceptions about their own lives, whether they believe that their mobility difficulties have affected their way of life."}, "sentence_sentiment": {"answer": "Negative", "rationale": "Because \"actional difficulties\" in the entry are negative."}, "language_style": {"answer": "Tactful and warm", "rationale": "The entry, which leads the respondents to reflect on the impact of their own mobility difficulties on their lifestyle, is more moderate in tone and does not directly express accusations or judgements about the difficulties."}, "time_dimension": {"answer": "Present", "rationale": "The entry asks whether the current lifestyle of the testee is affected by the difficulties of movement, and the time dimension is now judged."}, "sentence_type": "Interrogative sentence", "pos": {"nouns": {"Operational difficulties": 1, "Lifestyle": 1}, "verbs": {"Impact": 1}, "adverbs": {"you": 1, "- You're not": 1}, "numerals": {"4": 1}}}, {"content": "- Are you up to your daytime?", "word_count": 15, "label": "", "sentence_structure": "Subject鈥搗erb鈥搊bject", "subjective_objective": {"answer": "Subjective", "rationale": "Because this issue concerns the feelings and perceptions of the patient himself, it is a subjective one."}, "sentence_sentiment": {"answer": "Positive", "rationale": "Because the attitude of \"with plenty of energy to cope with everyday life\" is positive."}, "language_style": {"answer": "Tactful and warm", "rationale": "This is a more euphemistic language, presented in a question-and-answer format, expressing concern and concern for the daily life of the person interviewed, and not a direct order or criticism."}, "time_dimension": {"answer": "Present", "rationale": "The entry asks if the person tested is currently sufficiently motivated to cope with everyday life, without reference to past or future circumstances, and can therefore be judged as now."}, "sentence_type": "Interrogative sentence", "pos": {"nouns": {"- The energy": 1, "Day": 1}, "verbs": {"Payable": 1}, "adjectives": {"鈾?Full of 鈾?: 1}, "numerals": {"5": 1}, "pronouns": {"you": 1}}}, {"content": "Can you do your own day-to-day stuff?", "word_count": 14, "label": "", "sentence_structure": "Subject鈥搗erb鈥搊bject", "subjective_objective": {"answer": "Objective", "rationale": "The basis for the determination: the subject is an objective question of whether the patient can perform his or her tasks independently in everyday life, without the patient ' s subjective views or emotional colour."}, "sentence_sentiment": {"answer": "Positive", "rationale": "As the entry does not express any negative or negative emotional aspects, it is positive to ask the interviewees whether they can perform their tasks in daily life independently."}, "language_style": {"answer": "Tactful and warm", "rationale": "The language style of the entry is more euphemistic. It is in the form of a question-and-answer sentence, and is more accommodative and moderate. This way makes the respondents more receptive to questions and more willing to answer them. At the same time, this euphemism facilitates a good survey climate and improves the rate of questionnaire recovery and data quality."}, "time_dimension": {"answer": "Present", "rationale": ""}, "sentence_type": "Interrogative sentence", "pos": {"nouns": {"Things": 1, "Daily life": 1}, "verbs": {"Yes": 1, "Do it": 1}, "adverbs": {"self": 1}, "numerals": {"6": 1}}}, {"content": "Do you have a classmate who talks about employment?", "word_count": 15, "label": "", "sentence_structure": "Subject鈥搗erb鈥搊bject", "subjective_objective": {"answer": "Subjective", "rationale": "This is a subjective attitude of the patient, as it requires the cooperation of the other party in the discussion of employment."}, "sentence_sentiment": {"answer": "Positive", "rationale": "Because the purpose of the action \"to explore employment\" in the entry is positive and contributes to the individual's career planning and development."}, "language_style": {"answer": "Tactful and warm", "rationale": "The entry asks the respondents in a more moderate way whether they often discuss employment issues with other classmates, which makes it easier to elicit positive responses and honest answers from the interviewees, and also demonstrates the respect and interest of the researchers in the interviewees."}, "time_dimension": {"answer": "Present", "rationale": "The entry asks if there is a phenomenon or behaviour, namely, \"Do you have a student who talks to you about employment?\" Because the word \"yes\" is used in the sentence, it is a sexual doubt, emphasizing the situation, and therefore the judgment is \"now\"."}, "sentence_type": "Interrogative sentence", "pos": {"nouns": {"Students": 1, "Problem": 1, "Employment": 1}, "verbs": {"Explore": 1, "Constant with": 1, "have": 1}, "adverbs": {"Regular": 1}, "numerals": {"7": 1}}}, {"content": "8. Are you under pressure to get employed?", "word_count": 10, "label": "", "sentence_structure": "Subject鈥損redicate", "subjective_objective": {"answer": "Subjective", "rationale": "Because pressure is a subjective feeling, different people may feel differently about the same situation."}, "sentence_sentiment": {"answer": "Negative", "rationale": "Because the \"pressure of employment\" in the entry is negative."}, "language_style": {"answer": "Calm and direct", "rationale": "The entry uses a direct and explicit language style, with no excessive adoration or emotional colour, and is intended to gain a direct understanding of whether the other party feels the pressure of employment."}, "time_dimension": {"answer": "Present", "rationale": "The keyword \"attendance\" represents the current state or situation, so the entry asks about the current state of the person being tested."}, "sentence_type": "Interrogative sentence", "pos": {"nouns": {"Pressure": 1, "Employment": 1}, "verbs": {"Faced": 1}}}, {"content": "Do you have a drug dependence?", "word_count": 7, "label": "", "sentence_structure": "Subject鈥搗erb鈥搊bject", "subjective_objective": {"answer": "Subjective", "rationale": "Because drug dependence is a subjective experience and evaluation. While there are medical diagnostic criteria and scales to assess the extent of drug dependence, patients ' feelings of themselves are the most immediate observers and reporters, and the problem is primarily one of patient ' s subjective attitudes and experiences."}, "sentence_sentiment": {"answer": "Negative", "rationale": "Because the drug dependence behavior in the entry is negative."}, "language_style": {"answer": "Calm and direct", "rationale": "The entry asked the testee whether there was a drug dependence problem in a directly explicit language, which was more concise and did not involve excessive rhetorical or emotional rendering."}, "time_dimension": {"answer": "Present", "rationale": "The keyword \"Yes\" indicates the current state or situation, and \"drug dependence\" is the description of the state or situation. The entry is therefore an inquiry into whether the person tested currently has a drug dependence situation."}, "sentence_type": "Interrogative sentence", "pos": {"nouns": {"Drug dependence": 1}, "verbs": {"have": 1}}}, {"content": "Do you believe in love?", "word_count": 6, "label": "", "sentence_structure": "Subject鈥損redicate", "subjective_objective": {"answer": "Subjective", "rationale": "Because \"confident in love\" is a personal conviction and attitude, a subjective feeling and a notion."}, "sentence_sentiment": {"answer": "Neutral", "rationale": "Because the entry does not have any emotional color."}, "language_style": {"answer": "Tactful and warm", "rationale": "The entry is a soft, invitation-based conversation, asking if the other party believes in the subject of love, is more objective in tone and does not express itself too strongly or directly, leaving room for reflection and exploration."}, "time_dimension": {"answer": "Present", "rationale": "The entry now asks if the person tested currently believes in love and the time dimension is now judged."}, "sentence_type": "Interrogative sentence", "pos": {"nouns": {"Love": 1}, "verbs": {"Yes": 1}, "numerals": {"10": 1}, "classifiers": {"individual": 1}}}, {"content": "Is love a big influence on your life?", "word_count": 11, "label": "", "sentence_structure": "Subject鈥搗erb鈥搊bject", "subjective_objective": {"answer": "Subjective", "rationale": "Because this question is primarily concerned with the patient ' s perception and feelings of love, it is a subjective one."}, "sentence_sentiment": {"answer": "Positive", "rationale": "Because \"love\" is usually considered a positive emotion in life, the expression of this article is positive."}, "language_style": {"answer": "Tactful and warm", "rationale": "The entry is more adept and interactive when it is spoken in a gentle and inviting manner, rather than by direct statements or orders."}, "time_dimension": {"answer": "Present", "rationale": "The entry asks about the current situation of the respondents, i.e. whether love has a greater impact on their lives and therefore is of a time dimension."}, "sentence_type": "Interrogative sentence", "pos": {"nouns": {"Love": 1, "Life": 1}, "verbs": {"Impact": 1, "Large": 1}, "numerals": {"11": 1}, "classifiers": {"individual": 1}}}, {"content": "Are you satisfied with your past relationship?", "word_count": 10, "label": "", "sentence_structure": "Subject鈥搗erb鈥搊bject", "subjective_objective": {"answer": "Subjective", "rationale": "Because \"satisfaction\" is a subjective criterion for evaluation, different people may have different views and feelings."}, "sentence_sentiment": {"answer": "Negative", "rationale": "Because \"old relationships\" are often the negative experiences of people in their memories, this problem can trigger negative emotional reactions."}, "language_style": {"answer": "Tactful and warm", "rationale": "The language style of the entry is euphemistic. It guides the interviewee to share his feelings in a way that does not directly speak or order, making him feel respected and understood."}, "time_dimension": {"answer": "Past", "rationale": "The entry asks the testee about the satisfaction of the past relationship, and the temporal dimension is a past."}, "sentence_type": "Interrogative sentence", "pos": {"nouns": {"Love": 1, "Past": 1}, "verbs": {"Satisfied": 1}, "numerals": {"12": 1, "One": 1, "Two": 1}}}, {"content": "Are you satisfied with your relationship?", "word_count": 10, "label": "", "sentence_structure": "Subject鈥損redicate", "subjective_objective": {"answer": "Subjective", "rationale": "Because \"satisfaction\" is a subjective feeling and attitude."}, "sentence_sentiment": {"answer": "Neutral", "rationale": "This question is a sign of personal attitude and satisfaction with existing relationships, and therefore has a certain emotional connotation. If the interviewee is satisfied with existing relationships, the answer is \"positive\" ; if not, the answer is \"negative\" ; and if there is no special feeling or uncertainty, the answer is \"neutral\"."}, "language_style": {"answer": "Calm and direct", "rationale": "This language style is more objective and direct, and helps to obtain a true and accurate answer."}, "time_dimension": {"answer": "Present", "rationale": "The keywords \"now in love\" and \"is it satisfied?\" indicate that the question is asked about the current state of the relationship of the interviewee, which is the time dimension of the present."}, "sentence_type": "Interrogative sentence", "pos": {"nouns": {"Love": 1}, "verbs": {"Satisfied": 1, "have": 1}, "numerals": {"13": 1}, "pronouns": {"you": 1}}}, {"content": "Does dating have a great impact on your studies?", "word_count": 12, "label": "", "sentence_structure": "Subject鈥搗erb鈥搊bject", "subjective_objective": {"answer": "Subjective", "rationale": "As impact assessments are based on the feelings and experiences of the patients themselves."}, "sentence_sentiment": {"answer": "Negative", "rationale": "Because \"love\" in this entry can distract people and focus, and thus negatively affect learning."}, "language_style": {"answer": "Tactful and warm", "rationale": "The entry is more personal and interactive by speaking in a gentle, inviting tone, rather than by direct statement or order. At the same time, by using the emotional term \"love\", the interviewee is able to share more naturally his feelings and experiences in answering questions, thereby obtaining more realistic and in-depth data."}, "time_dimension": {"answer": "Present", "rationale": "The entry asks about the current reality, and the keywords are \"loan\" and \"your learning impact\", which indicates that the person tested is experiencing the act of dating, and asks about the extent of its impact on learning. Thus, the time dimension of the entry is now judged."}, "sentence_type": "Interrogative sentence", "pos": {"nouns": {"Love": 1, "Impact": 1, "Learning": 1, "Entry": 1}, "verbs": {"Talk": 1, "Large": 1}, "numerals": {"14": 1, "- You're not": 1}}}, {"content": "Does boyfriend/girlfriend affect you a lot?", "word_count": 13, "label": "", "sentence_structure": "Subject鈥搗erb鈥搊bject", "subjective_objective": {"answer": "Subjective", "rationale": "Because the question is examined by the patient ' s perception and perception of the impact of his/her boyfriend/girlfriend on himself, it is a subjective view of the patient."}, "sentence_sentiment": {"answer": "Positive", "rationale": "Because \"boyfriend/girlfriend\" in the entry is a key partner of a person, they can have a big impact."}, "language_style": {"answer": "Calm and direct", "rationale": "The entry is more straightforward, has no excessive rhetorical or emotional rendering, and asks the respondents鈥?views in a concise and clear manner."}, "time_dimension": {"answer": "Present", "rationale": "The entry is for the current situation of the testee, with the keyword \"boyfriend/girlfriend\" and \"high impact\", all pointing to the present."}, "sentence_type": "Interrogative sentence", "pos": {"nouns": {"Boyfriend": 1, "Girlfriend": 1, "Impact": 1, "Large": 1}, "verbs": {"to": 1}, "numerals": {"15": 1}, "pronouns": {"you": 1}}}, {"content": "How's your boyfriend/girlfriend?", "word_count": 11, "label": "", "sentence_structure": "Subject鈥搗erb鈥揷omplement", "subjective_objective": {"answer": "Subjective", "rationale": "Because satisfaction is a subjective feeling and assessment, it depends on the individual鈥檚 own feelings and expectations. The issue is therefore one of examining the subjective thoughts and feelings of the patient, which are subjective in nature."}, "sentence_sentiment": {"answer": "Neutral", "rationale": "As the entry is asked about objective facts, it does not involve an evaluation or attitude towards boyfriends/girlfriends."}, "language_style": {"answer": "Tactful and warm", "rationale": "According to the information provided in the title, \"Does the boyfriend/girlfriend have a good feeling?\" The entry is more polite, respectful, and focused on each other's feelings and needs. At the same time, it uses the means of questioning rather than orders or direct statements, making it more gentle and friendly and more acceptable and more responsive. Thus, the language style of the entry can be judged as warm."}, "time_dimension": {"answer": "Present", "rationale": "The entry now asks about the current situation of the person being tested, i.e. whether his/her relationship with the boyfriend/girlfriend is satisfactory and therefore falls within the current temporal dimension."}, "sentence_type": "Interrogative sentence", "pos": {"nouns": {"Boyfriend": 1, "Girlfriend": 1, "Satisfied": 2}, "verbs": {"to": 1, "you": 2}, "numerals": {"16": 1}}}, {"content": "Do relationships now affect your future?", "word_count": 14, "label": "", "sentence_structure": "Subject鈥搗erb鈥搊bject", "subjective_objective": {"answer": "Subjective", "rationale": "Because \"the relationship\" is a subjective issue that involves the patient's own emotional state and expectations, and \"the impact on the future\" is also a subjective issue based on the patient's personal judgement and expectations. So the entry looks at the patient's subjective attitudes and ideas."}, "sentence_sentiment": {"answer": "Neutral", "rationale": "Because the entry does not have any emotional color."}, "language_style": {"answer": "Tactful and warm", "rationale": "The language style of the entry is euphemismal. It uses a softer approach to ask questions, and it leads the respondents to share their views on the relationship between their current relationship and the future. This expression reduces the negative emotions and resistance that may arise, and makes the respondents more willing to answer the questions honestly."}, "time_dimension": {"answer": "Present", "rationale": "The entry asks whether the testees' current relationship will affect their future, and the keywords are \"now\" and \"have an impact on the future,\" all pointing to what is happening and thus judging as now."}, "sentence_type": "Interrogative sentence", "pos": {"nouns": {"Love": 1, "The future": 1}, "verbs": {"have": 1, "Impact": 1}, "numerals": {"17": 1, "Present": 1}, "pronouns": {"you": 1}}}, {"content": "How many friends are you?", "word_count": 8, "label": "", "sentence_structure": "Subject鈥損redicate", "subjective_objective": {"answer": "Subjective", "rationale": "Because \"more\" is a subjective assessment of the number of people who are friends."}, "sentence_sentiment": {"answer": "Neutral", "rationale": "Because the entry does not involve emotional colour, it merely asks for an objective fact."}, "language_style": {"answer": "Tactful and warm", "rationale": "The entry is more adept and interactive when it is spoken in a gentle and inviting manner, rather than by direct statements or orders."}, "time_dimension": {"answer": "Present", "rationale": ""}, "sentence_type": "Interrogative sentence", "pos": {"nouns": {"friends": 1, "Number": 1}, "verbs": {"More": 1}, "adverbs": {"- You're not": 1}, "numerals": {"18": 1}}}, {"content": "Are you satisfied with yourself?", "word_count": 7, "label": "", "sentence_structure": "Subject鈥損redicate", "subjective_objective": {"answer": "Subjective", "rationale": "Because the subject is a subjective assessment of the patient ' s self-perception and is a subjective examination."}, "sentence_sentiment": {"answer": "Positive", "rationale": "Because \"satisfaction\" in the entry is a positive attitude."}, "language_style": {"answer": "Tactful and warm", "rationale": "The language style of the entry is euphemism. It is more friendly and interactive by speaking in a gentle and inviting manner, asking the interviewee whether he is satisfied with himself, rather than directly speaking or ordering. At the same time, the second person's word, \"you,\" is used, adding a sense of affection and friendship, making it easier for the interviewee to accept and answer questions."}, "time_dimension": {"answer": "Present", "rationale": "So the time dimension is now judged."}, "sentence_type": "Interrogative sentence", "pos": {"nouns": {"self": 1}, "verbs": {"Satisfied": 1, "- You're not": 1}}}, {"content": "How was your relationship with your family?", "word_count": 10, "label": "", "sentence_structure": "Subject鈥損redicate", "subjective_objective": {"answer": "Subjective", "rationale": "Because the question is about the patient ' s subjective feelings about his/her family, it is about the patient ' s personal views and attitudes."}, "sentence_sentiment": {"answer": "Positive", "rationale": "Because the adjective \"happy\" in the entry expresses a positive emotional attitude."}, "language_style": {"answer": "Tactful and warm", "rationale": ""}, "time_dimension": {"answer": "Present", "rationale": "The entry asks the testee ' s current state of affairs, i.e. whether the current relationship with the family is pleasant, so the time dimension is now judged."}, "sentence_type": "Interrogative sentence", "pos": {"nouns": {"Family": 1, "Members": 1}, "verbs": {"Relations": 1, "Have a nice day": 1}}}, {"content": "Did you have a good relationship with your parents?", "word_count": 10, "label": "", "sentence_structure": "Subject鈥損redicate", "subjective_objective": {"answer": "Subjective", "rationale": "Because the feeling of \"good relationships\" is a subjective assessment of the patient."}, "sentence_sentiment": {"answer": "Positive", "rationale": "Because the \"Friendly Relations\" attitude in the entries is positive."}, "language_style": {"answer": "Tactful and warm", "rationale": "The language style of the entry is euphemistic. It asks the respondent about his parent relationship in a gentle and inviting manner, rather than whether the other is satisfied or not. It respects the person's privacy and makes him more willing to share his thoughts and feelings. Moreover, the description of \"happy\" gives the person surveyed a positive and positive impression that he or she will be more motivated to participate in the questionnaire."}, "time_dimension": {"answer": "Present", "rationale": "The entry asks about the situation of the person being tested in relation to his parents, the key word \"relationship with his parents\" is an indication of the current state, and \"Happy?\" further clarifies the time dimension of the issue as it is."}, "sentence_type": "Interrogative sentence", "pos": {"nouns": {"you": 1, "parents": 1, "Relations": 1, "Have a nice day": 1}, "verbs": {"and": 1, "have": 1}, "adjectives": {"- You're not": 1}, "numerals": {"21": 1}}}, {"content": "22, are you satisfied with your personal relationship?", "word_count": 12, "label": "", "sentence_structure": "Subject鈥搗erb鈥搊bject", "subjective_objective": {"answer": "Subjective", "rationale": "Because it is the subjective evaluation and perception of the patient ' s personal relationship."}, "sentence_sentiment": {"answer": "Neutral", "rationale": "Because the entry does not have any emotional color."}, "language_style": {"answer": "Tactful and warm", "rationale": "The language style of the entry is more gentle and friendly, and it leads the interviewee to think and express his/her views in a question-and-answer manner, without too much criticism or criticism. At the same time, the question is more closely related to personal feelings and feelings, and can easily trigger a resonance and in-depth response from the interviewee."}, "time_dimension": {"answer": "Present", "rationale": "The entry asks about the current situation, i.e. the person tested is satisfied with his or her personal relationship and thus judged as \"now\"."}, "sentence_type": "Interrogative sentence", "pos": {"nouns": {"Human relations": 1, "Satisfied": 1}, "verbs": {"self": 1}, "adverbs": {"- You're not": 1}, "numerals": {"22": 1}}}, {"content": "Do you have any friends who believe in them?", "word_count": 10, "label": "", "sentence_structure": "Subject鈥搗erb鈥搊bject", "subjective_objective": {"answer": "Subjective", "rationale": "Because \"have a fully trusted friend\" is a personal feeling and perception, a subjective examination."}, "sentence_sentiment": {"answer": "Neutral", "rationale": "Since the entry is an objective one, it does not involve personal feelings or attitudes and is therefore neutral."}, "language_style": {"answer": "Tactful and warm", "rationale": "The entry is more personal and interactive when it is being approached in a gentle and inviting manner, asking the interviewees whether they have trusted friends, rather than direct statements or orders."}, "time_dimension": {"answer": "Present", "rationale": "The entry asks whether the person being tested currently has a fully trusted friend, and therefore the time dimension is now judged."}, "sentence_type": "Interrogative sentence", "pos": {"nouns": {"friends": 1, "Total": 1}, "verbs": {"have": 1, "Letter": 1}, "adverbs": {"- You're not": 1}, "numerals": {"23": 1}}}, {"content": "Are you satisfied with your support from your friends?", "word_count": 12, "label": "", "sentence_structure": "Subject鈥搗erb鈥搊bject", "subjective_objective": {"answer": "Subjective", "rationale": "Because the problem is viewed as the patient ' s satisfaction with his friends ' support and as a subjective feeling."}, "sentence_sentiment": {"answer": "Positive", "rationale": "This indicates a positive emotional attitude, i.e. a positive relationship of expectation and support."}, "language_style": {"answer": "Calm and direct", "rationale": ""}, "time_dimension": {"answer": "Present", "rationale": "The entry is for the current feelings and assessment of the subject, and the keyword is \"you\" and \"satisfaction\", which point to the behaviour or state of the person in question."}, "sentence_type": "Interrogative sentence", "pos": {"nouns": {"friends": 1, "Support": 1}, "verbs": {"Here": 1, "Jean": 1, "Satisfied": 1}, "numerals": {"24": 1}}}, {"content": "- 25, do you like being with your friends?", "word_count": 12, "label": "", "sentence_structure": "Subject鈥搗erb鈥搊bject", "subjective_objective": {"answer": "Subjective", "rationale": "Because \"like\" is an individual's feeling or attitude, and it's subjective."}, "sentence_sentiment": {"answer": "Positive", "rationale": "Because the attitude of \"like being with friends\" in the entry is positive."}, "language_style": {"answer": "Tactful and warm", "rationale": "The entry uses a gentle, inquisitive tone, rather than direct command or statement, which is more personal and interactive. At the same time, the word \"like\" expresses concern and concern about the feelings of the other."}, "time_dimension": {"answer": "Present", "rationale": "The entry asks about the person's current behaviour or feelings, the word \"like\" implies that it is a subjective attitude or emotional experience, while \"friends with friends\" is a specific scene that indicates that such behaviour or feeling is related to a particular group of people (friends). Thus, in terms of time dimension, the entry is now."}, "sentence_type": "Interrogative sentence", "pos": {"nouns": {"you": 1, "friends": 1, "Entry": 1, "Together": 1}, "verbs": {"Yeah": 1, "in": 1}, "numerals": {"25": 1}, "classifiers": {"individual": 1}}}, {"content": "Is there a sense of security when you go home?", "word_count": 11, "label": "", "sentence_structure": "Subject鈥損redicate", "subjective_objective": {"answer": "Subjective", "rationale": "Because security is a personal feeling and experience, it is subjective."}, "sentence_sentiment": {"answer": "Positive", "rationale": "Because the attitude of \"home with a sense of security\" in the entry is positive."}, "language_style": {"answer": "Tactful and warm", "rationale": "The expression \"home\" itself is a certain emotional expression that allows the interviewee to feel friendly and familiar, so that the language style of the entry is more euphemistic and warm."}, "time_dimension": {"answer": "Present", "rationale": "The entry asks the testee's current feelings and experiences, and the keyword is \"there's a sense of security\" which means that it's a state of immediate concern or emotion. So the temporal dimension is now."}, "sentence_type": "Interrogative sentence", "pos": {"nouns": {"Home": 1, "Safe": 1}, "verbs": {"Back": 1, "Yes": 1}, "adjectives": {"One": 1}, "adverbs": {"- You're not": 1}, "numerals": {"26": 1}}}, {"content": "Are you happy with your family?", "word_count": 12, "label": "", "sentence_structure": "Subject鈥搗erb鈥搊bject", "subjective_objective": {"answer": "Subjective", "rationale": "This question is about the patient鈥檚 feelings or experiences, i.e. subjective feelings. While this feeling may be influenced by objective factors such as family relationships, the environment, etc., the problem itself is intended to understand the patient鈥檚 emotional response."}, "sentence_sentiment": {"answer": "Positive", "rationale": "Because the \"favorite\" act is positive."}, "language_style": {"answer": "Tactful and warm", "rationale": "The entry is a soft, inviting expression of dialogue, asking if the other is happy with his family, which is more personal and interactive. At the same time, the phrase \"Feel happy\" is subjective and focuses more on personal feelings."}, "time_dimension": {"answer": "Present", "rationale": ""}, "sentence_type": "Interrogative sentence", "pos": {"nouns": {"you": 1, "Family": 1, "Happiness": 1}, "verbs": {"in": 1, "Together": 1, "Sensor": 1, "Present": 1, "- You're not": 1}, "numerals": {"27": 1}, "pronouns": {"you": 1}}}, {"content": "28, do you like your life at school?", "word_count": 10, "label": "", "sentence_structure": "Subject鈥搗erb鈥搊bject", "subjective_objective": {"answer": "Subjective", "rationale": "Because like a subjective feeling of a person is related to the emotions and attitudes of the individual."}, "sentence_sentiment": {"answer": "Positive", "rationale": "Because the word \"likes\" expresses a positive emotional attitude."}, "language_style": {"answer": "Tactful and warm", "rationale": "The more moderate tone of the article, the use of the questionword and the use of \"you\" as the main word, make it easier for the interviewees to accept and be willing to answer questions and thus more personal and interactive in their expression."}, "time_dimension": {"answer": "Present", "rationale": "The entry asks the current state and feelings of the testee, the keyword being \"like\" and \"life in school\", all of which point to the activities or state that are being carried out."}, "sentence_type": "Interrogative sentence", "pos": {"nouns": {"Questionnaire": 1, "Entry": 1, "School": 1, "Life": 1}, "verbs": {"Yeah": 1}, "numerals": {"28": 1}}}, {"content": "Are there any people who would like to listen to your thoughts?", "word_count": 13, "label": "", "sentence_structure": "Subject鈥搗erb鈥搊bject", "subjective_objective": {"answer": "Subjective", "rationale": "Because the existence of a person willing to listen to one鈥檚 thoughts is a subjective feeling and judgement of one person, depending on their personal experience and personal relationships. While this may be influenced by objective circumstances, such as the existence or absence of social support systems, the ultimate subjective view is left to the individual."}, "sentence_sentiment": {"answer": "Positive", "rationale": "Because it expresses a positive quality, i.e., whether anyone can listen to themselves."}, "language_style": {"answer": "Tactful and warm", "rationale": "The language style of the entry is euphemismal. It uses a more intimate, intimate tone to ask questions, to express concern and respect for the feelings that are deep inside the interviewee, and to try to build a relationship of mutual trust."}, "time_dimension": {"answer": "Present", "rationale": "The keyword \"there\" and \"persons willing to listen to your thoughts\" indicate that this is an inquiry into the state of affairs."}, "sentence_type": "Interrogative sentence", "pos": {"nouns": {"you": 1, "Something's on the mind": 1, "person": 1}, "verbs": {"have": 1, "May I": 1, "Listen": 1}, "adverbs": {"More": 1, "- You're not": 1}, "numerals": {"29": 1}}}, {"content": "Do you have enough money?", "word_count": 6, "label": "", "sentence_structure": "Subject鈥損redicate", "subjective_objective": {"answer": "Subjective", "rationale": "Because the problem is examined by the patient ' s subjective feelings and experiences, that is, the perception of whether he or she has enough money to meet the needs of life."}, "sentence_sentiment": {"answer": "Neutral", "rationale": "Because the entry is about the financial situation of a person and does not involve positive or negative emotional aspects."}, "language_style": {"answer": "Tactful and warm", "rationale": "The entry is asked in a way that gives a sense of affection and care, unlike direct orders or statements, which are as a matter of indifference or depression. Moreover, the language of the article is simpler to understand and does not cause unnecessary distress or stress to the person interviewed."}, "time_dimension": {"answer": "Present", "rationale": "The keyword \"Are you well-paid?\" is to ask the testee about his current state and circumstances, without reference to past or future behaviour or mentality and therefore belongs to the present."}, "sentence_type": "Interrogative sentence", "pos": {"nouns": {"Money": 1, "Use": 1}, "verbs": {"That's enough": 1}, "adverbs": {"That's enough": 1}, "numerals": {"30": 1}}}, {"content": "- You still have your parents' money?", "word_count": 13, "label": "", "sentence_structure": "Subject鈥搗erb鈥搊bject", "subjective_objective": {"answer": "Objective", "rationale": "Since the identity of the provider of the cost of living is an objective fact, it is not influenced by the subjective consciousness of the person under investigation."}, "sentence_sentiment": {"answer": "Neutral", "rationale": "Because the entry is merely a question of fact and does not involve emotional attitudes or behaviour."}, "language_style": {"answer": "Calm and direct", "rationale": "The language style of the entry is relatively simple, and the question is directly related to the source of the cost of living of the person surveyed, which is more objective and practical than the use of excessive rhetorical or emotional colours."}, "time_dimension": {"answer": "Present", "rationale": "The entry asks the status of the testees, that is, their source of living, and can therefore be judged as \"now\"."}, "sentence_type": "Interrogative sentence", "pos": {"nouns": {"Cost of living": 1, "parents": 1}, "verbs": {"Provision": 1, "And": 1}, "adverbs": {"- You're not": 1}, "numerals": {"31": 1}, "pronouns": {"you": 1}}}, {"content": "Do you feel guilty about your parents providing for your own living?", "word_count": 16, "label": "", "sentence_structure": "Subject鈥搗erb鈥搊bject", "subjective_objective": {"answer": "Subjective", "rationale": "Because guilt is a subjective emotional feeling, a patient's observation and description of his own inner state."}, "sentence_sentiment": {"answer": "Negative", "rationale": "Because the \"guilty\" emotion in the entry is negative."}, "language_style": {"answer": "Tactful and warm", "rationale": "This entry is more personal and interactive."}, "time_dimension": {"answer": "Present", "rationale": "The entry asks the testee about his current feelings or attitudes, the key word being \"you provide his parents with a living allowance\", and it describes the current situation, not the past or the future.\""}, "sentence_type": "Interrogative sentence", "pos": {"nouns": {"32": 1, "parents": 1, "Living on your own": 1, "Guilt": 1}, "verbs": {"Provision": 1, "have": 1, "I feel": 1}, "numerals": {"32": 1, "One": 1}, "classifiers": {"Living on your own": 1}, "pronouns": {"you": 1}}}, {"content": "33 - Are you satisfied with your sex life?", "word_count": 11, "label": "", "sentence_structure": "Subject鈥搗erb鈥搊bject", "subjective_objective": {"answer": "Subjective", "rationale": "Because \"satisfaction\" is a subjective feeling and evaluation that requires the patient to feel and judge himself."}, "sentence_sentiment": {"answer": "Negative", "rationale": "The issue could trigger negative emotional reactions because it involved personal privacy and sensitive topics."}, "language_style": {"answer": "Tactful and warm", "rationale": "The entry is asked in a relatively mild manner, avoiding an overly direct or offensive way of questioning, while taking into account the privacy and sensitivity of the person under investigation, and being more humane and respectful."}, "time_dimension": {"answer": "Present", "rationale": "The entry is an inquiry into the current sexual satisfaction of the testee, and therefore the time dimension is now judged."}, "sentence_type": "Interrogative sentence", "pos": {"nouns": {"self": 1, "Sex": 1}, "verbs": {"Satisfied": 1}, "numerals": {"33": 1}}}, {"content": "- Are you having a high sexual frequency?", "word_count": 9, "label": "", "sentence_structure": "Subject鈥搗erb鈥搊bject", "subjective_objective": {"answer": "Subjective", "rationale": "Because \"sexuality frequency\" is an individual's privacy and belongs to the subjective sense and experience of the individual."}, "sentence_sentiment": {"answer": "Negative", "rationale": "The problem is one of personal privacy and some negative emotional aspects."}, "language_style": {"answer": "Calm and direct", "rationale": "The language style of the entry is more straightforward, there is no over-modified or euphemistic formulation, which directly asks about the sex frequency of the other person, and this expression is more direct and objective."}, "time_dimension": {"answer": "Present", "rationale": ""}, "sentence_type": "Interrogative sentence", "pos": {"nouns": {"Sex": 1, "Frequency": 1}, "verbs": {"High": 1, "- You're not": 1}, "numerals": {"34": 1}}}, {"content": "Will it take much time to communicate online?", "word_count": 14, "label": "", "sentence_structure": "Subject鈥搗erb鈥搊bject", "subjective_objective": {"answer": "Subjective", "rationale": "Because \"it takes a lot of time\" and \"to communicate with people online\" are the patient's own actions and ideas, and therefore the subjective perspective is being examined."}, "sentence_sentiment": {"answer": "Positive", "rationale": "Because the behaviour in the entry \"to spend a lot of time communicating with people online\" can be considered as a positive behaviour, which shows that individuals are happy to interact with others in society."}, "language_style": {"answer": "Calm and direct", "rationale": "The entry is more straightforward in language style, has no excessive speech or emotional rendering, and is more objective in tone. It specifically asks whether the respondents spend a significant amount of time communicating with people online, expressing the surveyers鈥?intentions and concerns."}, "time_dimension": {"answer": "Present", "rationale": "Now, based on the title \"It's gonna take a lot of time\" and \"Weet-to-people\" it can be judged to be a question of describing the current behaviour or habits of the testee, so the time dimension is now."}, "sentence_type": "Interrogative sentence", "pos": {"nouns": {"Time": 1, "Online": 1, "person": 1, "Communications": 1}, "verbs": {"Flowers": 1, "and": 1}, "adjectives": {"A lot": 1}, "adverbs": {"very": 1}, "numerals": {"35": 1}}}, {"content": "Do you like web communication better than face-to-face?", "word_count": 16, "label": "", "sentence_structure": "Subject鈥搗erb鈥搊bject", "subjective_objective": {"answer": "Subjective", "rationale": "Because \"like\" is a subjective attitude and emotion."}, "sentence_sentiment": {"answer": "Positive", "rationale": "Because the attitude of \"likes to communicate online more than to talk face to face\" in the entries is positive."}, "language_style": {"answer": "Calm and direct", "rationale": "The language style of the entry is more objective and straightforward, there is no excessive emotional accoutrements or rhetorical techniques, and the problem is expressed more clearly, allowing the respondents to understand the meaning of the problem clearly."}, "time_dimension": {"answer": "Present", "rationale": "The entry asks the testee about his current preferences and attitudes, the keywords being \"like,\" \"network communication\" and \"face-to-face conversation\", all pointing to the current state."}, "sentence_type": "Interrogative sentence", "pos": {"nouns": {"Network communication": 1, "Face-to-face": 1}, "verbs": {"Yeah": 1, "More than anything": 1, "Talk": 1}, "numerals": {"36": 1}}}, {"content": "Do you like people watching you on your social software?", "word_count": 16, "label": "", "sentence_structure": "Subject鈥搗erb鈥搊bject", "subjective_objective": {"answer": "Subjective", "rationale": "Because \"like\" is the patient's personal feeling and attitude, and it's subjective."}, "sentence_sentiment": {"answer": "Positive", "rationale": "Because the word \"like\" is a positive attitude."}, "language_style": {"answer": "Tactful and warm", "rationale": "The language style of the entry is more euphemistic, and dialogue is conducted by asking the other person about the attitude or preferences of the other person, avoiding direct orders or evaluation."}, "time_dimension": {"answer": "Present", "rationale": ""}, "sentence_type": "Interrogative sentence", "pos": {"nouns": {"Questionnaire": 1, "Entry": 1, "person": 1, "Social": 1, "Software": 1, "Attention": 1}, "verbs": {"Yeah": 1, "in": 1, "Go, go, go": 2}, "numerals": {"37": 1}, "pronouns": {"you": 1}}}, {"content": "Do you like your dynamics being commented on in your social software?", "word_count": 19, "label": "", "sentence_structure": "Subject鈥搗erb鈥搊bject", "subjective_objective": {"answer": "Subjective", "rationale": "Because it is a subjective emotion and attitude of one who likes or dislikes comments from others."}, "sentence_sentiment": {"answer": "Positive", "rationale": "Because the word \"like\" in the entry is a positive emotional attitude."}, "language_style": {"answer": "Tactful and warm", "rationale": "The language style of the entry is more euphemistic, not directly demanding or ordering, but rather a gentle way to ask the interviewee about the attitude of others to their own social software. Moreover, the entry uses more friendly language, such as \"you\", rather than \"you,\" which makes the questionnaire closer to the interviewee and enhances his participation."}, "time_dimension": {"answer": "Present", "rationale": "According to the title, \"Does anyone like to comment on your dynamics on your social software?\" The question is asking the testee what he thinks and feels, so the answer is \"now.\""}, "sentence_type": "Interrogative sentence", "pos": {"nouns": {"Questionnaire": 1, "Entry": 1, "38": 1, "others": 1, "Social software": 1, "Dynamic": 1}, "verbs": {"Yeah": 1, "Commenting": 1}, "numerals": {"38": 1}, "pronouns": {"you": 2}}}, {"content": "- You like your family watching your movements?", "word_count": 14, "label": "", "sentence_structure": "Subject鈥搗erb鈥搊bject", "subjective_objective": {"answer": "Subjective", "rationale": "Because \"like\" is a subjective attitude that reflects the patient's need and preferences for the attention of his family. And \"family attention to your dynamics\" is an objective fact or behaviour. So the subject is about the patient's subjective feelings and needs."}, "sentence_sentiment": {"answer": "Neutral", "rationale": "It is asking if the interviewees want their families to follow their movements."}, "language_style": {"answer": "Tactful and warm", "rationale": "The entry is more personal and interactive in terms of a gentle and inviting tone of conversation, asking if the other side wishes to have the interest and concern of the family."}, "time_dimension": {"answer": "Present", "rationale": "The entry is a question about the attitude and feelings of the testee to his or her family ' s concern for his or her dynamics, and the time dimension is now judged."}, "sentence_type": "Interrogative sentence", "pos": {"nouns": {"you": 1, "Family": 1, "Attention": 1, "Dynamic": 1}, "verbs": {"Yeah": 1}, "adverbs": {"- You're not": 1}, "numerals": {"39": 1}}}, {"content": "Are you worried about school safety?", "word_count": 14, "label": "", "sentence_structure": "Subject鈥搗erb鈥搊bject", "subjective_objective": {"answer": "Subjective", "rationale": "This is because of patients ' subjective perception and perception of safety at school campuses."}, "sentence_sentiment": {"answer": "Negative", "rationale": "Because the \"fear\" in the entry is a negative emotional attitude."}, "language_style": {"answer": "Tactful and warm", "rationale": "The entry is more softly phrased in terms of the way in which the view and perception of the other side about the safety of the school campus is expressed, which is more personal and interactive. At the same time, the word \"concern\" does not directly express negative sentiments, but rather raises questions in a more neutral manner, allowing the interviewee to be more willing to share his real thoughts."}, "time_dimension": {"answer": "Present", "rationale": "The entry asks the testee about his current attitude and perception of safety on the school campus, and the temporal dimension is now judged. \""}, "sentence_type": "Interrogative sentence", "pos": {"nouns": {"School": 1, "Campus": 1, "Clear": 1, "Problem": 1}, "verbs": {"Fear": 1, "- You're not": 1, "EMPTY": 1}, "numerals": {"1": 1}}}, {"content": "Do you feel safe?", "word_count": 10, "label": "", "sentence_structure": "Subject鈥搗erb鈥搊bject", "subjective_objective": {"answer": "Subjective", "rationale": "Because \"safe\" is the patient's subjective feeling and experience."}, "sentence_sentiment": {"answer": "Positive", "rationale": "Since the purpose of the article is affirmative, the person interviewed is not asked about his or her self-perception, which is not negative."}, "language_style": {"answer": "Tactful and warm", "rationale": "The entry, which guides the interviewee to the question of self-security, is more gentle and friendly, is not too direct or command-oriented, and makes it easier for the interviewee to relax and share his thoughts."}, "time_dimension": {"answer": "Present", "rationale": "The entry asks about the current perception of the testee, and the time dimension is now judged."}, "sentence_type": "Interrogative sentence", "pos": {"nouns": {"Clear": 1, "Sensor": 1}, "verbs": {"I think": 1, "have": 1}, "adverbs": {"From": 1}, "pronouns": {"you": 1}}}, {"content": "How is your school environment?", "word_count": 14, "label": "", "sentence_structure": "Subject鈥搗erb鈥搊bject", "subjective_objective": {"answer": "Objective", "rationale": "Because \"school environment\" is an objective thing, \"how is satisfaction?\" is evaluated from the point of viewer's point of view, and the entry is therefore viewed from an objective point of view."}, "sentence_sentiment": {"answer": "Positive", "rationale": "Because \"satisfaction\" is a positive word."}, "language_style": {"answer": "Calm and direct", "rationale": "The entry is a direct question to the respondents about their satisfaction with the school environment, the language style being simpler and less emotionally qualified, and the language style being a calm and straightforward one."}, "time_dimension": {"answer": "Present", "rationale": "The entry asks the testee's current feelings and views, the keyword being \"how satisfied\" and all pointing to the situation. So the answer is \"now\"."}, "sentence_type": "Interrogative sentence", "pos": {"nouns": {"School": 1, "Campus": 1, "Environment": 1, "Satisfaction": 1}, "verbs": {"How's that": 1, "to": 1, "Satisfied": 1, "Evaluation": 1}, "adjectives": {"of": 1}, "numerals": {"3": 1}}}, {"content": "Does it always make you feel easier to be comfortable?", "word_count": 15, "label": "", "sentence_structure": "Subject鈥搗erb鈥搊bject", "subjective_objective": {"answer": "Subjective", "rationale": "Because comfort is a subjective feeling and experience, different people may define comfort differently."}, "sentence_sentiment": {"answer": "Positive", "rationale": "Because the expression \"comfortful environment always makes you feel easier\" in the entry is positive."}, "language_style": {"answer": "Tactful and warm", "rationale": "The entry uses a more euphemistic and friendly approach to questions, without using language that is too direct or commandive to make people feel more comfortable and comfortable."}, "time_dimension": {"answer": "Present", "rationale": "The entry asks the testee how he feels about the comfort, the keywords are \"always\" and \"favoritely\" and they point to the state of the moment. So the judgment is now."}, "sentence_type": "Interrogative sentence", "pos": {"nouns": {"Environment": 1, "Comfortable": 1, "Feel": 1, "Easy": 1}, "verbs": {"Jean": 1}, "adjectives": {"More": 1, "- Yeah": 1}, "adverbs": {"Always": 1}, "pronouns": {"you": 1}}}, {"content": "How comfortable is your dorm?", "word_count": 10, "label": "", "sentence_structure": "Subject鈥損redicate", "subjective_objective": {"answer": "Subjective", "rationale": "Because comfort is a subjective feeling or an evaluation, not an objective fact."}, "sentence_sentiment": {"answer": "Neutral", "rationale": "Because it does not address emotional attitudes or behavioural reactions."}, "language_style": {"answer": "Tactful and warm", "rationale": "The entry is in the form of a question sentence, which is a soft invitation to ask the other side whether the residential experience is comfortable and without direct orders or criticism, and which is more easily accepted and willing to share their own ideas."}, "time_dimension": {"answer": "Present", "rationale": "The entry asks about the current residential experience of the person tested, and the keyword \"Is your dormitory comfortable\" indicates that it is an inquiry into the current situation."}, "sentence_type": "Interrogative sentence", "pos": {"nouns": {"Accommodation": 1, "Comfortable": 1}, "verbs": {"Stay": 1}, "adverbs": {"More": 1, "not": 1}, "numerals": {"5": 1}, "classifiers": {"individual": 1, "Sample": 1}}}, {"content": "6. Are you satisfied with the construction of public facilities in the school?", "word_count": 16, "label": "", "sentence_structure": "Subject鈥搗erb鈥搊bject", "subjective_objective": {"answer": "Subjective", "rationale": "Since \"satisfaction\" is a subjective feeling or perception that reflects the opinion of the interviewees regarding the public facilities of the school, the entry on the questionnaire is viewed from a subjective perspective."}, "sentence_sentiment": {"answer": "Positive", "rationale": "Because the attitude of \"satisfaction with the construction of public facilities in schools\" is positive."}, "language_style": {"answer": "Tactful and warm", "rationale": "The entry allows respondents to self-assess their satisfaction with the school public facilities by asking questions, does not directly express an assessment or bias in this regard, and therefore is more euphemistic and moderate."}, "time_dimension": {"answer": "Present", "rationale": ""}, "sentence_type": "Interrogative sentence", "pos": {"nouns": {"School": 1, "Public facilities": 1}, "verbs": {"Construction": 1, "I feel": 1, "Satisfied": 1}, "adverbs": {"- You're not": 1}, "numerals": {"6": 1}}}, {"content": "Are you satisfied with your own financial situation?", "word_count": 12, "label": "", "sentence_structure": "Subject鈥搗erb鈥搊bject", "subjective_objective": {"answer": "Subjective", "rationale": "This is because it involves a subjective assessment of the patient ' s financial situation."}, "sentence_sentiment": {"answer": "Neutral", "rationale": "Because the entry does not have any emotional color."}, "language_style": {"answer": "Tactful and warm", "rationale": ""}, "time_dimension": {"answer": "Present", "rationale": "The entry can be judged by the key word in the title, \"your present economic situation\", and the time dimension is now judged by the testee's satisfaction with his or her economic situation."}, "sentence_type": "Interrogative sentence", "pos": {"nouns": {"Economic conditions": 1, "Satisfied": 1}, "verbs": {"be": 1}, "adverbs": {"self": 1, "- You're not": 1}}}, {"content": "Do you often worry about your financial situation?", "word_count": 16, "label": "", "sentence_structure": "Subject鈥搗erb鈥搊bject", "subjective_objective": {"answer": "Subjective", "rationale": "Because the entry asks whether the patient is often anxious and worried, which is the patient ' s subjective feeling."}, "sentence_sentiment": {"answer": "Negative", "rationale": "Because the emotional attitude of \"anger\" in the entry is negative."}, "language_style": {"answer": "Tactful and warm", "rationale": ""}, "time_dimension": {"answer": "Present", "rationale": "The entry uses the term \"often\" to indicate a regular act, so the temporal dimension is judged to be the present. At the same time, the expression \"for ...\" also indicates a continuing state of affairs, so that the temporal dimension of the article can be confirmed as the present."}, "sentence_type": "Interrogative sentence", "pos": {"nouns": {"Status": 1, "Fear": 1, "Anxiety": 1}, "verbs": {"Regular": 1, "Yes": 1, "- Yes, sir": 1, "Consider": 1}, "adjectives": {"The long run": 1, "- Yeah": 1}, "adverbs": {"Often": 1}}}, {"content": "Are you happy because of stable economic conditions?", "word_count": 15, "label": "", "sentence_structure": "Subject鈥搗erb鈥搊bject", "subjective_objective": {"answer": "Subjective", "rationale": "Because the problem involves subjective perceptions and perceptions of the relationship between stable economic conditions and happiness. Although economic conditions are objective, people鈥檚 feelings and perceptions are subjective."}, "sentence_sentiment": {"answer": "Neutral", "rationale": "The entry is concerned with the impact of economic conditions on well-being and does not contain any positive or negative emotional aspects."}, "language_style": {"answer": "Calm and direct", "rationale": "Since the question is a clear inquiry, without any supposition or euphemism, it is possible to judge the language style as being calm and straightforward by directly asking the views and feelings of the person under investigation."}, "time_dimension": {"answer": "Present", "rationale": ""}, "sentence_type": "Interrogative sentence", "pos": {"nouns": {"Economic conditions": 1, "Happiness": 1}, "verbs": {"Yes": 2, "Jean": 1, "Feel": 1}, "adjectives": {"Steady": 1}, "classifiers": {"you": 1, "Sensor": 1}}}, {"content": "Are you easily receptive to new information, new knowledge?", "word_count": 13, "label": "", "sentence_structure": "Subject鈥搗erb鈥搊bject", "subjective_objective": {"answer": "Subjective", "rationale": "It is subjective because of the ease with which new information and knowledge are received, as it is an individual ' s feeling and attitude."}, "sentence_sentiment": {"answer": "Positive", "rationale": "Because the \"Easier to accept new information, new knowledge\" attitude in the entries is positive."}, "language_style": {"answer": "Tactful and warm", "rationale": "The entry is in the form of a question-and-answer period, with a more moderate tone and a more euphemistic expression, giving a sense of affection and concern, aimed at understanding the character characteristics of the person under investigation, rather than direct orders or statements. At the same time, the expression \"easy to receive new information, new knowledge\" is also neutral and objective, and does not put undue pressure or discomfort on the person under investigation."}, "time_dimension": {"answer": "Present", "rationale": "The entry asks the testee about his current behaviour or state, i.e. whether he/she is easily receptive to new information, knowledge. The time dimension is therefore judged as \"now\"."}, "sentence_type": "Interrogative sentence", "pos": {"nouns": {"Questionnaire": 1, "Entry": 1, "Can not open message": 1, "New knowledge": 1}, "verbs": {"Accept": 1, "Give": 1}, "adjectives": {"Easy": 1}, "numerals": {"10": 1}, "classifiers": {"individual": 1}}}, {"content": "Is it hard for you to have a new skill?", "word_count": 15, "label": "", "sentence_structure": "Subject鈥搗erb鈥揷omplement", "subjective_objective": {"answer": "Subjective", "rationale": "Because difficulty is a personal feeling and evaluation, it is viewed from the patient ' s subjective perspective."}, "sentence_sentiment": {"answer": "Neutral", "rationale": "Because the entry is asking about objective facts (i.e. the difficulty of learning new skills) and does not express any positive or negative emotional colours."}, "language_style": {"answer": "Tactful and warm", "rationale": "The entry is interactive and is neutral, using the term \"capable of a new skill\", without directly expressing subjective judgements about the magnitude of the difficulty, and with a greater focus on interaction and understanding with the person under investigation. At the same time, \"Are you having a lot of difficulty?\" The phrase is more gentle and friendly, and it is easy to trigger positive answers and sharing by the person under investigation."}, "time_dimension": {"answer": "Present", "rationale": "The entry asks the testee about the current situation, i.e. whether learning new skills is considered to be more difficult for himself and therefore the time dimension is now judged."}, "sentence_type": "Interrogative sentence", "pos": {"nouns": {"Door": 1, "Skills": 1, "you": 1}, "verbs": {"Got it": 1, "It's a lot of trouble": 1}, "adjectives": {"New": 1}, "numerals": {"One": 1}}}, {"content": "Will the acquisition of new skills bring you a sense of accomplishment?", "word_count": 14, "label": "", "sentence_structure": "Subject鈥搗erb鈥搊bject", "subjective_objective": {"answer": "Subjective", "rationale": "Because \"a sense of achievement\" is a subjective feeling, different people may have different feelings and experiences. The purpose of this questionnaire is to examine patients' subjective perceptions of new skills acquired and therefore to be viewed from a subjective perspective."}, "sentence_sentiment": {"answer": "Positive", "rationale": "Because the sense of achievement in the entry is a positive emotional experience."}, "language_style": {"answer": "Tactful and warm", "rationale": "The entry, in a gentle and inviting manner, asks the interviewees whether the acquisition of new skills will bring about a sense of achievement, without using any cold, direct or accusatory language. This expression is more likely to bring the respondents closer to sharing their real feelings and experiences."}, "time_dimension": {"answer": "Future", "rationale": "The entry contains visions and expectations for the future, and the keywords are \"new skills acquisition\" and \"a sense of achievement\", all of which point to situations that have not yet occurred and that may arise in the future."}, "sentence_type": "Interrogative sentence", "pos": {"nouns": {"New skills": 1, "A sense of achievement": 1}, "verbs": {"鈾?Bring it 鈾?: 1}, "adverbs": {"- You're not": 1}, "numerals": {"12": 1}}}, {"content": "13. Are you satisfied with your ability to learn new knowledge and skills?", "word_count": 18, "label": "", "sentence_structure": "Subject鈥搗erb鈥搊bject", "subjective_objective": {"answer": "Subjective", "rationale": "The question is examined from a subjective point of view, as it is an examination of the individual feelings and assessments of the patient."}, "sentence_sentiment": {"answer": "Positive", "rationale": "Because the word \"satisfaction\" is a positive emotional expression of a positive assessment of one's abilities."}, "language_style": {"answer": "Tactful and warm", "rationale": "The entry leads the respondents to think and express their views by asking questions, and uses more euphemistic language to avoid stress or discomfort. At the same time, this moderate tone makes the questionnaire more personal and interactive."}, "time_dimension": {"answer": "Present", "rationale": "The entry is for the purpose of asking the testee about his or her self-capacity and does not clearly indicate the time point of sexual orientation, and thus the judgement is now."}, "sentence_type": "Interrogative sentence", "pos": {"nouns": {"Capacity": 1, "self": 1, "New knowledge": 1, "New skills": 1}, "verbs": {"Learning": 1, "Satisfied": 1}, "adverbs": {"to": 1}, "numerals": {"13": 1}, "pronouns": {"you": 1}}}, {"content": "14. Do you have much access to new knowledge?", "word_count": 11, "label": "", "sentence_structure": "Subject鈥損redicate", "subjective_objective": {"answer": "Subjective", "rationale": "Because \"the way to acquire new knowledge\" is the patient's personal experience and habits, which are subjective observations."}, "sentence_sentiment": {"answer": "Neutral", "rationale": "According to the questionnaire, \"the means of acquiring new knowledge\" does not in itself carry any positive or negative emotional connotations. The emotional orientation of the purpose of the article is therefore neutral."}, "language_style": {"answer": "Tactful and warm", "rationale": "The entry, which guides the interviewee to reflect on the acquisition of new knowledge in a way that is more comfortable and acceptable to the interviewee than to direct statements or orders. At the same time, the use of the phrase \"does it\" makes the questionnaire more open and flexible, allowing the interviewee more space to answer questions."}, "time_dimension": {"answer": "Present", "rationale": "The entry asks whether the testee has a variety of ways to acquire new knowledge, and the time dimension is now judged."}, "sentence_type": "Interrogative sentence", "pos": {"nouns": {"you": 1, "New knowledge": 1, "Pathway": 1}, "verbs": {"Access": 1, "More": 1}, "adverbs": {"- You're not": 1}, "numerals": {"14": 1}}}, {"content": "Do you have a lot of opportunities for leisure and recreation?", "word_count": 14, "label": "", "sentence_structure": "Subject鈥搗erb鈥搊bject", "subjective_objective": {"answer": "Subjective", "rationale": "Because the chances are dependent on the patient ' s own feelings and experience, it is a subjective view."}, "sentence_sentiment": {"answer": "Positive", "rationale": "Because \"participating in leisure and recreation\" is an active activity."}, "language_style": {"answer": "Tactful and warm", "rationale": "The entry uses a more moderate tone of questioning, without a direct statement or order tone, but rather a dialogue in a question-and-answer format, which is more resonant and cooperative with the person under investigation, reflecting the respect and interest of the investigator."}, "time_dimension": {"answer": "Present", "rationale": "The entry asks the testee's current situation and feelings, and the time dimension is now judged."}, "sentence_type": "Interrogative sentence", "pos": {"nouns": {"you": 1, "Opportunities": 1, "Activities": 1, "Leisure and recreation": 1}, "verbs": {"Participation": 1, "More": 1}, "adverbs": {"very": 1}, "numerals": {"15": 1}}}, {"content": "How do you feel about your involvement in an event?", "word_count": 18, "label": "", "sentence_structure": "Subject鈥損redicate", "subjective_objective": {"answer": "Subjective", "rationale": "Because this is a question of asking the patient about his or her personal feelings and attitudes, the subject is examined for subjective knowledge and evaluation of the extent to which he or she is involved in the activity."}, "sentence_sentiment": {"answer": "Neutral", "rationale": "Because the entry does not have any emotional color."}, "language_style": {"answer": "Calm and direct", "rationale": "This language style is more appropriate for surveying questionnaires, as it is clear and can effectively capture the real feedback of the interviewee."}, "time_dimension": {"answer": "Present", "rationale": ""}, "sentence_type": "Interrogative sentence", "pos": {"nouns": {"Activities": 1, "self": 2}, "verbs": {"I think": 1, "Participation": 1}, "adjectives": {"How's it going": 1}, "adverbs": {"More": 1}, "numerals": {"16": 1}}}, {"content": "Do you like to take part in a recreational event?", "word_count": 14, "label": "", "sentence_structure": "Subject鈥搗erb鈥搊bject", "subjective_objective": {"answer": "Subjective", "rationale": "Because \"willing\" is an individual's subjective or emotional experience, reflecting the individual's attitude and tendency to engage in recreational activities."}, "sentence_sentiment": {"answer": "Positive", "rationale": "Because the \"yes\" attitude is positive, it means a positive attitude towards engaging in recreational activities."}, "language_style": {"answer": "Tactful and warm", "rationale": "The entry is in the form of a question-and-answer sentence, and the temperature of the speech and the absence of direct orders or coercion, which is more acceptable and willing to answer questions."}, "time_dimension": {"answer": "Present", "rationale": "The keywords \"willing\" and \"active participation\" indicate that the act is a current attitude and will, not a past experience or a future plan. So the temporal dimension is now."}, "sentence_type": "Interrogative sentence", "pos": {"nouns": {"you": 1, "One": 1, "Recreational activities": 1}, "verbs": {"I'd love to": 1, "Participation": 1, "Initiatives": 1, "Do it": 1}, "adverbs": {"- You're not": 1}, "classifiers": {"EMPTY": 1}}}, {"content": "18, does entertainment bring more joy to your life?", "word_count": 18, "label": "", "sentence_structure": "Subject鈥損redicate", "subjective_objective": {"answer": "Subjective", "rationale": "Because the subject is about how people feel and how they feel, whether recreational activities bring them more happiness. This is based on patients鈥?subjective perceptions and feelings."}, "sentence_sentiment": {"answer": "Positive", "rationale": "Because \"getting more happiness\" in the entry is a positive expression of the attitude or emotional colour that entertainment can have a positive impact on life."}, "language_style": {"answer": "Tactful and warm", "rationale": "The language style of the entry is euphemistic and warm, allowing the interviewee to share his views on recreational activities in a way that does not directly express his views or position, but rather leads him to answer questions in a caring and understanding manner. This way is more respectful of the subject's subjectivity and independence, and better understands their needs and ideas."}, "time_dimension": {"answer": "Present", "rationale": "The entry asks the respondents about the current situation, i.e. whether recreational activities bring more happiness to their lives."}, "sentence_type": "Interrogative sentence", "pos": {"nouns": {"Recreational activities": 1, "Life": 1, "Happy": 1}, "verbs": {"Bring it": 1, "have": 1}, "adjectives": {"More": 1}, "numerals": {"18": 1}, "pronouns": {"you": 1}}}, {"content": "19. Would it make you feel more presentable to participate in a recreational event?", "word_count": 17, "label": "", "sentence_structure": "Subject鈥搗erb鈥搊bject", "subjective_objective": {"answer": "Subjective", "rationale": "Because it is a subjective assessment and perception of what patients feel about themselves."}, "sentence_sentiment": {"answer": "Positive", "rationale": "Because the \"participate in a recreational activity\" in the entry is positive, and the \"get you more alive\" purpose is positive."}, "language_style": {"answer": "Tactful and warm", "rationale": "The language style of the entry is more euphemistic, and it guides the interviewer to think and expresses concern and understanding in his presentation. At the same time, the issue is more friendly and easy to communicate with and with the interviewee."}, "time_dimension": {"answer": "Present", "rationale": "The questionnaire entry asks whether the person currently believes that participating in a recreational event would make him feel more presentable, and thus the time dimension is now judged."}, "sentence_type": "Interrogative sentence", "pos": {"nouns": {"One": 1, "Recreational activities": 1, "Existence": 1}, "verbs": {"Jean": 1, "have": 1}, "adjectives": {"More": 1}, "adverbs": {"Yes": 1}, "numerals": {"19": 1}}}, {"content": "Are you satisfied with the transportation conditions around the school?", "word_count": 14, "label": "", "sentence_structure": "Subject鈥搗erb鈥搊bject", "subjective_objective": {"answer": "Subjective", "rationale": "Because \"satisfaction\" is a subjective feeling and attitude."}, "sentence_sentiment": {"answer": "Neutral", "rationale": "Since the entry is an objective one, it does not involve personal feelings or attitudes."}, "language_style": {"answer": "Tactful and warm", "rationale": "The language style of the entry is more euphemistic and gentle, using the \"Are you satisfied with?\" method of questioning, which expresses a sense of concern and respect for the other. At the same time, the description of \"schools around\" makes the questionnaire more relevant and geographically relevant, and makes the respondents feel more friendly and realistic."}, "time_dimension": {"answer": "Present", "rationale": "The entry asks the subject about his current feelings and attitudes, the keyword is \"you're satisfied\" and \"satisfaction\", which point to the situation."}, "sentence_type": "Interrogative sentence", "pos": {"nouns": {"School": 1, "Transport conditions": 1}, "verbs": {"Satisfied": 1}, "numerals": {"20": 1}}}, {"content": "Do your personal beliefs make your life more meaningful?", "word_count": 16, "label": "", "sentence_structure": "Subject鈥搗erb鈥搊bject", "subjective_objective": {"answer": "Subjective", "rationale": "Because \"making life more meaningful\" is the patient's subjective feeling and evaluation."}, "sentence_sentiment": {"answer": "Positive", "rationale": "Because the notion that \"personal belief makes life more meaningful\" is often considered a positive attitude or belief."}, "language_style": {"answer": "Tactful and warm", "rationale": "The entry is in the form of a question sentence, which is more gentle and open, gives the interviewee some space to think, and does not directly ask or order his answer. Moreover, the term \"more meaningful\" in the entry is also a certain emotional aspect, making the whole question phrase more personal."}, "time_dimension": {"answer": "Present", "rationale": "The entry is for the current personal views and feelings of the testee, and the time dimension is now judged."}, "sentence_type": "Interrogative sentence", "pos": {"nouns": {"Questionnaire": 1, "Entry": 1, "Personal": 1, "Faith": 1, "Life": 2, "Meaning": 1}, "verbs": {"Jean": 2, "have": 1}, "adjectives": {"More": 1, "Meaningful": 1}, "adverbs": {"More": 1}, "numerals": {"1": 2}, "pronouns": {"you": 1}}}, {"content": "2. Will your personal beliefs help you cope better with the stress of life?", "word_count": 22, "label": "", "sentence_structure": "Subject鈥搗erb鈥搊bject", "subjective_objective": {"answer": "Subjective", "rationale": "Because faith is the subjective belief and attitude of one person, it has a direct impact on the way individuals deal with stress. While belief may help some to better cope with stress, it depends on their personal beliefs and circumstances, and therefore is a subjective approach."}, "sentence_sentiment": {"answer": "Positive", "rationale": "Because the idea that personal beliefs can help you better cope with life stress is positive."}, "language_style": {"answer": "Tactful and warm", "rationale": "The entry leads to each other's thinking by asking questions and using the positive word \"you can\" in questions, creating an atmosphere of encouragement and support. At the same time, it avoids too direct or strong a tone, making the questionnaire more friendly and friendly."}, "time_dimension": {"answer": "Present", "rationale": "Keywords \"can\" and \"help\" and \"better response\" are all verbs that describe the behavior or state that is now being performed, so they're judged as now."}, "sentence_type": "Interrogative sentence", "pos": {"nouns": {"Questionnaire": 1, "Entry": 1, "Pressure": 1}, "verbs": {"Yes": 1, "Help": 1, "Response": 1, "Better": 1}, "adverbs": {"More": 1}, "numerals": {"2": 1}}}, {"content": "Can your faith give you more positive energy?", "word_count": 15, "label": "", "sentence_structure": "Subject鈥搗erb鈥搊bject", "subjective_objective": {"answer": "Subjective", "rationale": "Because faith and positive energy are both subjective feelings and attitudes of individuals."}, "sentence_sentiment": {"answer": "Positive", "rationale": "Because the expression \"can give you more positive energy\" is positive and positive."}, "language_style": {"answer": "Tactful and warm", "rationale": "The entry is more euphemistic, in the form of a question phrase and at the end of the sentence, the word \"does it\" gives a sense of affection and friendship. Moreover, the entry does not directly request or order, but rather asks the person under investigation in a gentle manner, making it easier for the person under investigation to accept and answer the question."}, "time_dimension": {"answer": "Present", "rationale": "The entry asks the testees about their current situation, i.e. whether they believe that their faith can bring positive energy to themselves. So, in terms of time dimension, it is now."}, "sentence_type": "Interrogative sentence", "pos": {"nouns": {"Faith": 1, "Energy": 1}, "verbs": {"Here": 1, "Yes": 1, "More": 2}, "adjectives": {"Jack": 1, "Extreme": 1}, "adverbs": {"- You're not": 1}, "numerals": {"3": 1}, "classifiers": {"individual": 1}, "pronouns": {"you": 1}}}, {"content": "4. Do you think your personal beliefs are important to your life?", "word_count": 20, "label": "", "sentence_structure": "Subject鈥搗erb鈥搊bject", "subjective_objective": {"answer": "Subjective", "rationale": "Because this is a question about the patient ' s own feelings, it is about the patient ' s perception of the importance of his or her beliefs in their lives."}, "sentence_sentiment": {"answer": "Neutral", "rationale": "Because the entry does not have any emotional color."}, "language_style": {"answer": "Tactful and warm", "rationale": "The entry is in the form of a question and a more euphemistic language when asking the other party's views, without giving a direct answer, either yes or no. This expression is more moderate and respectful."}, "time_dimension": {"answer": "Present", "rationale": "The entry asks the respondents about their current views and attitudes, and the keywords are \"felt\" and \"are of great importance to\" , which indicate that the issue is one of the beliefs and values that the interviewees are discussing right now.\""}, "sentence_type": "Interrogative sentence", "pos": {"nouns": {"you": 1, "Personal beliefs": 1, "Life": 1, "Importance": 1, "Level": 1}, "verbs": {"I think": 1, "to": 1, "Large": 1}, "adverbs": {"very": 1}, "numerals": {"4": 1}, "pronouns": {"you": 1}}}, {"content": "Does personal belief affect your learning?", "word_count": 15, "label": "", "sentence_structure": "Subject鈥搗erb鈥搊bject", "subjective_objective": {"answer": "Subjective", "rationale": "Because belief is a subjective attitude and conviction of a person, it is not an objective fact or act."}, "sentence_sentiment": {"answer": "Neutral", "rationale": "It is simply an inquiry about whether personal beliefs affect learning, without any apparent emotional or attitudinal tendencies."}, "language_style": {"answer": "Calm and direct", "rationale": "The entry is a direct question to the interviewee, specifically asking if his or her personal beliefs have an impact on learning, and is simple and clear, without too many words or qualifiers."}, "time_dimension": {"answer": "Present", "rationale": "The entry asks the testee's current status and views, the keywords being \"personal belief\", \"improvement of your learning\" and so on, all pointing to the current situation."}, "sentence_type": "Interrogative sentence", "pos": {"nouns": {"Personal beliefs": 1, "you": 1, "Learning": 1}, "verbs": {"Yes": 1, "Impact": 1, "Present": 1}, "adverbs": {"very": 1}, "numerals": {"5": 1}, "classifiers": {"individual": 1}}}]}

{"questionnaire_title": "Quality of Life Questionnaire for EORTCQLQ-C30", "questionnaire_category": "Emotion and affect", "item_count": 30, "emotion_shift_overall": "From negative to positive", "language_style_overall": "Calm and direct", "items": [{"content": "1. Do you feel difficult when you do some hard work, such as lifting heavy shopping bags or suitcases?", "word_count": 30, "label": "", "sentence_structure": "Subject鈥搗erb鈥搊bject", "subjective_objective": {"answer": "Objective", "rationale": "Since the difficulty of doing the work is the result of the patient ' s actual experience, it is objective without taking into account the patient ' s subjective ideas and attitudes."}, "sentence_sentiment": {"answer": "Negative", "rationale": "Because the \"difficult\" act in the entry is negative."}, "language_style": {"answer": "Calm and direct", "rationale": "The entry describes an action (heavy lifting) and possible reactions (difficult), in a clear and straightforward language, without excessive rhetorical or emotional language, and therefore can be judged to be a calm and straightforward language style."}, "time_dimension": {"answer": "Present", "rationale": ""}, "sentence_type": "Interrogative sentence", "pos": {"nouns": {"Actions": 2, "Shopping bags": 1, "Baggage": 1, "Problem": 1}, "verbs": {"Do it": 2, "- It's hard": 1, "T": 1, "I feel": 1, "Difficulties": 1}, "adjectives": {"Heavy": 1, "It's hard work": 1}, "numerals": {"1": 8}, "classifiers": {"individual": 1}, "pronouns": {"you": 3}}}, {"content": "2. Do you feel difficult walking at long distances?", "word_count": 13, "label": "", "sentence_structure": "Subject鈥搗erb鈥搊bject", "subjective_objective": {"answer": "Objective", "rationale": "Because the difficulty is the physical feeling of the patient, it is an objective description."}, "sentence_sentiment": {"answer": "Negative", "rationale": "Because the description \"difficult\" is negative."}, "language_style": {"answer": "Calm and direct", "rationale": "The entry directly asks if the interviewee has any difficulty walking long distances, is simple in language style, is not using any qualifier or emotional expression, and is more direct and objective."}, "time_dimension": {"answer": "Present", "rationale": "The entry now asks if the testee has any difficulty in walking at long distances in the current reality, so the temporal dimension is now judged."}, "sentence_type": "Interrogative sentence", "pos": {"nouns": {"Long distance": 1, "Walk": 1}, "verbs": {"I feel": 1, "Difficulties": 1}}}, {"content": "3. Do you feel difficulties while walking at short distances?", "word_count": 16, "label": "", "sentence_structure": "Subject鈥搗erb鈥搊bject", "subjective_objective": {"answer": "Objective", "rationale": "Because \"Do you feel difficult walking at short distances outside?\" is a question that needs to be answered by the patient, and the answer is the patient's subjective feelings and actual situation, and therefore it is considered from an objective perspective."}, "sentence_sentiment": {"answer": "Neutral", "rationale": "As the entry merely asks whether there are difficulties in taking short walks outside the home, it does not express positive or negative emotional tendencies."}, "language_style": {"answer": "Calm and direct", "rationale": "The entry is in a concise language and asks whether the other party has experienced difficulties in walking at short outdoor distances, is more objective and direct, and is not over-modified or emotional."}, "time_dimension": {"answer": "Present", "rationale": "The keyword \"for a short walk outside\" and \"for a difficult time\" suggests that it is a direct question of current behaviour, so the answer should be now."}, "sentence_type": "Interrogative sentence", "pos": {"nouns": {"Outdoor": 1, "Short Distance": 1, "Walking": 1, "you": 1, "Difficulties": 1}, "verbs": {"I feel": 1}, "adverbs": {"Time": 1}, "numerals": {"3": 1}}}, {"content": "Four. Do you have to lie down or sit on a chair during the day?", "word_count": 16, "label": "", "sentence_structure": "Subject鈥搗erb鈥搊bject", "subjective_objective": {"answer": "Objective", "rationale": "Since the question is whether the patient must be in bed or sitting on a chair during the day, it is an objective fact and is not influenced by the patient ' s subjective consciousness."}, "sentence_sentiment": {"answer": "Negative", "rationale": "Because this entry describes an act that must be laid or sat, it is generally considered to be inactive or unhealthy."}, "language_style": {"answer": "Calm and direct", "rationale": "Basis for the determination: The entry is in a concise language asking the interviewee whether he or she must be bedridden or sitting on a chair, without adding excessive qualifier or emotional colours, and with a more objective and direct tone."}, "time_dimension": {"answer": "Present", "rationale": "The entry now asks the person who was tested for his current state of behaviour, i.e. whether he or she needs to be bedridden or sitting on a chair during the day, and thus judges now."}, "sentence_type": "Interrogative sentence", "pos": {"nouns": {"Daytime": 1, "you": 2, "The bed": 1, "The chair": 1}, "verbs": {"Get in bed": 1, "Sit down": 1}, "numerals": {"4": 1, "I have to": 1, "or": 1, "in": 1}}}, {"content": "5. Do you need help with food, clothing, washing or toilet?", "word_count": 19, "label": "", "sentence_structure": "Subject鈥搗erb鈥搊bject", "subjective_objective": {"answer": "Objective", "rationale": "The need for assistance in feeding, dressing, washing or toilet is an objective condition for the patient and does not require a subjective consideration."}, "sentence_sentiment": {"answer": "Negative", "rationale": "Because the behavior in the entries \"needs help with food, clothing, washing or toilet\" is negative."}, "language_style": {"answer": "Calm and direct", "rationale": ""}, "time_dimension": {"answer": "Present", "rationale": ""}, "sentence_type": "Interrogative sentence", "pos": {"nouns": {"person": 2, "Assistance": 1, "Food": 1, "Get dressed": 1, "Wash": 1, "To the bathroom": 1}, "verbs": {"Yes": 1, "Assistance": 1, "Food": 1, "Get dressed": 1, "Wash": 1, "To the bathroom": 1}, "numerals": {"5": 1}, "classifiers": {"individual": 1}, "pronouns": {"you": 1}}}, {"content": "6. Are your work or daily activities physically restricted?", "word_count": 18, "label": "", "sentence_structure": "Subject鈥搗erb鈥搊bject", "subjective_objective": {"answer": "Objective", "rationale": "Because the problem is an inquiry into the patient ' s physical condition and capacity constraints, these are objective facts and are not influenced by the patient ' s subjective attitude."}, "sentence_sentiment": {"answer": "Neutral", "rationale": "Because the entry is merely a question of whether the interviewee has physical limitations and does not involve any positive or negative emotional aspects."}, "language_style": {"answer": "Calm and direct", "rationale": "The entry is directly asked about physical limitations in the work or daily activities of the person interviewed, the language style is simpler and clear, there are no excessive emotional colours or qualifying statements, and it is consistent with a calm and direct description."}, "time_dimension": {"answer": "Present", "rationale": "The entry asks about the current state of the person being tested and whether there are physical limitations in daily life/work, so the temporal dimension is now judged."}, "sentence_type": "Interrogative sentence", "pos": {"nouns": {"Work": 1, "Daily activities": 1, "Physical": 1, "Limits": 1}, "verbs": {"Yes": 1, "Impact": 1}, "adverbs": {"Is it possible": 1}, "numerals": {"6": 1}}}, {"content": "7. Are your leisure and leisure activities physically restricted?", "word_count": 19, "label": "", "sentence_structure": "Subject鈥搗erb鈥搊bject", "subjective_objective": {"answer": "Subjective", "rationale": "As amateur and leisure activities are subjective choices and arrangements for patients, physical constraints are a subjective feeling."}, "sentence_sentiment": {"answer": "Neutral", "rationale": "As the entry does not express positive or negative feelings or attitudes, it merely asks the actual situation of the interviewees."}, "language_style": {"answer": "Calm and direct", "rationale": "The language style of the entry is relatively calm and straightforward, and there is no excessive rhetorical or emotional colour, but rather an objective and concise inquiry into the extent to which the health of the person under investigation affects their amateur and leisure activities."}, "time_dimension": {"answer": "Present", "rationale": "The entry asks the testee about the current situation, and the time dimension is now judged. The keyword \"are not affected\" indicates that this is an assessment of the current situation."}, "sentence_type": "Interrogative sentence", "pos": {"nouns": {"Amateurity": 1, "Leisure activities": 1, "Physical limit": 1}, "verbs": {"Received": 1, "Limits": 1}, "numerals": {"7": 1}}}, {"content": "Have you ever felt short?", "word_count": 7, "label": "", "sentence_structure": "Subject鈥損redicate", "subjective_objective": {"answer": "Objective", "rationale": "Because a person ' s sense of low air is a direct sense of the patient, it is a subjective aspect of the examination."}, "sentence_sentiment": {"answer": "Negative", "rationale": "Because \"fright short\" is an uncomfortable experience, the entry is negative emotional."}, "language_style": {"answer": "Calm and direct", "rationale": "The entry is more calm and straightforward, and it opens the door to the question whether the interviewee has had a short experience, has not been overdoing the adornment or emotional rendering. This expression is more objective, clear, and it allows the interviewee to understand the meaning of the question and give an accurate answer."}, "time_dimension": {"answer": "Past", "rationale": "The entry asks if the person tested has ever had one of the symptoms (i.e. \"smear\"), and therefore the temporal dimension is judged to be past."}, "sentence_type": "Interrogative sentence", "pos": {"nouns": {"you": 1, "Short air": 1}, "verbs": {"Tsang": 1, "I feel": 1, "Short air": 1}}}, {"content": "Have you ever had any pain?", "word_count": 6, "label": "", "sentence_structure": "Subject鈥損redicate", "subjective_objective": {"answer": "Objective", "rationale": "Since the existence of pain is an objective experience and symptom of the patient, it is not influenced by the patient ' s subjective attitude."}, "sentence_sentiment": {"answer": "Negative", "rationale": "Because the experience of having pain is negative."}, "language_style": {"answer": "Calm and direct", "rationale": "The entry, which is based on a straightforward language style, briefly asked whether the person surveyed had experienced physical pain, had not been over-represented in rhetoric or emotional colours, and was more objective and neutral."}, "time_dimension": {"answer": "Present", "rationale": "The entry is now asking if the person tested feels pain at the moment, and judging the time dimension is now."}, "sentence_type": "Interrogative sentence", "pos": {"nouns": {"Pain": 1}, "verbs": {"have": 1}, "numerals": {"How many": 0, "individual": 0}}}, {"content": "Do you ever need a break?", "word_count": 7, "label": "", "sentence_structure": "Subject鈥損redicate", "subjective_objective": {"answer": "Subjective", "rationale": "The aim is to look at the patient from the subjective point of view, because \"Have you ever needed rest?\" It's about the patient's own feelings and experiences."}, "sentence_sentiment": {"answer": "Neutral", "rationale": "Because the entry merely asked the interviewee whether he had ever needed rest and had no emotional colour."}, "language_style": {"answer": "Tactful and warm", "rationale": "The entry uses the tone of inquiry and does not make it clear whether answers are required or mandatory to provide answers, which is more moderate and friendly, easier to comfort and relax the interviewee and thus easier to obtain real feedback."}, "time_dimension": {"answer": "Present", "rationale": ""}, "sentence_type": "Interrogative sentence", "pos": {"nouns": {"Questionnaire": 1, "Entry": 1, "you": 1, "Rest": 1}, "verbs": {"Yes": 1}, "adverbs": {"Tsang": 1}, "numerals": {"10": 1}}}, {"content": "Did you ever feel bad about sleeping?", "word_count": 9, "label": "", "sentence_structure": "Subject鈥損redicate", "subjective_objective": {"answer": "Subjective", "rationale": "Because poor sleep is the patient's subjective feeling and experience."}, "sentence_sentiment": {"answer": "Negative", "rationale": "Because the fact that \"sleep is bad\" in the entry is negative."}, "language_style": {"answer": "Tactful and warm", "rationale": ""}, "time_dimension": {"answer": "Past", "rationale": ""}, "sentence_type": "Interrogative sentence", "pos": {"nouns": {"Sleep": 1, "Not good": 1}, "verbs": {"I felt": 1}, "numerals": {"11": 1}}}, {"content": "Twelve. Have you ever felt weak?", "word_count": 7, "label": "", "sentence_structure": "Subject鈥損redicate", "subjective_objective": {"answer": "Subjective", "rationale": "Because feeling weak is a subjective feeling."}, "sentence_sentiment": {"answer": "Negative", "rationale": "Because \"weak\" in the entry is a negative emotional vocabulary."}, "language_style": {"answer": "Tactful and warm", "rationale": "The language style of the entry is more euphemistic, using a polite phrase such as \"You felt\" and not asking the other side whether it had ever been physically ill or sensitive topics like disease, but directing the interviewee to answer in a more euphemism and moderation. Moreover, the term \"weak\" is not too emotional, so the language style of the article is generally more euphemistic."}, "time_dimension": {"answer": "Present", "rationale": "It can be inferred from the time term \"had\" in the sentence that the question was whether the person tested had previously felt weak, but did not specify a specific time frame or duration, and the time dimension is therefore judged to be now."}, "sentence_type": "Interrogative sentence", "pos": {"nouns": {"you": 1, "Weakness": 1}, "verbs": {"I felt": 1}, "adverbs": {"- You're not": 1}, "numerals": {"12": 1}}}, {"content": "Do you ever feel like you don't have an appetite?", "word_count": 9, "label": "", "sentence_structure": "Subject鈥搗erb鈥搊bject", "subjective_objective": {"answer": "Subjective", "rationale": "Because feeling without appetite is a subjective feeling for the patient."}, "sentence_sentiment": {"answer": "Negative", "rationale": "Because the \"no appetite\" situation in the entry is negative."}, "language_style": {"answer": "Calm and direct", "rationale": "The language style of the entry is relatively simple, there is no excessive accoutrements or emotional colour, and the interviewee was asked whether he or she had ever felt unattractive and was speaking with a more objective and calm tone."}, "time_dimension": {"answer": "Past", "rationale": "The keyword \"did feel\" indicates that the entry was questioned by the testee's past experience or feelings, and therefore judged to be past."}, "sentence_type": "Interrogative sentence", "pos": {"nouns": {"Questionnaire": 1, "Entry": 1, "you": 1, "-Adulge": 1}, "verbs": {"I feel": 1, "have": 1}, "adverbs": {"Tsang": 1}, "numerals": {"13": 1}, "classifiers": {"individual": 1}}}, {"content": "14: Have you ever felt sick and sick?", "word_count": 10, "label": "", "sentence_structure": "Subject鈥搗erb鈥搊bject", "subjective_objective": {"answer": "Subjective", "rationale": "Because nausea is a subjective physical feeling, different people feel different about it and the extent."}, "sentence_sentiment": {"answer": "Negative", "rationale": "The basis for the determination: The \"disgusting to vomit\" in the entry is a symptom of physical discomfort and therefore has a negative emotional color."}, "language_style": {"answer": "Calm and direct", "rationale": "The entry raises questions in the form of simple and clear questions, without too much qualifier or emotional colour, with a more balanced and objective tone, and is typical of a calm and direct language style."}, "time_dimension": {"answer": "Present", "rationale": "The entry is to ask if the testee ever felt sick and sick, and the time dimension is the past. So the answer is \"the past.\""}, "sentence_type": "Interrogative sentence", "pos": {"nouns": {"Gross": 1, "I'm gonna throw up": 1}, "verbs": {"Feel": 1, "Tsang": 1}, "adverbs": {"Tsang": 1}, "numerals": {"14": 1}}}, {"content": "Have you ever vomited?", "word_count": 6, "label": "", "sentence_structure": "Subject鈥搗erb鈥搊bject", "subjective_objective": {"answer": "Objective", "rationale": "As vomiting is an observed behaviour or symptom, it is objective."}, "sentence_sentiment": {"answer": "Negative", "rationale": "As vomiting is usually a physical discomfort, it can cause discomfort or concern."}, "language_style": {"answer": "Calm and direct", "rationale": "The language style of the entry is relatively simple, there is no excessive qualifier or emotional colour, and it is designed only to obtain objective information and therefore belongs to the \"cool and direct\" language style."}, "time_dimension": {"answer": "Past", "rationale": "The entry asks if the person tested has ever experienced vomiting and the temporal dimension is judged to be past."}, "sentence_type": "Interrogative sentence", "pos": {"nouns": {"you": 1, "- Vomit": 1, "Mass": 1}, "verbs": {"- Vomit": 1, "Tsang": 1, "- I've been sick": 1}, "adverbs": {"Tsang": 1, "- You're not": 1}, "numerals": {"15": 1}, "pronouns": {"you": 1}}}, {"content": "Have you had constipation?", "word_count": 6, "label": "", "sentence_structure": "Subject鈥損redicate", "subjective_objective": {"answer": "Objective", "rationale": "Because constipation is a specific biological phenomenon, it falls within the realm of objective facts."}, "sentence_sentiment": {"answer": "Negative", "rationale": "Because constipation is a physical discomfort, which is usually considered negative."}, "language_style": {"answer": "Calm and direct", "rationale": "The entry asks the interviewees in a direct and clear manner whether they have had a constipated experience, a more calm and objective language style, and not using excessive rhetorical or emotional colours."}, "time_dimension": {"answer": "Past", "rationale": "The keyword \"had been\" indicates that the person to be tested had to answer whether there had been a situation of constipation at a certain point in time in the past. The temporal dimension of the article is therefore judged to be the past."}, "sentence_type": "Interrogative sentence", "pos": {"nouns": {"Decency": 1}, "verbs": {"There was": 1, "- You're not": 1}}}, {"content": "You ever had diarrhoea?", "word_count": 6, "label": "", "sentence_structure": "Subject鈥搗erb鈥搊bject", "subjective_objective": {"answer": "Subjective", "rationale": "\"You have had diarrhoea?\" is viewed from the patient's subjective point of view. Because whether a patient has had diarrhoea is a personal experience and a subjective situation."}, "sentence_sentiment": {"answer": "Neutral", "rationale": "Because the entry does not have any emotional color."}, "language_style": {"answer": "Calm and direct", "rationale": "The entry is more calm and objective in its language, and does not have too much emotional rendering or rhetorical techniques. It is simple to ask if the other person has ever had symptoms of diarrhoea and is more direct."}, "time_dimension": {"answer": "Past", "rationale": "The entry is for questioning the testee ' s past behaviour and the temporal dimension is a past."}, "sentence_type": "Interrogative sentence", "pos": {"nouns": {"Diarrhoea": 1}, "verbs": {"You did": 1}, "numerals": {"17": 1}}}, {"content": "Have you ever been tired?", "word_count": 7, "label": "", "sentence_structure": "Subject鈥損redicate", "subjective_objective": {"answer": "Subjective", "rationale": "Because fatigue is a human feeling, a subjective experience."}, "sentence_sentiment": {"answer": "Neutral", "rationale": "The entry was simply an inquiry into whether the person under investigation felt fatigue and did not have any emotional colours."}, "language_style": {"answer": "Tactful and warm", "rationale": "The entry asks the other side whether it has experienced fatigue in a concerned and understanding manner, rather than directly accusing or questioning, which is more humane and friendly. At the same time, the word \"had\" is used to give the person under investigation the space to think about his or her own experience, without being too offensive or uncomfortable."}, "time_dimension": {"answer": "Past", "rationale": "The entry asks the testee whether he has had some experience or experience, and the time dimension is judged to be past."}, "sentence_type": "Interrogative sentence", "pos": {"nouns": {"Weary": 1, "you": 1}, "verbs": {"I used to feel": 1}, "adverbs": {"Tsang": 1}}}, {"content": "19: Does pain interfere with your daily activities?", "word_count": 11, "label": "", "sentence_structure": "Subject鈥搗erb鈥搊bject", "subjective_objective": {"answer": "Objective", "rationale": "Because \"the pain hinders your daily activities\" is an objective phenomenon that can be felt or observed by the patient."}, "sentence_sentiment": {"answer": "Negative", "rationale": "Because \"the pain is obstructing\" is negative."}, "language_style": {"answer": "Tactful and warm", "rationale": "The entry is more adept and interactive when it is spoken in a gentle and inviting manner, rather than by direct statements or orders."}, "time_dimension": {"answer": "Present", "rationale": "The entry is for the current state of the subject, \"Was the pain obstructing your daily activities?\" The \"obstruction\" and \"daily activities\" in the \"Waste\" point to the behaviour or state currently under way, so the temporal dimension is now judged."}, "sentence_type": "Interrogative sentence", "pos": {"nouns": {"Pain": 1, "Obstruction": 1, "Daily activities": 1}, "verbs": {"Yours": 1, "Obstruction": 1, "Daily activities": 1}, "adverbs": {"- You're not": 1}, "pronouns": {"you": 2}}}, {"content": "20: Is it hard to concentrate on things like reading newspapers or watching TV?", "word_count": 20, "label": "", "sentence_structure": "Subject鈥搗erb鈥搊bject", "subjective_objective": {"answer": "Subjective", "rationale": "Because the issue involves the feelings and experiences of individuals, it is a subjective examination of the patient."}, "sentence_sentiment": {"answer": "Negative", "rationale": "Because the expression \"difficulties to focus on\" in the entry is negative, it indicates that difficulties and challenges in doing things can lead to depression and anxiety."}, "language_style": {"answer": "Calm and direct", "rationale": "The entry directly asked whether the respondents had difficulty in focusing on their daily lives, without too many rhetorical or emotional renderings, and in a more concise and straightforward language style."}, "time_dimension": {"answer": "Present", "rationale": "So the output should be \"now\"."}, "sentence_type": "Interrogative sentence", "pos": {"nouns": {"Attention": 1, "Yes": 1, "Report": 1, "Television": 1}, "verbs": {"Hard": 1, "Do it": 1, "Read it": 1, "Look": 1}, "adjectives": {"It's hard": 1}, "numerals": {"20": 1}}}, {"content": "Have you ever been nervous?", "word_count": 7, "label": "", "sentence_structure": "Subject鈥損redicate", "subjective_objective": {"answer": "Subjective", "rationale": "The test is based on the fact that the stress is a subjective experience or feeling, and the entry is therefore viewed from the patient ' s subjective perspective."}, "sentence_sentiment": {"answer": "Negative", "rationale": "Because \"stretch\" in the entry is a negative emotional experience."}, "language_style": {"answer": "Calm and direct", "rationale": "The language style of the entry is relatively calm and straightforward, and the subject of the question is not expressed in too many rhetorical or emotional terms, but rather in a concise and clear manner, which makes it easier for the interviewees to understand and answer."}, "time_dimension": {"answer": "Present", "rationale": "The entry asks if the testee feels nervous at the moment, and therefore the time dimension is now judged."}, "sentence_type": "Interrogative sentence", "pos": {"nouns": {"you": 1, "Nervous": 1}, "verbs": {"I felt": 1, "- You're not": 1}, "adverbs": {"Tsang": 1}, "numerals": {"21": 1}}}, {"content": "22: Have you ever been worried?", "word_count": 7, "label": "", "sentence_structure": "Subject鈥損redicate", "subjective_objective": {"answer": "Subjective", "rationale": "Because fear is subjective."}, "sentence_sentiment": {"answer": "Neutral", "rationale": "Since the entry was not of any emotional colour, it was merely an inquiry into whether the person under investigation had been concerned."}, "language_style": {"answer": "Calm and direct", "rationale": "The language style of the entry is more calm and objective, and the person surveyed is asked whether he or she has had a worrying emotional experience, has not had too many rhetorical or emotional expressions. Such expressions are easier to obtain a true answer from the person surveyed and can avoid the influence of the leading questions on the outcome."}, "time_dimension": {"answer": "Present", "rationale": "The entry asks if the person who was tested was ever worried in reply to the questionnaire, and therefore the temporal dimension is now judged."}, "sentence_type": "Interrogative sentence", "pos": {"nouns": {"you": 1, "I'm worried": 1}, "verbs": {"I felt": 1}, "adverbs": {"- You're not": 1}, "numerals": {"22": 1}}}, {"content": "Have you ever felt so angry?", "word_count": 9, "label": "", "sentence_structure": "Subject鈥搗erb鈥搊bject", "subjective_objective": {"answer": "Subjective", "rationale": "Because it's a subjective emotional experience and feeling of a person who feels easily angry."}, "sentence_sentiment": {"answer": "Negative", "rationale": "Because the term \"easy to get angry\" is negative."}, "language_style": {"answer": "Calm and direct", "rationale": "The article is based on simple language, direct inquiries into the interviewee鈥檚 anger, no qualifier or emotional colour, and a more objective tone. The language style of the entry is therefore straightforward and calm."}, "time_dimension": {"answer": "Present", "rationale": "Now the entry asks the testees about their current feelings and experiences, whether they ever felt easily angry. The keyword \"had felt\" indicates that this was a question of past experience, but it does not specify how long ago it was. So the time dimension of the entry is now."}, "sentence_type": "Interrogative sentence", "pos": {"nouns": {"you": 1, "Anger": 1, "Questionnaire": 1, "Entry": 1}, "verbs": {"I felt": 1, "Easy": 1, "Move": 1}, "adverbs": {"Tsang": 1, "- You're not": 1}, "numerals": {"23": 1}, "pronouns": {"you": 1}}}, {"content": "24. Have you ever felt depressed?", "word_count": 9, "label": "", "sentence_structure": "Subject鈥損redicate", "subjective_objective": {"answer": "Subjective", "rationale": "Because depression is the patient ' s own feelings and experiences, it is a subjective examination."}, "sentence_sentiment": {"answer": "Negative", "rationale": "Because \"frightful\" is a negative emotional state, the emotional tendency to use it is negative."}, "language_style": {"answer": "Tactful and warm", "rationale": "The entry uses a soft question-and-answer approach, which leads the interviewee to think at the beginning of \"You felt\" without directly blaming or imposing emotions, and which is more personal and interactive."}, "time_dimension": {"answer": "Past", "rationale": "The entry asks if the person tested had experienced any emotional depression and the temporal dimension was judged to be past."}, "sentence_type": "Interrogative sentence", "pos": {"nouns": {"Emotion": 1, "Down": 1}, "verbs": {"Sensor": 1, "Present": 1}, "adverbs": {"Tsang": 1}, "numerals": {"24": 1}}}, {"content": "Have you ever felt difficulty remembering?", "word_count": 10, "label": "", "sentence_structure": "Subject鈥搗erb鈥搊bject", "subjective_objective": {"answer": "Subjective", "rationale": "Because it is a subjective experience and feeling that you feel that you have a hard memory."}, "sentence_sentiment": {"answer": "Negative", "rationale": "Because \"rememberance difficulties\" are usually negative, indicating problems such as memory impairments or memory impairments."}, "language_style": {"answer": "Calm and direct", "rationale": "The entry is a direct question-and-answer dialogue, without too many qualifiers or emotional colours, and with a more calm and objective language style."}, "time_dimension": {"answer": "Past", "rationale": ""}, "sentence_type": "Interrogative sentence", "pos": {"nouns": {"you": 1, "Note": 1, "Difficulties": 1}, "verbs": {"I felt": 1}, "adverbs": {"Once": 1}, "numerals": {"25": 1}}}, {"content": "26. Does your physical condition or the process of treatment hinder your family life?", "word_count": 21, "label": "", "sentence_structure": "Subject鈥搗erb鈥搊bject", "subjective_objective": {"answer": "Subjective", "rationale": "This is because patients subjectively believe that their physical condition or the treatment process has affected their family life."}, "sentence_sentiment": {"answer": "Negative", "rationale": "Because the description \"construct your family\" in the entry is negative."}, "language_style": {"answer": "Calm and direct", "rationale": "The entry is based on a straightforward inquiry into the physical condition of the other party or whether the treatment process has affected his family life, is more balanced and objective, and does not employ excessive rhetorical or emotional rendering."}, "time_dimension": {"answer": "Present", "rationale": "The entry now asks whether the current state of health or the treatment process has impeded family life, and the temporal dimension is now judged."}, "sentence_type": "Interrogative sentence", "pos": {"nouns": {"Physical condition": 1, "The process of treatment": 1, "Family life": 1}, "verbs": {"Obstruction": 1, "have": 1}, "numerals": {"26": 1}, "pronouns": {"you": 1}}}, {"content": "27. Are your physical condition or treatments a hindrance to your social activities?", "word_count": 21, "label": "", "sentence_structure": "Subject鈥搗erb鈥搊bject", "subjective_objective": {"answer": "Subjective", "rationale": "Because the problem is primarily concerned with patients ' subjective feelings and experiences, i.e. whether their physical condition affects their social activities."}, "sentence_sentiment": {"answer": "Negative", "rationale": "Because the words \"therapeutic process\" and \"disrupting social activities\" in the entries have negative emotional connotations."}, "language_style": {"answer": "Calm and direct", "rationale": "The purpose of this questionnaire is straightforward, without any emotional or acquital tone, directly to the interviewee, in a clear and concise language, and without too many rhetorical or emotional accents. The language style of the article is therefore more incriminating."}, "time_dimension": {"answer": "Present", "rationale": "The entry asks whether the person tested is currently physically fit or is being treated in a way that hinders their social activities, and the temporal dimension is now. Key words include \"physical condition\", \"the treatment process\" and \"the impediment to your social activities\", all pointing to the current situation or state."}, "sentence_type": "Interrogative sentence", "pos": {"nouns": {"Physical condition": 1, "The process of treatment": 1, "Social events": 1}, "verbs": {"Obstruction": 1}}}, {"content": "28. Are you in financial distress because of your physical condition or the process of treatment?", "word_count": 21, "label": "", "sentence_structure": "Subject鈥損redicate", "subjective_objective": {"answer": "Objective", "rationale": "Because it is the objective fact of the patient ' s experience, not just subjective feelings or attitudes, whether or not economic hardship is caused."}, "sentence_sentiment": {"answer": "Negative", "rationale": "Because the result of the entry \"caused your economic difficulties\" is negative."}, "language_style": {"answer": "Calm and direct", "rationale": "The entry is relatively calm and objective in its language, and does not have too many emotional or rhetorical techniques."}, "time_dimension": {"answer": "Past", "rationale": "The entry asks whether the testee has caused economic hardship due to his or her state of health or the treatment process, and the temporal dimension is a matter of the past."}, "sentence_type": "Interrogative sentence", "pos": {"nouns": {"Physical condition": 1, "The process of treatment": 1, "Economic difficulties": 1}, "verbs": {"Cause": 1}, "numerals": {"28": 1}}}, {"content": "29. How do you assess your overall health over the past week?", "word_count": 18, "label": "", "sentence_structure": "Subject鈥搗erb鈥搊bject", "subjective_objective": {"answer": "Subjective", "rationale": "The examination of this question is a self-evaluation and perception of the patient ' s overall health status over the past week, which is a subjective view of the patient."}, "sentence_sentiment": {"answer": "Positive", "rationale": "Because the \"overall health\" in the entry is a positive evaluation criterion, it is an expression of self-assessment of the health of the person surveyed."}, "language_style": {"answer": "Tactful and warm", "rationale": "The language style of the entry is more moderate, using more objective and neutral terms such as \"assessment\" and \"overall health\", and is not subject to too much subjective judgement or emotional colours and can therefore be considered as a soft language style."}, "time_dimension": {"answer": "Past", "rationale": "The entry is an inquiry into the testee ' s self-assessment of the overall health condition over the past week, which is judged by the time dimension as the past."}, "sentence_type": "Interrogative sentence", "pos": {"nouns": {"Health status": 1, "A week": 1}, "verbs": {"Assessment": 1}, "adverbs": {"Past": 1, "Overall": 1}, "numerals": {"29": 1}}}, {"content": "30 How do you assess your overall quality of life over the past week?", "word_count": 18, "label": "", "sentence_structure": "Subject鈥搗erb鈥搊bject", "subjective_objective": {"answer": "Subjective", "rationale": "Because it is a question of assessing the patient ' s subjective view of his or her living conditions, it is a subjective one."}, "sentence_sentiment": {"answer": "Neutral", "rationale": "Because the entry was not emotional, it was merely an inquiry into the quality of life of the last week."}, "language_style": {"answer": "Tactful and warm", "rationale": "The entry is more friendly and acceptable in euphemistic terms, which allow respondents to self-assess the quality of life of the past week rather than directly giving an assessment or an order."}, "time_dimension": {"answer": "Past", "rationale": ""}, "sentence_type": "Interrogative sentence", "pos": {"nouns": {"A week": 1, "Quality of life": 1}, "verbs": {"Assessment": 1}, "adverbs": {"Past": 1, "How's that": 1}, "numerals": {"30": 1}}}]}

{"questionnaire_title": "Quality of Life Questionnaire for EORTCQLQ-LC13", "questionnaire_category": "Emotion and affect", "item_count": 56, "emotion_shift_overall": "From negative to positive", "language_style_overall": "Calm and direct", "items": [{"content": "31. Do you cough often?", "word_count": 6, "label": "", "sentence_structure": "Subject鈥損redicate", "subjective_objective": {"answer": "Objective", "rationale": "Coughing is a physical reaction, an objective fact that is not entirely controlled by the patient鈥檚 subjective will. The answer to this question should therefore be considered objective."}, "sentence_sentiment": {"answer": "Negative", "rationale": "Because cough is a symptom of physical discomfort, it is usually considered a negative situation."}, "language_style": {"answer": "Calm and direct", "rationale": "The entry is in direct, concise language asking the respondents whether they often cough, without excessive adoration or emotional rendering, and in a language style that is more calm and objective."}, "time_dimension": {"answer": "Past", "rationale": ""}, "sentence_type": "Interrogative sentence", "pos": {"nouns": {"Cough": 1}, "verbs": {"you": 1, "Regular": 1, "Cough": 1, "- You're not": 1}, "adverbs": {"Regular": 1}, "numerals": {"31": 1}, "pronouns": {"you": 1}}}, {"content": "You cough blood?", "word_count": 8, "label": "", "sentence_structure": "Subject鈥搗erb鈥搊bject", "subjective_objective": {"answer": "Objective", "rationale": "Because coughing blood is a symptom or a sign of the patient, it is an objective fact."}, "sentence_sentiment": {"answer": "Negative", "rationale": "Because coughing blood is a pathological expression, with some negative emotional color."}, "language_style": {"answer": "Calm and direct", "rationale": "The entry is directly and succinctly asked whether the person surveyed had coughed or bled, without using excessive qualifiers or emotional colours and with a more calm and straightforward language style."}, "time_dimension": {"answer": "Present", "rationale": "The entry asks if the person tested is currently coughing blood, and the temporal dimension is now. The keyword \"Do you cough blood\" and \"Little blood\" point to the current symptoms."}, "sentence_type": "Interrogative sentence", "pos": {"nouns": {"Blood": 1, "Ahem": 1, "Boom": 1}, "verbs": {"- Yeah": 1}, "adverbs": {"- You're not": 1}, "numerals": {"32": 1}}}, {"content": "Did you feel short on your break?", "word_count": 9, "label": "", "sentence_structure": "Subject鈥損redicate", "subjective_objective": {"answer": "Subjective", "rationale": "Because \"fright short\" is the patient's subjective feeling, self-evaluation and judgement of his or her own state."}, "sentence_sentiment": {"answer": "Negative", "rationale": "Because the emotional description of \"fright short\" in the entry is negative."}, "language_style": {"answer": "Calm and direct", "rationale": "This expression is clear and clear and helps to collect accurate data."}, "time_dimension": {"answer": "Present", "rationale": ""}, "sentence_type": "Interrogative sentence", "pos": {"nouns": {"Rest": 1, "Short air": 1}, "verbs": {"I feel": 1}, "numerals": {"33": 1}}}, {"content": "34. Did you feel short on your walk?", "word_count": 9, "label": "", "sentence_structure": "Subject鈥搗erb鈥搊bject", "subjective_objective": {"answer": "Subjective", "rationale": "Because feeling short is a subjective feeling."}, "sentence_sentiment": {"answer": "Negative", "rationale": "Because the description \"fright short\" is negative."}, "language_style": {"answer": "Calm and direct", "rationale": ""}, "time_dimension": {"answer": "Present", "rationale": "\"Showing short-tempered\" is the current state of affairs, which suggests that the person tested would have done so at the time of the act, and thus the judgement is now."}, "sentence_type": "Interrogative sentence", "pos": {"nouns": {"Questionnaire": 1, "Entry": 1, "you": 1, "Walking": 1, "Short air": 1}, "verbs": {"I feel": 1, "Question": 1}, "adverbs": {"Time": 1}, "numerals": {"34": 1}}}, {"content": "Do you feel short on the stairs?", "word_count": 10, "label": "", "sentence_structure": "Subject鈥搗erb鈥搊bject", "subjective_objective": {"answer": "Objective", "rationale": "Because \"fright short\" is a physical reaction, actually felt by the patient during the climbing of the stairs, and is an objective fact."}, "sentence_sentiment": {"answer": "Negative", "rationale": "Because the \"fright short\" sign in the entry is negative."}, "language_style": {"answer": "Calm and direct", "rationale": "The entry is in a direct, simple language and asks the interviewees whether they feel short on climbing the stairs, do not use excessive rhetorical techniques or emotional rendering, and is more balanced and objective and suitable for psychological questionnaire surveys."}, "time_dimension": {"answer": "Present", "rationale": "The entry is for the testee ' s current behaviour or state, and the time dimension is now judged."}, "sentence_type": "Interrogative sentence", "pos": {"nouns": {"Stairs": 1, "Short air": 1}, "verbs": {"Climb": 1, "I feel": 1}, "numerals": {"35": 1}}}, {"content": "36: Have you had any oral or tongue pain?", "word_count": 11, "label": "", "sentence_structure": "Subject鈥搗erb鈥搊bject", "subjective_objective": {"answer": "Objective", "rationale": "Because oral or tongue pain is an objective fact of the patient ' s experience."}, "sentence_sentiment": {"answer": "Negative", "rationale": "Because the \"sniffing of the mouth or tongue\" in the entry is negative."}, "language_style": {"answer": "Calm and direct", "rationale": ""}, "time_dimension": {"answer": "Past", "rationale": "The entry is for questioning the testee ' s past behaviour and the temporal dimension is a past."}, "sentence_type": "Interrogative sentence", "pos": {"nouns": {"36": 1, "Oral": 1, "tongue": 1, "Pain": 1}, "verbs": {"Yes": 1, "- You're not": 1}, "numerals": {"36": 1}}}, {"content": "You ever have trouble swallowing?", "word_count": 8, "label": "", "sentence_structure": "Subject鈥損redicate", "subjective_objective": {"answer": "Objective", "rationale": "Since ingestion difficulties are a physical condition, they are an objective fact that the patient has experienced."}, "sentence_sentiment": {"answer": "Negative", "rationale": "Because the \"swallowing difficulty\" in the entry is negative."}, "language_style": {"answer": "Calm and direct", "rationale": "The entry is simple and simple in asking the interviewees whether they have had a difficult experience of swallowing, and the language style is more objective and calm, without any rhetoric or emotional rendering."}, "time_dimension": {"answer": "Past", "rationale": ""}, "sentence_type": "Interrogative sentence", "pos": {"nouns": {"It's hard to swallow": 1}, "verbs": {"have": 1}, "numerals": {"37": 1}}}, {"content": "You ever get numbed or stingy?", "word_count": 10, "label": "", "sentence_structure": "Subject鈥損redicate", "subjective_objective": {"answer": "Subjective", "rationale": "Because arm and foot aching/stingling is a subjective feeling and experience of the patient, not an objective fact."}, "sentence_sentiment": {"answer": "Negative", "rationale": "Because the entry describes a symptom of physical discomfort, it is usually considered to be a negative emotional or state."}, "language_style": {"answer": "Calm and direct", "rationale": "This is more conducive to obtaining accurate data and information."}, "time_dimension": {"answer": "Present", "rationale": "The entry asks if the person tested has experienced a sterilisation or sting, and the temporal dimension is judged to be the past. Key words include \"had had\" and \"sting/sting.\" They all point to past experiences."}, "sentence_type": "Interrogative sentence", "pos": {"nouns": {"Hands and feet": 2, "I'm numb": 1, "Stings": 1}, "verbs": {"have": 1}, "numerals": {"38": 1}}}, {"content": "Have you ever had a hair loss?", "word_count": 6, "label": "", "sentence_structure": "Subject鈥損redicate", "subjective_objective": {"answer": "Objective", "rationale": "Because a hair loss is a physical condition and an objective fact."}, "sentence_sentiment": {"answer": "Neutral", "rationale": "Because the entry is a factual issue, it does not express any positive or negative emotional colours."}, "language_style": {"answer": "Tactful and warm", "rationale": "The language style of the entry is more moderate, and the interviewer is guided to answer the questions, without directly implying or highlighting the potential status of the interviewee, which makes it easier for the interviewee to relax and to share his/her true situation."}, "time_dimension": {"answer": "Past", "rationale": "The entry asked if the person tested had experienced a loss of hair and therefore the temporal dimension was judged to be past."}, "sentence_type": "Interrogative sentence", "pos": {"nouns": {"Take your hair off": 1}, "verbs": {"Pass": 1, "have": 1}, "adverbs": {"How many": 1}, "numerals": {"39": 1}, "classifiers": {"individual": 1}}}, {"content": "Have you had chest pains?", "word_count": 6, "label": "", "sentence_structure": "Subject鈥損redicate", "subjective_objective": {"answer": "Objective", "rationale": "Because chest pain is a perceived physical condition, it is not the patient ' s subjective feeling or attitude, but the objective physical condition."}, "sentence_sentiment": {"answer": "Neutral", "rationale": "The expression of purpose is not clearly emotional and is therefore neutral."}, "language_style": {"answer": "Calm and direct", "rationale": "The entry uses a direct, clear language style, which is simple enough to ask the interviewee whether he has experienced chest pain, which is more objective in tone, and does not have too many emotional renderings or rhetorical techniques. This language style applies to the questionnaire survey in a situation where rapid information is required."}, "time_dimension": {"answer": "Past", "rationale": "The entry asked if the person tested had experienced chest pain and the time dimension was judged to be past."}, "sentence_type": "Interrogative sentence", "pos": {"nouns": {"- Chestaches": 1}, "verbs": {"have": 1}, "numerals": {"40": 1, "Pass": 1}}}, {"content": "41. Have you had any pain in your arm or shoulder?", "word_count": 11, "label": "", "sentence_structure": "Subject鈥搗erb鈥搊bject", "subjective_objective": {"answer": "Objective", "rationale": "Because pain is an objective symptom, it is not related to the patient ' s subjective idea."}, "sentence_sentiment": {"answer": "Negative", "rationale": "Because the \"arms or shoulder pain\" in the entry is negative."}, "language_style": {"answer": "Calm and direct", "rationale": "The entry is based on a direct question about whether the other person has suffered pain in his arm or shoulder, without using excessive rhetoric or emotional rendering, and is more objective in tone. At the same time, the question is clearly purposeful, and it is more straightforward to ask directly what the symptoms are."}, "time_dimension": {"answer": "Past", "rationale": "The entry asks if the person tested has experienced pain in the arm or shoulder and the dimension of time is judged to be past."}, "sentence_type": "Interrogative sentence", "pos": {"nouns": {"Arms": 1, "Shoulders": 1, "Pain": 1}, "verbs": {"have": 1}, "numerals": {"41": 1}}}, {"content": "Have you ever had pain in any other part of your body?", "word_count": 13, "label": "", "sentence_structure": "Subject鈥搗erb鈥搊bject", "subjective_objective": {"answer": "Objective", "rationale": "Because the question is whether the patient has experienced a certain symptoms, which are objective."}, "sentence_sentiment": {"answer": "Neutral", "rationale": "The entry was not emotional, but merely asked if the person under investigation had experienced pain in other parts of the body."}, "language_style": {"answer": "Tactful and warm", "rationale": ""}, "time_dimension": {"answer": "Present", "rationale": "In the past, the entry asked the testee whether he had experienced pain in other parts of the body and the temporal dimension was judged to be past."}, "sentence_type": "Interrogative sentence", "pos": {"nouns": {"Body": 1, "Part": 1, "Pain": 1}, "verbs": {"have": 1}, "numerals": {"42": 1}}}, {"content": "Have you ever taken painkillers? If you've done, it's a lot of painkillers?", "word_count": 18, "label": "", "sentence_structure": "Subject鈥搗erb鈥搊bject", "subjective_objective": {"answer": "Objective", "rationale": "Because this is an objective fact of the behaviour and experience of the patient."}, "sentence_sentiment": {"answer": "Positive", "rationale": "Because the entries \"Take painkillers\" and \"Early Painkillers\" are positive behaviors or outcomes."}, "language_style": {"answer": "Calm and direct", "rationale": "The entry is more straightforward in language style, with no excessive aesthetic and emotional colour. It simply asks the interviewee whether he has taken painkillers and asks them how it works. This tone is neutral and objective."}, "time_dimension": {"answer": "Past", "rationale": "The entry asks if the person tested had ever been on painkillers and how well the painkillers worked, and the temporal dimension is a past."}, "sentence_type": "Interrogative sentence", "pos": {"nouns": {"you": 1, "Take it": 1, "Painkillers": 1, "Painkilling": 1}, "verbs": {"Yes": 1, "It's a pain": 1}, "adjectives": {"Large": 1}, "numerals": {"43": 1}}}, {"content": "1. Do you feel difficult when you do some hard work, such as lifting heavy shopping bags or suitcases?", "word_count": 30, "label": "", "sentence_structure": "Subject鈥搗erb鈥搊bject", "subjective_objective": {"answer": "Subjective", "rationale": "This question is based on questions about patients ' subjective feelings and experiences in specific situations, i.e. whether the lifting of weights is difficult, rather than objective facts."}, "sentence_sentiment": {"answer": "Negative", "rationale": "Because the entry asks whether it is difficult to perform the laborious work, it implies that the possible physical discomfort or fatigue is negative."}, "language_style": {"answer": "Tactful and warm", "rationale": "The language style of the entry is euphemism. First, it uses the form of doubt, not the order or direct statement, which makes the respondents feel more respected and noticed. Second, it is more gentle and friendly, without too strong emphasis or stress, and it helps respondents to share their true feelings and experiences."}, "time_dimension": {"answer": "Present", "rationale": "The entry is for the purpose of asking if the person tested has any difficulties with the current behaviour experience and judging the time dimension as it is."}, "sentence_type": "Interrogative sentence", "pos": {"nouns": {"Actions": 2, "- It's hard": 1, "Shopping bags": 1, "Baggage": 1, "Difficulties": 1}, "verbs": {"Do it": 1, "T": 1, "I feel": 1, "Is it possible": 1}, "adjectives": {"Heavy": 1}, "adverbs": {"Like": 1}, "numerals": {"Some": 1, "Heavy": 1}, "classifiers": {"individual": 1, "Box": 1}, "pronouns": {"you": 2}}}, {"content": "2. Do you feel difficult walking at long distances?", "word_count": 13, "label": "", "sentence_structure": "Subject鈥搗erb鈥搊bject", "subjective_objective": {"answer": "Subjective", "rationale": "Because the difficulties are felt by the patient himself, they are subjective."}, "sentence_sentiment": {"answer": "Negative", "rationale": "Because the expression \"difficult\" is negative."}, "language_style": {"answer": "Calm and direct", "rationale": "Because the question is asked whether the person surveyed is having difficulty walking long distances, without using a rhetorical technique or excessive emotional rendering. At the same time, it is more objective, honest and understandable."}, "time_dimension": {"answer": "Present", "rationale": "The entry is for the testee ' s feelings on long walk, and the time dimension is now judged."}, "sentence_type": "Interrogative sentence", "pos": {"nouns": {"Distance": 1, "Walk": 1, "Difficulties": 1}, "verbs": {"I feel": 1}, "adverbs": {"Long": 1}}}, {"content": "3. Do you feel difficulties while walking at short distances?", "word_count": 16, "label": "", "sentence_structure": "Subject鈥搗erb鈥搊bject", "subjective_objective": {"answer": "Objective", "rationale": "Because the question is whether the patient has experienced difficulties in travelling at short distances, which is the patient鈥檚 personal experience and perception, and is the subject of subjective examination. But the problem does not involve any subjective evaluation or judgement, but simply raises a question of fact and is therefore considered objectively."}, "sentence_sentiment": {"answer": "Negative", "rationale": "Because the description \"difficult\" is negative, it means physical discomfort or poor physical condition, and is therefore negative emotional."}, "language_style": {"answer": "Calm and direct", "rationale": ""}, "time_dimension": {"answer": "Present", "rationale": ""}, "sentence_type": "Interrogative sentence", "pos": {"nouns": {"Outdoor": 1, "Short Distance": 1, "Walking": 1}, "verbs": {"I feel": 1, "Difficulties": 1}, "numerals": {"3": 1}}}, {"content": "Four. Do you have to lie down or sit on a chair during the day?", "word_count": 16, "label": "", "sentence_structure": "Subject鈥搗erb鈥搊bject", "subjective_objective": {"answer": "Objective", "rationale": "As this is a question of the patient ' s condition that needs to be answered, there is no need to consider the patient ' s subjective attitude and thoughts."}, "sentence_sentiment": {"answer": "Negative", "rationale": "Because bed-bed or chair-seating behaviour is usually a manifestation of physical discomfort, the entry is of a negative emotional colour."}, "language_style": {"answer": "Calm and direct", "rationale": "The entry is in a concise language and directly asks the interviewees whether they need to be bedridden or sitting on chairs during the day, without using excessive emotional or speech modification, and with a more objective and calm tone."}, "time_dimension": {"answer": "Present", "rationale": "The entry asks the testee about his current behaviour and state, and the temporal dimension judges it now. The keyword \"must\" indicates that it is a matter of the current state of affairs, while the verbs \"bed-ups\" and \"sitting on the chair\" point to the present behavior or state."}, "sentence_type": "Interrogative sentence", "pos": {"nouns": {"Daytime": 1, "you": 1, "Get in bed": 1, "On the chair": 1}, "verbs": {"I have to": 1, "Get in bed": 1, "Sit down": 1, "in": 1}, "adverbs": {"Is it possible": 2}, "numerals": {"4": 1, "in": 1}, "classifiers": {"individual": 1}}}, {"content": "5. Do you need help with food, clothing, washing or toilet?", "word_count": 19, "label": "", "sentence_structure": "Subject鈥搗erb鈥搊bject", "subjective_objective": {"answer": "Objective", "rationale": "This is an objective fact because of the patient ' s ability to live."}, "sentence_sentiment": {"answer": "Negative", "rationale": "Because the act of \"needing assistance in feeding, dressing, washing or toilet in the entry is negative and indicates that the person under investigation is less able to provide for himself."}, "language_style": {"answer": "Calm and direct", "rationale": "This is more consistent with the strict and objective requirements of the questionnaire."}, "time_dimension": {"answer": "Present", "rationale": "The entry is for the purpose of asking the person ' s current state of health and needs, and therefore the time dimension is now judged."}, "sentence_type": "Interrogative sentence", "pos": {"nouns": {"Questionnaire": 1, "Entry": 1, "others": 1, "Assistance": 1, "Food": 1, "Get dressed": 1, "Wash": 1, "To the bathroom": 1}, "verbs": {"Yes": 1, "Assistance": 1, "Food": 1, "Get dressed": 1, "Wash": 1, "To the bathroom": 1}}}, {"content": "6. Are your work or daily activities physically restricted?", "word_count": 18, "label": "", "sentence_structure": "Subject鈥搗erb鈥搊bject", "subjective_objective": {"answer": "Objective", "rationale": "Since the question is about the actual experience and situation of the patient, the objective fact that the patient's work or daily activities are really restricted by physical capacity, not just by their subjective thoughts or feelings, is what needs to be answered."}, "sentence_sentiment": {"answer": "Neutral", "rationale": "Because the entry is merely a question of whether physical limitations exist and does not involve positive or negative emotional aspects."}, "language_style": {"answer": "Calm and direct", "rationale": "The language style of the entry is more objective and straightforward, and it does not use excessive rhetorical or emotional colours, but rather raises questions in a question-and-answer manner, allowing the interviewee to answer clearly and clearly.\""}, "time_dimension": {"answer": "Past", "rationale": "The entry is for questioning the testee ' s past behaviour and the temporal dimension is a past."}, "sentence_type": "Interrogative sentence", "pos": {"nouns": {"Work": 1, "Daily activities": 1, "Physical limit": 1}, "verbs": {"Yes": 1, "Limit": 1}, "numerals": {"6": 1}}}, {"content": "7. Are your leisure and leisure activities physically restricted?", "word_count": 19, "label": "", "sentence_structure": "Subject鈥搗erb鈥搊bject", "subjective_objective": {"answer": "Subjective", "rationale": "This is a subjective question because it concerns the patient鈥檚 description of his or her subjective experience and feelings, i.e. whether they believe that their physical condition has affected or limited their amateur and leisure activities."}, "sentence_sentiment": {"answer": "Neutral", "rationale": "Since the entry does not have any positive or negative emotional aspects, it merely asks whether the testee ' s amateurity and leisure activities are restricted by physical abilities."}, "language_style": {"answer": "Calm and direct", "rationale": "The entry directly asks the other party whether the subject of amateur and leisure activities is physically restricted, does not use excessive rhetorical or emotional rendering, and is more objective in tone."}, "time_dimension": {"answer": "Present", "rationale": "The entry asks the testee whether his amateur and leisure activities are physically restricted. The keyword \"is\" means that this is a description of the current state or situation."}, "sentence_type": "Interrogative sentence", "pos": {"nouns": {"Questionnaire": 1, "Entry": 1, "Physical": 1, "Amateurity": 1, "Leisure activities": 1}, "verbs": {"Received": 1, "Limit": 1}, "numerals": {"7": 1}}}, {"content": "Have you ever felt short?", "word_count": 7, "label": "", "sentence_structure": "Subject鈥損redicate", "subjective_objective": {"answer": "Objective", "rationale": "Because feeling short is the patient's physical feeling, it's an objective description of the facts."}, "sentence_sentiment": {"answer": "Negative", "rationale": "Shortness is a manifestation of discomfort or illness and is often considered a negative emotional experience."}, "language_style": {"answer": "Calm and direct", "rationale": "The entry asks the other side in direct and concise language whether it has ever felt too short, without using excessive emotional colours or rhetorical techniques to express it in a more objective and calm manner.\""}, "time_dimension": {"answer": "Past", "rationale": "The keywords are \"had felt\" and \"short \" , which point to past experience or situation, and it is therefore possible to judge whether the person tested had experienced short experience in the past."}, "sentence_type": "Interrogative sentence", "pos": {"nouns": {"you": 1, "Short air": 1}, "verbs": {"I felt": 1}}}, {"content": "Have you ever had any pain?", "word_count": 6, "label": "", "sentence_structure": "Subject鈥損redicate", "subjective_objective": {"answer": "Subjective", "rationale": "Because pain is a subjective feeling, each person may experience and describe differently."}, "sentence_sentiment": {"answer": "Negative", "rationale": "Because pain is an unpleasant experience, associated with negative emotions."}, "language_style": {"answer": "Calm and direct", "rationale": "The entry is in direct and concise language, is not too platonic or emotional and is more objective and cold.\""}, "time_dimension": {"answer": "Past", "rationale": "The entry asks if the person tested has experienced pain in the past and the dimension of time is the past."}, "sentence_type": "Interrogative sentence", "pos": {"nouns": {"you": 1, "Pain": 1}, "verbs": {"have": 1}, "numerals": {"9": 1}}}, {"content": "Do you ever need a break?", "word_count": 7, "label": "", "sentence_structure": "Subject鈥損redicate", "subjective_objective": {"answer": "Objective", "rationale": "Because \"Whether or not to rest\" is an observable fact, not a patient's subjective attitude or emotional experience."}, "sentence_sentiment": {"answer": "Neutral", "rationale": "Because the entry is asked about an objective fact and is not of any emotional colour."}, "language_style": {"answer": "Calm and direct", "rationale": "This language style is more appropriate for a more functional and practical psychological questionnaire."}, "time_dimension": {"answer": "Past", "rationale": "The entry asks if the person tested had needed rest in the past and therefore judged it to be the past."}, "sentence_type": "Interrogative sentence", "pos": {"nouns": {"you": 1, "Rest": 1}, "verbs": {"I need it": 1, "- You're not": 1}, "numerals": {"10": 1}}}, {"content": "Did you ever feel bad about sleeping?", "word_count": 9, "label": "", "sentence_structure": "Subject鈥損redicate", "subjective_objective": {"answer": "Subjective", "rationale": "Because the quality of sleep is an individual's subjective feeling, the requirements and evaluation criteria for sleep quality may vary from one person to another. The answer to this questionnaire is based on the patient's subjective experience and feelings."}, "sentence_sentiment": {"answer": "Negative", "rationale": "Because the description of \"sleeping bad\" is negative, suggesting that the interviewee may have sleep problems."}, "language_style": {"answer": "Tactful and warm", "rationale": "This is more acclaimable, friendly and more likely to trigger resonance and collaboration among the interviewees."}, "time_dimension": {"answer": "Past", "rationale": ""}, "sentence_type": "Interrogative sentence", "pos": {"nouns": {"Sleep": 1, "Okay": 1}, "verbs": {"I felt": 1}, "numerals": {"11": 1}}}, {"content": "Twelve. Have you ever felt weak?", "word_count": 7, "label": "", "sentence_structure": "Subject鈥搗erb鈥搊bject", "subjective_objective": {"answer": "Subjective", "rationale": "The questionnaire entry is viewed from the patient鈥檚 subjective point of view. The judgement is based on the subjective feeling of weakness, not the objective fact or result."}, "sentence_sentiment": {"answer": "Negative", "rationale": "Because \"weakness\" is a negative term, implying discomfort or poor health."}, "language_style": {"answer": "Calm and direct", "rationale": "The language style of the entry is calm and straightforward, because the question is simple, without any emotional accolades, and simply asks the interviewee whether he or she has ever felt weak. This style helps the interviewee to answer the question accurately and avoid unnecessary speculation and misunderstanding."}, "time_dimension": {"answer": "Past", "rationale": "The keyword \"did feel\" means that it is a question of past experience, and the temporal dimension is a matter of past."}, "sentence_type": "Interrogative sentence", "pos": {"nouns": {"Weakness": 1}, "verbs": {"I felt": 1}, "adverbs": {"Tsang": 1}, "numerals": {"12": 1, "you": 1}}}, {"content": "Do you ever feel like you don't have an appetite?", "word_count": 9, "label": "", "sentence_structure": "Subject鈥搗erb鈥搊bject", "subjective_objective": {"answer": "Subjective", "rationale": "Because \"fear without appetite\" is a subjective experience or feeling, determined by the patient ' s consciousness and emotional state, not by the objective existence of an entity or phenomenon. The problem is therefore viewed from the patient ' s subjective perspective."}, "sentence_sentiment": {"answer": "Negative", "rationale": "Because the \"fear no appetite\" act in the entry is negative."}, "language_style": {"answer": "Tactful and warm", "rationale": "Because the entry is more personal and interactive in that it is more accessible to respondents by asking them, in a gentle and inviting manner, whether they have had no appetite, and is better able to direct respondents to open the box and express their feelings in a true way."}, "time_dimension": {"answer": "Past", "rationale": ""}, "sentence_type": "Interrogative sentence", "pos": {"nouns": {"-Adulge": 1}, "verbs": {"I'm feeling it": 1, "None": 1, "Present": 1}, "numerals": {"13": 1}, "pronouns": {"you": 1}}}, {"content": "14: Have you ever felt sick and sick?", "word_count": 10, "label": "", "sentence_structure": "Subject鈥搗erb鈥搊bject", "subjective_objective": {"answer": "Objective", "rationale": "Because vomiting is a physical feeling or symptom, it is an objective fact. Although it may be related to the emotional and psychological state of the individual, it is considered an objective matter to measure and study in this particular investigation."}, "sentence_sentiment": {"answer": "Negative", "rationale": "Because the emotional experience of \"smuggling\" in the entry is negative."}, "language_style": {"answer": "Calm and direct", "rationale": "The article is very simple and clear in its purpose, and does not use excessive adoration or emotional colours. It asks the interviewee whether he or she has ever felt sick or sick, and is sound, objective and free of any personal emotions or subjective assessment, which is consistent with the normal style of the language."}, "time_dimension": {"answer": "Past", "rationale": "The entry contains the word \"had\" and indicates that this experience has occurred in the past."}, "sentence_type": "Interrogative sentence", "pos": {"nouns": {"Gross": 1, "I'm gonna throw up": 1}, "verbs": {"Feel": 1}, "numerals": {"14": 1}}}, {"content": "Have you ever vomited?", "word_count": 6, "label": "", "sentence_structure": "Subject鈥損redicate", "subjective_objective": {"answer": "Objective", "rationale": "Because vomiting is a physical reaction of the patient and an objective fact."}, "sentence_sentiment": {"answer": "Negative", "rationale": "Because the act of vomiting in the entry is negative."}, "language_style": {"answer": "Calm and direct", "rationale": "The language style of the entry is more objective, direct, and does not involve excessive adoration and emotional rendering, and is used primarily to collect factual information or data, which are typical of the direct expression of calm."}, "time_dimension": {"answer": "Past", "rationale": "The verb \"had\" contained in the entry denotes past actions and therefore judgement is past."}, "sentence_type": "Interrogative sentence", "pos": {"nouns": {"you": 1, "- Vomit": 1}, "verbs": {"Tsang": 1, "- I've been sick": 1}, "numerals": {"15": 1}}}, {"content": "Have you had constipation?", "word_count": 6, "label": "", "sentence_structure": "Subject鈥損redicate", "subjective_objective": {"answer": "Objective", "rationale": "Because constipation is a physical condition, it does not require subjective evaluation by the patient."}, "sentence_sentiment": {"answer": "Negative", "rationale": "Because constipation is a symptom of physical discomfort, which is usually considered negative."}, "language_style": {"answer": "Tactful and warm", "rationale": "The entry is more personal and interactive when it is presented in a soft, invitation-like tone, rather than in direct statements or orders. At the same time, it does not overemphasize personal hygiene or privacy issues, but rather expresses them more neutrally and in a language style more in line with the general psychological questionnaire."}, "time_dimension": {"answer": "Past", "rationale": "The entry asked if the person tested had had a constipated experience and the temporal dimension was judged to be past."}, "sentence_type": "Interrogative sentence", "pos": {"nouns": {"Decency": 1}, "verbs": {"There was": 1}, "numerals": {"16": 1}}}, {"content": "You ever had diarrhoea?", "word_count": 6, "label": "", "sentence_structure": "Subject鈥搗erb鈥搊bject", "subjective_objective": {"answer": "Objective", "rationale": "Because diarrhoea is a real experience of patients, it does not require subjective judgement."}, "sentence_sentiment": {"answer": "Negative", "rationale": "Because \"diarrhea\" is a disease or a state of ill health, the expression in this article has a negative emotional connotation."}, "language_style": {"answer": "Calm and direct", "rationale": "The entry asks the other side whether he has had a diarrhoeal experience, does not have too many rhetorical or emotional renderings, and is more calm and straightforward in language style."}, "time_dimension": {"answer": "Past", "rationale": "The entry asks if the person tested has had any experience with diarrhoea and the temporal dimension is a past."}, "sentence_type": "Interrogative sentence", "pos": {"nouns": {"Diarrhoea": 1}, "verbs": {"you": 1, "There was": 1}, "numerals": {"17": 1}}}, {"content": "Have you ever been tired?", "word_count": 7, "label": "", "sentence_structure": "Subject鈥損redicate", "subjective_objective": {"answer": "Subjective", "rationale": "Because feeling tired is a subjective feeling, different people experience the same situation differently."}, "sentence_sentiment": {"answer": "Negative", "rationale": "Because \"fear fatigue\" is a symptom of physical discomfort, which is usually considered negative."}, "language_style": {"answer": "Tactful and warm", "rationale": "The entry uses the means of inquiry and does not give the answer directly, but rather allows the respondent to answer itself, which is more euphemistic and respectful of the other. At the same time, the term temperature of the article is not such as to put pressure or discomfort on the person under investigation and is therefore a warm and euphemismal language style."}, "time_dimension": {"answer": "Present", "rationale": "The entry asks about the current feelings or experiences of the testee, and the keyword \"twice\" indicates that the phenomenon is a past experience, but whether it is still ongoing requires the testee to judge on its own merits."}, "sentence_type": "Interrogative sentence", "pos": {"nouns": {"Weary": 1}, "verbs": {"Feel": 1, "Weary": 1}}}, {"content": "19: Does pain interfere with your daily activities?", "word_count": 11, "label": "", "sentence_structure": "Subject鈥搗erb鈥搊bject", "subjective_objective": {"answer": "Objective", "rationale": "Because \"the pain hinders your daily activity\" is a phenomenon or result that can be observed and measured, it is objective."}, "sentence_sentiment": {"answer": "Negative", "rationale": "Because the \"wortness hinders your daily activities\" in the entry is negative."}, "language_style": {"answer": "Calm and direct", "rationale": "The entry asks the respondents whether the pain affects daily activities, is objective, does not have too much emotional rendering or rhetorical, and is in keeping with the language style of calm and straightforward."}, "time_dimension": {"answer": "Present", "rationale": "The entry is for the current situation of the testee, and the keyword \"obstruction\" and \"daily activity\" indicate that this is a description of the current behaviour."}, "sentence_type": "Interrogative sentence", "pos": {"nouns": {"Pain": 1, "Obstruction": 1, "you": 1, "Day": 1, "Activities": 1}, "verbs": {"Blockage": 1, "Obstruction": 1}, "numerals": {"19": 1}}}, {"content": "20: Is it hard to concentrate on things like reading newspapers or watching TV?", "word_count": 20, "label": "", "sentence_structure": "Subject鈥搗erb鈥搊bject", "subjective_objective": {"answer": "Subjective", "rationale": "Because \"hard to concentrate\" is the patient's subjective feeling and experience."}, "sentence_sentiment": {"answer": "Negative", "rationale": "Because the \"hard to focus on\" behavior in the entry is negative."}, "language_style": {"answer": "Tactful and warm", "rationale": "The entry leads the interviewee to share his or her situation by means of inquiries, avoids direct accusations or judgements, is more friendly and friendly and better guides the interviewee to open up his or her communication."}, "time_dimension": {"answer": "Present", "rationale": "The entry asks the testee whether he is currently in a position to focus, and the time dimension is now judged. \""}, "sentence_type": "Interrogative sentence", "pos": {"nouns": {"Attention": 1, "Newspapers": 1, "Television": 1}, "verbs": {"Focus": 1, "Do it": 1, "Look": 2}, "adverbs": {"very": 1, "Hard": 1}, "numerals": {"20": 1}, "classifiers": {"Grandpa": 1}}}, {"content": "Have you ever been nervous?", "word_count": 7, "label": "", "sentence_structure": "Subject鈥損redicate", "subjective_objective": {"answer": "Subjective", "rationale": "Because stress is a subjective feeling, and because the patient ' s own experience and perception, the entry is viewed from a subjective perspective."}, "sentence_sentiment": {"answer": "Negative", "rationale": "Because the emotional experience of \"frightful\" in the entries is negative."}, "language_style": {"answer": "Calm and direct", "rationale": "The language style of the entry is relatively calm and straightforward, and the interviewee is asked in a concise and clear manner whether he or she has ever been nervous, has not had too many rhetorical or emotional features and is speaking in a more objective manner."}, "time_dimension": {"answer": "Past", "rationale": ""}, "sentence_type": "Interrogative sentence", "pos": {"nouns": {"Nervous": 1}, "verbs": {"I felt": 1}}}, {"content": "22: Have you ever been worried?", "word_count": 7, "label": "", "sentence_structure": "Subject鈥損redicate", "subjective_objective": {"answer": "Subjective", "rationale": "Because \"fear\" is a emotional reaction, a subjective feeling and attitude of the patient."}, "sentence_sentiment": {"answer": "Negative", "rationale": "Because \"fear\" is a negative sentiment."}, "language_style": {"answer": "Tactful and warm", "rationale": "The expression is more personal and interactive, in a gentle and inviting tone, rather than in direct statements or orders. At the same time, it makes the respondents more willing to share their true feelings."}, "time_dimension": {"answer": "Past", "rationale": "The entry asks if the person tested had past experience of fear and therefore is based on past."}, "sentence_type": "Interrogative sentence", "pos": {"nouns": {"you": 1, "I'm worried": 1}, "verbs": {"I felt": 1}, "adverbs": {"- You're not": 1}, "numerals": {"22": 1}}}, {"content": "Have you ever felt so angry?", "word_count": 9, "label": "", "sentence_structure": "Subject鈥搗erb鈥搊bject", "subjective_objective": {"answer": "Subjective", "rationale": "Because \"fEss\" is usually a patient's subjective feeling or experience."}, "sentence_sentiment": {"answer": "Negative", "rationale": "Because \"excited\" is a negative emotional reaction."}, "language_style": {"answer": "Tactful and warm", "rationale": "The phrase \"Have you ever been more angry?\" is a more euphemistic expression of the researchers' desire to know whether the person under investigation is angry."}, "time_dimension": {"answer": "Past", "rationale": "The entry asks whether the person tested has experienced an emotional reaction, i.e. an emotional feeling, which is about the past and is therefore judged as \"the past\"."}, "sentence_type": "Interrogative sentence", "pos": {"nouns": {"you": 1, "Excited": 1}, "verbs": {"I felt": 1}, "adjectives": {"Easier than I thought": 1}, "adverbs": {"Easy": 1}, "numerals": {"23": 1}}}, {"content": "24. Have you ever felt depressed?", "word_count": 9, "label": "", "sentence_structure": "Subject鈥損redicate", "subjective_objective": {"answer": "Subjective", "rationale": "Because \"frightful\" is a patient's subjective feeling or experience."}, "sentence_sentiment": {"answer": "Negative", "rationale": "Because \"frightful\" in the entry is a negative emotional state."}, "language_style": {"answer": "Tactful and warm", "rationale": "The entry is more adept and interactive when it is spoken in a gentle and inviting manner, rather than by direct statements or orders."}, "time_dimension": {"answer": "Present", "rationale": "The entry asks the subject about the current emotional state, the keywords being \"had felt\" and \"now\" and they all point to the current, ongoing situation."}, "sentence_type": "Interrogative sentence", "pos": {"nouns": {"Emotion": 1, "Down": 1}, "verbs": {"I feel": 1, "- You're not": 1}, "numerals": {"24": 1}}}, {"content": "Have you ever felt difficulty remembering?", "word_count": 10, "label": "", "sentence_structure": "Subject鈥損redicate", "subjective_objective": {"answer": "Subjective", "rationale": "Because \"rememberance\" is a subjective feeling and experience, the perception and experience of the same thing may differ from one person to another. It is therefore a question of examining whether the patient has ever had such a subjective feeling."}, "sentence_sentiment": {"answer": "Negative", "rationale": "Because the \"remembering difficulties\" in the entries are negative."}, "language_style": {"answer": "Calm and direct", "rationale": "The entry is a direct question-and-answer dialogue, with a calm and objective tone, without too many emotional or rhetorical techniques, and is able to convey the message that is sought with clarity."}, "time_dimension": {"answer": "Past", "rationale": ""}, "sentence_type": "Interrogative sentence", "pos": {"nouns": {"Questionnaire Entry": 1, "Note": 1, "Difficulties": 1}, "verbs": {"I feel": 1, "Note": 1}, "numerals": {"25": 1, "you": 1}, "pronouns": {"you": 1}}}, {"content": "26. Does your physical condition or the process of treatment hinder your family life?", "word_count": 21, "label": "", "sentence_structure": "Subject鈥搗erb鈥搊bject", "subjective_objective": {"answer": "Subjective", "rationale": "This is about the patient鈥檚 personal views and emotional reactions, and therefore subjective."}, "sentence_sentiment": {"answer": "Negative", "rationale": "Because the word \"obstruction\" in the entry expresses a negative emotional attitude."}, "language_style": {"answer": "Calm and direct", "rationale": "The entry is simple and simple by means of direct inquiries, with no excessive emotional or rhetorical style, and appears to be more objective and professional."}, "time_dimension": {"answer": "Past", "rationale": "The entry is for the testee to judge the past in terms of the past behavior and temporal dimensions."}, "sentence_type": "Interrogative sentence", "pos": {"nouns": {"Physical condition": 1, "The process of treatment": 1, "Family life": 1}, "verbs": {"Obstruction": 1}, "numerals": {"26": 1}}}, {"content": "27. Are your physical condition or treatments a hindrance to your social activities?", "word_count": 21, "label": "", "sentence_structure": "Subject鈥搗erb鈥搊bject", "subjective_objective": {"answer": "Subjective", "rationale": "This is because it is an examination of the patient ' s physical condition or whether the treatment process has had an impact on their social activities, based on the patient ' s personal feelings and experiences and therefore is subjective in nature."}, "sentence_sentiment": {"answer": "Negative", "rationale": "Because the act of \"obstructing your social activities\" is negative."}, "language_style": {"answer": "Tactful and warm", "rationale": "This is a more humane and respectful way of expressing a good Question and Answer atmosphere."}, "time_dimension": {"answer": "Present", "rationale": "The entry now asks whether the person tested is now having an impact on his or her social activities due to his or her physical condition or the process of treatment, and therefore it is now judged."}, "sentence_type": "Interrogative sentence", "pos": {"nouns": {"Physical condition": 1, "The process of treatment": 1, "Social events": 1}, "verbs": {"Obstruction": 1}, "numerals": {"27": 1}}}, {"content": "28. Are you in financial distress because of your physical condition or the process of treatment?", "word_count": 21, "label": "", "sentence_structure": "Subject鈥搗erb鈥搊bject", "subjective_objective": {"answer": "Objective", "rationale": "Because the patient's financial difficulties are the objective facts arising from his or her state of health or the course of treatment.鈥?}, "sentence_sentiment": {"answer": "Negative", "rationale": "Because the result of the entry \"economically difficult\" is negative."}, "language_style": {"answer": "Calm and direct", "rationale": "The entry is based on a direct inquiry into the person ' s state of health or whether the treatment process caused him or her financial difficulties, the fact that he or she did not use excessive speech techniques or emotional rendering, the soundness of the speech is more objective, and the language style is therefore considered to be calm and straightforward."}, "time_dimension": {"answer": "Past", "rationale": "The entry asked whether the testee ' s past physical condition or treatment caused him or her financial hardship and the temporal dimension was a matter of past."}, "sentence_type": "Interrogative sentence", "pos": {"nouns": {"you": 1, "Physical condition": 1, "The process of treatment": 1, "Economic difficulties": 1}, "verbs": {"Cause": 1}, "numerals": {"28": 1}}}, {"content": "29. How do you assess your overall health over the past week?", "word_count": 18, "label": "", "sentence_structure": "Subject鈥搗erb鈥搊bject", "subjective_objective": {"answer": "Subjective", "rationale": "Since the question is examined by the patient in terms of his or her personal assessment and perception of his or her health during the past week, it is examined from the patient ' s subjective perspective."}, "sentence_sentiment": {"answer": "Positive", "rationale": "Because \"the overall state of health\" is a positive word, which means an assessment of the state of the body."}, "language_style": {"answer": "Tactful and warm", "rationale": "The entry is more euphemistic and gentle, using an invitation to ask the other side about the assessment of his or her overall health over the past week, without direct orders or evaluation."}, "time_dimension": {"answer": "Past", "rationale": ""}, "sentence_type": "Interrogative sentence", "pos": {"nouns": {"Health status": 1, "Week": 1}, "verbs": {"Assessment": 1, "Past": 1}, "adverbs": {"Overall": 1}, "numerals": {"29": 1, "A week": 1}}}, {"content": "30. How do you assess your overall quality of life over the past week?", "word_count": 18, "label": "", "sentence_structure": "Subject鈥搗erb鈥搊bject", "subjective_objective": {"answer": "Subjective", "rationale": "Because the evaluation of the overall quality of life is a subjective feeling and attitude of the patient."}, "sentence_sentiment": {"answer": "Neutral", "rationale": "Because the entry is asked for objective quality of life assessments and does not contain any emotional colours."}, "language_style": {"answer": "Calm and direct", "rationale": "The language style of the entry is relatively objective and direct, and there is no excessive emotional rendering or rhetorical technique, mainly by asking questions to enable the respondents to assess the quality of life over the past week."}, "time_dimension": {"answer": "Past", "rationale": ""}, "sentence_type": "Interrogative sentence", "pos": {"nouns": {"Questionnaire": 1, "Entry": 1, "Past": 1, "A week": 1, "Overall quality": 1, "Life": 1}, "verbs": {"Assessment": 1, "How's that": 1}, "adverbs": {"Past": 1, "A week": 1, "Overall": 1, "How's that": 1}, "numerals": {"30": 1}}}, {"content": "31. Do you cough often?", "word_count": 6, "label": "", "sentence_structure": "Subject鈥損redicate", "subjective_objective": {"answer": "Subjective", "rationale": "The questionnaire entry is viewed from the patient's subjective point of view. Because cough is the patient's physical condition, it requires a patient's own report to arrive at the answer, not an objective fact that can be observed. The answer is therefore \"subjective.\""}, "sentence_sentiment": {"answer": "Neutral", "rationale": "Because the entry does not have any emotional color."}, "language_style": {"answer": "Calm and direct", "rationale": "The entry is asked in the form of a direct question whether the person surveyed has a regular cough, without any additional emotional colour or qualifier, and with a more direct and concise language style."}, "time_dimension": {"answer": "Past", "rationale": ""}, "sentence_type": "Interrogative sentence", "pos": {"nouns": {"you": 1, "Cough": 1}, "verbs": {"Regular": 1, "Do it": 1}, "numerals": {"31": 1}}}, {"content": "You cough blood?", "word_count": 8, "label": "", "sentence_structure": "Subject鈥損redicate", "subjective_objective": {"answer": "Objective", "rationale": "Because coughing (sipple blood) is a possible symptom of the patient, it is an observed phenomenon and is therefore objective."}, "sentence_sentiment": {"answer": "Negative", "rationale": "As this entry relates to physical discomfort, it may cause negative emotions and concerns."}, "language_style": {"answer": "Calm and direct", "rationale": "The entry asks the other person whether he coughs blood, is simple and clear, is not too pedicular or emotional and is in a calm and direct manner."}, "time_dimension": {"answer": "Present", "rationale": "So, \"32. Did you cough blood?\" This questionnaire item is now."}, "sentence_type": "Interrogative sentence", "pos": {"nouns": {"you": 1, "Cough blood": 1, "Boom": 1, "Blood": 1}, "verbs": {"you": 1, "Ahem": 1, "- Yeah": 1}, "adjectives": {"Snack": 1}, "numerals": {"32": 1}}}, {"content": "Did you feel short on your break?", "word_count": 9, "label": "", "sentence_structure": "Subject鈥損redicate", "subjective_objective": {"answer": "Subjective", "rationale": "Because \"fear\" represents the patient's own feelings and subjective experience, it's a subjective issue."}, "sentence_sentiment": {"answer": "Negative", "rationale": "Because \"short\" is usually seen as an expression of discomfort or disease, this emotional attitude is negative."}, "language_style": {"answer": "Calm and direct", "rationale": "The language style of the entry is calm, as it is a clear, concise question, not using too many qualifiers or emotional colours, but asking the respondents in an objective way about their feelings. This language style helps to ensure that the questionnaire is accurate and effective and that more realistic answers are available."}, "time_dimension": {"answer": "Present", "rationale": "The entry is for the testee's current state, so it's judged to be \"now\"."}, "sentence_type": "Interrogative sentence", "pos": {"nouns": {"Rest": 1, "Time": 1, "Short air": 1}, "verbs": {"you": 1, "I feel": 1, "- You're not": 1}, "numerals": {"33": 1}}}, {"content": "34. Did you feel short on your walk?", "word_count": 9, "label": "", "sentence_structure": "Subject鈥搗erb鈥搊bject", "subjective_objective": {"answer": "Objective", "rationale": "Because \"fright short\" is the biological feeling of the patient, and it's an objective observation."}, "sentence_sentiment": {"answer": "Negative", "rationale": "Because \"fright short\" is a reaction that is physically uncomfortable and is usually considered negative emotions or experiences."}, "language_style": {"answer": "Calm and direct", "rationale": "The language style of the entry is calm and straightforward. It is a closed question, using clear language and asking directly whether the interviewee feels short when walking, and does not involve too much emotional or attitudinal expression."}, "time_dimension": {"answer": "Present", "rationale": "The entry is for the present feeling or experience of the testee, and the temporal dimension is judged as now. \"Do you feel short on walking?\" The words \"fear\" and \"walk\" indicate what is happening, so it is judged as now."}, "sentence_type": "Interrogative sentence", "pos": {"nouns": {"you": 1, "Walking": 1, "Short air": 1}, "verbs": {"I feel": 1}, "adverbs": {"Time": 1}, "numerals": {"34": 1}}}, {"content": "Do you feel short on the stairs?", "word_count": 10, "label": "", "sentence_structure": "Subject鈥搗erb鈥搊bject", "subjective_objective": {"answer": "Objective", "rationale": "Because \"fright short\" is a quantifiable experience that doesn't need to be judged by the patient's subjective feelings."}, "sentence_sentiment": {"answer": "Negative", "rationale": "Because the \"fright short\" act in the entry is negative."}, "language_style": {"answer": "Calm and direct", "rationale": "The language style of the entry is more objective and concise, and the person is asked directly, by asking questions, whether there is a short-lived situation when climbing the stairs, without too much embellishment or emotional rendering, and in accordance with the linguistic characteristics and requirements of the questionnaire."}, "time_dimension": {"answer": "Present", "rationale": "The keyword \"fright short\" and the verb \"crawling stairs\" both indicate the actions being carried out by the testees, and the temporal dimensions are now judged."}, "sentence_type": "Interrogative sentence", "pos": {"nouns": {"you": 1, "Stairs": 1, "Short air": 1}, "verbs": {"Climb": 1, "I feel": 1, "- You're not": 1}, "adverbs": {"Time": 1}, "numerals": {"35": 1}}}, {"content": "36: Have you had any oral or tongue pain?", "word_count": 11, "label": "", "sentence_structure": "Subject鈥搗erb鈥搊bject", "subjective_objective": {"answer": "Subjective", "rationale": "Because \"does it feel like it's a pain in the mouth or tongue\" is a subjective feeling that requires a patient to report himself to get the answer."}, "sentence_sentiment": {"answer": "Negative", "rationale": "Because it involves physical discomfort and pain and has negative emotional connotations."}, "language_style": {"answer": "Calm and direct", "rationale": "The entry is based on a more neutral and direct approach, with no excessive use of qualifiers or rhetorical techniques and a more calm and straightforward tone."}, "time_dimension": {"answer": "Present", "rationale": "The keyword \"had\" indicates a past act or circumstance, but the conduct or situation continues to this point and may continue, so that the temporal dimension of the article can be judged to be the present."}, "sentence_type": "Interrogative sentence", "pos": {"nouns": {"Oral": 1, "tongue": 1, "Pain": 1}, "verbs": {"Yes": 1}, "numerals": {"36": 1}}}, {"content": "You ever have trouble swallowing?", "word_count": 8, "label": "", "sentence_structure": "Subject鈥搗erb鈥搊bject", "subjective_objective": {"answer": "Objective", "rationale": "Because \"ingestion difficulty\" is an objective physical condition, not a patient's subjective feeling or attitude. This is the result of a doctor's examination or a patient's self-observation, not the patient's own feelings or ideas."}, "sentence_sentiment": {"answer": "Negative", "rationale": "Because the difficulty of swallowing is usually seen as a negative physical condition."}, "language_style": {"answer": "Tactful and warm", "rationale": "The entry is more personal and interactive when it is spoken in a gentle, invitation-like tone, rather than in direct statements or orders. At the same time, the tone of the issue is more objective and neutral, and there is no excessive emotional or guiding expression that allows respondents to be more comfortable and more accessible to accurate information in their responses."}, "time_dimension": {"answer": "Present", "rationale": ""}, "sentence_type": "Interrogative sentence", "pos": {"nouns": {"you": 1, "It's hard to swallow": 1}, "verbs": {"have": 1}, "numerals": {"37": 1}}}, {"content": "You ever get numbed or stingy?", "word_count": 10, "label": "", "sentence_structure": "Subject鈥損redicate", "subjective_objective": {"answer": "Subjective", "rationale": "Because \"sicking/stingling\" is the experience and feeling that patients may experience, and it is a subjective experience and perception."}, "sentence_sentiment": {"answer": "Negative", "rationale": "Because \"sicking/stingling\" is a symptom of physical discomfort, it is generally considered a negative experience."}, "language_style": {"answer": "Calm and direct", "rationale": "The entry asks the person surveyed whether he has ever experienced a ting or a sting, in a direct and clear manner, and is more objective in language style, with no excessive adoration and emotional colour.\""}, "time_dimension": {"answer": "Present", "rationale": "The keyword \"yes\" and \"over\" means that the act occurred in the past and is over, but still has an impact on it, so the temporal dimension is now."}, "sentence_type": "Interrogative sentence", "pos": {"nouns": {"Hands and feet": 1, "I'm numb": 1, "Stings": 1}, "verbs": {"have": 1}, "adverbs": {"/": 1}, "numerals": {"38": 1}}}, {"content": "Have you ever had a hair loss?", "word_count": 6, "label": "", "sentence_structure": "Subject鈥損redicate", "subjective_objective": {"answer": "Objective", "rationale": "Since a hysteria is an objective physical phenomenon, the patient ' s subjective perception is not required to determine its existence."}, "sentence_sentiment": {"answer": "Neutral", "rationale": "Because the entry is merely a question of fact and does not involve any emotional colour."}, "language_style": {"answer": "Calm and direct", "rationale": ""}, "time_dimension": {"answer": "Present", "rationale": "The keyword \"had had a hair off\" occurred in the past, but the question was about the current situation of the testee and was therefore judged to be now."}, "sentence_type": "Interrogative sentence", "pos": {"nouns": {"Take your hair off": 1}, "verbs": {"Pass": 1, "have": 1}, "adverbs": {"- You're not": 1}, "numerals": {"39": 1}}}, {"content": "Have you had chest pains?", "word_count": 6, "label": "", "sentence_structure": "Subject鈥損redicate", "subjective_objective": {"answer": "Objective", "rationale": "Because chest pain is a biological phenomenon that can be observed and measured, not a subjective feeling or attitude of the patient. The problem is therefore viewed from the perspective of the patient."}, "sentence_sentiment": {"answer": "Negative", "rationale": "Since chest pain is a symptom of disease, it is often considered to be a sign of negative experience or health problems."}, "language_style": {"answer": "Calm and direct", "rationale": "The entry uses direct language to ask the interviewees whether they have experienced chest pains, and does not use any rhetorical or emotional colours to render the problem serious and to be more objective in its tone."}, "time_dimension": {"answer": "Past", "rationale": ""}, "sentence_type": "Interrogative sentence", "pos": {"nouns": {"- Chestaches": 1}, "verbs": {"have": 1}, "classifiers": {"individual": 1}, "pronouns": {"you": 1}}}, {"content": "41. Have you had any pain in your arm or shoulder?", "word_count": 11, "label": "", "sentence_structure": "Subject鈥損redicate", "subjective_objective": {"answer": "Objective", "rationale": "Since the existence of pain in the arm or shoulder is a de facto state that can be directly observed and measured, it is an objective question to be examined."}, "sentence_sentiment": {"answer": "Negative", "rationale": "Because the word \"in pain\" in the entry is usually considered negative."}, "language_style": {"answer": "Calm and direct", "rationale": "The entry is in direct, concise language and asks the person surveyed whether he or she has suffered pain in his or her arm or shoulder, is not using a speech-reading technique or emotional rendering, and is more objective and neutral."}, "time_dimension": {"answer": "Past", "rationale": "The entry asks about past behavior and the keyword is \"had.\""}, "sentence_type": "Interrogative sentence", "pos": {"nouns": {"Arms": 1, "Shoulders": 1, "Pain": 1}, "verbs": {"have": 1}, "numerals": {"41": 1}}}, {"content": "42 Have you experienced pain in other parts of your body? If so, write down the parts:", "word_count": 21, "label": "", "sentence_structure": "Subject鈥搗erb鈥搊bject", "subjective_objective": {"answer": "Objective", "rationale": "The entry examines whether the patient has experienced pain in other parts of the body, an objective fact that is not influenced by the patient鈥檚 subjective consciousness. Although the patient needs to write down the part of the pain he feels, it is only a description of the objective facts that have occurred and is still an objective examination."}, "sentence_sentiment": {"answer": "Neutral", "rationale": "Because the entry does not have any emotional color."}, "language_style": {"answer": "Calm and direct", "rationale": "This language style is more calm and objective, and does not have too many emotional or speech skills to use in research and research."}, "time_dimension": {"answer": "Present", "rationale": "The entry asks the testee about his current state of health, so the time dimension is now judged. The words \"Is there ever\" and \"Whether to write a part\" indicate that this is a question of current experience or state."}, "sentence_type": "Interrogative sentence", "pos": {"nouns": {"Body": 1, "Part": 1, "Pain": 1}, "verbs": {"have": 1, "Write": 1, "Out": 1}, "numerals": {"42": 1}, "classifiers": {"individual": 1}, "pronouns": {"you": 1}}}, {"content": "Have you ever taken painkillers?", "word_count": 8, "label": "", "sentence_structure": "Subject鈥搗erb鈥搊bject", "subjective_objective": {"answer": "Objective", "rationale": "Since the fact that painkillers were used was an objective fact of the patient ' s experience, it did not require a subjective judgement from the patient."}, "sentence_sentiment": {"answer": "Neutral", "rationale": "Because the entry merely asked whether the person surveyed had ever taken a drug, it did not express a positive or negative attitude towards the use of the drug."}, "language_style": {"answer": "Calm and direct", "rationale": "This entry is based on a straightforward language style, which is simple enough to ask the interviewee whether he has used painkillers, without using a rhetorical technique or emotional rendering."}, "time_dimension": {"answer": "Past", "rationale": ""}, "sentence_type": "Interrogative sentence", "pos": {"nouns": {"Painkillers": 1}, "verbs": {"I've taken it": 1}, "numerals": {"43": 1}}}]}

{"questionnaire_title": "The student relationship test questionnaire.", "questionnaire_category": "Behavioral questionnaire", "item_count": 28, "emotion_shift_overall": "From negative to positive", "language_style_overall": "Tactful and warm", "items": [{"content": "(1) There is some difficulty in saying what is troubling you.", "word_count": 11, "label": "", "sentence_structure": "Subject鈥搗erb鈥揷omplement", "subjective_objective": {"answer": "Subjective", "rationale": "Because it's the patient's subjective feeling and thoughts about his own troubles."}, "sentence_sentiment": {"answer": "Negative", "rationale": "Because the term \"problems\" in the entry gives people a negative, uncomfortable feeling."}, "language_style": {"answer": "Tactful and warm", "rationale": "This expression is more respectful of the interviewee鈥檚 feelings and supports."}, "time_dimension": {"answer": "Present", "rationale": "The entry for this questionnaire therefore judged the time dimension of the testee ' s constant difficulty in being able to speak to others."}, "sentence_type": "Interrogative sentence", "pos": {"nouns": {"self": 1, "Trouble": 1}, "verbs": {"have": 1, "It's hard to say": 1}}}, {"content": "(2) It is not natural to meet strangers.", "word_count": 11, "label": "", "sentence_structure": "Subject鈥搗erb鈥揷omplement", "subjective_objective": {"answer": "Subjective", "rationale": "Because \"feel\" is usually a personal subjective feeling."}, "sentence_sentiment": {"answer": "Negative", "rationale": "Because the description of \"frightful\" in the entry expresses negative emotions or feelings."}, "language_style": {"answer": "Tactful and warm", "rationale": ""}, "time_dimension": {"answer": "Present", "rationale": "\"It is not natural to meet strangers\" describes a subjective or emotional experience that exists now, so that the temporal dimension of the article is now."}, "sentence_type": "Interrogative sentence", "pos": {"nouns": {"Stranger": 1, "Meet me": 1, "Feel": 1, "Naturally": 1}, "verbs": {"and": 1, "Meet me": 1, "Feel": 1, "Naturally": 1}, "adjectives": {"not": 1}, "numerals": {"2": 1}}}, {"content": "(3) Exult envy and envy.", "word_count": 10, "label": "", "sentence_structure": "Subject鈥搗erb鈥揷omplement", "subjective_objective": {"answer": "Subjective", "rationale": "Because envy and envy for others' feelings and thoughts are subjective attitudes of patients."}, "sentence_sentiment": {"answer": "Negative", "rationale": "Because \"envious and jealous\" is a manifestation of negative emotions, and it is a negative emotional color."}, "language_style": {"answer": "Tactful and warm", "rationale": "The entry uses a more euphemistic term, \"envious\" and \"envious,\" rather than a term that is directly negative, which reduces each other's resistance and encourages them to share their true feelings. At the same time, the article is more intimate and caring."}, "time_dimension": {"answer": "Future", "rationale": "The keyword \"excessive envy and envy\" refers to possible future behaviour, so the entry expresses vision and expectations for the future and judges it as the future."}, "sentence_type": "Negative declarative sentence", "pos": {"nouns": {"Grandpa": 1, "Envious": 1, "Jealousy": 1, "others": 1}, "verbs": {"That's too much": 1, "have": 1, "Envious": 1, "Jealousy": 1}, "adjectives": {"It's too much": 1}, "numerals": {"III": 1}}}, {"content": "(4) Too little dealing with heterosexuals.", "word_count": 7, "label": "", "sentence_structure": "Subject鈥搗erb鈥揷omplement", "subjective_objective": {"answer": "Subjective", "rationale": "Because \"too little heterosexuality\" is a subjective feeling or evaluation that reflects the patient's perception and experience of heterosexuality."}, "sentence_sentiment": {"answer": "Negative", "rationale": "Because the \"too little heterosexuality\" in the entries is negative."}, "language_style": {"answer": "Tactful and warm", "rationale": "The entry is more personal and interactive when it is spoken in a gentle and inviting manner, rather than in direct statements or orders. At the same time, it expresses the seriousness of the problem by using the word \"too\" without too much emphasis on the wrongs of the other, reflecting a euphemism in the language style."}, "time_dimension": {"answer": "Present", "rationale": ""}, "sentence_type": "Negative declarative sentence", "pos": {"nouns": {"Heterosexual": 1, "- To be with someone": 1}, "verbs": {"Too much": 1, "Less": 1}, "numerals": {"4": 1}}}, {"content": "(5) Difficulties in the continuation of talks.", "word_count": 12, "label": "", "sentence_structure": "Subject鈥搗erb鈥揷omplement", "subjective_objective": {"answer": "Subjective", "rationale": "Because feeling difficult is a subjective feeling or experience."}, "sentence_sentiment": {"answer": "Negative", "rationale": "Because the expression \"difficult\" in the entry is negative."}, "language_style": {"answer": "Tactful and warm", "rationale": ""}, "time_dimension": {"answer": "Past", "rationale": "The entry describes the feelings and experiences of the subjects during the talks, without a clear time frame, and therefore does not allow for a judgement as to whether the time dimension is past, present or future."}, "sentence_type": "Negative declarative sentence", "pos": {"nouns": {"Meetings": 2, "Difficulties": 1}, "verbs": {"to": 1, "I feel": 1, "Continuous": 1}, "adjectives": {"It's not going to stop": 1}, "numerals": {"5": 1}}}, {"content": "(6) In social settings, tensions are felt.", "word_count": 9, "label": "", "sentence_structure": "Subject鈥揷opula鈥損redicative", "subjective_objective": {"answer": "Subjective", "rationale": "Because stress is a subjective feeling, reflecting the emotional state of the patient."}, "sentence_sentiment": {"answer": "Negative", "rationale": "Because the \"frightful\" act is negative."}, "language_style": {"answer": "Calm and direct", "rationale": "The entry directly asked the interviewees whether they were nervous in social settings, were plain and clear, were not over-repeated or emotional, and were more objective and direct."}, "time_dimension": {"answer": "Present", "rationale": "The entry asks the testee how he feels in a social setting, and the time dimension is now judged."}, "sentence_type": "Affirmative declarative sentence", "pos": {"nouns": {"Socially": 1, "Nervous": 1}, "verbs": {"I feel": 1}}}, {"content": "(7) He often hurts others.", "word_count": 6, "label": "", "sentence_structure": "Subject鈥損redicate", "subjective_objective": {"answer": "Subjective", "rationale": "Because \"often hurting others\" is a person's behaviour and attitude, it reflects the patient's mental state and orientation and is therefore subjective."}, "sentence_sentiment": {"answer": "Negative", "rationale": "Because the \"too often hurting\" act in the entry is negative."}, "language_style": {"answer": "Calm and direct", "rationale": "The entry asks the interviewees in a direct and clear manner whether they are often harmful to others, with a more calm and objective language style, with no excessive emotional rendering or rhetorical tactics, and with a greater focus on facts and evidence."}, "time_dimension": {"answer": "Past", "rationale": "However, the term \"normal\" suggests that the conduct is recurrent. This implies that it may have occurred in the past and may continue to exist, or even continue to be in the future. We are therefore unable to say precisely whether the temporal dimension of the entry is past, present or future, but should be understood as that the act may occur at some point in the past, present and future. In conclusion, we cannot determine the temporal dimension of the problem, as required by the title, but rather need to understand it as a constant description of behaviour."}, "sentence_type": "Negative declarative sentence", "pos": {"nouns": {"Hurt": 1, "others": 1}, "verbs": {"Often": 1}}}, {"content": "(8) It is not natural to have sexual relations with opposite sex.", "word_count": 10, "label": "", "sentence_structure": "Subject鈥搗erb鈥揷omplement", "subjective_objective": {"answer": "Subjective", "rationale": "Because it's the subjective feeling and experience inside the patient."}, "sentence_sentiment": {"answer": "Negative", "rationale": "Because the emotional attitude of \"unnatural\" in the entries is negative."}, "language_style": {"answer": "Tactful and warm", "rationale": ""}, "time_dimension": {"answer": "Present", "rationale": ""}, "sentence_type": "Negative declarative sentence", "pos": {"nouns": {"Heterosexual": 1, "- To be with someone": 1}, "verbs": {"Travel": 1, "Feel": 1, "Naturally": 1}, "numerals": {"8": 1}}}, {"content": "(9) With a large group of friends, it is common to feel lonely or lost.", "word_count": 17, "label": "", "sentence_structure": "Subject鈥搗erb鈥揷omplement", "subjective_objective": {"answer": "Subjective", "rationale": "Because \"sorture or loss\" is the feeling and experience inside the patient, and it is the subjective emotion of the patient."}, "sentence_sentiment": {"answer": "Negative", "rationale": "Because \"silent or lost\" in the entry is a negative emotional experience."}, "language_style": {"answer": "Tactful and warm", "rationale": "The entry describes in a more soft way the potential of the person surveyed to feel in a social setting, without using strong negative or positive language, but rather expressing concern and understanding about the situation. This tone is more acceptable and willing to share their true feelings."}, "time_dimension": {"answer": "Present", "rationale": ""}, "sentence_type": "Imperative sentence", "pos": {"nouns": {"friends": 1, "Loneliness": 1, "Lost": 1}, "verbs": {"in": 1, "Together": 1, "I feel": 1}, "adverbs": {"Regular": 1}, "numerals": {"One": 2}}}, {"content": "(10) Extremely vulnerable.", "word_count": 4, "label": "", "sentence_structure": "Subject鈥揷opula鈥損redicative", "subjective_objective": {"answer": "Subjective", "rationale": "Because extreme vulnerability is the feeling and experience of the patient, it is a subjective description."}, "sentence_sentiment": {"answer": "Negative", "rationale": "Because the \"vulnerability\" act in the entry is negative."}, "language_style": {"answer": "Calm and direct", "rationale": "The language style of the entry is calm, as it is presented in the form of simple direct questions, without any verbs or emotional colours used to describe the feeling. It directly asks whether the interviewee often feels vulnerable, is neutral and objective, is free of emotion or implication, and is easy to understand and answer."}, "time_dimension": {"answer": "Present", "rationale": ""}, "sentence_type": "Negative declarative sentence", "pos": {"verbs": {"Easy": 1}, "adjectives": {"Very easy": 1, "Unh": 1}}}, {"content": "(11) Inhabitation with others.", "word_count": 9, "label": "", "sentence_structure": "Subject鈥搗erb鈥揷omplement", "subjective_objective": {"answer": "Subjective", "rationale": "Because it is a subjective feeling and attitude of one."}, "sentence_sentiment": {"answer": "Negative", "rationale": "Because the \"not living together\" act in the entry is negative."}, "language_style": {"answer": "Tactful and warm", "rationale": "The entry is more adept and interactive when it is spoken in a gentle and inviting manner, rather than by direct statements or orders."}, "time_dimension": {"answer": "Past", "rationale": ""}, "sentence_type": "Negative declarative sentence", "pos": {"nouns": {"Questionnaire": 1, "Entry": 1, "others": 1}, "verbs": {"No, I can't": 1, "Get along": 1}, "numerals": {"11": 1}}}, {"content": "(12) It is not known how it is appropriate to live with opposite sex.", "word_count": 14, "label": "", "sentence_structure": "Subject鈥搗erb鈥揷omplement", "subjective_objective": {"answer": "Subjective", "rationale": "Subjective, because it's the subjective feeling and distress of the patient about how he and he are living together."}, "sentence_sentiment": {"answer": "Negative", "rationale": "Because the \"unsure\" attitude in the entry is negative."}, "language_style": {"answer": "Tactful and warm", "rationale": "The entry is more gentle and gentle in its language, using a more relaxed, friendly tone to ask if the other person is confused or confused when he or she is having sex with a different sex. This expression makes it easier for the interviewee to open his or her heart and to share his or her real thoughts."}, "time_dimension": {"answer": "Present", "rationale": "\"(12) Does not know\" indicates that the testee is in this situation, that \"how to live with opposite sex is appropriate\" is a description of the problem, highlighting the problems and shortcomings that the testee may have in dealing with heterosexual relationships, and it is therefore possible to judge that the entry is about the present problem."}, "sentence_type": "Interrogative sentence", "pos": {"nouns": {"Heterosexual": 1, "Get along": 1, "Stop it": 1}, "verbs": {"Yes": 1, "How's that": 1, "Stop it": 1}, "adverbs": {"How's that": 1}, "numerals": {"12": 1}}}, {"content": "(13) When an unfamiliar person tells himself of his life and asks for sympathy, he is often uncomfortable.", "word_count": 32, "label": "", "sentence_structure": "Subject鈥搗erb鈥搊bject", "subjective_objective": {"answer": "Subjective", "rationale": "Because this is a patient's description of his or her own feelings and is subjective."}, "sentence_sentiment": {"answer": "Negative", "rationale": "Because it describes an uncomfortable emotional experience, which is \"unsatisfied,\" which is negative."}, "language_style": {"answer": "Tactful and warm", "rationale": "The entry is more personal and interactive when it is spoken in a euphemistic way, rather than in direct statements or orders. At the same time, the word \"whether or not\" is used to guide the other party to answer questions, to make questionnaires more open and inclusive and to facilitate access to more realistic and comprehensive information."}, "time_dimension": {"answer": "Present", "rationale": "Now, the entry describes the feelings and reactions of the testee in the face of a particular situation, \"often\" indicating that this happens very often, and \"likely feeling uncomfortable\" is the emotional experience of the moment. So the time dimension of the article is now."}, "sentence_type": "Interrogative sentence", "pos": {"nouns": {"person": 1, "Lifetime": 1, "Compassion": 1, "self": 2}, "verbs": {"to": 1, "Speech": 1, "I think": 1, "Regular": 1, "I'm not comfortable": 1}, "adjectives": {"I know you": 1, "I'm not comfortable": 1}, "adverbs": {"When": 1, "Is it possible": 1}, "numerals": {"13": 1}}}, {"content": "(14) Fear of what others may think of themselves.", "word_count": 12, "label": "", "sentence_structure": "Subject鈥搗erb鈥揷omplement", "subjective_objective": {"answer": "Subjective", "rationale": "Because \"fear\" and \"impression\" are feelings or thoughts inside the patient, and they are subjective."}, "sentence_sentiment": {"answer": "Negative", "rationale": "Because \"fear\" is usually considered a negative state of mind."}, "language_style": {"answer": "Tactful and warm", "rationale": "The entry is more adept and interactive when it is spoken in a gentle and inviting manner, rather than by direct statements or orders."}, "time_dimension": {"answer": "Present", "rationale": "Now the \"fear\" in the entry expresses the concerns and anxiety of the testee about the current situation, while the \"images\" indicate that the testee is thinking about the views or evaluations of others. The questionnaire is therefore mainly concerned with the current thinking and feeling of the testee."}, "sentence_type": "Interrogative sentence", "pos": {"nouns": {"person": 1, "Impression": 1}, "verbs": {"I'm worried": 1, "have": 1}, "numerals": {"How many": 1, "individual": 1}, "pronouns": {"to": 1}}}, {"content": "(15) Always try to impress themselves.", "word_count": 11, "label": "", "sentence_structure": "Subject鈥搗erb鈥揷omplement", "subjective_objective": {"answer": "Subjective", "rationale": "Because \"always try to make others appreciate\" is a form of subjective desire and behaviour."}, "sentence_sentiment": {"answer": "Positive", "rationale": "Because the behaviour and attitude of `best efforts to make others appreciate' in the entry is positive, indicating that the interviewees want to be recognized and commended by others."}, "language_style": {"answer": "Tactful and warm", "rationale": "The expression \"always try\" also gives a positive sense of direction, which is conducive to the active participation of respondents in the questionnaire."}, "time_dimension": {"answer": "Present", "rationale": "The entry can be judged by the time key word \"always\" in the sentence as describing the testee's habitual and customary behaviour or attitude, and thus the temporal dimension is now judged."}, "sentence_type": "Imperative sentence", "pos": {"verbs": {"Try": 1, "鈾?And I'm gonna be 鈾?: 1, "others": 1, "Admirable": 1}, "adverbs": {"Always": 1}}}, {"content": "(16) Concealed in the opposite sex.", "word_count": 6, "label": "", "sentence_structure": "Subject鈥搗erb鈥搊bject", "subjective_objective": {"answer": "Subjective", "rationale": "Because conceiting for opposite sex is an inner thought or emotional experience, it is a subjective examination."}, "sentence_sentiment": {"answer": "Negative", "rationale": "Because the description of the act of \"mixing yourselves with the opposite sex\" has a negative emotional connotation."}, "language_style": {"answer": "Tactful and warm", "rationale": "The entry is more personal and interactive by speaking in a gentle and inviting manner, rather than by direct statement or order. At the same time, the use of the term \"suspect\" avoids over-blank or offensive language and greater respect for the privacy of the interviewee."}, "time_dimension": {"answer": "Past", "rationale": ""}, "sentence_type": "Affirmative declarative sentence", "pos": {"verbs": {"I'm thinking about myself": 1}, "adjectives": {"Heterosexual": 1}}}, {"content": "(17) It is often avoided to express its feelings.", "word_count": 11, "label": "", "sentence_structure": "Subject鈥搗erb鈥揷omplement", "subjective_objective": {"answer": "Subjective", "rationale": "It is a subjective attitude and behaviour of the patient that is being examined whether the patient \"often avoids\" expressing feelings."}, "sentence_sentiment": {"answer": "Negative", "rationale": "Because the \"show your feelings often\" act is negative."}, "language_style": {"answer": "Calm and direct", "rationale": "The entry is more calm and straightforward, and the respondents are asked directly, through simple inquiries, whether they often avoid expressing their feelings, without excessive ado or emotional colours."}, "time_dimension": {"answer": "Present", "rationale": "The term \"expression\" and \"reception\" are both actions or states in progress, so it can be judged that the temporal dimension of the article is now."}, "sentence_type": "Imperative sentence", "pos": {"verbs": {"Often": 1, "Avoid": 1, "Expression": 1, "self": 1, "Feel": 1}, "adverbs": {"Regular": 1}, "numerals": {"7": 1}}}, {"content": "(18) Unconvinced self-image (physical appearance).", "word_count": 11, "label": "", "sentence_structure": "Subject鈥搗erb鈥揷omplement", "subjective_objective": {"answer": "Subjective", "rationale": "Because it is not sure that the instrument is the patient's subjective feeling and thoughts."}, "sentence_sentiment": {"answer": "Negative", "rationale": "Because the entry \"not sure about his instrument\" expresses a negative sentiment."}, "language_style": {"answer": "Calm and direct", "rationale": "The language style of the entry is relatively simple, with no excessive rhetorical or emotional overtook, and is presented mainly in the form of direct questions, which are more in line with the questionnaire. At the same time, the question is a straightforward expression of interest in and understanding of the perceived self-confidence of the interviewee, which facilitates subsequent data analysis and research."}, "time_dimension": {"answer": "Past", "rationale": ""}, "sentence_type": "Negative declarative sentence", "pos": {"nouns": {"Instrument": 1, "Looks like a": 1}, "verbs": {"Sure": 1, "No, I can't": 1}, "adverbs": {"Sure": 1}}}, {"content": "(19) Disgusting someone or being hated by someone.", "word_count": 11, "label": "", "sentence_structure": "Subject鈥損redicate", "subjective_objective": {"answer": "Subjective", "rationale": "Because it is a subjective emotion and feeling, not an objective fact, that is loathing or being hated."}, "sentence_sentiment": {"answer": "Negative", "rationale": "Because the \"hot someone\" act in the entry is negative."}, "language_style": {"answer": "Tactful and warm", "rationale": "The entry uses the word \"obsessed\" in a softer way, but does not directly ask the interviewee whether he or she is or is not hated by others, but rather, in this way, allows the interviewee to think about whether he or she has done so and express his or her feelings. This language style is more intimate, gentle, and more likely to trigger resonance and positive responses from the interviewee."}, "time_dimension": {"answer": "Past", "rationale": "The entry asks whether the person tested had had \"disturbed or hated someone\" in the past and could therefore be judged to have been past."}, "sentence_type": "Imperative sentence", "pos": {"nouns": {"person": 2}, "verbs": {"Shit": 2, "I hate it": 1}, "numerals": {"19": 1}}}, {"content": "(20) Discretionary.", "word_count": 5, "label": "", "sentence_structure": "Subject鈥搗erb鈥揷omplement", "subjective_objective": {"answer": "Subjective", "rationale": "Because the contempt for opposite sex is a subjective attitude or perception of the patient, not an objective fact. It depends on the patient鈥檚 personal experience and values and may be influenced by emotions and emotions."}, "sentence_sentiment": {"answer": "Negative", "rationale": "Because the attitude of \"defeating opposite sex\" in the entries is negative."}, "language_style": {"answer": "Calm and direct", "rationale": "The entry describes the problem in a straightforward language, with no excessive emotional accoutrements or euphemisms, and is more objective and neutral."}, "time_dimension": {"answer": "Present", "rationale": "The entry now asks about the current behaviour or attitude of the person being tested, the keyword being \"demeanor\", which is an emotional tendency or attitude and does not relate to past or future periods. The time dimension of the entry is therefore now."}, "sentence_type": "Affirmative declarative sentence", "pos": {"nouns": {"Heterosexual": 1}, "verbs": {"I'm not looking down": 1}}}, {"content": "(21) No attention can be paid to listening.", "word_count": 7, "label": "", "sentence_structure": "Subject鈥搗erb鈥揷omplement", "subjective_objective": {"answer": "Objective", "rationale": "Because listening is a factual description, not a subjective feeling or attitude of the patient. This is a capability that needs to be determined by observation and testing, and therefore it is objective."}, "sentence_sentiment": {"answer": "Negative", "rationale": "Because the \"not listening\" in the entry is negative."}, "language_style": {"answer": "Tactful and warm", "rationale": "The entry is more euphemistic, uses invitations to ask questions, and makes the interviewee feel more comfortable and comfortable in answering questions. At the same time, the entry does not accuse or criticize the interviewee, but asks him about his feelings and circumstances in a caring and understanding manner. This moderate expression helps the interviewee to open his heart and provide more real answers."}, "time_dimension": {"answer": "Present", "rationale": "The keyword \"can't listen with attention\" means the current difficulties of the testees in listening, so the entry is the present situation."}, "sentence_type": "Negative declarative sentence", "pos": {"nouns": {"Focus": 1, "Listen": 1}, "verbs": {"No, I can't": 1}}}, {"content": "(22) No one can complain about their own problems.", "word_count": 10, "label": "", "sentence_structure": "Subject鈥搗erb鈥揷omplement", "subjective_objective": {"answer": "Subjective", "rationale": "Because \"no one can complain\" is the patient's subjective judgment and feelings."}, "sentence_sentiment": {"answer": "Negative", "rationale": "Because \"no one can complain\" in the entry is an expression of a negative sentiment and agitation."}, "language_style": {"answer": "Tactful and warm", "rationale": "The language style of the entry is euphemistic and warm, resonating with the other side by using the expression \"no one can complain about his or her troubles\"."}, "time_dimension": {"answer": "Present", "rationale": ""}, "sentence_type": "Negative declarative sentence", "pos": {"nouns": {"self": 1, "Trouble": 1}, "verbs": {"The complaint": 1}, "numerals": {"22": 1}}}, {"content": "(23) Exclusion and indifference.", "word_count": 8, "label": "", "sentence_structure": "Subject鈥搗erb鈥揷omplement", "subjective_objective": {"answer": "Subjective", "rationale": "Because being excluded and apathy is a subjective feeling and experience of the patient."}, "sentence_sentiment": {"answer": "Negative", "rationale": "Because \"exclude and apathy\" in this entry is a negative emotional experience."}, "language_style": {"answer": "Calm and direct", "rationale": "The entry raises a specific problem in a directly explicit language, without using a rhetorical technique or emotional rendering, and with a more balanced and objective tone."}, "time_dimension": {"answer": "Present", "rationale": "The key words \"exclusion\" and \"exclusion and indifference\" in the previous entry indicate that this is a question of past experience or feelings and asks whether the subjects have experienced exclusion and indifference in the past."}, "sentence_type": "Negative declarative sentence", "pos": {"nouns": {"others": 1, "Exclusion": 1, "Coldness": 1}, "verbs": {"Yes": 1}, "adverbs": {"very": 1}, "numerals": {"23": 1}, "pronouns": {"EMPTY": 1}}}, {"content": "(24) Discretionary.", "word_count": 6, "label": "", "sentence_structure": "Subject鈥搗erb鈥揷omplement", "subjective_objective": {"answer": "Subjective", "rationale": "Because being \"regarded by the opposite sex\" is a subjective feeling or experience, a patient's perception and understanding of his or her own evaluation in the opposite eye, not an objective fact."}, "sentence_sentiment": {"answer": "Negative", "rationale": "Because the \"sightened\" act in the entry is negative."}, "language_style": {"answer": "Tactful and warm", "rationale": "The entry is presented in a euphemism that avoids direct questioning or accusing of the other. It is described in more objective and neutral language, and provides the interviewee with sufficient space to think and answer questions. Such language style helps to create a good atmosphere of communication and foster a greater willingness of the interviewee to share their feelings and experiences."}, "time_dimension": {"answer": "Past", "rationale": "At the same time, the issue did not refer explicitly to the present or future situation, but merely asked about past experience."}, "sentence_type": "Negative declarative sentence", "pos": {"nouns": {"Heterosexual": 1, "I'm not looking down": 1}, "verbs": {"By": 1}, "numerals": {"24": 1}}}, {"content": "(25) The views and opinions of the various parties cannot be widely heard.", "word_count": 13, "label": "", "sentence_structure": "Subject鈥搗erb鈥揷omplement", "subjective_objective": {"answer": "Subjective", "rationale": "Since the issue concerns the attitude and the perception of the patient, namely, whether he or she can be heard widely, the subjective perspective of the patient is examined."}, "sentence_sentiment": {"answer": "Negative", "rationale": "Because the \"unable to hear a wide range of opinions and opinions\" in the entry is negative."}, "language_style": {"answer": "Tactful and warm", "rationale": "The entry is more adept and interactive when it is spoken in a question-and-answer tone rather than directly speaking or ordering. At the same time, it uses euphemism, which is \"not broadly \" as opposed to \"not hearing all opinions and opinions\" and is more moderate in tone, less directly critical of each other's behaviour and more acceptable and willing to reflect on one's shortcomings."}, "time_dimension": {"answer": "Present", "rationale": "\"Unable to hear a wide range of opinions and opinions\" describes a capability or attitude, referring to the current situation. The time dimension of this entry is therefore the present."}, "sentence_type": "Negative declarative sentence", "pos": {"nouns": {"Observations": 2}, "verbs": {"Listen": 1}, "adjectives": {"Wide": 1, "No, I can't": 1}, "numerals": {"25": 1}}}, {"content": "(26) He is often in a state of deep self-inflicted grief as a result of his injuries.", "word_count": 12, "label": "", "sentence_structure": "Subject鈥搗erb鈥揷omplement", "subjective_objective": {"answer": "Subjective", "rationale": "Because \"smuggling\" is an emotional experience, a subjective feeling of the patient."}, "sentence_sentiment": {"answer": "Negative", "rationale": "Because the \"injured and heartbroken\" act in the entry is negative."}, "language_style": {"answer": "Tactful and warm", "rationale": "The entry is more euphemistic, using the expression \"self-intravented self-inflicted sadness\" instead of asking directly whether the other is injured or depressed, and instead directing the other to thinking about his/her inner feelings in a more subtle and caring way. At the same time, the use of the term \"simplified self-inflicted self-pity\" is more subtle, avoiding pressure or discomfort."}, "time_dimension": {"answer": "Past", "rationale": ""}, "sentence_type": "Affirmative declarative sentence", "pos": {"nouns": {"Hurt": 1, "Sad": 1}, "verbs": {"self": 1, "Regular": 1, "Because": 1, "Yes": 1, "And": 1, "From the shadows": 1}, "adverbs": {"Regular": 1, "From the shadows": 1}, "numerals": {"26": 1}, "pronouns": {"self": 1}}}, {"content": "(27) Often talked about and fooled.", "word_count": 8, "label": "", "sentence_structure": "Subject鈥搗erb鈥揷omplement", "subjective_objective": {"answer": "Subjective", "rationale": "Because being talked about and fooled by people is a subjective feeling and experience of patients and a subjective assessment of the state of mind."}, "sentence_sentiment": {"answer": "Negative", "rationale": "Because the \"screw, fool\" in the entry is negative."}, "language_style": {"answer": "Calm and direct", "rationale": "The language style of the entry is a cool, straightforward type. It presents a subject in a straightforward manner, without too much adoration and emotional rendering, and is more objective in tone.\""}, "time_dimension": {"answer": "Present", "rationale": "The temporal dimension of the entry is now, as it uses the verb \"sweet\" of the general current state of affairs, indicating that such behaviour is occurring regularly at present."}, "sentence_type": "Negative declarative sentence", "pos": {"nouns": {"person": 1, "Talk": 1, "Smuggle": 1}, "verbs": {"By": 1}, "numerals": {"27": 1}}}, {"content": "(28) Ignorance of how heterosexual relationships can be better managed.", "word_count": 14, "label": "", "sentence_structure": "Subject鈥搗erb鈥揷omplement", "subjective_objective": {"answer": "Subjective", "rationale": "Because the question is about the confusion and feelings of patients in their heterosexual relations, it is necessary to look at the patient from a subjective perspective."}, "sentence_sentiment": {"answer": "Negative", "rationale": "Because the \"I don't know how to get along\" in the entry is negative."}, "language_style": {"answer": "Tactful and warm", "rationale": "The entry is more euphemistic and personal, using the phrase \"I don't know how to get along better,\" avoiding a direct sense of criticism or blame, and providing respondents with a more comfortable and inclusive response environment. At the same time, the question is more open, encouraging respondents to share their ideas and experiences."}, "time_dimension": {"answer": "Present", "rationale": "The keyword \"to have sex with the opposite sex\" and \"to have a better relationship\" indicate that it is the trial's present obsession and doubt."}, "sentence_type": "Interrogative sentence", "pos": {"nouns": {"He's a heterosexual": 1, "Get along": 1}, "verbs": {"Yes": 1, "How's that": 1, "Better": 1, "Location": 1}, "adverbs": {"Land": 1}, "numerals": {"28": 1}}}]}

{"questionnaire_title": "Exchange of fear self-exemplary forms (PRCA-24)", "questionnaire_category": "Mental health", "item_count": 24, "emotion_shift_overall": "From negative to positive", "language_style_overall": "Neutral", "items": [{"content": "I do not like to participate in panel discussions;", "word_count": 10, "label": "", "sentence_structure": "Subject鈥損redicate", "subjective_objective": {"answer": "Subjective", "rationale": "Because \"not like\" is the patient's attitude or opinion towards participating in the panel discussion, it is subjective."}, "sentence_sentiment": {"answer": "Negative", "rationale": "Because \"not like\" in the entry is a negative emotional attitude."}, "language_style": {"answer": "Calm and direct", "rationale": "The language style of the entry is more of a cold and straightforward one, and it clearly expresses in negative terms the attitude of individuals who do not like to participate in panel discussions, without too many adorations or emotional expressions."}, "time_dimension": {"answer": "Present", "rationale": "The keywords \"not like\" and \"to participate in the panel discussion\" are both expressions of current attitudes and behaviour, so the entry describes the present feelings and behaviour of the testee, and the temporal dimension is now judged."}, "sentence_type": "Negative declarative sentence", "pos": {"nouns": {"Panel discussion": 1}, "verbs": {"Yeah": 1, "Participation": 1}, "adjectives": {"I don't like it": 1}}}, {"content": "2. I usually feel natural when participating in panel discussions;", "word_count": 15, "label": "", "sentence_structure": "Subject鈥揷opula鈥損redicative", "subjective_objective": {"answer": "Subjective", "rationale": "Because it is a subjective experience and feeling, it is the patient ' s own cognitive and emotional response."}, "sentence_sentiment": {"answer": "Positive", "rationale": "Because the expression \"felt natural\" in the entries is positive emotional, it is comfortable and confident to indicate that the process of participation in the panel discussions is a comfortable one."}, "language_style": {"answer": "Tactful and warm", "rationale": "The entry is more personal and interactive when it is spoken in a gentle and inviting tone, rather than in direct statements or orders. At the same time, the word \"normally\" also indicates that this is a relatively common phenomenon and does not overemphasize personal feelings or emotions."}, "time_dimension": {"answer": "Present", "rationale": "The entry describes the feelings of the person who was tested during the panel discussion, indicating that it is a regular, customary act or state, and therefore the temporal dimension is now judged."}, "sentence_type": "Affirmative declarative sentence", "pos": {"nouns": {"Panel": 1, "Discussion": 1}, "verbs": {"Participation": 1, "I feel": 1, "Naturally": 1}, "pronouns": {"I": 1}}}, {"content": "3. I was nervous to participate in the panel discussion;", "word_count": 15, "label": "", "sentence_structure": "Subject鈥搗erb鈥揷omplement", "subjective_objective": {"answer": "Subjective", "rationale": "Because it is a personal experience and reaction, it is a subjective observation."}, "sentence_sentiment": {"answer": "Negative", "rationale": "Because the \"frightful\" sentiment in the entry is negative."}, "language_style": {"answer": "Tactful and warm", "rationale": "The language style of the entry is more moderate, with no excessive negative emotional vocabulary or strong criticism, but rather a tone of encouragement and understanding that makes it easier for interviewees to open their hearts and share their feelings."}, "time_dimension": {"answer": "Present", "rationale": "The entry is based on the phrase \"at the panel discussion\" and \"attractive\" and the phrase \"at the time of the trial\" and the answer is \"now\"."}, "sentence_type": "Affirmative declarative sentence", "pos": {"nouns": {"Panel discussion": 1, "Nervous": 1, "Unsettled": 1}, "verbs": {"Participation": 1, "I feel": 1}, "numerals": {"3": 1}}}, {"content": "4. I would like to participate in the panel discussion;", "word_count": 9, "label": "", "sentence_structure": "Subject鈥搗erb鈥揷omplement", "subjective_objective": {"answer": "Subjective", "rationale": "Because it is the subjective attitude and feelings of patients who like or do not like to participate in the panel discussions."}, "sentence_sentiment": {"answer": "Positive", "rationale": "Because the behavior in the entries \"likes to participate in panel discussions\" is positive."}, "language_style": {"answer": "Tactful and warm", "rationale": "The entry is more adept and interactive when it is spoken in a gentle and inviting manner, rather than by direct statements or orders."}, "time_dimension": {"answer": "Present", "rationale": "The entry describes the current behaviour or attitude of the testee, which is \"like to participate\" as an expression of preference or preference, while \"panel discussion\" is an action of the present state of affairs, so it can be judged to be a description of the present conduct."}, "sentence_type": "Affirmative declarative sentence", "pos": {"verbs": {"Yeah": 1, "Participation": 1}, "adjectives": {"Panel": 1}, "adverbs": {"Discussion": 1}}}, {"content": "5. I was nervous to participate in the panel discussion with the living;", "word_count": 15, "label": "", "sentence_structure": "Subject鈥搗erb鈥揷omplement", "subjective_objective": {"answer": "Subjective", "rationale": "Because \"stretch\" is a subjective emotional experience, and is the feeling and reaction within the respondents, the issue is viewed from the patient's subjective perspective."}, "sentence_sentiment": {"answer": "Negative", "rationale": "Because the \"stretch\" in the entry is negative."}, "language_style": {"answer": "Calm and direct", "rationale": "The entry describes a phenomenon or feeling in a simple and clear language, without too many rhetorical or emotional colours, and is more calm and objective in tone.\""}, "time_dimension": {"answer": "Present", "rationale": "The entry describes the current feelings and experiences of the testees, in which the word \"make\" indicates that this feeling is due to participation in the panel discussions, and therefore the temporal dimension is now judged."}, "sentence_type": "Affirmative declarative sentence", "pos": {"nouns": {"Livers": 1, "Panel discussion": 1}, "verbs": {"鈾?And I'm gonna be 鈾?: 1, "Nervous": 1}, "adverbs": {"Together": 1}}}, {"content": "6. I was calm and relaxed in my participation in the panel discussion;", "word_count": 13, "label": "", "sentence_structure": "Subject鈥搗erb鈥揷omplement", "subjective_objective": {"answer": "Subjective", "rationale": "Because I'm here to speak for the patient, and the feeling of being in the group is the patient's subjective experience. So, this is a subjective perspective."}, "sentence_sentiment": {"answer": "Neutral", "rationale": "Because the entry describes an act without a clear emotional tendency."}, "language_style": {"answer": "Tactful and warm", "rationale": "The entry uses a more moderate and encouraging language style, which guides respondents to answer questions in the form of inquiries, and uses a positive word of \"silence and relaxation\" to give people a comfortable feeling of comfort. Such expression is more likely to make the interviewee feel respected and understood, and to get real answers."}, "time_dimension": {"answer": "Present", "rationale": "The entry \"at the panel table\" indicates the behaviour that is now being performed, and the description \"silence and relaxation\" is also a description of the current state, so it is possible to judge the time dimension of the article as it is."}, "sentence_type": "Affirmative declarative sentence", "pos": {"nouns": {"Panel discussion": 1, "Calm down": 1, "Relax": 1}, "verbs": {"Participation": 1, "be": 1}, "adjectives": {"I": 1}}}, {"content": "7. I am usually nervous about attending meetings;", "word_count": 13, "label": "", "sentence_structure": "Subject鈥揷opula鈥損redicative", "subjective_objective": {"answer": "Subjective", "rationale": "Because stress is a subjective feeling."}, "sentence_sentiment": {"answer": "Negative", "rationale": "Because the act of \"stretching\" in the entry is negative."}, "language_style": {"answer": "Calm and direct", "rationale": ""}, "time_dimension": {"answer": "Present", "rationale": ""}, "sentence_type": "Affirmative declarative sentence", "pos": {"nouns": {"Meetings": 1, "Nervous": 1}, "verbs": {"Participation": 1, "I feel": 1}, "adverbs": {"Usually": 1}, "numerals": {"7": 1}}}, {"content": "I usually feel calm and relaxed in attending meetings;", "word_count": 16, "label": "", "sentence_structure": "Subject鈥搗erb鈥揷omplement", "subjective_objective": {"answer": "Subjective", "rationale": "Because \"feeling calm and relaxing\" is a feeling and attitude within a person, subjective."}, "sentence_sentiment": {"answer": "Positive", "rationale": "Because the entry \"Feeling calm and relaxed\" expresses a positive sentiment."}, "language_style": {"answer": "Tactful and warm", "rationale": ""}, "time_dimension": {"answer": "Present", "rationale": ""}, "sentence_type": "Affirmative declarative sentence", "pos": {"nouns": {"Meetings": 1, "I": 1}, "verbs": {"- Yes, I'm here": 1, "I feel": 1, "Relax": 1}, "adjectives": {"Calm down": 1}}}, {"content": "9. When asked to present my views, I felt very calm and relaxed;", "word_count": 23, "label": "", "sentence_structure": "Subject鈥搗erb鈥揷omplement", "subjective_objective": {"answer": "Subjective", "rationale": "Because \"sense of great calm and relaxation\" is a personal feeling or emotional reaction, and it's the patient's subjective attitude."}, "sentence_sentiment": {"answer": "Positive", "rationale": "Because the entry \"Feeling very calm and relaxed\" shows a positive emotional attitude."}, "language_style": {"answer": "Tactful and warm", "rationale": ""}, "time_dimension": {"answer": "Present", "rationale": "\"When asked to present my views at this meeting\" is what happened in the past, and \"I feel very calm and relaxed\" is what is happening, and it is judged now."}, "sentence_type": "Affirmative declarative sentence", "pos": {"nouns": {"Yes": 1, "My point": 1}, "verbs": {"Requested": 1, "Statement": 1, "I feel": 1, "Relax": 1}, "adjectives": {"Very": 2, "Calm down": 1}, "adverbs": {"On the occasion of the Conference": 1}, "pronouns": {"I": 1}}}, {"content": "I was afraid to express my views at the meeting;", "word_count": 13, "label": "", "sentence_structure": "Subject鈥搗erb鈥揷omplement", "subjective_objective": {"answer": "Subjective", "rationale": "Because fear is a subjective emotional reaction, although it may be linked to the objective fact that a statement is made in a meeting."}, "sentence_sentiment": {"answer": "Negative", "rationale": "Because \"fear\" is a negative emotional vocabulary, indicating a negative sentiment about the conference environment and the expression of self-expression."}, "language_style": {"answer": "Calm and direct", "rationale": "The entry is more straightforward in language style, with no excessive speech or emotional rendering, and is more objective in tone."}, "time_dimension": {"answer": "Present", "rationale": "The entry uses the now completed tense \"refraid\" as an indication that this fear has been going on for some time, emphasizing what it feels now."}, "sentence_type": "Negative declarative sentence", "pos": {"nouns": {"Questionnaire": 1, "Entry": 1, "Yes": 1, "Observations": 1}, "verbs": {"Scared": 1, "Expression": 1}, "adverbs": {"self": 1}, "numerals": {"10": 1}, "pronouns": {"I": 1}}}, {"content": "11. Interaction at meetings often makes me unnatural;", "word_count": 12, "label": "", "sentence_structure": "Subject鈥搗erb鈥揷omplement", "subjective_objective": {"answer": "Subjective", "rationale": "Because \"unnatural\" is a subjective feeling and feeling, it is the patient's perception and experience of meeting and interacting."}, "sentence_sentiment": {"answer": "Negative", "rationale": "Because the description of \"unnatural\" in this entry is usually considered as negative emotions or experiences."}, "language_style": {"answer": "Calm and direct", "rationale": "The language style of the entry is more calm and straightforward, without too much emotional and rhetorical, but simply describes a phenomenon or feeling. This expression is more in line with the language requirements of the questionnaire, because it conveys the message clearly and objectively and avoids causing subjective feelings among the person under investigation."}, "time_dimension": {"answer": "Past", "rationale": "The entry describes the feelings and experiences of the testees during the meeting and does not address past or future behaviour or expectations, so the temporal dimension is now judged."}, "sentence_type": "Negative declarative sentence", "pos": {"nouns": {"Yes": 1, "- To be with someone": 1}, "verbs": {"鈾?And I'm gonna be 鈾?: 1, "It's not natural": 1}, "adjectives": {"Naturally": 1}, "adverbs": {"Regular": 1}, "numerals": {"11": 1}, "classifiers": {"individual": 0}, "pronouns": {"I": 1}}}, {"content": "12. I was very relaxed in answering the inter-questions at the meeting;", "word_count": 14, "label": "", "sentence_structure": "Subject鈥搗erb鈥揷omplement", "subjective_objective": {"answer": "Subjective", "rationale": "Because \"relax\" is a subjective feeling and attitude, not an objective fact or behaviour. This entry looks at the feelings and emotional state of patients who answer questions in a meeting, and it is therefore a subjective one."}, "sentence_sentiment": {"answer": "Positive", "rationale": "Because the description of \"very relaxed\" in the entry expresses positive attitudes and emotions."}, "language_style": {"answer": "Calm and direct", "rationale": "The entry directly expresses the psychological state of the person who asked the other person to answer the question during the meeting, without using excessive rhetorical or emotional rendering, and is more objective in tone. The language style of the entry is therefore \"cool and direct\"."}, "time_dimension": {"answer": "Present", "rationale": "Now the entry describes the situation of the person who was tested in the meeting in answer to the question, the keywords being \"at the meeting\" and \"very relaxed\", which point to the current, ongoing behaviour or state. Thus, it can be judged to be the present."}, "sentence_type": "Affirmative declarative sentence", "pos": {"nouns": {"Yes": 1, "Problem": 1, "Inter-problem": 1}, "verbs": {"Answer": 1, "Go, go, go": 1}, "adjectives": {"Relax": 1, "Very": 1}, "adverbs": {"Land": 1}, "numerals": {"12": 1}}}, {"content": "I was very disturbed when I spoke to new people;", "word_count": 16, "label": "", "sentence_structure": "Subject鈥揷opula鈥損redicative", "subjective_objective": {"answer": "Subjective", "rationale": "Because feeling very uneasy is a subjective feeling."}, "sentence_sentiment": {"answer": "Negative", "rationale": "Because the attitude of \"very uneasy\" in the entry is negative."}, "language_style": {"answer": "Calm and direct", "rationale": "By asking directly whether the interviewees were disturbed when talking to new people, the entry \"Cool and Direct\" avoided excessive emotional rendering and rhetorical tactics, with a relatively calm and straightforward language style."}, "time_dimension": {"answer": "Present", "rationale": ""}, "sentence_type": "Negative declarative sentence", "pos": {"nouns": {"person": 1, "Talk": 1}, "verbs": {"Same": 1, "Yes": 1, "I feel": 1, "Very": 1, "Unsettled": 1}, "adverbs": {"Very": 1}, "numerals": {"13": 1}}}, {"content": "14. I was not afraid when I spoke in conversation;", "word_count": 13, "label": "", "sentence_structure": "Subject鈥搗erb鈥揷omplement", "subjective_objective": {"answer": "Subjective", "rationale": "Because \"I\" represents the patient's subjective perspective."}, "sentence_sentiment": {"answer": "Positive", "rationale": "Because \"no fear when speaking\" is a positive attitude and quality, which means that a person is confident and good at expressing his own thoughts."}, "language_style": {"answer": "Calm and direct", "rationale": "The phrase is more direct in language, with no excessive rhetoric or emotional colour. It is simple and clear in its interview with the person under investigation, and it is more objective and calm in tone."}, "time_dimension": {"answer": "Present", "rationale": ""}, "sentence_type": "Affirmative declarative sentence", "pos": {"verbs": {"Say it": 1, "Scared": 1}, "adjectives": {"A little": 1}, "adverbs": {"not": 1}, "numerals": {"14": 1}}}, {"content": "15. I'm usually very nervous in my conversations.", "word_count": 13, "label": "", "sentence_structure": "Subject鈥搗erb鈥揷omplement", "subjective_objective": {"answer": "Subjective", "rationale": "This entry examines patients ' subjective feelings and experiences, i.e. the sense of tension in conversations."}, "sentence_sentiment": {"answer": "Negative", "rationale": "Because the \"frightful\" in the entry is an expression of a negative emotion."}, "language_style": {"answer": "Tactful and warm", "rationale": "The language style of the entry is more euphemistic, expresses emotional intensity by using the word \"very\" and uses the tone of the inquiry rather than direct statements or orders, which makes it easier to direct respondents to share their real feelings and experiences. Moreover, the term \"interview\" makes the issue more specific and understandable, and facilitates access to more accurate information."}, "time_dimension": {"answer": "Present", "rationale": "The words \"normally\" and \"in conversation\" make it possible to judge that the act is a customary situation that is taking place now."}, "sentence_type": "Negative declarative sentence", "pos": {"nouns": {"Talk": 1, "Nervous": 1, "Unsettled": 1}, "verbs": {"in": 1, "be": 1, "Talk": 1, "Nervous": 1, "Unsettled": 1}, "adverbs": {"Very": 1}, "numerals": {"15": 1}}}, {"content": "16. I have usually been very calm and relaxed in my conversations;", "word_count": 14, "label": "", "sentence_structure": "Subject鈥搗erb鈥揷omplement", "subjective_objective": {"answer": "Subjective", "rationale": "Because calm and relaxation are patients' subjective feelings and attitudes."}, "sentence_sentiment": {"answer": "Positive", "rationale": "Because the entry \"quiet and relax\" expresses positive emotional attitudes and behavioural characteristics."}, "language_style": {"answer": "Calm and direct", "rationale": "The subject is a straightforward question about the emotional state of the person under investigation during the communication process, without any rhetorical or emotional accent, and is more objective. Thus, the language style of the article is considered to be straightforward and calm."}, "time_dimension": {"answer": "Present", "rationale": ""}, "sentence_type": "Affirmative declarative sentence", "pos": {"nouns": {"Talk": 1, "Calm down": 1, "Relax": 1}, "verbs": {"in": 1, "be": 1, "Talk": 1}, "adjectives": {"Very": 1, "Calm down": 1, "Relax": 1}, "numerals": {"16": 1}}}, {"content": "17. I feel relaxed when talking to new people;", "word_count": 17, "label": "", "sentence_structure": "Subject鈥揷opula鈥損redicative", "subjective_objective": {"answer": "Subjective", "rationale": "Because \"feeling relaxed\" is the patient's subjective feeling and experience."}, "sentence_sentiment": {"answer": "Positive", "rationale": "Because the entry \"sweetly relaxed\" expresses a positive emotional attitude."}, "language_style": {"answer": "Tactful and warm", "rationale": "Warm and warm."}, "time_dimension": {"answer": "Present", "rationale": "The entry describes the current behaviour and feelings of the subject, \"when talking to a new person\" indicating that the act is being done, and \"I feel very relaxed\" describes the current feeling. So the temporal dimension of the article is now judged."}, "sentence_type": "Affirmative declarative sentence", "pos": {"nouns": {"Questionnaire Entry": 1, "person": 1, "Talk": 1, "Relax": 1}, "verbs": {"I feel": 1, "Tens": 1, "be": 1}, "adverbs": {"in": 1, "Same": 1}, "numerals": {"17": 1, "Tens": 1}, "classifiers": {"individual": 1}, "pronouns": {"I": 1}}}, {"content": "18. I was afraid to speak in my conversation;", "word_count": 9, "label": "", "sentence_structure": "Subject鈥揷opula鈥損redicative", "subjective_objective": {"answer": "Subjective", "rationale": "Fear of speaking is a subjective feeling and experience, not an objective fact or event."}, "sentence_sentiment": {"answer": "Negative", "rationale": "Because the \"fear to speak\" attitude in the entry is negative."}, "language_style": {"answer": "Calm and direct", "rationale": "The language style of the entry is calm and direct, and it asks the interviewee in a clear and direct manner, without too much ado or emotional expression."}, "time_dimension": {"answer": "Present", "rationale": "\"I'm afraid to talk\" means the state of mind and behavior of the moment, so the basis of the judgment is \"now\"."}, "sentence_type": "Negative declarative sentence", "pos": {"nouns": {"Talk": 2}, "verbs": {"Scared": 1, "Talk": 1}, "numerals": {"18": 1}}}, {"content": "I'm not afraid of speeches at all;", "word_count": 10, "label": "", "sentence_structure": "Subject鈥搗erb鈥揷omplement", "subjective_objective": {"answer": "Subjective", "rationale": "Because \"never afraid\" is the patient's subjective idea or attitude."}, "sentence_sentiment": {"answer": "Neutral", "rationale": "Since the entry merely expresses the individual ' s attitude towards the speech, it does not relate to specific acts or outcomes and therefore has no obvious emotional colours."}, "language_style": {"answer": "Tactful and warm", "rationale": "The language style of the entry is more euphemistic and moderate, not using the negative or positive language directly, but rather directing the respondents to answer the questions by presenting their views."}, "time_dimension": {"answer": "Present", "rationale": "In this sentence, \"I\" and \"no fear\" indicate that the act or state is now being performed. Moreover, \"not at all\" further highlights the extent of this feeling, implying that it is a very positive answer. So I think the time in this questionnaire entry is described as \"now\"."}, "sentence_type": "Affirmative declarative sentence", "pos": {"verbs": {"to": 1, "Scared": 1}, "adjectives": {"A little": 1, "not": 1, "Scared": 1}, "adverbs": {"very": 1}, "numerals": {"19": 1}, "pronouns": {"I": 1}}}, {"content": "20. When I spoke, certain parts of the body were very tense and rigid;", "word_count": 19, "label": "", "sentence_structure": "Subject鈥揷opula鈥損redicative", "subjective_objective": {"answer": "Subjective", "rationale": "Because \"heavy\" and \"silent\" are the patient's own subjective experience and emotional reaction to the speech."}, "sentence_sentiment": {"answer": "Negative", "rationale": "Because \"some parts of the body are very nervous and rigid\" in the entry is a negative emotional expression."}, "language_style": {"answer": "Calm and direct", "rationale": "The language style of the entry is straightforward, as it directly expresses the researcher ' s concern about the stress and rigidity of certain parts of the body during the interview and is speaking in a balanced and objective manner."}, "time_dimension": {"answer": "Present", "rationale": ""}, "sentence_type": "Affirmative declarative sentence", "pos": {"nouns": {"Speech": 1, "Body": 1, "Part": 1, "Nervous": 1, "Stunning": 1}, "verbs": {"Speech": 1, "Body": 1, "I feel": 1, "Very": 1}, "adjectives": {"Nervous": 1, "Stunning": 1}, "adverbs": {"Very": 1}, "classifiers": {"Part": 1}}}, {"content": "21. I was relaxed in my speech;", "word_count": 9, "label": "", "sentence_structure": "Subject鈥揷opula鈥損redicative", "subjective_objective": {"answer": "Subjective", "rationale": "Because feeling relaxed is a subjective feeling for a person."}, "sentence_sentiment": {"answer": "Positive", "rationale": "Because the \"Easy to Feel Relaxed\" act in the entry is positive."}, "language_style": {"answer": "Tactful and warm", "rationale": "The entry uses a soft and warm tone, which leads the respondents to share their views about whether they feel relaxed when speaking, rather than directly judging or expressing their views."}, "time_dimension": {"answer": "Present", "rationale": ""}, "sentence_type": "Affirmative declarative sentence", "pos": {"nouns": {"I": 1, "Speech": 1, "Relax": 1}, "verbs": {"in": 1, "I feel": 1}, "adverbs": {"Time": 1}}}, {"content": "22. I have become confused and incoherent in my speeches;", "word_count": 15, "label": "", "sentence_structure": "Subject鈥揷opula鈥損redicative", "subjective_objective": {"answer": "Subjective", "rationale": "Because \"the confusion and incoherence of thinking\" is the patient's assessment and perception of his or her own performance, and it's a subjective feeling."}, "sentence_sentiment": {"answer": "Negative", "rationale": "Because the description \"disturbed and incoherent\" is negative, indicating that there are problems in speaking."}, "language_style": {"answer": "Calm and direct", "rationale": "The language style of the entry is straightforward and calm, as it directly describes the problems that the person under investigation may encounter in his or her speech, and does not use any qualifying term or expression to mitigate or emphasize the importance of the issue."}, "time_dimension": {"answer": "Present", "rationale": "The entry is for the interview of the person who was the subject of the speech, and the time dimension is now judged."}, "sentence_type": "Negative declarative sentence", "pos": {"nouns": {"Speech": 1, "Thinking": 1, "Chaos": 1, "Coherence": 1}, "verbs": {"鈾?And become 鈾?: 1}, "adjectives": {"It's messy": 1, "Incoherent": 1}, "numerals": {"22": 1}, "pronouns": {"I": 1}}}, {"content": "I look forward to the confidence with which I speak;", "word_count": 12, "label": "", "sentence_structure": "Subject鈥搗erb鈥揷omplement", "subjective_objective": {"answer": "Subjective", "rationale": "Because \"expectation\" is a subjective attitude or emotion."}, "sentence_sentiment": {"answer": "Positive", "rationale": "Because the expectation of confidence in the speech is a positive attitude and behaviour, a demonstration of confidence in itself and a desire for better performance."}, "language_style": {"answer": "Tactful and warm", "rationale": "The language style of the entry is more euphemistic, making the issue more moderate, friendly and constructive by using the words \"suspect\" and \"confident.\" This is more able to draw the attention of the respondents and encourage them to answer questions in a true and honest manner."}, "time_dimension": {"answer": "Present", "rationale": "The entry contains visions and expectations for the future, and the word \"expectation\" expresses the testee's expectations about a future, so the temporal dimension is judged to be the future. And \"confident in speaking\" also refers to behaviour or outcome towards the future."}, "sentence_type": "Affirmative declarative sentence", "pos": {"nouns": {"Speeches": 1, "Confidence": 1}, "verbs": {"Expectations": 1, "In": 1, "Fill": 1}, "adverbs": {"Total": 1}, "numerals": {"23": 1}, "pronouns": {"I": 1}}}, {"content": "I was so nervous when I was speaking that I forgot everything I really knew.", "word_count": 23, "label": "", "sentence_structure": "Subject鈥搗erb鈥揷omplement", "subjective_objective": {"answer": "Subjective", "rationale": "Because \"stretch\" and \"forget\" are subjective feelings and behaviours of patients."}, "sentence_sentiment": {"answer": "Negative", "rationale": "Because \"too nervous\" and \"forget what I do know\" are negative emotions, attitudes or behaviours."}, "language_style": {"answer": "Tactful and warm", "rationale": "The issue is presented in a way that is not ordered, giving a sense of moderation; 2. The problem is not one in which strong emotional terms or rhetoric are used, but rather in which the facts are presented directly and in a more objective manner; 3. The problem is one in which the story and feelings of the speaker are depicted in the words of \"too tense\" and \"forget\" and show the interest and understanding of the interviewee."}, "time_dimension": {"answer": "Past", "rationale": "The entry describes the past experience and feelings, \"too nervous in speech\" and \"forgetting everything I really know\" are all descriptions of what has happened. So, the temporal dimension is the past."}, "sentence_type": "Negative declarative sentence", "pos": {"nouns": {"Speech": 1, "Nervous": 1, "Things": 1}, "verbs": {"in": 1, "Forget it": 1, "Yes": 1, "I was nervous when I was speaking": 1, "I forgot everything I really knew": 1}, "adjectives": {"Too much": 1, "of": 2, "Indeed": 1}, "adverbs": {"in": 1, "Time": 1, "Too much": 1}, "classifiers": {"individual": 1}, "pronouns": {"I": 1}}}]}

{"questionnaire_title": "Table of selected interpersonal trust", "questionnaire_category": "Mental health", "item_count": 32, "emotion_shift_overall": "From negative to positive", "language_style_overall": "Tactful and warm", "items": [{"content": "If I am complimented, I will ask him whether he is sincere in his praise of me.", "word_count": 20, "label": "", "sentence_structure": "Subject鈥搗erb鈥搊bject", "subjective_objective": {"answer": "Subjective", "rationale": "This is because of the self-perception and attitude of the patient when considering others ' evaluation."}, "sentence_sentiment": {"answer": "Positive", "rationale": "Because the purpose of the entry is to \"show praise from the heart.\""}, "language_style": {"answer": "Tactful and warm", "rationale": "The language style of the questionnaire entry is euphemism. It uses a gentle and inviting tone of speech to engage in dialogue, rather than a direct statement or order, which is more personal and interactive. The entry seeks to understand the views and attitudes of the interviewees about others and to guide them to reflection and answers by asking a question."}, "time_dimension": {"answer": "Future", "rationale": "The entry contains visions and expectations for the future, and the keyword is \"if\" and it means that a hypothetical scenario may occur in the future. And \"Question\" also indicates that this is an act that has not yet occurred and will occur. So the answer is the future."}, "sentence_type": "Interrogative sentence", "pos": {"nouns": {"- Congratulations": 2, "Heart": 1}, "verbs": {"Question": 1, "From": 1, "be": 1}, "numerals": {"One": 2}, "pronouns": {"I": 1}}}, {"content": "2. If we have an appointment to have lunch somewhere, I'm sure _I'll be there.", "word_count": 20, "label": "", "sentence_structure": "Subject鈥搗erb鈥搊bject", "subjective_objective": {"answer": "Subjective", "rationale": "Because the expression in the questionnaire's purpose contains the word \"I am sure\" and indicates that it is the patient's subjective attitude."}, "sentence_sentiment": {"answer": "Positive", "rationale": "Because the \"sure to be there\" in the entry is a positive attitude and behavior."}, "language_style": {"answer": "Tactful and warm", "rationale": "The language style of the entry tends to be euphemistic, because it uses words such as \"yes\" and \"yes\" to express commitment and confidence, which give people a sense of kindness and trust. At the same time, it is more gentle, friendly, not strong or commanding, and therefore more in line with the euphemism of language."}, "time_dimension": {"answer": "Present", "rationale": "Therefore, this entry should be classified as now."}, "sentence_type": "Affirmative declarative sentence", "pos": {"verbs": {"we": 1, "- A date": 1, "Lunch": 1, "I": 1, "The Council": 1, "Here we go": 1}, "adverbs": {"Sure": 1}, "pronouns": {"Somewhere": 1}}}, {"content": "I will travel with _ on foot in a strange place, if _ is certain that he or she is familiar with the area.", "word_count": 27, "label": "", "sentence_structure": "Subject鈥搗erb鈥搊bject", "subjective_objective": {"answer": "Subjective", "rationale": "Because it is a subjective sentence, it involves the attitude and choice of the individual."}, "sentence_sentiment": {"answer": "Neutral", "rationale": "Basis of judgement: The entry is not clearly expressed in a positive, negative or emotional manner, but rather describes a scene and conditions and can therefore be considered neutral."}, "language_style": {"answer": "Tactful and warm", "rationale": "The language style of the entry is euphemistic, firstly raising questions in an invitation-style manner, making the respondents comfortable and comfortable, and also making them more open and flexible by using the conditional phrase \"if _respect of his or her familiarity with the area\", which allows them to better understand the actual needs and expectations of the respondents."}, "time_dimension": {"answer": "Present", "rationale": "And the whole sentence is a normal future, which means that the act has not yet happened, but the tester is willing to do it. So the time dimension is now."}, "sentence_type": "Interrogative sentence", "pos": {"nouns": {"Local": 1, "Walking": 1, "If": 1, "he": 1, "she": 1, "Region": 1}, "verbs": {"Yes": 1, "and": 1, "Sure": 1}, "adverbs": {"Strange": 1, "The": 1}, "numerals": {"3": 1}, "pronouns": {"If": 1, "he": 1, "she": 1}}}, {"content": "I will not buy any old furniture from 鈥?because I do not believe in his/her valuation.", "word_count": 25, "label": "", "sentence_structure": "Subject鈥搗erb鈥搊bject", "subjective_objective": {"answer": "Objective", "rationale": "Because this is the patient's opinion and opinion of \"he/her\", which is subjective; however, the idea is based on an objective fact (purchase of old furniture), and the entry is therefore more objective."}, "sentence_sentiment": {"answer": "Negative", "rationale": "Because it contains mistrust and suspicion of others, it is a negative emotional attitude."}, "language_style": {"answer": "Calm and direct", "rationale": "The entry is more calm and straightforward in its language, with no excessive rhetorical and emotional rendering. It directly expresses the subject of asking the other side whether to buy old furniture in some places and why, and emphasizes the sense of mistrust in the negative, with a clearer and objective tone.\""}, "time_dimension": {"answer": "Present", "rationale": "The keyword \"no\" and \"no\" and \"his/her valuation\" indicate that the attitude and behaviour of the testees towards the purchase of second-hand furniture are now present."}, "sentence_type": "Negative declarative sentence", "pos": {"nouns": {"Furniture": 1, "Valuation": 1}, "verbs": {"Buy": 1, "Yes": 1, "There": 1}, "adjectives": {"Old": 1}, "numerals": {"One": 1, "Four": 1}, "classifiers": {"Annex": 1}, "pronouns": {"I": 2, "He/she": 1}}}, {"content": "I believe that _ will be impartial.", "word_count": 8, "label": "", "sentence_structure": "Subject鈥搗erb鈥揷omplement", "subjective_objective": {"answer": "Subjective", "rationale": "Because the word \"convinced\" reflects the patient's views, beliefs or attitudes to \"fairness\" and is a highly subjective expression of language."}, "sentence_sentiment": {"answer": "Positive", "rationale": "Because it expresses a trust and a positive expectation."}, "language_style": {"answer": "Tactful and warm", "rationale": "The entry, which was dialogueed in a moderate and positive tone, emphasized the belief in justice and did not use too strong or direct language expressions, which were more personal and interactive."}, "time_dimension": {"answer": "Past", "rationale": "The entry describes the degree of confidence that the person tested has in a person or institution, which is based on the conclusions of past experience and observations and is therefore judged to be past."}, "sentence_type": "Interrogative sentence", "pos": {"nouns": {"Yes": 2}, "verbs": {"OPERATIONS": 1}, "adjectives": {"Justice": 1}, "classifiers": {"Yes": 1}}}, {"content": "If I cannot go to the post office, I can rest assured that _ will send an important letter to me.", "word_count": 24, "label": "", "sentence_structure": "Subject鈥搗erb鈥搊bject", "subjective_objective": {"answer": "Subjective", "rationale": "Since the questionnaire entries were asked about the patient ' s will and sense of trust, their subjective attitude was examined."}, "sentence_sentiment": {"answer": "Positive", "rationale": "It is positive because it shows the tendency of respondents to trust others in their ability to handle important matters and to be willing to entrust others."}, "language_style": {"answer": "Tactful and warm", "rationale": "The language style of the entry is more euphemistic, using the second-person proxy \"you,\" making it easier for the person questioned to resonate and feel a sense of identity. At the same time, the expression is more polite, polite, and gives a friendly and intimate impression. Moreover, the issue is not clearly defined by the specific person or role, but rather leaves the space for the person questioned to choose, further reflecting the warmness of the style."}, "time_dimension": {"answer": "Present", "rationale": "The questionnaire entry now asks the testee whether he or she is willing to commission important correspondence in person, which usually needs to be done in a timely manner, and therefore the time dimension is now judged."}, "sentence_type": "Interrogative sentence", "pos": {"nouns": {"Post Office": 1, "Letter": 1}, "verbs": {"Let's go": 1}, "classifiers": {"One": 1}, "pronouns": {"I": 2}}}, {"content": "I can tell the secret to _, and I know he/she wanted to hear it.", "word_count": 18, "label": "", "sentence_structure": "Subject鈥搗erb鈥搊bject", "subjective_objective": {"answer": "Subjective", "rationale": "The patient ' s subjective attitude and conviction, which he believes is the person ' s willingness to listen to his secrets, are examined."}, "sentence_sentiment": {"answer": "Positive", "rationale": "Because the \"willing to listen\" act in the entry is positive and represents the ability to build trust and good relations."}, "language_style": {"answer": "Tactful and warm", "rationale": ""}, "time_dimension": {"answer": "Present", "rationale": "According to the sentence entered, the descriptions of \"I can reveal secrets to _\" and \"I know he/she wants to hear\" are in the current behavior or state, so the time dimension of this questionnaire entry should be now."}, "sentence_type": "Interrogative sentence", "pos": {"nouns": {"Secrets": 1, "he": 1, "person": 1}, "verbs": {"Show": 1, "The original intent": 1, "Listen": 1}, "adverbs": {"Yes": 1, "And": 1}, "numerals": {"7": 1}, "pronouns": {"I": 1, "he": 1}}}, {"content": "I am sure that _ will tell me the truth.", "word_count": 11, "label": "", "sentence_structure": "Subject鈥搗erb鈥搊bject", "subjective_objective": {"answer": "Subjective", "rationale": "Because \"convincing\" is a personal conviction and attitude, a subjective approach."}, "sentence_sentiment": {"answer": "Neutral", "rationale": "Because the entry does not have a clear emotional and evaluative tone, it is merely asking the interviewees whether they believe someone will tell the truth."}, "language_style": {"answer": "Tactful and warm", "rationale": "The entry uses an invitation tone, not a direct statement or order, expressing respect for and expectation of the real ideas of the interviewee. At the same time, the expression \"I believe\" implies trust and understanding of the interviewee, making the questionnaire more personal and interactive. So I think the language style of the questionnaire entry is euphemistic."}, "time_dimension": {"answer": "Present", "rationale": "Now the word \"I\" in the entry indicates that the person who was tested is expressing his conviction, while \"Believe\" indicates that belief is current, present. So the temporal dimension of the article is now."}, "sentence_type": "Interrogative sentence", "pos": {"nouns": {"I": 1, "The truth": 1}, "verbs": {"Yes": 1, "Tell": 1}}}, {"content": "If I'm going to catch a plane, I'm not sure if I can be taken to the airport on time.", "word_count": 23, "label": "", "sentence_structure": "Subject鈥搗erb鈥搊bject", "subjective_objective": {"answer": "Subjective", "rationale": "Because it describes the patient's subjective uncertainty and concern."}, "sentence_sentiment": {"answer": "Negative", "rationale": "Because the expression \"unconvinced\" in the entry is indicative of uncertainty and disquiet and negative emotional colours."}, "language_style": {"answer": "Calm and direct", "rationale": "This is a language that is simple and clear, which is a direct statement of the problem, and does not add too many verbs or emotional colours."}, "time_dimension": {"answer": "Future", "rationale": "The entry contains concerns and uncertainties about what might happen in the future, namely, \"can I be brought to the airport on time\", which suggests that it points to the future."}, "sentence_type": "Interrogative sentence", "pos": {"verbs": {"Yes": 1, "Hurry up": 1, "Aircraft": 1, "No, I'm not": 1, "Hold on": 1, "Will": 1}, "adjectives": {"On time": 1}, "adverbs": {"Is it possible": 1}, "pronouns": {"I": 1}}}, {"content": "If I do something or say something, and I laugh unexpectedly, I wonder if he/she blames me or does not mean anything.", "word_count": 41, "label": "", "sentence_structure": "Subject鈥搗erb鈥搊bject", "subjective_objective": {"answer": "Subjective", "rationale": "Because the suspicion is the patient's own subjective idea, not the objective fact."}, "sentence_sentiment": {"answer": "Negative", "rationale": "Because the expression \"suspect him/her or not to me\" in this article is a negative emotion and attitude."}, "language_style": {"answer": "Tactful and warm", "rationale": "The language style of the entry is euphemism. It uses a gentle, invitation-style tone, which expresses concern and respect for the person under investigation in a way that is caring and understanding. It is not a direct accusation or an order, but rather a question that leads the person under investigation to think and share their thoughts and feelings, thus creating a good interaction."}, "time_dimension": {"answer": "Present", "rationale": "In the entry, words like \"do,\" \"do\" and \"can doubt\" indicate that this is a description and a sense of the current situation. So the basis of the judgment is now."}, "sentence_type": "Interrogative sentence", "pos": {"nouns": {"Yes": 1, "Word": 1, "It's unexpected": 1, "Smile": 2, "he": 1, "she": 1, "Blame": 1, "I don't mean to be": 1}, "verbs": {"Do it": 1, "Say it": 1, "Doubt": 1}, "adjectives": {"It's unexpected": 1}, "adverbs": {"If": 1}, "numerals": {"10": 1}, "pronouns": {"I": 2}}}, {"content": "I can talk to _ at will, and I know _ I love to listen.", "word_count": 15, "label": "", "sentence_structure": "Subject鈥搗erb鈥搊bject", "subjective_objective": {"answer": "Subjective", "rationale": "The title contains the term \"I know\" as subjective judgement, which examines the patient's knowledge and perception of others and therefore the subjective perspective. At the same time, the expression \"I can talk to _ and know _ love\" is based on the patient's subjective perception and feelings, and therefore the entry is subjective."}, "sentence_sentiment": {"answer": "Positive", "rationale": "Because the words \"discreet\" and \"know what he/she likes to hear\" in the entries are both positive attitudes and emotional expressions."}, "language_style": {"answer": "Tactful and warm", "rationale": "The language style of the entry is euphemistic, and the problem is expressed by using words such as \"can\" and \"know\" rather than direct command or questioning. At the same time, the interaction between \"me\" and \"_\" is more natural. The questionnaire entry can therefore be classified as a soft language style."}, "time_dimension": {"answer": "Present", "rationale": ""}, "sentence_type": "Interrogative sentence", "pos": {"nouns": {"I": 1, "Talk": 1, "Yes": 1, "I love it": 1}, "verbs": {"Yeah": 1, "Whatever": 1}, "adverbs": {"And": 1}, "numerals": {"11": 1}, "pronouns": {"锛?: 2}}}, {"content": "12. _ in no way deliberately misrepresented my views to others.", "word_count": 18, "label": "", "sentence_structure": "Subject鈥搗erb鈥揷omplement", "subjective_objective": {"answer": "Subjective", "rationale": "Because \"never willful\" is an individual's attitude or conviction, it reflects the subjective perception and expectations of the respondent's own conduct and is a subjective one."}, "sentence_sentiment": {"answer": "Positive", "rationale": "Because it's positive that the actions in the entries \"never deliberately misrepresent my views.\""}, "language_style": {"answer": "Calm and direct", "rationale": "The entry uses a direct language style and clearly expresses the subject matter, without excessive embellishment or emotional rendering. This language style is usually considered more objective and professional.\""}, "time_dimension": {"answer": "Present", "rationale": "So the time dimension is now judged."}, "sentence_type": "Negative declarative sentence", "pos": {"nouns": {"Never": 1, "On purpose": 1, "Mine": 1, "Views": 1, "others": 1}, "verbs": {"Will": 1, "Transmission": 1}, "adjectives": {"It's a distortion": 1}, "adverbs": {"Never": 1, "On purpose": 1, "Land": 1}, "numerals": {"12": 1}, "pronouns": {"Mine": 1}}}, {"content": "If _knows what hurts my feelings, I will never fear that he/she will use these things against me ... (even if he/she does not do so after our relationship has changed).", "word_count": 55, "label": "", "sentence_structure": "Subject鈥搗erb鈥搊bject", "subjective_objective": {"answer": "Subjective", "rationale": "This is based on the patient鈥檚 inner feelings and experience and does not involve objective facts or results."}, "sentence_sentiment": {"answer": "Neutral", "rationale": "The purpose of the article is expressed in a non-manifest emotional manner, but only in a hypothetical context."}, "language_style": {"answer": "Tactful and warm", "rationale": "The language style of the entry is more euphemistic and warm. It implies an atmosphere of trust and understanding by using the words \"if\" and \"no worries\". It also does not directly state or order what the interviewee should think or do, but rather engages in dialogue in a gentle, inviting tone, more personal and interactive manner."}, "time_dimension": {"answer": "Present", "rationale": "It can be inferred from the phrase \"if\" and \"without fear\" that this is a hypothetical and attitudinal description of a situation in the present context, and therefore the present is judged."}, "sentence_type": "Affirmative declarative sentence", "pos": {"nouns": {"Things": 2, "Emotion": 1, "Relations": 1}, "verbs": {"Roger that": 1, "I'm worried": 1, "Come on": 1, "Yes": 2, "Utilization": 1, "Change": 1, "Do it": 1}, "adjectives": {"It hurts": 1, "Never": 1}, "adverbs": {"If": 1, "Even if": 1}, "numerals": {"13": 1, "2": 1}, "pronouns": {"he": 2, "she": 2, "I": 2}}}, {"content": "If - and promise to help me, he/she will help me.", "word_count": 15, "label": "", "sentence_structure": "Subject鈥搗erb鈥搊bject", "subjective_objective": {"answer": "Subjective", "rationale": "Because this statement relates to the patient ' s personal convictions and expectations, it is subjective."}, "sentence_sentiment": {"answer": "Positive", "rationale": "The \"must help me\" in the entry is a positive expression of confidence and certainty."}, "language_style": {"answer": "Tactful and warm", "rationale": "The entry uses an indirect and euphemistic approach to express a request or expectation, without direct orders or threats. By using the conditional phrase \"if there is always\" the respondent has greater discretion and space to think in answering the question. At the same time, this expression is more likely to create a positive, friendly and respectful atmosphere of communication, which will help to increase the effectiveness of the questionnaire and the participation of the respondent."}, "time_dimension": {"answer": "Present", "rationale": "In the future, the entry contains assumptions and expectations, in which \"if\" indicates the future condition, \"sure\" indicates a strong projection and expectation, pointing to the future act or result. The temporal dimension of the article is therefore the future."}, "sentence_type": "Affirmative declarative sentence", "pos": {"nouns": {"Questionnaire Entry": 1, "Help": 2, "he": 1, "person": 1}, "verbs": {"Promise": 1, "Yes": 1, "Help": 2, "Answer": 1, "I": 1}, "adverbs": {"Just": 1}, "numerals": {"14": 1}, "pronouns": {"He/she": 1, "I'm sure": 1}}}, {"content": "If _ think I haven't dealt with something, he/she won't criticize me in front of others.", "word_count": 25, "label": "", "sentence_structure": "Subject鈥搗erb鈥搊bject", "subjective_objective": {"answer": "Subjective", "rationale": "Because the sentence expresses the patient's perception and expectations of others (i.e. \" he/she \" ), it relates to the patient's own subjective feelings and judgement."}, "sentence_sentiment": {"answer": "Positive", "rationale": "Because the behavior in the entry \"will not criticize me in front of others\" is positive."}, "language_style": {"answer": "Calm and direct", "rationale": "The entry is in plain and simple language, asking whether the person who is being tried is critical of himself in the face of others."}, "time_dimension": {"answer": "Present", "rationale": ""}, "sentence_type": "Interrogative sentence", "pos": {"nouns": {"Yes": 1}, "verbs": {"I think": 1, "Take care of it": 1, "Yes": 1, "Criticism": 1}, "adverbs": {"he": 1, "she": 1, "In front of someone else": 1}, "numerals": {"15": 1}, "pronouns": {"I": 2}}}, {"content": "If I told myself what he/she was worried about, he/she would not think that my fear seemed silly.", "word_count": 26, "label": "", "sentence_structure": "Subject鈥搗erb鈥搊bject", "subjective_objective": {"answer": "Subjective", "rationale": "Because I'm subjective in my own way, and that's what affects the other people's reaction to me. So it's my subjective idea."}, "sentence_sentiment": {"answer": "Positive", "rationale": "It is positive because it emphasizes that the person concerned will not be ridiculed when he or she is told his or her own fears, giving a sense of support and understanding."}, "language_style": {"answer": "Tactful and warm", "rationale": "The language style of the entry tends to be gentle and inviting rather than direct statements or orders. It guides the interviewee to answer questions in a gentle manner and encourages him to express his thoughts and feelings. This expression is more personal and interactive, helping to create a more comfortable and free expression of his views and opinions."}, "time_dimension": {"answer": "Present", "rationale": ""}, "sentence_type": "Negative declarative sentence", "pos": {"nouns": {"self": 1, "I'm worried": 2, "What": 1, "he": 1, "she": 1}, "verbs": {"Tell": 1, "Yes": 1, "I think": 1, "It seems": 1, "Stupid": 1}, "adverbs": {"very": 1}, "numerals": {"16": 1, "2": 1}, "pronouns": {"If": 1}}}, {"content": "17. If my alarm clock is broken and I ask to call at some point, I will definitely get a call.", "word_count": 33, "label": "", "sentence_structure": "Subject鈥搗erb鈥搊bject", "subjective_objective": {"answer": "Objective", "rationale": "Since requests and calls are objective events, they are not related to the patient ' s subjective attitude."}, "sentence_sentiment": {"answer": "Positive", "rationale": "Because the entry describes a positive act, i.e., the other party will help wake up if needed."}, "language_style": {"answer": "Tactful and warm", "rationale": "The entry is more adept and interactive when it is spoken in a gentle and inviting manner, rather than by direct statements or orders."}, "time_dimension": {"answer": "Present", "rationale": "According to the phrase \"if\" and \"if I will,\" it is a hypothetical scenario. But, because it is a hypothetical statement, it is actually a description of the current mentality or belief (i.e., when an alarm clock breaks, asking someone to call and wake up is certain to get a call). So the time dimension of the article is now."}, "sentence_type": "Affirmative declarative sentence", "pos": {"verbs": {"Bad": 1, "Please": 3, "Answer": 1}, "adverbs": {"If": 1, "of": 2, "Sure": 1, "Time": 1}, "numerals": {"One": 1, "Some": 1}, "pronouns": {"I": 1, "And": 1, "Telephone": 2}}}, {"content": "18. If _ fails to meet me as planned, I am sure that he/she has met something important.", "word_count": 26, "label": "", "sentence_structure": "Subject鈥搗erb鈥搊bject", "subjective_objective": {"answer": "Subjective", "rationale": "I believe it's the patient's subjective attitude."}, "sentence_sentiment": {"answer": "Positive", "rationale": "Because \"I believe he/she must have met something important\" in the entry is a positive expression of an emotion of understanding, trust and concern."}, "language_style": {"answer": "Tactful and warm", "rationale": "The language style of the entry is more euphemistic, because it is expressed in a sense of understanding and care. For example, the words \"I believe\" show understanding and respect for each other, rather than direct blame or criticism. At the same time, the use of \"must encounter something important\" to explain why it failed to meet the plan, is also mild and does not create much pressure or anxiety."}, "time_dimension": {"answer": "Future", "rationale": "The words \"if\" and \"be sure\" both imply that this is a vision or a presumption for the future, so the entry is about the future."}, "sentence_type": "Interrogative sentence", "pos": {"nouns": {"Entry": 1, "Questionnaire": 1, "Planned": 1, "he": 1, "she": 1, "Things": 1}, "verbs": {"Analysis": 1, "Not": 1, "Press": 1, "Yes": 1, "Sure": 1, "I met him": 1, "I've got it": 1}, "adjectives": {"Important": 1}, "numerals": {"18": 1}, "pronouns": {"If": 1, "he": 1, "she": 1}}}, {"content": "If _ were to drive me somewhere but he/she was not able to come on time, I would assume that he/she had good reasons.", "word_count": 37, "label": "", "sentence_structure": "Subject鈥搗erb鈥搊bject", "subjective_objective": {"answer": "Subjective", "rationale": "Because it's a patient's guess and idea, it's a subjective examination."}, "sentence_sentiment": {"answer": "Neutral", "rationale": "As this entry describes a hypothetical situation, it does not involve any emotional or attitudinal tendencies."}, "language_style": {"answer": "Tactful and warm", "rationale": "The language style of this questionnaire entry is more euphemistic and inclusive, using a more moderate tone to guide respondents to think about issues, without showing a direct or strong emotional tone. Moreover, it contains some implicit words, such as \"there must be a good reason,\" which may make the respondents more willing to share their views. So I think the language style of the questionnaire is very euphemistic."}, "time_dimension": {"answer": "Present", "rationale": "The entry asks how the person tested would think and react in a given case, and the time dimension is now judged."}, "sentence_type": "Affirmative declarative sentence", "pos": {"nouns": {"Ready": 1, "Car": 1, "Local": 1, "person": 1, "Rationale": 1}, "verbs": {"Drive": 1, "Send": 1, "Come on": 1, "Guess": 1, "have": 1}, "adverbs": {"On time": 1}, "numerals": {"he": 2, "she": 2, "I'm sure": 1}, "classifiers": {"EMPTY": 1}, "pronouns": {"I": 1}}}, {"content": "I can count on the most appropriate arrangements for me if I am injured or injured.", "word_count": 25, "label": "", "sentence_structure": "Subject鈥搗erb鈥搊bject", "subjective_objective": {"answer": "Subjective", "rationale": "Because the title \"I\" shows that it's the patient's subjective idea."}, "sentence_sentiment": {"answer": "Positive", "rationale": "Because the behavior in the entry \"make the most appropriate arrangements for me\" is positive."}, "language_style": {"answer": "Tactful and warm", "rationale": ""}, "time_dimension": {"answer": "Present", "rationale": "The temporal dimension of this questionnaire entry is therefore judged to be present and future, as it relates to the current situation (injury) and the possible future situation (needing the help of others)."}, "sentence_type": "Interrogative sentence", "pos": {"nouns": {"Wounded": 2, "Hurt": 1, "The most appropriate arrangements": 1}, "verbs": {"Yes": 1, "I hope": 1, "Substitute": 1, "Do it": 1, "Out": 1}, "numerals": {"1": 2}, "pronouns": {"I": 1}}}, {"content": "If _ borrows a valuable item but it is damaged when returned, _ pays me the repair fee.", "word_count": 27, "label": "", "sentence_structure": "Subject鈥搗erb鈥搊bject", "subjective_objective": {"answer": "Objective", "rationale": "Because it is a descriptive statement, it does not involve subjective attitudes or feelings of the person interviewed. It is merely a statement of a possible event and outcome."}, "sentence_sentiment": {"answer": "Positive", "rationale": "Because it expresses a just and fair attitude of behaviour."}, "language_style": {"answer": "Tactful and warm", "rationale": "The language style of this questionnaire entry is euphemism and warmth. It is a matter of obtaining information by asking about the behaviour of the other party in a given situation, rather than direct orders or instructions. At the same time, using a phrase structure like \"if but\" makes the question more polite and friendly, reflecting respect and understanding of the person under investigation."}, "time_dimension": {"answer": "Present", "rationale": "The entry describes the situation in which a particular act (i.e., \"to borrow something worth of money\") occurs in the present context, which leads to another act (i.e. \"to pay me for repairs\". This situation is based on the situation of borrowed goods that is common in real life and emphasizes the obligations and responsibilities of both parties in such cases. Thus, in terms of time dimension, the entry describes the current situation."}, "sentence_type": "Interrogative sentence", "pos": {"nouns": {"Annex": 1, "Value": 1, "Something": 2, "Repairs": 1, "I": 1}, "verbs": {"Excuse me": 1, "Return": 1, "I'll pay": 1, "Repair": 1}, "adjectives": {"Damage": 1}, "numerals": {"2": 1}, "pronouns": {"锛?: 2}}}, {"content": "If my alarm clock is broken and I ask to call me at some point, I will certainly get a call.", "word_count": 35, "label": "", "sentence_structure": "Subject鈥搗erb鈥搊bject", "subjective_objective": {"answer": "Subjective", "rationale": "Because it describes a person's belief or expectation in a particular situation and is based on their own experience or belief, it is examined from the perspective of the whole."}, "sentence_sentiment": {"answer": "Positive", "rationale": "Because the entry expresses a sense of trust and dependence, it is a positive attitude and behaviour."}, "language_style": {"answer": "Calm and direct", "rationale": "The language style of the questionnaire entry is more calm and straightforward, and is not expressed in excessive rhetorical or emotional terms, but rather in a direct presentation of facts and assumptions, giving people an objective and clear sense of the word."}, "time_dimension": {"answer": "Present", "rationale": "In terms of time dimension, this item is described as covering both the present (if my alarm clock is broken) and the future (I am sure that I will be able to get a call by then). Thus, the purpose of the article is both present and future."}, "sentence_type": "Interrogative sentence", "pos": {"nouns": {"Alarm clock": 1, "Telephone": 2}, "verbs": {"Bad": 1, "Request": 1, "Fight": 1, "Please": 1, "Answer": 1}, "adverbs": {"Sure": 1, "And then": 1}, "classifiers": {"One": 2}, "pronouns": {"I": 1}}}, {"content": "If _ promised to feed my pets while I was gone, I would not have to worry about the lack of care for the pets.", "word_count": 28, "label": "", "sentence_structure": "Subject鈥搗erb鈥搊bject", "subjective_objective": {"answer": "Subjective", "rationale": "Because the sentence relates to patients ' concerns about whether others can take the place of their pets, it is the patient ' s subjective idea and attitude."}, "sentence_sentiment": {"answer": "Positive", "rationale": "Because the entry describes someone who is willing to help with his pets when he is not home, it is a positive social act."}, "language_style": {"answer": "Tactful and warm", "rationale": "1. The euphemism of \"promising to feed my pets while I'm away\" was used rather than direct orders or demands. 2. The trust and gratitude of the questionnaire was conveyed by expressing \"I don't need to worry about the lack of care for the pets\". 3. The whole sentence was smooth and warm, and did not have too much emotional overtones or strong emotional overtones."}, "time_dimension": {"answer": "Future", "rationale": "The keywords \"if\" and \"promise that when I'm not\" indicate that this is the vision and expectation for the future, and therefore is judged as the future of behaviour or mentality."}, "sentence_type": "Interrogative sentence", "pos": {"nouns": {"Questionnaire Entry": 1, "Answer": 1, "Time": 1, "I": 1, "not": 1, "in": 1, "Substitute": 1, "Hey": 1, "Pets": 2, "No need": 1, "I'm worried": 1, "Care": 1}, "verbs": {"If": 1, "Answers should be": 1, "Substitute": 1, "Hey": 1, "No need": 1, "I'm worried": 1}, "adverbs": {"When": 1, "Time": 1}, "numerals": {"4": 1}, "pronouns": {"锛?: 1, "I": 2, "not": 1}}}, {"content": "If _ promised to help me, he/she would help me.", "word_count": 16, "label": "", "sentence_structure": "Subject鈥搗erb鈥搊bject", "subjective_objective": {"answer": "Subjective", "rationale": "Because this is a subjective attitude to the expectations and trust of the patient towards someone."}, "sentence_sentiment": {"answer": "Positive", "rationale": "Because \"must\" in the entry indicates trust and expectation, and it's a positive emotional color."}, "language_style": {"answer": "Calm and direct", "rationale": "The entry directly expresses the fact that if the other party had promised to help, he/she would have actually fulfilled his/her commitments, without using excessive rhetoric or emotional rendering, and with a more balanced and objective language style."}, "time_dimension": {"answer": "Present", "rationale": "Key words \"if\" , \"yes\" and \"yes\" are all words that describe the current situation, indicating that the testee is considering the current situation and making assumptions. The entry is therefore in the presence of the interviewee."}, "sentence_type": "Affirmative declarative sentence", "pos": {"nouns": {"Help": 1, "I": 2, "He/she": 1}, "verbs": {"Promise": 1, "Yes": 1, "Help": 1}, "adverbs": {"If": 1, "I'll bet": 1}, "numerals": {"5": 1}}}, {"content": "If he/she would drive me somewhere but he/she would not be here on time, I would assume that he/she would have good reasons.", "word_count": 34, "label": "", "sentence_structure": "Subject鈥搗erb鈥搊bject", "subjective_objective": {"answer": "Subjective", "rationale": "This is a subjective idea that involves the patient ' s interpretation and inference of the behaviour of another person."}, "sentence_sentiment": {"answer": "Positive", "rationale": "Because it's a \"good reason\" in the entry, it's a positive and optimistic attitude."}, "language_style": {"answer": "Tactful and warm", "rationale": ""}, "time_dimension": {"answer": "Present", "rationale": ""}, "sentence_type": "Affirmative declarative sentence", "pos": {"nouns": {"Questionnaire": 1, "Entry": 1, "Drive": 1, "Local": 1, "he": 1, "she": 1, "Rationale": 1}, "verbs": {"I'll drive": 1, "Send it to him": 1, "Guess": 1, "have": 2, "I'm sure": 1, "Present": 1, "Come on": 1}, "adverbs": {"If": 1, "Not": 1, "very": 1}, "numerals": {"6": 1, "One": 3, "I'm sure": 1}, "classifiers": {"individual": 1}, "pronouns": {"I": 1}}}, {"content": "7. I would be willing to lend money to _, at any amount he/she can, as soon as he/she is able to do so.", "word_count": 27, "label": "", "sentence_structure": "Subject鈥搗erb鈥搊bject", "subjective_objective": {"answer": "Subjective", "rationale": "Because \"yes\" and \"get me back as soon as possible\" are subjective attitudes and ideas of patients."}, "sentence_sentiment": {"answer": "Positive", "rationale": "Because the purpose of the article is positive, it expresses a willingness to help others and a desire for timely return."}, "language_style": {"answer": "Tactful and warm", "rationale": "The language style of the entry is more euphemistic and friendly, using words such as \"will,\" \"because he/she will return me as soon as he/she can.\" At the same time, \"for whatever money you borrow\" shows trust and support for each other, which is warm and comfortable."}, "time_dimension": {"answer": "Present", "rationale": "The entry now asks the testee about the attitude and behaviour of the person borrowing from a particular person, and the time dimension is now judged."}, "sentence_type": "Affirmative declarative sentence", "pos": {"nouns": {"Money": 2, "he": 1, "she": 1}, "verbs": {"Yes": 1, "Borrow": 1, "And": 1, "Here": 1, "Okay": 1, "have": 1, "Capacity": 1}, "adverbs": {"As soon as possible": 1}, "numerals": {"7": 1, "More": 1}, "classifiers": {"individual": 1}, "pronouns": {"I": 1}}}, {"content": "If _ fails to meet me as planned, I believe that he/she must have met something important.", "word_count": 26, "label": "", "sentence_structure": "Subject鈥搗erb鈥搊bject", "subjective_objective": {"answer": "Subjective", "rationale": "Because this is the patient ' s own conviction and hypothesis, it is subjective."}, "sentence_sentiment": {"answer": "Neutral", "rationale": "Because the entry merely expresses a point of view, it does not reflect a positive or negative emotional colour."}, "language_style": {"answer": "Tactful and warm", "rationale": "The language style of the entry is euphemism, because it is more euphemistic, polite, not directly accusing or critical, but rather expressing understanding of what is important to each other in an understanding and support. At the same time, the phrase is used in the form of \"I believe\" and further demonstrates positive feelings of concern and trust, increasing the interaction and proximity of the questionnaire to the respondents."}, "time_dimension": {"answer": "Past", "rationale": "Taken together, the content of this questionnaire entry is a survey of abstract concepts of trust, understanding and not of specific past, present or future behaviour or mindset."}, "sentence_type": "Affirmative declarative sentence", "pos": {"nouns": {"Questionnaire Entry": 1, "person": 2, "Something important": 1}, "verbs": {"Analysis": 1, "Yes": 1, "I met him": 1, "I couldn't meet him as planned": 1, "I'm sure": 1, "he": 1, "she": 1}, "adjectives": {"Important": 1}, "adverbs": {"I'm sure": 1}, "pronouns": {"8": 1, "If": 1, "I": 2}}}, {"content": "I can talk to _, and I know _ would like to hear.", "word_count": 16, "label": "", "sentence_structure": "Subject鈥搗erb鈥搊bject", "subjective_objective": {"answer": "Objective", "rationale": "Because the sentence \"I know\" is a patient's subjective judgment, and \"can talk\" and \"will listen\" are the attitudes of the other, and it's objective."}, "sentence_sentiment": {"answer": "Positive", "rationale": "The article ' s expression expresses positive attitudes and behaviour, i.e., the freedom to talk freely and the knowledge that the other party is willing to listen."}, "language_style": {"answer": "Tactful and warm", "rationale": "The entry is a soft, invitation-style conversation, using the words \"may\" and \"asever\" to express an open and receptive attitude and to emphasize the will of the other, and is more adept and interactive, in keeping with the euphemism of the language."}, "time_dimension": {"answer": "Present", "rationale": "The entry indicates that the person tested is able to talk to someone at any time and knows that the other person is willing to listen, which is the current and present behaviour."}, "sentence_type": "Interrogative sentence", "pos": {"nouns": {"Talk": 1, "Yes": 1}, "verbs": {"Yeah": 1, "I'm tired": 1, "and": 1, "May I": 1, "Listen": 1}, "adverbs": {"I'm tired": 1}, "pronouns": {"I": 1, "锛?: 2}}}, {"content": "I'm not going to be deliberately distorted to convey my views to anyone.", "word_count": 18, "label": "", "sentence_structure": "Subject鈥搗erb鈥揷omplement", "subjective_objective": {"answer": "Subjective", "rationale": "Because the question is whether the patient will deliberately distort the facts in conveying his/her views, it is the patient ' s subjective attitude."}, "sentence_sentiment": {"answer": "Positive", "rationale": "Because the entry is an expression of honesty and integrity and is not negative in emotional terms."}, "language_style": {"answer": "Calm and direct", "rationale": "The entry uses a direct and concise language, which is not accompanied by too many qualifiers or emotional colours."}, "time_dimension": {"answer": "Present", "rationale": "So the temporal dimension of the topic is judged as \"now\"."}, "sentence_type": "Negative declarative sentence", "pos": {"verbs": {"Never": 1, "On purpose": 1, "Misdirection": 1, "Will": 1, "Mine": 2, "Views": 1, "Transmission": 1, "Here": 1, "others": 1}, "adverbs": {"Never": 1, "On purpose": 1, "Misdirection": 1}, "numerals": {"10": 1}, "pronouns": {"I": 1}}}, {"content": "11. If _ knew what was hurting my feelings, he would not use it against me even if our relationship changed.", "word_count": 42, "label": "", "sentence_structure": "Subject鈥搗erb鈥搊bject", "subjective_objective": {"answer": "Subjective", "rationale": "Because it is the patient's subjective idea and expectations."}, "sentence_sentiment": {"answer": "Positive", "rationale": "Because the expression of the purpose of the article contained positive intent and conduct, i.e. the other party would not use harm to itself to retaliate, it was positive."}, "language_style": {"answer": "Tactful and warm", "rationale": "The entry is more gentle in its language style. It uses terms such as \"if\" and \"so\" to express assumptions and expectations about the other, not directly blaming or ordering the other, but rather directing the other to thinking in a more soft and calm way. Moreover, the end of the sentence expresses understanding and respect for the other, and this attitude is warmer and more cordial."}, "time_dimension": {"answer": "Future", "rationale": "The entry should therefore be classified as future."}, "sentence_type": "Interrogative sentence", "pos": {"nouns": {"Things": 1, "Hurt": 1, "Emotion": 1, "Relations": 1, "he": 1}, "verbs": {"Yes": 1, "be": 1, "Come on": 1, "Change": 1, "Utilization": 1, "Deal": 1}, "adjectives": {"What": 1, "Even if": 1, "These": 1}, "adverbs": {"Yeah": 1}, "numerals": {"11": 1}, "pronouns": {"If": 1, "Well": 1, "I": 1}}}, {"content": "I was able to tell 鈥?and know that he/she would not talk to others about my concerns.", "word_count": 27, "label": "", "sentence_structure": "Subject鈥搗erb鈥搊bject", "subjective_objective": {"answer": "Subjective", "rationale": "Enter: \"12. I can tell -- and know that he/she won't talk to anyone about what I care about.\" And output: \"The subjectivity is the patient's perception and feelings of someone he/she can trust.\""}, "sentence_sentiment": {"answer": "Positive", "rationale": "Because the entries describe acts that can trust and share secrets, this is a positive quality."}, "language_style": {"answer": "Tactful and warm", "rationale": "The entry expresses a relationship of trust and dependency by using terms such as \"can\" and \"know\" and is more gentle and friendly. At the same time, the main phrase is the plural form of first and second names, giving people a sense of equality and friendship. Thus, we can judge the language style of the entry as euphemism."}, "time_dimension": {"answer": "Present", "rationale": "The entry asks if the person who was tested is currently able to trust and tell secrets to him, and the time dimension is now judged."}, "sentence_type": "Affirmative declarative sentence", "pos": {"nouns": {"Questionnaire Entry": 1, "Secrets": 1, "person": 2, "others": 1, "Yes": 1}, "verbs": {"Yes": 2, "In": 1, "Dump": 1, "No, I won't": 1, "Arguments": 1}, "adverbs": {"And": 1}, "numerals": {"12": 1}, "classifiers": {"individual": 1}, "pronouns": {"鈥?: 1, "he": 1, "she": 1}}}, {"content": "I believe that _ will tell me the truth.", "word_count": 12, "label": "", "sentence_structure": "Subject鈥搗erb鈥揷omplement", "subjective_objective": {"answer": "Subjective", "rationale": "Because \"convincing\" is the patient's subjective attitude or belief, and \"the truth of the facts\" is not an objective thing, it is a question of examining the patient's subjective thoughts."}, "sentence_sentiment": {"answer": "Positive", "rationale": "Because \"rebel\" is positive."}, "language_style": {"answer": "Calm and direct", "rationale": "The entry is directly asked, is not overly pedantic or emotional, is more objective and meets the linguistic style requirements of the questionnaire."}, "time_dimension": {"answer": "Future", "rationale": "The keyword \"will tell\" and \"the truth of the facts\" indicates that the entry asks the testee for a future or belief, and is therefore judged to be the future."}, "sentence_type": "Affirmative declarative sentence", "pos": {"nouns": {"Grandpa": 1, "Questionnaire": 1, "Entry": 1, "The truth": 1}, "verbs": {"Yes": 1, "Tell": 1, "Here": 1}, "adverbs": {"Yes": 1}, "numerals": {"13": 1}, "pronouns": {"I": 1}}}]}

{"questionnaire_title": "Trust in others' scales.", "questionnaire_category": "Mental health", "item_count": 5, "emotion_shift_overall": "From positive to negative", "language_style_overall": "Tactful and warm", "items": [{"content": "1. Some say that most people are trustworthy, others say that special care must be taken when dealing with others. What do you think?", "word_count": 36, "label": "", "sentence_structure": "Subject鈥搗erb鈥搊bject", "subjective_objective": {"answer": "Subjective", "rationale": "Because the subject is asking about the views of individuals on \"Are most people trustworthy\" and \"The need to be particularly careful when dealing with others\", this is a subjective issue."}, "sentence_sentiment": {"answer": "Neutral", "rationale": "Because the entry asks the respondents about the different views, it does not explicitly express positive or negative emotional tendencies."}, "language_style": {"answer": "Tactful and warm", "rationale": "\"Slow and warm.\""}, "time_dimension": {"answer": "Past", "rationale": ""}, "sentence_type": "Interrogative sentence", "pos": {"nouns": {"person": 2, "Others": 1, "- To be with someone": 1, "Observations": 1, "Questionnaire": 1, "Entry": 1}, "verbs": {"be": 1, "It's worth it": 1, "Trust": 1, "Say it": 2, "I have to": 1, "Special": 1, "Watch out": 1, "How's that": 1, "And": 1}, "adjectives": {"Most people": 1}, "adverbs": {"Yeah": 1}, "numerals": {"1": 9}, "classifiers": {"individual": 1}, "pronouns": {"you": 1}}}, {"content": "Do you think most people like to help others or do you just like to take care of yourself?", "word_count": 22, "label": "", "sentence_structure": "Subject鈥搗erb鈥搊bject", "subjective_objective": {"answer": "Subjective", "rationale": "Because \"like\" is a subjective attitude or view, it is the view or opinion of the subject that is examined and is subjective in nature."}, "sentence_sentiment": {"answer": "Positive", "rationale": "Because the \"like to help\" attitude is considered positive."}, "language_style": {"answer": "Calm and direct", "rationale": "The entry is asked in a direct and clear manner, without excessive rhetorical or emotional rendering, and is more objective in tone, with the aim of obtaining the other side ' s view of whether most people prefer to help others or simply care for themselves."}, "time_dimension": {"answer": "Present", "rationale": "The entry asks what the testee is currently thinking and understanding of the majority, so the time dimension is now judged."}, "sentence_type": "Interrogative sentence", "pos": {"nouns": {"person": 1, "Help": 1, "others": 1, "Care": 1, "self": 1}, "verbs": {"I think": 1, "Yeah": 2}, "adverbs": {"Most": 1, "Only": 1}, "numerals": {"2": 1}}}, {"content": "3. If you are not careful, people will take advantage of you.", "word_count": 17, "label": "", "sentence_structure": "Subject鈥搗erb鈥搊bject", "subjective_objective": {"answer": "Subjective", "rationale": "Because this view is an individual idea or view, it reflects the subjective understanding of interpersonal relationships among patients."}, "sentence_sentiment": {"answer": "Negative", "rationale": "Because the \"take advantage\" act in the entry is negative."}, "language_style": {"answer": "Calm and direct", "rationale": "This entry expresses the view that if a person is not careful, others may use him or her. Such language style is more objective and rational, and does not have too much emotional or rhetorical style."}, "time_dimension": {"answer": "Present", "rationale": "The \"you\" and \"will\" in the entry indicate that this is a description of the current situation, while \"if you are not careful\" is a conditional phrase, which is a hypothetical situation. So, the time dimension of the entry is now."}, "sentence_type": "Interrogative sentence", "pos": {"nouns": {"self": 1, "person": 2, "Cheap": 1}, "verbs": {"I was just": 1, "Jim": 1}, "adverbs": {"Just": 1}, "numerals": {"3": 1}, "pronouns": {"you": 1}}}, {"content": "When you focus on something, no one will care what happens to you.", "word_count": 23, "label": "", "sentence_structure": "Subject鈥搗erb鈥搊bject", "subjective_objective": {"answer": "Subjective", "rationale": "Because it is the patient ' s perception and feelings of the matter, it is subjective."}, "sentence_sentiment": {"answer": "Neutral", "rationale": "As this entry does not express positive or negative emotions."}, "language_style": {"answer": "Tactful and warm", "rationale": "The entry is more gentle and gentle. It uses a more peaceful way to convey a message that when people focus on something, the outside world does not interfere or care too much about what they are going through. Such a expression is more friendly and understanding and is more likely to receive positive responses from the respondents."}, "time_dimension": {"answer": "Present", "rationale": ""}, "sentence_type": "Interrogative sentence", "pos": {"nouns": {"Things": 2, "person": 1}, "verbs": {"Focus": 1, "Processing": 1, "Yes": 2, "Care": 1, "I met him": 1}, "adjectives": {"Focus": 1}, "numerals": {"4": 1, "Two": 1}, "pronouns": {"you": 1}}}, {"content": "5. People are essentially cooperative.", "word_count": 15, "label": "", "sentence_structure": "Subject鈥揷opula鈥損redicative", "subjective_objective": {"answer": "Subjective", "rationale": "Because \"a spirit of cooperation\" is an individual's view and conviction, and it's a subjective perspective."}, "sentence_sentiment": {"answer": "Positive", "rationale": "Because \"the spirit of cooperation\" is a positive attitude and quality."}, "language_style": {"answer": "Tactful and warm", "rationale": "The language style of the entry is more euphemistic, because it uses positive language and is relatively soft. It does not directly deny or criticize, but rather presents a point of view that the interviewee wishes to agree with. This expression is more resonant and responsive to the interviewee."}, "time_dimension": {"answer": "Present", "rationale": "The keyword \"humans\" and \"cooperative spirit\" in essence suggests that this is a view of human nature or universal phenomena, and does not relate to past or future situations. Thus, the temporal dimension is now judged."}, "sentence_type": "Affirmative declarative sentence", "pos": {"nouns": {"person": 2, "Spirit": 1}, "verbs": {"Speak": 1, "be": 1, "Rich": 1, "Cooperation": 1}, "adjectives": {"In essence": 1}, "numerals": {"5": 1}}}]}

{"questionnaire_title": "Mental health test sheet PHI", "questionnaire_category": "Mental health", "item_count": 168, "emotion_shift_overall": "From negative to positive", "language_style_overall": "Neutral", "items": [{"content": "One, I get up in the morning, I feel like I'm sleeping enough and I'm awake.", "word_count": 20, "label": "", "sentence_structure": "Subject鈥搗erb鈥揷omplement", "subjective_objective": {"answer": "Subjective", "rationale": "Because \"thinks\" is a subjective expression of feeling."}, "sentence_sentiment": {"answer": "Positive", "rationale": "Because the description \"Being up in the morning, feeling asleep, awake\" is positive and expresses positive emotional attitudes."}, "language_style": {"answer": "Tactful and warm", "rationale": "The language style of the entry is relatively euphemistic and sweet, using the descriptions of \"mostly\" and \"bright mind\" to give a more positive impression, rather than simply indicating whether the interviewee feels asleep enough or clear. Moreover, the phrase \"when he wakes up in the morning\" is a more intimate and natural one."}, "time_dimension": {"answer": "Present", "rationale": ""}, "sentence_type": "Affirmative declarative sentence", "pos": {"nouns": {"Time": 1, "Sleep": 1, "Brain": 1}, "verbs": {"Rise": 1, "I think": 1, "Enough": 1, "Clear": 1}, "adjectives": {"Morning": 1, "Mostly": 1, "Wake up": 1}, "numerals": {"1": 2}, "pronouns": {"I": 1}}}, {"content": "I'm easily woken up.", "word_count": 7, "label": "", "sentence_structure": "Subject鈥揷opula鈥損redicative", "subjective_objective": {"answer": "Subjective", "rationale": "Because \"fright to wake up\" is a subjective feeling, different people may experience and judge differently, and it is therefore viewed from the patient's subjective perspective."}, "sentence_sentiment": {"answer": "Negative", "rationale": "Because \"always waking up\" is a negative situation or experience."}, "language_style": {"answer": "Calm and direct", "rationale": "The entry directly expresses the question of whether the subject is easily awakened, does not use excessive rhetorical or emotional rendering, and is more objective in tone."}, "time_dimension": {"answer": "Present", "rationale": "The keyword \"I am easily awaken\" expresses the sense and state of the moment, without information about the past or the future."}, "sentence_type": "Affirmative declarative sentence", "pos": {"verbs": {"Easy": 1, "Wake up": 1}, "adjectives": {"very": 1}, "adverbs": {"Land": 1}}}, {"content": "3 - My daily life is filled with things that interest me.", "word_count": 18, "label": "", "sentence_structure": "Subject鈥揷opula鈥損redicative", "subjective_objective": {"answer": "Subjective", "rationale": "Because \"making yourself interested\" is a subjective feeling and attitude."}, "sentence_sentiment": {"answer": "Positive", "rationale": "Because the description \"filled with things that interest me\" is positive."}, "language_style": {"answer": "Tactful and warm", "rationale": "The entry is more euphemistic and warm, and raises questions about the researcher鈥檚 interest in the daily life of the person. At the same time, the tone is more natural and comfort and ease for the person."}, "time_dimension": {"answer": "Present", "rationale": "The entry describes the situation and feelings of the subjects in their daily lives, and the \"filled\" in it indicates that this is the state and experience of the present, so the temporal dimension is now judged."}, "sentence_type": "Affirmative declarative sentence", "pos": {"nouns": {"Life": 1, "Interest": 1, "Things": 1}, "verbs": {"Fill": 1, "鈾?And I'm gonna be 鈾?: 1, "Interested": 1}, "adverbs": {"Day": 1}, "numerals": {"3": 1}, "pronouns": {"I": 1}}}, {"content": "I'm working (learning) now, much like before.", "word_count": 16, "label": "", "sentence_structure": "Subject鈥搗erb鈥揷omplement", "subjective_objective": {"answer": "Subjective", "rationale": "Since the sentence deals with the assessment and comparison of the patient ' s abilities, it is based on the individual ' s feelings and perceptions and is therefore subjective."}, "sentence_sentiment": {"answer": "Neutral", "rationale": "Because the entry does not have any positive or negative emotional connotations, it is merely a description of an individual ' s assessment of his or her abilities."}, "language_style": {"answer": "Calm and direct", "rationale": "The entry is based on a simple and clear language that directly asks the other person whether the current work or learning abilities are comparable to those of the past, is not over-modified or emotionally expressed, is sound and objective, and is of a calm and direct language style."}, "time_dimension": {"answer": "Present", "rationale": "The entry asks about the current state and capabilities, and the time dimension is now judged."}, "sentence_type": "Affirmative declarative sentence", "pos": {"nouns": {"Capacity": 1, "Present": 1, "Work": 1, "Learning": 1}, "verbs": {"Almost": 1, "be": 1}, "adverbs": {"Before": 1}, "pronouns": {"I": 1}}}, {"content": "5. A person should know his or her dreams and receive guidance and warning.", "word_count": 22, "label": "", "sentence_structure": "Subject鈥搗erb鈥搊bject", "subjective_objective": {"answer": "Subjective", "rationale": "Because the title refers to \"Know your dreams and get guidance and warnings\", it is a subjective idea or attitude that examines the patient's personal views."}, "sentence_sentiment": {"answer": "Positive", "rationale": "Because the behavior in the entries \"to know your dream\" and the purpose \"to get guidance and warning from\" are positive."}, "language_style": {"answer": "Tactful and warm", "rationale": "The language style of the entry is more euphemistic, and the suggested way to guide the interviewee to reflect on the meaning of his dream, without using strong orders or direct negative tone."}, "time_dimension": {"answer": "Future", "rationale": "The keyword \"should understand\" and \"get guidance and warn\" suggests that it is a vision and goal setting, and therefore judgement is the future."}, "sentence_type": "Affirmative declarative sentence", "pos": {"nouns": {"person": 1, "Dreams": 1, "Guidance": 1, "Warning": 1}, "verbs": {"I think so": 1, "Go on": 1, "Roger that": 1, "Got it": 1}, "numerals": {"One": 2}, "classifiers": {"individual": 1}, "pronouns": {"self": 1}}}, {"content": "I think sometimes I think of something bad that I can't say.", "word_count": 16, "label": "", "sentence_structure": "Subject鈥搗erb鈥搊bject", "subjective_objective": {"answer": "Subjective", "rationale": "Because what is thought of is the patient ' s subjective thinking and memory, which may be real or fictional in themselves, this purpose is examined primarily from a subjective perspective."}, "sentence_sentiment": {"answer": "Negative", "rationale": "Because the \"Thinking of Something Bad\" in the entry is negative."}, "language_style": {"answer": "Tactful and warm", "rationale": "The topic is asked in a more moderate way whether the person surveyed had any bad intentions, avoiding direct blame or accusations. At the same time, using a description like \"bad-word\" makes the problem more euphemism and implicit. This language style makes it easier for the person surveyed to accept and share their ideas and facilitates the acquisition of more realistic and accurate data."}, "time_dimension": {"answer": "Present", "rationale": "The entry asks if the person tested occasionally thinks of something that makes him uncomfortable or unhappy and can therefore be judged as \"now\"."}, "sentence_type": "Affirmative declarative sentence", "pos": {"nouns": {"Yes": 1, "Bad": 1}, "verbs": {"Yes": 2, "Present": 1, "Say it": 1}, "adjectives": {"Out": 1, "mouth": 1, "Okay": 1, "Sometimes": 1}, "adverbs": {"None": 1}, "numerals": {"6": 1}, "pronouns": {"I": 1}}}, {"content": "I'm convinced that life is cruel to me.", "word_count": 11, "label": "", "sentence_structure": "Subject鈥揷opula鈥損redicative", "subjective_objective": {"answer": "Subjective", "rationale": "Because \"convincing\" is a subjective attitude and belief, an objective fact that is seen and interpreted. Therefore, it is a subjective idea and feeling that life is cruel to itself."}, "sentence_sentiment": {"answer": "Negative", "rationale": "Because the expression \"life is cruel to me\" in the entry is negative."}, "language_style": {"answer": "Calm and direct", "rationale": "The entry is a direct statement of the interviewee ' s views on life, and is not expressed in euphemism or warm language, so that the language style is straightforward and calm."}, "time_dimension": {"answer": "Past", "rationale": "The entry describes the testee ' s beliefs or attitudes to life and uses the general current to indicate that this is a persistent view and thus to judge as the past."}, "sentence_type": "Negative declarative sentence", "pos": {"nouns": {"Life": 1, "I": 2, "Cruel": 1}, "verbs": {"Yes": 1, "to": 1}, "adjectives": {"Cruel": 1}, "numerals": {"7": 1}, "pronouns": {"I": 1}}}, {"content": "I have very few problems with toilet failure.", "word_count": 11, "label": "", "sentence_structure": "Subject鈥搗erb鈥揷omplement", "subjective_objective": {"answer": "Objective", "rationale": "Because \"contaminated\" is an objective physical condition, whether it occurs or not, and is not influenced by the patient's subjective will. The entry is therefore considered from an objective perspective."}, "sentence_sentiment": {"answer": "Neutral", "rationale": "Because the entry does not have any emotional color."}, "language_style": {"answer": "Calm and direct", "rationale": "The entry is in a direct question-and-answer manner, with no excessive rhetorical or emotional rendering, and is more objective in tone and in line with the language style of the questionnaire."}, "time_dimension": {"answer": "Present", "rationale": ""}, "sentence_type": "Negative declarative sentence", "pos": {"nouns": {"8": 1, "Shit": 1, "Mistake": 1}, "verbs": {"have": 1, "All": 1}, "adjectives": {"Less": 1, "not": 1}, "adverbs": {"very": 1}, "numerals": {"How many": 1}, "pronouns": {"I": 1}}}, {"content": "9 - Sometimes I really want to leave home.", "word_count": 9, "label": "", "sentence_structure": "Subject鈥損redicate", "subjective_objective": {"answer": "Subjective", "rationale": "Because \"very much wants to leave home\" is a subjective description of what the patient feels inside."}, "sentence_sentiment": {"answer": "Negative", "rationale": "Because the idea of \"very much wanting to leave home\" in the entry is negative."}, "language_style": {"answer": "Calm and direct", "rationale": "The entry is in a straightforward tone asking the other side whether there is often a desire to leave the home, without too much emotional rendering or rhetorical techniques, and a more calm and objective language style."}, "time_dimension": {"answer": "Present", "rationale": ""}, "sentence_type": "Affirmative declarative sentence", "pos": {"verbs": {"Leave": 1, "Yes": 1}, "adjectives": {"Very": 1}, "pronouns": {"I": 1}}}, {"content": "I cry and laugh sometimes, and I can't even control myself.", "word_count": 18, "label": "", "sentence_structure": "Subject鈥搗erb鈥搊bject", "subjective_objective": {"answer": "Subjective", "rationale": "Because \"not even control yourself\" expresses the feelings and experiences of the patient."}, "sentence_sentiment": {"answer": "Negative", "rationale": "While crying and laughing are natural emotional reactions of people, the fact that even one cannot control itself may indicate that there is a high degree of mood volatility or emotional problems, and the entry is therefore negatively emotional."}, "language_style": {"answer": "Tactful and warm", "rationale": ""}, "time_dimension": {"answer": "Present", "rationale": "Now the entry describes the current behaviour or state of the subject, \"sometimes\", \"cry\" and \"uncontrollable\" indicating a customary, self-controlive mood swing. So the temporal dimension is now."}, "sentence_type": "Affirmative declarative sentence", "pos": {"nouns": {"Line": 2, "self": 1}, "verbs": {"Yes": 1, "Cry": 1, "Smile": 1, "Control": 1}, "adverbs": {"Sometimes": 1, "A while": 1}, "numerals": {"10": 1}}}, {"content": "11, it seems that no one knows me.", "word_count": 10, "label": "", "sentence_structure": "Subject鈥揷opula鈥損redicative", "subjective_objective": {"answer": "Subjective", "rationale": "Because it expresses the feelings and subjective view of the patient that no one understands himself, rather than the objective facts that exist."}, "sentence_sentiment": {"answer": "Negative", "rationale": "Because \"no one knows me\" in the entry expresses a feeling of loneliness and helplessness, and it's negative."}, "language_style": {"answer": "Tactful and warm", "rationale": "The language style of the entry is more euphemistic, friendly, and expresses a sense of uncertainty in a sentence beginning with \"seemingly\" rather than a direct determination of the behaviour or attitude of a person, which is more moderate and acceptable. At the same time, the use of \"no one knows me\" to convey his feelings and to make the person questioned concerned and concerned, rather than criticizing or blaming."}, "time_dimension": {"answer": "Present", "rationale": ""}, "sentence_type": "Negative declarative sentence", "pos": {"nouns": {"person": 1, "I": 2}, "verbs": {"Roger that": 1, "Nope": 1, "have": 1}, "adjectives": {"One": 1, "Seems like": 1}, "numerals": {"Eleven": 1}}}, {"content": "I want to be a singer.", "word_count": 8, "label": "", "sentence_structure": "Subject鈥損redicate", "subjective_objective": {"answer": "Subjective", "rationale": "Because \"want to be a singer\" is a personal wish or expectation, a subjective attitude of the patient."}, "sentence_sentiment": {"answer": "Positive", "rationale": "Because the attitude of \"want to be a singer\" is an expression of personal commitment and purpose."}, "language_style": {"answer": "Tactful and warm", "rationale": "The entry is presented in an encouraging and supportive manner, asking if the other party wants to become a singer, and is expressed in a more gentle and friendly manner, which can stimulate the other party鈥檚 motivation and participation. At the same time, the article is also more positive in its tone and helps to create a good Q & A atmosphere."}, "time_dimension": {"answer": "Present", "rationale": "The entry indicates the current thinking and desire of the speaker to become a singer. Although this desire may be realized in the future, at the current point of time it is a subjective expression of will. So, \"I want to be a singer\" is a current expression."}, "sentence_type": "Affirmative declarative sentence", "pos": {"nouns": {"Singers": 1}, "verbs": {"I want to be": 1}}}, {"content": "I'm gonna swear sometimes.", "word_count": 7, "label": "", "sentence_structure": "Subject鈥損redicate", "subjective_objective": {"answer": "Subjective", "rationale": "Because \"I really want to curse\" reflects the patient's feelings and thoughts, and it's a subjective examination."}, "sentence_sentiment": {"answer": "Negative", "rationale": "Because the \"want to curse\" act is negative."}, "language_style": {"answer": "Calm and direct", "rationale": "The language style of the entry was more direct, with no excessive reticulation or euphemism, but rather a straightforward and straightforward way of asking the respondents whether they had had any experience of abusive behaviour, which appeared to be relatively straightforward."}, "time_dimension": {"answer": "Present", "rationale": ""}, "sentence_type": "Imperative sentence", "pos": {"verbs": {"Time": 1, "Yes": 1, "Scolded": 1}, "adjectives": {"True": 1}, "adverbs": {"Sometimes": 1}}}, {"content": "I found it hard to focus on a job.", "word_count": 18, "label": "", "sentence_structure": "Subject鈥搗erb鈥搊bject", "subjective_objective": {"answer": "Subjective", "rationale": "Because the expression \"discovery\" and \"hard\" are the patient's personal feelings and subjective attitudes."}, "sentence_sentiment": {"answer": "Negative", "rationale": "Because the \"recognizing hard to focus\" behavior in the entry is negative."}, "language_style": {"answer": "Tactful and warm", "rationale": "The language style of the entry is more moderate, not direct criticism or command, but rather questions in a more understanding and caring manner."}, "time_dimension": {"answer": "Present", "rationale": "\"I find \"discovery\" means the most recent feeling or perception, and \"I have difficulty focusing on a job\" means a continuing situation. So, the entry should be the current time dimension."}, "sentence_type": "Imperative sentence", "pos": {"nouns": {"Attention": 1, "Work": 1}, "verbs": {"Found": 1, "Focus": 1}, "adjectives": {"It's hard": 1}, "numerals": {"14": 1, "One": 1}, "pronouns": {"I": 1}}}, {"content": "I had a very special, very strange experience.", "word_count": 14, "label": "", "sentence_structure": "Subject鈥搗erb鈥揷omplement", "subjective_objective": {"answer": "Subjective", "rationale": "Because the senses of \"special\" and \"unusual\" are usually subjective feelings and experiences of individuals."}, "sentence_sentiment": {"answer": "Neutral", "rationale": "Because the entry is merely a description of an experience and does not contain any emotional tendencies."}, "language_style": {"answer": "Tactful and warm", "rationale": "The language style of the entry is more euphemistic, using more subjective and sensitive terms (such as \"special\" and \"unusual\"), and leading the interviewee to a relatively gentle way of reminisce of the unique experiences of the past. This expression is more likely to arouse the emotional resonance of the interviewee and encourage them to share more real feelings."}, "time_dimension": {"answer": "Past", "rationale": ""}, "sentence_type": "Exclamatory sentence", "pos": {"nouns": {"Experience": 1}, "verbs": {"Yes": 1, "Special": 2, "Strange": 1}, "adjectives": {"very": 3}, "numerals": {"15": 1}}}, {"content": "16 I would have achieved much more if I had not been confronted.", "word_count": 20, "label": "", "sentence_structure": "Subject鈥搗erb鈥揷omplement", "subjective_objective": {"answer": "Subjective", "rationale": "Because this phrase expresses patients鈥?subjective attitude and belief that they can achieve more success without external interference. It is not an objective fact, but a belief in their abilities."}, "sentence_sentiment": {"answer": "Negative", "rationale": "Because the premise in the entry that \"if no one is against me\" is negative, implying distrust and hostility from outside, is a negative emotional attitude. And \"I will certainly achieve more\" is a positive expectation or target setting, but it does not change the emotional color of the article."}, "language_style": {"answer": "Calm and direct", "rationale": "The language style of the entry is more direct and objective, and there is no excessive emotional rendering or rhetorical technique. It raises a question in a direct way and wants to get a real answer from the other side, rather than trying to trigger emotional resonance or reflection. Thus, the language style of the article is \"cool and straight.\""}, "time_dimension": {"answer": "Present", "rationale": "The entry now represents the subjective view and conviction of the person being tested and does not address past or future acts or events, and thus the judgement is now."}, "sentence_type": "Negative declarative sentence", "pos": {"nouns": {"person": 1, "Achievements": 1}, "verbs": {"have": 1, "- Yes": 1, "Yes": 1}, "adjectives": {"More": 1, "Large": 1}, "adverbs": {"If not": 1}, "numerals": {"16": 1, "Bigger": 1}, "pronouns": {"I": 1}}}, {"content": "I'm not worried about my health.", "word_count": 10, "label": "", "sentence_structure": "Subject鈥揷opula鈥損redicative", "subjective_objective": {"answer": "Subjective", "rationale": "The fear of being a victim is a subjective judgement and feeling of the patient."}, "sentence_sentiment": {"answer": "Neutral", "rationale": "Because the entry merely stated a fact and did not contain any emotional colour."}, "language_style": {"answer": "Calm and direct", "rationale": "The entry directly asks whether the other party is often concerned about his or her health, is clear in language, is not over-modified or emotional and is a more calm and direct expression."}, "time_dimension": {"answer": "Past", "rationale": "The entry describes the past fears of the testee, and the keyword is \"very little\", which means that the testee has less concern for his or her health in the past. The dimension of time is therefore judged to be past."}, "sentence_type": "Negative declarative sentence", "pos": {"nouns": {"Health": 1}, "verbs": {"I'm worried": 1, "Less": 1}, "adjectives": {"self": 1}, "adverbs": {"Very few": 1}, "numerals": {"17": 1}}}, {"content": "18, I'd rather sit in my head than do anything.", "word_count": 20, "label": "", "sentence_structure": "Subject鈥搗erb鈥搊bject", "subjective_objective": {"answer": "Subjective", "rationale": "Because \"sitting\" and \"not doing anything\" are personal thoughts and feelings of patients, subjectively."}, "sentence_sentiment": {"answer": "Negative", "rationale": "Because \"I'd rather sit in the air than do anything\" is negative."}, "language_style": {"answer": "Tactful and warm", "rationale": "The language style of the entry is more euphemistic, leads the other side to answer by asking questions, uses the term \"better\" than \"no\" and is softer and more friendly."}, "time_dimension": {"answer": "Present", "rationale": ""}, "sentence_type": "Negative declarative sentence", "pos": {"nouns": {"Time": 2, "I": 2, "Things": 1}, "verbs": {"have": 1, "I'd rather": 1, "Sit down": 1, "And": 1, "I don't want to": 1, "Do it": 1}, "adjectives": {"A lot": 1, "Any": 1}, "adverbs": {"I'm not sure": 1}, "numerals": {"18": 1}, "classifiers": {"individual": 1}, "pronouns": {"This": 1}}}, {"content": "19 I did not want to do anything for days, weeks or months because I was always in a state of indecency.", "word_count": 29, "label": "", "sentence_structure": "Subject鈥搗erb鈥搊bject", "subjective_objective": {"answer": "Subjective", "rationale": "This emotional reaction is a subjective one because it is the patient ' s own feeling and experience."}, "sentence_sentiment": {"answer": "Negative", "rationale": "Because the words \"doesn't want to do anything\" and \"still can't lift a spirit\" in the entries convey a negative emotional state."}, "language_style": {"answer": "Tactful and warm", "rationale": "The entry is more personal and interactive when it is spoken in a gentle and inviting manner, rather than directly speaking or ordering. At the same time, the language style of the article is more euphemistic, using softer expressions such as \"I didn't want to do anything for days, weeks, months\" and \"because I can't always do anything about my spirit\" and avoiding language that is too direct or stimulating."}, "time_dimension": {"answer": "Past", "rationale": "The keyword \"had\" and \"always\" means that the act occurred in the past and could be repeated."}, "sentence_type": "Affirmative declarative sentence", "pos": {"nouns": {"Oh, my God": 1, "Week": 1, "Month": 1, "Fuck": 1, "Spirit": 1}, "verbs": {"Tsang": 1, "One-man": 1, "Yes": 1, "T": 1, "Rise": 1}, "adverbs": {"Always": 1, "Because": 1}, "numerals": {"A few days": 1, "How many": 1, "A few months": 1}, "pronouns": {"I": 2}}}, {"content": "I was up in bed and I was so easily woken up.", "word_count": 10, "label": "", "sentence_structure": "Subject鈥揷opula鈥損redicative", "subjective_objective": {"answer": "Subjective", "rationale": "Because Ann and the \"frightful awakening\" are patients' subjective feelings and experiences."}, "sentence_sentiment": {"answer": "Negative", "rationale": "Because \"sleeping uneasy\" and \"suspicion\" in the entries are negative behavioral descriptions."}, "language_style": {"answer": "Tactful and warm", "rationale": "This moderate tone reduces the respondents鈥?resistance and makes it easier to get real feedback."}, "time_dimension": {"answer": "Present", "rationale": "Now the entry is about the current state of the subject, the keywords being \"I sleep unsuspecting\" and \"I am easily awakening\" and they all indicate the behavior or state that is now being performed. So, the time dimension of this questionnaire entry is now."}, "sentence_type": "Affirmative declarative sentence", "pos": {"nouns": {"I": 1, "Sleep": 1, "Wake up": 1}, "verbs": {"Sleep well": 1, "Easy": 1, "By": 1}, "adjectives": {"Unsettled": 1}, "numerals": {"20": 1}}}, {"content": "I'm more critical than ever.", "word_count": 14, "label": "", "sentence_structure": "Subject鈥揷opula鈥損redicative", "subjective_objective": {"answer": "Subjective", "rationale": "Because \"my judgment\" is self-evaluation by the patient, and it's a subjective feeling."}, "sentence_sentiment": {"answer": "Positive", "rationale": "Because \"good judgment\" is a positive expression, which means that individuals are better able to judge."}, "language_style": {"answer": "Tactful and warm", "rationale": "The entry uses a self-affirming tone, asking if the other party believes that his or her judgment has improved, rather than directly evaluating or questioning. Such language style is more gentle, friendly, and easily comfortable and confident."}, "time_dimension": {"answer": "Future", "rationale": "Based on the phrase \"better than ever before\", it can be inferred that this is the expectation and the vision of the future, and thus the temporal dimension is the future."}, "sentence_type": "Affirmative declarative sentence", "pos": {"verbs": {"be": 1, "- Yeah": 1, "Before": 1, "Any": 1, "Time": 1, "Both": 1, "Okay": 1}, "adjectives": {"of": 2, "Previous": 1}, "numerals": {"21": 1}, "pronouns": {"I": 1}}}, {"content": "I've had at least one or two times a week, and suddenly I feel all hot and unprovoked.", "word_count": 24, "label": "", "sentence_structure": "Subject鈥搗erb鈥揷omplement", "subjective_objective": {"answer": "Subjective", "rationale": "Because \"I feel feverish for no reason\" is the patient's subjective feeling and experience."}, "sentence_sentiment": {"answer": "Neutral", "rationale": "Because the entry does not address any positive or negative emotional, attitudinal, behavioural, purpose or quality. It merely describes a person鈥檚 physical feelings."}, "language_style": {"answer": "Tactful and warm", "rationale": ""}, "time_dimension": {"answer": "Present", "rationale": "It is judged by the phrases \"weekly\" and \"at least one or two times\" to describe the current frequency and number; and \"at times, it suddenly feels the heat in the body\" is a description of the behaviour or state, and it refers to the current situation. The entry should therefore be classified as a description of the present behaviour or state."}, "sentence_type": "Affirmative declarative sentence", "pos": {"nouns": {"Week": 1, "Number of times": 1, "Heat": 1}, "verbs": {"have": 1, "I think": 1, "Yes": 1, "Heat": 2, "All over": 1, "Suddenly": 1, "For no reason": 1}, "adverbs": {"At least": 1, "Every": 1, "One": 1, "Two": 1, "Suddenly": 1, "For no reason": 1}, "numerals": {"Number of times": 1, "Week": 1, "Two": 1, "Heat": 2}, "pronouns": {"I": 1}}}, {"content": "It is preferable to take all laws away.", "word_count": 13, "label": "", "sentence_structure": "Subject鈥損redicate", "subjective_objective": {"answer": "Subjective", "rationale": "This sentence reflects the attitude and perception of the patient towards the law and is subjective."}, "sentence_sentiment": {"answer": "Negative", "rationale": "Because the idea of \"better not law\" in the entry is negative."}, "language_style": {"answer": "Calm and direct", "rationale": "The entry expresses a direct, strong and negative view of the law, without any euphemism or abating language, and is more ludicrous in its language style."}, "time_dimension": {"answer": "Past", "rationale": "The entry \"do not take all laws\" expresses an expectation and attitude, but there is no clear time point, and therefore judgement is the past."}, "sentence_type": "Negative declarative sentence", "pos": {"nouns": {"Law": 1}, "verbs": {"I'd better": 1, "be": 1, "- Put it on": 1, "Don't": 1}, "adverbs": {"All of them": 1}, "numerals": {"23": 1}}}, {"content": "I never felt pain or pain in my chest.", "word_count": 18, "label": "", "sentence_structure": "Subject鈥搗erb鈥搊bject", "subjective_objective": {"answer": "Objective", "rationale": "The feeling of chest pain or heartache is the patient ' s experience, but whether or not it is the patient ' s subjective view of it."}, "sentence_sentiment": {"answer": "Neutral", "rationale": "Because the entry describes only a physical feeling and does not involve any emotional attitudes or behaviour."}, "language_style": {"answer": "Calm and direct", "rationale": "The entry is based on a direct question and is formulated in a concise and concise manner, without too many emotional or rhetorical styles, so that the language style can be classified as \"cool and straight\"."}, "time_dimension": {"answer": "Present", "rationale": "The keyword \"never\" indicates that the act never happened, so its temporal dimension is now. Moreover, \"fearing\" is a description of the state of affairs, which is further confirmation of this."}, "sentence_type": "Negative declarative sentence", "pos": {"nouns": {"- Boobs": 1, "Heartache": 1, "Trouble": 1}, "verbs": {"Never": 1, "I feel": 1}, "adverbs": {"鈾?Never since 鈾?: 1}, "numerals": {"24": 1}, "pronouns": {"I": 1}}}, {"content": "When I was a kid, I was punished for being a kid.", "word_count": 18, "label": "", "sentence_structure": "Subject鈥搗erb鈥搊bject", "subjective_objective": {"answer": "Subjective", "rationale": "Because it is the patient ' s own memory of past experience and experience, it is the subjective feeling and memory of the individual."}, "sentence_sentiment": {"answer": "Neutral", "rationale": "Because the entry describes only a past experience and does not involve positive or negative emotional aspects."}, "language_style": {"answer": "Calm and direct", "rationale": ""}, "time_dimension": {"answer": "Past", "rationale": "The keywords \"when we were kids\" and \"when we were in school for a joke\" indicate that the entry is an inquiry into the testee's past experience."}, "sentence_type": "Affirmative declarative sentence", "pos": {"nouns": {"Time": 1, "When I was little": 1, "Disposition": 1}, "verbs": {"Yes": 1, "- Bullshit": 1}, "numerals": {"25": 1}, "pronouns": {"I": 1}}}, {"content": "26, my body is as healthy as most of my friends.", "word_count": 17, "label": "", "sentence_structure": "Subject鈥揷opula鈥損redicative", "subjective_objective": {"answer": "Objective", "rationale": "Because \"my body is as healthy as most of my friends\" is an objective description of the patient's condition. Although this may be influenced by the patient's self-perception, it is based on observable facts (such as weight, height, health, etc.). So this questionnaire entry is viewed from an objective perspective."}, "sentence_sentiment": {"answer": "Positive", "rationale": "Because the physical state of the entries is healthy."}, "language_style": {"answer": "Calm and direct", "rationale": "The language style of the entry is a calm and straightforward type. It conveys a factual message in a concise and clear language, with no excessive emotional or speech modification, and is sound and objective, and meets the needs of the questionnaire."}, "time_dimension": {"answer": "Present", "rationale": ""}, "sentence_type": "Affirmative declarative sentence", "pos": {"nouns": {"Body": 1, "friends": 1, "Most of them": 1}, "verbs": {"Health": 1}, "adverbs": {"Same thing": 1}, "numerals": {"26": 1}, "pronouns": {"Mine": 2}}}, {"content": "I'll pretend I didn't see any friends who were in school or not, unless they first greeted me.", "word_count": 30, "label": "", "sentence_structure": "Subject鈥搗erb鈥搊bject", "subjective_objective": {"answer": "Subjective", "rationale": "This is a psychological state and behavioural choice that reflects patients ' subjective attitudes and behaviour."}, "sentence_sentiment": {"answer": "Negative", "rationale": "Because the \"fakeless\" act in the entry is negative."}, "language_style": {"answer": "Calm and direct", "rationale": "The language style of the entry is relatively calm and straightforward, and a person鈥檚 behaviour is expressed in a narrative, without excessive emotional or rhetorical style."}, "time_dimension": {"answer": "Past", "rationale": ""}, "sentence_type": "Affirmative declarative sentence", "pos": {"nouns": {"Students": 1, "Common": 1, "friends": 1, "they": 1, "Hello": 1}, "verbs": {"I met him": 1, "Pretend": 1, "I don't see it": 1}, "adjectives": {"Not often": 1}, "adverbs": {"First": 1, "Otherwise": 1, "Just": 1, "Unless": 1}, "numerals": {"27": 1}, "classifiers": {"individual": 1}, "pronouns": {"I": 1}}}, {"content": "I don't read every editorial in the newspaper every day.", "word_count": 16, "label": "", "sentence_structure": "Subject鈥搗erb鈥搊bject", "subjective_objective": {"answer": "Objective", "rationale": "Because \"Do I read every editorial in the newspaper every day\" is an act of the patient at a certain time and is therefore an objective test."}, "sentence_sentiment": {"answer": "Neutral", "rationale": "Because the entry does not have any emotional color."}, "language_style": {"answer": "Calm and direct", "rationale": "The entry is based on an objective and neutral language style, with a preponderance of facts, with no excessive emotional rendering or rhetorical techniques, and is more calm and straightforward."}, "time_dimension": {"answer": "Present", "rationale": ""}, "sentence_type": "Negative declarative sentence", "pos": {"nouns": {"Oh, my God": 1, "Newspapers": 1, "Title": 1, "editorial": 1}, "verbs": {"Look": 1, "have": 1, "Both": 1}, "adverbs": {"Not every day": 1}, "numerals": {"28": 1}, "pronouns": {"I": 1}}}, {"content": "I've never had a normal life.", "word_count": 10, "label": "", "sentence_structure": "Subject鈥損redicate", "subjective_objective": {"answer": "Subjective", "rationale": "Because \"normal life\" is a subjective assessment, different people may define normal life differently. Therefore, it is a subjective view that considers that they have never had a normal life as a personal view and feeling of the patient."}, "sentence_sentiment": {"answer": "Negative", "rationale": "Because \"there's never been a normal life\" expresses a negative emotion and attitude."}, "language_style": {"answer": "Calm and direct", "rationale": "The entry is more calmly and straightforward, not using excessive rhetorical or emotional techniques to express the idea of questioning, but rather in a simple and straightforward way. At the same time, the expression \"never had a normal life\" is also strong and direct, implying that the person under investigation may have experienced difficult or unusual things, which might make the person under investigation uncomfortable or nervous."}, "time_dimension": {"answer": "Present", "rationale": "The entry \"Never\" indicates that there has never been a normal life in the past, while \"now\" refers to the current state, so it can be judged to describe the present behaviour or mentality of the person being tested."}, "sentence_type": "Imperative sentence", "pos": {"nouns": {"Life": 1}, "verbs": {"have": 1, "Pass": 1, "Tsang": 1}, "adjectives": {"Normal": 1}, "adverbs": {"Never": 1}}}, {"content": "I feel like a fire, a sting, a worm crawling, numbing in some parts of my body.", "word_count": 22, "label": "", "sentence_structure": "Subject鈥搗erb鈥揷omplement", "subjective_objective": {"answer": "Subjective", "rationale": "Because these feelings are usually the subjective feelings and experiences of patients."}, "sentence_sentiment": {"answer": "Negative", "rationale": "Because the physical symptoms described in this entry are usually negative."}, "language_style": {"answer": "Tactful and warm", "rationale": "Warm and warm."}, "time_dimension": {"answer": "Past", "rationale": ""}, "sentence_type": "Affirmative declarative sentence", "pos": {"nouns": {"Body": 2, "Part": 1, "Fire": 1, "Stings": 1, "Bug crawling": 1, "I'm numb": 1}, "verbs": {"have": 1, "Elephant": 1, "Feel": 1}, "adjectives": {"Regular": 1}, "numerals": {"30": 1, "Some": 1}, "pronouns": {"I": 1}}}, {"content": "31, I can see around me what others can't see, animals and people.", "word_count": 22, "label": "", "sentence_structure": "Subject鈥搗erb鈥搊bject", "subjective_objective": {"answer": "Subjective", "rationale": "Because it is subjective to see around me what others do not see, animals and people are the senses or views of the patient himself."}, "sentence_sentiment": {"answer": "Neutral", "rationale": "Because the entry does not have a clear emotional tendency or behavioural description. It simply describes the fact that the subject of the investigation can see things that others may not notice, whether animals or humans. Therefore, the emotional color of the purpose is neutral."}, "language_style": {"answer": "Tactful and warm", "rationale": "This entry uses a more euphemistic and guiding language style, asking if the person surveyed has the ability to observe what others may ignore."}, "time_dimension": {"answer": "Present", "rationale": "The entry is to ask the testee whether he has some special ability or perception, and the keyword is \"to see what others cannot see\", and the temporal dimension is now judged."}, "sentence_type": "Affirmative declarative sentence", "pos": {"nouns": {"I": 1, "Something": 1, "Animals": 1, "person": 2}, "verbs": {"I can see it": 1}, "numerals": {"31": 1, "Other": 1}}}, {"content": "I wish I could be as happy as anyone else.", "word_count": 12, "label": "", "sentence_structure": "Subject鈥搗erb鈥搊bject", "subjective_objective": {"answer": "Subjective", "rationale": "Because \"favorite\" is patient's subjective attitude and desire."}, "sentence_sentiment": {"answer": "Neutral", "rationale": "Because the entry is only an expression of the wishes of the interviewees, it is not clearly emotional."}, "language_style": {"answer": "Tactful and warm", "rationale": "By using the word \"hope,\" the entry expresses a desire and expectation, rather than a direct statement of one's state or emotions. This expression is more euphemism and warmer, and more likely to trigger resonance and reaction. At the same time, the tone of the article is more positive, giving the impression of openness and friendship."}, "time_dimension": {"answer": "Present", "rationale": "The entry \"Hope\" expresses the voice of dissatisfaction with the current state and the desire to change it, and \"Happy as others\" is the goal or vision of the future, so the time dimension is now judged."}, "sentence_type": "Imperative sentence", "pos": {"nouns": {"Hope": 1, "I": 1, "others": 1, "Happy": 1}, "verbs": {"Yes": 1, "Yeah": 1, "That way": 1, "Happy": 1}, "numerals": {"32": 1}, "pronouns": {"Well": 1}}}, {"content": "33 I never felt pain behind my neck.", "word_count": 12, "label": "", "sentence_structure": "Subject鈥搗erb鈥揷omplement", "subjective_objective": {"answer": "Objective", "rationale": "Because \"never feeling\" is the patient's subjective feeling and experience, but \"punishment behind the neck\" is the objective situation that the patient may face. So \"I never feel pain behind the neck\" is an objective fact that the patient has seen from the subjective perspective of the patient."}, "sentence_sentiment": {"answer": "Neutral", "rationale": "Because the entry merely stated a fact, and it did not have any emotional colour."}, "language_style": {"answer": "Calm and direct", "rationale": "The language style of the entry is calm and straightforward. It asks the person surveyed whether he or she has ever felt pain in the neck or behind the neck in a simple and clear manner."}, "time_dimension": {"answer": "Present", "rationale": ""}, "sentence_type": "Negative declarative sentence", "pos": {"nouns": {"Neck": 1, "The neck": 1, "Pain": 1}, "verbs": {"I never felt it": 1}, "adverbs": {"Never": 1}, "numerals": {"33": 1}}}, {"content": "I'm upset that I feel uncomfortable every few days or often.", "word_count": 20, "label": "", "sentence_structure": "Subject鈥搗erb鈥搊bject", "subjective_objective": {"answer": "Subjective", "rationale": "Because the feeling of discomfort is the experience and feeling of the patient himself, and it is viewed from a subjective perspective."}, "sentence_sentiment": {"answer": "Negative", "rationale": "Because the \"indiscomfort\" in the entry is negative."}, "language_style": {"answer": "Calm and direct", "rationale": "The language style of the entry is more straightforward, not using too many rhetorical techniques or emotional renderings, but expressing the problem in an objective manner and in a concise language."}, "time_dimension": {"answer": "Present", "rationale": ""}, "sentence_type": "Affirmative declarative sentence", "pos": {"nouns": {"Oh, my God": 1, "Stomach": 1}, "verbs": {"I feel": 1, "Trouble": 1}, "adjectives": {"Heart": 1, "I'm not feeling well": 1}, "adverbs": {"Every": 1, "Regular": 1}, "numerals": {"34": 1, "A few days": 1}}}, {"content": "I'm a big guy.", "word_count": 7, "label": "", "sentence_structure": "Subject鈥揷opula鈥損redicative", "subjective_objective": {"answer": "Subjective", "rationale": "Because \"important\" is a subjective assessment and self-awareness, not about objective facts."}, "sentence_sentiment": {"answer": "Positive", "rationale": "Because the expression \"I'm a key person\" in the entry is positive, with a sense of self-confidence and self-assertion."}, "language_style": {"answer": "Calm and direct", "rationale": "The entry uses a subjective expression, \"I'm a key person\", which can trigger self-awareness conflicts or defensive psychologicalities of the person under investigation, and is not conducive to obtaining real and effective information. Thus, in linguistic style, the purpose is more straightforward and is not expressed too narrowly or vaguely."}, "time_dimension": {"answer": "Present", "rationale": "The entry expresses a self-awareness or self-evaluation, usually related to the state of current thinking and emotion, and thus is judged as \"now\"."}, "sentence_type": "Affirmative declarative sentence", "pos": {"nouns": {"I": 1, "Important people": 1}, "numerals": {"35": 1}}}, {"content": "I get angry sometimes.", "word_count": 5, "label": "", "sentence_structure": "Subject鈥搗erb鈥揷omplement", "subjective_objective": {"answer": "Subjective", "rationale": "Because anger is a emotional reaction of one person, it belongs to the subjective feelings and attitudes of the patient."}, "sentence_sentiment": {"answer": "Negative", "rationale": "Because \"freak\" is a negative emotion."}, "language_style": {"answer": "Tactful and warm", "rationale": "The entry is more adept and interactive when it is spoken in a gentle and inviting manner, rather than by direct statements or orders."}, "time_dimension": {"answer": "Present", "rationale": ""}, "sentence_type": "Affirmative declarative sentence", "pos": {"verbs": {"- Get angry": 1}, "adverbs": {"Time": 1, "Sometimes": 1}}}, {"content": "Recently, I felt it was easy to give up hope for something.", "word_count": 18, "label": "", "sentence_structure": "Subject鈥搗erb鈥揷omplement", "subjective_objective": {"answer": "Subjective", "rationale": "Because \"felt easy to give up\" is a subjective feeling."}, "sentence_sentiment": {"answer": "Negative", "rationale": "Because it expresses a negative emotional attitude, \"easy to give up hope for something\", and a frustration with the future."}, "language_style": {"answer": "Calm and direct", "rationale": "The entry is more straightforward in language style, with no excessive rhetorical or emotional rendering, and is also more objective in tone. It asks whether the respondents have recently found it easy to give up their hopes for certain things, allowing them to express their feelings and thoughts more accurately, thus providing more reliable data support for research."}, "time_dimension": {"answer": "Present", "rationale": "The term \"recently\" in this entry means time limits, which refer to the act or state of the recent occurrence. Thus, the basis of the judgement is \"now\"."}, "sentence_type": "Negative declarative sentence", "pos": {"nouns": {"Recently": 1, "Hope": 1}, "verbs": {"I think": 1, "Easy": 1, "Abandon": 1, "to": 1, "Some": 1, "Things": 1}, "adverbs": {"very": 1}, "numerals": {"37": 1}}}, {"content": "I sometimes get so much attracted to others' things, like shoes, gloves, that I'm not helping me, but I always try to touch them or steal them.", "word_count": 43, "label": "", "sentence_structure": "Subject鈥搗erb鈥搊bject", "subjective_objective": {"answer": "Subjective", "rationale": "Because \"I always want to touch it or steal it\" is the patient's subjective thought and will to act."}, "sentence_sentiment": {"answer": "Negative", "rationale": "Because the entry \"want to touch it or steal it\" is negative."}, "language_style": {"answer": "Tactful and warm", "rationale": "The language style of the entry is euphemism. It does not directly question or accuse the other of misconduct, but rather asks the other in a gentle and understandable way about whether that is the case and provides a subject that can be discussed safely. This expression creates a good atmosphere of communication that allows the other to share their thoughts and feelings."}, "time_dimension": {"answer": "Present", "rationale": "The entry describes the current behaviour and mentality of the subject, \"sometimes\" indicating that this is not normal, but it still happens. \"I'm attracted to something else and I'm attracted to it,\" \"I want to touch it or steal it\" all happens now. So, time dimensions are now judged."}, "sentence_type": "Affirmative declarative sentence", "pos": {"nouns": {"Questionnaire": 1, "Item": 1, "Shoes": 1, "Gloves": 1}, "verbs": {"be": 2, "Attractive": 1, "to": 1, "Yes": 2, "Touch": 1, "Steal": 1}, "adjectives": {"Serious": 1, "Nothing": 1}, "adverbs": {"Sometimes": 1}, "numerals": {"38": 1, "I": 1}, "pronouns": {"it": 2}}}, {"content": "39 I do lack self-confidence.", "word_count": 8, "label": "", "sentence_structure": "Subject鈥搗erb鈥揷omplement", "subjective_objective": {"answer": "Subjective", "rationale": "Because lack of self-confidence is an individual鈥檚 subjective feeling and experience. While this may be influenced to some extent by the external environment, the ultimate decision lies in the individual鈥檚 own perception and assessment of himself."}, "sentence_sentiment": {"answer": "Negative", "rationale": "Because \"lack of confidence\" is a negative emotion and attitude."}, "language_style": {"answer": "Tactful and warm", "rationale": "The entry is more personal and interactive when it is spoken in a question-and-answer tone, rather than directly speaking or ordering. At the same time, by using the word \"verbal\" it makes the person surveyed feel that his or her situation is understood and taken into account. This language style makes it easier for the person under investigation to open his or her heart and to share his or her thoughts and feelings."}, "time_dimension": {"answer": "Past", "rationale": "The \"necessary lack\" in the entry indicates that the respondent, in answering the question, reflected and assessed his past experience and judged it to be the past."}, "sentence_type": "Negative declarative sentence", "pos": {"nouns": {"Self-confidence": 1}, "verbs": {"Lack": 1}, "adjectives": {"Indeed": 1}, "numerals": {"39": 1}}}, {"content": "40, I always feel that life is worth it.", "word_count": 11, "label": "", "sentence_structure": "Subject鈥揷opula鈥損redicative", "subjective_objective": {"answer": "Subjective", "rationale": "This is a patient ' s personal conviction and opinion, which is subjective."}, "sentence_sentiment": {"answer": "Positive", "rationale": "Because it expresses a positive emotional expression of affirmation and confidence in life."}, "language_style": {"answer": "Tactful and warm", "rationale": "The language style of the entry is euphemism. It asks the interviewee about life in a way that encourages and supports it, instead of using direct or command language, but in a gentle way, leads the interviewee to share his thoughts and feelings. This expression is easier to comfort and respect the interviewee, and therefore more willing to participate in the questionnaire."}, "time_dimension": {"answer": "Present", "rationale": "The entry describes the current thinking and attitude of the testee and does not address past or future behaviour or circumstances, and therefore is judged to be \"now\"."}, "sentence_type": "Affirmative declarative sentence", "pos": {"nouns": {"Life": 1, "Value": 1}, "verbs": {"I think": 1}, "adverbs": {"Total": 1}, "numerals": {"40": 1}, "pronouns": {"I": 1}}}, {"content": "41. There is a debate about what is to be done to convince the majority of the truth.", "word_count": 22, "label": "", "sentence_structure": "Subject鈥搗erb鈥搊bject", "subjective_objective": {"answer": "Subjective", "rationale": "Because it's a subjective judgment to think of \"majority\" and \"truth.\""}, "sentence_sentiment": {"answer": "Neutral", "rationale": "Because the entry does not have any emotional color."}, "language_style": {"answer": "Tactful and warm", "rationale": "The entry is more personal and interactive when it is presented in a gentle and inviting tone, rather than in direct statements or orders. At the same time, the sentence does not provide a direct answer to the question of the other party鈥檚 perception of convincing others to accept the view, thus giving the interviewees more freedom to play."}, "time_dimension": {"answer": "Present", "rationale": "The entry describes a general situation or experience, with no clear time-pointing, and should therefore be judged as present behaviour or mentality."}, "sentence_type": "Affirmative declarative sentence", "pos": {"nouns": {"person": 1, "The facts": 1, "The truth": 1, "Debate": 1}, "verbs": {"Yes": 2, "鈾?And I'm gonna be 鈾?: 1, "Marriage": 1, "Letter": 1, "Pass": 1, "The long run": 1, "A little bit": 1}, "adverbs": {"Large": 1}, "numerals": {"41銆?: 1}, "pronouns": {"There is a debate about what is to be done to convince the majority of the truth": 1}}}, {"content": "Sometimes I drag things that should be done today to tomorrow.", "word_count": 17, "label": "", "sentence_structure": "Subject鈥損redicate", "subjective_objective": {"answer": "Subjective", "rationale": "Because this is a patient ' s description and reflection of his or her behaviour, it is a subjective individual feeling."}, "sentence_sentiment": {"answer": "Negative", "rationale": "Because \"delay\" is a negative act."}, "language_style": {"answer": "Calm and direct", "rationale": "This entry describes a behaviour pattern of the person under investigation in a concise and clear language, without excessive rhetoric or emotional rendering."}, "time_dimension": {"answer": "Present", "rationale": ""}, "sentence_type": "Affirmative declarative sentence", "pos": {"nouns": {"Oh, my God": 1, "Yes": 1}, "verbs": {"Do it": 1, "Drag": 1}, "adverbs": {"Sometimes": 1, "The": 1}, "numerals": {"42": 1}, "classifiers": {"individual": 1}}}, {"content": "43 I think most people lie about climbing up.", "word_count": 19, "label": "", "sentence_structure": "Subject鈥搗erb鈥搊bject", "subjective_objective": {"answer": "Subjective", "rationale": "Because \"I think\" means that it's a personal opinion and feeling of the patient, a subjective view."}, "sentence_sentiment": {"answer": "Negative", "rationale": "Because the entry \"for climbing up, lying at all\" is negative."}, "language_style": {"answer": "Calm and direct", "rationale": "The language style of the entry is calm and straightforward. It presents a point of view, which is not too emotional or rhetorical to be interpreted in a more objective and balanced manner."}, "time_dimension": {"answer": "Present", "rationale": "The entry expresses the personal views and opinions of the speaker and does not address past or future conduct or circumstances, and therefore the judgement is now."}, "sentence_type": "Affirmative declarative sentence", "pos": {"nouns": {"person": 2, "Climb": 1}, "verbs": {"I think": 1, "For": 1, "Climb up": 1, "Whatever": 1, "Liar": 1}, "adjectives": {"Most of them": 1}, "adverbs": {"of": 2}, "numerals": {"43": 1}, "pronouns": {"I": 1}}}, {"content": "44- Many things I regret when I do.", "word_count": 13, "label": "", "sentence_structure": "Subject鈥搗erb鈥揷omplement", "subjective_objective": {"answer": "Subjective", "rationale": "Because regret is the patient's subjective feeling and attitude."}, "sentence_sentiment": {"answer": "Negative", "rationale": "Because the entry \"registers after doing it\" is negative."}, "language_style": {"answer": "Tactful and warm", "rationale": "The entry uses a more general social phenomenon as a topic, which is more euphemistic and sweet, without directly expressing strong negative feelings or accusations. At the same time, the words \"doing,\" \"regrets\" are used to describe personal experiences more objectively than in a strongly emotional language. So, I think, its language style is \"precipitous.\""}, "time_dimension": {"answer": "Past", "rationale": ""}, "sentence_type": "Negative declarative sentence", "pos": {"nouns": {"Things": 2, "Regret": 1}, "verbs": {"Do it": 1}, "adjectives": {"Many": 1}, "adverbs": {"Later": 1}, "numerals": {"44": 1}}}, {"content": "I hardly ever had a fight with my family.", "word_count": 12, "label": "", "sentence_structure": "Subject鈥搗erb鈥揷omplement", "subjective_objective": {"answer": "Objective", "rationale": "Because it is an objective fact to argue, not the patient ' s subjective feelings or attitudes."}, "sentence_sentiment": {"answer": "Positive", "rationale": "Because the \"no fight with the family\" act is positive."}, "language_style": {"answer": "Tactful and warm", "rationale": "The entry uses the negative expression \"nearly\" and expresses a more harmonious and gentle tone of relations with the family. Moreover, the entry does not directly ask the other side whether they have had a fight with their family, but instead leads the interviewee to self-evaluation by describing his/her personal relationship with his/her family, which also reflects a warmer language style."}, "time_dimension": {"answer": "Past", "rationale": "\"Nearly\" in the entry is an expression of past behaviour, and the temporal dimension is a past."}, "sentence_type": "Negative declarative sentence", "pos": {"verbs": {"Did you": 1, "Fight": 2}, "adjectives": {"Almost": 1}, "numerals": {"45": 1}, "pronouns": {"I": 1, "Family": 1}}}, {"content": "46 I think the hardest thing is to control myself.", "word_count": 12, "label": "", "sentence_structure": "Subject鈥搗erb鈥搊bject", "subjective_objective": {"answer": "Subjective", "rationale": "Because \"think\" means that it's a subjective view and an assessment of the patient."}, "sentence_sentiment": {"answer": "Negative", "rationale": "Because the entry \"the hardest part is to control myself\" expresses a negative attitude and self-evaluation."}, "language_style": {"answer": "Tactful and warm", "rationale": "The language style of the entry is more euphemistic and gentle, and dialogue is conducted in a way that cares about each other's feelings and understanding of their inner contradictions. By using the expression \"I think\" it avoids too direct or command-style tone, making the atmosphere of the questionnaire more natural. At the same time, the subject \"the hardest thing to control myself\" is more personal and sensitive, and the more soft language style is more conducive to comforting the interviewee and is willing to share his real thoughts and feelings."}, "time_dimension": {"answer": "Present", "rationale": "The \"concept\" entry in this questionnaire indicates a subjective view and attitude, and \"the hardest part is to control myself\" is the current, current state. The entry therefore indicates the current thinking and feeling of the testee, which should be judged as the present."}, "sentence_type": "Affirmative declarative sentence", "pos": {"nouns": {"Questionnaire": 1, "Answer": 1, "Most": 1, "Hard": 1, "be": 1, "I": 1, "self": 1}, "verbs": {"I think": 1, "Control": 1}, "adverbs": {"Most": 1}, "numerals": {"46": 1}, "pronouns": {"I": 1}}}, {"content": "47 I have few muscle cramps or tremors.", "word_count": 14, "label": "", "sentence_structure": "Subject鈥搗erb鈥搊bject", "subjective_objective": {"answer": "Objective", "rationale": "Since the occurrence of muscle cramps or tremors is an objective fact, no subjective judgement by the patient is required."}, "sentence_sentiment": {"answer": "Positive", "rationale": "Because the statement \"very few muscle cramps or tremors\" shows that the respondents are in better health."}, "language_style": {"answer": "Calm and direct", "rationale": "The entry uses a direct presentation to ask the other of the other person whether he has a muscle cramp or a tremor pain, without the use of emotional rendering and speech techniques, so the language style is more calm and straightforward.\""}, "time_dimension": {"answer": "Present", "rationale": ""}, "sentence_type": "Negative declarative sentence", "pos": {"nouns": {"Muscles": 1, "Twitches": 1, "Wreck": 1, "Mistake": 1}, "verbs": {"have": 1, "Very few": 1, "Smoke": 1}, "adjectives": {"Very few": 1}, "adverbs": {"very": 1, "of": 2}, "numerals": {"47": 1}}}, {"content": "I don't seem to care about anything.", "word_count": 12, "label": "", "sentence_structure": "Subject鈥揷opula鈥損redicative", "subjective_objective": {"answer": "Subjective", "rationale": "Because \"not caring\" is a description of subjective attitudes or feelings, not an observation of external facts."}, "sentence_sentiment": {"answer": "Negative", "rationale": "Because the expression \"does not care about anything\" conveys a indifference, lack of motivation and enthusiasm, and is a negative emotional expression."}, "language_style": {"answer": "Calm and direct", "rationale": ""}, "time_dimension": {"answer": "Past", "rationale": "The entry asks the extent to which the testee is indifferent to the matter, and the time dimension does not clearly point to past, present or future, and therefore cannot accurately be categorized as past, present or future behaviour or mentality."}, "sentence_type": "Negative declarative sentence", "pos": {"nouns": {"Things": 1}, "verbs": {"I care": 1, "Seems like": 1}, "adjectives": {"Both": 1}, "numerals": {"48": 1}, "pronouns": {"I": 1}}}, {"content": "49, when I'm not feeling well, I get angry sometimes.", "word_count": 15, "label": "", "sentence_structure": "Subject鈥損redicate", "subjective_objective": {"answer": "Subjective", "rationale": "Because \"I sometimes have a temper\" is the patient's subjective feeling and behavior. Although this may be influenced by the health condition, in this case, the condition does not directly determine whether the patient is going to be angry, but rather is a potential factor for emotional reaction. So, \"I sometimes have a temper\" is the patient's subjective attitude and behaviour."}, "sentence_sentiment": {"answer": "Negative", "rationale": "Because \"crazy\" is usually considered a negative behavior or emotional reaction."}, "language_style": {"answer": "Tactful and warm", "rationale": "The language style of this entry is more gentle and friendly, with the first person calling me the entry point, making it easier for the interviewee to resonate. And \"sometimes\" also suggests that this is not always happening, and that it is more humane and understanding."}, "time_dimension": {"answer": "Past", "rationale": "The entry \"when I'm not feeling well\" indicates something or experience of the past, and \"time-tempered\" indicates something that has already happened. So the dimension of time is the past."}, "sentence_type": "Affirmative declarative sentence", "pos": {"nouns": {"Body": 1, "Time": 1, "- Grumpy": 1}, "verbs": {"I": 2, "Feel": 1, "Let's go": 1, "Sometimes": 1}, "adverbs": {"I'm not feeling well": 1, "Sometimes": 1}, "numerals": {"49": 1, "1": 3}, "pronouns": {"I": 2}}}, {"content": "I always feel like I've done something wrong or something.", "word_count": 21, "label": "", "sentence_structure": "Subject鈥損redicate", "subjective_objective": {"answer": "Subjective", "rationale": "Because it's a self-perception and subjective attitude of the patient."}, "sentence_sentiment": {"answer": "Negative", "rationale": "Because \"thinking you've done something wrong or committed a crime\" is a self-repeated and derogatory emotion, which is a negative emotional color."}, "language_style": {"answer": "Calm and direct", "rationale": "The entry is directly asked, without excessive rhetorical or emotional rendering, and is more objective in tone."}, "time_dimension": {"answer": "Present", "rationale": "\"Thousands\" means \"required\" as a regular, customary act, while \"felt\" as a general state of affairs means that the act has not only occurred in the past but may still be ongoing. Thus, the entry is for the purpose of questioning the person being tested."}, "sentence_type": "Negative declarative sentence", "pos": {"nouns": {"Yes": 2, "Sin": 2}, "verbs": {"Do it": 1, "Yes": 1, "I always thought": 1}, "adjectives": {"self": 2, "It looks like": 1}, "adverbs": {"Always": 1}, "numerals": {"50": 1}}}, {"content": "I'm always happy.", "word_count": 7, "label": "", "sentence_structure": "Subject鈥揷opula鈥損redicative", "subjective_objective": {"answer": "Subjective", "rationale": "Because \"happy\" is a subjective emotional feeling that cannot be judged by objective observations, it depends on the patient's self-reporting or description."}, "sentence_sentiment": {"answer": "Positive", "rationale": "Because the entry \"often happy\" expresses a positive emotional attitude."}, "language_style": {"answer": "Tactful and warm", "rationale": "The words \"often\" and \"happy\" are used in a more euphemistic way, without asking or ordering each other directly to be happy. This expression makes people feel more friendly and friendly."}, "time_dimension": {"answer": "Present", "rationale": "Now the entry uses the term \"often\" to indicate that it is a customary act, and the temporal dimension is now. At the same time, the sentence is about the person who was tested, his own feelings and attitudes, and does not address past or future situations. Thus, the basis of the judgement is \"now\"."}, "sentence_type": "Affirmative declarative sentence", "pos": {"verbs": {"be": 1, "Regular": 1, "Happy": 1}, "adjectives": {"Happy": 1}, "numerals": {"51": 1}}}, {"content": "52, I often feel like my head is swollen.", "word_count": 11, "label": "", "sentence_structure": "Subject鈥揷opula鈥損redicative", "subjective_objective": {"answer": "Subjective", "rationale": "Because this perception is usually the patient鈥檚 subjective experience and feeling. While it may be related to objective factors (e.g. allergic reactions), it is an important information that questionnaire researchers want to understand."}, "sentence_sentiment": {"answer": "Negative", "rationale": "Because the physical symptoms of \"head-breathing\" in the entry are negative."}, "language_style": {"answer": "Calm and direct", "rationale": "The entry describes the symptoms of the person under investigation in a concise and clear manner, without too many rhetorical or emotional renderings, and with a more calm and objective expression."}, "time_dimension": {"answer": "Present", "rationale": ""}, "sentence_type": "Affirmative declarative sentence", "pos": {"nouns": {"Head": 1, "Nose plug": 1}, "verbs": {"I think": 1, "Boo": 1, "Like": 1}, "adjectives": {"Regular": 1, "Time": 1}, "numerals": {"52": 1}}}, {"content": "And of those who are too arrogant, even though I know that they are right, I will do it with them.", "word_count": 24, "label": "", "sentence_structure": "Subject鈥損redicate", "subjective_objective": {"answer": "Subjective", "rationale": "This is because of the patient ' s personal feelings and attitudes and is subjective."}, "sentence_sentiment": {"answer": "Negative", "rationale": "The judgement is based on \"satisfaction to others' hegemonic behaviour and adherence to their positions\", which is a negative attitude."}, "language_style": {"answer": "Tactful and warm", "rationale": ""}, "time_dimension": {"answer": "Present", "rationale": "The entry describes the current behaviour or mentality of the testee, and \"some of the people are too arrogant\" suggests that it is the testee's view of the current social phenomenon, and that \"even if I know they're right, I want to fight them\" is the reaction or response of the testee. This information points to the current situation."}, "sentence_type": "Affirmative declarative sentence", "pos": {"nouns": {"person": 2, "Hero": 1, "Ming": 1, "to": 1, "they": 2, "Fuck": 1}, "verbs": {"Too much": 1, "be": 1, "Even if": 1, "I know": 1, "Yes": 1, "and": 1, "Right": 1}, "adjectives": {"Hero": 1}, "adverbs": {"Too much": 1, "Even if": 1}, "numerals": {"53": 1, "2": 2, "1": 8}, "pronouns": {"I": 1}}}, {"content": "54, someone tried to hurt me.", "word_count": 5, "label": "", "sentence_structure": "Subject鈥搗erb鈥搊bject", "subjective_objective": {"answer": "Subjective", "rationale": "Because \"think\" is a subjective idea or perception, which reflects the patient鈥檚 inner feelings and perceptions, not an objective fact. Although there may be some real threat or danger, the idea itself is the patient鈥檚 personal experience and judgment."}, "sentence_sentiment": {"answer": "Negative", "rationale": "Because the expression \"want to hurt me\" in the entry is negative."}, "language_style": {"answer": "Tactful and warm", "rationale": "The entry is more personal and interactive when it is spoken in a gentle, inquisitive manner, rather than in direct statements or orders. At the same time, it contains some emotional colours (i.e., anxiety and fear), but it is not strong, and more so it is a euphemism to direct the interviewee to think and answer questions."}, "time_dimension": {"answer": "Present", "rationale": "The entry in the future describes the vision and apprehension of the testee about a possibility that may arise in the future, the keyword being \"someone wants to harm me\", pointing to a future that has not yet happened."}, "sentence_type": "Affirmative declarative sentence", "pos": {"nouns": {"person": 1, "Shit": 1}, "verbs": {"Yes": 1, "Shit": 1}, "numerals": {"54": 1}}}, {"content": "55 I often believe that it is necessary to insist on what I think is right.", "word_count": 19, "label": "", "sentence_structure": "Subject鈥搗erb鈥搊bject", "subjective_objective": {"answer": "Subjective", "rationale": "Because the idea of \"think\" and \"maintain\" is patiently subjective."}, "sentence_sentiment": {"answer": "Positive", "rationale": "Because the \"Standing to the Right\" act in the entry expresses a positive attitude and quality."}, "language_style": {"answer": "Calm and direct", "rationale": "The language style of the entry is more straightforward, not using excessive rhetorical techniques or emotional rendering, but rather expressing in an objective and clear manner whether the subject matter of what they believe is often felt to be necessary."}, "time_dimension": {"answer": "Past", "rationale": ""}, "sentence_type": "Affirmative declarative sentence", "pos": {"nouns": {"I": 2, "Yes": 1}, "verbs": {"I always think": 1, "Hold on": 1, "be": 1, "I think": 1, "I have to": 1, "Do it": 1}, "adjectives": {"Those": 1, "Correct": 1}, "adverbs": {"Often": 1, "I have to": 1}, "numerals": {"55": 1, "2": 1}}}, {"content": "I often feel like I have a tight belt on my head.", "word_count": 18, "label": "", "sentence_structure": "Subject鈥揷opula鈥損redicative", "subjective_objective": {"answer": "Subjective", "rationale": "Because \"often feeling\" describes a subjective feeling or experience of the patient, the view of this questionnaire entry is subjective."}, "sentence_sentiment": {"answer": "Negative", "rationale": "Because the expression \"like a tight tape on the head\" has a negative emotional color."}, "language_style": {"answer": "Calm and direct", "rationale": "The entry uses simple statements, without too much rhetorical or emotional rendering, and is more balanced and objective. This language style is more in line with the language requirements of the psychological questionnaire and allows the interviewee to reflect his/her feelings and thoughts more honestly and accurately."}, "time_dimension": {"answer": "Past", "rationale": "The entry, therefore, relates to the current emotional state of the testee and should be classified as the present."}, "sentence_type": "Affirmative declarative sentence", "pos": {"nouns": {"Head": 1, "One": 1, "The tape": 1}, "verbs": {"I think": 1, "have": 1, "Band-Aids": 1}, "adjectives": {"Tighter": 1}, "numerals": {"56": 1}}}, {"content": "57. I was in school and sometimes called by the leadership for the nonsense.", "word_count": 18, "label": "", "sentence_structure": "Subject鈥搗erb鈥搊bject", "subjective_objective": {"answer": "Subjective", "rationale": "The author also claims that the author's death was due to the fact that he was not a victim of a crime, because this statement reflects the patient's subjective experience and feelings."}, "sentence_sentiment": {"answer": "Negative", "rationale": "Because the \"fun\" in the entries is negative."}, "language_style": {"answer": "Calm and direct", "rationale": "The language style of the entry is a relatively calm and straightforward type. It refers explicitly to the specific circumstances and reasons for being called by the leader, without adding too many rhetorical or emotional colours and speaking in a balanced and objective manner. This way helps the interviewee to understand the questions more accurately, clearly and provide a true answer."}, "time_dimension": {"answer": "Past", "rationale": ""}, "sentence_type": "Affirmative declarative sentence", "pos": {"nouns": {"I": 1, "Time": 1, "School": 1, "Leadership": 1}, "verbs": {"in": 1, "have": 1, "Call": 1}, "adverbs": {"Sometimes": 1, "Because": 1, "And": 1}, "numerals": {"57": 1}, "classifiers": {"individual": 1}}}, {"content": "I'm out eating with friends when I'm a lot more disciplined than at home.", "word_count": 22, "label": "", "sentence_structure": "Subject鈥搗erb鈥搊bject", "subjective_objective": {"answer": "Subjective", "rationale": "Because this is the subjective feeling and comparison of the patient, it is subjective."}, "sentence_sentiment": {"answer": "Positive", "rationale": "Because the behavior in the entries is \"more positive than at home when eating with friends outside.\""}, "language_style": {"answer": "Tactful and warm", "rationale": "The language style of the entry is a soft and warmer category. The article is expressed in a more moderate way, without a direct tone of criticism or command, but rather in a way that describes a phenomenon and raises a question, which is more likely to attract resonance and participation from the person under investigation. At the same time, the comparative phrase \"more discipline than at home\" reflects a kind of accompaniment and interaction."}, "time_dimension": {"answer": "Past", "rationale": "The entry describes past experiences or habits, \"when eating with friends outside\" as a picture of past events, \"more discipline than at home\" as a representation of customary behaviour compared to present or future."}, "sentence_type": "Affirmative declarative sentence", "pos": {"nouns": {"Outside": 1, "friends": 1, "Rice": 1, "Home": 1}, "verbs": {"in": 2, "Eat": 1, "Rule": 1, "More": 1}, "adjectives": {"Rules": 1}, "adverbs": {"very": 1}, "numerals": {"58": 1}, "pronouns": {"I": 1}}}, {"content": "59 I believe someone is behind me.", "word_count": 8, "label": "", "sentence_structure": "Subject鈥搗erb鈥搊bject", "subjective_objective": {"answer": "Subjective", "rationale": "Because belief is subjective."}, "sentence_sentiment": {"answer": "Negative", "rationale": "Because the \"concealed that someone is behind me\" attitude in the entry is negative."}, "language_style": {"answer": "Calm and direct", "rationale": "The basis for the judgement: the language of the article is simple and clear, and there is no overly embellished term or emotional rendering, but simply a point or belief, and therefore a calm and straightforward language style."}, "time_dimension": {"answer": "Future", "rationale": "The keyword \"convince\" and \"implicit\" and the time-forming of the sentence point to what might happen in the future, so the entry is for the future."}, "sentence_type": "Affirmative declarative sentence", "pos": {"nouns": {"person": 1, "Undercount": 1}, "verbs": {"Yes": 1, "Undercount": 1}, "adverbs": {"I": 1}, "numerals": {"59": 1}}}, {"content": "I'm as smart as the people around me.", "word_count": 15, "label": "", "sentence_structure": "Subject鈥揷opula鈥損redicative", "subjective_objective": {"answer": "Subjective", "rationale": "Because \"it seems\" indicates a feeling or a perception, a subjective attitude of the patient."}, "sentence_sentiment": {"answer": "Neutral", "rationale": "Because the entry does not show positive or negative emotional colours. It simply describes the feeling of a person who feels as smart as the people around him."}, "language_style": {"answer": "Tactful and warm", "rationale": "The entry uses a more euphemistic and modest way of expressing its views, avoiding a direct assertion of the superiority of one鈥檚 abilities or of a superior position to others. Such language styles are more reflective of a modest and low-key attitude and more resonant and supportive."}, "time_dimension": {"answer": "Present", "rationale": ""}, "sentence_type": "Affirmative declarative sentence", "pos": {"nouns": {"person": 1, "Questionnaire": 1, "Entry": 1}, "verbs": {"Seems like": 1, "and": 1, "Same thing": 1, "Smart and capable": 1}, "adjectives": {"Smart and capable": 1}, "adverbs": {"Seems like": 1}, "numerals": {"60": 1}, "pronouns": {"I": 1}}}, {"content": "61, I believe someone's following me.", "word_count": 9, "label": "", "sentence_structure": "Subject鈥搗erb鈥搊bject", "subjective_objective": {"answer": "Subjective", "rationale": "It is believed that the patient's subjective attitude."}, "sentence_sentiment": {"answer": "Negative", "rationale": "Because the idea of \"convincing someone following me\" in the entry is negative."}, "language_style": {"answer": "Tactful and warm", "rationale": "The entry opens in a question-and-answer manner and uses the word \"convincing\" to give a more moderate and unaccused tone, which is more in line with the language style of the psychological questionnaire. At the same time, the entry does not directly indicate whether the other party is actually being followed, but rather gives the interviewee a space to respond to it on his own, which is also a sign of a warmer language style."}, "time_dimension": {"answer": "Past", "rationale": ""}, "sentence_type": "Affirmative declarative sentence", "pos": {"nouns": {"person": 1, "Track": 1}, "verbs": {"Yes": 1, "in": 1}, "adverbs": {"Both": 1}, "numerals": {"61": 1}, "pronouns": {"I": 1}}}, {"content": "62. Most people do not want to lose opportunities by improper means to gain.", "word_count": 24, "label": "", "sentence_structure": "Subject鈥搗erb鈥搊bject", "subjective_objective": {"answer": "Subjective", "rationale": "Because the sentence concerns subjective judgments and choices about morality and ethics. While one may consider such behaviour to be unjustified, they may still consider using these means to gain benefits in certain circumstances."}, "sentence_sentiment": {"answer": "Negative", "rationale": "Because the actions in the entries are not justified, there is a clear negative tendency."}, "language_style": {"answer": "Tactful and warm", "rationale": "This is a more moderate and open way of expressing the view of the respondents that most people are using inappropriate means to gain their own interests, while expressing an attitude towards opportunity."}, "time_dimension": {"answer": "Past", "rationale": "The entry describes a general phenomenon or attitude in which \"most\" refers to a group, while \"old\" implies that this phenomenon has existed or occurred in the past."}, "sentence_type": "Affirmative declarative sentence", "pos": {"nouns": {"person": 1, "Means": 1, "Interest": 1, "Opportunities": 1}, "verbs": {"Whatever": 1, "- Getting it": 1, "Lose": 1}, "adjectives": {"It's not fair": 1, "Most of them": 1}, "adverbs": {"And": 2}, "numerals": {"62": 1}}}, {"content": "63, my stomach is a lot of trouble.", "word_count": 8, "label": "", "sentence_structure": "Subject鈥揷opula鈥損redicative", "subjective_objective": {"answer": "Objective", "rationale": "Because it is an objective fact whether the patient has a stomach condition or not, the patient ' s knowledge and perception of his or her condition may be subjective."}, "sentence_sentiment": {"answer": "Negative", "rationale": "Because the expression \"my stomach has a lot of problems\" is negative."}, "language_style": {"answer": "Tactful and warm", "rationale": "Warm and warm."}, "time_dimension": {"answer": "Past", "rationale": "In the entry, \"I have a lot of stomach problems\" means a past health condition or disease experience, so the temporal dimension is a past."}, "sentence_type": "Affirmative declarative sentence", "pos": {"nouns": {"Stomach": 1, "Diseases": 1}, "verbs": {"have": 1}, "adjectives": {"More": 1}, "numerals": {"63": 1}}}, {"content": "64, I know who caused my troubles.", "word_count": 12, "label": "", "sentence_structure": "Subject鈥搗erb鈥搊bject", "subjective_objective": {"answer": "Subjective", "rationale": "Because knowing, knowing, who causes the trouble is the patient's subjective perception and feelings, not the objective facts."}, "sentence_sentiment": {"answer": "Neutral", "rationale": "Because the entry does not have any emotional color."}, "language_style": {"answer": "Tactful and warm", "rationale": "This topic uses a more moderate and inviting tone, rather than a direct statement or order, which is more personal and interactive. At the same time, the issue gives the respondents a free expression of their views in an open way, giving them enough respect and trust."}, "time_dimension": {"answer": "Present", "rationale": "The entry is an inquiry into the testee ' s perception of the cause of the distress, and the temporal dimension is now judged."}, "sentence_type": "Affirmative declarative sentence", "pos": {"nouns": {"Mine": 1, "Trouble": 1, "Who": 1, "Cause": 1}, "verbs": {"Yes": 1, "be": 1}, "numerals": {"64": 1}}}, {"content": "I'm not worried about getting sick.", "word_count": 8, "label": "", "sentence_structure": "Subject鈥搗erb鈥揷omplement", "subjective_objective": {"answer": "Subjective", "rationale": "Because \"no worries\" is a subjective feeling and attitude."}, "sentence_sentiment": {"answer": "Positive", "rationale": "Because the entry \"no fear of disease\" expresses a positive mentality."}, "language_style": {"answer": "Tactful and warm", "rationale": "The entry uses a negative sentence, and does not directly express a fear of illness, but rather a more relaxed attitude. This language style makes people feel comfortable and friendly, and makes them more resonant and responsive to the respondents."}, "time_dimension": {"answer": "Past", "rationale": "The keywords \"illness\" and \"fear\" indicate that the entry asks if the person tested had in the past reacted to the mood of fear or anxiety about the disease, and therefore the temporal dimension is a matter of the past."}, "sentence_type": "Negative declarative sentence", "pos": {"nouns": {"Diseases": 1}, "verbs": {"Okay": 1, "I'm worried": 1}, "adverbs": {"not": 1}, "numerals": {"65": 1}}}, {"content": "Sometimes my mind runs too fast to express itself.", "word_count": 18, "label": "", "sentence_structure": "Subject鈥搗erb鈥揷omplement", "subjective_objective": {"answer": "Subjective", "rationale": "Because \"thinking too fast\" is a psychological experience or experience, a subjective perception and a sense of the patient."}, "sentence_sentiment": {"answer": "Negative", "rationale": "Because the description of \"too fast to express\" is negative."}, "language_style": {"answer": "Tactful and warm", "rationale": "The language style of the entry is more euphemismal. It does not ask the other side directly whether there is a problem of expression, but rather raises it in an implicit and mild way, giving people a comfort. Moreover, it is more concise and understandable."}, "time_dimension": {"answer": "Present", "rationale": "\"Sometimes\" means that this is something that happens at some point, and it is not clear whether it is the past or the future, but the descriptions of \"thinks too fast\" and \"fast to express\" are both current, so we can judge that the entry is primarily asking about the situation."}, "sentence_type": "Affirmative declarative sentence", "pos": {"nouns": {"Time": 1, "Ideas": 1, "Run": 1, "Expression": 1, "Come out": 1}, "verbs": {"Too soon": 1, "It's too late": 1, "Expression": 1}, "adjectives": {"Come on": 1, "It's too late": 1}, "adverbs": {"Sometimes": 1, "Both": 1}, "numerals": {"66": 1}}}, {"content": "67 If people treat me well, I often doubt that they have any ulterior motives.", "word_count": 18, "label": "", "sentence_structure": "Subject鈥損redicate", "subjective_objective": {"answer": "Subjective", "rationale": "Because suspicion is a subjective feeling and judgement, it is not an objective fact."}, "sentence_sentiment": {"answer": "Negative", "rationale": "Because the attitude of \"suspecting someone for an unintentional purpose\" in the entry is negative."}, "language_style": {"answer": "Tactful and warm", "rationale": "The reference to the questionnaire was based on: 1. The reference to a relatively mild tone, which did not directly deny or accuse the other party; and 2. The term \"often\" was used to indicate that the doubt was not always present, but rather was of a temporal nature, neutral and objective."}, "time_dimension": {"answer": "Present", "rationale": "\"If others treat me well, I often doubt that they have any ulterior motives.\" This questionnaire entry concerns the interpretation and interpretation of the behaviour of others. So, in terms of time dimension, it can be classified as the current state of affairs, because it describes the current situation or the customary reaction. At the same time, it also contains past experience or memory (i.e., \"I often doubt that they have any ulterior motives\"), but it does not affect the behaviour or attitude that we judge them as the present. So the output is: \"Now\"."}, "sentence_type": "Negative declarative sentence", "pos": {"nouns": {"67": 1, "person": 1, "Heart": 1}, "verbs": {"- Wait": 1, "Regular": 1, "Doubt": 1, "have": 1}, "adjectives": {"Okay": 1, "Don't be mean": 1}, "adverbs": {"Often": 1}, "pronouns": {"I": 1}}}, {"content": "I sometimes feel like I have to hurt myself or someone else.", "word_count": 17, "label": "", "sentence_structure": "Subject鈥搗erb鈥揷omplement", "subjective_objective": {"answer": "Subjective", "rationale": "Because \"I feel like I have to hurt myself or someone else\" is the patient's subjective feeling and idea."}, "sentence_sentiment": {"answer": "Negative", "rationale": "Because the action in the entry is \"harm yourself or someone else,\" it's a negative act."}, "language_style": {"answer": "Calm and direct", "rationale": "This language style is usually more common in psychological questionnaires, because it conveys information in a concise and clear manner and avoids misunderstanding."}, "time_dimension": {"answer": "Past", "rationale": "The words \"like\" and \"like\" in the entry indicate that it is the feeling or thought of the person being tested, and that there is no clear time point. It is therefore not possible to determine whether the past, present or future behaviour or mentality is present. But, since these words are usually used to describe subjective experiences, we can infer that the question is asking about past experiences or feelings."}, "sentence_type": "Affirmative declarative sentence", "pos": {"nouns": {"Questionnaire": 1, "Entry": 1, "I": 2, "self": 1, "others": 1}, "verbs": {"I think": 1, "I have to": 1, "Hurt": 1, "It's like": 1}, "adverbs": {"Sometimes": 1}, "numerals": {"68": 1}}}, {"content": "I'm not sure I'm useful at all.", "word_count": 12, "label": "", "sentence_structure": "Subject鈥揷opula鈥損redicative", "subjective_objective": {"answer": "Subjective", "rationale": "Because \"I think\" means that it's a patient's subjective feeling and self-evaluation, not an objective fact or result."}, "sentence_sentiment": {"answer": "Negative", "rationale": "Because the term \"no use\" is negative, and it is a sign of self-denial and negative sentiment."}, "language_style": {"answer": "Tactful and warm", "rationale": "The entry raises questions in a concerned way, trying to understand what the person is feeling in the person in question, rather than directly accusing or denying. This expression is easier to open up to the person in question and more conducive to the investigators gathering real and effective information."}, "time_dimension": {"answer": "Present", "rationale": "Sometimes it's \"sometimes\" that means \"sometimes\" that the frequency is not certain, while \"feel\" that takes the normal current state of affairs means that the act has not only occurred in the past, but may still be ongoing. So the entry to the questionnaire describes the current state of behaviour or mental state."}, "sentence_type": "Interrogative sentence", "pos": {"nouns": {"69": 1, "Time": 1, "I": 2, "Feel": 1, "It's useless": 1}, "verbs": {"I think": 1, "Really": 1, "be": 1}, "adjectives": {"It's useless": 1}, "adverbs": {"Sometimes": 1}}}, {"content": "70, sometimes I want to fight for reasons.", "word_count": 11, "label": "", "sentence_structure": "Subject鈥搗erb鈥搊bject", "subjective_objective": {"answer": "Subjective", "rationale": "Because the word \"want\" means it's a subjective wish or idea."}, "sentence_sentiment": {"answer": "Negative", "rationale": "Because the \"fight with someone else\" act is offensive and negative."}, "language_style": {"answer": "Tactful and warm", "rationale": "The language style of the entry is more euphemistic and caring, and it does not directly ask whether there are frequent attempts to fight, but rather to guide the interviewee to express his feelings in a way that is understanding and supportive."}, "time_dimension": {"answer": "Past", "rationale": "Past/Now"}, "sentence_type": "Imperative sentence", "pos": {"verbs": {"Yes": 1, "I'm sorry": 1, "Fight": 1, "Array": 1}, "adverbs": {"Sometimes": 1}, "numerals": {"70": 1}}}, {"content": "I like to wander around. If I don't, I'm not happy.", "word_count": 16, "label": "", "sentence_structure": "Subject鈥搗erb鈥搊bject", "subjective_objective": {"answer": "Subjective", "rationale": "Because \"likes to wander\" and \"unsatisfied\" are the feelings and emotional experiences of the patient, and the subjective attitudes of the patient."}, "sentence_sentiment": {"answer": "Negative", "rationale": "Because the \"I'm not happy if I don't hang out\" attitude in the entry is negative."}, "language_style": {"answer": "Tactful and warm", "rationale": "The entry is more gentle and friendly by asking each other's preferences, rather than by direct statements or orders. And, \"I'm not happy if I don't get it,\" is also a euphemism, with no overly strong or negative emotional expression."}, "time_dimension": {"answer": "Present", "rationale": "So the time dimension of this entry is judged now."}, "sentence_type": "Exclamatory sentence", "pos": {"nouns": {"I like it": 1, "Everywhere": 1, "I'm just hanging around": 1, "If": 1, "No way": 1, "I": 2, "I'm not happy": 1}, "verbs": {"Yeah": 1, "- Go on": 1, "Okay": 1, "I'm not happy": 1}, "adverbs": {"Everywhere": 1, "Chaos": 1, "If": 1}, "pronouns": {"I": 2}}}, {"content": "72, someone's been trying to poison me.", "word_count": 8, "label": "", "sentence_structure": "Subject鈥搗erb鈥搊bject", "subjective_objective": {"answer": "Subjective", "rationale": "Because \"someone's been trying to poison me\" is a subjective idea or belief, not an objective fact."}, "sentence_sentiment": {"answer": "Negative", "rationale": "Because the \"sintent to poison\" in the entry is negative."}, "language_style": {"answer": "Tactful and warm", "rationale": "The language style of the entry is relatively cold, with the strong emotional term \"toxication\" used to describe the behaviour of the other, and not in a euphemism or indulging expression, giving a more serious and tense atmosphere."}, "time_dimension": {"answer": "Past", "rationale": "The entry is for the purpose of asking if the person tested had experienced a situation where someone wanted to poison him, and the time dimension was judged to be past."}, "sentence_type": "Affirmative declarative sentence", "pos": {"nouns": {"person": 1, "Poison": 1, "I": 1}, "verbs": {"Yes": 1, "Poison": 1}, "adverbs": {"Always": 1}, "numerals": {"72": 1}}}, {"content": "73, most of the years, I've been fine.", "word_count": 16, "label": "", "sentence_structure": "Subject鈥搗erb鈥揷omplement", "subjective_objective": {"answer": "Objective", "rationale": "Because health is an objective fact of the patient's experience."}, "sentence_sentiment": {"answer": "Positive", "rationale": "Because the entry describes better health and is a positive description."}, "language_style": {"answer": "Tactful and warm", "rationale": "The language style of the entry is euphemism. It is not a matter of asking the other person about his or her physical condition, but of speaking in a more positive and encouraging way, and is more caring."}, "time_dimension": {"answer": "Past", "rationale": ""}, "sentence_type": "Affirmative declarative sentence", "pos": {"nouns": {"Years": 1, "Time": 1, "Body": 1}, "verbs": {"Both": 1, "Okay": 1}, "adjectives": {"Large": 1, "Part": 1}, "adverbs": {"Come on": 1, "Near": 1}, "numerals": {"73": 1}}}, {"content": "74, I've never had a convulsion problem.", "word_count": 10, "label": "", "sentence_structure": "Subject鈥搗erb鈥揷omplement", "subjective_objective": {"answer": "Subjective", "rationale": "The answer to this questionnaire is therefore subjective."}, "sentence_sentiment": {"answer": "Neutral", "rationale": "Because the entry merely describes a fact and does not express any emotional colour."}, "language_style": {"answer": "Calm and direct", "rationale": "The entry is presented in a negative manner and is not accompanied by too many qualifiers or emotional colours, which are more calm and straightforward."}, "time_dimension": {"answer": "Present", "rationale": ""}, "sentence_type": "Negative declarative sentence", "pos": {"nouns": {"Problem": 1, "Twitches": 1}, "verbs": {"Never": 1, "have": 1}, "adjectives": {"Mistake": 1}, "numerals": {"74": 1}, "pronouns": {"I": 1}}}, {"content": "75 I feel that I am often punished without any reason.", "word_count": 15, "label": "", "sentence_structure": "Subject鈥揷opula鈥損redicative", "subjective_objective": {"answer": "Subjective", "rationale": "Because I think it's about the patient's own subjective feelings and opinions."}, "sentence_sentiment": {"answer": "Negative", "rationale": "Because the entry \"often and without justification\" expresses negative emotions and experiences."}, "language_style": {"answer": "Calm and direct", "rationale": "The entry directly asks whether the other party has been subjected to frequent and unjustified punishment, is simple in language style, and does not have too many rhetorical or emotional colours. It therefore determines that the language style is \"cool and direct\"."}, "time_dimension": {"answer": "Past", "rationale": ""}, "sentence_type": "Negative declarative sentence", "pos": {"nouns": {"I think": 1, "I'm always": 1, "For no reason": 1, "Punishment": 1}, "verbs": {"I think": 1, "Often": 1, "Received": 1, "Punishment": 1}, "adjectives": {"For no reason": 1}, "numerals": {"75": 1}, "pronouns": {"I": 2}}}, {"content": "76, I'm easy to cry.", "word_count": 4, "label": "", "sentence_structure": "Subject鈥揷opula鈥損redicative", "subjective_objective": {"answer": "Subjective", "rationale": "Because \"cry\" is a patient's self-perception and experience, a subjective description."}, "sentence_sentiment": {"answer": "Negative", "rationale": "Because \"Easy to cry\" is negative."}, "language_style": {"answer": "Tactful and warm", "rationale": "The entry is in a milder language, not directly revealing the weaknesses or shortcomings of the interviewee, but rather raising questions in a caring manner, which makes it easier for the interviewee to relax and share his real feelings. At the same time, the sentence is also emotional, expressing concern and understanding of the interviewee and more natural."}, "time_dimension": {"answer": "Past", "rationale": ""}, "sentence_type": "Affirmative declarative sentence", "pos": {"verbs": {"Cry": 1}, "adjectives": {"Easy": 1}, "numerals": {"76": 1}}}, {"content": "77, I can't understand what I read as I did before.", "word_count": 17, "label": "", "sentence_structure": "Subject鈥搗erb鈥揷omplement", "subjective_objective": {"answer": "Subjective", "rationale": "Because the subject of \"I\" cannot understand what the book reads is a subjective feeling and experience."}, "sentence_sentiment": {"answer": "Negative", "rationale": "Because the entry expresses a sense of reduced competence, which may involve learning, reading, etc., and is negative."}, "language_style": {"answer": "Tactful and warm", "rationale": "According to the sentence, it is not a question of direct accusations or criticism of the interviewees, but rather a more moderate way of asking questions. Moreover, there is no strong negative emotional vocabulary in the sentence, such as \"no\" or \"no\" but an objective, peaceful expression of meaning, which is consistent with the euphemism of the language style."}, "time_dimension": {"answer": "Past", "rationale": "\"I cannot say that this sentence describes past experiences and behaviour, as I did before, and therefore judge it as \"the past\"."}, "sentence_type": "Negative declarative sentence", "pos": {"nouns": {"I": 2, "Once upon a time": 1, "Something": 1}, "verbs": {"No, I can't": 1, "Understood": 1, "Read it": 1}, "adjectives": {"Elephant": 1, "That way": 1, "Sou-san": 1, "Yes": 1}, "numerals": {"77": 1}}}, {"content": "78, sometimes I feel like my head hurts when it hits.", "word_count": 14, "label": "", "sentence_structure": "Subject鈥揷opula鈥損redicative", "subjective_objective": {"answer": "Subjective", "rationale": "Because it's the patient's subjective feeling, \"sometimes I feel\" , not the objective fact."}, "sentence_sentiment": {"answer": "Negative", "rationale": "Because the description of \"a bump on the top of the head\" in the entry is a negative expression of physical discomfort."}, "language_style": {"answer": "Calm and direct", "rationale": "The language style of the entry is relatively calm and straightforward, rather than using excessive rhetoric or emotional expressions, the question is presented in an objective, concise manner that makes it easier for the interviewees to understand and respond."}, "time_dimension": {"answer": "Present", "rationale": "The keyword \"sometimes feel\" indicates that it's a sentence describing the current feeling, that \"the pain upon the head\" is a subjective feeling and experience that happens to the person who talks. So the time dimension of this questionnaire entry can be judged as now."}, "sentence_type": "Affirmative declarative sentence", "pos": {"nouns": {"Time": 1, "Head over there": 1, "Ouch": 1}, "verbs": {"I think": 1, "Ouch": 1, "Pong": 1}, "adverbs": {"Sometimes": 1}, "numerals": {"78": 1}}}, {"content": "79, I'm not tired.", "word_count": 6, "label": "", "sentence_structure": "Subject鈥揷opula鈥損redicative", "subjective_objective": {"answer": "Subjective", "rationale": "Because \"not tired\" is a self-perception and feeling of a patient, a subjective examination."}, "sentence_sentiment": {"answer": "Positive", "rationale": "Because the \"hard to get tired\" act is positive."}, "language_style": {"answer": "Tactful and warm", "rationale": "The article is more euphemistic and emotional, and it does not directly indicate whether the person under investigation is tired, but rather raises the question in a question-and-answer manner. It is more personal and interactive, and it is more likely to trigger the resonance and participation of the person under investigation. At the same time, the use of the word \"no\" to deny is a common emotional way of rendering the entry more lively. So, I think, the entry is of the kind of \"false\" type."}, "time_dimension": {"answer": "Present", "rationale": "The entry describes a current state or character of the person being tested, i.e., it is not easy to be tired and can therefore be judged as now."}, "sentence_type": "Affirmative declarative sentence", "pos": {"verbs": {"Easy": 1, "Tired": 1}, "adjectives": {"not": 1}}}, {"content": "I like to study and read things about my current work.", "word_count": 19, "label": "", "sentence_structure": "Subject鈥搗erb鈥搊bject", "subjective_objective": {"answer": "Subjective", "rationale": "Subjectivity is the patient ' s subjective attitude because of the interest in research and reading."}, "sentence_sentiment": {"answer": "Positive", "rationale": "Because the action of \"like to study and read things about my current work\" in the entry is positive."}, "language_style": {"answer": "Tactful and warm", "rationale": "The entry is more euphemistic, using positive terms such as \"like\" and \"research\" and expressing the degree of love and commitment of the respondents to their work. At the same time, the term \"related\" has been replaced by specific fields or professional terms, making the expression more familiar, understandable and easily resonant with the interviewees."}, "time_dimension": {"answer": "Past", "rationale": "It is therefore not possible to judge its temporal dimension."}, "sentence_type": "Affirmative declarative sentence", "pos": {"nouns": {"Work": 1, "Something": 1}, "verbs": {"Yeah": 1, "Research": 1, "Reading": 1, "Related": 1}, "adverbs": {"Current": 1}, "numerals": {"80": 1}}}, {"content": "I like to meet some important people, and that makes me feel as important as I am.", "word_count": 24, "label": "", "sentence_structure": "Subject鈥搗erb鈥搊bject", "subjective_objective": {"answer": "Subjective", "rationale": "Because it is the patient ' s subjective attitude to like to be friends and it is manifested through his own behaviour."}, "sentence_sentiment": {"answer": "Positive", "rationale": "Because the entry \"likes to meet some important people\" expresses a positive attitude and behavior."}, "language_style": {"answer": "Tactful and warm", "rationale": "The entry uses a gentle, invitation-style tone to ask if the other side is interested in knowing important people and suggests that such behaviour may give them a sense of self-worth. This expression is more friendly and interactive, and better guides respondents to think and share their ideas."}, "time_dimension": {"answer": "Present", "rationale": "The time dimension of the article is therefore the present."}, "sentence_type": "Affirmative declarative sentence", "pos": {"nouns": {"81": 1, "I": 1, "person": 1, "Important people": 1, "self": 1}, "verbs": {"Yeah": 1, "I'm meeting someone": 1, "鈾?And I'm gonna be 鈾?: 1, "I feel": 1}, "adjectives": {"Important": 2}, "numerals": {"81": 1, "How many": 1}, "classifiers": {"Some": 1}, "pronouns": {"I": 1}}}, {"content": "82, I don't care what people think of me.", "word_count": 13, "label": "", "sentence_structure": "Subject鈥搗erb鈥搊bject", "subjective_objective": {"answer": "Subjective", "rationale": "Because \"not care what people think about me\" is the patient's subjective attitude and emotional expression."}, "sentence_sentiment": {"answer": "Neutral", "rationale": "Because the entry does not express a positive or negative emotional attitude, it simply states the fact that \"it does not care what others think of themselves\" and is therefore neutral."}, "language_style": {"answer": "Calm and direct", "rationale": "The language style of the entry is more straightforward, with no excessive adoration or emotional colour, and the expression of views through brief statements gives people a sense of calmness and objectivity."}, "time_dimension": {"answer": "Present", "rationale": "The verb \"not care\" in the current state of affairs is used in the entry, indicating that the mentality is the current state or attitude. While this may be a continuing state, in this particular sentence, \"now\" is the most appropriate time description."}, "sentence_type": "Negative declarative sentence", "pos": {"nouns": {"person": 1, "Observations": 1}, "verbs": {"in": 1, "Yes": 1, "have": 1, "to": 1, "What": 1, "Look": 1}, "adverbs": {"not": 1, "I": 1}, "numerals": {"82": 1}, "pronouns": {"Don't": 1}}}, {"content": "83, I'm rarely dazzled.", "word_count": 7, "label": "", "sentence_structure": "Subject鈥搗erb鈥揷omplement", "subjective_objective": {"answer": "Objective", "rationale": "Because dizziness is a symptom or an objective fact of the patient ' s experience."}, "sentence_sentiment": {"answer": "Neutral", "rationale": "The entry is not an emotional expression."}, "language_style": {"answer": "Calm and direct", "rationale": "The entry is in a straightforward manner asking the respondents whether they are often suffering from dizziness, not using excessive rhetorical or emotional rendering, and in a more calm and objective way."}, "time_dimension": {"answer": "Present", "rationale": ""}, "sentence_type": "Negative declarative sentence", "pos": {"verbs": {"You're so dazzling": 1}, "adjectives": {"Very few": 1}, "numerals": {"83": 1}}}, {"content": "84, my mother was a good man.", "word_count": 7, "label": "", "sentence_structure": "Subject鈥揷opula鈥損redicative", "subjective_objective": {"answer": "Subjective", "rationale": "Because \"My mother is a good person\" is a judgment and a perception of the mother, and a subjective attitude."}, "sentence_sentiment": {"answer": "Neutral", "rationale": "The entry is not a clear emotional expression."}, "language_style": {"answer": "Tactful and warm", "rationale": "The language style of the entry is more euphemistic, expressing a positive assessment of the mother, rather than directly describing her strengths or characteristics, but rather using a subjective and emotional expression. Such expression is more likely to resonate and identify the person surveyed, while avoiding the discomfort that too direct or conflicting language may cause."}, "time_dimension": {"answer": "Present", "rationale": "The entry describes the individual view and assessment of the mother of the testee, and there is no specific point in the temporal dimension, so it can be judged as now."}, "sentence_type": "Affirmative declarative sentence", "pos": {"nouns": {"Mother": 1, "Good man": 1}, "verbs": {"be": 1}, "adjectives": {"Okay": 1}, "numerals": {"84": 1}, "pronouns": {"I": 1}}}, {"content": "I'm not so bad at memory.", "word_count": 10, "label": "", "sentence_structure": "Subject鈥揷opula鈥損redicative", "subjective_objective": {"answer": "Subjective", "rationale": "Because \"like\" is a subjective sense, which means that the person who talks feels good about his memory, is a subjective description."}, "sentence_sentiment": {"answer": "Neutral", "rationale": "As the entry describes only an objective fact, it does not involve any emotion or attitude."}, "language_style": {"answer": "Tactful and warm", "rationale": "The article ' s purpose is more accessible, without strong subjective evaluation or direct presentation, and it is more friendly and natural."}, "time_dimension": {"answer": "Present", "rationale": "The entry describes the current state of the testee's feelings and state, and \"seemingly good\" means that his memory does not seem to be in question at this time, so the temporal dimension is now judged."}, "sentence_type": "Affirmative declarative sentence", "pos": {"nouns": {"Memory": 1}, "verbs": {"Seems like": 1, "And": 1, "Good": 1}, "adjectives": {"Okay": 1}, "adverbs": {"And": 1, "Good": 1}, "numerals": {"85": 1}}}, {"content": "I don't think I know what to say when I meet a living person.", "word_count": 20, "label": "", "sentence_structure": "Subject鈥揷opula鈥損redicative", "subjective_objective": {"answer": "Subjective", "rationale": "Because it's the patient's self-perception and attitude."}, "sentence_sentiment": {"answer": "Negative", "rationale": "Because the phrase \"I don't know what to say\" in the entry is negative."}, "language_style": {"answer": "Tactful and warm", "rationale": "The entry is more euphemistic and sensitive to emotional experiences. It uses the first person's term \"me\" to express subjective feelings, and does not judge the person interviewed's behaviour or character too directly or harshly."}, "time_dimension": {"answer": "Present", "rationale": "The entry describes a feeling or state of the person being tested, i.e., that of not knowing what to say when meeting a stranger. This feeling or state is not a past experience, nor a vision of the future, but a present experience, and therefore a judgment."}, "sentence_type": "Affirmative declarative sentence", "pos": {"nouns": {"I": 1, "Time": 1, "Livers": 1, "What": 1}, "verbs": {"I think": 1, "I met him": 1, "Say it": 2, "Okay": 1}, "adjectives": {"Fan": 1}, "adverbs": {"Just": 1, "not": 1}, "numerals": {"86": 1}}}, {"content": "87, when I'm bored, I get in trouble and I'm happy.", "word_count": 14, "label": "", "sentence_structure": "Subject鈥搗erb鈥搊bject", "subjective_objective": {"answer": "Subjective", "rationale": "Because it's the patient's subjective attitude to talk to someone to ease his trouble."}, "sentence_sentiment": {"answer": "Negative", "rationale": "Because the \"problem\" in the entry is negative."}, "language_style": {"answer": "Calm and direct", "rationale": "The language style of the entry is more straightforward, not using excessive rhetorical techniques or emotional rendering, but rather using direct presentation. This is more concise, helping respondents to quickly understand and answer questions."}, "time_dimension": {"answer": "Present", "rationale": "The keyword \"sickness\" and \"disturbing to be happy\" suggests that it is a description of a customary or regular act, and that it occurs at a \"sickness\" time. So the time dimension of this entry should be now."}, "sentence_type": "Affirmative declarative sentence", "pos": {"nouns": {"Time": 1, "Yes": 1, "Happy": 1}, "verbs": {"Yes": 1, "Fuck": 1, "Seek": 1}, "adjectives": {"Boring": 1}, "numerals": {"87": 1}}}, {"content": "88, I'm afraid I'll go crazy.", "word_count": 7, "label": "", "sentence_structure": "Subject鈥搗erb鈥揷omplement", "subjective_objective": {"answer": "Subjective", "rationale": "Because \"crazy\" is the patient's subjective fear and fear."}, "sentence_sentiment": {"answer": "Negative", "rationale": "Because it expresses feelings of fear and fear and has a negative emotional connotation."}, "language_style": {"answer": "Tactful and warm", "rationale": "The entry, which is a moderate and caring expression of concern, expresses a sense of concern and is not subject to too many negative assessments or accusations, which are more likely to resonate and inspire trust among the other party."}, "time_dimension": {"answer": "Present", "rationale": "Fear of the future is expressed in the entry, and \"crazy\" is an unacknowledged possibility, so the temporal dimension is now judged."}, "sentence_type": "Negative declarative sentence", "pos": {"nouns": {"self": 1, "Crazy": 1}, "verbs": {"Yes": 1}, "adverbs": {"Yes": 1, "self": 1}, "numerals": {"88": 1}, "pronouns": {"I": 1}}}, {"content": "89, I hear voices from time to time, and I don't know where they come from.", "word_count": 22, "label": "", "sentence_structure": "Subject鈥搗erb鈥搊bject", "subjective_objective": {"answer": "Subjective", "rationale": "\"The voice of the voice of the voice\" and \"Don't know where it came from\" are subjective experiences and feelings of the patient. Thus, the entry of the questionnaire is viewed from the patient ' s subjective perspective. The basis of the judgement is: - The content of the question concerns subjective factors such as the person ' s perception, experience and emotions; - No objective facts or data are provided to examine it."}, "sentence_sentiment": {"answer": "Neutral", "rationale": "Because the entry only describes a phenomenon, it does not express any positive or negative emotional colours."}, "language_style": {"answer": "Tactful and warm", "rationale": "The entry is more personal and interactive when it is spoken in a gentle and inviting manner, rather than in direct statements or orders. At the same time, the expression \"frequently\" and \"unknown\" is more euphemistic, without too much emphasis or blame on the other, and is acceptable and willing to answer questions."}, "time_dimension": {"answer": "Present", "rationale": "The word \"time\" in \"I often\" denotes a regular, customary act, and \"hear\" adopts the normal current, meaning that the act not only has occurred in the past but may still be ongoing. So the entry describes the person being tested in his current act or state."}, "sentence_type": "Interrogative sentence", "pos": {"nouns": {"Sound": 2, "Local": 1}, "verbs": {"Yes": 2, "From": 1}, "adverbs": {"Often": 1}, "numerals": {"89": 1}, "pronouns": {"I": 1}}}, {"content": "I often find my hands shaking when I have to do something.", "word_count": 20, "label": "", "sentence_structure": "Subject鈥搗erb鈥揷omplement", "subjective_objective": {"answer": "Subjective", "rationale": "The questionnaire is intended to observe the behaviour and feelings of \"me\" and is therefore viewed from a subjective perspective."}, "sentence_sentiment": {"answer": "Negative", "rationale": "Because the \"hand shaking\" act in the entry is negative."}, "language_style": {"answer": "Tactful and warm", "rationale": "The entry uses a more subjective description of feelings ( \"shocking\" ) and a more euphemistic expression, which leads the respondents to share their inner feelings in a more moderate and understanding way, rather than directly asking or assessing. This style usually helps to build trust and comfort and encourages respondents to answer questions more honestly."}, "time_dimension": {"answer": "Present", "rationale": "The entry describes the current physiological reaction of the testee, i.e., that he or she will find his or her hand shaking when he or she wants to do something. Therefore, the time dimension is now judged."}, "sentence_type": "Affirmative declarative sentence", "pos": {"nouns": {"Yes": 1, "Hands": 1, "# Wresh #": 1}, "verbs": {"Do it": 1, "I realized": 1}, "adverbs": {"Regular": 1}, "numerals": {"90": 1}}}, {"content": "91, I can read for a long time, and my eyes don't feel tired.", "word_count": 17, "label": "", "sentence_structure": "Subject鈥搗erb鈥揷omplement", "subjective_objective": {"answer": "Subjective", "rationale": "Because the entry describes the patient鈥檚 subjective feelings and experiences, not the observed facts or behaviours."}, "sentence_sentiment": {"answer": "Positive", "rationale": "Because the act of \"reading for a long time\" is positive, and it means good focus and persistence."}, "language_style": {"answer": "Tactful and warm", "rationale": "Because the entry does not directly use strong language or command tone to question each other, but rather engages in a gentle and invited dialogue. It provides a context and tries to understand whether the other party can still feel good after reading for a long time. This expression is more intimate and interactive, and it feels respected and understood."}, "time_dimension": {"answer": "Present", "rationale": "The entry describes the current situation or customary behaviour of the person being tested, in which the \"readable long time\" indicates that the person being tested has such capability and is using it, and thus judging the current state."}, "sentence_type": "Affirmative declarative sentence", "pos": {"nouns": {"Time": 1, "Eyes": 1}, "verbs": {"Readable": 1, "I think": 1}, "adjectives": {"Long": 1, "I'm tired": 1}, "numerals": {"91": 1}, "pronouns": {"I": 1}}}, {"content": "92, many times I feel powerless.", "word_count": 11, "label": "", "sentence_structure": "Subject鈥損redicate", "subjective_objective": {"answer": "Subjective", "rationale": "Because the verb \"think\" suggests that it's a patient's subjective feeling or experience."}, "sentence_sentiment": {"answer": "Negative", "rationale": "Because the description of \"frightful\" in the entry is negative."}, "language_style": {"answer": "Tactful and warm", "rationale": ""}, "time_dimension": {"answer": "Present", "rationale": ""}, "sentence_type": "Affirmative declarative sentence", "pos": {"nouns": {"Time": 1, "I'm so weak": 1}, "verbs": {"I think": 1}, "adverbs": {"Many": 1}, "numerals": {"92": 1}}}, {"content": "Ninety-three, I have little headaches.", "word_count": 5, "label": "", "sentence_structure": "Subject鈥揷opula鈥損redicative", "subjective_objective": {"answer": "Objective", "rationale": "Since headaches or non-headaches are the expression of objective symptoms of the patient, the frequency of non-headaches is also an objective fact that can be directly observed and measured."}, "sentence_sentiment": {"answer": "Neutral", "rationale": "Because the entry does not have any emotional color."}, "language_style": {"answer": "Calm and direct", "rationale": "The language style of the entry is calm and straightforward, because it presents the fact in a concise and clear manner that the person under investigation has little headaches. Instead of using any rhetorical or emotional colour to qualify the situation, it adopts an objective neutral attitude."}, "time_dimension": {"answer": "Past", "rationale": ""}, "sentence_type": "Negative declarative sentence", "pos": {"verbs": {"Very few": 1, "Ouch": 1}, "adjectives": {"Headache": 1}, "numerals": {"93": 1}}}, {"content": "It's not difficult to walk smooth when I'm 94.", "word_count": 12, "label": "", "sentence_structure": "Subject鈥搗erb鈥揷omplement", "subjective_objective": {"answer": "Objective", "rationale": "Since calm does not depend too much on the patient鈥檚 will and will, it is an objective result."}, "sentence_sentiment": {"answer": "Neutral", "rationale": "Because the entry does not have any emotional color."}, "language_style": {"answer": "Tactful and warm", "rationale": "The entry uses positive expressions to describe behaviour characteristics, conveys a state of ease, moderate tone and encouragement by expressing \"no difficulty\"."}, "time_dimension": {"answer": "Present", "rationale": "The keyword \"I walk\" refers to the behaviour that is currently being performed, and \"salts, not difficult\" also indicates that it is a state or behaviour. The entry therefore indicates the current situation of the person who was tested when completing the questionnaire."}, "sentence_type": "Affirmative declarative sentence", "pos": {"nouns": {"I": 1, "Walk": 1}, "verbs": {"Hold on": 1, "and": 1, "not": 1, "Difficulties": 1}, "adjectives": {"Steady": 1, "Difficulties": 1}, "adverbs": {"Time": 1, "not": 1}, "numerals": {"94": 1}}}, {"content": "95, I don't have asthma.", "word_count": 8, "label": "", "sentence_structure": "Subject鈥搗erb鈥搊bject", "subjective_objective": {"answer": "Subjective", "rationale": "Because no asthma is a subjective judgment or feeling, different people have different physical conditions and self-perception."}, "sentence_sentiment": {"answer": "Neutral", "rationale": "Because the entry merely stated a fact, it did not express any positive or negative feelings."}, "language_style": {"answer": "Calm and direct", "rationale": "The language style of the entry is calm and straightforward, as it clearly presents a fact and does not use any qualifier or expression of strong emotions, and is more objective."}, "time_dimension": {"answer": "Present", "rationale": "The entry appears \"No asthma-like disease\", indicating that the person tested is not currently suffering from asthma-like diseases. Thus, the temporal dimension is now."}, "sentence_type": "Negative declarative sentence", "pos": {"nouns": {"Asthma": 1, "Category I": 1, "Diseases": 1}, "verbs": {"Nope": 1}, "numerals": {"95": 1}}}, {"content": "I was in a few instances when I suddenly had no control over my actions or words, but I was still awake.", "word_count": 32, "label": "", "sentence_structure": "Subject鈥搗erb鈥搊bject", "subjective_objective": {"answer": "Subjective", "rationale": "Because the description mainly concerns the patient's own experience and feelings, including \"I've had a few times\" and \"my mind is clear,\" which are subjective feelings and perceptions of the patient."}, "sentence_sentiment": {"answer": "Positive", "rationale": "Because the description of \"sane mind\" in the entry indicates that, although there has been a situation of uncontrolledness, the individual's awareness and judgment still exist in that context, which is a relatively positive description."}, "language_style": {"answer": "Calm and direct", "rationale": "The entry uses a clear and concise language describing the physical condition of the subject, and there is no excessive rhetorical or emotional colour. At the same time, the question is more straightforward, and it is understood at first glance. The language style of the questionnaire entry can therefore be judged as \"cooling straight.\""}, "time_dimension": {"answer": "Past", "rationale": ""}, "sentence_type": "Affirmative declarative sentence", "pos": {"nouns": {"Number of times": 1, "Action": 1, "self": 2, "Words": 1, "Brain": 1, "Wake up": 1}, "verbs": {"Yes": 1, "Suddenly": 1, "I can't control it": 1, "And": 1}, "adverbs": {"Once": 1, "But": 1, "At the time": 1}, "numerals": {"96": 1, "How many": 1}, "pronouns": {"I": 1}}}, {"content": "97, someone's been trying to rob me of something.", "word_count": 10, "label": "", "sentence_structure": "Subject鈥搗erb鈥搊bject", "subjective_objective": {"answer": "Subjective", "rationale": "Because it is a subjective feeling or experience, not an objective fact."}, "sentence_sentiment": {"answer": "Negative", "rationale": "Because it expresses a negative offensive behaviour."}, "language_style": {"answer": "Tactful and warm", "rationale": "The entry presents questions in a more euphemistic way, not directly accusing or attacking the other, but directing the other to answer questions in an understanding and respectable manner. At the same time, this expression is easier to accept and answer questions by the interviewee."}, "time_dimension": {"answer": "Past", "rationale": ""}, "sentence_type": "Affirmative declarative sentence", "pos": {"nouns": {"person": 1, "Something": 1}, "verbs": {"have": 1, "Yes": 1, "Grab it": 1}, "adverbs": {"Always": 1}, "numerals": {"97": 1}}}, {"content": "I'm not too much of a phantom.", "word_count": 5, "label": "", "sentence_structure": "Subject鈥搗erb鈥揷omplement", "subjective_objective": {"answer": "Subjective", "rationale": "Because \"irritive\" is a subjective psychological activity, the entry is viewed from the subjective perspective of the patient."}, "sentence_sentiment": {"answer": "Neutral", "rationale": "Because the entry does not have any emotional color."}, "language_style": {"answer": "Calm and direct", "rationale": "The language style of the entry is calm and straightforward. It uses simple and clear language, does not have too many rhetorical or emotional expressions, is more objective in tone, and is directly asked whether the person under investigation often engages in the act of ulterior thought."}, "time_dimension": {"answer": "Present", "rationale": "Now the keyword \"very rarely\" means that the act is in its current state of custom, that \"irritive\" is a past act, and that is why the entry is judged to be asking about the present behaviour or mentality."}, "sentence_type": "Negative declarative sentence", "pos": {"verbs": {"I'm not sure": 1}, "adjectives": {"Very few": 1}, "numerals": {"98": 1}}}, {"content": "99, someone tried to steal my ideas and plans.", "word_count": 12, "label": "", "sentence_structure": "Subject鈥搗erb鈥搊bject", "subjective_objective": {"answer": "Subjective", "rationale": "Because \"someone wants to steal my ideas and plans\" is a subjective feeling and a perception of a human being, not an objective fact."}, "sentence_sentiment": {"answer": "Negative", "rationale": "Because the \"wanted to steal my thoughts and plans\" act in the entry is negative."}, "language_style": {"answer": "Calm and direct", "rationale": "The entry is more straightforward in language style, is not overly phrasing or emotional, but is presented in an objective factual manner, expressing concerns and concerns about privacy and safety."}, "time_dimension": {"answer": "Present", "rationale": "The entry now asks if the person who was tested currently has a situation where someone is trying to steal their ideas and plans, and thus judges as the current state of affairs."}, "sentence_type": "Affirmative declarative sentence", "pos": {"nouns": {"person": 1, "Thoughts": 1, "Planned": 1}, "verbs": {"Yes": 1, "Steal": 1}, "numerals": {"99": 1}, "pronouns": {"Mine": 1}}}, {"content": "I believe I'm a condemned man.", "word_count": 12, "label": "", "sentence_structure": "Subject鈥搗erb鈥揷omplement", "subjective_objective": {"answer": "Subjective", "rationale": "Because \"convincing\" is the patient's subjective attitude, and \"criticized\" is the patient's subjective assessment of himself."}, "sentence_sentiment": {"answer": "Negative", "rationale": "Because the description of \"stigmatized person\" in the entry is negative."}, "language_style": {"answer": "Calm and direct", "rationale": "The language style of the entry is calm and straightforward, as it uses simple sentence structures and direct expressions to ask the other side whether it believes that it is a condemned person. Moreover, it does not contain any emotional or rhetorical style, but presents the problem in an objective manner."}, "time_dimension": {"answer": "Present", "rationale": "The keywords are \"Believe\" and \"Be\". They all express the current attitude or belief of the speaker. So, the entry is asking the person who's been tested for what he thinks and feels."}, "sentence_type": "Affirmative declarative sentence", "pos": {"nouns": {"person": 1, "Condemnation": 1}, "verbs": {"Yes": 1, "be": 1, "By": 1}, "numerals": {"100": 1}, "pronouns": {"I": 1}}}, {"content": "101, if I were a journalist, I would like to report to the theatre.", "word_count": 22, "label": "", "sentence_structure": "Subject鈥搗erb鈥搊bject", "subjective_objective": {"answer": "Subjective", "rationale": "Because \"news like to report to the theatre\" is the expression of the respondents' attitude and perception of their supposed situation, which is subjective."}, "sentence_sentiment": {"answer": "Positive", "rationale": "Because it expresses an attitude of being a fan of reporting to the theatre news, there is no negative emotion or neutrality."}, "language_style": {"answer": "Tactful and warm", "rationale": "The language style of the entry is euphemism. It does not express \"I like to report on the theatre\" directly, but rather by assuming that I am a journalist and expressing my own feelings of a good news story. This expression is more polite and kind and more resonant and acclaimable."}, "time_dimension": {"answer": "Present", "rationale": "In the future, the \"if\" in the entry indicates that if the future scenario is assumed, \"will like to report\" indicates the expectation and planning of the future, and therefore the temporal dimension is judged to be the future."}, "sentence_type": "Hypothetical sentence", "pos": {"nouns": {"Journalists": 1, "Theatrical world": 1, "Public information": 2}, "verbs": {"be": 1, "Yeah": 1, "Report to the station": 1}, "adverbs": {"If": 1}, "numerals": {"101": 1}, "pronouns": {"I": 1}}}, {"content": "102, sometimes I can't help but try to steal something.", "word_count": 13, "label": "", "sentence_structure": "Subject鈥損redicate", "subjective_objective": {"answer": "Subjective", "rationale": "Because \"want to steal something\" is the patient's inner thoughts and feelings, and it's a subjective examination."}, "sentence_sentiment": {"answer": "Negative", "rationale": "Because the \"I can't control the desire to steal\" thing in the entry is negative."}, "language_style": {"answer": "Tactful and warm", "rationale": "This entry is used in a more euphemistic way, which leads the interviewee to reflect on his/her behaviour, rather than directly accusing or criticizing him/her."}, "time_dimension": {"answer": "Present", "rationale": ""}, "sentence_type": "Affirmative declarative sentence", "pos": {"verbs": {"Control": 1, "Yes": 1, "Steal": 1}, "adjectives": {"Points": 1}, "adverbs": {"Sometimes": 1}, "numerals": {"102": 1}}}, {"content": "103. I like many different kinds of games and entertainment.", "word_count": 15, "label": "", "sentence_structure": "Subject鈥搗erb鈥搊bject", "subjective_objective": {"answer": "Subjective", "rationale": "Because I like being patient subjective."}, "sentence_sentiment": {"answer": "Positive", "rationale": "Because the desire for different types of games and entertainment demonstrates an open, pluralistic mentality and a positive emotional attitude."}, "language_style": {"answer": "Tactful and warm", "rationale": "The language style of the entry is more euphemistic, using positive words like \"like\" and not too direct or hard-line to ask each other about their interests, but rather to engage in a friendly and gentle dialogue. Moreover, \"many different types\" also show the respect and inclusiveness of the questionnaire designers in terms of diversity, which can make the interviewee feel more comfortable and freely expressing his thoughts."}, "time_dimension": {"answer": "Present", "rationale": "\"I like\" moves take place at some point in the present and is a continuous feeling and evaluation of \"many different kinds of games and entertainment.\" So the time dimension of this questionnaire entry is now."}, "sentence_type": "Affirmative declarative sentence", "pos": {"nouns": {"Game": 1, "Recreation": 1}, "verbs": {"Yeah": 1, "Many": 1, "Different": 1, "Category": 1}, "adjectives": {"Different types": 1}, "numerals": {"103": 1}}}, {"content": "104, I like to laugh with the opposite sex.", "word_count": 8, "label": "", "sentence_structure": "Subject鈥搗erb鈥搊bject", "subjective_objective": {"answer": "Subjective", "rationale": "Because I like to laugh with opposite sex is my own subjective feeling or attitude."}, "sentence_sentiment": {"answer": "Positive", "rationale": "Because \"like\" this attitude is positive."}, "language_style": {"answer": "Tactful and warm", "rationale": ""}, "time_dimension": {"answer": "Present", "rationale": ""}, "sentence_type": "Affirmative declarative sentence", "pos": {"nouns": {"Heterosexual": 1, "Smile": 1}, "verbs": {"Yeah": 1, "Say it": 1}, "numerals": {"104": 1}}}, {"content": "105 I believe my sins are inexcusable.", "word_count": 13, "label": "", "sentence_structure": "Subject鈥損redicate", "subjective_objective": {"answer": "Subjective", "rationale": "It is an objective fact that belief is a subjective attitude and conviction, and whether evil is condoned requires a judgement based on specific legal or moral standards. The sentence is a subjective idea of the patient and is a subjective one."}, "sentence_sentiment": {"answer": "Negative", "rationale": "Because the attitude in the entry, \"Believe that your sins are unforgivable\" is negative."}, "language_style": {"answer": "Calm and direct", "rationale": "The language style of the entry is calm and straightforward. It clearly expresses the view of the interviewees about their own evils, and does not use too much embellished or emotional expressions, which are more neutral and objective."}, "time_dimension": {"answer": "Present", "rationale": "The entry uses the first person 's name 'me' and expresses a belief or attitude, so the temporal dimension is now judged."}, "sentence_type": "Negative declarative sentence", "pos": {"nouns": {"Sin": 1, "Forgiveness": 1}, "verbs": {"Yes": 1, "be": 1, "No way": 1, "Forgive me": 1}, "adjectives": {"I'm not going to forgive you": 1}, "numerals": {"105": 1, "I": 1}, "pronouns": {"I": 1}}}, {"content": "106, everything tastes the same.", "word_count": 14, "label": "", "sentence_structure": "Subject鈥揷opula鈥損redicative", "subjective_objective": {"answer": "Objective", "rationale": "Because it describes the attributes of the thing, not the individual ' s feelings or perceptions, it is objective."}, "sentence_sentiment": {"answer": "Neutral", "rationale": "Because the entry expresses an objective fact, which has no obvious emotional colour."}, "language_style": {"answer": "Calm and direct", "rationale": "The language style of the entry is more objective and calm, with no excessive emotional or rhetorical tact, and a direct statement of a view or fact, which is more direct in tone."}, "time_dimension": {"answer": "Present", "rationale": "\"Eating\" is the current state of play, and the description of \"everything\" is based on current feelings and observations, so the subject is the current perception of the subjects."}, "sentence_type": "Affirmative declarative sentence", "pos": {"nouns": {"Something": 2, "Tastes": 1}, "verbs": {"Eat": 1, "Both": 1}, "adjectives": {"Same thing": 1}, "numerals": {"106": 1}}}, {"content": "107, my family treats me like a child, not as an adult.", "word_count": 22, "label": "", "sentence_structure": "Subject鈥搗erb鈥揷omplement", "subjective_objective": {"answer": "Subjective", "rationale": "Because this phrase expresses the patient ' s view and feelings that he/she believes that his/her family member does not treat him/her as an adult, it is the patient ' s subjective idea."}, "sentence_sentiment": {"answer": "Negative", "rationale": "Because it expresses a feeling of contempt and neglect and has a negative emotional connotation."}, "language_style": {"answer": "Tactful and warm", "rationale": "The entry is more euphemistic, is more implicit in its language and does not involve too direct accusations or criticism. At the same time, by using the expressions \"as a child\" and \"as a person without adult consideration\" it allows the respondents to accept questions and answer questions more comfortably, increasing the completion rate of the questionnaire."}, "time_dimension": {"answer": "Present", "rationale": "The sentence describes the current situation, where \"the family\" and \"the child\" and \"not treating me as an adult\" are described in terms of the current state and circumstances. It therefore determines that the entry is in the current time dimension."}, "sentence_type": "Negative declarative sentence", "pos": {"nouns": {"person": 2, "Kids": 1, "Sir": 1}, "verbs": {"- Put it on": 3, "I'll take it as a treat": 2, "View": 1}, "numerals": {"107": 1, "2": 1, "1": 4}, "classifiers": {"individual": 1}, "pronouns": {"I": 1}}}, {"content": "I have a family that lacks love and warmth compared to others.", "word_count": 18, "label": "", "sentence_structure": "Subject鈥搗erb鈥搊bject", "subjective_objective": {"answer": "Subjective", "rationale": "Because \"lack of love and warmth\" is a subjective feeling and evaluation."}, "sentence_sentiment": {"answer": "Negative", "rationale": "Because \"Leave Love and Warm\" in the entry is negative."}, "language_style": {"answer": "Tactful and warm", "rationale": "By comparing itself with others and expressing its feelings in the negative word \"lack,\" the entry shows a mild and implicit emotional tendency. At the same time, the abstract concept of \"love and warm\" is used, making the question more deep and open, allowing respondents to think and answer further."}, "time_dimension": {"answer": "Past", "rationale": "At the same time, \"lack of love and warmth\" shows the testee's feelings about his family environment, which is subjective and uncertain in terms of time dimensions. Taken together, the temporal dimension of the article can be judged as \"present or past\"."}, "sentence_type": "Negative declarative sentence", "pos": {"nouns": {"Family": 2, "Love": 1, "Warm": 1}, "verbs": {"Comparison": 1, "Lack": 1, "Here": 1}, "numerals": {"108": 1, "2": 1}, "classifiers": {"individual": 1}, "pronouns": {"I": 1}}}, {"content": "I often feel I'm worried about something.", "word_count": 15, "label": "", "sentence_structure": "Subject鈥搗erb鈥搊bject", "subjective_objective": {"answer": "Subjective", "rationale": "Because \"I feel often\" is part of the patient's subjective feeling."}, "sentence_sentiment": {"answer": "Negative", "rationale": "Because \"fear\" in the entry is a negative emotional experience."}, "language_style": {"answer": "Calm and direct", "rationale": "The entry is dialogueable in a direct question-and-answer manner, without excessive rhetorical or emotional rendering, and is more objective in tone."}, "time_dimension": {"answer": "Present", "rationale": "\"I often feel \"always\" means \"recurring, habitually\" and \"sense\" uses the general current, meaning that this behaviour not only has occurred in the past, but may still be ongoing. So the entry describes the current feeling or state, which is in the present context."}, "sentence_type": "Affirmative declarative sentence", "pos": {"nouns": {"self": 1, "Yes": 1, "Fear": 1}, "verbs": {"Often": 1, "I feel": 1, "Fear": 1}, "adverbs": {"Often": 1, "Yes": 1}, "numerals": {"109": 1}, "pronouns": {"I": 1}}}, {"content": "110, I love my mother.", "word_count": 6, "label": "", "sentence_structure": "Subject鈥搗erb鈥搊bject", "subjective_objective": {"answer": "Subjective", "rationale": "Because \"I love you\" is a subjective emotional expression and attitude that reflects the feeling and emotional state of the individual. So this questionnaire entry is viewed from the patient's subjective point of view."}, "sentence_sentiment": {"answer": "Positive", "rationale": "Because the phrase \"I love my mother\" in the entry is positive."}, "language_style": {"answer": "Tactful and warm", "rationale": "The entry is presented in a way that expresses love, is more tender and friendly, and does not have a direct statement or order, and is more intimate and interactive."}, "time_dimension": {"answer": "Past", "rationale": "The entry expresses the emotional attitude of the testee to the past, because \"love\" is a verb describing the emotional state of the past and does not have a clear point of reference for time."}, "sentence_type": "Affirmative declarative sentence", "pos": {"nouns": {"110": 1, "Mother": 1}, "verbs": {"Love": 1}}}, {"content": "111, even if I cannot repay my favor later, I will turn to my friends for help.", "word_count": 19, "label": "", "sentence_structure": "Subject鈥損redicate", "subjective_objective": {"answer": "Subjective", "rationale": "The patient is subjective in his willingness to turn to his friends."}, "sentence_sentiment": {"answer": "Positive", "rationale": "The entry is an expression of gratitude to friends and a willingness to help them."}, "language_style": {"answer": "Tactful and warm", "rationale": "The analysis is based on the following: the words \"even if...\" are used in the beginning of the article, and are often structured to express a euphemism or will, and to show a sense of respect and gratitude by the speaker. At the same time, the entry uses the term \"friend\" as a more intimate and friendly way of describing it. Thus, in terms of language style, the entry is warm."}, "time_dimension": {"answer": "Present", "rationale": "The entry uses the term \"after\", but the entire sentence expresses the current psychological state and emotional tendency to seek help from friends even if it is not possible to repay in the future. So, it is now."}, "sentence_type": "Affirmative declarative sentence", "pos": {"nouns": {"Grace": 1, "friends": 1}, "verbs": {"Refund": 1, "Help": 1}, "adverbs": {"Even if": 1}, "numerals": {"111": 1}, "pronouns": {"I": 2}}}, {"content": "112, my parents often object to those who are with me.", "word_count": 15, "label": "", "sentence_structure": "Subject鈥搗erb鈥搊bject", "subjective_objective": {"answer": "Subjective", "rationale": "This is because the views and feelings of the patient are subjective towards those with whom his or her parents oppose dealings."}, "sentence_sentiment": {"answer": "Negative", "rationale": "Because the \"parents against the object\" in the entry is negative."}, "language_style": {"answer": "Calm and direct", "rationale": "The entry directly expresses the subject of questioning the parents of the other party about their frequent opposition to their clients, is more objective and is not based on excessive rhetoric or emotional rendering.\""}, "time_dimension": {"answer": "Present", "rationale": "My parents have a general state of affairs, and the \"recurring opposition\" is also an ongoing act, so the entry is an expression of the current behaviour or state."}, "sentence_type": "Imperative sentence", "pos": {"nouns": {"parents": 1, "person": 2, "- To be with someone": 1}, "verbs": {"Objection": 1, "- To be with someone": 1}, "adverbs": {"Regular": 1}, "numerals": {"112": 1}, "pronouns": {"Those": 1}}}, {"content": "113, I've never been in a panic.", "word_count": 11, "label": "", "sentence_structure": "Subject鈥揷opula鈥損redicative", "subjective_objective": {"answer": "Objective", "rationale": "Because it is the experience of patients who do not feel the panic, it is an objective fact."}, "sentence_sentiment": {"answer": "Neutral", "rationale": "This entry is merely a description of the fact that an individual has never felt a panic, and does not express a positive or negative emotional attitude."}, "language_style": {"answer": "Calm and direct", "rationale": "The entry directly asks the interviewees whether they have had a short-term experience of panic, a more calm and objective language style, and not too much adoration or emotional colour."}, "time_dimension": {"answer": "Past", "rationale": "The entry asks the testee about the past feeling that he or she has been in a state of panic before the current time point for completing the questionnaire."}, "sentence_type": "Affirmative declarative sentence", "pos": {"nouns": {"I'm scared": 1, "Short air": 1}, "verbs": {"I feel": 1}, "adverbs": {"Never": 1}, "numerals": {"113": 1}}}, {"content": "114, I have a lot of things on my mind.", "word_count": 6, "label": "", "sentence_structure": "Subject鈥搗erb鈥揷omplement", "subjective_objective": {"answer": "Subjective", "rationale": "Because \"many things\" are subjective descriptions and perceptions of the inner world."}, "sentence_sentiment": {"answer": "Neutral", "rationale": "Basis of judgement: The entry describes only a phenomenon and does not contain any obvious emotional colours."}, "language_style": {"answer": "Tactful and warm", "rationale": "The language style of the entry is euphemism. It uses more moderate words and tone, such as \"many things in the heart,\" without directly accusing or criticizing the other. At the same time, the subject is also an invitation to direct respondents to answer, giving them a sense of affection."}, "time_dimension": {"answer": "Present", "rationale": "The entry describes the current state and feelings of the person being tested, uses the general current state and does not clearly point to the past or future time dimension."}, "sentence_type": "Affirmative declarative sentence", "pos": {"nouns": {"Something's on the mind": 1}, "verbs": {"have": 1}, "numerals": {"114": 1}}}, {"content": "115, sometimes I'm very restless and restless.", "word_count": 11, "label": "", "sentence_structure": "Subject鈥搗erb鈥揷omplement", "subjective_objective": {"answer": "Subjective", "rationale": "The patient's subjective feelings and manifestations are the result of irritation and restlessness."}, "sentence_sentiment": {"answer": "Negative", "rationale": "Because \"freak\" and \"sitting\" are negative emotional and behavioural expressions."}, "language_style": {"answer": "Tactful and warm", "rationale": "The entry uses a first-person expression that allows respondents to express their feelings more naturally and uses a moderate, indirect language style, making the questionnaire more incisive."}, "time_dimension": {"answer": "Past", "rationale": "The entry uses \"sometimes\" to indicate that such irritating and restless behaviour may have occurred occasionally and is described at a point in time in the past."}, "sentence_type": "Affirmative declarative sentence", "pos": {"nouns": {"I'm so upset": 1, "Sit up": 1, "Unsettled": 1}, "verbs": {"Time": 1, "have": 1, "I'm so upset": 1, "Sit up": 1, "Unsettled": 1}, "adjectives": {"Tens": 1}, "numerals": {"115": 1}}}, {"content": "116, I've been in love.", "word_count": 6, "label": "", "sentence_structure": "Subject鈥搗erb鈥揷omplement", "subjective_objective": {"answer": "Objective", "rationale": "Because \"lost\" is an objective fact that patients have experienced."}, "sentence_sentiment": {"answer": "Negative", "rationale": "Because \"lost\" is usually considered a negative life experience."}, "language_style": {"answer": "Calm and direct", "rationale": "The entry directly expresses the subject of questioning the other party for negligence, without excessive rhetorical or emotional rendering, and with a more objective tone."}, "time_dimension": {"answer": "Past", "rationale": "The entry contains the word \"had\" and \"had broken love\" and they all point to events or states that have occurred in the past and therefore judge as past."}, "sentence_type": "Affirmative declarative sentence", "pos": {"verbs": {"Once": 1, "Broken love": 1}, "numerals": {"116": 1}}}, {"content": "I've always dreamed of something that's not to be seen.", "word_count": 12, "label": "", "sentence_structure": "Subject鈥搗erb鈥揷omplement", "subjective_objective": {"answer": "Subjective", "rationale": "Because the content of a dream is usually a reflection of the thoughts or feelings that are deep inside the individual, the entry is viewed from a subjective perspective."}, "sentence_sentiment": {"answer": "Negative", "rationale": "Because the entry \"dreaming something unsettled\" could cause anxiety and negative emotions."}, "language_style": {"answer": "Tactful and warm", "rationale": "The entry uses a relatively euphemistic expression, which does not directly ask the other side whether they often have unspoken dreams, but rather leads the other side to think and answer questions in an implicit way that protects the privacy of the person interviewed and makes the questionnaire more natural."}, "time_dimension": {"answer": "Present", "rationale": "I often dream of \"normal\" behaviour, which is frequent, customary, and \"dreaming\" that uses the general current, which means that it has not only happened in the past, but may still be ongoing. So the entry describes the current state and experience of the testee."}, "sentence_type": "Affirmative declarative sentence", "pos": {"nouns": {"Yes": 1, "Dreams": 1}, "verbs": {"Regular": 1, "I dreamt about it": 1}, "adjectives": {"No one's ever heard of it": 1}, "numerals": {"117": 1}, "pronouns": {"I": 1}}}, {"content": "118, I believe I'm no more sensitive than others.", "word_count": 15, "label": "", "sentence_structure": "Subject鈥搗erb鈥搊bject", "subjective_objective": {"answer": "Subjective", "rationale": "Because I believe that this is the patient ' s personal opinion or belief, and it is a subjective attitude or opinion."}, "sentence_sentiment": {"answer": "Neutral", "rationale": "Because the entry does not have any emotional color."}, "language_style": {"answer": "Calm and direct", "rationale": "The entry is asked in a straightforward and concise manner whether the person surveyed did not consider himself to be more sensitive than others, without excessive rhetoric or emotional rendering, and expressed more objectively calm."}, "time_dimension": {"answer": "Past", "rationale": "Because the word \"convincing\" in the sentence is a manifestation of past beliefs or attitudes and it describes a general situation, namely, \"no more sensitive than others\", and therefore judges the past."}, "sentence_type": "Negative declarative sentence", "pos": {"nouns": {"Yes": 1, "others": 1, "You're a bit of a sane": 1}, "verbs": {"Yes": 2, "and": 1, "- Yeah": 1, "not": 1, "More": 1, "be": 1}, "adverbs": {"and": 1, "not": 1, "More": 1}, "numerals": {"118": 1}, "pronouns": {"I": 1}}}, {"content": "119, I'm almost in pain.", "word_count": 11, "label": "", "sentence_structure": "Subject鈥搗erb鈥揷omplement", "subjective_objective": {"answer": "Objective", "rationale": "Because pain is an objective physical state, it is also an objective sensory experience to feel pain. Although patients鈥?subjective feelings may influence their answers, the answer to this question is based on their actual observation and perception of their physical state, and therefore objective."}, "sentence_sentiment": {"answer": "Neutral", "rationale": "Because the entry does not have any emotional color."}, "language_style": {"answer": "Calm and direct", "rationale": "The entry is based on simple speech structure, \"I have almost no pain\" which directly expresses the subject, and is not accompanied by excessive rhetoric or emotional rendering, which is more objective and balanced. At the same time, the question-setting of the article does not involve any emotional or implicit meaning, which is more in keeping with a calm and straightforward description."}, "time_dimension": {"answer": "Present", "rationale": "The entry \"near anywhere in pain\" indicates the current state of the person being tested, so the temporal dimension is now judged."}, "sentence_type": "Negative declarative sentence", "pos": {"nouns": {"Local": 1, "Pain": 1}, "verbs": {"have": 1, "Almost": 1}, "adjectives": {"What": 1, "Almost": 1}, "adverbs": {"Both": 1}, "numerals": {"119": 1}, "classifiers": {"individual": 1}, "pronouns": {"I": 1}}}, {"content": "I'm not sure how I do things.", "word_count": 12, "label": "", "sentence_structure": "Subject鈥搗erb鈥搊bject", "subjective_objective": {"answer": "Subjective", "rationale": "Because \"vulnerable\" reflects the patient's perception and feelings about his behaviour and is subjective."}, "sentence_sentiment": {"answer": "Negative", "rationale": "Because the expression \"vulnerable\" is negative."}, "language_style": {"answer": "Tactful and warm", "rationale": "The entry, by means of an inquiry, allows the respondent to share his thoughts and feelings on his own initiative, is relatively gentle and avoids the possibility of direct criticism or accusations, and can therefore be considered a mild expression."}, "time_dimension": {"answer": "Present", "rationale": ""}, "sentence_type": "Negative declarative sentence", "pos": {"nouns": {"- How to do things": 1, "person": 1}, "verbs": {"Easy": 1, "Misunderstanded": 1}, "numerals": {"120": 1}}}, {"content": "My parents and family are too picky on me.", "word_count": 14, "label": "", "sentence_structure": "Subject鈥搗erb鈥搊bject", "subjective_objective": {"answer": "Subjective", "rationale": "Because this phrase expresses patients ' subjective feelings and perceptions of themselves, that is, they consider their families to be too critical to themselves."}, "sentence_sentiment": {"answer": "Negative", "rationale": "Because the word \"choice\" in the entry is a derogatory term, which is a negative assessment."}, "language_style": {"answer": "Tactful and warm", "rationale": "The language style of the entry is more euphemistic, because it does not directly criticize or accuse the parents or family of the other, but rather describes the individual鈥檚 feelings in an objective and understandable manner."}, "time_dimension": {"answer": "Present", "rationale": ""}, "sentence_type": "Negative declarative sentence", "pos": {"nouns": {"parents": 1, "Family": 1}, "verbs": {"to": 1, "Too much": 1, "Picky": 1}, "adjectives": {"Family": 1}, "adverbs": {"Pass": 1}, "numerals": {"121": 1, "One": 1, "Two": 1, "III": 1}, "pronouns": {"I": 1}}}, {"content": "I have red spots on my neck, which are frequent.", "word_count": 11, "label": "", "sentence_structure": "Subject鈥搗erb鈥搊bject", "subjective_objective": {"answer": "Objective", "rationale": "Because \"red spots are often found on the neck\" is an objective physical condition of the patient."}, "sentence_sentiment": {"answer": "Negative", "rationale": "Because the sign \"red spots\" in the entry is negative."}, "language_style": {"answer": "Tactful and warm", "rationale": "The entry uses a milder description, without directly emphasizing the severity of the illness or symptoms, but rather the means of directing the interviewee to express his feelings and experiences, which makes it easier for the interviewee to develop a sense of proximity and security."}, "time_dimension": {"answer": "Present", "rationale": "The entry is for the purpose of asking the testee about the current behaviour or state of the person, and therefore the judgement is now."}, "sentence_type": "Affirmative declarative sentence", "pos": {"nouns": {"Neck": 1, "The neck": 1, "Red spots": 1}, "adjectives": {"Often": 1}, "numerals": {"122": 1}}}, {"content": "123, I sometimes feel very happy for no reason, even when things are not going well.", "word_count": 25, "label": "", "sentence_structure": "Subject鈥搗erb鈥揷omplement", "subjective_objective": {"answer": "Subjective", "rationale": "Because \"like\" is very subjective."}, "sentence_sentiment": {"answer": "Positive", "rationale": "Because the entry \"Feeling very happy\" expresses a positive emotional attitude."}, "language_style": {"answer": "Tactful and warm", "rationale": "The language style of the entry is more gentle and friendly, is more common and easy to understand, and is not expressed in too cold or direct language, which makes people feel more comfortable and natural. At the same time, the interviewee feels respected and cared for by asking if he/she understands the emotional experience."}, "time_dimension": {"answer": "Present", "rationale": "The entry describes an emotional experience and state of the person being tested, which is not necessarily linked to a particular period of time, but may occur at any time. Therefore, depending on the time dimension, the entry should be \"now\"."}, "sentence_type": "Affirmative declarative sentence", "pos": {"nouns": {"Time": 1, "Happy": 1, "Things": 1}, "verbs": {"I think": 1, "Yes": 1, "Even": 1, "have": 1, "in": 1, "I'll be there": 1, "For no reason": 1, "It's not going well": 1}, "adjectives": {"Very": 1}, "adverbs": {"Sometimes": 1, "very": 1}, "numerals": {"123": 1}, "pronouns": {"I": 1}}}, {"content": "124. No one cares about what happened to anyone.", "word_count": 9, "label": "", "sentence_structure": "Subject鈥搗erb鈥搊bject", "subjective_objective": {"answer": "Objective", "rationale": "Because \"no one cares about what happens to them\" is a description of the facts, not of the individual's feelings or attitudes."}, "sentence_sentiment": {"answer": "Negative", "rationale": "Because the act \"no one cares about what happens to no one\" is a sign of indifference and ruthlessness and a negative emotional tendency."}, "language_style": {"answer": "Calm and direct", "rationale": "The entry is more direct, concise, has no excessive emotional rendering or speech skills, and is more objective and calm. It also directly expresses the question of whether the other person is interested in the subject."}, "time_dimension": {"answer": "Past", "rationale": ""}, "sentence_type": "Negative declarative sentence", "pos": {"nouns": {"Who": 3, "Problems": 1}, "verbs": {"Care": 1, "Problems": 1}, "adverbs": {"Yeah": 1, "not": 2}, "numerals": {"124": 1}, "pronouns": {"Who": 3}}}, {"content": "125, I'm sometimes energetic.", "word_count": 7, "label": "", "sentence_structure": "Subject鈥搗erb鈥揷omplement", "subjective_objective": {"answer": "Subjective", "rationale": "Because \"absorbing\" is a subjective feeling and experience."}, "sentence_sentiment": {"answer": "Neutral", "rationale": "Because the entry describes a factual situation and does not express positive or negative emotional sentiments."}, "language_style": {"answer": "Tactful and warm", "rationale": "The entry is more personal and interactive when it is spoken in a gentle and inviting manner, rather than in direct statements or orders. At the same time, the language style of the article is more concise, with no excessive rhetorical or emotional accent, and more focused on getting the real thoughts and feelings of the other party."}, "time_dimension": {"answer": "Present", "rationale": "The keyword \"sometimes energetic\" suggests that it is a description of the current state, and therefore the present state of affairs."}, "sentence_type": "Affirmative declarative sentence", "pos": {"verbs": {"have": 1, "Time": 1}, "adjectives": {"- I'm very energetic": 1}, "adverbs": {"Sometimes": 1}}}, {"content": "126, I have one or two numbs on my skin.", "word_count": 12, "label": "", "sentence_structure": "Subject鈥揷opula鈥損redicative", "subjective_objective": {"answer": "Objective", "rationale": "Because numbing is a physical condition that can be observed and measured, it is objective."}, "sentence_sentiment": {"answer": "Negative", "rationale": "Because the \"smoking\" feeling of discomfort in the entry is negative."}, "language_style": {"answer": "Calm and direct", "rationale": "The entry is more directly written, has no excessive rhetorical or emotional overturning, and presents a fact in an objective and calm manner. This way allows the person surveyed to answer the question more accurately and reduces misunderstandings and deviations."}, "time_dimension": {"answer": "Present", "rationale": "According to the sentence entered, \"I have one or two numbs on my skin,\" the questionnaire entry is asking the person's current state of health, so it can be judged to be the time dimension of this entry."}, "sentence_type": "Affirmative declarative sentence", "pos": {"nouns": {"Skin": 1, "One or two": 1, "I'm numb": 1}, "verbs": {"have": 1, "I'm numb": 1}, "numerals": {"Twelve six": 1, "One or two": 2}, "classifiers": {"Location": 1}, "pronouns": {"I": 1}}}, {"content": "127, someone controls my mind.", "word_count": 9, "label": "", "sentence_structure": "Subject鈥搗erb鈥搊bject", "subjective_objective": {"answer": "Subjective", "rationale": "Because \"control\" is the patient's own feelings and perceptions, and it's a subjective approach."}, "sentence_sentiment": {"answer": "Negative", "rationale": "Because the expression \"someone controls my mind\" in the entry is negative emotional, suggesting that the subject may be subject to some form of psychological manipulation or control."}, "language_style": {"answer": "Tactful and warm", "rationale": "The language style of the entry is euphemistic. Although the term \"control\" is mentioned, it does not use strong derogatory or accusatory language. Moreover, quotation marks have been added to the title, making the sentence more subjective and enabling respondents to express their thoughts and feelings better."}, "time_dimension": {"answer": "Present", "rationale": ""}, "sentence_type": "Negative declarative sentence", "pos": {"nouns": {"person": 1, "Ideas": 1}, "verbs": {"Controlled": 1}, "numerals": {"127": 1}}}, {"content": "I often feel like some strangers are staring at me with a picky eye.", "word_count": 19, "label": "", "sentence_structure": "Subject鈥搗erb鈥揷omplement", "subjective_objective": {"answer": "Subjective", "rationale": "Because it's a subjective feeling."}, "sentence_sentiment": {"answer": "Negative", "rationale": "Because the feeling in the entry that \"I feel strangers picking on myself\" is negative."}, "language_style": {"answer": "Tactful and warm", "rationale": ""}, "time_dimension": {"answer": "Present", "rationale": ""}, "sentence_type": "Affirmative declarative sentence", "pos": {"nouns": {"View": 1, "Stranger": 1, "Entry": 1}, "verbs": {"I think": 1, "Keep an eye on it": 1, "Often": 1}, "adjectives": {"Picky": 1}, "numerals": {"128": 1}, "pronouns": {"I": 1}}}, {"content": "129, I usually love my family and occasionally hate them.", "word_count": 13, "label": "", "sentence_structure": "Subject鈥搗erb鈥揷omplement", "subjective_objective": {"answer": "Subjective", "rationale": "Because \"love\" and \"hate\" are patients' own emotional experiences, subjective perspectives."}, "sentence_sentiment": {"answer": "Neutral", "rationale": "Because the entry describes the frequency of personal emotional change, there is no apparent emotional tendency."}, "language_style": {"answer": "Tactful and warm", "rationale": "The entry contains a dual emotional description of family members, both love and hate. But the overall tone is relatively mild and not too emotional, so it can be judged as a soft and warm language style."}, "time_dimension": {"answer": "Past", "rationale": "Past."}, "sentence_type": "Affirmative declarative sentence", "pos": {"nouns": {"Home": 1, "person": 1}, "verbs": {"Love": 1, "Hate": 1, "Usually": 1, "Yeah": 1, "Sometimes": 1}, "adverbs": {"Usually": 1, "Sometimes": 1}, "numerals": {"129": 1}, "pronouns": {"I": 1}}}, {"content": "130, I'm sure someone's talking about me.", "word_count": 10, "label": "", "sentence_structure": "Subject鈥揷opula鈥損redicative", "subjective_objective": {"answer": "Subjective", "rationale": "This is because of the patient ' s self-perception and conviction and is subjective."}, "sentence_sentiment": {"answer": "Negative", "rationale": "Because the expression \"responsible\" and \"describe me\" have negative emotional connotations."}, "language_style": {"answer": "Tactful and warm", "rationale": "The language style of the entry is more moderate and euphemistic, and it is not too direct or strong, but rather a calm and understanding approach to guide the interviewee to express his feelings and ideas."}, "time_dimension": {"answer": "Present", "rationale": "\"I am sure\" in \"convinced\" indicates the current beliefs and attitudes, and \"is\" indicates that the conduct in progress points to the present situation. So the temporal dimension of the article is now."}, "sentence_type": "Affirmative declarative sentence", "pos": {"nouns": {"person": 1, "Arguments": 1}, "verbs": {"Sure": 1, "In the process of": 1, "have": 1}, "adverbs": {"In the process of": 1}, "numerals": {"130": 1, "others": 1}, "pronouns": {"I": 1}}}, {"content": "At least once or twice in my life, I felt that I had been prompted to do something by implication.", "word_count": 27, "label": "", "sentence_structure": "Subject鈥搗erb鈥搊bject", "subjective_objective": {"answer": "Subjective", "rationale": "Because \"I think\" means that it's a subjective feeling or a perception, not an objective fact."}, "sentence_sentiment": {"answer": "Neutral", "rationale": "Because the entry does not express any emotional colour, it merely presents a fact."}, "language_style": {"answer": "Tactful and warm", "rationale": "The language style of the entry is more euphemistic. It starts with the first person's name, \"I\" to make the interviewer feel more close and natural. Moreover, it uses \"I think\" as a more subjective expression, without directly accusing or criticizing others. This gentle and intimate tone makes it easier for the interviewee to share his true feelings."}, "time_dimension": {"answer": "Past", "rationale": "\"at least once or twice\" indicates that the person tested has experienced such experiences in the past, and therefore the entire sentence is focused on the past."}, "sentence_type": "Affirmative declarative sentence", "pos": {"nouns": {"Life": 1, "Number of times": 2, "person": 1, "Things": 1}, "verbs": {"I think": 1, "鈾?And I'm gonna be 鈾?: 1, "Do it": 1}, "adverbs": {"At least": 1}, "numerals": {"One": 2, "Two": 1}, "pronouns": {"I": 1}}}, {"content": "132, someone's been trying to influence my mind.", "word_count": 12, "label": "", "sentence_structure": "Subject鈥搗erb鈥搊bject", "subjective_objective": {"answer": "Subjective", "rationale": "Because \"want to influence\" is the patient's subjective expectations and feelings for others."}, "sentence_sentiment": {"answer": "Negative", "rationale": "Because the act of \"someone has always wanted to influence my mind\" in the entry may be a matter of control or unease and a negative emotional colour."}, "language_style": {"answer": "Calm and direct", "rationale": "The entry directly expresses the subject of an inquiry into whether the other party has attempted to influence his mind, without excessive rhetoric or emotional rendering, and is more objective in tone."}, "time_dimension": {"answer": "Present", "rationale": "Now the entry describes what the subject is now going through, and the keyword is \"someone has always wanted\" and \"impact\", which all indicate that this is an ongoing act."}, "sentence_type": "Negative declarative sentence", "pos": {"nouns": {"person": 1, "Ideas": 1}, "verbs": {"Impact": 1, "Yes": 1}, "numerals": {"132": 1, "Always": 1}, "pronouns": {"Mine": 1}}}, {"content": "133. Life is a lot of work for me.", "word_count": 17, "label": "", "sentence_structure": "Subject鈥揷opula鈥損redicative", "subjective_objective": {"answer": "Subjective", "rationale": "Because it reflects the feelings and experiences of the patient, and it is subjective.\""}, "sentence_sentiment": {"answer": "Negative", "rationale": "Because the phrase \"life is a hard thing for me\" in the entry conveys negative emotions and attitudes."}, "language_style": {"answer": "Calm and direct", "rationale": "The language style of the entry is relatively calm and straightforward, rather than over-utilizing or rendering, it is the simple and clear language that makes the issue clearer."}, "time_dimension": {"answer": "Present", "rationale": "\"Many times\" suggests that this is a recurrent situation and that it is a general state of affairs, which means that it still exists. Thus, the entry asks the testee about the current feelings and behaviour, and the temporal dimension is now judged."}, "sentence_type": "Affirmative declarative sentence", "pos": {"nouns": {"Time": 1, "Life": 1, "Yes": 1}, "verbs": {"to": 1, "be": 1, "Tell me": 1, "Come on": 1}, "adjectives": {"Many": 1}, "numerals": {"133": 1}, "pronouns": {"I": 1}}}, {"content": "134, I'm sensitive to something so I can't bring it up.", "word_count": 18, "label": "", "sentence_structure": "Subject鈥揷opula鈥損redicative", "subjective_objective": {"answer": "Subjective", "rationale": "Because the words \"sensitive\" and \"not able to mention\" in the sentence are the subjective feelings and emotional reactions of the patient."}, "sentence_sentiment": {"answer": "Negative", "rationale": "Because \"sensitization\" and \"not to mention\" in the entries are negative emotional expressions."}, "language_style": {"answer": "Tactful and warm", "rationale": "The entry uses a more euphemistic language to avoid directly speaking the problems or shortcomings that the other party may have. At the same time, language style is a certain comfort and understanding that makes people feel respected and concerned. This expression is more friendly and acceptable."}, "time_dimension": {"answer": "Past", "rationale": "The keywords \"sensitive\" and \"not able to bring up\" describe the past, implying that the person tested had felt this at some point in the past."}, "sentence_type": "Negative declarative sentence", "pos": {"nouns": {"Things": 1, "Sensitive": 1}, "verbs": {"鈾?And I'm gonna be 鈾?: 1, "T": 1, "Rise": 1}, "adjectives": {"very": 1, "Some": 1}, "adverbs": {"And so": 1}, "numerals": {"134": 1, "1": 2}, "pronouns": {"I": 1}}}, {"content": "I'm often lonely even with people.", "word_count": 17, "label": "", "sentence_structure": "Subject鈥搗erb鈥揷omplement", "subjective_objective": {"answer": "Subjective", "rationale": "Because \"often feeling alone\" is a subjective feeling."}, "sentence_sentiment": {"answer": "Negative", "rationale": "Because the \"often feeling lonely\" sentiment in the entry is negative."}, "language_style": {"answer": "Tactful and warm", "rationale": "The entry is more personal and interactive when it is spoken in a gentle and inviting manner, rather than in direct statements or orders. It also expresses the subject of the potential for isolation on the other side, which is more objective in tone."}, "time_dimension": {"answer": "Present", "rationale": ""}, "sentence_type": "Negative declarative sentence", "pos": {"nouns": {"People": 1, "Alone": 1}, "verbs": {"in": 1, "I feel": 1, "Together": 1}, "adjectives": {"Regular": 1}, "adverbs": {"Or is it": 1, "Regular": 1}, "numerals": {"135": 1}, "pronouns": {"I": 1}}}, {"content": "136, I don't like people around me.", "word_count": 10, "label": "", "sentence_structure": "Subject鈥搗erb鈥揷omplement", "subjective_objective": {"answer": "Subjective", "rationale": "Because \"not like\" is patient's subjective attitude."}, "sentence_sentiment": {"answer": "Negative", "rationale": "Because \"not like\" in the entry is a negative emotional attitude."}, "language_style": {"answer": "Calm and direct", "rationale": "The language style of the entry is more straightforward, there is no excessive emotional qualification or euphemism, and the tone is more objective, with the aim of allowing the person under investigation to express his or her attitude accurately."}, "time_dimension": {"answer": "Present", "rationale": "According to the phrase \"not like,\" it can be established that the emotional attitude of the article is present in the feelings or emotions of the moment. Moreover, the word \"yes\" indicates that the feeling is caused by someone or some person being around the person being tested. So the entry asks about the person being tested's feelings and emotional experiences."}, "sentence_type": "Negative declarative sentence", "pos": {"nouns": {"person": 1, "鈾?With me 鈾?: 1}, "verbs": {"Yeah": 1, "in": 1}, "numerals": {"136": 1}, "pronouns": {"I": 1}}}, {"content": "137, I'm more sensitive than most people.", "word_count": 9, "label": "", "sentence_structure": "Subject鈥揷opula鈥損redicative", "subjective_objective": {"answer": "Subjective", "rationale": "Because \"more sensitive than most people\" is a personal feeling and perception, a subjective assessment."}, "sentence_sentiment": {"answer": "Negative", "rationale": "Because the \"sensitive\" feature in the entry is usually considered negative."}, "language_style": {"answer": "Tactful and warm", "rationale": ""}, "time_dimension": {"answer": "Present", "rationale": ""}, "sentence_type": "Affirmative declarative sentence", "pos": {"nouns": {"137": 1, "person": 1, "Sensitive": 1}, "verbs": {"- Yeah": 1, "More": 1, "Sensitive": 1}, "adjectives": {"Large": 1, "Most": 1}, "numerals": {"137": 1, "More": 1}, "classifiers": {"person": 1}, "pronouns": {"I": 1}}}, {"content": "138, I can easily feel overwhelmed.", "word_count": 10, "label": "", "sentence_structure": "Subject鈥搗erb鈥揷omplement", "subjective_objective": {"answer": "Subjective", "rationale": "Because \"frightful\" is a personal feeling and experience, a subjective perspective."}, "sentence_sentiment": {"answer": "Negative", "rationale": "Because \"unsure\" means a negative emotional state."}, "language_style": {"answer": "Calm and direct", "rationale": "The entry directly expresses the question whether the other party often feels overwhelmed by the subject, does not use excessive rhetoric or emotional rendering, and is more objective in tone."}, "time_dimension": {"answer": "Present", "rationale": "The entry asks the person's current psychological state, using the verb \"easy to feel\" which indicates that it is a feeling or behaviour that is going through. Thus, the temporal dimension is now judged."}, "sentence_type": "Affirmative declarative sentence", "pos": {"nouns": {"138": 1, "I": 1, "Grandpa": 1, "I don't know what to do": 1}, "adjectives": {"Easy": 1}, "numerals": {"138": 1}, "classifiers": {"Grandpa": 1}, "pronouns": {"I": 1}}}, {"content": "My mother or father often asked me to obey him, even if I did not think it was reasonable.", "word_count": 24, "label": "", "sentence_structure": "Subject鈥搗erb鈥搊bject", "subjective_objective": {"answer": "Subjective", "rationale": "Because the entry describes the subjective feeling of the patient to his parents demanding that he or she obey them. This feeling is subjective, because it relates to the emotions and perceptions of the individual, not just to objective facts."}, "sentence_sentiment": {"answer": "Negative", "rationale": "Because the entry \"always asked me to obey him, even if I thought it was unreasonable\" expressed a negative emotion and attitude."}, "language_style": {"answer": "Calm and direct", "rationale": "The language style of the questionnaire entry is relatively calm and straightforward, and it does not use too many emotional qualifiers, but rather expresses a clear view: the mother or father of the interviewee sometimes asks them to obey something they think is unreasonable. This expression is simple and clear, and it makes it easier for the interviewee to understand the implications of the problem accurately."}, "time_dimension": {"answer": "Past", "rationale": ""}, "sentence_type": "Affirmative declarative sentence", "pos": {"nouns": {"I": 2, "Mother": 1, "Father": 1, "Obey": 1, "It doesn't make sense": 1}, "verbs": {"Yes": 1, "I think": 1}, "adjectives": {"Often": 1}, "numerals": {"139": 1}}}, {"content": "140, I sometimes can't change my voice, even if I don't catch a cold.", "word_count": 22, "label": "", "sentence_structure": "Subject鈥搗erb鈥揷omplement", "subjective_objective": {"answer": "Subjective", "rationale": "This is a description of the patient ' s subjective feelings and is therefore subjective."}, "sentence_sentiment": {"answer": "Neutral", "rationale": "Because the entry describes an objective fact, there is no apparent emotional tendency."}, "language_style": {"answer": "Tactful and warm", "rationale": ""}, "time_dimension": {"answer": "Present", "rationale": "The entry now describes the occasional difficulty of the person being tested in speaking in the absence of a cold, which suggests that this is a present phenomenon, not a past experience or future expectation."}, "sentence_type": "Affirmative declarative sentence", "pos": {"nouns": {"140": 1, "Cold": 1, "Sound": 3, "Change": 1}, "verbs": {"Let's go": 1, "Out": 1, "Change": 1}, "adverbs": {"Sometimes": 1}, "numerals": {"140": 1, "1": 3}, "pronouns": {"I": 2}}}, {"content": "141, sometimes I smell strange.", "word_count": 11, "label": "", "sentence_structure": "Subject鈥搗erb鈥揷omplement", "subjective_objective": {"answer": "Objective", "rationale": "The basis for the judgement: The entry describes a perceived phenomenon that the patient may experience, namely, \"smelt strange smell,\" which is an objective fact. Although this perception may affect the patient, it is not subjective or thought in itself, and is therefore an objective examination."}, "sentence_sentiment": {"answer": "Neutral", "rationale": "The entry is not a positive or negative emotional expression."}, "language_style": {"answer": "Calm and direct", "rationale": "The entry is more calm and objective in its language, without too many emotional qualifiers or rhetorical techniques. It presents a direct observation and asks the interviewee whether he has had a similar experience, is plain and personal."}, "time_dimension": {"answer": "Present", "rationale": ""}, "sentence_type": "Affirmative declarative sentence", "pos": {"nouns": {"Smell": 1}, "verbs": {"Yes": 1, "Smell it": 1}, "adjectives": {"Strange": 1}, "numerals": {"141": 1}}}, {"content": "I can't concentrate on one thing.", "word_count": 11, "label": "", "sentence_structure": "Subject鈥搗erb鈥揷omplement", "subjective_objective": {"answer": "Subjective", "rationale": "Because \"I can't concentrate on one thing\" is the patient's self-perception and subjective evaluation, reflecting his psychological state and emotional experience."}, "sentence_sentiment": {"answer": "Negative", "rationale": "Because the \"can't focus on one thing\" act in the entry is negative."}, "language_style": {"answer": "Calm and direct", "rationale": "The language style of the entry is more straightforward, with no excessive adoration or emotional colour, and the question of whether the other party is under-focused is expressed in a concise manner."}, "time_dimension": {"answer": "Present", "rationale": "The entry describes the current state and experience of the testee, and does not address the temporal dimensions of the past or the future. The keyword \"not to concentrate\" indicates that this is an ongoing act or state, and therefore the judgement is now."}, "sentence_type": "Negative declarative sentence", "pos": {"verbs": {"Focus": 1, "in": 1}, "adjectives": {"One": 1, "Things": 1, "Go, go, go": 1}, "numerals": {"142": 1}}}, {"content": "143, I'm easily impatient.", "word_count": 11, "label": "", "sentence_structure": "Subject鈥搗erb鈥揷omplement", "subjective_objective": {"answer": "Subjective", "rationale": "Because \"easy to be impatient\" is the patient's subjective feeling and attitude."}, "sentence_sentiment": {"answer": "Negative", "rationale": "Because the attitude of \"intolerantness\" in the entry is negative."}, "language_style": {"answer": "Calm and direct", "rationale": "The language style of the entry is more objective and straightforward, and there is no excessive rhetorical or emotional rendering. It is a simple expression of questions about a sexual characteristic, and it is flat and unsettling, giving people a sense of calm and decisiveness."}, "time_dimension": {"answer": "Present", "rationale": ""}, "sentence_type": "Negative declarative sentence", "pos": {"nouns": {"person": 1, "Questionnaire": 1, "Entry": 1, "Sensor": 1, "I'm impatient": 1}, "verbs": {"to": 1, "I feel": 1, "be": 1}, "adjectives": {"very": 1}, "adverbs": {"Easy": 1}, "numerals": {"143": 1}}}, {"content": "144, I've been anxious about something or someone almost all day.", "word_count": 18, "label": "", "sentence_structure": "Subject鈥搗erb鈥揷omplement", "subjective_objective": {"answer": "Subjective", "rationale": "Because \"the whole day is anxious for something or someone\" is the patient's inner feeling and experience, reflecting their emotional state and psychological stress. This is seen from the patient's subjective perspective."}, "sentence_sentiment": {"answer": "Negative", "rationale": "Because the \"suspering for something or someone\" act in the entry is negative."}, "language_style": {"answer": "Calm and direct", "rationale": ""}, "time_dimension": {"answer": "Present", "rationale": "In that sentence, \"daytime\" means a period of time, a state of the moment, and therefore a judgment of the moment. And the \"growing for something or someone\" state is an ongoing and a manifestation of the current state of affairs."}, "sentence_type": "Affirmative declarative sentence", "pos": {"nouns": {"Oh, my God": 1, "Yes": 1, "person": 1}, "verbs": {"in": 1, "Tight": 1, "Yes": 2, "And": 1, "- It's urgent": 1}, "adjectives": {"Almost": 1}, "adverbs": {"All day long": 1}, "numerals": {"144": 1}, "pronouns": {"I": 1}}}, {"content": "And that which I worry is far greater than that which I should.", "word_count": 18, "label": "", "sentence_structure": "Subject鈥搗erb鈥搊bject", "subjective_objective": {"answer": "Subjective", "rationale": "Because \"what I'm concerned about\" and \"what I should be concerned about\" are comparisons from the patient's subjective point of view."}, "sentence_sentiment": {"answer": "Negative", "rationale": "Because \"the worry is more than what should be concerned\" in the entry expresses an attitude of excessive anxiety and anxiety, which is a negative emotional colour."}, "language_style": {"answer": "Tactful and warm", "rationale": "Warm and warm."}, "time_dimension": {"answer": "Present", "rationale": "\"I'm worried about\" in \"what I'm concerned\" means what is happening, and \"well, far beyond\" means that it is going beyond the right range, implying that there may be situations of excessive anxiety or anxiety. So, this entry relates to the state of emotion and mental state."}, "sentence_type": "Negative declarative sentence", "pos": {"nouns": {"Yes": 2, "Mind your own business": 2, "Grandpa": 1}, "verbs": {"Mind your own business": 2, "Over": 1}, "adjectives": {"Far beyond that": 1}, "numerals": {"145": 1, "All that should worry": 1}, "pronouns": {"I": 1, "Sou-san": 3}}}, {"content": "146. Sometimes my hearing is too sensitive, but it bothers me.", "word_count": 18, "label": "", "sentence_structure": "Subject鈥揷opula鈥損redicative", "subjective_objective": {"answer": "Subjective", "rationale": "Because \"fearing\" is the patient's feeling and experience, and it's considered from the patient's subjective point of view."}, "sentence_sentiment": {"answer": "Negative", "rationale": "The article is a source of emotional distress."}, "language_style": {"answer": "Tactful and warm", "rationale": "The entry is relatively euphemistic and gentle, without direct criticism or criticism. It asks the respondents, in a caring and understanding manner, whether they are sometimes troubled by the sensitivity of their hearing, which is more likely to resonate with and support from the respondents and to contribute to a good questionnaire-based atmosphere."}, "time_dimension": {"answer": "Present", "rationale": "Now the entry describes the experience and the experience that the testees are feeling, \"sometimes\" indicating that this feeling is not always present, but that it is haunting them at this particular moment. The keyword \"almost acoustic, but rather agitating me\" also shows that it is the experience of the testees, not the past or the future."}, "sentence_type": "Affirmative declarative sentence", "pos": {"nouns": {"Hearing": 1, "Smart": 1, "Trouble": 1}, "verbs": {"鈾?And I'm gonna be 鈾?: 1, "I feel": 1, "Too much": 1, "Reactions": 1, "Yes": 1}, "adjectives": {"Sometimes": 1, "On the contrary": 1, "鈾?Make me 鈾?: 1}, "numerals": {"146": 1}}}, {"content": "I forgot what I was told.", "word_count": 15, "label": "", "sentence_structure": "Subject鈥損redicate", "subjective_objective": {"answer": "Objective", "rationale": "Because \"forget it right away\" is an objective expression of the patient, describing his ability to remember what he says to others."}, "sentence_sentiment": {"answer": "Neutral", "rationale": "As the entry describes an objective fact, it does not contain a clear emotional tendency."}, "language_style": {"answer": "Calm and direct", "rationale": "The basis for the determination: - The entry is based on a simple and straightforward language structure, with no excessive rhetoric or emotional rendering. - The question is simply whether the interviewee is easy to forget what he/she says, and the language is clear."}, "time_dimension": {"answer": "Present", "rationale": "The keyword \"forgetth\" describes a customary, recurrent act, that is, to forget what others say to themselves immediately. Thus, the entry is a present act or mentality."}, "sentence_type": "Affirmative declarative sentence", "pos": {"nouns": {"person": 1, "Word": 1}, "verbs": {"to": 1, "Say it": 1, "Forget it": 1}, "adverbs": {"Now": 1}, "numerals": {"147": 1}}}, {"content": "I will do it again, even if it is trivial.", "word_count": 18, "label": "", "sentence_structure": "Subject鈥搗erb鈥搊bject", "subjective_objective": {"answer": "Subjective", "rationale": "This is a subjective approach, since it is a way of thinking and decision-making process for patients."}, "sentence_sentiment": {"answer": "Neutral", "rationale": "Because the entry is merely a description of the behaviour, and there is no obvious emotional colour."}, "language_style": {"answer": "Calm and direct", "rationale": "The entry is more straightforward in language style, with no qualifying vocabulary or emotional expression. It simply expresses the view that the person under investigation is going to think in depth when dealing with things, without too much emotional or subjective judgment.\""}, "time_dimension": {"answer": "Past", "rationale": "The sentence contains the word \"will\" and refers to a pattern or customary behaviour, using the past pattern of \"repeated consideration\" and therefore judgement."}, "sentence_type": "Affirmative declarative sentence", "pos": {"nouns": {"It's a little bit of a thing": 1, "Consider": 2}, "verbs": {"Do it": 1, "be": 1}, "adjectives": {"Tiny": 1, "Little": 1}, "adverbs": {"Even": 1, "Three": 1}, "numerals": {"148": 1}, "classifiers": {"individual": 1}}}, {"content": "149, I often feel like nothing is true.", "word_count": 14, "label": "", "sentence_structure": "Subject鈥揷opula鈥損redicative", "subjective_objective": {"answer": "Subjective", "rationale": "Because \"often\" and \"as if nothing is true\" are described from the patient's subjective point of view."}, "sentence_sentiment": {"answer": "Negative", "rationale": "Because the expression \"all is not true\" in the entries is pessimistic and negative."}, "language_style": {"answer": "Calm and direct", "rationale": "The language style of the entry is a calm and straightforward type, as it uses direct statements to ask the person under investigation about his or her feelings and thoughts, and does not use excessive emotional accommodations or rhetorical techniques."}, "time_dimension": {"answer": "Present", "rationale": "Based on the word \"normal\" in the sentence and the general current \"perception\", the entry asks about the current emotional state or feelings of the person being tested, and the temporal dimension judges it as now."}, "sentence_type": "Affirmative declarative sentence", "pos": {"nouns": {"Questionnaire Entry": 1, "Everything": 1}, "verbs": {"I think": 1, "be": 1, "No, it's not": 1}, "adjectives": {"Really": 1}, "adverbs": {"Often": 1}, "numerals": {"149銆?: 1}}}, {"content": "150, mostly, I think I'm better off dead.", "word_count": 15, "label": "", "sentence_structure": "Subject鈥搗erb鈥揷omplement", "subjective_objective": {"answer": "Subjective", "rationale": "Because the sentence contains \"I feel\" that it's a patient's subjective idea or feeling."}, "sentence_sentiment": {"answer": "Negative", "rationale": "Because the expression \"I feel better I'm dead\" is a pessimism and a negative emotional attitude."}, "language_style": {"answer": "Tactful and warm", "rationale": "The entry uses metaphoric terms to convey the pessimism of the interviewee's state, though heavy, but does not directly express a strong negative feeling, but rather to channel the interviewer to share his feelings in a euphemism. Thus, it can be considered a more euphemism and gentle way of asking questions."}, "time_dimension": {"answer": "Past", "rationale": ""}, "sentence_type": "Negative declarative sentence", "pos": {"nouns": {"Time": 1, "I": 1, "Die": 1}, "verbs": {"I think": 1, "Okay": 1, "be": 1}, "adjectives": {"Large": 1, "Time": 1}, "numerals": {"150": 1}}}, {"content": "I'm afraid those who were too close to me.", "word_count": 13, "label": "", "sentence_structure": "Subject鈥搗erb鈥搊bject", "subjective_objective": {"answer": "Subjective", "rationale": "Because \"preventive\" is the patient's subjective attitude, it means that the patient has a sense of vigilance and prevention for some people."}, "sentence_sentiment": {"answer": "Negative", "rationale": "Because the word \"prevent\" in the entry is a sense of vigilance and suspicion, implying that there may be negative perceptions or feelings of people who are too close."}, "language_style": {"answer": "Tactful and warm", "rationale": ""}, "time_dimension": {"answer": "Past", "rationale": "\"Over-intimate\" and \"intentional\" acts have occurred in the past and may still have had effects."}, "sentence_type": "Affirmative declarative sentence", "pos": {"nouns": {"person": 1}, "verbs": {"Watch out": 1, "to": 2, "That's too much": 1, "Close": 1}, "adjectives": {"It's too much": 1}, "numerals": {"151": 1}, "pronouns": {"I": 1}}}, {"content": "152, I have some strange and special thoughts.", "word_count": 12, "label": "", "sentence_structure": "Subject鈥搗erb鈥搊bject", "subjective_objective": {"answer": "Subjective", "rationale": "Because the strange and special idea is the patient's own idea, which is subjective."}, "sentence_sentiment": {"answer": "Neutral", "rationale": "Because the entry does not have any emotional color."}, "language_style": {"answer": "Tactful and warm", "rationale": "The entry uses the words \"unusual\" and \"special\" but the overall context is mild, without highlighting or criticizing the other party's ideas. At the same time, by asking questions, the other side is given some space for reflection and expression, reflecting some respect and understanding. The language style of the questionnaire entry can therefore be considered as euphemism."}, "time_dimension": {"answer": "Present", "rationale": "The keyword \"some\" indicates that such behaviour is ongoing or exists."}, "sentence_type": "Affirmative declarative sentence", "pos": {"nouns": {"Thinking": 1, "Special": 1, "Strange": 1}, "verbs": {"have": 1}, "adjectives": {"Some": 1, "Strange": 1, "Special": 1}, "numerals": {"152": 1}, "classifiers": {"individual": 1}, "pronouns": {"I": 1}}}, {"content": "153, when I was alone, I heard strange voices.", "word_count": 15, "label": "", "sentence_structure": "Subject鈥揷opula鈥損redicative", "subjective_objective": {"answer": "Subjective", "rationale": "This is a subjective entry, because it concerns the perception and experience of the patient when he is alone, which is a subjective feeling and observation of the individual. Although strange voices may be objective, it is subjective to hear reactions that are real, meaningful or that cause the patient to fear."}, "sentence_sentiment": {"answer": "Negative", "rationale": "Because the description \"hearing strange voices\" in the entry may be scary or uneasy and has a negative emotional connotation."}, "language_style": {"answer": "Calm and direct", "rationale": "The language style of the entry is a calm and direct type. It does not use any rhetorical or emotional rendering, but rather expresses the question in a concise language, allowing the interviewee to understand and answer the question quickly. Moreover, the article is more objective and neutral, and does not have too much emotional colour and is in line with the psychological questionnaire."}, "time_dimension": {"answer": "Present", "rationale": ""}, "sentence_type": "Affirmative declarative sentence", "pos": {"nouns": {"Time": 1, "Sound": 1}, "verbs": {"Yes": 1, "in": 1, "Alone": 1, "Give": 1}, "adjectives": {"Strange": 1}, "numerals": {"153": 1}, "pronouns": {"I": 1}}}, {"content": "I'm not sure when I have to leave my house for a short time.", "word_count": 21, "label": "", "sentence_structure": "Subject鈥揷opula鈥損redicative", "subjective_objective": {"answer": "Subjective", "rationale": "Because \"frightful\" is the feeling and experience of a patient leaving home in the short term, and it is subjective."}, "sentence_sentiment": {"answer": "Negative", "rationale": "Because the entry \"deep\" shows negative attitudes or emotions."}, "language_style": {"answer": "Calm and direct", "rationale": "The entry directly expresses the subject of an emotional query about whether the other party will be disturbed while temporarily leaving the house, without using excessive rhetorical or emotional rendering, which is more objective and balanced."}, "time_dimension": {"answer": "Past", "rationale": "The description of \"I have to leave home for a short time\" shows that the act has been or is happening, while \"I'm going to be confused\" means a sense of immediate emotion and emotional experience, so the entry contains information on the two dimensions of time, past and present."}, "sentence_type": "Affirmative declarative sentence", "pos": {"nouns": {"I": 1, "Time": 1}, "verbs": {"I feel": 1, "I'm not sure I can": 1, "Out": 1, "Short-term departures": 1}, "adjectives": {"I have to": 1, "Short-term": 1, "I'm not sure I can": 1}, "adverbs": {"When": 1}, "numerals": {"154銆?: 1}}}, {"content": "155, I'm afraid of something or someone, though I know they won't hurt me.", "word_count": 23, "label": "", "sentence_structure": "Subject鈥搗erb鈥搊bject", "subjective_objective": {"answer": "Subjective", "rationale": "Because \"fear\" and \"know they won't hurt me\" are the subjective feelings and thoughts inside the patient."}, "sentence_sentiment": {"answer": "Negative", "rationale": "Because the \"fear\" in the entry is negative."}, "language_style": {"answer": "Tactful and warm", "rationale": "The language style of the entry is more euphemismal. It asks the other side, in an understanding and support, whether there are some things or people who are afraid, even though they do not harm the other. This expression is more personal and sympathetic, helping to build better interaction and trust."}, "time_dimension": {"answer": "Present", "rationale": "The entry describes the current state and feelings of the testee, and the keyword \"fear\" and \"know\" all indicate that this is a present emotional experience."}, "sentence_type": "Negative declarative sentence", "pos": {"nouns": {"155": 1, "I": 2, "Some": 1, "Something": 1, "person": 1, "they": 1, "Hurt": 1}, "verbs": {"Scared": 1, "I know": 1, "be": 1, "Yes": 1, "No, I won't": 1, "Hurt": 1}, "adjectives": {"Some": 1}, "adverbs": {"Although": 1}, "numerals": {"155": 1, "One": 3, "Two": 1}, "classifiers": {"individual": 2}, "pronouns": {"I": 2, "they": 1}}}, {"content": "156, I'm afraid of using knives or anything sharp.", "word_count": 15, "label": "", "sentence_structure": "Subject鈥搗erb鈥搊bject", "subjective_objective": {"answer": "Subjective", "rationale": "Fear is the patient's subjective feeling."}, "sentence_sentiment": {"answer": "Negative", "rationale": "Because it describes a feeling of fear and negative emotional colour."}, "language_style": {"answer": "Tactful and warm", "rationale": "The language style of the entry is more euphemistic, using the word \"fear\" to express the fear that may exist in the other side, rather than directly to state whether the other side is afraid. This language style is more caring and understanding, and it is better able to trigger the echoes of the person under investigation."}, "time_dimension": {"answer": "Past", "rationale": "The entry expresses the testee's past behavior or experience, i.e., \"I'm afraid of using knives or anything sharp,\" and therefore judges the past."}, "sentence_type": "Negative declarative sentence", "pos": {"nouns": {"156": 1, "Knife": 1, "Something sharp": 1}, "verbs": {"Scared": 1, "Use": 1}, "numerals": {"156": 1, "1": 1}}}, {"content": "157, I seem to be more difficult to concentrate than others.", "word_count": 13, "label": "", "sentence_structure": "Subject鈥揷opula鈥損redicative", "subjective_objective": {"answer": "Subjective", "rationale": "\"Substantial/objective\" is determined on the basis of the judgement of the question. If the question is about the patient's feelings or opinions, then it is about subjective; if the question is about the facts or the results, then it is objective. In this example, \"I seem to be more difficult to concentrate than others.\" It is about the patient's subjective feelings, and therefore it is subjective."}, "sentence_sentiment": {"answer": "Negative", "rationale": "Because the expression \"more difficult to concentrate than anyone else\" is negative."}, "language_style": {"answer": "Tactful and warm", "rationale": ""}, "time_dimension": {"answer": "Present", "rationale": "\"It seems harder\" in \"it seems\" suggests that it is a subjective feeling or perception, \"it is harder\" in a comparative description, all pointing to the current situation or behaviour. The entry is therefore to ask the testee about the current focus."}, "sentence_type": "Affirmative declarative sentence", "pos": {"nouns": {"Attention": 1, "others": 1, "self": 1}, "verbs": {"Seems like": 1, "- Yeah": 1, "It's harder to concentrate": 1}, "adverbs": {"It's harder": 1}, "numerals": {"157": 1}, "pronouns": {"I": 1}}}, {"content": "158. I have some bad words in my head that are often terrible, but I can't get rid of them.", "word_count": 26, "label": "", "sentence_structure": "Subject鈥搗erb鈥揷omplement", "subjective_objective": {"answer": "Subjective", "rationale": "Because \"some bad words in my head are often terrible words, but I can't get rid of them\" are the thoughts and feelings of the patients themselves, and are therefore subjective."}, "sentence_sentiment": {"answer": "Negative", "rationale": "Because the \"indefinite\" and \"horrible\" descriptions in the entries have negative feelings."}, "language_style": {"answer": "Calm and direct", "rationale": "The language style of the entry is a calm and straightforward one. It directly asks the interviewee whether there are some negative ideas that are difficult to escape, without using too many emotional qualifiers or rhetorical techniques. This direct expression makes it easier for the respondent to understand and respond to the questions, and reduces possible misunderstandings and confusion."}, "time_dimension": {"answer": "Past", "rationale": "In summary, it is not possible to determine, on the basis of the information provided, whether the time dimension of the questionnaire entry is past, present or future."}, "sentence_type": "Affirmative declarative sentence", "pos": {"nouns": {"Brain": 1, "Words": 2, "Horrible": 1, "They": 1}, "verbs": {"Come on": 1, "be": 1, "And": 1, "Cannot": 1, "Get rid of it": 1}, "adjectives": {"Bad": 1}, "adverbs": {"Often": 1, "But": 1}, "numerals": {"158": 1}, "pronouns": {"I": 1}}}, {"content": "159, sometimes, I have been troubled for days by the invisibility of ideas.", "word_count": 24, "label": "", "sentence_structure": "Subject鈥搗erb鈥揷omplement", "subjective_objective": {"answer": "Subjective", "rationale": "The questionnaire is intended to examine the patient ' s perception of his or her own feelings and thoughts and to be a subjective one."}, "sentence_sentiment": {"answer": "Negative", "rationale": "Because the entry \"some irrelevant thoughts haunt me, making me uncomfortable for days\" is negative."}, "language_style": {"answer": "Tactful and warm", "rationale": ""}, "time_dimension": {"answer": "Present", "rationale": "The keywords \"sometimes\" and \"disturbing for many days\" indicate that it is a state of continuity and takes place within the time frame of the testee. Thus, the entry describes the current behavior or mentality."}, "sentence_type": "Affirmative declarative sentence", "pos": {"nouns": {"Thinking": 1, "I": 1, "A few days": 1}, "verbs": {"鈾?Wingings 鈾?: 1, "鈾?And I'm gonna be 鈾?: 1, "I feel": 1, "Unsettled": 1}, "adverbs": {"Sometimes": 1}, "numerals": {"159": 1, "A lot": 1}, "classifiers": {"Some": 1}}}, {"content": "160, almost every day something that scares me.", "word_count": 15, "label": "", "sentence_structure": "Subject鈥搗erb鈥揷omplement", "subjective_objective": {"answer": "Subjective", "rationale": "Fear is a subjective emotional reaction, determined by the patient鈥檚 own feelings and experiences. Although fear may be objective, it is perceived and experienced in a subjective way."}, "sentence_sentiment": {"answer": "Negative", "rationale": "Because the description of \"the thing of fear\" in the entry is negative."}, "language_style": {"answer": "Calm and direct", "rationale": "The entry is more directly linguistically, not reproducing it with too many rhetorical or emotional colours, but asking questions in an objective and neutral manner. This expression is more concise and can enable the respondents to better understand and answer the questions."}, "time_dimension": {"answer": "Present", "rationale": "\"almost daily\" means very high frequency, is an expression of the current state of affairs, and the entry therefore describes the current behaviour or state of the person being tested."}, "sentence_type": "Affirmative declarative sentence", "pos": {"nouns": {"Things": 1, "Come on": 1}, "verbs": {"have": 1, "鈾?And I'm gonna be 鈾?: 1, "Come on": 1}, "adjectives": {"Almost": 1, "Chile": 1, "Scared": 1}, "adverbs": {"Both": 1}, "numerals": {"160": 1}}}, {"content": "161. I was talked about in insulting and indecent terms.", "word_count": 15, "label": "", "sentence_structure": "Subject鈥搗erb鈥搊bject", "subjective_objective": {"answer": "Subjective", "rationale": "Because \"someone talks about me in insulting and indecent terms\" is a personal feeling and experience, a subjective cognitive and emotional reaction of the patient, and a subjective examination."}, "sentence_sentiment": {"answer": "Negative", "rationale": "Because the words \"insulting and indecent\" in the entry are derogatory terms and express negative emotional attitudes."}, "language_style": {"answer": "Calm and direct", "rationale": "The language style of the entry is relatively simple and not too emotional or rhetorical, but simply asking whether the other person has ever been commented on in insulting terms.\""}, "time_dimension": {"answer": "Past", "rationale": "\"Someone\" indicates that there was such a situation at some point or time in the past, and \"meaning me in insulting and indecent terms\" also indicates that this is a description of what happened in the past. So, this entry is about past behaviour."}, "sentence_type": "Negative declarative sentence", "pos": {"nouns": {"person": 1, "Word": 1, "Insulting": 1, "Naughty": 1}, "verbs": {"Use": 1, "Arguments": 1, "By": 1}, "adjectives": {"Nasty": 1, "Insultive": 1}, "numerals": {"161": 1}, "pronouns": {"I": 1}}}, {"content": "162, I'm always upset to be in the house.", "word_count": 10, "label": "", "sentence_structure": "Subject鈥揷opula鈥損redicative", "subjective_objective": {"answer": "Subjective", "rationale": "Because \"uneasy\" is the patient's subjective feeling."}, "sentence_sentiment": {"answer": "Negative", "rationale": "Because the attitude of \"uneasy\" in the entries is negative."}, "language_style": {"answer": "Calm and direct", "rationale": "The entry directly expresses the subject of an inquiry into whether the other party has an internal anxiety, does not use excessive rhetoric or emotional rendering, and is more objective in tone."}, "time_dimension": {"answer": "Present", "rationale": "The entry expresses a continuous emotional experience, which is \"always disturbed,\" implying that this is a situation that has been happening for some time. Thus, the judgement is based on the time dimension of \"now\"."}, "sentence_type": "Negative declarative sentence", "pos": {"nouns": {"The house": 1, "Unsettled": 1}, "verbs": {"Stay": 1, "Sensor": 1, "Present": 1}, "adjectives": {"Total": 1}, "numerals": {"162": 1}}}, {"content": "Sometimes I feel so much trouble.", "word_count": 10, "label": "", "sentence_structure": "Subject鈥搗erb鈥揷omplement", "subjective_objective": {"answer": "Subjective", "rationale": "Because it is a subjective feeling or experience that feels so difficult."}, "sentence_sentiment": {"answer": "Negative", "rationale": "The basis for the judgement is the negative description of \"difficult.\""}, "language_style": {"answer": "Tactful and warm", "rationale": "The language style of the entry is euphemistic, because it uses the word \"sometimes\" to give a more neutral and objective impression, without directly implying or criticizing the other. At the same time, the term \"difficult\" is expressed in a relatively soft, unheated emotional way, consistent with the soft and warm language."}, "time_dimension": {"answer": "Present", "rationale": "The entry now describes a current feeling or state, and the keywords are \"sometimes\" and \"fears,\" which indicate that this feeling is happening at the moment. At the same time, \"fears are difficult\" are also a specific description of the current emotional situation, highlighting the problems and challenges faced by the testees."}, "sentence_type": "Affirmative declarative sentence", "pos": {"nouns": {"Difficulties": 1, "Thick": 1}, "verbs": {"Common sense": 1, "Present": 1}, "adverbs": {"Sometimes": 1}, "numerals": {"163": 1}}}, {"content": "164. Generally, people demand more respect for themselves than they do for others.", "word_count": 27, "label": "", "sentence_structure": "Subject鈥損redicate", "subjective_objective": {"answer": "Subjective", "rationale": "Because the subject is a subject of subjective views and attitudes of individuals."}, "sentence_sentiment": {"answer": "Negative", "rationale": "Because it expresses a negative act, which is \"very little respect for yourself\", which reflects a negative attitude and emotional colour."}, "language_style": {"answer": "Tactful and warm", "rationale": "The purpose of this article is to express itself in a more moderate manner, without direct criticism or criticism, but rather in a way that leads the interviewee to reflect on his or her behaviour. At the same time, the entry also emphasizes the phenomenon of \"generally people demanding that others respect themselves more \" , which makes the language more euphemistic and objective."}, "time_dimension": {"answer": "Past", "rationale": "In this entry, the words \"generally,\" \"requirement\" and \"respect\" all express a description or view of past events. Thus, the temporal dimension is a past."}, "sentence_type": "Affirmative declarative sentence", "pos": {"nouns": {"People": 2, "Request": 1, "others": 3, "Respect": 2, "self": 2}, "verbs": {"Say it": 1, "More": 1, "Less": 1, "Respect": 1}, "adverbs": {"General": 1, "And": 1, "But": 1}, "numerals": {"164": 1}, "pronouns": {"self": 2}}}, {"content": "165. I like social events to be with people.", "word_count": 17, "label": "", "sentence_structure": "Subject鈥搗erb鈥搊bject", "subjective_objective": {"answer": "Subjective", "rationale": "Because \"like social activities\" reflects patients' subjective attitudes and preferences and is a subjective assessment of social activities."}, "sentence_sentiment": {"answer": "Positive", "rationale": "Because the entry indicates that the purpose of the socialization is to interact with people, it is a positive emotion and attitude."}, "language_style": {"answer": "Tactful and warm", "rationale": "The language style of the entry is euphemistic, because it uses the emotional expression of love, \"like\", and expresses its needs in a gentle way, that of wanting to interact with people. This expression is more friendly and interactive, and makes people feel more comfortable and receptive."}, "time_dimension": {"answer": "Present", "rationale": ""}, "sentence_type": "Affirmative declarative sentence", "pos": {"nouns": {"People": 1, "Social events": 1}, "verbs": {"Yeah": 2, "and": 1, "Together": 1}, "numerals": {"165": 1}, "classifiers": {"individual": 1}}}, {"content": "166, I like the crowds.", "word_count": 10, "label": "", "sentence_structure": "Subject鈥搗erb鈥揷omplement", "subjective_objective": {"answer": "Subjective", "rationale": "Because \"like\" is a subjective feeling and attitude of a person."}, "sentence_sentiment": {"answer": "Positive", "rationale": "Because the \"like a crowd of people\" act is positive."}, "language_style": {"answer": "Tactful and warm", "rationale": "The entry is based on a soft and warm language style by asking the other side whether they like the popular scenes, the temperature of the language and respect for the will of the other side."}, "time_dimension": {"answer": "Present", "rationale": "The entry expresses the current attitude and preferences of the testee and therefore the judgement is now."}, "sentence_type": "Affirmative declarative sentence", "pos": {"nouns": {"I": 1, "person": 1, "Event": 1}, "verbs": {"Yeah": 1, "More": 1, "It's hilarious": 1}, "numerals": {"166": 1}, "classifiers": {"individual": 1}}}, {"content": "For some time, I felt so strong that I didn't need to sleep for days.", "word_count": 24, "label": "", "sentence_structure": "Subject鈥搗erb鈥揷omplement", "subjective_objective": {"answer": "Subjective", "rationale": "Because it is the patient ' s subjective feeling, it is also the patient ' s subjective experience whether or not to sleep."}, "sentence_sentiment": {"answer": "Positive", "rationale": "Because the words \"fears strong\" and \"no sleep\" in the entries are positive emotions and behaviors."}, "language_style": {"answer": "Tactful and warm", "rationale": "The entry uses a light and positive tone to ask questions, trying to resonate the memory and feelings of each other's experiences. Using a number of rhetorical techniques (such as \"smoking\" and \"no sleep for days at a time\"), the language is more interesting and interactive with the interviewee."}, "time_dimension": {"answer": "Present", "rationale": "Moreover, \"Esperately, so long as you don't need to sleep for days\" describes an emotional state and physical reaction, which means that the behaviour has not only occurred in the past, but may still be ongoing. So, in terms of time dimensions, the entry can also be classified as current."}, "sentence_type": "Affirmative declarative sentence", "pos": {"nouns": {"Time": 2, "Powerful": 1, "Oh, my God": 1, "Sleep": 1}, "verbs": {"I feel": 1, "Yes": 1}, "adjectives": {"Perfect": 1, "How many": 1}, "numerals": {"167": 1, "One-man": 1}, "classifiers": {"individual": 1}}}, {"content": "168. I like parties and social events.", "word_count": 10, "label": "", "sentence_structure": "Subject鈥搗erb鈥搊bject", "subjective_objective": {"answer": "Subjective", "rationale": "Because \"like\" is the patient's personal emotional attitude, it's a subjective assessment."}, "sentence_sentiment": {"answer": "Positive", "rationale": "Because the act of \"like parties and social events\" is positive."}, "language_style": {"answer": "Tactful and warm", "rationale": "This entry is presented in a more moderate way and leads respondents to express their attitude to social activities by using the active word \"like\"."}, "time_dimension": {"answer": "Present", "rationale": "The key words in the sentence, \"like\" and \"party\" and \"social activity\", describe the present preferences and behaviours of the testee and therefore are part of the current temporal dimension."}, "sentence_type": "Affirmative declarative sentence", "pos": {"nouns": {"Party": 1, "Social events": 1}, "verbs": {"Yeah": 1}, "adverbs": {"and": 1}, "numerals": {"168": 1}}}]}

{"questionnaire_title": "Questionnaire on parenting", "questionnaire_category": "Behavioral questionnaire", "item_count": 66, "emotion_shift_overall": "From positive to negative", "language_style_overall": "Neutral", "items": [{"content": "I feel that my parents interfered with everything I did.", "word_count": 15, "label": "", "sentence_structure": "Subject鈥揷opula鈥損redicative", "subjective_objective": {"answer": "Subjective", "rationale": "Because \"think\" is the patient's subjective feeling or attitude."}, "sentence_sentiment": {"answer": "Negative", "rationale": "Because the description \"Parents interfere with everything I do\" is negative emotional and expresses dissatisfaction and aversion to parental intervention."}, "language_style": {"answer": "Calm and direct", "rationale": ""}, "time_dimension": {"answer": "Present", "rationale": "\"I think the words \"felt,\" \"interfer\" and \"do\" in everything I do mean that it's the sentence that the tester evaluates the situation, so it's \"now.\""}, "sentence_type": "Negative declarative sentence", "pos": {"nouns": {"parents": 2, "Yes": 2}, "verbs": {"Interference": 1, "Do it": 1, "I think": 1}, "adverbs": {"Every piece": 1}, "pronouns": {"I": 2}}}, {"content": "I can feel him through his parents' words and his faces.", "word_count": 19, "label": "", "sentence_structure": "Subject鈥搗erb鈥揷omplement", "subjective_objective": {"answer": "Subjective", "rationale": "Because \"fear\" is the patient's subjective feeling and understanding."}, "sentence_sentiment": {"answer": "Positive", "rationale": "Because the word \"likes\" has a positive emotional color."}, "language_style": {"answer": "Tactful and warm", "rationale": "The entry is more personal and interactive when it is spoken in a gentle and inviting manner, rather than in direct statements or orders. At the same time, the content of the entry is more likely to address subjective feelings, which are consistent with the euphemism of the language."}, "time_dimension": {"answer": "Past", "rationale": "The entry describes the person's current or past behaviour or state, and the keyword \"can\" and \"favorite\" indicates that it is an examination of the person's current perception and understanding, and can therefore be judged as now."}, "sentence_type": "Affirmative declarative sentence", "pos": {"nouns": {"Questionnaire Entry": 1, "parents": 1, "Talk": 1, "Emoticons": 1, "he": 1, "she": 1, "I": 1}, "verbs": {"It's working": 1, "Feel": 1, "Yeah": 1}, "numerals": {"2": 1}}}, {"content": "I'm more fond of my brothers and sisters than my brothers.", "word_count": 14, "label": "", "sentence_structure": "Subject鈥搗erb鈥揷omplement", "subjective_objective": {"answer": "Subjective", "rationale": "Because love is a subjective attitude and evaluation, it is not an objective reality."}, "sentence_sentiment": {"answer": "Neutral", "rationale": "This entry is a comparison of the treatment of oneself and siblings with regard to their parents and does not convey any positive or negative emotional aspects."}, "language_style": {"answer": "Tactful and warm", "rationale": "The entry is more forthcoming and interactive in a more moderate way, asking the person surveyed whether the parents or guardians feel more caring and favouring themselves rather than directly making statements or orders."}, "time_dimension": {"answer": "Present", "rationale": "The keywords are \"by\" and \"by\" \"by\" and \"by \"by\" and they all occur in comparison with the situation of the tested person with his siblings, so the time dimension of the article is now."}, "sentence_type": "Negative declarative sentence", "pos": {"nouns": {"Brothers and sisters": 2, "In terms of": 1, "Pity": 1}, "verbs": {"- Yeah": 1, "Pup": 1}, "numerals": {"3": 1}, "pronouns": {"I": 1}}}, {"content": "I can feel my parents' love for me.", "word_count": 11, "label": "", "sentence_structure": "Subject鈥揷opula鈥損redicative", "subjective_objective": {"answer": "Subjective", "rationale": "Because it is a personal feeling to feel the love of parents, it is a subjective examination."}, "sentence_sentiment": {"answer": "Positive", "rationale": "Because the entry indicates that the love and affection of parents is a positive emotional experience."}, "language_style": {"answer": "Tactful and warm", "rationale": ""}, "time_dimension": {"answer": "Present", "rationale": ""}, "sentence_type": "Affirmative declarative sentence", "pos": {"nouns": {"I": 1, "parents": 1, "Love it": 1}, "verbs": {"I can feel it": 1}, "numerals": {"4": 1}}}, {"content": "5. Even if it was a small mistake, my parents punished me.", "word_count": 14, "label": "", "sentence_structure": "Subject鈥搗erb鈥搊bject", "subjective_objective": {"answer": "Subjective", "rationale": "It is the individual feelings and experiences of the patient that are punished."}, "sentence_sentiment": {"answer": "Negative", "rationale": "Because \"punished\" is usually seen as a negative experience or feeling."}, "language_style": {"answer": "Calm and direct", "rationale": "The language style of the entry is more direct and concise, and there is no excessive emotional or rhetorical approach, but simply a statement of whether the person under investigation was punished by his parents for minor negligence when he was a child. \""}, "time_dimension": {"answer": "Past", "rationale": "The entry asks the testees about their past experience, i.e. whether they have been punished by their parents for their minor faults."}, "sentence_type": "Negative declarative sentence", "pos": {"nouns": {"Error": 1, "parents": 1}, "verbs": {"have": 1, "Punishment": 1, "be": 1}, "adjectives": {"Very small": 1}, "numerals": {"5": 1}, "pronouns": {"I": 1}}}, {"content": "Parents always try to influence me in a way that makes me a remarkable person.", "word_count": 23, "label": "", "sentence_structure": "Subject鈥搗erb鈥揷omplement", "subjective_objective": {"answer": "Subjective", "rationale": "Because it is the patient ' s subjective feeling that parents are trying to influence themselves to become good people."}, "sentence_sentiment": {"answer": "Positive", "rationale": "Because the \"parents try to influence me in a subterranean way, making me a remarkable person\" act and purpose are positive."}, "language_style": {"answer": "Calm and direct", "rationale": "The language style of the entry is a calm and straightforward one. It uses clear and concise language to express the subject, without too much emotional or rhetorical. At the same time, the entry does not use too much passive language, but instead conveys the subject of the investigation directly in active speech.\""}, "time_dimension": {"answer": "Present", "rationale": "The words \"always trying\" and \"silently affecting\" indicate that the act has been and is continuing, so the entry describes the situation now. And \"makes me a superior person\" is a description of what parents expect of me or what I want, which may be needed in the future, but for the time being it does not fall within the realm of the past or the future. So, the time dimension of this questionnaire is mainly the present."}, "sentence_type": "Affirmative declarative sentence", "pos": {"nouns": {"parents": 1, "Smuggle": 1, "Impact": 1, "person": 1}, "verbs": {"Total": 1, "Try": 1, "鈾?And I'm gonna be 鈾?: 1, "Be": 1, "It's a great idea": 1}, "adverbs": {"Total": 1, "Subsistence": 1}, "classifiers": {"individual": 1}}}, {"content": "I think my parents have given me some kind of uniqueness.", "word_count": 18, "label": "", "sentence_structure": "Subject鈥揷opula鈥損redicative", "subjective_objective": {"answer": "Subjective", "rationale": "Because it is a subjective feeling to feel that it is the patient ' s personal view and opinion."}, "sentence_sentiment": {"answer": "Positive", "rationale": "The entry indicates that parents allow the child to be independent and autonomous in certain respects, and is a positive attitude."}, "language_style": {"answer": "Tactful and warm", "rationale": "The entry is a soft, inviting conversation, using subjective expressions like \"I think\" that avoid too direct a expression and allow the interviewee to feel respected and understood. And, at the same time, \"permissiveness to allow me to have something unique\" implies trust and support for the interviewee, with a more euphemismal language."}, "time_dimension": {"answer": "Present", "rationale": "The entry describes a subjective feeling or perception of the subject, which is that the verbs \"like\" and \"permissive\" indicate that the feeling is present, not past or future."}, "sentence_type": "Affirmative declarative sentence", "pos": {"nouns": {"parents": 1, "I": 1, "Where I come from": 1}, "verbs": {"Allow": 1, "have": 1}, "numerals": {"7": 1}}}, {"content": "Parents give me what other siblings don't get.", "word_count": 19, "label": "", "sentence_structure": "Subject鈥搗erb鈥揷omplement", "subjective_objective": {"answer": "Subjective", "rationale": "This is the patient ' s subjective view that his or her parents can provide special treatment for themselves."}, "sentence_sentiment": {"answer": "Negative", "rationale": "Because, while parents can provide some special treatment to their children, this may affect the equality and mutual assistance between siblings and may trigger some negative emotional and competitive psychology."}, "language_style": {"answer": "Tactful and warm", "rationale": "The words \"can't get\" and \"can't get\" are used to be more gentle and euphemistic, avoiding too direct or powerful a narrative to feel respected and understood. At the same time, the interaction with respondents is enhanced by asking about each other's feelings and experiences."}, "time_dimension": {"answer": "Present", "rationale": "According to the sentence, \"What parents can give me to other siblings\" is a current behavior or state, so it can be judged that the entry is about the present situation."}, "sentence_type": "Affirmative declarative sentence", "pos": {"nouns": {"parents": 1, "Brothers and sisters": 1, "Something": 1}, "verbs": {"Jean": 1, "Okay": 1}, "numerals": {"8": 1}}}, {"content": "9. The punishment of my parents is fair and appropriate.", "word_count": 14, "label": "", "sentence_structure": "Subject鈥揷opula鈥損redicative", "subjective_objective": {"answer": "Subjective", "rationale": "Because \"fair\" and \"right\" are the patient's own subjective feelings and judgments."}, "sentence_sentiment": {"answer": "Positive", "rationale": "Because the description \"fair and appropriate\" is a positive recognition of parents and indicates that they can treat their children with just and appropriate punishment, it is positive emotional."}, "language_style": {"answer": "Calm and direct", "rationale": "The entry is asked in a straightforward manner, without excessive rhetoric or emotional tact, and with a more calm and objective language style."}, "time_dimension": {"answer": "Present", "rationale": "The entry describes the current feelings or attitudes of the testee, the key words being \"parents punish me\" and \"fair and appropriate\", all pointing to the present situation."}, "sentence_type": "Affirmative declarative sentence", "pos": {"nouns": {"parents": 2, "I": 1, "Punishment": 1, "Fair": 1, "Appropriate": 1}, "verbs": {"be": 1, "to": 1, "Conduct": 1}, "adjectives": {"Justice": 1, "Appropriate": 1}, "numerals": {"9": 1}}}, {"content": "I think my parents are tough on me.", "word_count": 10, "label": "", "sentence_structure": "Subject鈥揷opula鈥損redicative", "subjective_objective": {"answer": "Subjective", "rationale": "Because it is the subjective feeling and attitude of the patient, it is a subjective view."}, "sentence_sentiment": {"answer": "Negative", "rationale": "Because the description \"draconian\" in the entry is negative."}, "language_style": {"answer": "Calm and direct", "rationale": "The entry raises questions in an objective and direct manner, without any rhetorical or emotional colours to influence the respondent ' s thinking, and therefore the language style is considered to be calm and straightforward."}, "time_dimension": {"answer": "Present", "rationale": ""}, "sentence_type": "Affirmative declarative sentence", "pos": {"nouns": {"parents": 1, "I": 1}, "verbs": {"I think": 1, "to": 1, "very": 1, "Hard": 1}, "adjectives": {"Hard": 1}, "numerals": {"10": 1}}}, {"content": "Parents always decide what I should wear or what I should dress up like.", "word_count": 22, "label": "", "sentence_structure": "Subject鈥搗erb鈥揷omplement", "subjective_objective": {"answer": "Subjective", "rationale": "Because \"always right and left\" and \"what to wear or what to dress\" describe the behaviour of parents from the perspective of the patient, it is the patient's subjective feeling."}, "sentence_sentiment": {"answer": "Negative", "rationale": "Because the \"parents always decide what I should wear or what I should dress\" in the entry is negative and has a control and restraint colour."}, "language_style": {"answer": "Tactful and warm", "rationale": "The language style of the entry is more moderate, with euphemism, without direct orders or criticism. It also expresses a sense of concern and understanding, which makes the interviewee feel concerned and respected."}, "time_dimension": {"answer": "Past", "rationale": ""}, "sentence_type": "Negative declarative sentence", "pos": {"nouns": {"parents": 1, "Clothes": 1, "Get dressed": 1, "Look": 1}, "verbs": {"Always": 1, "Around": 1, "Put it on": 1}, "adverbs": {"The": 2}, "numerals": {"11": 1}}}, {"content": "12. Parents don't allow me to do something other than what other kids can do because they're afraid of me.", "word_count": 28, "label": "", "sentence_structure": "Subject鈥搗erb鈥揷omplement", "subjective_objective": {"answer": "Subjective", "rationale": "This is the patient ' s subjective perception and understanding of his parents, not the objective fact."}, "sentence_sentiment": {"answer": "Negative", "rationale": "Because the behavior in the entry (parents do not allow me to do what other children can do) and the purpose (they fear I will get hurt) are all negative emotional."}, "language_style": {"answer": "Tactful and warm", "rationale": "The language style of the entry tends to be euphemistic and understanding, and attempts to comfort and accommodate the person under investigation. It uses the term \"fear\" to express a sense of concern or concern without directly blaming or criticizing the behaviour of parents. At the same time, the entry provides an open question space to encourage the person under investigation to share their own experiences and feelings."}, "time_dimension": {"answer": "Past", "rationale": ""}, "sentence_type": "Negative declarative sentence", "pos": {"nouns": {"parents": 1, "Some": 1, "Other": 1, "Son": 1, "Things": 2, "they": 1, "self": 1, "Risk": 1}, "verbs": {"Allow": 1, "Do it": 1, "Scared": 1, "Yes": 1}, "adjectives": {"Yeah": 1, "Other": 1}, "numerals": {"12": 1}, "pronouns": {"I": 1}}}, {"content": "When I was a kid, my parents beat me or reprimanded me in front of others.", "word_count": 20, "label": "", "sentence_structure": "Subject鈥搗erb鈥搊bject", "subjective_objective": {"answer": "Subjective", "rationale": "Because \"striking me or scolding me in front of others\" is an objective fact of the patient's memory, but the feeling and effect of being beaten and reprimanded is the patient's subjective experience."}, "sentence_sentiment": {"answer": "Negative", "rationale": "Because \"strike\" and \"criticism\" are negative."}, "language_style": {"answer": "Tactful and warm", "rationale": "The entry is more personal and interactive when it is spoken in a gentle and inviting manner, rather than in direct statements or orders. At the same time, the language style of the entry is more euphemistic, avoiding too violent or stimulating language, which helps respondents to better answer questions and remain calm."}, "time_dimension": {"answer": "Past", "rationale": ""}, "sentence_type": "Affirmative declarative sentence", "pos": {"nouns": {"Time": 1, "parents": 1, "others": 1, "Noodles": 1, "Son": 1, "Fight": 1, "Censorship": 1}, "verbs": {"Tsang": 1, "In front of you": 1, "Fight": 1, "Censorship": 1, "Say it": 1}, "adverbs": {"in": 1, "When I was little": 1}, "numerals": {"13": 1}, "classifiers": {"individual": 1}, "pronouns": {"I": 1}}}, {"content": "Parents always care what I do at night.", "word_count": 13, "label": "", "sentence_structure": "Subject鈥搗erb鈥揷omplement", "subjective_objective": {"answer": "Subjective", "rationale": "Because concern is the subjective attitude of parents."}, "sentence_sentiment": {"answer": "Negative", "rationale": "Because the expression \"always concerned\" in the entry is derogatory and expresses an act of excessive interference and is therefore a negative emotion."}, "language_style": {"answer": "Calm and direct", "rationale": "This entry is based on direct questions, is not based on too much rhetorical or emotional presentation and is more objective in tone."}, "time_dimension": {"answer": "Present", "rationale": "The words \"Always\" and \"What\" in \"Parents\" are always concerned about what I do at night, which means current behaviour or circumstances, so it can be judged that the entry is an inquiry into the present situation of the person being tested."}, "sentence_type": "Declarative sentence", "pos": {"nouns": {"parents": 1, "Attention": 1, "Night": 1, "Fuck": 1}, "verbs": {"Total": 1, "be": 1}, "adverbs": {"very": 1}, "numerals": {"14": 1}, "pronouns": {"I": 1}}}, {"content": "When things go wrong, I feel like my parents are trying to encourage me to get some comfort.", "word_count": 28, "label": "", "sentence_structure": "Subject鈥揷opula鈥損redicative", "subjective_objective": {"answer": "Subjective", "rationale": "Because \"Feel\" is the patient's own perception and understanding, it is viewed from the patient's subjective perspective. Although the parents' behaviour is objective, this entry is mainly an examination of how the patient feels and knows about it."}, "sentence_sentiment": {"answer": "Positive", "rationale": "Because the entry \"Parents are trying to encourage me, to give me some comfort\" is positive."}, "language_style": {"answer": "Tactful and warm", "rationale": "The language style of the entry is euphemism, because it describes the attitude of parents towards their children in a caring, supportive and encouraging way, without directly stating or ordering."}, "time_dimension": {"answer": "Present", "rationale": "Now, the keyword \"can feel\" suggests that it is a sense and a sense of what it is now, and \"to give some comfort\" describes the current situation, so the entry is mainly about the current feelings and circumstances of the subject, which are the dimensions of the time."}, "sentence_type": "Affirmative declarative sentence", "pos": {"nouns": {"Yes": 1, "I": 2, "parents": 1, "Consolation": 1}, "verbs": {"Yes": 2, "鈾?And I'm gonna be 鈾?: 2, "I feel": 1, "in": 1, "Try": 1, "Encourage": 1, "Got it": 1}, "adverbs": {"It's not easy": 1, "Some": 1}, "numerals": {"15": 1}}}, {"content": "Parents are always too worried about my health.", "word_count": 12, "label": "", "sentence_structure": "Subject鈥揷opula鈥損redicative", "subjective_objective": {"answer": "Subjective", "rationale": "Because excessive fear is a subjective judgement, it depends on the person ' s own perception and understanding."}, "sentence_sentiment": {"answer": "Negative", "rationale": "Because \"too much to worry\" is negative."}, "language_style": {"answer": "Tactful and warm", "rationale": "The entry is more personal and interactive when it is spoken in a gentle and inviting manner, rather than in direct statements or orders. Moreover, the article is more neutral and has no excessive emotional colours, and can therefore be judged as a warmer style."}, "time_dimension": {"answer": "Past", "rationale": "In this entry, the \"always over-afraid\" indicates the person who was tested for past behaviour or experience, and therefore judged to be past."}, "sentence_type": "Negative declarative sentence", "pos": {"nouns": {"parents": 1, "That's too much": 1, "Fear": 1, "Health": 1}, "verbs": {"Always": 1, "be": 1}, "adjectives": {"Mine": 1}, "numerals": {"16": 1}, "pronouns": {"I": 1}}}, {"content": "Parents often punish me more than I deserve.", "word_count": 17, "label": "", "sentence_structure": "Subject鈥揷opula鈥損redicative", "subjective_objective": {"answer": "Subjective", "rationale": "Because \"the punishment for me is often more than I deserve\" is the patient's subjective sense and judgment of his own experience."}, "sentence_sentiment": {"answer": "Negative", "rationale": "Because the \"punishment is often more than I deserve\" in this entry is negative."}, "language_style": {"answer": "Calm and direct", "rationale": "The language of the entry is more calm and straightforward, with the emphasis on factual statements, and there is no excessive emotional or rhetorical approach to make it easier for the interviewees to understand and answer questions."}, "time_dimension": {"answer": "Past", "rationale": "The entry describes what happened in the past, and the keywords are \"parents punish me\" and \"often more than I deserve\", all of which point to past behaviour or circumstances."}, "sentence_type": "Negative declarative sentence", "pos": {"nouns": {"parents": 1, "I": 2, "Punishment": 1, "Level": 1}, "verbs": {"to": 1, "More than": 1, "Yes": 1}, "adjectives": {"Often": 1}, "numerals": {"17": 1}}}, {"content": "If I don't do what I say at home, my parents will get mad.", "word_count": 16, "label": "", "sentence_structure": "Subject鈥損redicate", "subjective_objective": {"answer": "Objective", "rationale": "The sentence describes the objective fact that parents are angry when the patient does not follow the instructions of his or her parents at home."}, "sentence_sentiment": {"answer": "Negative", "rationale": "Because the article is \"crazy,\" which is a negative emotional reaction."}, "language_style": {"answer": "Calm and direct", "rationale": "The language style of the entry is more direct, explicit and conveys a link between behaviour and consequences, and uses more objective expressions. There is no excessive emotional rendering or euphemism, so it can be judged as \"cool and direct\"."}, "time_dimension": {"answer": "Present", "rationale": "If I don't listen at home, if I say \"if\" means \"a hypothetical situation, \"not at home\" describes a current or a regular act, and \"will\" uses a general future pattern, indicating that the result may occur in the future, so the entry is a description of present and future behaviour."}, "sentence_type": "Affirmative declarative sentence", "pos": {"nouns": {"Home": 1, "Lee": 1, "Yes": 1, "parents": 2, "Fire": 1}, "verbs": {"in": 1, "Listen": 1, "Let's go": 1}, "numerals": {"18": 1}, "pronouns": {"I": 1}}}, {"content": "If I do something wrong, parents always protest a sad look that gives me a sense of guilt or guilt.", "word_count": 33, "label": "", "sentence_structure": "Subject鈥搗erb鈥搊bject", "subjective_objective": {"answer": "Subjective", "rationale": "Because it's what patients think and feel about their own behavior."}, "sentence_sentiment": {"answer": "Negative", "rationale": "Because the words \"protest\" and \"worried\" in the entries are negative emotional and give people a bad feeling."}, "language_style": {"answer": "Calm and direct", "rationale": "This is a more objective and calm language that is suitable for psychological questionnaires."}, "time_dimension": {"answer": "Present", "rationale": "In the future, the entry describes the circumstances and feelings that the testees may experience in the future, i.e., when they make a mistake, their parents may protest in a sad manner and make them feel guilty or guilty. Therefore, time horizons are judged to be the future."}, "sentence_type": "Affirmative declarative sentence", "pos": {"nouns": {"Yes": 2, "parents": 1, "Protest": 1, "Sad": 1, "Look": 1, "- A sense of guilt": 1, "Guilt": 1}, "verbs": {"Do it": 1, "Wrong": 1, "Always": 1, "Protest": 1, "鈾?And I'm gonna be 鈾?: 1, "have": 1}, "adjectives": {"Sad": 1, "Crime": 1, "Guilt": 1}, "numerals": {"9": 1}, "pronouns": {"I": 1}}}, {"content": "I think parents are hard to get close to.", "word_count": 9, "label": "", "sentence_structure": "Subject鈥揷opula鈥損redicative", "subjective_objective": {"answer": "Subjective", "rationale": "Because \"think\" is the patient's subjective feeling and attitude, it's seen from a subjective perspective."}, "sentence_sentiment": {"answer": "Negative", "rationale": "The basis for the judgement: the description in the entry \"hard to get close\" conveys a negative sentiment, implying that there may be problems of communication or tension between the interviewee and his parents. The emotional color of the article is therefore negative."}, "language_style": {"answer": "Calm and direct", "rationale": "The entry allows the respondents to express their views of parents in a direct question, and the language style is more calm and straightforward, with no excessive adoration or emotional colour."}, "time_dimension": {"answer": "Present", "rationale": "The keywords are \"felt\" and \"hard to get close,\" which describe the feelings and perceptions of the subjects now, so the temporal dimension is now."}, "sentence_type": "Negative declarative sentence", "pos": {"nouns": {"parents": 1, "Hard": 1, "Close": 1}, "verbs": {"I think": 1}}}, {"content": "21. I was embarrassed by the fact that my parents were talking about something I said or done in front of others.", "word_count": 30, "label": "", "sentence_structure": "Subject鈥搗erb鈥搊bject", "subjective_objective": {"answer": "Subjective", "rationale": "Because this is the patient's own subjective experience and feelings."}, "sentence_sentiment": {"answer": "Negative", "rationale": "Because the behavior in the entries, \"Parents in front of others\" and \"Easy\" are negative."}, "language_style": {"answer": "Tactful and warm", "rationale": ""}, "time_dimension": {"answer": "Present", "rationale": ""}, "sentence_type": "Negative declarative sentence", "pos": {"nouns": {"parents": 1, "person": 1, "Front": 1, "Noodles": 1, "others": 1, "Word": 2, "Yes": 2, "- No, no, no": 1, "I": 2, "I feel": 1, "I'm embarrassed": 1}, "verbs": {"in": 1, "Tsang": 1, "Say it": 1, "Do it": 1, "鈾?And I'm gonna be 鈾?: 1}, "adverbs": {"very": 1, "Okay": 1}, "numerals": {"21": 1}, "pronouns": {"Here": 1}}}, {"content": "I think my parents prefer me to my brothers and sisters.", "word_count": 18, "label": "", "sentence_structure": "Subject鈥揷opula鈥損redicative", "subjective_objective": {"answer": "Subjective", "rationale": "This is because patients are subjectively evaluating and feeling the degree of parental preference."}, "sentence_sentiment": {"answer": "Negative", "rationale": "Because the expression \"parents prefer me\" in the entry expresses negative opinions about their brothers and sisters."}, "language_style": {"answer": "Tactful and warm", "rationale": "The language style of the entry is euphemistic, because it uses a question-and-answer approach, allowing interviewees to express their views in a gentle and respectful manner. It also does not use too direct or critical language, but rather uses an open and understanding approach to induce respondents to answer questions. Such an expression can make them more comfortable and relaxed, and thus more willing to share their real feelings and ideas."}, "time_dimension": {"answer": "Past", "rationale": "\"Now\", the sentence expresses the current feelings and views and does not address past or future acts or events."}, "sentence_type": "Affirmative declarative sentence", "pos": {"nouns": {"parents": 1, "I": 1, "Brothers and sisters": 1}, "verbs": {"I think": 1, "More like it": 1, "be": 1, "And": 1, "- Yeah": 1, "of": 3}, "adverbs": {"22": 1}, "numerals": {"22": 1}, "pronouns": {"EMPTY": 1}}}, {"content": "23. Parents are small in meeting my needs.", "word_count": 16, "label": "", "sentence_structure": "Subject鈥揷opula鈥損redicative", "subjective_objective": {"answer": "Subjective", "rationale": "Because \"smugness\" is a subjective assessment that reflects the attitude and feelings of patients about what I need."}, "sentence_sentiment": {"answer": "Negative", "rationale": "Because the expression \"slightly \" in the entry is a derogatory term, which is a negative emotional expression."}, "language_style": {"answer": "Calm and direct", "rationale": "This language style helps respondents to understand the meaning and purpose of the question more clearly and to provide an accurate answer."}, "time_dimension": {"answer": "Present", "rationale": "The word \"now\" in time is used to judge the entry as asking the person to whom it is currently being asked about his or her current feelings and views, so the answer is \"now\"."}, "sentence_type": "Negative declarative sentence", "pos": {"nouns": {"Something": 1, "parents": 1}, "verbs": {"Satisfied": 1, "be": 1, "Here": 1}, "adjectives": {"very": 1, "- You're a little bit of a jerk": 1}, "adverbs": {"very": 1}, "numerals": {"23": 1, "One": 1}, "classifiers": {"individual": 1}, "pronouns": {"I": 1}}}, {"content": "Parents often care about my score.", "word_count": 12, "label": "", "sentence_structure": "Subject鈥搗erb鈥揷omplement", "subjective_objective": {"answer": "Subjective", "rationale": "Because \"care\" represents the attitude and perception of patients towards their parents, and is subjective."}, "sentence_sentiment": {"answer": "Positive", "rationale": "Because the \"parents often care about my score\" in the entry is positive as an expression of parents' concern and support for themselves."}, "language_style": {"answer": "Calm and direct", "rationale": "This expression is clear and direct, and places greater emphasis on the importance attached to the outcome. It can therefore be said that the language style of the entry to the questionnaire is \"direct and emphasising the outcome\"."}, "time_dimension": {"answer": "Present", "rationale": ""}, "sentence_type": "Affirmative declarative sentence", "pos": {"nouns": {"parents": 1, "Score": 1}, "verbs": {"I care": 1, "Access": 1}, "adjectives": {"Often": 1}, "numerals": {"24": 1}, "pronouns": {"I": 1}}}, {"content": "25. If I have a hard feeling of support from my parents,", "word_count": 22, "label": "", "sentence_structure": "Subject鈥搗erb鈥搊bject", "subjective_objective": {"answer": "Subjective", "rationale": "Because \"felt parental support\" is a subjective feeling and a perception of a person."}, "sentence_sentiment": {"answer": "Positive", "rationale": "Because the entry \"can feel support from parents\" expresses a positive emotional attitude."}, "language_style": {"answer": "Tactful and warm", "rationale": "The entry uses a more moderate language style, by expressing the \"if you have difficulties\" scenario and introducing on that basis the positive expectation of \"you can feel the support of your parents\" to make the respondents feel more friendly and natural in their responses. At the same time, this euphemism can also lead to better resonance and emotional communication among the respondents, thereby improving the effectiveness and reliability of the questionnaire."}, "time_dimension": {"answer": "Present", "rationale": "The word \"capable\" in the entry indicates the current feelings and attitudes of the testee, and \"with a difficult task\" and \"with parental support\" are also the words describing the current situation. So, the time dimension of the entry is now."}, "sentence_type": "Interrogative sentence", "pos": {"nouns": {"Difficulties": 1, "Support": 1}, "verbs": {"Faced": 1, "Yes": 1, "I feel": 1}, "adjectives": {"Item": 1}, "numerals": {"25": 1, "I": 1}, "pronouns": {"they": 1}}}, {"content": "At home, they are often used as 鈥渟acrifices鈥?or 鈥渄ams鈥?, "word_count": 16, "label": "", "sentence_structure": "Subject鈥搗erb鈥揷omplement", "subjective_objective": {"answer": "Objective", "rationale": "Because this problem is a description of an objective phenomenon, namely the role and treatment of patients in the home. This phenomenon is not determined by the patient鈥檚 choice, but by the family environment and other external factors."}, "sentence_sentiment": {"answer": "Negative", "rationale": "Because the expression \"reputed as a \"sacred faller\" or \"a \"damn horse\" in the entry is negative emotional and indicates that the role of the family is not recognized or misunderstood."}, "language_style": {"answer": "Tactful and warm", "rationale": ""}, "time_dimension": {"answer": "Present", "rationale": ""}, "sentence_type": "Negative declarative sentence", "pos": {"nouns": {"Home": 1, "A scapegoat": 1, "The bad guys": 1}, "verbs": {"They're often taken for granted": 1}, "adverbs": {"Often": 1}}}, {"content": "Parents always pick on my favorite friends.", "word_count": 13, "label": "", "sentence_structure": "Subject鈥搗erb鈥搊bject", "subjective_objective": {"answer": "Subjective", "rationale": "The entry for this questionnaire is a question of the views and feelings of the patient and is therefore subjective."}, "sentence_sentiment": {"answer": "Negative", "rationale": "Because the \"always picky\" behavior in the entries is negative."}, "language_style": {"answer": "Tactful and warm", "rationale": "The language style of the entry is more euphemistic and sweet, using the word \"always\" to express a continuing state of affairs without directly blaming or criticizing the other. At the same time, the word \"crasy\" is not too strong emotional, but rather expresses its feelings in a more objective way. Such expression is more acceptable and understandable and helps to create a good atmosphere of communication."}, "time_dimension": {"answer": "Present", "rationale": "\"Always picky\" indicates that such behaviour is recurrent and customary, and therefore the temporal dimension is now judged."}, "sentence_type": "Negative declarative sentence", "pos": {"nouns": {"parents": 1, "I": 1, "friends": 1}, "verbs": {"Always": 1, "Pick": 1, "Choose": 1, "Yeah": 1}, "adjectives": {"Picky": 1}, "numerals": {"27": 1}}}, {"content": "Parents always think I caused their trouble.", "word_count": 16, "label": "", "sentence_structure": "Subject鈥搗erb鈥揷omplement", "subjective_objective": {"answer": "Subjective", "rationale": "Because it involves the patient ' s understanding and feelings of his or her role in the family, it is the patient ' s subjective perception."}, "sentence_sentiment": {"answer": "Negative", "rationale": "Basis: The expression \"parents think their discomfort is caused by me\" in this entry implies that children may be misunderstood or blamed in family relationships, which may cause negative feelings of frustration, anxiety or helplessness to be perceived as negative."}, "language_style": {"answer": "Tactful and warm", "rationale": "This entry uses a more euphemistic formulation, which avoids direct accusations or criticism by asking whether the respondents feel that their parents have changed their moods as a result of their actions."}, "time_dimension": {"answer": "Present", "rationale": ""}, "sentence_type": "Negative declarative sentence", "pos": {"nouns": {"parents": 1, "Not fast": 1, "I": 1}, "verbs": {"I thought": 1, "Cause": 1}, "adverbs": {"By": 1}, "numerals": {"28": 1}}}, {"content": "Parents always try to encourage me to be the best.", "word_count": 15, "label": "", "sentence_structure": "Subject鈥搗erb鈥揷omplement", "subjective_objective": {"answer": "Subjective", "rationale": "Because \"to be the best\" is the subjective desire and expectation of parents."}, "sentence_sentiment": {"answer": "Positive", "rationale": "According to the description in the entry, \"Parents always try to encourage me\", this behaviour is positive and is intended to help children achieve self-worth and growth."}, "language_style": {"answer": "Tactful and warm", "rationale": "The words \"and try to encourage\" and \"and make me the man\" are used in this entry, which is more gentle and caring, giving people a sense of affection, so language style is euphemism."}, "time_dimension": {"answer": "Present", "rationale": "The word \"general\" means regular, customary behavior, \"tried to encourage me\" and \"make me the best\" are the actions of parents. So, this entry describes the situation."}, "sentence_type": "Affirmative declarative sentence", "pos": {"nouns": {"parents": 1, "Good man": 1}, "verbs": {"Total": 1, "Encourage": 1, "鈾?And I'm gonna be 鈾?: 1}, "adverbs": {"Always": 1}, "numerals": {"29": 1}}}, {"content": "Parents always tell me they love me.", "word_count": 13, "label": "", "sentence_structure": "Subject鈥揷opula鈥損redicative", "subjective_objective": {"answer": "Subjective", "rationale": "This is a subjective survey of how patients feel about their family environment."}, "sentence_sentiment": {"answer": "Positive", "rationale": "Because the behavior in the entry (parents expressing love for their children) is positive."}, "language_style": {"answer": "Tactful and warm", "rationale": "The entry is more adept and interactive when it is spoken in a gentle and inviting manner, rather than by direct statements or orders."}, "time_dimension": {"answer": "Past", "rationale": "The keyword \"parents always say to me \"to express the past's regular, customary behaviour, and \"to love me\" also points to the past. Thus, the entry relates to the past's behaviour or mentality."}, "sentence_type": "Affirmative declarative sentence", "pos": {"nouns": {"parents": 1, "Organisation": 1, "Love": 1}, "verbs": {"In": 1, "be": 2}, "adverbs": {"Total": 1}, "numerals": {"30": 1}, "pronouns": {"I": 1}}}, {"content": "31. Parents trusted me and allowed me to do something alone.", "word_count": 18, "label": "", "sentence_structure": "Subject鈥揷opula鈥損redicative", "subjective_objective": {"answer": "Subjective", "rationale": "Because \"very trusting and allowing me to do something alone\" is the patient's subjective feeling and understanding."}, "sentence_sentiment": {"answer": "Positive", "rationale": "This item is a positive emotional attitude, as it expresses the trust and encouragement of parents in their children."}, "language_style": {"answer": "Tactful and warm", "rationale": "The language style of the entry is more euphemistic. It uses a more moderate tone to ask the respondents about their relationship with their parents, rather than direct orders or statements of facts. This expression is more personal and interactive and facilitates a more realistic and effective answer."}, "time_dimension": {"answer": "Present", "rationale": "The verb \"is allowed\" is used in both the general current, meaning that the act has not only occurred in the past, but may still be ongoing. And the word \"very\" in \"trusted and\" is a strong emotional state or attitude, which usually refers to the current state or situation. So the entry to the questionnaire asks the person who was tested about his or her current feelings and circumstances, and the time dimension is judged to be the present."}, "sentence_type": "Affirmative declarative sentence", "pos": {"nouns": {"parents": 1, "I": 2, "Something": 1}, "verbs": {"to": 1, "very": 1, "Trust": 1, "and": 1, "Allow": 1, "Completed": 1, "Alone": 1}, "adjectives": {"Independent": 1}, "adverbs": {"Alone": 1}, "numerals": {"31": 1}, "pronouns": {"I": 2}}}, {"content": "I think my parents respect my point of view.", "word_count": 12, "label": "", "sentence_structure": "Subject鈥揷opula鈥損redicative", "subjective_objective": {"answer": "Subjective", "rationale": "As this entry addresses the patient ' s feelings and perception that the patient believes that the parents respect his views and are therefore subjective in nature."}, "sentence_sentiment": {"answer": "Positive", "rationale": "The purpose of this article is to express a positive attitude of the person under investigation that he or she considers to be respected."}, "language_style": {"answer": "Tactful and warm", "rationale": "The expression is more personal and interactive, in a gentle and inviting manner, rather than in direct statements or orders. At the same time, the tone of the article is positive, expressing a positive view of the family environment of the interviewee, which is conducive to the active and cooperativeness of the interviewee."}, "time_dimension": {"answer": "Present", "rationale": "The keywords \"felt\" and \"respect\" both indicate that this is the current feeling and perception of the subject, and does not address past or future situations. Thus, the temporal dimension of the article is now."}, "sentence_type": "Affirmative declarative sentence", "pos": {"nouns": {"Questionnaire Entry": 1, "Views": 1, "parents": 1}, "verbs": {"I think": 1, "Respect": 1, "Give": 1}, "adjectives": {"Down there": 1, "very": 1}, "numerals": {"32": 1}, "pronouns": {"I": 1}}}, {"content": "I think parents would love to be with me.", "word_count": 13, "label": "", "sentence_structure": "Subject鈥揷opula鈥損redicative", "subjective_objective": {"answer": "Subjective", "rationale": "Because \"think\" is a subjective feeling, it reflects the patient's perception and feelings of being with his parents. So the perspective of this questionnaire entry is based on the patient's subjective perspective."}, "sentence_sentiment": {"answer": "Positive", "rationale": "Because the entry \"I'd love to be with you\" expresses positive attitudes and emotions."}, "language_style": {"answer": "Tactful and warm", "rationale": "The entry leads the respondents to express their views on their parents by asking questions, and describes their attitude in the way that they are \"willing to be with me,\" showing a sense of concern and concern. This expression is more personal and interactive than a direct statement, and it feels more comfortable and understandable."}, "time_dimension": {"answer": "Present", "rationale": "So, in terms of time dimension, the entry is in its current state.\""}, "sentence_type": "Affirmative declarative sentence", "pos": {"nouns": {"I": 1, "parents": 1, "Together": 1}, "verbs": {"I think": 1, "Yes": 1, "in": 1, "Call": 1}, "adjectives": {"very": 1}, "numerals": {"33": 1}}}, {"content": "34. I think my parents are mean to me. They're mean to me.", "word_count": 13, "label": "", "sentence_structure": "Subject鈥揷opula鈥損redicative", "subjective_objective": {"answer": "Subjective", "rationale": "Because it is perceived as a description of the individual feelings of the patient, it is a subjective view and evaluation."}, "sentence_sentiment": {"answer": "Negative", "rationale": "Because the words \"smugness\" and \"smugness\" in the entries are derogatory emotional expressions of dissatisfaction and complaints about parents."}, "language_style": {"answer": "Calm and direct", "rationale": "The entry is asked directly about the attitude and views of the parents, and is not over-modified or emotionally retroverted."}, "time_dimension": {"answer": "Present", "rationale": "The entry \"Feel\" and \"Stingy\" describe the current feelings and attitudes of the testees, and \"Persons are small\" are the acts or events of the past, so the time dimension of the entry is now judged."}, "sentence_type": "Negative declarative sentence", "pos": {"nouns": {"parents": 1, "I": 1, "- You're a little bit of a jerk": 1, "Stingy": 1}, "verbs": {"I think": 1, "to": 1}, "adjectives": {"very": 2, "Stingy": 1, "- You're a little bit of a jerk": 1}, "numerals": {"34": 1}}}, {"content": "Parents always say to me, \"If you do this, I'll be sad.\"", "word_count": 22, "label": "", "sentence_structure": "Subject鈥搗erb鈥搊bject", "subjective_objective": {"answer": "Subjective", "rationale": "This is because patients feel and understand subjectively what parents say about themselves."}, "sentence_sentiment": {"answer": "Negative", "rationale": "Because the words and content of the \"parents always say to me \"if you do this I would be sad\" are of a blame and threatening nature, expressing negative emotional tendencies."}, "language_style": {"answer": "Calm and direct", "rationale": "The entry asks the other in a more straightforward manner whether the parents are often heard to say similar words, without excessive rhetorical or emotional rendering, and with a more balanced and objective tone."}, "time_dimension": {"answer": "Present", "rationale": "Now, \"Parents\" always say \"always\" to me, \"always\" means \"recurring, \"customary\" and \"said\" means that it is not only a practice in the past, but may still be a practice. So the entry is to ask the testee what is happening now, i.e. whether their parents often say similar things to them."}, "sentence_type": "Affirmative declarative sentence", "pos": {"nouns": {"parents": 1, "Word": 1}, "verbs": {"Always": 1, "Say it": 1, "Do it": 1, "Yes": 1, "Sad": 1}, "adjectives": {"Similar": 1}, "adverbs": {"Always": 1}, "numerals": {"35": 1}, "pronouns": {"I": 1}}}, {"content": "36. Parents have to tell me what I'm doing when I'm home.", "word_count": 23, "label": "", "sentence_structure": "Subject鈥搗erb鈥搊bject", "subjective_objective": {"answer": "Subjective", "rationale": "Because the \"parents' demands\" and \"must have to be explained to them\" describe the feelings and thoughts of patients in the face of this situation, they are examined from a subjective perspective."}, "sentence_sentiment": {"answer": "Neutral", "rationale": "Because the entry does not express any positive or negative feelings, it merely presents a fact."}, "language_style": {"answer": "Calm and direct", "rationale": "The entry is in a straightforward manner, which is more objective and insensitive to the interviewee's communication with his parents within the family."}, "time_dimension": {"answer": "Present", "rationale": "The keywords are \"go home,\" \"must explain to them\" and \"doing things,\" which indicate that this is a current behavior or state. The entry is therefore a question about the situation of the person being tested."}, "sentence_type": "Affirmative declarative sentence", "pos": {"nouns": {"parents": 2, "Things": 1}, "verbs": {"Request": 1, "Back": 1, "I have to": 1, "Okay": 1, "In": 1, "Annotations": 1, "Do it": 1}, "adverbs": {"I'm sure": 1, "Okay": 1}, "numerals": {"36": 1}, "classifiers": {"individual": 1}, "pronouns": {"I": 1}}}, {"content": "37. I think parents are trying to make my youth more meaningful and more diverse.", "word_count": 22, "label": "", "sentence_structure": "Subject鈥搗erb鈥揷omplement", "subjective_objective": {"answer": "Subjective", "rationale": "Because \"the feeling\" suggests that it is a subjective view or feeling, not an objective fact."}, "sentence_sentiment": {"answer": "Positive", "rationale": "Because the expression in the entry is positive, it expresses a recognition and gratitude to the parents."}, "language_style": {"answer": "Tactful and warm", "rationale": "The language style of the entry is more euphemistic and sweet, and it is comfortable and kind to express gratitude for the love and support of parents through the expression \"making my youth more meaningful and diverse\", and without any language of direct blame or criticism."}, "time_dimension": {"answer": "Past", "rationale": "The entry is for the testee ' s experience of the past, and the time dimension is the past."}, "sentence_type": "Affirmative declarative sentence", "pos": {"nouns": {"parents": 1, "I": 1, "Youth": 2, "Meaning": 1, "It's a lot of color": 1}, "verbs": {"I think": 1, "鈾?And I'm gonna be 鈾?: 1, "have": 1}, "adjectives": {"Try": 1, "More meaningful and diverse": 1}, "numerals": {"37": 1}}}, {"content": "Parents often say to me the same thing: \"Is this the reward we get for your work all day?\"", "word_count": 30, "label": "", "sentence_structure": "Subject鈥搗erb鈥搊bject", "subjective_objective": {"answer": "Subjective", "rationale": "This is because the patient ' s perception and perception of his or her parents ' behaviour is a subjective expression of his or her views."}, "sentence_sentiment": {"answer": "Negative", "rationale": "Because the words in the entry, \"This is the reward for your daily labor?\" are negative because they have a derogatory and blameful emotional tone."}, "language_style": {"answer": "Calm and direct", "rationale": "The entry is based on a direct statement that parents will speak similar language to their children. This expression is more objective and clear, and there is no excessive emotional rendering or rhetorical technique, so that the language style can be described as being calm and straightforward."}, "time_dimension": {"answer": "Present", "rationale": ""}, "sentence_type": "Interrogative sentence", "pos": {"nouns": {"parents": 1, "I": 2, "Word": 1}, "verbs": {"Regular": 1, "Presentation": 1, "Tell me": 1, "Okay": 1, "Got it": 1, "The reward for all the work": 1}, "adjectives": {"Similar": 1}, "adverbs": {"Just": 1}, "numerals": {"38": 1}, "pronouns": {"Here": 1, "we": 1}}}, {"content": "Parents often use the excuse not to be fond of me as a reason to not meet my demands.", "word_count": 19, "label": "", "sentence_structure": "Subject鈥搗erb鈥搊bject", "subjective_objective": {"answer": "Subjective", "rationale": "Because words like \"not me\" and \"not me\" are the patient's assessment of and feelings for the behaviour of his parents, and they are subjective."}, "sentence_sentiment": {"answer": "Negative", "rationale": "Because the entry \"I can't be used to justify not meeting my demands\" is negative."}, "language_style": {"answer": "Tactful and warm", "rationale": "The language style of the entry is more euphemistic. The term \"excus\" is used to suggest the contradictory mentality and possible misunderstanding of parents, while avoiding direct accusations or attacks. At the same time, more relaxed terms (such as \"meet my demands\") are used to express concern and understanding of individual needs."}, "time_dimension": {"answer": "Present", "rationale": "The entry indicates the current situation, where parents are not meeting my demands on the pretext that they are not being comfortable with me. Therefore, time dimensions are now judged."}, "sentence_type": "Negative declarative sentence", "pos": {"nouns": {"parents": 1, "- An excuse": 1, "Request": 1}, "verbs": {"Always": 1, "Satisfied": 1}, "adverbs": {"No, I can't": 1, "Cozy": 1}, "numerals": {"39": 1}, "pronouns": {"I": 2}}}, {"content": "I'm not feeling at ease if I don't do what my parents want.", "word_count": 24, "label": "", "sentence_structure": "Subject鈥搗erb鈥揷omplement", "subjective_objective": {"answer": "Subjective", "rationale": "Because \"frightful\" is a subjective emotional response, which depends on the patient's inner feelings and emotional experience, not on objective facts."}, "sentence_sentiment": {"answer": "Negative", "rationale": "Because the act of \"conscience disorder\" in the entry is negative."}, "language_style": {"answer": "Calm and direct", "rationale": "The article ' s purpose statement is more straightforward, is not based on too much qualifier or emotional overtones, is objective and firm. At the same time, it is not a matter of euphemism or inquiry, but rather of specifying what consequences should be expected of parents if they do not do so."}, "time_dimension": {"answer": "Present", "rationale": ""}, "sentence_type": "Negative declarative sentence", "pos": {"nouns": {"parents": 1, "Expectations": 1, "Conscience": 1}, "verbs": {"Do it": 2, "鈾?And I'm gonna be 鈾?: 1, "I feel": 1, "Unsettled": 1}, "numerals": {"40": 1}, "pronouns": {"I": 1}}}, {"content": "I think parents have a high demand for my academic achievement, physical activity or something like that.", "word_count": 28, "label": "", "sentence_structure": "Subject鈥揷opula鈥損redicative", "subjective_objective": {"answer": "Subjective", "rationale": "Because \"the feeling\" means that it's a personal view and feeling of the patient, a subjective examination."}, "sentence_sentiment": {"answer": "Negative", "rationale": "Because the \"high standard requirements\" in the entry may cause stress and anxiety to the child, there is a certain negative impact."}, "language_style": {"answer": "Calm and direct", "rationale": "The entry raises questions in a direct manner and is clear in language, with no excessive rhetorical or emotional colours. It is therefore judged to be \"cool and straight\"."}, "time_dimension": {"answer": "Present", "rationale": "Now the entry is a description of the current feelings and perceptions of the testee, and the words \"like\" and \"highly demanding\" indicate that this is a description of the current state."}, "sentence_type": "Affirmative declarative sentence", "pos": {"nouns": {"Achievements": 1, "Sports activities": 1, "Things": 1, "parents": 1}, "verbs": {"I think": 1, "have": 1, "Higher": 1, "Request": 1}, "adjectives": {"of": 4}, "numerals": {"41": 1}, "pronouns": {"I": 1}}}, {"content": "42. When I feel sad, I can get comfort from my parents.", "word_count": 20, "label": "", "sentence_structure": "Subject鈥搗erb鈥揷omplement", "subjective_objective": {"answer": "Subjective", "rationale": "Because feeling sad and being comforted is the subjective feeling of the patient."}, "sentence_sentiment": {"answer": "Positive", "rationale": "Because the purpose of the entry is to get comfort from parents."}, "language_style": {"answer": "Tactful and warm", "rationale": "The entry uses a relatively soft language style to express the concerns and concerns of the interviewees, which makes them feel admitted and understood. At the same time, the article is expressed more directly, without too many words or complex words, which are easily understood and answered."}, "time_dimension": {"answer": "Present", "rationale": "\"When sad\" means a state of emotion, and \"can be comforted\" also points to the current situation, so the entry should belong to the present."}, "sentence_type": "Affirmative declarative sentence", "pos": {"nouns": {"Time": 1, "parents": 1, "Consolation": 1}, "verbs": {"I feel": 1, "Got it": 1}, "adjectives": {"Sad": 1}, "numerals": {"42": 1}, "pronouns": {"I": 1}}}, {"content": "Parents punished me for no reason.", "word_count": 11, "label": "", "sentence_structure": "Subject鈥搗erb鈥揷omplement", "subjective_objective": {"answer": "Subjective", "rationale": "Because \"punishment\" is the patient's subjective memory and interpretation of the past."}, "sentence_sentiment": {"answer": "Negative", "rationale": "Because the \"punishment without cause\" in the entry is negative."}, "language_style": {"answer": "Calm and direct", "rationale": "The entry directly expresses the subject of questioning whether the other party has been punished without justification by his parents, without excessive rhetorical or emotional rendering, and with a more objective tone."}, "time_dimension": {"answer": "Past", "rationale": "The entry asks about the testee ' s past experience, i.e. whether the parents have ever punished him/her without any reason, and therefore the temporal dimension is the past."}, "sentence_type": "Negative declarative sentence", "pos": {"nouns": {"parents": 1, "Punishment": 1, "I": 1}, "verbs": {"Tsang": 1, "For no reason": 1, "Land": 1}, "adjectives": {"For no reason": 1}, "adverbs": {"For no reason": 1}, "numerals": {"43": 1}}}, {"content": "Parents have allowed me to do things that my friends do.", "word_count": 17, "label": "", "sentence_structure": "Subject鈥搗erb鈥搊bject", "subjective_objective": {"answer": "Subjective", "rationale": "Because \"permissible\" is a subjective attitude and decision that reflects the acceptance of the behaviour of the parents of the interviewee."}, "sentence_sentiment": {"answer": "Neutral", "rationale": "Because the entry does not have any emotional color."}, "language_style": {"answer": "Tactful and warm", "rationale": "This is easier to obtain the cooperation and trust of the interviewee and is conducive to the effectiveness of the investigation."}, "time_dimension": {"answer": "Present", "rationale": "The entry now asks whether the person being tested is allowed to do what their friends do by their parents ' current behaviour or circumstances, and therefore the time dimension is now judged."}, "sentence_type": "Affirmative declarative sentence", "pos": {"nouns": {"parents": 1, "Things": 2}, "verbs": {"Allow": 1, "Do it": 1}, "adverbs": {"Some": 1}, "numerals": {"44": 1}, "pronouns": {"I": 1, "they": 1}}}, {"content": "Parents often tell me they don't like my behavior at home.", "word_count": 19, "label": "", "sentence_structure": "Subject鈥搗erb鈥搊bject", "subjective_objective": {"answer": "Subjective", "rationale": "Because \"parents often say to me that they don't like my behaviour at home\" is an examination that expresses the views of the parents of the patient about their behaviour at home, based on the patient's subjective feelings and the evaluation of others."}, "sentence_sentiment": {"answer": "Negative", "rationale": "Because the expression \"not likes\" in the entry is negative."}, "language_style": {"answer": "Calm and direct", "rationale": "The entry asks the parents whether they are often dissatisfied with them in a direct presentation, the temperature of the language and the lack of excessive rhetorical or emotional rendering, and the expression is more objective and direct."}, "time_dimension": {"answer": "Past", "rationale": "According to the keyword and sentence structure, the words \"parents\" and \"often\" and \"speaks\" indicate that the act existed and is present, and can therefore be judged as \"old/present\"."}, "sentence_type": "Negative declarative sentence", "pos": {"nouns": {"parents": 1, "I": 1, "Home": 1, "Performance": 1}, "verbs": {"Say it": 2, "Yeah": 1, "in": 1, "Do it": 1}, "adverbs": {"Regular": 1}, "numerals": {"45": 1}}}, {"content": "46. Every time I eat, my parents persuade me or force me to eat more.", "word_count": 20, "label": "", "sentence_structure": "Subject鈥搗erb鈥搊bject", "subjective_objective": {"answer": "Objective", "rationale": "This is an objective fact of the patient's experience."}, "sentence_sentiment": {"answer": "Negative", "rationale": "Because the entry expresses a coercive and controlled attitude, which is negative in emotional terms."}, "language_style": {"answer": "Tactful and warm", "rationale": "The entry is more personal and interactive when it is presented in a soft, invitation-like tone, rather than in direct statements or orders. It also implies the interest and respect of the surveyer for the respondents."}, "time_dimension": {"answer": "Present", "rationale": "The entry describes the situation in which the person being tested is now regularly persuaded or forced by his parents to eat, and therefore falls within the time dimension of \"now\"."}, "sentence_type": "Affirmative declarative sentence", "pos": {"nouns": {"Rice": 1, "parents": 1}, "verbs": {"Eat": 1, "- I'm gonna get you out of here": 1, "Forced": 1, "Eat up": 2}, "adverbs": {"Every": 1, "Both": 1}, "numerals": {"46": 1, "Some": 1}, "classifiers": {"Some": 1}, "pronouns": {"I": 1}}}, {"content": "47. Parents often criticize me for being lazy and useless in front of others.", "word_count": 19, "label": "", "sentence_structure": "Subject鈥搗erb鈥搊bject", "subjective_objective": {"answer": "Objective", "rationale": "Because \"parents often criticize me for being lazy and useless\" is the patient's experience and observed objective facts, and it looks at the external effects of the patient, not the patient's subjective attitudes or feelings."}, "sentence_sentiment": {"answer": "Negative", "rationale": "Because the words \"criticism,\" \"slutness\" and \"no use\" in the entries are negative."}, "language_style": {"answer": "Calm and direct", "rationale": "The entry is more directly linguistically, not using excessive rhetorical or euphemisms, but rather asking the person under investigation about the specific facts. This straightforward approach may be offensive to the person under investigation, but it also allows more accurate access to the information required."}, "time_dimension": {"answer": "Past", "rationale": "The entry indicates that the respondent needs to recall his past in answering the question, and therefore the temporal dimension is the past.\""}, "sentence_type": "Negative declarative sentence", "pos": {"nouns": {"parents": 2, "others": 1, "Face": 1, "Criticism": 1, "I": 2, "Both": 1, "And": 1, "Lazy": 1, "It's useless": 1}, "verbs": {"Regular": 1, "In front of you": 1, "Noodles": 1, "Criticism": 2}, "adjectives": {"Lazy": 1, "It's useless": 1}, "adverbs": {"Regular": 1}, "numerals": {"47": 1}, "pronouns": {"I": 2}}}, {"content": "Parents are always concerned about what kind of friends I'm seeing.", "word_count": 15, "label": "", "sentence_structure": "Subject鈥搗erb鈥搊bject", "subjective_objective": {"answer": "Subjective", "rationale": "The degree and manner of concern of parents is based on their subjective views and values, and the perspective of the questionnaire is therefore subjective."}, "sentence_sentiment": {"answer": "Neutral", "rationale": "Because the entry does not have any emotional color."}, "language_style": {"answer": "Calm and direct", "rationale": "The entry is more straightforward in language by asking the interviewee whether he or she has found his or her parents often interested in friends who are in contact."}, "time_dimension": {"answer": "Present", "rationale": "Now, the entry \"often\" indicates that it is a regular act, while the verb \"concern\" adopts the general current, which means that the act has not only occurred in the past but may still be ongoing. Thus, the entry relates to the present act or state."}, "sentence_type": "Interrogative sentence", "pos": {"nouns": {"parents": 2, "friends": 1}, "verbs": {"Attention": 1, "- To be with someone": 1}, "adverbs": {"Often": 1}, "numerals": {"48": 1}}}, {"content": "49. If anything happens, I'm often the only one of my brothers and sisters who's ever been blamed.", "word_count": 25, "label": "", "sentence_structure": "Subject鈥損redicate", "subjective_objective": {"answer": "Subjective", "rationale": "Because \"it's often the only thing blamed among brothers and sisters\" is a person's subjective feeling and perception."}, "sentence_sentiment": {"answer": "Negative", "rationale": "Because the expression \"often the only thing blames the siblings\" in the entry has a negative emotional color."}, "language_style": {"answer": "Tactful and warm", "rationale": "The entry uses a more euphemistic expression that explores the role and situation of the other in the family by asking whether the other is often blamed by his siblings."}, "time_dimension": {"answer": "Past", "rationale": "\"If something happens\" suggests that this is a hypothetical situation of the past, and that it is often the only one of brothers and sisters who is blamed.\" The article describes the past or experience."}, "sentence_type": "Negative declarative sentence", "pos": {"nouns": {"Things": 1, "Blame": 1, "Brothers and sisters": 1}, "verbs": {"Come on": 1, "be": 1, "Yes": 1}, "adverbs": {"Often": 1}, "numerals": {"49": 1}, "pronouns": {"I": 1}}}, {"content": "50. Parents can make me grow like I want.", "word_count": 12, "label": "", "sentence_structure": "Subject鈥搗erb鈥揷omplement", "subjective_objective": {"answer": "Subjective", "rationale": "The subject is examined by the patient ' s opinion or perception of the way the parents treat themselves, which is a subjective assessment of the patient."}, "sentence_sentiment": {"answer": "Neutral", "rationale": "The entry is based on the fact that parents support the free development of the individual and do not express any positive or negative emotional aspects. It can therefore be considered neutral."}, "language_style": {"answer": "Tactful and warm", "rationale": "This is presented in a way that emphasizes the trust and support of parents in children and encourages their development at their own pace and interest."}, "time_dimension": {"answer": "Present", "rationale": "The key words \"can\" and \"let\" tell you what the state or situation is, so the answer is \"now\"."}, "sentence_type": "Interrogative sentence", "pos": {"nouns": {"parents": 1, "Development": 1}, "verbs": {"Jean": 1, "Yes": 1, "Let it be": 1}, "adjectives": {"Naturally": 1}, "adverbs": {"Land": 1}, "numerals": {"50": 1}, "classifiers": {"individual": 1}}}, {"content": "Parents often behaved rudely against me.", "word_count": 10, "label": "", "sentence_structure": "Subject鈥搗erb鈥揷omplement", "subjective_objective": {"answer": "Subjective", "rationale": "This is a subjective description of the patient ' s feelings and reflects the patient ' s perception and evaluation of his or her parent ' s behaviour."}, "sentence_sentiment": {"answer": "Negative", "rationale": "Because the act of \"coarseness\" is negative, it describes the inappropriate attitudes and behaviour of parents towards others."}, "language_style": {"answer": "Calm and direct", "rationale": "The language style of the entry is more calm and straightforward, and it questions the person surveyed about his or her parents鈥?behaviour in a factual way, without using excessive rhetorical techniques or emotional rendering."}, "time_dimension": {"answer": "Present", "rationale": ""}, "sentence_type": "Negative declarative sentence", "pos": {"nouns": {"parents": 1, "I": 1, "Nasty": 1, "Insolent": 1}, "verbs": {"to": 1, "Regular": 1, "Naughty": 1}, "numerals": {"51": 1}}}, {"content": "And sometimes even for a little bit of a little bit of a little bit of a pony, my parents would punish me very severely.", "word_count": 25, "label": "", "sentence_structure": "Subject鈥搗erb鈥搊bject", "subjective_objective": {"answer": "Subjective", "rationale": "Because \"draconian punishment\" and \"minority\" are described in terms of the patient's personal feelings and perceptions, they are subjective."}, "sentence_sentiment": {"answer": "Negative", "rationale": "Because the \"dramatic punishment\" in the entry is negative."}, "language_style": {"answer": "Calm and direct", "rationale": "The entry describes, in an objective manner, the severity of the parents ' handling of minor matters, the lack of excessive emotional rendering or reverence of language, and the relative calmness and straightforwardness of the language."}, "time_dimension": {"answer": "Past", "rationale": "The entry describes events or behaviour in the past, the keywords being \"sometimes\" and \"also\" and points to the past. And, \"will punish me severely\" means that this behaviour is repetitive and that it happened in the past."}, "sentence_type": "Negative declarative sentence", "pos": {"nouns": {"Time": 1, "Points": 1, "Porky, porcelain": 1, "It's nothing": 1, "parents": 1, "Yes": 2, "Hard": 1, "Punishment": 1, "I": 1}, "verbs": {"have": 1, "be": 1, "Even": 1, "Yes": 1, "- Hardly": 1, "Punishment": 1}, "adjectives": {"Small": 1, "Tight": 1}, "adverbs": {"Sometimes": 1, "Even": 1}, "numerals": {"52": 1}}}, {"content": "Parents beat me up for no reason.", "word_count": 11, "label": "", "sentence_structure": "Subject鈥搗erb鈥揷omplement", "subjective_objective": {"answer": "Objective", "rationale": "As beating is an objective act, it is a fact whether the parents have a reason or not."}, "sentence_sentiment": {"answer": "Negative", "rationale": "Because the \"battered without cause\" act in the entry is negative."}, "language_style": {"answer": "Calm and direct", "rationale": "The language style of the entry is relatively calm and straightforward, and the use of excessive rhetorical techniques or emotional renderings is not used to describe in an objective manner the domestic violence that the person under investigation may have experienced."}, "time_dimension": {"answer": "Past", "rationale": ""}, "sentence_type": "Negative declarative sentence", "pos": {"nouns": {"parents": 2, "For no reason": 1, "Fight": 1, "I": 1}, "verbs": {"Tsang": 1, "For no reason": 1, "Fight": 1}, "adjectives": {"For no reason": 1}, "adverbs": {"Land": 1}, "numerals": {"53": 1}, "pronouns": {"I": 1}}}, {"content": "54. Parents usually take part in my hobby.", "word_count": 15, "label": "", "sentence_structure": "Subject鈥搗erb鈥搊bject", "subjective_objective": {"answer": "Subjective", "rationale": "Because \"parents usually engage in my hobby\" is a personal feeling and perception that is viewed from the patient's subjective point of view."}, "sentence_sentiment": {"answer": "Positive", "rationale": "This entry is an indication of the support and care that parents give to their children and is positive emotional orientation."}, "language_style": {"answer": "Tactful and warm", "rationale": "The entry expresses a caring and supportive attitude, more euphemistic and friendly language, by asking the other side about parents鈥?involvement in their hobby. At the same time, this approach to the issue is more likely to resonate and respond positively."}, "time_dimension": {"answer": "Present", "rationale": "So the answer is \"now\"."}, "sentence_type": "Affirmative declarative sentence", "pos": {"nouns": {"parents": 1, "Amateur activities": 1}, "verbs": {"Yes": 1, "Participation": 1}, "numerals": {"54": 1}, "pronouns": {"I": 1}}}, {"content": "I've been beaten by my parents a lot.", "word_count": 7, "label": "", "sentence_structure": "Subject鈥搗erb鈥揷omplement", "subjective_objective": {"answer": "Subjective", "rationale": "Because \"hit by parents\" is a description of the patient's personal experience and feelings, and it's a subjective perspective."}, "sentence_sentiment": {"answer": "Negative", "rationale": "Because the \"strike-to-parent\" act in the entry is negative."}, "language_style": {"answer": "Tactful and warm", "rationale": "The phrase \"55. I am often beaten by my parents\" is more calm and straightforward, and it directly expresses the subject of asking the respondents whether they are often subjected to domestic violence, without excessive rhetorical or emotional overtook. While this expression appears to be objective and straightforward, it may have some negative psychological effects on the respondents."}, "time_dimension": {"answer": "Past", "rationale": "According to the sentence entered, \"I'm often beaten by my parents\" indicates that this behaviour is more frequent, and the \"hit\" action occurred in the past, so the entry is mainly about the testee's past experience."}, "sentence_type": "Negative declarative sentence", "pos": {"nouns": {"55": 1, "I": 1, "parents": 1, "Fight": 1}, "verbs": {"Regular": 1, "Scrap": 1, "Fight": 1}, "adverbs": {"Regular": 1}, "numerals": {"55": 1}, "classifiers": {"individual": 1}, "pronouns": {"I": 1}}}, {"content": "Parents often allow me to go where I like, and they don't worry too much.", "word_count": 25, "label": "", "sentence_structure": "Subject鈥搗erb鈥搊bject", "subjective_objective": {"answer": "Objective", "rationale": "Because \"parents often allow me to go where I like\" and \"they don't worry too much\" are the descriptions of the objective facts. The subjective idea of examining is subjective."}, "sentence_sentiment": {"answer": "Positive", "rationale": "Because \"I'm allowed to go where I like\" and \"not too worried\" are positive behaviors or attitudes."}, "language_style": {"answer": "Tactful and warm", "rationale": "The language style of the entry is more euphemism. It does not directly criticize or accuse parents of their actions, but rather expresses the author鈥檚 support and gratitude for them in a positive and positive way. It is also expressed in a way that is full of understanding and tolerance, giving people a comfortable and sweet feeling."}, "time_dimension": {"answer": "Present", "rationale": "\"Parents often allow me to go where I like,\" \"often\" indicates that it is a regular act and that it has happened over time. And \"and they don't worry too much\" also indicates that it is a state of affairs. So, the temporal dimension of this entry is now."}, "sentence_type": "Affirmative declarative sentence", "pos": {"nouns": {"parents": 1, "Local": 2, "I'm worried": 1}, "verbs": {"Often": 1, "Allow": 1, "Go on": 1, "That's too much": 1}, "adjectives": {"Yeah": 1}, "adverbs": {"And": 1}, "numerals": {"56": 1}, "pronouns": {"I": 1, "they": 1}}}, {"content": "57. Parents have strict restrictions on what I should do, what I should not do, and they don't give in.", "word_count": 26, "label": "", "sentence_structure": "Subject鈥搗erb鈥搊bject", "subjective_objective": {"answer": "Subjective", "rationale": "Because \"restricted and uncompromising\" is the patient's personal feeling and perception, and it is subjective."}, "sentence_sentiment": {"answer": "Negative", "rationale": "Because the words \"strictly restrained\" and \"insubordination\" in this entry indicate negative control and interference."}, "language_style": {"answer": "Calm and direct", "rationale": "Calm down straight."}, "time_dimension": {"answer": "Present", "rationale": ""}, "sentence_type": "Affirmative declarative sentence", "pos": {"nouns": {"parents": 2, "What": 3, "I shouldn't": 2, "Strict": 1, "Limits": 1, "Never": 1, "Compliment": 1}, "verbs": {"have": 1, "Do it": 3, "Jean": 1}, "adjectives": {"Strict": 1}, "adverbs": {"Both": 1, "Absolutely": 1}, "numerals": {"57": 1}, "pronouns": {"I": 1}}}, {"content": "Parents treat me in a way that makes me feel bad.", "word_count": 17, "label": "", "sentence_structure": "Subject鈥搗erb鈥揷omplement", "subjective_objective": {"answer": "Subjective", "rationale": "This is a perception that is generated by the patient himself and is observed from a subjective perspective."}, "sentence_sentiment": {"answer": "Negative", "rationale": "Because the term \"I'm embarrassed\" in the entry is negative emotional."}, "language_style": {"answer": "Calm and direct", "rationale": "In this entry, the words \"parents\" and \"normally\" and \"inconsulting me\" speak directly to the core of the problem, without too many words or emotionally strong words, and with a more straightforward tone. The language style of the article can therefore be described as \"cool and straight.\""}, "time_dimension": {"answer": "Past", "rationale": "The word \"normal\" in this entry denotes a regular, customary act, and \"in an awkward way\" implies that it has continued in the past, so the entry is a description of the past."}, "sentence_type": "Negative declarative sentence", "pos": {"nouns": {"parents": 1, "Modalities": 1}, "verbs": {"Treatment": 1, "鈾?And I'm gonna be 鈾?: 1}, "adjectives": {"I'm embarrassed": 1}, "numerals": {"58": 1}}}, {"content": "I think the fear of my parents about what might happen to me is exaggerated and excessive.", "word_count": 21, "label": "", "sentence_structure": "Subject鈥揷opula鈥損redicative", "subjective_objective": {"answer": "Subjective", "rationale": "Because \"fear\" and \"exaggerated, excessive\" are subjective perceptions of parents' attitudes."}, "sentence_sentiment": {"answer": "Negative", "rationale": "Because the words \"exaggered\" and \"excessive\" in the entries are negative jargon, implying that the author's attitude to parental concerns is negative."}, "language_style": {"answer": "Tactful and warm", "rationale": "The language style of the entry is more euphemistic, using the words \"possible,\" \"exaggerated,\" \"excessive\" to express one's views, without directly accusing or attacking the other. This expression is more polite, does not make people feel offended or harmed, and is more conducive to maintaining a good atmosphere of communication."}, "time_dimension": {"answer": "Present", "rationale": ""}, "sentence_type": "Negative declarative sentence", "pos": {"nouns": {"parents": 1, "I": 2, "I'm worried": 1, "Yes": 1}, "verbs": {"I think": 1, "Something's wrong": 1, "Maybe": 1, "be": 1, "Exaggeration": 1, "That's too much": 1}, "adjectives": {"Exaggerating": 1, "It's too much": 2}, "adverbs": {"Maybe": 1}, "numerals": {"59": 1}}}, {"content": "I feel a warm, sweet and intimate feeling between my parents and my parents.", "word_count": 21, "label": "", "sentence_structure": "Subject鈥揷opula鈥損redicative", "subjective_objective": {"answer": "Subjective", "rationale": "Because I think it's the patient's subjective feeling, the entry is subjectively viewed."}, "sentence_sentiment": {"answer": "Positive", "rationale": "Because the \"hot, sweet and intimate\" feeling in the entry is positive, and it expresses a positive emotional attitude."}, "language_style": {"answer": "Tactful and warm", "rationale": "The language style of the entry tends to be euphemistic, conveys positive feelings of the author about his/her parentage by using the words \"hot\", \"thanky\" and \"intimate.\" This expression is relatively subtle, does not express feelings too directly or strongly, and is more acceptable to the interviewees and willing to answer questions."}, "time_dimension": {"answer": "Present", "rationale": "The entry describes the current feelings and state of the subjects, i.e. how they feel about their current relationship with their parents, and thus judges \"now\"."}, "sentence_type": "Affirmative declarative sentence", "pos": {"nouns": {"parents": 1, "Warm": 1, "Kind": 1, "Feel": 1}, "verbs": {"Existence": 1, "I think": 1, "have": 1, "and": 1}, "adjectives": {"Intimacy": 1}, "numerals": {"60": 1}}}, {"content": "Parents tolerate that I have different opinions from them.", "word_count": 15, "label": "", "sentence_structure": "Subject鈥揷opula鈥損redicative", "subjective_objective": {"answer": "Subjective", "rationale": "Because \"Persons can tolerate my views differently from them\" is a patient's subjective feeling and perception. While this may also reflect objective factors such as family circumstances or parents' educational methods, in this case, the subject is more concerned with patients' subjective perceptions and experiences of their relationship with their parents."}, "sentence_sentiment": {"answer": "Neutral", "rationale": "Because the entry merely describes the fact that parents can tolerate different views from them, it does not show positive or negative emotional tendencies."}, "language_style": {"answer": "Tactful and warm", "rationale": "The entry uses a more euphemistic, invitation-like tone to ask the other side about his/her feelings, rather than direct orders or statements."}, "time_dimension": {"answer": "Present", "rationale": "The keywords are \"forlerable\" and \"different opinion\", which describe the current or recent situation and involve observations or experiences of parental behaviour. The temporal dimension of the article should therefore be the present."}, "sentence_type": "Affirmative declarative sentence", "pos": {"nouns": {"parents": 1, "Observations": 1}, "verbs": {"Tolerance": 1, "have": 1}, "numerals": {"61": 1}, "pronouns": {"I": 1, "they": 1}}}, {"content": "Parents often get mad at me without knowing why.", "word_count": 21, "label": "", "sentence_structure": "Subject鈥搗erb鈥揷omplement", "subjective_objective": {"answer": "Subjective", "rationale": "This is because the patients ' feelings and perceptions of their parents ' anger towards themselves are subjective."}, "sentence_sentiment": {"answer": "Negative", "rationale": "Because the \"breath\" in the entry is negative."}, "language_style": {"answer": "Tactful and warm", "rationale": "The entry is more euphemistic, soft in its language, not directly expressing negative emotions or accusations, but rather asking each other's feelings in a peaceful manner. At the same time, the entry uses the expression \"unknowable reasons\" to show understanding and concern for the feelings of the interviewee."}, "time_dimension": {"answer": "Past", "rationale": ""}, "sentence_type": "Negative declarative sentence", "pos": {"nouns": {"parents": 1, "Situation": 1, "- Grumpy": 1}, "verbs": {"Often": 1, "in": 1, "to": 1, "Let's go": 1, "Yes": 1, "Reason": 1}, "adjectives": {"Big hair": 1}, "adverbs": {"I don't know": 1}, "numerals": {"62": 1}, "pronouns": {"I": 1}}}, {"content": "When I did what I did, my street parents were proud of me.", "word_count": 21, "label": "", "sentence_structure": "Subject鈥搗erb鈥搊bject", "subjective_objective": {"answer": "Subjective", "rationale": "Because \"proportionate\" and \"goodly done\" are the subjective feelings of the patients."}, "sentence_sentiment": {"answer": "Positive", "rationale": "Because the \"Parents are very proud of me\" behavior and attitude in the entry are positive."}, "language_style": {"answer": "Tactful and warm", "rationale": "The entry is more personal and interactive when it is spoken in a gentle and inviting manner, rather than directly speaking or ordering. At the same time, the article is more compassionate in its content, asking whether the person surveyed was recognized and appreciated by his parents when he succeeded, reflecting interest and support for others, and a softer and warmer language."}, "time_dimension": {"answer": "Present", "rationale": "\"When I did something successful,\" and \"My street parents are very proud of me\" describe a normal situation or state, suggesting that this could be a frequent or ongoing event, and judging this entry as now."}, "sentence_type": "Affirmative declarative sentence", "pos": {"nouns": {"Yes": 1, "Success": 1, "Street": 1, "parents": 1, "Proud": 1}, "verbs": {"Do it": 1, "Access": 1, "Street": 1, "I feel": 1}, "numerals": {"63": 1}, "pronouns": {"I": 2}}}, {"content": "Parents often love me more than my brothers and sisters.", "word_count": 16, "label": "", "sentence_structure": "Subject鈥揷opula鈥損redicative", "subjective_objective": {"answer": "Subjective", "rationale": "Because \"preference\" is a subjective assessment or feeling."}, "sentence_sentiment": {"answer": "Negative", "rationale": "Because the word \"preference\" in the entries is negative."}, "language_style": {"answer": "Calm and direct", "rationale": "The tone of the article is a direct indication of the core of the problem, and is not retrofitted by excessive rhetorical or emotional colours. At the same time, the article is expressed in a more objective and neutral manner, avoiding any subjective assumptions or prejudices that may exist and facilitating more accurate and genuine feedback."}, "time_dimension": {"answer": "Present", "rationale": "The entry is a question about the testee's past, and the time dimension is a judgement of the past. The keyword \"in comparison to my siblings\" and \"often in love with me\" are all descriptions of the past, so it can be established that the entry is an inquiry about the past behavior and mental state."}, "sentence_type": "Negative declarative sentence", "pos": {"nouns": {"Brothers and sisters": 1, "parents": 2, "I": 2}, "verbs": {"Prejudice": 1}, "numerals": {"64": 1}, "pronouns": {"Often": 1}}}, {"content": "65. Sometimes even if I was wrong, parents blamed their siblings for it.", "word_count": 21, "label": "", "sentence_structure": "Subject鈥搗erb鈥搊bject", "subjective_objective": {"answer": "Subjective", "rationale": "Because \"privileging responsibility to siblings\" is a subjective judgement and behaviour of parents."}, "sentence_sentiment": {"answer": "Negative", "rationale": "Because the entry expresses a negative family environment and family relationship, the act of \"responsibility is attributed to siblings\" is negative."}, "language_style": {"answer": "Calm and direct", "rationale": "The entry raises the question in a straightforward language, with no excessive rhetorical or emotional colours and a more objective and calm tone."}, "time_dimension": {"answer": "Present", "rationale": ""}, "sentence_type": "Negative declarative sentence", "pos": {"nouns": {"Time": 1, "Error": 1, "I": 1, "parents": 2, "Responsibility": 1, "Blame": 1, "Brothers and sisters": 1}, "verbs": {"Sometimes": 1, "in": 1, "- Put it on": 1}, "adverbs": {"Yeah": 1}, "numerals": {"65": 1}, "pronouns": {"鈾?And the 鈾?: 1}}}, {"content": "Parents always hugged me.", "word_count": 7, "label": "", "sentence_structure": "Subject鈥搗erb鈥揷omplement", "subjective_objective": {"answer": "Subjective", "rationale": "Because hugging is a subjective move, parents are required to make a choice whether or not to proceed."}, "sentence_sentiment": {"answer": "Positive", "rationale": "Because hugging is an act of love and concern."}, "language_style": {"answer": "Tactful and warm", "rationale": "The entry is more adept and interactive when it is spoken in a gentle and inviting manner, rather than by direct statements or orders."}, "time_dimension": {"answer": "Present", "rationale": ""}, "sentence_type": "Affirmative declarative sentence", "pos": {"nouns": {"parents": 1, "Hug": 1}, "verbs": {"Regular": 1}, "numerals": {"66": 1}}}]}

{"questionnaire_title": "Auto Think Questionnaire", "questionnaire_category": "Mental health", "item_count": 30, "emotion_shift_overall": "From positive to negative", "language_style_overall": "Calm and direct", "items": [{"content": "I feel that living in the world is difficult.", "word_count": 11, "label": "", "sentence_structure": "Subject鈥揷opula鈥損redicative", "subjective_objective": {"answer": "Subjective", "rationale": "Because \"like\" means it's a subjective assessment or feeling."}, "sentence_sentiment": {"answer": "Negative", "rationale": "Because the entry \"difficult\" expresses a pessimistic, negative emotional attitude."}, "language_style": {"answer": "Calm and direct", "rationale": "This is a way to give the interviewer a more direct answer to the questions and to the formality of the questionnaire."}, "time_dimension": {"answer": "Present", "rationale": "The entry \"Feel\" uses the current state of affairs, indicating the current feelings and thoughts, and \"Live in the world\" describes the current situation of the person being tested, and can therefore be judged as now."}, "sentence_type": "Negative declarative sentence", "pos": {"nouns": {"鈾?In the world 鈾?: 1, "Difficulties": 1}, "verbs": {"I think": 1}, "adjectives": {"Thick": 1}}}, {"content": "I'm not good.", "word_count": 3, "label": "", "sentence_structure": "Subject鈥揷opula鈥損redicative", "subjective_objective": {"answer": "Subjective", "rationale": "Because \"I'm not good\" is what patients say about themselves, reflecting their subjective feelings and emotional state."}, "sentence_sentiment": {"answer": "Negative", "rationale": "Because \"bad\" in the entries is a negative emotional attitude."}, "language_style": {"answer": "Tactful and warm", "rationale": "The language style of the entry is more euphemistic and implicit, rather than directly accusing the other, but rather expressing his or her dissatisfaction or disagreement with one side in an indirect way. This expression is more resonant and thought-provoking, while avoiding conflicts and contradictions."}, "time_dimension": {"answer": "Present", "rationale": "And we can judge it from the time mark \"I\" in the sentence, which is a description of the situation, and \"bad\" is a subjective expression of the person being tested in his current state or emotional experience. So the time dimension of the article is now."}, "sentence_type": "Negative declarative sentence", "pos": {"verbs": {"I": 1, "Okay": 1}, "adjectives": {"not": 1}}}, {"content": "Why can't I always succeed?", "word_count": 9, "label": "", "sentence_structure": "Subject鈥搗erb鈥搊bject", "subjective_objective": {"answer": "Subjective", "rationale": "Because the problem involves the thinking, conviction and attitude of individuals, all of which are viewed from the patient鈥檚 subjective perspective. For example, patients may feel that they lack confidence or skills, or that they may feel that they have been treated unfairly, which is a subjective view of individuals."}, "sentence_sentiment": {"answer": "Negative", "rationale": "Because the question \"Why can't I ever succeed?\" in the entry is a bit negative."}, "language_style": {"answer": "Calm and direct", "rationale": "The entry directly asked why the subject of the investigation was always unsuccessful, without any qualifier or emotional term and with a more objective tone."}, "time_dimension": {"answer": "Past", "rationale": "The entry asks the testee about his or her success and therefore can be judged as \"now\" or \"the past\" from a time perspective."}, "sentence_type": "Interrogative sentence", "pos": {"nouns": {"Success": 1, "Reason": 1}, "verbs": {"Yes": 1, "Total": 1, "not": 1, "Success": 1}, "adverbs": {"Always": 1}, "numerals": {"3": 1}, "pronouns": {"I": 1}}}, {"content": "No one understands me.", "word_count": 6, "label": "", "sentence_structure": "Subject鈥搗erb鈥揷omplement", "subjective_objective": {"answer": "Subjective", "rationale": "Because understanding or not understanding is a subjective feeling."}, "sentence_sentiment": {"answer": "Negative", "rationale": "Because the expression \"no one understands me\" in the entry expresses a lonely, helpless feeling, which is negative."}, "language_style": {"answer": "Tactful and warm", "rationale": "The entry is more inclusive and cogent in a caring and understandable way by asking the interviewees whether there has been an ununderstanding situation, rather than directly accusing or questioning."}, "time_dimension": {"answer": "Past", "rationale": "The entry does not have clear time dimension information and therefore cannot be judged accurately as past, present or future."}, "sentence_type": "Negative declarative sentence", "pos": {"verbs": {"Nobody understands": 1}, "adjectives": {"I": 1}}}, {"content": "I am disappointed.", "word_count": 5, "label": "", "sentence_structure": "Subject鈥搗erb鈥揷omplement", "subjective_objective": {"answer": "Subjective", "rationale": "Because \"depression\" is an emotional experience, a patient's subjective feeling and evaluation."}, "sentence_sentiment": {"answer": "Negative", "rationale": "Because the \"depressing\" act in the entry is negative."}, "language_style": {"answer": "Tactful and warm", "rationale": "The entry is more expressions of care and support and helps to build good interpersonal relationships by engaging in dialogue in a way that is implicit and sensitive to the feelings of the other party, rather than directly criticizing or accusing."}, "time_dimension": {"answer": "Present", "rationale": "The entry indicates that the testee is now experiencing a negative sentiment, namely, that he is making others disappointed, an expression of the current state of affairs."}, "sentence_type": "Interrogative sentence", "pos": {"verbs": {"Disappointing": 1}}}, {"content": "6. I feel like I can't go on.", "word_count": 8, "label": "", "sentence_structure": "Subject鈥揷opula鈥損redicative", "subjective_objective": {"answer": "Subjective", "rationale": "Because it's the patient's subjective and emotional experience."}, "sentence_sentiment": {"answer": "Negative", "rationale": "Because the entry \"over\" expresses negative emotional attitudes."}, "language_style": {"answer": "Calm and direct", "rationale": "The entry is more straightforward and concise, expressing negative feelings about the state of life, with no excessive rhetoric or emotional rendering, and relatively calm and straightforward language styles."}, "time_dimension": {"answer": "Present", "rationale": "The entry expresses the current emotional state and feelings of the subject, and the word \"I think\" expresses subjective feelings and perceptions without addressing the temporal dimensions of the past or the future."}, "sentence_type": "Negative declarative sentence", "pos": {"verbs": {"I think": 1, "Pass": 1}, "adjectives": {"not": 1}, "adverbs": {"Down": 1, "Go on": 1}, "pronouns": {"I": 1}}}, {"content": "I wish I could be better.", "word_count": 8, "label": "", "sentence_structure": "Subject鈥搗erb鈥揷omplement", "subjective_objective": {"answer": "Subjective", "rationale": "Because \"hope better\" is a subjective attitude that expresses the patient's inner desires."}, "sentence_sentiment": {"answer": "Positive", "rationale": "Because the article is expressed as a desire to improve itself and has a positive emotional orientation."}, "language_style": {"answer": "Tactful and warm", "rationale": "The language style of the entry is more euphemistic, because it takes the form of a prayer sentence and expresses a positive expression of desire to improve their situation, giving a sense of encouragement and support. At the same time, the phrase \"better\" is more specific and practical, and more in line with the language habits of psychological questionnaires."}, "time_dimension": {"answer": "Future", "rationale": "The entry expresses the testee's expectations and vision for the future, and \"I wish I could be better\" indicates the testee's desire to look forward to and improve on the future's own situation, and therefore to be judged as the future."}, "sentence_type": "Exclamatory sentence", "pos": {"verbs": {"Hope": 1, "Yes": 1, "Okay": 1}, "adjectives": {"True": 1}, "pronouns": {"I": 1}}}, {"content": "I am weak.", "word_count": 4, "label": "", "sentence_structure": "Subject鈥揷opula鈥損redicative", "subjective_objective": {"answer": "Subjective", "rationale": "The basis for the judgement: \"I'm weak\" describes the patient's feelings and self-evaluation, and is therefore a subjective examination."}, "sentence_sentiment": {"answer": "Negative", "rationale": "Because \"weak\" is usually seen as a negative description."}, "language_style": {"answer": "Calm and direct", "rationale": "The entry directly expresses the subject of questioning the other party about its weakness, without excessive rhetorical or emotional rendering, and is more objective in tone."}, "time_dimension": {"answer": "Past", "rationale": "And in conclusion, the answer is \"now\"."}, "sentence_type": "Negative declarative sentence", "pos": {"verbs": {"very": 1, "Weakness": 1}, "adjectives": {"very": 1}}}, {"content": "9. My life does not go as I wish.", "word_count": 12, "label": "", "sentence_structure": "Subject鈥損redicate", "subjective_objective": {"answer": "Subjective", "rationale": "Because \"development as I wish\" reflects the patient's specific expectations and aspirations for life, which is a subjective feeling for the patient."}, "sentence_sentiment": {"answer": "Negative", "rationale": "Because the expression \"life does not go according to my wishes\" in the entry expresses a negative emotional attitude."}, "language_style": {"answer": "Tactful and warm", "rationale": "The entry is more adept and interactive when it is spoken in a gentle and inviting manner, rather than by direct statements or orders."}, "time_dimension": {"answer": "Present", "rationale": "The entry is about the subjective perception of the person being tested for the current life, and the temporal dimension is now judged."}, "sentence_type": "Exclamatory sentence", "pos": {"nouns": {"Life": 1, "Wish": 1}, "verbs": {"Development": 1}, "adjectives": {"Press": 1}, "adverbs": {"not": 1}}}, {"content": "I am not satisfied with myself.", "word_count": 8, "label": "", "sentence_structure": "Subject鈥搗erb鈥揷omplement", "subjective_objective": {"answer": "Subjective", "rationale": "Because \"very dissatisfied\" is an expression of subjective emotions and attitudes, it is an assessment of self-awareness by patients and is therefore subjective."}, "sentence_sentiment": {"answer": "Negative", "rationale": "Because \"unsatisfactory\" in the entry is negative."}, "language_style": {"answer": "Calm and direct", "rationale": "The entry uses simple, clear language to describe the subject, does not contain too many emotional or rhetorical techniques, and is more objective and calm. It asks the respondents in a direct question about their satisfaction, not about the purpose of trying to guide them to answer or conceal the question."}, "time_dimension": {"answer": "Past", "rationale": "The keyword \"self-dissatisfied\" shows the subjective feelings of the person being tested and also reflects his current psychological state."}, "sentence_type": "Negative declarative sentence", "pos": {"verbs": {"to": 1, "self": 1, "Satisfied": 1}, "adjectives": {"not": 1}, "numerals": {"10": 1}, "pronouns": {"I": 1}}}, {"content": "I feel like everything is going to be all right.", "word_count": 9, "label": "", "sentence_structure": "Subject鈥揷opula鈥損redicative", "subjective_objective": {"answer": "Subjective", "rationale": "Because the entry expresses the patient鈥檚 subjective feelings and perceptions that everything is bad. It is not an objective fact, but a judgment and attitude within the patient."}, "sentence_sentiment": {"answer": "Negative", "rationale": "Because the expression \"everything's bad\" is negative."}, "language_style": {"answer": "Calm and direct", "rationale": "The language style of the entry is calm, as it is a direct expression of the feelings of the interviewee, without too many rhetorical or emotional renderings. This expression is more direct, more concise and better able to convey the real thoughts and feelings of the interviewee."}, "time_dimension": {"answer": "Present", "rationale": "The entry uses subjective expression \"I feel\" and does not mention past or future time lines, so it can be judged as asking the present feelings of the subjects."}, "sentence_type": "Negative declarative sentence", "pos": {"nouns": {"Everything": 1}, "verbs": {"I think": 1, "It's not good": 1}, "adverbs": {"Both": 1}, "numerals": {"11": 1}}}, {"content": "I cannot continue.", "word_count": 7, "label": "", "sentence_structure": "Subject鈥搗erb鈥揷omplement", "subjective_objective": {"answer": "Subjective", "rationale": "Because \"can't keep going\" is a subjective feeling and an idea for a man."}, "sentence_sentiment": {"answer": "Negative", "rationale": "Because the \"can't keep\" act in the entry is negative."}, "language_style": {"answer": "Tactful and warm", "rationale": "The entry uses subjective, emotional terms of \"no\" and adds an exclamation mark to the end, giving people a strong sense of feeling and emotional resonance. This language style is more moderate, contributing to a good communication climate and a greater sense of participation by the respondents."}, "time_dimension": {"answer": "Present", "rationale": "The entry is about the feelings and experiences that are now, at this moment, that I feel I can't keep going."}, "sentence_type": "Negative declarative sentence", "pos": {"verbs": {"Hold on": 1, "Get down": 1}, "adverbs": {"Cannot": 1}}}, {"content": "I cannot start over.", "word_count": 7, "label": "", "sentence_structure": "Subject鈥搗erb鈥揷omplement", "subjective_objective": {"answer": "Subjective", "rationale": "Because \"renewal\" is a subjective idea or aspiration, not an objective thing or event."}, "sentence_sentiment": {"answer": "Negative", "rationale": "Because \"can't start again\" in the entry is an expression of a weak, negative emotion."}, "language_style": {"answer": "Calm and direct", "rationale": "The language style of the entry is relatively simple, with no excessive qualifier or emotional colour, and is more flat and objective, and more in line with the characteristics and requirements of the psychological questionnaire study."}, "time_dimension": {"answer": "Present", "rationale": "Based on the sentence 'can't start again', it can be judged that the entry is to ask the testee about the current state or feeling, and therefore the temporal dimension should be classified as now."}, "sentence_type": "Exclamatory sentence", "pos": {"verbs": {"Cannot": 1, "Start": 1}, "adjectives": {"Restart": 1}}}, {"content": "What the hell is wrong with me?", "word_count": 9, "label": "", "sentence_structure": "Subject鈥搗erb鈥揷omplement", "subjective_objective": {"answer": "Subjective", "rationale": "Because \"what's wrong with you\" is subjective, and it's subjective to assess it according to the person's own feelings and perceptions."}, "sentence_sentiment": {"answer": "Negative", "rationale": "Because the entry expresses a self-doubt and negative sentiment."}, "language_style": {"answer": "Calm and direct", "rationale": "The entry is more directly linguistically, with no excessive reticulation or euphemism, but rather directly asks the respondents whether they have certain undesirable habits or problems, which are neutral and concise."}, "time_dimension": {"answer": "Past", "rationale": "The entry asks the testee's own case, and the time dimension is the past."}, "sentence_type": "Interrogative sentence", "pos": {"nouns": {"Mistake": 1}, "verbs": {"Yes": 1, "What the hell": 1}}}, {"content": "I wish I was in another place.", "word_count": 12, "label": "", "sentence_structure": "Subject鈥搗erb鈥揷omplement", "subjective_objective": {"answer": "Subjective", "rationale": "Because hope is a feeling and expectation inside one person, and it is a subjective attitude."}, "sentence_sentiment": {"answer": "Negative", "rationale": "Because the entry expresses a feeling of dissatisfaction with the current situation and of a desire to change, it is a negative feeling."}, "language_style": {"answer": "Tactful and warm", "rationale": ""}, "time_dimension": {"answer": "Present", "rationale": "The key word for the future, \"I wish I was in another place\", expresses the expectations and aspirations of the subjects for the future, points to situations that have not yet occurred and that may arise in the future, and therefore judges the future."}, "sentence_type": "Exclamatory sentence", "pos": {"verbs": {"Hope": 1, "in": 1}, "adverbs": {"True": 1, "Other": 1, "One": 1}, "numerals": {"15": 1}, "pronouns": {"I": 1}}}, {"content": "I cannot deal with these matters simultaneously.", "word_count": 11, "label": "", "sentence_structure": "Subject鈥搗erb鈥揷omplement", "subjective_objective": {"answer": "Subjective", "rationale": "Because \"I can't deal with these things at the same time\" reflects a subjective feeling or perception of the patient. It is based on his personal experience, conviction and emotion and cannot be considered as objective facts."}, "sentence_sentiment": {"answer": "Negative", "rationale": "Because the expression \"can't deal with it simultaneously\" is negative."}, "language_style": {"answer": "Calm and direct", "rationale": "The language style of the entry is relatively calm and straightforward, and there is no excessive rhetorical or emotional rendering, but rather a simple and straightforward expression of the subject of asking if the other side is having difficulties in dealing with multiple tasks. This expression is more objective and straightforward."}, "time_dimension": {"answer": "Past", "rationale": "\"I can't deal with these things at the same time,\" \"I can't deal with them at the same time\" is a past experience and feeling, which means that I've been having trouble dealing with them at some point in the past."}, "sentence_type": "Interrogative sentence", "pos": {"verbs": {"Deal": 1, "These": 1}, "adjectives": {"Cannot": 1}, "pronouns": {"I": 1}}}, {"content": "I hate myself.", "word_count": 5, "label": "", "sentence_structure": "Subject鈥搗erb鈥揷omplement", "subjective_objective": {"answer": "Subjective", "rationale": "Because \"hate yourself\" is a subjective emotional experience."}, "sentence_sentiment": {"answer": "Negative", "rationale": "Because \"hate\" is a strong negative emotional vocabulary."}, "language_style": {"answer": "Calm and direct", "rationale": "The entry directly expresses the subject of a question about whether the other party is often concerned about past events, and does not use excessive rhetoric or emotional rendering, which is more objective and balanced."}, "time_dimension": {"answer": "Present", "rationale": "In the future, the entry contains visions and expectations for the future, with the keywords \"hate\" and \"self\" pointing to situations that have not yet occurred and that may arise in the future."}, "sentence_type": "Exclamatory sentence", "pos": {"verbs": {"Hate": 1, "I": 1}, "adjectives": {"self": 1}}}, {"content": "I'm worthless.", "word_count": 5, "label": "", "sentence_structure": "Subject鈥揷opula鈥損redicative", "subjective_objective": {"answer": "Subjective", "rationale": "Because \"no value\" is the patient's subjective feeling and evaluation."}, "sentence_sentiment": {"answer": "Negative", "rationale": "Because the expression \"no value\" in the entry is negative, and it represents denial and debasement of oneself."}, "language_style": {"answer": "Tactful and warm", "rationale": "The entry uses implicit and euphemistic expressions to ask the other side whether it is self-explanatory, does not directly accuse or question the other side of values, and is more respectful of the feelings and feelings of the interviewee. Such language styles tend to generate a greater resonance and trust among the interviewee and help to create a better communication climate and information gathering effect."}, "time_dimension": {"answer": "Present", "rationale": "The entry is an inquiry into the current feelings and mindset of the subject, and the keyword \"valueless\" indicates that it is a subjective emotional experience, so the temporal dimension is now judged."}, "sentence_type": "Negative declarative sentence", "pos": {"nouns": {"Value": 1}, "verbs": {"Nothing": 1, "be": 1}, "adjectives": {"Nothing": 1, "Value": 1}, "numerals": {"18": 1}, "pronouns": {"I": 1}}}, {"content": "I wish I had disappeared.", "word_count": 11, "label": "", "sentence_structure": "Subject鈥搗erb鈥揷omplement", "subjective_objective": {"answer": "Subjective", "rationale": "Because the words \"fear\" and \"disappear\" are the feelings and thoughts inside the patient, and are the subjective descriptions."}, "sentence_sentiment": {"answer": "Negative", "rationale": "Because the idea of \"disappear\" in the entry is negative."}, "language_style": {"answer": "Calm and direct", "rationale": "The entry expresses the respondent ' s desire for disappearance in a straightforward manner, without any qualification or disguise, and is more calm and direct in language style."}, "time_dimension": {"answer": "Future", "rationale": "The temporal dimension of the purpose of the article is therefore judged to be the future."}, "sentence_type": "Exclamatory sentence", "pos": {"nouns": {"Hope": 1, "I": 1, "Disappear": 1}, "verbs": {"Hope": 1, "Just": 1, "Disappear": 1}, "adverbs": {"True": 1, "One second": 1}}}, {"content": "What's wrong with me?", "word_count": 6, "label": "", "sentence_structure": "Subject鈥損redicate", "subjective_objective": {"answer": "Subjective", "rationale": "Because \"What's this?\" is a question expression that reflects the patient's confusion and doubt about his current state, which is the subjective feeling inside the patient."}, "sentence_sentiment": {"answer": "Negative", "rationale": "Because the tone of the article expresses confusion and unease, it has a certain negative emotional tone."}, "language_style": {"answer": "Tactful and warm", "rationale": ""}, "time_dimension": {"answer": "Present", "rationale": "The entry does not specify the order of time, but rather describes a current state or feeling, so we can judge it as being a question of the present."}, "sentence_type": "Interrogative sentence", "pos": {"nouns": {"I": 1}, "verbs": {"be": 1, "Yes": 1, "What": 1}, "adverbs": {"Here": 1}}}, {"content": "I am a loser.", "word_count": 6, "label": "", "sentence_structure": "Subject鈥揷opula鈥損redicative", "subjective_objective": {"answer": "Subjective", "rationale": "Because \"failers\" are subjective evaluations that reflect patients' perceptions and evaluations of themselves."}, "sentence_sentiment": {"answer": "Negative", "rationale": "Because the expression \"lost\" in the entry is negative."}, "language_style": {"answer": "Tactful and warm", "rationale": "The language style of the entry is euphemism. Although it expresses negative emotions and self-demeanor views, it is presented in a less nuanced way, avoiding too direct or aggressive a tone, and is more sensitive and understandable. This type of questionnaire is usually designed to encourage respondents to open their hearts, share their real feelings and experiences, and also to gain their trust and support."}, "time_dimension": {"answer": "Present", "rationale": "The entry is for self-awareness and evaluation of the testee, and the time dimension is now judged."}, "sentence_type": "Exclamatory sentence", "pos": {"nouns": {"Questionnaire": 1, "Entry": 1, "Loser": 1}, "verbs": {"be": 1}}}, {"content": "My life is a mess.", "word_count": 7, "label": "", "sentence_structure": "Subject鈥揷opula鈥損redicative", "subjective_objective": {"answer": "Subjective", "rationale": "Because \"a mess\" is a subjective feeling or evaluation that reflects patients' subjective perception of their own lives."}, "sentence_sentiment": {"answer": "Negative", "rationale": "Because the expression \"a mess\" has a negative emotional connotation, which means a bad state of affairs."}, "language_style": {"answer": "Calm and direct", "rationale": "The language style of the entry is more straightforward, not using euphemism or subtle expression, but simply asking if the other side feels that its life is a mess. It is easier to get the real thoughts and feelings of the answerers."}, "time_dimension": {"answer": "Past", "rationale": "The entry does not clearly indicate the point of time, but it can be inferred from the language expression that the testee describes and feels his or her current state and can therefore be judged as the past."}, "sentence_type": "Interrogative sentence", "pos": {"verbs": {"It's a mess": 1}, "adjectives": {"Mine": 1, "Shit": 1}}}, {"content": "I have done nothing.", "word_count": 5, "label": "", "sentence_structure": "Subject鈥搗erb鈥揷omplement", "subjective_objective": {"answer": "Subjective", "rationale": "This is a self-evaluation and subjective feeling of the patient."}, "sentence_sentiment": {"answer": "Negative", "rationale": "Because the expression \"does not work\" in the entry is negative."}, "language_style": {"answer": "Calm and direct", "rationale": ""}, "time_dimension": {"answer": "Past", "rationale": "The entry indicates the evaluation and perception of the testee, the temporal dimension of which does not clearly point to the past, present or future, and therefore the judgement is the past. At the same time, the \"nothing\" assessment is a summary and reflection of past experiences."}, "sentence_type": "Negative declarative sentence", "pos": {"verbs": {"Nothing": 1}, "adjectives": {"I": 1}}}, {"content": "I can't do it well.", "word_count": 6, "label": "", "sentence_structure": "Subject鈥損redicate", "subjective_objective": {"answer": "Subjective", "rationale": "Because \"no good work\" is the patient's subjective idea or belief."}, "sentence_sentiment": {"answer": "Negative", "rationale": "Because the expression \"can't do well\" in the entry is negative."}, "language_style": {"answer": "Calm and direct", "rationale": "The entry is of a blunt and straightforward language, with too many rhetorical or emotional qualifiers to express the negative view of the other side of its abilities in a concise and clear manner."}, "time_dimension": {"answer": "Future", "rationale": "The \"unlike\" in this entry expresses the negative attitude and perception of the testee about the future, namely, his doubts about future capabilities or achievements, or even his negative attitude. The subject therefore falls into the category of future issues."}, "sentence_type": "Exclamatory sentence", "pos": {"verbs": {"Good job": 1, "Maybe": 1}, "adjectives": {"Impossible": 1}}}, {"content": "25. I feel helpless.", "word_count": 7, "label": "", "sentence_structure": "Subject鈥揷opula鈥損redicative", "subjective_objective": {"answer": "Subjective", "rationale": "The feeling of isolation is a subjective or emotional experience."}, "sentence_sentiment": {"answer": "Negative", "rationale": "Because \"Isolation\" expresses a negative emotional attitude."}, "language_style": {"answer": "Tactful and warm", "rationale": "Warm and warm."}, "time_dimension": {"answer": "Present", "rationale": ""}, "sentence_type": "Negative declarative sentence", "pos": {"nouns": {"I": 1, "Isolation": 1, "No help": 1}, "verbs": {"I think": 1}}}, {"content": "26. There are things that must change.", "word_count": 8, "label": "", "sentence_structure": "Subject鈥揷opula鈥損redicative", "subjective_objective": {"answer": "Subjective", "rationale": "Because the word \"must\" shows the patient's subjective attitude and will."}, "sentence_sentiment": {"answer": "Neutral", "rationale": "Because the entry does not have any emotional color."}, "language_style": {"answer": "Calm and direct", "rationale": "The entry is simple and straightforward, directly expressing the view that something needs to be changed, without too many rhetorical or emotional colours, and with a more objective and calm tone."}, "time_dimension": {"answer": "Present", "rationale": ""}, "sentence_type": "Affirmative declarative sentence", "pos": {"nouns": {"Something": 1, "Change": 1}, "verbs": {"I have to": 1, "Change": 1}, "numerals": {"26": 1}}}, {"content": "I am sure there are problems.", "word_count": 6, "label": "", "sentence_structure": "Subject鈥揷opula鈥損redicative", "subjective_objective": {"answer": "Subjective", "rationale": "Because \"yes\" expresses patients' subjective attitude."}, "sentence_sentiment": {"answer": "Negative", "rationale": "Because \"there's definitely a problem\" in the entry is a self-denial and negative sentiment."}, "language_style": {"answer": "Calm and direct", "rationale": "The entry expresses the recognition and perception of its own problems in a straightforward manner, with a concise and straightforward language style, without too many rhetorical or emotional renderings."}, "time_dimension": {"answer": "Present", "rationale": "The entry uses the absolute word \"yes,\" implying that it is the person who is being tested for the moment or the idea, so it is judged as \"now\"."}, "sentence_type": "Affirmative declarative sentence", "pos": {"verbs": {"Sure": 1, "have": 1, "Problem": 1}, "adjectives": {"Sure": 1}, "numerals": {"27": 1}}}, {"content": "28. My future is hopeless.", "word_count": 8, "label": "", "sentence_structure": "Subject鈥揷opula鈥損redicative", "subjective_objective": {"answer": "Subjective", "rationale": "Because \"no hope\" is a subjective, negative perception or emotional experience, and a patient's subjective expectation of the future."}, "sentence_sentiment": {"answer": "Negative", "rationale": "The entry is an expression of negative sentiments and attitudes that are hopeless for the future."}, "language_style": {"answer": "Calm and direct", "rationale": ""}, "time_dimension": {"answer": "Future", "rationale": "The entry contains visions and expectations for the future, and the keyword \"my future\", which points to situations that have not yet occurred and that may arise in the future."}, "sentence_type": "Negative declarative sentence", "pos": {"nouns": {"The future": 1}, "verbs": {"have": 1, "Hope": 1}, "adjectives": {"Nothing": 1}}}, {"content": "29. This is of no value at all.", "word_count": 7, "label": "", "sentence_structure": "Subject鈥揷opula鈥損redicative", "subjective_objective": {"answer": "Subjective", "rationale": "Because this expression expresses the attitude and views of the patient, it is the patient ' s subjective feeling."}, "sentence_sentiment": {"answer": "Negative", "rationale": "Because the expression \"no value\" in the entry is negative."}, "language_style": {"answer": "Calm and direct", "rationale": "The entry describes in a negative and direct way the views of the subject on a certain matter, is not overly emotional or acrimony, and is more objectively ablaze in tone."}, "time_dimension": {"answer": "Present", "rationale": "The negative and subjective assessment expressed in the sentence, \"this is of no value at all\", makes it possible to judge that the issue tends to assess the views and attitudes of the interviewees on something or behaviour, and thus is of the time dimension."}, "sentence_type": "Negative declarative sentence", "pos": {"verbs": {"Here": 1, "Nothing": 1, "Value": 1}, "adjectives": {"Not at all": 1}}}, {"content": "I have no end in everything.", "word_count": 10, "label": "", "sentence_structure": "Subject鈥搗erb鈥搊bject", "subjective_objective": {"answer": "Subjective", "rationale": "This is a self-evaluation of the patient and expresses his/her own attitude and feelings."}, "sentence_sentiment": {"answer": "Negative", "rationale": "Because \"a single end\" means a lack of perseverance and persistence, and it's a negative description."}, "language_style": {"answer": "Calm and direct", "rationale": "The entry directly expresses the tendency to ask the other side whether it is easy to do things halfway, without excessive rhetorical or emotional rendering, and with a more objective tone."}, "time_dimension": {"answer": "Present", "rationale": "The words \"what to do\" in the sentence denotes an ongoing or recurrent act, and \"whoever has \" describes the characteristics or results of such conduct, does not relate to future or future plans or intentions and therefore determines the present conduct or state."}, "sentence_type": "Negative declarative sentence", "pos": {"verbs": {"Fuck": 1, "have": 1, "Head": 1, "None": 1, "End": 1}, "adjectives": {"What": 1, "Both": 1}, "adverbs": {"30": 1}}}]}

{"questionnaire_title": "Questionnaire on simple response modalities", "questionnaire_category": "Behavioral questionnaire", "item_count": 20, "emotion_shift_overall": "From positive to negative", "language_style_overall": "Tactful and warm", "items": [{"content": "1. Relief through work learning or some other activity", "word_count": 15, "label": "", "sentence_structure": "Subject鈥搗erb鈥揷omplement", "subjective_objective": {"answer": "Subjective", "rationale": "Because \"resolve\" is a subjective feeling and experience, different patients may have different understandings and reactions. The purpose of this questionnaire is to understand the patient鈥檚 subjective psychological state, not just the objective facts. So, \"release through work or some other activity\" should be considered a subjective entry."}, "sentence_sentiment": {"answer": "Positive", "rationale": "Because the purpose of the behaviour \"release through work learning or some other activity\" in the entry is positive and can alleviate negative emotions."}, "language_style": {"answer": "Tactful and warm", "rationale": "The entry is more adept and interactive when it is spoken in a gentle and inviting manner, rather than by direct statements or orders."}, "time_dimension": {"answer": "Present", "rationale": "According to the entry entered, \"1. By working or some other activity, we can judge it in two parts: the past: the entry does not contain any information about the past, and therefore it cannot be established that it is asking the testee about past behaviour or experience. Now: the entry does not have a clear time mark, but the use of the verbs \"by\" and \"deprive\" may suggest that this is an ongoing activity. At the same time, the expression \"work learning or other activity\" may indicate that the activity is a regular, customary act. In the future, since the entry does not contain any information about the future, it cannot be determined that it is a future plan or expectation for the interviewee. As stated above, based on the entry in the questionnaire, \"1 by working learning or some other activity\" is based on the \"now\"."}, "sentence_type": "Imperative sentence", "pos": {"nouns": {"Work studies": 1, "Other activities": 1, "Release": 1}, "verbs": {"Pass": 1, "Release": 1}, "adverbs": {"Some": 1}}}, {"content": "2. Talk to people, talk to people about their internal concerns", "word_count": 10, "label": "", "sentence_structure": "Subject鈥搗erb鈥揷omplement", "subjective_objective": {"answer": "Subjective", "rationale": "Because talking to people and speaking of their internal concerns are the patient ' s subjective will and behaviour."}, "sentence_sentiment": {"answer": "Positive", "rationale": "Because the purpose of the \"dissemination of inner distress\" in the entry is positive."}, "language_style": {"answer": "Tactful and warm", "rationale": "The entry is more friendly and interactive when it is presented in a gentle and inviting tone, rather than in direct statements or orders. At the same time, the article is more acclaimed, more accommodative and more open to the interviewee and is willing to participate in questions and answers."}, "time_dimension": {"answer": "Present", "rationale": "So the time dimension is now judged."}, "sentence_type": "Imperative sentence", "pos": {"nouns": {"Talk": 1, "person": 1, "Trouble": 1}, "verbs": {"and": 1, "Speech": 1, "Inside": 1}}}, {"content": "3. To see the good side of things as much as possible", "word_count": 10, "label": "", "sentence_structure": "Subject鈥搗erb鈥揷omplement", "subjective_objective": {"answer": "Subjective", "rationale": "Because seeing the good side of things is the patient's subjective opinion and attitude."}, "sentence_sentiment": {"answer": "Positive", "rationale": "Because the purpose of the entry is to see the good side of things."}, "language_style": {"answer": "Tactful and warm", "rationale": "The entry guides the interviewee in an encouraging language, allowing him to view things as positively as possible, rather than directly asking or ordering what the other party should do. This expression is more likely to resonate and cooperate with the interviewee, making the questionnaire more natural."}, "time_dimension": {"answer": "Present", "rationale": "The keywords \"as far as possible\", \"see\" and \"good side\" all indicate the behaviour or attitude that is being pursued, so it can be judged to be the behaviour or attitude of the person being questioned."}, "sentence_type": "Imperative sentence", "pos": {"nouns": {"Noodles": 1, "Things": 1, "Okay": 1}, "verbs": {"Yeah": 1, "Try": 1}, "adjectives": {"End of story": 1, "Maybe": 1, "Okay": 1}, "adverbs": {"Try": 1}, "classifiers": {"One": 2}}}, {"content": "4. Change your mind and rediscover what's important in life", "word_count": 18, "label": "", "sentence_structure": "Subject鈥搗erb鈥搊bject", "subjective_objective": {"answer": "Subjective", "rationale": "This sentence describes the process of self-reforming and reveals the importance of life, which reflects the patient ' s subjective attitudes and behaviour."}, "sentence_sentiment": {"answer": "Positive", "rationale": "Because \"change yourself\" and \"rediscover the importance of life\" in the entries are positive behaviors and attitudes."}, "language_style": {"answer": "Tactful and warm", "rationale": "The language style of the entry is more euphemistic and encouraging, allowing interviewees to feel comfortable in their thinking and encouraging them to explore the real importance of life proactively."}, "time_dimension": {"answer": "Present", "rationale": "The descriptions of \"change your mind\" and \"rediscover what matters in life\" are asking the testees about the current situation, so the time dimension of the article is now."}, "sentence_type": "Imperative sentence", "pos": {"nouns": {"Thoughts": 1, "Life": 1, "Important": 1}, "verbs": {"Change": 1, "Rediscover": 1}, "numerals": {"4锛?: 1}}}, {"content": "5. Not to take the problem too seriously.", "word_count": 9, "label": "", "sentence_structure": "Subject鈥搗erb鈥揷omplement", "subjective_objective": {"answer": "Subjective", "rationale": "This is a subjective examination of the patient ' s perception and attitude towards himself."}, "sentence_sentiment": {"answer": "Negative", "rationale": "Because the attitude in the entry that \"no problem is seen too seriously\" may lead to neglect of the existence of the problem, with potentially negative consequences."}, "language_style": {"answer": "Tactful and warm", "rationale": "The entry is more personal and interactive when it is presented in a gentle and inviting tone, rather than in direct statements or orders. At the same time, the article is more liberal in its tone, without a climate of too serious or tense for people to feel comfortable in communication."}, "time_dimension": {"answer": "Future", "rationale": "\"Do not take the problem too seriously\" is an idea and attitude to what may happen in the future and is judged to be the future."}, "sentence_type": "Imperative sentence", "pos": {"nouns": {"Questionnaire Entry": 1, "Problem": 2}, "verbs": {"Look": 1, "- Put it on": 1, "Too much": 1}, "adjectives": {"Serious": 1}, "adverbs": {"not": 1}, "numerals": {"5锛?: 1}}}, {"content": "6. To stand up for the struggle that you want to get.", "word_count": 16, "label": "", "sentence_structure": "Subject鈥搗erb鈥搊bject", "subjective_objective": {"answer": "Subjective", "rationale": "Because \"maintenance\" and \"the struggle for what you want\" are subjective attitudes and behaviours of patients."}, "sentence_sentiment": {"answer": "Positive", "rationale": "Because the words \"maintenance of your position\" and \"the struggle for what you want\" in this entry show positive attitudes and a tendency to act."}, "language_style": {"answer": "Calm and direct", "rationale": "The entry uses more direct and explicit language to express the themes of \"maintenance of your position\" and \"the struggle for one's own mind\" and does not use too much rhetorical or emotional overtook. This tone and presentation is more in keeping with the calm and direct language style."}, "time_dimension": {"answer": "Present", "rationale": "The entry describes some form of attitude or behaviour of the testee, namely, to stand up for his position and fight for what he wants. This attitude and behaviour is in the state of the present, and therefore is judged as \"now\"."}, "sentence_type": "Imperative sentence", "pos": {"nouns": {"self": 2, "Position": 1, "Fight": 1}, "verbs": {"Hold on": 1, "Okay": 1}, "adverbs": {"very": 1}, "pronouns": {"self": 1}}}, {"content": "7. Identification of several different ways to solve the problem", "word_count": 14, "label": "", "sentence_structure": "Subject鈥搗erb鈥搊bject", "subjective_objective": {"answer": "Subjective", "rationale": "Because finding is the patient's subjective move, and \"different ways to solve problems\" is the subjective goal that the patient wants to achieve."}, "sentence_sentiment": {"answer": "Positive", "rationale": "Because the purpose of the entries is to find a variety of ways to solve the problem, which is positive."}, "language_style": {"answer": "Tactful and warm", "rationale": "The entry is more adept and interactive when it is spoken in a gentle and inviting manner, rather than by direct statements or orders."}, "time_dimension": {"answer": "Present", "rationale": "The entry asks the respondent what strategy is used to solve the problem, and does not address the determination of the time dimension."}, "sentence_type": "Imperative sentence", "pos": {"nouns": {"Methodology": 2, "Problem": 1}, "verbs": {"Find": 1, "Out": 1, "Resolve": 1}, "numerals": {"How many": 1}, "classifiers": {"Plant": 1}}}, {"content": "8. Seek advice from relatives, friends or classmates", "word_count": 12, "label": "", "sentence_structure": "Subject鈥搗erb鈥搊bject", "subjective_objective": {"answer": "Objective", "rationale": "Because seeking advice is a specific act of behaviour, even though patients may have subjective ideas and motivations in seeking advice, the question itself is still being considered from an objective perspective. The answer is therefore \"objective\"."}, "sentence_sentiment": {"answer": "Positive", "rationale": "Because the purpose of the entry, \"to seek advice from relatives and friends or classmates\", is positive and helps to solve problems and gain new insights."}, "language_style": {"answer": "Tactful and warm", "rationale": ""}, "time_dimension": {"answer": "Present", "rationale": "The time word \"now\" in the sentence can be judged to be asking the testee about his current behaviour and circumstances, so the answer is \"now\"."}, "sentence_type": "Imperative sentence", "pos": {"nouns": {"Relatives": 1, "friends": 1, "Students": 1, "Recommendations": 1}, "verbs": {"Seek": 1, "Give it to me": 1}}}, {"content": "9. Some issues of changing practices or self-standing", "word_count": 17, "label": "", "sentence_structure": "Subject鈥搗erb鈥揷omplement", "subjective_objective": {"answer": "Subjective", "rationale": "Because \"change,\" \"some practices or some of their own problems\" are the will and decisions of the patient, and therefore the subjective perspective."}, "sentence_sentiment": {"answer": "Neutral", "rationale": "Because the entry is not emotional, it merely presents the fact that certain practices or problems of its own are changed."}, "language_style": {"answer": "Tactful and warm", "rationale": "The language style of the entry is relatively euphemistic, friendly, and without too much emphasis or command. It guides the respondents to think and answer questions in a way that makes them more comfortable and receptive, and thus more willing to cooperate in the study. At the same time, the language of the entry is made more accessible and understandable by using words that are not directly expressed (such as \"changes\", \"some of the original practices or some of their own problems\"."}, "time_dimension": {"answer": "Present", "rationale": "Key words such as \"some\" in the entry, \"original practice\" and \"his own problem\" indicate that the testee needs to change in the current situation, so the temporal dimension is now judged."}, "sentence_type": "Imperative sentence", "pos": {"nouns": {"Questionnaire Entry": 1, "Some": 2, "Approach": 1, "self": 1, "Problem": 1}, "verbs": {"Change": 1}, "numerals": {"9锛?: 1}, "classifiers": {"individual": 1}, "pronouns": {"Some": 2}}}, {"content": "10. Approaches to drawing on others to address similar difficult situations", "word_count": 15, "label": "", "sentence_structure": "Subject鈥搗erb鈥搊bject", "subjective_objective": {"answer": "Objective", "rationale": "The question is whether the patient has a habit of taking into account the experience or advice of others in the face of difficulties. This is the patient鈥檚 own personal behaviour and attitude, which is subjective. But the question is whether the person is asking for specific behaviour, so the answer depends more on whether the patient actually refers to the other person鈥檚 methods, i.e. objective."}, "sentence_sentiment": {"answer": "Neutral", "rationale": "Because the entry does not have any emotional color."}, "language_style": {"answer": "Tactful and warm", "rationale": "The language style of the entry is relatively warm, and the interviewer is invited to share his experience and views, rather than direct orders or statements. Positive terms such as \"learning\" and \"how\" are used to help the interviewee to answer questions with an open and positive mind."}, "time_dimension": {"answer": "Future", "rationale": ""}, "sentence_type": "Imperative sentence", "pos": {"nouns": {"Approach": 1, "Scenario": 1}, "verbs": {"Learning": 1, "Processing": 1, "I met him": 1, "Run away": 1}, "adjectives": {"Similar": 1, "Difficulties": 1}, "numerals": {"10": 1, "Others": 1}, "classifiers": {"individual": 1}, "pronouns": {"you": 1}}}, {"content": "11. Seeking amateurism and actively participating in cultural activities", "word_count": 14, "label": "", "sentence_structure": "Subject鈥損redicate", "subjective_objective": {"answer": "Subjective", "rationale": "Since \"seeking amateur hobbies\" and \"active participation in cultural activities\" are both individual behavioural choices and attitudes of patients, they are examined in terms of their willingness and motivation to participate actively and are therefore subjective in nature."}, "sentence_sentiment": {"answer": "Positive", "rationale": "Because the entries \"Finding for amateurs\" and \"Enjoying in the arts\" express positive emotional attitudes."}, "language_style": {"answer": "Tactful and warm", "rationale": "This is more acclaimable and helps to create a good atmosphere of communication."}, "time_dimension": {"answer": "Present", "rationale": "The entry describes the interests of the person tested and the behaviour of participating in the physical activity, and the temporal dimension does not clearly point to the past, present or future. The temporal dimension of the article can therefore be judged as general or neutral."}, "sentence_type": "Imperative sentence", "pos": {"nouns": {"Amateurity": 1, "Physical activities": 1}, "verbs": {"Seek": 1, "Participation": 1}, "adverbs": {"Positive": 1}, "numerals": {"11": 1}}}, {"content": "12. To exercise maximum restraint in their disappointment, remorse, grief and anger.", "word_count": 16, "label": "", "sentence_structure": "Subject鈥搗erb鈥揷omplement", "subjective_objective": {"answer": "Subjective", "rationale": "Because \"as restrained as much as possible\", \"depression, regret, sorrow and anger\" are the feelings and emotions inside the patient, which are his subjective attitudes and thoughts."}, "sentence_sentiment": {"answer": "Negative", "rationale": "Because the words \"disappointment, remorse, grief and anger\" in the entry are negative, implying that the interviewees need to contain them as much as possible, and are therefore negative."}, "language_style": {"answer": "Calm and direct", "rationale": "The language style of the entry is more calm and objective, and there is no excessive emotional rendering or rhetorical technique, but simply a requirement is made clear.\""}, "time_dimension": {"answer": "Present", "rationale": ""}, "sentence_type": "Imperative sentence", "pos": {"nouns": {"self": 1, "Disappointment": 1, "Regret": 1, "Sad": 1, "Anger": 1}, "verbs": {"Restraint": 1, "be": 1}, "adjectives": {"Try": 1}, "numerals": {"12锛?: 1}}}, {"content": "13. Attempt to take a break or leave, leaving the problem (fears) aside for a while", "word_count": 16, "label": "", "sentence_structure": "Subject鈥搗erb鈥搊bject", "subjective_objective": {"answer": "Subjective", "rationale": "It is the patient ' s subjective will and attitude to try to rest or take leave in order to ease his emotions and distress temporarily."}, "sentence_sentiment": {"answer": "Positive", "rationale": "Because the act of \"attempting to rest or take leave\" in the entry is positive and helps people to ease stress and anxiety."}, "language_style": {"answer": "Tactful and warm", "rationale": "The language style of the entry is more euphemistic. It uses the words \"attempted,\" \"for the moment to leave,\" rather than direct orders or strong demands that the interviewee relax and rest. This expression is more accommodative, more moderate and more acceptable to the interviewee. At the same time, the expression \"problem\" is more closely related to the interviewee's mentality, making it easier to resonate and to be willing to participate in the investigation."}, "time_dimension": {"answer": "Present", "rationale": "The keywords \"attempted rest or leave\" and \"for the time being, leave the problem behind\" describe the current behaviour or state. Thus, the temporal dimension of the article is now."}, "sentence_type": "Imperative sentence", "pos": {"nouns": {"Problem": 1, "Trouble": 1}, "verbs": {"Try": 1, "Leave": 1, "Throw it away": 1}, "adverbs": {"For now": 1}, "numerals": {"13": 1}}}, {"content": "14. To ease the trouble by smoking, drinking alcohol, taking drugs and eating.", "word_count": 17, "label": "", "sentence_structure": "Subject鈥搗erb鈥搊bject", "subjective_objective": {"answer": "Subjective", "rationale": "Because the way in which the person concerned is to be treated is the patient ' s subjective choice and behaviour."}, "sentence_sentiment": {"answer": "Negative", "rationale": "Because the purpose of the entry is to \"resolve troubles through smoking, drinking, taking drugs and eating\"."}, "language_style": {"answer": "Calm and direct", "rationale": "The language style of the article ' s purpose can therefore be judged as being straightforward and straightforward."}, "time_dimension": {"answer": "Present", "rationale": "The words \"by\" and \"by\" in the entry indicate that this is a present act, describing the current habits or preferences of the subject."}, "sentence_type": "Imperative sentence", "pos": {"nouns": {"Smoke": 1, "Wine": 1, "Medicine": 1, "Food": 1, "Trouble": 1}, "verbs": {"Pass": 1, "Smoking": 1, "Drink": 1, "Drug use": 1, "Eat": 1, "Undo": 1}, "numerals": {"14": 1}}}, {"content": "15. Considering that time will change the status quo, the only thing to do is wait", "word_count": 18, "label": "", "sentence_structure": "Subject鈥搗erb鈥搊bject", "subjective_objective": {"answer": "Objective", "rationale": "Because it is an objective fact that changes in time and waiting for them to happen, it is the patient ' s subjective attitude to believe that these events will or will not happen."}, "sentence_sentiment": {"answer": "Negative", "rationale": "Because the \"waiting\" attitude in the entry is negative, emphasizing passive acceptance and waiting."}, "language_style": {"answer": "Tactful and warm", "rationale": "The entry presents the point in a moderate and encouraging way, emphasizing that time can change the status quo and implying that such a change can be achieved only by waiting. Such language styles are more acceptable and understandable, and are also more friendly."}, "time_dimension": {"answer": "Present", "rationale": ""}, "sentence_type": "Imperative sentence", "pos": {"nouns": {"Time": 1, "Status": 1, "Wait": 1}, "verbs": {"I think": 1, "Change": 1, "Do it": 1}, "adverbs": {"One": 1}, "numerals": {"15": 1}, "pronouns": {"鈾?And the 鈾?: 1}}}, {"content": "16. Trying to forget the whole thing.", "word_count": 8, "label": "", "sentence_structure": "Subject鈥搗erb鈥揷omplement", "subjective_objective": {"answer": "Subjective", "rationale": "The attempt is to forget the subjective feelings and behaviour of the mental state of the person."}, "sentence_sentiment": {"answer": "Negative", "rationale": "Because the act of \"alleging to forget the whole thing\" expresses an attitude of evasiveness and avoiding problems, and it is negative."}, "language_style": {"answer": "Tactful and warm", "rationale": "The entry is more euphemistic and sweet, uses a gentle approach to questioning and expresses concern and understanding of the feelings of the person under investigation, giving a sense of comfort and affection."}, "time_dimension": {"answer": "Present", "rationale": ""}, "sentence_type": "Imperative sentence", "pos": {"nouns": {"Things": 1}, "verbs": {"Try": 1, "Forget it": 1, "The whole thing": 1}, "numerals": {"16": 1}}}, {"content": "17. Relying on others to solve problems", "word_count": 8, "label": "", "sentence_structure": "Subject鈥搗erb鈥揷omplement", "subjective_objective": {"answer": "Subjective", "rationale": "Because it is a matter of relying on others to solve problems requires patients to have a subjective sense of seeking help from others."}, "sentence_sentiment": {"answer": "Negative", "rationale": "Because the action of relying on others to solve problems in the entries is negative."}, "language_style": {"answer": "Tactful and warm", "rationale": "The entry is more adept and interactive when it is spoken in a gentle and inviting manner, rather than by direct statements or orders."}, "time_dimension": {"answer": "Present", "rationale": "The \"Leave to another person to solve the problem\" in this entry is the behaviour or tendency of the person being tested now or often, and thus the judgement is now."}, "sentence_type": "Imperative sentence", "pos": {"nouns": {"Problem": 1, "others": 1, "Dependence": 1}, "verbs": {"Break": 1, "Decision": 1, "Shit": 1}, "numerals": {"17": 1}}}, {"content": "18. Accept reality because there is no other way", "word_count": 12, "label": "", "sentence_structure": "Subject鈥損redicate", "subjective_objective": {"answer": "Objective", "rationale": "For accepting reality is an objective description and understanding of the current situation, without any subjective emotional colours."}, "sentence_sentiment": {"answer": "Negative", "rationale": "Because the act of \"accepting reality\" in the entry is negative."}, "language_style": {"answer": "Calm and direct", "rationale": "The language style of the entry is more calm and straightforward, making it clear that acceptance of reality is the only option, and that it is not used too much qualifier or rhetoric to make emotional. It is therefore judged to be \"cool and direct.\""}, "time_dimension": {"answer": "Present", "rationale": "The keyword \"accept reality\" and \"no other way\" represent the current attitude and perception of the testee, so the entry is to ask the testee about the present behaviour or mentality."}, "sentence_type": "Imperative sentence", "pos": {"nouns": {"Reality": 1, "Approach": 1}, "verbs": {"Accept": 1, "have": 1}, "adverbs": {"Because": 1, "Other": 1}, "pronouns": {"No, I'm not": 1}}}, {"content": "19. Imagination could have a miracle to change the situation.", "word_count": 15, "label": "", "sentence_structure": "Subject鈥損redicate", "subjective_objective": {"answer": "Subjective", "rationale": "Because fantasy is patients' subjective attitude."}, "sentence_sentiment": {"answer": "Neutral", "rationale": "Since the entry is not of a clear emotional orientation, it only describes a possible situation."}, "language_style": {"answer": "Tactful and warm", "rationale": "The entry uses the term \"fanta\" to give a mild, mysterious feeling, not directly indicating whether there is a belief that a miracle will change the situation, but rather to guide the interviewee to express his thoughts with an open mind and be more friendly."}, "time_dimension": {"answer": "Future", "rationale": "The entry contains visions and expectations for the future, and the key words are \"the illusion may have some miracle\" and \"change the status quo\" , all of which point to situations that have not yet occurred and that may occur in the future. The temporal dimension of the questionnaire is therefore judged to be the future."}, "sentence_type": "Imperative sentence", "pos": {"nouns": {"Fantasy": 1, "Miracles": 1, "Status": 1}, "verbs": {"Come on": 1, "Change": 1}, "numerals": {"Some": 1, "Plant": 1}}}, {"content": "20. Consolate yourself.", "word_count": 6, "label": "", "sentence_structure": "Subject鈥搗erb鈥揷omplement", "subjective_objective": {"answer": "Subjective", "rationale": "Because it is a subjective idea or act that comforts itself."}, "sentence_sentiment": {"answer": "Positive", "rationale": "Because the purpose of the action of \"self-consoling\" in the entry is positive."}, "language_style": {"answer": "Tactful and warm", "rationale": "The language style of the entry is more moderate, more thoughtful, and has some orientation and motivation, trying to arouse the emotional resonance and self-reflection of the person under investigation, thereby gaining a deeper understanding of his or her psychological state. So I think the language style of the entry is a \"fluent\" type."}, "time_dimension": {"answer": "Present", "rationale": "The entry contains a keyword that indicates the current or ongoing actions, \"self-consolation\". This indicates that the testees are describing their behaviour or feelings at this moment, and therefore are part of the current time dimension."}, "sentence_type": "Imperative sentence", "pos": {"nouns": {"Questionnaire Entry": 1, "Consolation": 1, "self": 2}, "verbs": {"I'll make myself comfortable": 1}, "numerals": {"20": 1}}}]}

{"questionnaire_title": "Double-stage emotional disorder self-assessment", "questionnaire_category": "Mental health", "item_count": 12, "emotion_shift_overall": "From positive to negative", "language_style_overall": "Tactful and warm", "items": [{"content": "I say a lot more than usual, or I speak much faster than usual.", "word_count": 24, "label": "", "sentence_structure": "Subject鈥搗erb鈥揷omplement", "subjective_objective": {"answer": "Subjective", "rationale": "Because the entry describes a subjective feeling or experience, that is, \"sometimes\" feels more or more rapid than usual. This requires self-observation and assessment by the patient itself, and therefore is subjective."}, "sentence_sentiment": {"answer": "Positive", "rationale": "Because \"speech\" and \"speech\" in the entries can be considered an expression of excitement or vitality."}, "language_style": {"answer": "Tactful and warm", "rationale": "The language style of the entry is euphemismal and gentle, using a number of emotional terms, such as \"sometimes\", \"more\" and \"more\" than usual, which give people a sense of affection and understanding. At the same time, the entry does not directly state or order the other side to change its own way of speaking, but rather raises questions in an invitation-style tone that enables the other side to respond more naturally and feel respected and understood. The language style of the article can therefore be described as euphemism."}, "time_dimension": {"answer": "Present", "rationale": ""}, "sentence_type": "Affirmative declarative sentence", "pos": {"nouns": {"Words": 2, "Speed": 2, "Normal": 2}, "verbs": {"More": 3, "Say it": 2}, "adverbs": {"Sometimes": 1, "- Yeah": 4, "It's much faster": 1, "More than that": 1, "Normal": 2}, "numerals": {"1": 1}, "pronouns": {"I": 1}}}, {"content": "I sometimes do much more than usual, or do more than usual.", "word_count": 25, "label": "", "sentence_structure": "Subject鈥搗erb鈥揷omplement", "subjective_objective": {"answer": "Subjective", "rationale": "Because the questionnaire entries describe a person's subjective feelings and behaviour, such as \"sometimes more than usual\" and \"doing more than usual\" are subjective experiences and behavioural choices of patients. Thus, the view of the questionnaire entries is subjective."}, "sentence_sentiment": {"answer": "Positive", "rationale": "Because \"more proactive\" and \"more than usual\" in the entries are positive behavioral."}, "language_style": {"answer": "Tactful and warm", "rationale": ""}, "time_dimension": {"answer": "Present", "rationale": "The entry describes the behaviour of the person tested at a given time (i.e. \"sometimes\"), and the conduct may occur at any time, and thus the temporal dimension is now judged."}, "sentence_type": "Imperative sentence", "pos": {"nouns": {"Questionnaire": 1, "Entry": 1}, "verbs": {"Analysis": 1, "be": 1, "- Yeah": 1, "Much more proactive": 1, "Do it": 1}, "adjectives": {"Normal": 2, "More": 1}, "adverbs": {"Sometimes": 1, "very": 1}, "numerals": {"2": 1}, "pronouns": {"I": 1}}}, {"content": "I'll be in a mood of excitement and anger.", "word_count": 11, "label": "", "sentence_structure": "Subject鈥揷opula鈥損redicative", "subjective_objective": {"answer": "Subjective", "rationale": "Because \"excited mood\" is a description of a subjective feeling or emotional state, and it is the individual experience of the patient. Although it may be caused by objective factors (e.g. stress, anxiety, etc.), it is itself a subjective experience. So, \"I will get into a mood of irritation\" is a subjective entry."}, "sentence_sentiment": {"answer": "Negative", "rationale": "Because the emotional state of \"excited and angry\" in the entry is negative."}, "language_style": {"answer": "Tactful and warm", "rationale": "Warm and warm."}, "time_dimension": {"answer": "Present", "rationale": "And the word \"I\" also indicates that it's a self-report of the testee. So, the time dimension of this entry can be judged now."}, "sentence_type": "Affirmative declarative sentence", "pos": {"nouns": {"The mood": 1, "Excited": 1, "Easier than I thought": 1}, "verbs": {"鈾?I'm gonna fall in 鈾?: 1}, "adverbs": {"3": 1, "very": 0}, "numerals": {"3": 1}}}, {"content": "I sometimes get excited and depressed at the same time.", "word_count": 13, "label": "", "sentence_structure": "Subject鈥搗erb鈥揷omplement", "subjective_objective": {"answer": "Subjective", "rationale": "Because \"excited\" and \"depressed\" are the feelings of the patient, it's a subjective examination."}, "sentence_sentiment": {"answer": "Neutral", "rationale": "Because the entry describes a possible emotional state, it does not explicitly express positive, negative or neutral emotional tendencies."}, "language_style": {"answer": "Tactful and warm", "rationale": "The language style of the entry is more euphemistic, leading each other to share their emotional experience in an invitation-style tone, and there is not much direct affirmation or denial, which is more inclusive and understanding."}, "time_dimension": {"answer": "Present", "rationale": "This sentence describes the feelings of the voice at some point, which both contain positive excitement and negative depression. So, the time dimension of the sentence is now."}, "sentence_type": "Affirmative declarative sentence", "pos": {"nouns": {"Excited": 1, "I'm depressed": 1}, "verbs": {"Sometimes": 1, "Yes": 1, "I think": 1, "Meanwhile": 1, "and": 1, "Say it": 1, "I": 1}}}, {"content": "I sometimes show a much greater interest in sex than usual.", "word_count": 18, "label": "", "sentence_structure": "Subject鈥搗erb鈥揷omplement", "subjective_objective": {"answer": "Subjective", "rationale": "Because interest is a subjective feeling and attitude that reflects the individual perception and feelings of sexuality.\""}, "sentence_sentiment": {"answer": "Negative", "rationale": "Because the \"more than usual interest in sex\" act in the entry may be considered excessive or unhealthy."}, "language_style": {"answer": "Tactful and warm", "rationale": "The language style of the entry is a euphemism, which enhances the sensitivity and interaction of the questionnaire by using \"sometimes\" and \"more than usual\" expressions, avoiding direct, over-heavy tone and in a more moderate way leading respondents to questions."}, "time_dimension": {"answer": "Past", "rationale": "In conclusion, the time dimension of this questionnaire entry is the past."}, "sentence_type": "Affirmative declarative sentence", "pos": {"nouns": {"Interest": 1, "Sex": 1}, "verbs": {"Yes": 1, "Performance": 1, "to": 1}, "adjectives": {"Bigger": 1}, "adverbs": {"Sometimes": 1}, "numerals": {"5": 1}}}, {"content": "My self-perceptions have changed between over-indulgentity and overconfidence.", "word_count": 20, "label": "", "sentence_structure": "Subject鈥搗erb鈥揷omplement", "subjective_objective": {"answer": "Subjective", "rationale": "Because \"too low\" and \"too self-confident\" are the feelings of the patient's own heart, and are subjective."}, "sentence_sentiment": {"answer": "Negative", "rationale": "Because the description of \"too low and too self-confident\" in the entries is negative."}, "language_style": {"answer": "Tactful and warm", "rationale": "The entry uses euphemism, using terms such as \"too low\" and \"too self-confident\" to describe the psychological state of the person under investigation, which is relatively gentle and friendly, and which better guides the interviewee to open his heart and participate in the answer. At the same time, the term \"change\" also shows a dynamic psychological state, not static, making the question more exploratory and open."}, "time_dimension": {"answer": "Present", "rationale": ""}, "sentence_type": "Affirmative declarative sentence", "pos": {"nouns": {"I": 1, "Self-feeling": 1, "I'm too low on myself": 1, "Overconfidence": 1}, "verbs": {"Change": 1}, "adverbs": {"In between": 1}, "numerals": {"6": 1}}}, {"content": "The quality and quantity of my work sometimes change very dramatically.", "word_count": 21, "label": "", "sentence_structure": "Subject鈥搗erb鈥揷omplement", "subjective_objective": {"answer": "Subjective", "rationale": "This description is based on the views and feelings of individuals and involves subjective concepts such as \"quality of work\" and \"quantity change\". At the same time, the entry does not provide any facts or data that would support whether it is objective."}, "sentence_sentiment": {"answer": "Negative", "rationale": "Because the expression \"the quality and quantity of work sometimes changes very significantly\", has some negative sentiment, which may indicate that respondents are dissatisfied with or under greater pressure to work."}, "language_style": {"answer": "Calm and direct", "rationale": "The language style of the entry is more objective and neutral, and there is no excessive emotional or rhetorical style, but rather a description of the situation at work in the form of a statement of facts, which gives a clearer picture of the nature of the problem."}, "time_dimension": {"answer": "Past", "rationale": "The entry describes the subjective perception of the quality and quantity of the work of the person being tested, the temporal dimension of which does not clearly point to the past, present or future and therefore cannot be judged accurately."}, "sentence_type": "Affirmative declarative sentence", "pos": {"nouns": {"Work": 1, "Quality": 1, "Number": 1}, "verbs": {"Come on": 1, "Change": 1}, "adjectives": {"Very": 1, "Large": 1}, "adverbs": {"Sometimes": 1}}}, {"content": "I sometimes get angry and hostile to others for no reason.", "word_count": 19, "label": "", "sentence_structure": "Subject鈥搗erb鈥揷omplement", "subjective_objective": {"answer": "Subjective", "rationale": "Because \"unjustified excitement\" and \"enemies to others\" are subjective experiences and emotional reactions of patients."}, "sentence_sentiment": {"answer": "Negative", "rationale": "Because the \"enemy to others\" attitude in the entry is negative."}, "language_style": {"answer": "Calm and direct", "rationale": "It expresses in a direct manner whether the other party is sometimes asked about emotional instability, which is more objective and balanced."}, "time_dimension": {"answer": "Past", "rationale": "The entry describes the behaviour or mentality of the person tested at some point in time, and does not clearly point to the present or the future. Thus, we can judge that the temporal dimension of the article is past."}, "sentence_type": "Affirmative declarative sentence", "pos": {"nouns": {"Excited": 1, "Others": 1, "Hostile": 1}, "verbs": {"None": 1, "Wing": 1, "have": 1}, "adverbs": {"Sometimes": 1}, "numerals": {"8": 1}, "pronouns": {"I": 1}}}, {"content": "I sometimes have a bad mind, and sometimes I have a flexible mind to have a lot of innovative insights.", "word_count": 26, "label": "", "sentence_structure": "Subject鈥搗erb鈥揷omplement", "subjective_objective": {"answer": "Objective", "rationale": "Because good and bad minds, flexibility of thinking and the availability of innovative ideas are the manifestations or experiences of patients in different contexts, and are objective facts."}, "sentence_sentiment": {"answer": "Positive", "rationale": "/ Neutral. Because the entry is a representation of a change of state and of mental ability, it is not clearly emotional and is therefore neutral."}, "language_style": {"answer": "Tactful and warm", "rationale": "The entry is a more modest and implicit query about the respondents' performance in terms of their ability to think and innovate, without directly evaluating or criticizing them, which is more personal and interactive. At the same time, the terms \"sometimes very insensitive\" and \"thinking and flexible enough to have a lot of innovative insights\" are also relatively euphemisms that express the subject of the problem while avoiding offensive or discomfort."}, "time_dimension": {"answer": "Past", "rationale": "The entry \"very bad mind\" and \"thinking flexible\" shows the difference in the testee's state at different times and can therefore be judged as the past."}, "sentence_type": "Affirmative declarative sentence", "pos": {"nouns": {"Brain": 1, "Innovation": 1, "Observations": 1}, "verbs": {"be": 1, "Sometimes": 2, "have": 1, "And": 2, "very": 1, "Present": 1}, "adjectives": {"It's not working": 1, "Flexibility": 1}, "adverbs": {"Sometimes": 2}, "pronouns": {"I": 1}}}, {"content": "I sometimes show a great deal of interest in interpersonal interaction, and sometimes I prefer to be alone.", "word_count": 27, "label": "", "sentence_structure": "Subject鈥搗erb鈥搊bject", "subjective_objective": {"answer": "Subjective", "rationale": "Because the sentence describes the patient鈥檚 subjective attitude and emotional state, which sometimes interest in interpersonal interaction and sometimes prefers to be alone. These are the feelings and choices that the patient feels and chooses, and are subjective."}, "sentence_sentiment": {"answer": "Neutral", "rationale": "Although the expressions \"sometimes\" and \"only one person is willing to be alone\" are somewhat negative, the whole entry means that social interest sometimes exists, which can be considered a positive attitude. So, overall, the entry is not clearly emotional and is neutral."}, "language_style": {"answer": "Tactful and warm", "rationale": "The entry is in a soft, interrogating language style. It does not directly order or criticize the interviewee鈥檚 interpersonal mode, but rather is a balanced way of understanding the character of the interviewee. Moreover, the purpose of the article is expressed in a manner that is more personal and interactive, and more readily acceptable to the interviewee and willing to answer questions."}, "time_dimension": {"answer": "Present", "rationale": "The keyword \"sometimes\", \"only willing\" indicates that it is a description of the person's current behaviour and mental state, and therefore the judgement is now."}, "sentence_type": "Affirmative declarative sentence", "pos": {"nouns": {"Human contact": 1, "Interest": 2, "person": 1, "Alone": 1}, "verbs": {"Performance": 1, "Yes": 1, "- To be with someone": 1, "in": 1, "Sometimes": 2}, "adjectives": {"Large": 1}, "adverbs": {"Only": 1}, "numerals": {"10": 1}, "pronouns": {"I": 1}}}, {"content": "My ideas have changed between extreme optimism and extreme pessimism.", "word_count": 18, "label": "", "sentence_structure": "Subject鈥揷opula鈥損redicative", "subjective_objective": {"answer": "Subjective", "rationale": "Because the descriptions of \"extreme optimism\" and \"extreme pessimism\" are the patient's personal emotions and thoughts, and are subjective."}, "sentence_sentiment": {"answer": "Negative", "rationale": "Because \"extreme optimism and pessimism\" in the entry is a negative emotional swing, indicating emotional instability."}, "language_style": {"answer": "Calm and direct", "rationale": "The entry directly asks the respondents in a concise language whether they often find themselves in two extremes (optimistic and pessimistic), without using excessive rhetoric or emotional rendering, and with a more calm and direct language style."}, "time_dimension": {"answer": "Present", "rationale": "The entry asks the testee whether there has been an extreme change in his/her views or attitudes, and therefore the temporal dimension is now judged."}, "sentence_type": "Affirmative declarative sentence", "pos": {"nouns": {"Perceptions": 1, "Optimistic": 1, "Pessimism": 1}, "verbs": {"Change": 1}, "adjectives": {"Extreme": 2}, "numerals": {"11": 1}}}, {"content": "I sometimes wake up with tears that I can't contain, and sometimes I laugh too much.", "word_count": 24, "label": "", "sentence_structure": "Subject鈥搗erb鈥揷omplement", "subjective_objective": {"answer": "Subjective", "rationale": "Because \"insolvency\" and \"excessive\" are the individual feelings and emotional reactions of the patient, this is viewed from the patient's subjective perspective."}, "sentence_sentiment": {"answer": "Neutral", "rationale": "\"Sometimes, the tears that can't be contained\" express positive emotional experiences, and \"too much love is joked\" may have negative emotional aspects, so the entry is both positive and negative and cannot simply be classified as positive, negative or neutral."}, "language_style": {"answer": "Tactful and warm", "rationale": "Because it uses narratives rather than orders or direct statements, it feels more friendly and friendly, and it expresses more emotional feelings, both depressing and positive. This euphemism is more resonant and participatory."}, "time_dimension": {"answer": "Present", "rationale": "The words \"sometimes\" and \"will\" and \"insolvable\" or \"or\" indicate that the act is somewhat repetitive and cyclical, and therefore judged as now."}, "sentence_type": "Affirmative declarative sentence", "pos": {"nouns": {"鈾?Tears to wake up 鈾?: 1, "You're kidding": 1}, "verbs": {"Yes": 3, "I can't help it": 1, "Excess": 1}, "adverbs": {"Sometimes": 2}, "numerals": {"12": 1}}}]}

{"questionnaire_title": "Speaker's confidence self-assessment", "questionnaire_category": "Mental health", "item_count": 30, "emotion_shift_overall": "From positive to negative", "language_style_overall": "Tactful and warm", "items": [{"content": "I look forward to the opportunity to speak before the public.", "word_count": 15, "label": "", "sentence_structure": "Subject鈥搗erb鈥搊bject", "subjective_objective": {"answer": "Subjective", "rationale": "Because \"hope\" is the patient's personal emotion and desire, and it's a subjective examination."}, "sentence_sentiment": {"answer": "Positive", "rationale": "Because the \"hope\" attitude in the entry is positive."}, "language_style": {"answer": "Tactful and warm", "rationale": "The entry uses euphemism, dialogue by asking each other's views or feelings, and is not directly presented or ordered, which is more personal and interactive. At the same time, the word \"hope\" reflects a positive and more intimate attitude."}, "time_dimension": {"answer": "Future", "rationale": "According to the phrase \"seeing\" and \"the opportunity to speak before the public\", it is an expectation or a plan for the future, and therefore a judgement for the future."}, "sentence_type": "Affirmative declarative sentence", "pos": {"nouns": {"I": 1, "The public": 1, "Speeches": 1, "Opportunities": 1}, "verbs": {"Hope": 1, "Before": 1, "Speeches": 1, "be": 1}, "adverbs": {"Here": 1}, "numerals": {"1": 3}, "pronouns": {"Here": 1}}}, {"content": "2. My hands are shaking as I take what is on the podium.", "word_count": 16, "label": "", "sentence_structure": "Subject鈥搗erb鈥揷omplement", "subjective_objective": {"answer": "Subjective", "rationale": "Because my hand is shaking is a personal experience and feeling, and it is viewed from the subjective perspective of the patient."}, "sentence_sentiment": {"answer": "Negative", "rationale": "Because the \"hand shaking\" act is negative."}, "language_style": {"answer": "Tactful and warm", "rationale": "The entry is more euphemistic, with more gentle language and expression, such as \"me\" rather than \"you\", and a descriptive term like \"weave\" as an alternative to expressing tension or anxiety directly, so that it avoids too direct and offensive language and can accurately convey each other's feelings."}, "time_dimension": {"answer": "Present", "rationale": "According to the context given to the title, the title is described as a handshake that occurs in an act \"when taking something from the podium\". As the action is ongoing and no information about the past or future is involved, the entry is now time-dimensional."}, "sentence_type": "Affirmative declarative sentence", "pos": {"nouns": {"From the rostrum": 1, "Something": 1, "Hands": 1}, "verbs": {"in": 1, "Fire": 1, "Take it": 1, "Wreck": 1}, "adjectives": {"Mine": 1}, "numerals": {"2锛?: 1, "in": 1}, "classifiers": {"individual": 1}}}, {"content": "I am always afraid to forget what I'm talking about.", "word_count": 13, "label": "", "sentence_structure": "Subject鈥揷opula鈥損redicative", "subjective_objective": {"answer": "Subjective", "rationale": "Because \"fear\" is a subjective emotional word that shows the fear and fear inside the patient."}, "sentence_sentiment": {"answer": "Negative", "rationale": "Because the \"fear to forget\" mood and attitude in the entry is negative."}, "language_style": {"answer": "Tactful and warm", "rationale": "The entry asks each other in an understanding and caring manner whether they are concerned about forgetting the content of the speech, in a more moderate, friendly way, without language that directly rejects or blames. Moreover, the issue is not too complex in terms of vocabulary and sentence structure to understand. The language style of the questionnaire entry can therefore be described as \"precipitous.\""}, "time_dimension": {"answer": "Present", "rationale": "The \"always\" in this entry indicates that this fear is a long-standing problem, and \"fear to forget\" is a current behavior or emotion. So the time dimension of this entry is now."}, "sentence_type": "Interrogative sentence", "pos": {"nouns": {"I": 1, "Speeches": 1, "Contents": 1}, "verbs": {"Always": 1, "Scared": 1, "Forget it": 1}, "adverbs": {"Always": 1}, "numerals": {"3": 1}}}, {"content": "4. When I spoke to the audience, they seemed friendly.", "word_count": 15, "label": "", "sentence_structure": "Subject鈥揷opula鈥損redicative", "subjective_objective": {"answer": "Objective", "rationale": "It looks at the response of the audience, it's an objective fact.\""}, "sentence_sentiment": {"answer": "Positive", "rationale": "Because the entry describes the kind of reaction that listeners have to respond to me when I speak, which is a positive emotional attitude."}, "language_style": {"answer": "Tactful and warm", "rationale": "The entry uses a subjective expression that \"they seem friendly\" and does not specify whether the other party is really friendly, which is euphemistic, avoids direct criticism or offensive to the other, and has some degree of kinship. At the same time, the sentence is simple, easy to understand and corresponds to the linguistic style of the psychological questionnaire."}, "time_dimension": {"answer": "Present", "rationale": "The sentence describes the feelings and observations of those who speak when speaking to the audience, which have already occurred and been completed, and thus the judgement is now."}, "sentence_type": "Affirmative declarative sentence", "pos": {"nouns": {"Audience": 1, "Speeches": 1, "Friendly": 1}, "verbs": {"When": 1, "to": 1, "Seems like": 1, "Yeah": 1}, "adjectives": {"Friendly": 1}, "adverbs": {"Yeah": 1}, "numerals": {"4": 1}, "pronouns": {"I": 1}}}, {"content": "5. I was in a state of anxiety when I was preparing my speech.", "word_count": 15, "label": "", "sentence_structure": "Subject鈥揷opula鈥損redicative", "subjective_objective": {"answer": "Subjective", "rationale": "Because \"always in an anxiety\" is a description of subjective feelings and experiences, viewed from the perspective of the patient."}, "sentence_sentiment": {"answer": "Negative", "rationale": "Because \"in an anxiety state\" is a negative emotional experience."}, "language_style": {"answer": "Calm and direct", "rationale": "The language style of the entry is calm, because it is a straightforward expression of whether the subject is anxious when the person is preparing his or her speech. There is no rhetorical or emotional rendering, but the problem is expressed in simple and quick language."}, "time_dimension": {"answer": "Present", "rationale": "The keyword \"always\" and \"in\" means that it's an act or mentality that is happening, so the time dimension of the purpose is now."}, "sentence_type": "Affirmative declarative sentence", "pos": {"nouns": {"Speeches": 1, "Anxiety status": 1}, "verbs": {"Ready": 1, "in": 1}, "numerals": {"5": 1}}}, {"content": "6. I ended with a pleasant experience.", "word_count": 16, "label": "", "sentence_structure": "Subject鈥揷opula鈥損redicative", "subjective_objective": {"answer": "Subjective", "rationale": "Because it's a subjective feeling to be happy."}, "sentence_sentiment": {"answer": "Positive", "rationale": "Because the entries describe a happy experience, it's a positive emotional color."}, "language_style": {"answer": "Tactful and warm", "rationale": "The entry is more personal and interactive, based on a description of personal feelings and using a gentle and inviting tone for dialogue rather than direct statements or orders."}, "time_dimension": {"answer": "Present", "rationale": "The questionnaire entry asked the respondents how they felt at the end of the presentation, indicating the behaviour or state of the person in progress and thus judging it as now."}, "sentence_type": "Affirmative declarative sentence", "pos": {"nouns": {"Speeches": 1, "End": 1, "Experience": 1}, "verbs": {"I feel": 1, "be": 1}, "adjectives": {"Have a nice day": 1}, "pronouns": {"I": 1}}}, {"content": "I do not like to express myself in my body or in my voice.", "word_count": 16, "label": "", "sentence_structure": "Subject鈥搗erb鈥揷omplement", "subjective_objective": {"answer": "Subjective", "rationale": "Because \"not like\" is a subjective feeling or attitude."}, "sentence_sentiment": {"answer": "Neutral", "rationale": "Because the entry does not have any emotional color."}, "language_style": {"answer": "Calm and direct", "rationale": "The language style of this questionnaire entry is calm and straightforward. The entry clearly indicates whether the other party does not like to use body and voice to express his will, and is speaking objectively and without excessive emotional rendering."}, "time_dimension": {"answer": "Present", "rationale": "The keyword is \"not like,\" which means that the testee is dissatisfied with the state of the moment, implying that it is a description of the current state of affairs. And, \"expression with my body and voice\" is also a description of the current way of acting. So the temporal dimension of this article should be judged as \"now\"."}, "sentence_type": "Negative declarative sentence", "pos": {"nouns": {"Body": 1, "Sound": 1}, "verbs": {"I don't like it": 1, "Use": 1, "Conduct": 1, "Expression": 1}}}, {"content": "8. When I spoke in front of an audience, my mind began to be confused and incoherent.", "word_count": 23, "label": "", "sentence_structure": "Subject鈥揷opula鈥損redicative", "subjective_objective": {"answer": "Subjective", "rationale": "The question is about the patient's subjective feelings and experiences."}, "sentence_sentiment": {"answer": "Negative", "rationale": "Because the entry describes a negative behaviour or state of mind, i.e. confusion and incoherence."}, "language_style": {"answer": "Tactful and warm", "rationale": "The entry is more personal and interactive, by means of a gentle, invited tone of speech rather than a direct statement or order. At the same time, it uses a more objective and neutral language to describe the interviewee鈥檚 feelings, without too much emotional or subjective evaluation, and is more in keeping with the euphemism of the language."}, "time_dimension": {"answer": "Present", "rationale": ""}, "sentence_type": "Affirmative declarative sentence", "pos": {"nouns": {"Audience": 1, "Thinking": 2, "Chaos": 1, "Incoherence": 1}, "verbs": {"in": 1, "Say it": 1, "Start": 1, "Chaos": 1, "Incoherence": 1}, "adjectives": {"One": 1}, "numerals": {"8": 1}, "pronouns": {"I": 1}}}, {"content": "I am not afraid to face the audience.", "word_count": 8, "label": "", "sentence_structure": "Subject鈥搗erb鈥搊bject", "subjective_objective": {"answer": "Subjective", "rationale": "Because fear is the patient's subjective attitude."}, "sentence_sentiment": {"answer": "Positive", "rationale": "Because the \"near to face the audience\" attitude in the entry is positive."}, "language_style": {"answer": "Tactful and warm", "rationale": "The language style of the entry is more euphemistic, expressing confidence, courage and the specific context of \"no fear\" by using the negative word, and directing the interviewee to think about his or her ability to speak. This expression is more friendly, encouraging and easier to ask the questions."}, "time_dimension": {"answer": "Present", "rationale": ""}, "sentence_type": "Affirmative declarative sentence", "pos": {"nouns": {"Audience": 1}, "verbs": {"Face it": 1, "Not afraid": 1}, "adverbs": {"not": 1}, "pronouns": {"I": 1}}}, {"content": "10. Although I was nervous before I got up, I soon forgot to be afraid and liked the experience.", "word_count": 31, "label": "", "sentence_structure": "Subject鈥揷opula鈥損redicative", "subjective_objective": {"answer": "Subjective", "rationale": "The basis for the judgment: because \"frightful\" and \"forgetting afraid and enjoying it\" are the personal feelings and attitudes of the patient to this experience, this is a subjective examination."}, "sentence_sentiment": {"answer": "Positive", "rationale": "Because it describes the participants as quickly forgetting fear and enjoying experience and expressing positive attitudes and feelings."}, "language_style": {"answer": "Tactful and warm", "rationale": ""}, "time_dimension": {"answer": "Past", "rationale": "\"Although I\" indicates that this is a memory and description of past experience, so the temporal dimension of the article is the past."}, "sentence_type": "Affirmative declarative sentence", "pos": {"nouns": {"I'm nervous": 1, "Experience": 1}, "verbs": {"Stand up": 1, "Forget it": 1, "Yeah": 1}, "adverbs": {"Soon": 1, "and": 1}, "numerals": {"10": 1}, "pronouns": {"I": 1}}}, {"content": "I look forward to the confidence I will have in my presentation.", "word_count": 14, "label": "", "sentence_structure": "Subject鈥搗erb鈥搊bject", "subjective_objective": {"answer": "Subjective", "rationale": "Because \"expectation\" and \"confident\" are the expectations and feelings inside the patient, and they are subjective."}, "sentence_sentiment": {"answer": "Positive", "rationale": "Because the attitude of \"seeing confidence in speaking\" in the entries is positive."}, "language_style": {"answer": "Tactful and warm", "rationale": "The words are more euphemistic, more euphemistic, and use words like \"expected\" and \"capable\" to express expectations and aspirations for the future, and do not directly use strong emotional or command-style tone. Such expressions are more friendly and friendly and help to create a good atmosphere of communication."}, "time_dimension": {"answer": "Future", "rationale": ""}, "sentence_type": "Affirmative declarative sentence", "pos": {"nouns": {"Speeches": 1, "Confidence": 1}, "verbs": {"Expectations": 1, "Yes": 1, "Fill": 1}, "numerals": {"11": 1}, "pronouns": {"I": 1}}}, {"content": "12. When I spoke, I felt I had my whole heart and soul.", "word_count": 16, "label": "", "sentence_structure": "Subject鈥搗erb鈥揷omplement", "subjective_objective": {"answer": "Subjective", "rationale": "Because \"feel\" is usually a subjective feeling or experience of a person, not an objective fact or result. In this sentence, \"I feel I have my whole body and soul\" is a patient's description of the subjective feelings that I have in the speech."}, "sentence_sentiment": {"answer": "Positive", "rationale": "Because the entry \"I feel I have my whole heart\" expresses a positive emotional attitude."}, "language_style": {"answer": "Tactful and warm", "rationale": "Although the entry directly asks the person who was interviewed about his speech, the tone is more moderate and encouraging. The sentence uses a positive expression like \"I feel I have my whole body and soul,\" which does not mean criticism or blame. This language style is more likely to stimulate the positive feelings of the person under investigation, and thus to gather more effective information."}, "time_dimension": {"answer": "Present", "rationale": "\"When it is\" means an action or state that is in progress or is taking place, and \"I feel\" also uses the normal current state of affairs, which means that it is the current feeling or state of the situation. So, the entry to the questionnaire asks the person who was being interviewed for the present feeling in his speech, and the temporal dimension is now."}, "sentence_type": "Affirmative declarative sentence", "pos": {"nouns": {"When": 1, "I": 2, "Speeches": 1, "Feel": 1, "I'm all I'm ever gonna be": 1}, "verbs": {"Speeches": 1, "Feel": 1, "Possession": 1}, "adverbs": {"Time": 1}, "numerals": {"12": 1}, "pronouns": {"I": 2}}}, {"content": "13. I would like to put the notebook on the podium in case I forget the speech.", "word_count": 23, "label": "", "sentence_structure": "Subject鈥搗erb鈥搊bject", "subjective_objective": {"answer": "Subjective", "rationale": "Because \"like\" is the subjective aspect of the patient's personal feelings and attitude towards putting the notebook on the podium."}, "sentence_sentiment": {"answer": "Positive", "rationale": "Because the behaviour in the entry is positive, i.e., being prepared to adequately address possible problems."}, "language_style": {"answer": "Tactful and warm", "rationale": "The language style of the entry is euphemism, because it uses expressions such as \"like\" and \"in case of case\" to describe the behaviour and attitude of the dominant language, without directly stating or ordering the other to do so. This is more personal and interactive."}, "time_dimension": {"answer": "Past", "rationale": ""}, "sentence_type": "Affirmative declarative sentence", "pos": {"nouns": {"From the rostrum": 1, "The notebook": 1, "Speeches": 1}, "verbs": {"Yeah": 1, "Fire": 1, "Forget it": 1}, "adverbs": {"What if": 1}, "numerals": {"13": 1}, "classifiers": {"individual": 1}, "pronouns": {"I": 1}}}, {"content": "14. I would like to observe the audience ' s reaction when I speak.", "word_count": 14, "label": "", "sentence_structure": "Subject鈥搗erb鈥搊bject", "subjective_objective": {"answer": "Subjective", "rationale": "Because \"like\" is a personal emotion and attitude, it is subjective. And when you talk about the audience's reaction is an act or an act, it describes what is objectively present or what is happening, so the subject of this questionnaire is subjective."}, "sentence_sentiment": {"answer": "Positive", "rationale": "Because the \"like to watch the audience's reaction during the speech\" act in the entry is proactive."}, "language_style": {"answer": "Tactful and warm", "rationale": "The entry is more personal and interactive when it is being approached in a gentle and inviting manner, asking whether the other side prefers to observe the audience ' s reaction when speaking, rather than directly speaking or ordering."}, "time_dimension": {"answer": "Present", "rationale": "The words \"like\" and \"observation\" in this entry are generally current, indicating the present behaviour or habits of the testee, and therefore judging as now."}, "sentence_type": "Affirmative declarative sentence", "pos": {"nouns": {"I": 1, "Speeches": 1, "Response": 1}, "verbs": {"Yeah": 1, "Observation": 1}, "numerals": {"14": 1}}}, {"content": "15. Although I speak well with my friends, I drop my tumblings when I speak to the podium.", "word_count": 28, "label": "", "sentence_structure": "Subject鈥搗erb鈥搊bject", "subjective_objective": {"answer": "Subjective", "rationale": "Although it is the patient ' s subjective feeling to talk to his friends, the drops and drops in the speech on the stage are the objective result."}, "sentence_sentiment": {"answer": "Negative", "rationale": "Because the \"Drill Four\" act in the entry is negative."}, "language_style": {"answer": "Tactful and warm", "rationale": "The entry uses a relatively mild language style to describe the ability of the interviewee to express himself in different situations. It does not directly point to the respondent's shortcomings, but rather uses a more objective presentation that allows the interviewee to accept the problem more naturally and may be more willing to share his or her real feelings and ideas."}, "time_dimension": {"answer": "Present", "rationale": "The entry now describes the current behaviour or state of the subject, namely, \"I'm speaking with my friends, and I'm throwing away my feet when I speak to the podium\" and is a visual look at the present."}, "sentence_type": "Affirmative declarative sentence", "pos": {"nouns": {"Friends": 1, "Talk": 1, "From the rostrum": 1, "Speeches": 1}, "verbs": {"鈾?Though 鈾?: 1, "and": 1, "Fluid": 1, "Drop it": 1}, "adverbs": {"But": 1}, "numerals": {"15": 1}}}, {"content": "16. I was relieved and comfortable with my presentation.", "word_count": 12, "label": "", "sentence_structure": "Subject鈥揷opula鈥損redicative", "subjective_objective": {"answer": "Subjective", "rationale": "Because feeling relaxed and comfortable is a subjective or emotional experience."}, "sentence_sentiment": {"answer": "Positive", "rationale": "Because the entry \"relax and comfortable\" describes positive emotions and attitudes."}, "language_style": {"answer": "Tactful and warm", "rationale": "The entry is more adept and interactive when it is spoken in a gentle and inviting manner, rather than by direct statements or orders."}, "time_dimension": {"answer": "Present", "rationale": "The keyword \"in the speech\" denotes the behaviour currently being performed, so the temporal dimension is now judged."}, "sentence_type": "Affirmative declarative sentence", "pos": {"nouns": {"Speeches": 1, "Time": 1, "I feel": 1, "Relax": 1, "and": 1, "Comfortable": 1}, "verbs": {"I": 1}, "adjectives": {"Relax": 1, "Comfortable": 1}, "numerals": {"16": 1}}}, {"content": "17. Although I do not like speaking in public, I am not particularly afraid of it.", "word_count": 22, "label": "", "sentence_structure": "Subject鈥揷opula鈥損redicative", "subjective_objective": {"answer": "Subjective", "rationale": "The purpose of this questionnaire is to judge subjective. Because, although this person does not like speaking in public, he is not particularly afraid of it, which is his subjective attitude and feelings."}, "sentence_sentiment": {"answer": "Positive", "rationale": "Because \"no special fear\" in the entry indicates that the voice has the ability and courage to overcome the difficulties."}, "language_style": {"answer": "Tactful and warm", "rationale": "The entry uses a more moderate tone, which both expresses the respondent鈥檚 dislike of public speaking and shows that he is not particularly afraid or troubled by it. This expression is relatively euphemism and considerate, and makes it easier for the interviewee to share his real thoughts."}, "time_dimension": {"answer": "Present", "rationale": "\"Not though it is\" indicates that the speaker has some attitude or view of something he does not like, and that such attitude or view still exists. Thus, the act of \"not being particularly afraid\" occurs now, so the entry describes the situation now."}, "sentence_type": "Negative declarative sentence", "pos": {"nouns": {"Questionnaire Entry": 1, "Public": 1, "Speeches": 2}, "verbs": {"Yeah": 1, "Fear": 1, "in": 1, "Special": 1, "No, it's not": 1, "to": 1}, "adjectives": {"not": 2, "Special": 1, "Before the public": 1, "Fear": 1}, "adverbs": {"鈾?Though 鈾?: 1, "Yeah": 2}, "numerals": {"17": 1, "2": 1}, "pronouns": {"鈾?And the 鈾?: 1, "I": 1}}}, {"content": "18. I always try to avoid speaking in public, if possible.", "word_count": 17, "label": "", "sentence_structure": "Subject鈥搗erb鈥搊bject", "subjective_objective": {"answer": "Subjective", "rationale": "Because \"defusing\" and \"public speaking\" are patients' subjective thoughts and behavior."}, "sentence_sentiment": {"answer": "Negative", "rationale": "Because the \"avoid speaking in public\" act in the entry is negative."}, "language_style": {"answer": "Tactful and warm", "rationale": "The language style of the entry is more euphemistic, as it does not directly question the shortcomings or deficiencies of the individual, but rather questions the person under investigation in a more subtle and respectful manner. Moreover, the use of the expression \"if possible\" makes the issue more moderate and does not put excessive pressure on the person under investigation."}, "time_dimension": {"answer": "Present", "rationale": "The entry expresses a customary act or tendency of the subject, \"to avoid as much as possible\", which may have occurred in the past, but which places greater emphasis on the current situation. At the same time, the sentence does not address the concept of the future time, so the temporal dimension is now judged."}, "sentence_type": "Affirmative declarative sentence", "pos": {"nouns": {"Maybe": 1, "Speeches": 1}, "verbs": {"Avoid": 1, "Try": 1}, "adjectives": {"Public": 1, "Front": 1}, "adverbs": {"Always": 1, "in": 1}, "pronouns": {"I": 1}}}, {"content": "19. As I looked to my listeners, their faces became blurred.", "word_count": 23, "label": "", "sentence_structure": "Subject鈥搗erb鈥揷omplement", "subjective_objective": {"answer": "Subjective", "rationale": "Because \"insinuity\" is a subjective feeling or experience."}, "sentence_sentiment": {"answer": "Negative", "rationale": "Because the description \"face is insensitive\" is negative."}, "language_style": {"answer": "Tactful and warm", "rationale": "The entry uses descriptive language to describe what is observed, rather than directly evaluating or criticizing. At the same time, the tone is more calm and less subjective. Thus, the language style of the purpose is \"pretent.\""}, "time_dimension": {"answer": "Present", "rationale": "The entry describes the feelings and experiences of the person who is being tested when observing the audience, and involves memories or perceptions of the past. The temporal dimension is therefore a past."}, "sentence_type": "Affirmative declarative sentence", "pos": {"nouns": {"Questionnaire Entry": 1, "Listeners": 1, "Face": 1}, "verbs": {"Dynamite": 1, "Look over there": 1, "鈾?And become 鈾?: 1, "Fuzzy": 1}, "adjectives": {"Mine": 1}, "numerals": {"19": 1}, "pronouns": {"I": 1}}}, {"content": "20. After I tried to speak to a group of people, I was disgusted by myself.", "word_count": 21, "label": "", "sentence_structure": "Subject鈥搗erb鈥揷omplement", "subjective_objective": {"answer": "Subjective", "rationale": "Because it's a subjective emotional experience."}, "sentence_sentiment": {"answer": "Negative", "rationale": "Because the \"repulsion\" in the entry is negative."}, "language_style": {"answer": "Calm and direct", "rationale": "The entry uses the term \"averse\" with a direct expression and a strong emotional colour, does not use euphemism, ambiguity or accommodative language, and is more strongly and clearly worded; 2. The entry expresses a negative emotional experience, i.e., a sense of great dissatisfaction and frustration after failing to speak. This emotional expression is consistent with the straightforward linguistic style of calmness."}, "time_dimension": {"answer": "Past", "rationale": "The entry is to ask if the person tested has felt this in his or her past experience and therefore belongs to the temporal dimension of the past."}, "sentence_type": "Negative declarative sentence", "pos": {"nouns": {"Questionnaire": 1, "Entry": 1, "person": 1, "Speeches": 1, "Myself": 1}, "verbs": {"Try": 1, "to": 1, "I feel": 1, "Disgusting": 1}, "adjectives": {"Group": 1}, "adverbs": {"Both": 1}}}, {"content": "I like the preparation of the presentations.", "word_count": 10, "label": "", "sentence_structure": "Subject鈥搗erb鈥揷omplement", "subjective_objective": {"answer": "Subjective", "rationale": "Because \"like\" is a personal emotional attitude and opinion, a patient's subjective feeling."}, "sentence_sentiment": {"answer": "Positive", "rationale": "Because the \"like\" in the entry expresses a positive attitude."}, "language_style": {"answer": "Tactful and warm", "rationale": "The entry is in a positive language, and \"I like\" expresses the other's love for the preparation of the speech, gives a warmer and warmer feeling."}, "time_dimension": {"answer": "Present", "rationale": "The words \"like\" and \"preparation\" in the entry indicate the behaviour or attitude currently being taken, so it can be judged as now."}, "sentence_type": "Affirmative declarative sentence", "pos": {"nouns": {"Ready": 1, "Work": 1, "Speeches": 1}, "verbs": {"Yeah": 1, "Do it": 1}, "adverbs": {"EMPTY": 1}, "numerals": {"21": 1}}}, {"content": "22. When I look at the audience, my mind is sober.", "word_count": 15, "label": "", "sentence_structure": "Subject鈥揷opula鈥損redicative", "subjective_objective": {"answer": "Subjective", "rationale": "Because \"sane brain\" is a subjective feeling or perception, not an objective fact. Although it describes experience or reaction in a given situation, it is still determined by the individual's own feelings and perceptions and is therefore considered subjective."}, "sentence_sentiment": {"answer": "Positive", "rationale": "Because the term \"sane brain\" is usually positive and positive."}, "language_style": {"answer": "Tactful and warm", "rationale": "The language style of the entry is euphemism. Use the word \"sensitization\" to express confidence and recognition of your state. At the same time, the tone of the sentence is more modest, with little embellishment or emotional colour, and it is a feeling of kindness and a growing sense of courtesy."}, "time_dimension": {"answer": "Present", "rationale": ""}, "sentence_type": "Affirmative declarative sentence", "pos": {"nouns": {"Audience": 1, "Brain": 1}, "verbs": {"Face it": 1, "be": 1, "Wake up": 1}, "numerals": {"22": 1}}}, {"content": "23: I'm pretty fluent.", "word_count": 7, "label": "", "sentence_structure": "Subject鈥搗erb鈥揷omplement", "subjective_objective": {"answer": "Subjective", "rationale": "Since fluidity is usually the level of competence that individuals feel subjectively, and is based on their own assessment of speech ability, the answer to this article is subjective."}, "sentence_sentiment": {"answer": "Positive", "rationale": "According to the above definition, it is a positive expression because \"I speak fairly fluently\" expresses the fluent and clear language of speech."}, "language_style": {"answer": "Calm and direct", "rationale": "The language style of the entry is relatively simple, not too much phrasing or emotional, and it appears to be more calm and straightforward."}, "time_dimension": {"answer": "Present", "rationale": "The entry now describes the conduct of the speaker at a certain point in time in the past and uses the usual past pattern of \"speak\" in the narrative to indicate that the act has been completed, but it is impossible to determine when it occurred, because there is no clear point of reference for time, and therefore only for now."}, "sentence_type": "Affirmative declarative sentence", "pos": {"verbs": {"Say it": 1, "Fluid": 1}, "adjectives": {"Pretty much": 1}, "adverbs": {"very": 1}, "numerals": {"23": 1}, "pronouns": {"I": 1}}}, {"content": "24. I was sweating when I was about to begin my presentation.", "word_count": 12, "label": "", "sentence_structure": "Subject鈥搗erb鈥揷omplement", "subjective_objective": {"answer": "Subjective", "rationale": "Because \"sweaking\" is a physical reaction and a sense of experience of the patient, it is a subjective approach."}, "sentence_sentiment": {"answer": "Negative", "rationale": "Because the expression \"sweeting\" in the entries expresses tension, fear, and is negative."}, "language_style": {"answer": "Calm and direct", "rationale": "The entry directly expresses the physical reaction of the person who is asking about the speech, does not use excessive rhetorical or emotional rendering, and is more objective in tone."}, "time_dimension": {"answer": "Present", "rationale": "\"To start\" means what's about to happen, which corresponds to \"now\"."}, "sentence_type": "Affirmative declarative sentence", "pos": {"nouns": {"Speeches": 1, "Sweat": 1, "Whim": 1}, "verbs": {"Start": 1, "# Wresh #": 1, "Sweat": 1}, "adverbs": {"Just": 1, "Yes": 1}, "numerals": {"24": 1}}}, {"content": "25. I feel that my position is rigid.", "word_count": 12, "label": "", "sentence_structure": "Subject鈥揷opula鈥損redicative", "subjective_objective": {"answer": "Subjective", "rationale": "Because the word \"think\" suggests that it is a subjective observation and feeling, not an objective fact or description."}, "sentence_sentiment": {"answer": "Negative", "rationale": "Because the expression \"standing is not natural\" in the entry is negative."}, "language_style": {"answer": "Tactful and warm", "rationale": ""}, "time_dimension": {"answer": "Present", "rationale": ""}, "sentence_type": "Affirmative declarative sentence", "pos": {"nouns": {"Position": 1, "Stunning": 1, "It's not natural": 1}, "verbs": {"I think": 1, "Stunning": 1, "It's not natural": 1}, "numerals": {"25": 1}}}, {"content": "26. I was always afraid and nervous when I spoke in front of the crowd.", "word_count": 17, "label": "", "sentence_structure": "Subject鈥揷opula鈥損redicative", "subjective_objective": {"answer": "Subjective", "rationale": "Because fear and stress are subjective emotional experiences, it reflects the patient ' s inner feeling of speaking in front of the crowd."}, "sentence_sentiment": {"answer": "Negative", "rationale": "Because the attitude and feeling of \"always afraid and nervous\" in the entry is negative."}, "language_style": {"answer": "Calm and direct", "rationale": "The language style of the entry is relatively simple, there is no excessive emotional modification or rhetorical technique, and it is more objective and direct in tone, and therefore can be judged as a \"cool straight\" type of questionnaire."}, "time_dimension": {"answer": "Present", "rationale": "The entry now describes the current emotions and experiences of the testees, that is, the feelings of fear and stress that they feel when speaking in front of the crowd. While these feelings may be the result of past experience or memory, they are considered to be the current state and experience of the testee in this matter. So the time dimension is now."}, "sentence_type": "Affirmative declarative sentence", "pos": {"nouns": {"Population": 1, "Speeches": 1, "Scared": 1, "Nervous": 1}, "verbs": {"I feel": 1, "Always": 1, "I'm speaking before": 1}, "adverbs": {"Always": 1, "and": 1}, "numerals": {"26": 1}, "pronouns": {"I": 1}}}, {"content": "I find it a little pleasant to have a presentation.", "word_count": 13, "label": "", "sentence_structure": "Subject鈥搗erb鈥揷omplement", "subjective_objective": {"answer": "Subjective", "rationale": "Because \"happy\" is subjective."}, "sentence_sentiment": {"answer": "Positive", "rationale": "Because the entry \"Looking forward to a nice speech\" expresses a positive emotional attitude."}, "language_style": {"answer": "Tactful and warm", "rationale": "This entry answers the question in a more relaxed way, using the neutral term \"slightly\" and not expressing feelings or attitudes too strongly. This expression is relatively euphemismal, moderate, and does not put too much pressure or discomfort on the respondents."}, "time_dimension": {"answer": "Past", "rationale": "The entry is a reference to the feelings and experiences of the subject at a certain point in the past, and the key words are \"discover\" and \"see\" and \"a little pleasure\" and they all point to what has happened."}, "sentence_type": "Affirmative declarative sentence", "pos": {"nouns": {"Speeches": 1, "Have a nice day": 1}, "verbs": {"I realized": 1, "Look forward to it": 1}, "adverbs": {"A little": 1}, "pronouns": {"I": 1}}}, {"content": "28. It is difficult for me to find the right words to express my thoughts in a calm manner.", "word_count": 24, "label": "", "sentence_structure": "Subject鈥揷opula鈥損redicative", "subjective_objective": {"answer": "Subjective", "rationale": "Because \"It's hard to find the right words to express my thoughts in a calm manner\" is the patient's own feelings and thoughts, and it's a subjective examination."}, "sentence_sentiment": {"answer": "Negative", "rationale": "Because the description \"it is difficult to find the right words in a calm manner\" in the entry is negative and indicates that there are some difficulties and challenges."}, "language_style": {"answer": "Tactful and warm", "rationale": "The entry is more adept and interactive when it is spoken in a gentle and inviting manner, rather than by direct statements or orders."}, "time_dimension": {"answer": "Present", "rationale": ""}, "sentence_type": "Affirmative declarative sentence", "pos": {"nouns": {"Questionnaire": 1, "Entry": 1, "Thinking": 1}, "verbs": {"Found it": 1, "Expression": 1, "Calm down": 1}, "adjectives": {"Suits": 1, "of": 2}, "adverbs": {"very": 1, "Land": 2}, "numerals": {"28": 1}, "pronouns": {"I": 1}}}, {"content": "I was afraid when I thought about speaking in front of the crowd.", "word_count": 15, "label": "", "sentence_structure": "Subject鈥揷opula鈥損redicative", "subjective_objective": {"answer": "Subjective", "rationale": "Because fear is a subjective emotional response."}, "sentence_sentiment": {"answer": "Negative", "rationale": "Because the \"fear\" sentiment in the entry is negative."}, "language_style": {"answer": "Tactful and warm", "rationale": "The entry expresses the inner fear and tension of the interviewee in a friendly manner by using the first-person pronoun \"I\" and the term \"fear\" describing personal feelings, while also reflecting the attention and support of the surveyer. This language is more personal and emotional, helping to create a good atmosphere of investigation and increase the participation of the interviewee."}, "time_dimension": {"answer": "Present", "rationale": "The term \"the thought\" suggests that this is a subjective feeling, which is now being experienced and thus the temporal dimension of the article can be judged to be the present."}, "sentence_type": "Affirmative declarative sentence", "pos": {"nouns": {"Population": 1, "Speeches": 1, "Fear": 1}, "verbs": {"Yes": 1, "I feel": 1, "Speeches": 1}, "numerals": {"29": 1}, "pronouns": {"I": 1}}}, {"content": "30. I have a sense of agility in the face of listeners.", "word_count": 16, "label": "", "sentence_structure": "Subject鈥揷opula鈥損redicative", "subjective_objective": {"answer": "Subjective", "rationale": "This is a perception of the patient ' s subjective experience and feelings and of his or her emotional state."}, "sentence_sentiment": {"answer": "Positive", "rationale": "Because the expression \"mutely\" in the entry is positive."}, "language_style": {"answer": "Tactful and warm", "rationale": "The entry uses subjectively defined feelings, and learns about their performance by asking if the other person feels sensitive to the audience. This way gives the interviewee a sense of respect and understanding, and a greater willingness to share real thoughts and feelings. So the language style of the entry is euphemism."}, "time_dimension": {"answer": "Present", "rationale": ""}, "sentence_type": "Affirmative declarative sentence", "pos": {"nouns": {"Audience": 1, "Thinking": 1, "Feel": 1}, "verbs": {"have": 1, "Agility": 1}, "adjectives": {"Sensitivity": 1}, "numerals": {"30": 1}, "pronouns": {"I": 1}}}]}

{"questionnaire_title": "One likes to rotate himself for a long time.", "questionnaire_category": "Mental health", "item_count": 56, "emotion_shift_overall": "From positive to negative", "language_style_overall": "Calm and direct", "items": [{"content": "Learn to do one simple thing, but soon forget it.", "word_count": 15, "label": "", "sentence_structure": "Subject鈥搗erb鈥搊bject", "subjective_objective": {"answer": "Subjective", "rationale": "Because \"learning\" and \"fast forget\" are subjective feelings and observations of patients, and they are subjective."}, "sentence_sentiment": {"answer": "Neutral", "rationale": "As the entry describes only one act or skill, it does not express any positive or negative emotional, attitude or value judgement."}, "language_style": {"answer": "Tactful and warm", "rationale": "The entry is more adept and interactive when it is spoken in a gentle and inviting manner, rather than by direct statements or orders."}, "time_dimension": {"answer": "Present", "rationale": "The entry is for questioning the behaviour or habits of the person being tested, and the time dimension is now judged. Key words \"learning to do\" , \"simplistic things\" and \"forgetting out\" point to the situation that is currently under way, without reference to the time period of the past or the future. So the time dimension of this questionnaire entry is now."}, "sentence_type": "Affirmative declarative sentence", "pos": {"nouns": {"Yes": 1}, "verbs": {"Learn": 1, "Do it": 1, "Forget it": 1, "Come on": 1}, "adjectives": {"Simple": 1}, "numerals": {"Two": 1}}}, {"content": "3 There are often no environmental exposures or requests for interaction.", "word_count": 16, "label": "", "sentence_structure": "Subject鈥搗erb鈥揷omplement", "subjective_objective": {"answer": "Subjective", "rationale": "Because \"require\" is a subjective attitude or need."}, "sentence_sentiment": {"answer": "Negative", "rationale": "Because the act of \"no environmental exposure or requirement of communication\" in the entry is negative."}, "language_style": {"answer": "Calm and direct", "rationale": "The entry directly expresses in a concise language the subject of whether the subject of the survey is often not exposed to the environment or required to interact, the temperature of the language and the absence of excessive rhetoric."}, "time_dimension": {"answer": "Present", "rationale": "The keyword is \"frequent\" , indicating that the act is normal or frequent at this stage; at the same time, the word \"require\" implies that the act may be passive, externally imposed pressure or restriction. The combination of considerations is intended to mean that the person being tested is often not exposed to the environment or to the need or opportunity to interact at this stage."}, "sentence_type": "Negative declarative sentence", "pos": {"nouns": {"Request": 1, "Exposure": 1, "Environment": 1, "- To be with someone": 1}, "verbs": {"No, I'm not": 1, "Conduct": 1, "Exposure": 1}, "adverbs": {"Regular": 1}, "numerals": {"3": 1}}}, {"content": "4 Often cannot accept simple instructions (e.g. sit down, come over, etc.)", "word_count": 17, "label": "", "sentence_structure": "Subject鈥搗erb鈥揷omplement", "subjective_objective": {"answer": "Subjective", "rationale": "Because the simpleness of the capacity to accept is the subjective feeling and judgement of the individual."}, "sentence_sentiment": {"answer": "Negative", "rationale": "Because the \"unable to accept simple instructions\" act in the entry is negative."}, "language_style": {"answer": "Calm and direct", "rationale": "The language style of the entry is more straightforward, with no excessive rhetorical or emotional rendering, but simply a description of an observed phenomenon. This expression makes the issue clearer and more concise."}, "time_dimension": {"answer": "Past", "rationale": "The keyword \"unacceptability\" indicates that this occurred in the past and may have affected the conduct of the person tested. The entry is therefore a matter of past practice to ask whether the person tested was often unable to accept simple instructions in the past."}, "sentence_type": "Negative declarative sentence", "pos": {"nouns": {"Command": 1, "Sit down": 1, "Come here": 1}, "verbs": {"Accept": 1, "Here": 1}, "adjectives": {"Simple": 1}, "adverbs": {"Often": 1}, "numerals": {"4": 1}}}, {"content": "Five doesn't play toys.", "word_count": 17, "label": "", "sentence_structure": "Subject鈥搗erb鈥揷omplement", "subjective_objective": {"answer": "Objective", "rationale": "Because \"no play toys\" is a behavioral or observational result, not a patient's subjective attitude or experience. This is an objective fact observed by a doctor or other researcher, and therefore an objective entry."}, "sentence_sentiment": {"answer": "Negative", "rationale": "Because the behaviour of \"no toy playing\" is negative, it means that children lack some skills and social skills."}, "language_style": {"answer": "Calm and direct", "rationale": "The entry is a direct indication of the more objective and concise language style and the lack of excessive rhetorical or emotional colours for the correct use of toys by the interviewees."}, "time_dimension": {"answer": "Present", "rationale": "The entry is judged by the keyword \"no\" and the verb \"play toy\" to be an inquiry into whether the person tested is now playing with certain specific types of toys and is therefore a current behavioural or attitude inquiry."}, "sentence_type": "Negative declarative sentence", "pos": {"nouns": {"Toys": 2, "Turn": 1, "Throw it": 1, "Scram": 1}, "verbs": {"No, I won't": 1, "Play": 2, "Turn": 1, "Throw it": 1, "Wait": 1}, "numerals": {"5": 1}, "classifiers": {"individual": 1}}}, {"content": "6 Poor visual recognition (e.g., poor recognition of characteristics, size, colour, location, etc. of an object).", "word_count": 28, "label": "", "sentence_structure": "Subject鈥搗erb鈥揷omplement", "subjective_objective": {"answer": "Objective", "rationale": "Because \"visual recognition\" is a physical or medical objective state, which can be derived from the corresponding tests or measurements. And the patient's perception and subjective description are based on this objective state, so the entry to the questionnaire looks at the patient's subjective feelings and perceptions, not the objective facts themselves."}, "sentence_sentiment": {"answer": "Negative", "rationale": "Because the description of the purpose of the article refers to \"difficult ability to identify\", which is a negative expression."}, "language_style": {"answer": "Calm and direct", "rationale": "The entry is more calm and straightforward in language, with no excessive adoration or emotional colour. It clearly presents a fact or observation that the person under investigation has difficulty identifying a particular aspect of the visual identity, rather than trying to provoke emotional reactions or to induce the person under investigation to answer."}, "time_dimension": {"answer": "Present", "rationale": ""}, "sentence_type": "Affirmative declarative sentence", "pos": {"nouns": {"Visual": 1, "Identification": 2, "Capacity": 1, "Objects": 1, "Characteristics": 1, "Size": 1, "Colour": 1, "Location": 1}, "verbs": {"Bad": 1, "Like": 1, "to": 1}, "numerals": {"6": 1}}}, {"content": "7 No sexual smiles (i.e. no nods, no greeting, no smiles)", "word_count": 17, "label": "", "sentence_structure": "Subject鈥搗erb鈥揷omplement", "subjective_objective": {"answer": "Subjective", "rationale": "Because smiles are emotional expressions and relationships that require the active involvement and choice of individuals. If a person does not show a smile that is personal, it may be due to their character, mental state, or other personal reasons, so the entry needs to be viewed from the patient鈥檚 subjective perspective."}, "sentence_sentiment": {"answer": "Negative", "rationale": "Because the \"no-motion smile\" in the entry is negative."}, "language_style": {"answer": "Calm and direct", "rationale": "The entry shows directly that the subject of the survey is \"No-For-Fact Smile\", that it does not use excessive rhetorical techniques or emotional rendering, that it is more objective in tone and that it is a calm and direct language style."}, "time_dimension": {"answer": "Past", "rationale": "The words \"unconnective smile\" and \"not noding, greeting, smiling\" in the entry describe the acts that were tried in the past and therefore judge as the past."}, "sentence_type": "Affirmative declarative sentence", "pos": {"nouns": {"\"Smiling in relationships.\"": 1, "Nod your head": 1, "Hello": 1, "Smile": 1}, "verbs": {"Yes": 1, "and": 1, "Conduct": 1}, "adverbs": {"None": 1}, "numerals": {"7": 1}, "classifiers": {"individual": 1}}}, {"content": "8-words are used to reverse or confuse.", "word_count": 14, "label": "", "sentence_structure": "Subject鈥搗erb鈥揷omplement", "subjective_objective": {"answer": "Subjective", "rationale": "Because \"8-Adverted or Chaos\" is a human language, subjective behaviour."}, "sentence_sentiment": {"answer": "Neutral", "rationale": "Because the entry describes an objective fact, which is not emotional."}, "language_style": {"answer": "Tactful and warm", "rationale": "The entry uses a more euphemistic tone, asking if the other person has a \"8-nominal inverted or confused\" in his language. Such expression is more respectful and understanding of the person under investigation, and contributes to a good communication atmosphere."}, "time_dimension": {"answer": "Past", "rationale": "The \"E8\" in this entry is a reference to the past situation of the person tested, and therefore the temporal dimension is a reference to the past."}, "sentence_type": "Interrogative sentence", "pos": {"nouns": {"8 pronouns": 1, "Use": 1, "Turn upside down": 1, "Chaos": 1, "you": 1, "I": 2}, "verbs": {"Use": 1, "Turn upside down": 1, "Chaos": 1, "Disaggregat": 1}, "adjectives": {"Turn upside down": 1, "Chaos": 1}, "numerals": {"8": 1}, "pronouns": {"you": 1, "I": 2}}}, {"content": "Nine years of holding something.", "word_count": 10, "label": "", "sentence_structure": "Subject鈥搗erb鈥揷omplement", "subjective_objective": {"answer": "Subjective", "rationale": "This is a subjective question because it is a subjective feeling and behaviour that is always held by something for a long time."}, "sentence_sentiment": {"answer": "Negative", "rationale": "Because long-term possession of the same object can lead to physical fatigue, increased stress and other problems, it is a health hazard."}, "language_style": {"answer": "Calm and direct", "rationale": "The entry uses a simple and clear language and asks the interviewee whether he or she often holds an object for long periods of time, without excessive ado or emotional colour, and is more calm and direct in his or her language style."}, "time_dimension": {"answer": "Past", "rationale": "In it, \"long\" means continuity of conduct, while \"general\" emphasizes the frequency and extent of the conduct. The \"take something\" act may have occurred and continues, so it can be judged to contain elements of the past and the present."}, "sentence_type": "Affirmative declarative sentence", "pos": {"nouns": {"Time": 1, "Something": 1}, "verbs": {"Take it": 1}, "adjectives": {"Long": 1}, "adverbs": {"Total": 1}, "numerals": {"9": 1}}}, {"content": "Ten seems to be listening to people so that they suspect he has hearing problems.", "word_count": 20, "label": "", "sentence_structure": "Subject鈥揷opula鈥損redicative", "subjective_objective": {"answer": "Subjective", "rationale": "Because the description is an assessment of the patient's feelings and attitudes (i.e. \"suspect of hearing problems\"), it is not an objective fact or behavioural result."}, "sentence_sentiment": {"answer": "Negative", "rationale": "Because the expression \"suspect of hearing problems\" in the entry is negative and has a emotional tone of questioning and mistrust towards the person described."}, "language_style": {"answer": "Calm and direct", "rationale": "This is because the entry uses a more direct expression that clearly identifies the problems that the person being observed may have, and does not use euphemism or vague language."}, "time_dimension": {"answer": "Present", "rationale": "\"As if not listening\" and \"suspecting\" both point to the current situation, that is, the behaviour or attitude that the person being tested is showing. The entry therefore describes the current situation."}, "sentence_type": "Negative declarative sentence", "pos": {"nouns": {"10": 1, "person": 1, "Hearing": 2, "Problem": 1}, "verbs": {"in": 1, "Listen": 1, "Say it": 1, "Jean": 1, "Doubt": 1}, "adjectives": {"No, I'm not": 1, "Hearing": 1}, "adverbs": {"Seems like": 1, "to": 1}, "numerals": {"10": 1, "2": 1}, "classifiers": {"individual": 1}, "pronouns": {"he": 1}}}, {"content": "Eleven doesn't sound right.", "word_count": 9, "label": "", "sentence_structure": "Subject鈥揷opula鈥損redicative", "subjective_objective": {"answer": "Objective", "rationale": "Because speech is not sound and rhythm is a characteristic of the patient ' s behaviour and is considered from an objective perspective."}, "sentence_sentiment": {"answer": "Negative", "rationale": "Because the entry describes a linguistic impairment with a certain negative emotional colour."}, "language_style": {"answer": "Tactful and warm", "rationale": "The language style of the entry is more moderate, not too direct criticism or criticism, but rather a description of a phenomenon in a more understanding and inclusive manner. This expression is more acceptable and understandable and can reduce the negative emotional reactions that may arise."}, "time_dimension": {"answer": "Present", "rationale": "The entry now describes a current behavior or state, \"speaks out of tune, no rhythm\" which means that the person who was tested did not speak with the normal tone and rhythm, and that is an act that is now being carried out."}, "sentence_type": "Imperative sentence", "pos": {"nouns": {"11": 1, "Talk": 1, "Sound": 1, "Spacing": 1}, "verbs": {"- Yeah": 1, "None": 1}, "numerals": {"1": 1}, "classifiers": {"individual": 1}}}, {"content": "Twelve long swings of the body.", "word_count": 7, "label": "", "sentence_structure": "Subject鈥搗erb鈥揷omplement", "subjective_objective": {"answer": "Objective", "rationale": "Because the long swinging body is an objective description of the patient's physical movements."}, "sentence_sentiment": {"answer": "Negative", "rationale": "Because this behaviour of shaking bodies is often seen as a sign of lack of self-control and concentration, it has a negative emotional connotation."}, "language_style": {"answer": "Calm and direct", "rationale": "The language style of the questionnaire entry is calm and straightforward. It simply raises a single question, not using too many rhetorical or emotional colours, but presenting it to the interviewee in an objective and direct way. This way helps to ensure accuracy and clarity, allowing the interviewee to understand and answer the question clearly."}, "time_dimension": {"answer": "Present", "rationale": "The entry asks whether the testee ' s behaviour is constantly and permanently shaking his body and the temporal dimension is judged to be past or present."}, "sentence_type": "Imperative sentence", "pos": {"nouns": {"12": 1, "Time": 1, "Body": 1}, "verbs": {"Swing": 1, "Long time": 1}, "adverbs": {"Long": 1, "Time": 1}, "classifiers": {"individual": 1}}}, {"content": "13 To get something, but not where the body can reach (i.e., underestimating the distance between itself and the object).", "word_count": 34, "label": "", "sentence_structure": "Subject鈥搗erb鈥揷omplement", "subjective_objective": {"answer": "Objective", "rationale": "Because the title describes a specific act (to get something) and the actual result of the event, based on the distance of the object, it is an objective angle."}, "sentence_sentiment": {"answer": "Neutral", "rationale": "Because the entry describes an objective situation, there is no apparent emotional tendency or attitude."}, "language_style": {"answer": "Calm and direct", "rationale": "The entry presents a specific situation in a concise and clear language, with no excessive rhetorical and emotional rendering, with greater emphasis on practicality and accuracy in the transmission of information. At the same time, the language temperature and objectivity of the issue do not create a sense of stress or discomfort."}, "time_dimension": {"answer": "Present", "rationale": "The entry asks about the person ' s current behaviour or state, which involves distance estimation and mobility, and therefore the time dimension is now judged."}, "sentence_type": "Affirmative declarative sentence", "pos": {"nouns": {"Something": 1, "Local": 2, "Distance": 1}, "verbs": {"Yes": 2, "Take it": 1, "Go on": 1, "Achieved": 1, "estimate": 1, "Insufficient": 1}, "adjectives": {"Self": 1, "Objects": 1}, "numerals": {"13": 1}, "classifiers": {"individual": 1}}}, {"content": "14 Serious response to changes in the environment and daily life patterns", "word_count": 19, "label": "", "sentence_structure": "Subject鈥搗erb鈥搊bject", "subjective_objective": {"answer": "Subjective", "rationale": "Because \"serious reaction\" is a subjective feeling and an assessment."}, "sentence_sentiment": {"answer": "Negative", "rationale": "The \"serious reaction\" in the entry is an indication of negative sentiments about changes in the environment and in daily life patterns."}, "language_style": {"answer": "Calm and direct", "rationale": "This is a topic that is asked in a straightforward and clear manner, without too many rhetorical or emotional descriptions, and which is more calm and direct. This expression makes it easier for respondents to understand and answer questions, while also allowing them to obtain the information they need more accurately."}, "time_dimension": {"answer": "Present", "rationale": "The entry's \"Responding to\" indicates the current state or behaviour, and the keyword \"14\" may refer to a particular act or event that, at the time of its fourteenth occurrence, provoked the reaction of the testee, so the subject is looking at the current reaction of the testee to changes in the environment and daily life patterns."}, "sentence_type": "Imperative sentence", "pos": {"nouns": {"to": 1, "Environment": 1, "The law of daily life": 1, "Response": 1}, "verbs": {"Production": 1, "Fan": 1, "Change": 1}, "adjectives": {"Serious": 1}, "numerals": {"14": 1}}}, {"content": "When he was with the others, he called his name, and he didn't respond.", "word_count": 20, "label": "", "sentence_structure": "Subject鈥搗erb鈥揷omplement", "subjective_objective": {"answer": "Objective", "rationale": "Because calling for and responding to others' names is an objective act and result, independent of the subjective will of the individual. Even if the patient himself may have ignored or not noticed, it still occurs and can be observed and recorded. Thus, \"15 When he is with others, calling his name, he does not react\" is objective."}, "sentence_sentiment": {"answer": "Negative", "rationale": "Because the \"no reaction\" act in the entry is negative."}, "language_style": {"answer": "Calm and direct", "rationale": "The entry describes the behaviour of the person under investigation in a social setting in an objective manner, without excessive rhetorical or emotional rendering, and with a more calm and straightforward language style."}, "time_dimension": {"answer": "Present", "rationale": ""}, "sentence_type": "Negative declarative sentence", "pos": {"nouns": {"Other": 1, "person": 2, "Call": 1, "he": 2, "Name": 1, "Response": 1}, "verbs": {"and": 1, "in": 1, "Together": 1, "Time": 1, "No, I'm not": 1, "have": 1, "Response": 1}, "adverbs": {"When": 1, "Other": 1, "in": 1, "Together": 1, "Time": 1, "No, I'm not": 1}, "numerals": {"15": 1}, "classifiers": {"individual": 1}, "pronouns": {"he": 2}}}, {"content": "16 often do forward movement, rotation, toe-toe walking, finger-squeeze, etc.", "word_count": 21, "label": "", "sentence_structure": "Subject鈥搗erb鈥揷omplement", "subjective_objective": {"answer": "Subjective", "rationale": "Because these actions are usually the subjective behaviour of the patient."}, "sentence_sentiment": {"answer": "Neutral", "rationale": "Because the entry describes some movement, it does not involve positive or negative emotional colours."}, "language_style": {"answer": "Calm and direct", "rationale": "The entry directly describes some of the actions that the respondents may have shown, which are not overly accommodative or emotional, and which are more objective and calm."}, "time_dimension": {"answer": "Present", "rationale": "The entry now describes the current behaviour of the subject, using the term \"often\" to emphasize that such behaviour is long-standing or frequent. At the same time, \"acts like front-slashing, rotation, toe-scratching, finger-screeching, etc.\" are also described in relation to the specific behaviour of the time, and therefore judged to be the present."}, "sentence_type": "Affirmative declarative sentence", "pos": {"nouns": {"Forward": 1, "Rotate": 1, "- Go to the right foot": 1, "Finger": 1, "Squeeze": 1, "Light": 1, "Actions": 1}, "verbs": {"Make": 1}, "adverbs": {"Regular": 1}, "numerals": {"16": 1}}}, {"content": "17 doesn't respond to the facial or emotional expressions of others.", "word_count": 16, "label": "", "sentence_structure": "Subject鈥搗erb鈥揷omplement", "subjective_objective": {"answer": "Subjective", "rationale": "Because \"no response to other people's facial expressions or feelings\" is a description of a personal subjective feeling."}, "sentence_sentiment": {"answer": "Negative", "rationale": "Because the behaviour described in the entry as \"unresponsive to the face or feelings of others\" is negative, it shows that the person lacks social skills and emotional resonance."}, "language_style": {"answer": "Calm and direct", "rationale": "The entry is in a simple and clear language, and the interviewee is directly asked about the degree of reaction to the face or feelings of others, which is not overly adour or emotional, and is therefore of a calm and straightforward language style."}, "time_dimension": {"answer": "Past", "rationale": "According to the sentence entered, \"17\" is the number of times, and the latter \"no response to the face or feelings of others\" describes the behaviour and attitude of the person who was tested in the past. The entry therefore asks about the behaviour and attitude of the person who was tested in the past."}, "sentence_type": "Negative declarative sentence", "pos": {"nouns": {"17": 1, "person": 1, "Face expression": 1, "Emotion": 1}, "verbs": {"have": 1, "Nope": 1, "Response": 1}, "numerals": {"to": 1}, "classifiers": {"individual": 1}}}, {"content": "\"Yes\" or \"I\" are rarely used when you speak.", "word_count": 11, "label": "", "sentence_structure": "Subject鈥搗erb鈥揷omplement", "subjective_objective": {"answer": "Objective", "rationale": "Because it is an objective fact to use words like \"yes\" or \"me\" when you speak, and whether they are less subjective to the patient."}, "sentence_sentiment": {"answer": "Negative", "rationale": "Because the behaviour of this entry is negative, i.e. too modest and avoids self-centre, it may be interpreted as a lack of self-confidence or unwillingness to assume responsibility."}, "language_style": {"answer": "Tactful and warm", "rationale": "The entry is more personal and interactive when it is spoken in a gentle, inviting manner, rather than directly speaking or ordering. At the same time, the content of the entry appears to be leading the respondents to reflect on their language habits in their daily communication, and the way they are asked is more euphemism, without too much judgement or criticism of the language used by the interviewees."}, "time_dimension": {"answer": "Present", "rationale": "So the answer is \"now\"."}, "sentence_type": "Affirmative declarative sentence", "pos": {"nouns": {"Word": 3, "be": 1, "I": 1}, "verbs": {"Say it": 1, "Use": 1}, "adverbs": {"Very few": 1}, "numerals": {"18": 1}, "classifiers": {"individual": 1}, "pronouns": {"Wait": 1}}}, {"content": "There's one aspect of special abilities that seem to be incompatible with low intellectual capacity.", "word_count": 21, "label": "", "sentence_structure": "Subject鈥揷opula鈥損redicative", "subjective_objective": {"answer": "Subjective", "rationale": "Because the descriptions of \"special abilities\" and \"intellectual incompetence\" are based on subjective observations and feelings of patients."}, "sentence_sentiment": {"answer": "Neutral", "rationale": "Because the entry merely stated a fact, it did not express any emotional colour."}, "language_style": {"answer": "Calm and direct", "rationale": "The entry asks the interviewees in a direct and clear manner whether they have a particular ability to express themselves in a simple and clear manner, without any rhetorical or emotional rendering, and in a more calm and straightforward way."}, "time_dimension": {"answer": "Present", "rationale": "The entry used to describe the testee's past, and the keywords were \"19 years old\" and \"with a particular ability that does not seem to correspond to a mental impairment.\" This information points to what happened in the past. So the time dimension of the entry is the past."}, "sentence_type": "Affirmative declarative sentence", "pos": {"nouns": {"On the one hand": 1, "Special capabilities": 1, "Intellectual": 2, "Low": 1}, "verbs": {"have": 1, "Like": 1, "and": 1, "Match": 1}, "adjectives": {"Low": 1, "Match": 1}, "adverbs": {"Down": 1}, "numerals": {"19": 1}, "pronouns": {"In a certain way": 1}}}, {"content": "20 cannot execute simple instructions containing phrases (e.g., putting the ball on or in a box)", "word_count": 30, "label": "", "sentence_structure": "Subject鈥損redicate", "subjective_objective": {"answer": "Objective", "rationale": "Because \"the execution of simple instructions with words\" is the patient's daily behaviour and is an objective fact."}, "sentence_sentiment": {"answer": "Negative", "rationale": "Because the description \"not to execute a simple instruction containing a phrase\" in the entry is negative and indicates that the person tested has a problem."}, "language_style": {"answer": "Calm and direct", "rationale": "The language style of the entry is straightforward and calm, as it clearly describes the specific tasks to be performed by the person under examination, without any rhetorical or emotional rendering."}, "time_dimension": {"answer": "Present", "rationale": "Key words such as \"20\", \"not able to implement\", \"simple\", \"directives containing phrases\" indicate that the entry is about the ability of the testee to understand and implement simple instructions, and therefore the judgement is now."}, "sentence_type": "Negative declarative sentence", "pos": {"nouns": {"20": 1, "Implementation": 1, "Simple": 1, "Ham": 1, "have": 1, "Interpret": 1, "Statement": 1, "Command": 1, "Ball": 1, "Box": 2}, "verbs": {"- Put it on": 2, "Fire": 2}, "adjectives": {"Implementation": 1, "Simple": 1}, "numerals": {"20": 1}, "classifiers": {"individual": 1}}}, {"content": "Sometimes it doesn't surprise you to hear a big sound.", "word_count": 25, "label": "", "sentence_structure": "Subject鈥搗erb鈥揷omplement", "subjective_objective": {"answer": "Objective", "rationale": "Because reactions to loud voices are an observable result of behaviour that is not influenced by an individual鈥檚 subjective sense. While this may lead to the misperception of the patient鈥檚 hearing, it remains an objective fact that can be viewed from the perspective of behaviour."}, "sentence_sentiment": {"answer": "Negative", "rationale": "Because the action of \"no surprise reaction to a big voice\" in the entry is negative."}, "language_style": {"answer": "Tactful and warm", "rationale": "The entry is more personal and interactive when it is spoken in a gentle, inquisitive manner rather than directly speaking or ordering."}, "time_dimension": {"answer": "Past", "rationale": ""}, "sentence_type": "Affirmative declarative sentence", "pos": {"nouns": {"21": 1, "Sound": 1, "Response": 1, "Deaf": 1}, "verbs": {"鈾?Made out of 鈾?: 1, "Jean": 1, "Yes": 1}, "adjectives": {"Large": 1, "Surprise": 1}, "adverbs": {"Sometimes": 1}, "numerals": {"21": 1, "very": 1}, "pronouns": {"he": 1}}}, {"content": "Twenty-two beats the shit out of them.", "word_count": 5, "label": "", "sentence_structure": "Subject鈥搗erb鈥揷omplement", "subjective_objective": {"answer": "Objective", "rationale": "Because the batterer is the patient ' s behaviour and is a description of objective facts."}, "sentence_sentiment": {"answer": "Neutral", "rationale": "Because the entry does not have any emotional color."}, "language_style": {"answer": "Calm and direct", "rationale": "The entry directly asked the interviewees whether they were often spanking their hands, the language style was more straightforward, the tone was less balanced or emotional and the tone was less objective than the excessive use of rhetorical techniques."}, "time_dimension": {"answer": "Past", "rationale": ""}, "sentence_type": "Imperative sentence", "pos": {"verbs": {"Regular": 1, "Beating": 1, "Hands": 1}, "numerals": {"22": 1}}}, {"content": "23, or a little bit of a little.", "word_count": 11, "label": "", "sentence_structure": "Subject鈥搗erb鈥揷omplement", "subjective_objective": {"answer": "Subjective", "rationale": "Because \"smuggling\" is an emotional experience, a subjective feeling and attitude of the patient."}, "sentence_sentiment": {"answer": "Negative", "rationale": "Because \"crazy\" is usually considered a negative emotional reaction, the emotional color of this purpose is negative."}, "language_style": {"answer": "Calm and direct", "rationale": "The entry directly asked whether the respondents were prone to a temperamentality, without too many rhetorical or emotional renderings, and with a more objective tone."}, "time_dimension": {"answer": "Past", "rationale": "The entry asks if the person tested has had a constant temper for some time and therefore judged it to be the past."}, "sentence_type": "Imperative sentence", "pos": {"nouns": {"- Grumpy": 1, "Points": 1}, "verbs": {"Let's go": 2, "- You're losing your temper": 1}, "adverbs": {"Large": 1, "Regular": 1}, "numerals": {"23": 1, "individual": 1}}}, {"content": "24 Actively avoiding eye contact", "word_count": 12, "label": "", "sentence_structure": "Subject鈥搗erb鈥揷omplement", "subjective_objective": {"answer": "Subjective", "rationale": "Because \"scramble\" and \"eye contact\" are psychological feelings and behaviour, subjective."}, "sentence_sentiment": {"answer": "Negative", "rationale": "Because the act of \"actively avoiding\" in the entry is negative."}, "language_style": {"answer": "Calm and direct", "rationale": "The entry uses a simple and clear language style, without any rhetorical or emotional rendering, which is more objective and cold and therefore a straightforward style of calmness."}, "time_dimension": {"answer": "Past", "rationale": "The words \"excusing\" and \"excusing\" in the entry are all expressions of past actions or states, and \"24\" also indicates a pattern or custom, so it can be judged to be about the testee's past behaviour."}, "sentence_type": "Interrogative sentence", "pos": {"nouns": {"24": 1, "Initiatives": 1, "Avoidance": 1, "View": 1, "Exposure": 1}}}, {"content": "25 refuse to touch or embrace others", "word_count": 10, "label": "", "sentence_structure": "Subject鈥搗erb鈥揷omplement", "subjective_objective": {"answer": "Objective", "rationale": "It is an objective fact that the denial of contact or hugging is the manifestation of the patient ' s behaviour."}, "sentence_sentiment": {"answer": "Negative", "rationale": "Because the \"denial of contact or embrace\" in the entry is negative."}, "language_style": {"answer": "Calm and direct", "rationale": "The entry is a straightforward query about whether respondents often refuse to contact or embrace others, the language style is more direct and clear, there is no excessive aesthetic or emotional colour, and there is a greater focus on obtaining objective factual information."}, "time_dimension": {"answer": "Present", "rationale": "The main phrase \"25 Refusal of others to contact or embrace\" implies that it is a description of a group of acts, while the verb \"No\" indicates that the act is a current act. The entry therefore asks about the person being tested for the present act or attitude."}, "sentence_type": "Interrogative sentence", "pos": {"nouns": {"25": 1, "others": 1, "Exposure": 1, "Hug": 1}, "verbs": {"Reject": 1}, "numerals": {"25": 1}}}, {"content": "Sometimes it doesn't react to painful irritation, like a fall, a cut or a injection.", "word_count": 22, "label": "", "sentence_structure": "Subject鈥揷opula鈥損redicative", "subjective_objective": {"answer": "Objective", "rationale": "As it is a biological reaction to external irritation, it is not controlled by personal consciousness and is therefore objectively viewed."}, "sentence_sentiment": {"answer": "Negative", "rationale": "Because \"no response to painful irritation such as a fall, cut or injection\" is a negative physical reaction."}, "language_style": {"answer": "Calm and direct", "rationale": "The entry directly describes the fact that some people are not responding to painful irritations, are not too emotional or rhetorical and are more calm and straightforward."}, "time_dimension": {"answer": "Present", "rationale": "In the past, key words such as \"sometimes\", \"wrecking\", \"cut\" and \"injection\" appeared in the entry, all of which related to past experiences and experiences. Thus, the temporal dimension of the purpose of the article is to judge the past."}, "sentence_type": "Imperative sentence", "pos": {"nouns": {"26": 1, "Sometimes": 1, "It's painful": 1, "Exciting": 1, "Broken": 1, "Cut": 1, "Injection": 1, "Response": 1}, "verbs": {"Cause": 1, "not": 1}, "adjectives": {"Pain": 1}, "adverbs": {"very": 1, "Sometimes": 1}, "numerals": {"26": 1}}}, {"content": "27 is very rigid and difficult to hold.", "word_count": 11, "label": "", "sentence_structure": "Subject鈥搗erb鈥揷omplement", "subjective_objective": {"answer": "Subjective", "rationale": "Because \"physical behaviour is rigid and difficult to hold\" is a subjective feeling and experience of the patient, not an objective fact. This feeling is the result of the patient鈥檚 physical and psychological state, and is therefore subjective."}, "sentence_sentiment": {"answer": "Negative", "rationale": "Because the description \"physically rigid and difficult to hold\" is negative, suggesting that there may be physical health problems or insufficient motor capacity."}, "language_style": {"answer": "Tactful and warm", "rationale": "The entry uses a soft, invitation-style tone to ask questions, is less direct or command-like, is more apt and more acceptable. At the same time, the question is not too much focused on personal problems or wrongdoing, but is presented in an open and understanding manner, which facilitates the respondents to be more willing to participate and respond."}, "time_dimension": {"answer": "Present", "rationale": "The keyword \"stight physical behaviour, hard to hold\" indicates the condition of the person who was tested when filling out the questionnaire, so the temporal dimension is now judged."}, "sentence_type": "Negative declarative sentence", "pos": {"nouns": {"Body": 1, "Hug": 1}, "verbs": {"Performance": 1, "Stunning": 1, "Hold on": 1}, "adjectives": {"very": 2, "Hard": 1}, "numerals": {"27": 1}}}, {"content": "When I looked at him, I felt his muscles loose.", "word_count": 22, "label": "", "sentence_structure": "Subject鈥搗erb鈥揷omplement", "subjective_objective": {"answer": "Objective", "rationale": "The muscle laxity is a physical characteristic that can be observed, not a subjective feeling or attitude of the patient."}, "sentence_sentiment": {"answer": "Negative", "rationale": "Because the description of \"muscle loose\" in the entry is usually considered a negative feature."}, "language_style": {"answer": "Tactful and warm", "rationale": "The entry is more adept and interactive when it is spoken in a gentle and inviting manner, rather than by direct statements or orders."}, "time_dimension": {"answer": "Present", "rationale": "The word \"kick\" means the action being taken, and \"felt\" is now being carried out, pointing to the situation at this point, so it can be judged that the entry is about the person who is being tested."}, "sentence_type": "Affirmative declarative sentence", "pos": {"nouns": {"Hug": 1, "Muscles": 1, "person": 1, "The man who held him": 1}, "verbs": {"Time": 1, "Look": 1, "I feel": 1, "Close": 1, "Hug": 2}, "adjectives": {"Relax": 1, "not": 1}, "numerals": {"28": 1}, "pronouns": {"he": 2}}}, {"content": "29 expresses what is desired in position and gesture (not in language)", "word_count": 24, "label": "", "sentence_structure": "Subject鈥搗erb鈥搊bject", "subjective_objective": {"answer": "Subjective", "rationale": "Because this questionnaire entry describes the patient ' s tendency to express his needs using gestures and gestures, such behaviour and ideas are subjective."}, "sentence_sentiment": {"answer": "Neutral", "rationale": "Because the entry does not have any emotional color."}, "language_style": {"answer": "Calm and direct", "rationale": ""}, "time_dimension": {"answer": "Past", "rationale": "The entry describes the past customary behaviour of the testee, i.e. the expression of demand through gestures and gestures rather than reliance on words."}, "sentence_type": "Affirmative declarative sentence", "pos": {"nouns": {"29": 1, "Position": 1, "Gestures": 1, "Something": 1}, "verbs": {"Organisation": 1, "Preferative": 1, "Got it": 1}, "adverbs": {"not": 1}, "numerals": {"Here": 1}, "classifiers": {"individual": 1}}}, {"content": "30 feet walk.", "word_count": 6, "label": "", "sentence_structure": "Subject鈥搗erb鈥揷omplement", "subjective_objective": {"answer": "Objective", "rationale": "Because \"frequent foot walking\" describes an objective behaviour that does not require subjective evaluation or interpretation by the patient."}, "sentence_sentiment": {"answer": "Negative", "rationale": "Because toes walk can cause bodily harm, the behaviour is negative."}, "language_style": {"answer": "Calm and direct", "rationale": "The entry directly asked whether respondents often used the practice of walking to the point of their daily life, without too many rhetorical or emotional colours, and with a more direct and objective language style."}, "time_dimension": {"answer": "Present", "rationale": "According to the description of \"commonly used\" in the sentence, \"frequently to walk\" means a regular customary act that occurs at or before the moment of the speech, so it can be judged that the item describes the person being tested in his present behaviour or state."}, "sentence_type": "Imperative sentence", "pos": {"nouns": {"Toes": 1, "Walk": 1}, "verbs": {"Common": 1}, "numerals": {"30": 1}}}, {"content": "31 to hurt people by biting, hitting, kicking, etc.", "word_count": 14, "label": "", "sentence_structure": "Subject鈥搗erb鈥搊bject", "subjective_objective": {"answer": "Objective", "rationale": "Because \"harming someone with a bite, bump, kick\" is an act that the patient may exhibit, and whether or not it occurs does not depend on the patient鈥檚 subjective will. The questionnaire entry is therefore viewed from an objective perspective."}, "sentence_sentiment": {"answer": "Negative", "rationale": "Because \"harming someone\" in the entry is negative."}, "language_style": {"answer": "Calm and direct", "rationale": "The entry is a direct and explicit expression of the will not to allow the person under investigation to use the acts of bite, hit, kick, etc. to harm others. This expression is relatively strong, with little emotional recitation or rhetorical techniques, and is more consistent with objective descriptions."}, "time_dimension": {"answer": "Present", "rationale": "The entry describes current behaviour or habits, \"by biting, hitting, kicking, etc.\" which indicates that this is an ongoing move, so it is the current state of affairs."}, "sentence_type": "Affirmative declarative sentence", "pos": {"nouns": {"Behaviour": 1, "person": 2, "Bite": 1, "Hit him": 1, "Kick him": 1, "Hurt": 1}, "verbs": {"Use": 1, "Hurt": 1, "Wait": 1}, "numerals": {"31": 1}}}, {"content": "32 repeats the short sentences.", "word_count": 7, "label": "", "sentence_structure": "Subject鈥搗erb鈥揷omplement", "subjective_objective": {"answer": "Subjective", "rationale": "Because \"repeat\" is an act of the patient's own initiative, reflecting his psychological state and internal needs, it is a subjective examination."}, "sentence_sentiment": {"answer": "Neutral", "rationale": "Because the entry does not express any positive or negative feelings, attitudes, behaviour, purpose or quality, it merely describes a fact."}, "language_style": {"answer": "Tactful and warm", "rationale": "The entries use terms such as \"continuous\" and \"short sentences\" that are more gentle, euphemistic and give people a sense of kindness, rather than being too direct or tough to express their issues. The language style of the entries can therefore be considered as being soft."}, "time_dimension": {"answer": "Future", "rationale": "The entry contains visions and expectations for the future, and the keywords are \"repeated\" and \"short sentences\" which point to situations that have not yet occurred and that may arise in the future."}, "sentence_type": "Imperative sentence", "pos": {"nouns": {"32": 1, "Short sentences": 1}, "verbs": {"Repeat": 1}, "adverbs": {"Constantly": 1}, "numerals": {"32": 1}}}, {"content": "33 games without imitating other children", "word_count": 10, "label": "", "sentence_structure": "Subject鈥搗erb鈥揷omplement", "subjective_objective": {"answer": "Subjective", "rationale": "Because \"Imitation of other children\" is a behavior, and whether and to what extent it occurs is an objective fact that can be observed and measured. And the title is called \"No imitation,\" which is a subjective attitude or view. So, \"33 games without imitation of other children\" is viewed from the patient's subjective point of view."}, "sentence_sentiment": {"answer": "Neutral", "rationale": "Because the entry does not have any emotional color."}, "language_style": {"answer": "Calm and direct", "rationale": "The language style of the entry is more calm and straightforward, without too much embellishment or emotional rendering, but simply expresses a requirement or regulation, without any euphemism or hint. This expression is more understandable and acceptable, and it is easier to enforce and regulate."}, "time_dimension": {"answer": "Present", "rationale": "The time indicator \"no imitation in game\" in this entry indicates that it is the behaviour of the testee in playing games, and that the act is now being performed and the dimension of time is now judged."}, "sentence_type": "Negative declarative sentence", "pos": {"nouns": {"Game": 1, "Children": 1}, "verbs": {"Do not imitate": 1}, "numerals": {"33": 1}}}, {"content": "When the light is radiant, it's often blinking.", "word_count": 15, "label": "", "sentence_structure": "Subject鈥搗erb鈥揷omplement", "subjective_objective": {"answer": "Objective", "rationale": "Because \"when a strong light is sighted\" is a physical reaction of a human being, without taking into account the individual's will and feelings, and is therefore objective."}, "sentence_sentiment": {"answer": "Negative", "rationale": "Because \"no blinking\" in the entries could cause visual impairment, it's negative."}, "language_style": {"answer": "Calm and direct", "rationale": "The entry uses a direct language style, which describes a phenomenon succinctly and without excessive rhetorical or emotional colours. This language style is objective, neutral and accurate in communicating information."}, "time_dimension": {"answer": "Present", "rationale": ""}, "sentence_type": "Negative declarative sentence", "pos": {"nouns": {"Strong Light": 1, "Eyes": 2, "Blink": 1}, "verbs": {"When": 1, "Exposure": 1, "Regular": 1, "not": 1}, "adverbs": {"Often": 1}, "numerals": {"34": 1}}}, {"content": "35 self-inflicted with head bumps, bites, etc.", "word_count": 10, "label": "", "sentence_structure": "Subject鈥搗erb鈥揷omplement", "subjective_objective": {"answer": "Objective", "rationale": "Self-inflicted injuries due to head bumps, bites, etc. are an objective fact of the patient."}, "sentence_sentiment": {"answer": "Negative", "rationale": "Because self-inflicted injuries like head bumps and bites are negative in the entries."}, "language_style": {"answer": "Calm and direct", "rationale": "The language style of the entry is more straightforward, concise, without too many rhetorical or emotional renderings, and more factually based questioning and description."}, "time_dimension": {"answer": "Present", "rationale": "The entry describes the person's current conduct, with the keyword \"in the form of\" and \"such as\" , which indicates that the act is taking place or is taking place on a regular basis. Thus, the temporal dimension of the purpose of the article is now judged."}, "sentence_type": "Affirmative declarative sentence", "pos": {"nouns": {"Behaviour": 1, "Self-inflicted": 1}, "verbs": {"Head bump": 1, "Bite your hand": 1, "Here": 1}, "numerals": {"35": 1}}}, {"content": "36 can't wait for anything.", "word_count": 21, "label": "", "sentence_structure": "Subject鈥搗erb鈥揷omplement", "subjective_objective": {"answer": "Subjective", "rationale": "Because what you want cannot wait for is a patient's personal feeling and desire, it's a subjective description."}, "sentence_sentiment": {"answer": "Positive", "rationale": "Because the behavior in the entry \"wants something to wait\" expresses a positive attitude, which is to pursue immediate satisfaction and action."}, "language_style": {"answer": "Calm and direct", "rationale": "The entry expresses the problem in a direct and clear language, without too many rhetorical or emotional colours, and is more calm and objective."}, "time_dimension": {"answer": "Present", "rationale": ""}, "sentence_type": "Imperative sentence", "pos": {"nouns": {"Something": 2, "Wait": 1, "Yes": 2}, "verbs": {"Yes": 4, "Got it": 1, "Wait": 1}, "adverbs": {"No, I can't": 1}, "numerals": {"36": 1}}}, {"content": "No names of more than 5 objects could be identified", "word_count": 12, "label": "", "sentence_structure": "Subject鈥搗erb鈥揷omplement", "subjective_objective": {"answer": "Subjective", "rationale": "Since the subject is to ask the testee about his cognitive abilities (can he name more than five objects) and requires reflection and memory, this is a subjective examination."}, "sentence_sentiment": {"answer": "Negative", "rationale": "Because the act of \"not identifying the name of more than five objects\" in the entry is negative and indicates a perceived impairment in the capacity of the person under investigation."}, "language_style": {"answer": "Calm and direct", "rationale": "The language style of the entry is more calm and straightforward, it clearly raises a specific problem and does not use any rhetorical or emotional expression."}, "time_dimension": {"answer": "Present", "rationale": "The keyword is \"not to point out\", which is an act of ability or skill, which occurs in the present state and is therefore judged to be now."}, "sentence_type": "Negative declarative sentence", "pos": {"nouns": {"37": 1, "5": 1, "Objects": 1, "Name": 1}, "verbs": {"Note": 1}, "numerals": {"1": 2, "individual": 1}}}, {"content": "38 can't develop any friendship.", "word_count": 19, "label": "", "sentence_structure": "Subject鈥搗erb鈥揷omplement", "subjective_objective": {"answer": "Subjective", "rationale": "Because \"not having friends with kids\" is the patient's subjective idea."}, "sentence_sentiment": {"answer": "Negative", "rationale": "Because the act of \"not developing any friendship\" is negative."}, "language_style": {"answer": "Calm and direct", "rationale": "The language style of the entry is of a calm and direct type. It expresses the problem in a concise and clear manner, without too many rhetorical or emotional terms. This style is usually more appropriate in situations such as questionnaires that require objective and accurate information collection."}, "time_dimension": {"answer": "Past", "rationale": ""}, "sentence_type": "Imperative sentence", "pos": {"nouns": {"Friendship": 1, "Kids": 1, "friends": 1}, "verbs": {"Development": 1, "Travel": 1, "Hand it over": 1}, "adjectives": {"Any": 1}, "adverbs": {"No, I can't": 1, "Yes": 1, "not": 1}, "numerals": {"38": 1}, "pronouns": {"锛?: 1, "锛?: 1}}}, {"content": "When 39 sounds, they're always covered in their ears.", "word_count": 14, "label": "", "sentence_structure": "Subject鈥搗erb鈥搊bject", "subjective_objective": {"answer": "Subjective", "rationale": "Because it's an expression of self-perception and subjective attitudes, reflecting the patient's feelings for a certain sound.\""}, "sentence_sentiment": {"answer": "Negative", "rationale": "Because the behavior in the entry \"covered the ears\" expresses a negative attitude, i.e. displeased or unbearable with the noise."}, "language_style": {"answer": "Tactful and warm", "rationale": "The entry is more adept and interactive when it is spoken in a gentle and inviting manner, rather than by direct statements or orders."}, "time_dimension": {"answer": "Present", "rationale": "The entry describes the normal reaction of the person being tested when faced with many voices - covering his ears - a customary, recurrent act, which is the time dimension of the test, and therefore the present."}, "sentence_type": "Affirmative declarative sentence", "pos": {"nouns": {"Many": 1, "Sound": 1}, "verbs": {"Cover it": 1, "Ears": 1}, "adjectives": {"Often": 1, "Time": 1}, "adverbs": {"39": 1, "have": 1}}}, {"content": "40 Frequent rotation of impact objects", "word_count": 8, "label": "", "sentence_structure": "Subject鈥搗erb鈥揷omplement", "subjective_objective": {"answer": "Subjective", "rationale": "Because \"resulting objects\" are a human movement habit and a tendency to be subject to subjective individual feelings and experiences."}, "sentence_sentiment": {"answer": "Neutral", "rationale": "Because the entry describes an act without a clear emotional tendency."}, "language_style": {"answer": "Calm and direct", "rationale": "The language style of the entry is a straightforward type of calm. It clearly presents a behaviour or phenomenon, and does not have too many emotional or rhetorical techniques, but simply explicit statements and inquiries."}, "time_dimension": {"answer": "Past", "rationale": "The temporal dimension of the purpose of the article should therefore be judged as \"now\"."}, "sentence_type": "Affirmative declarative sentence", "pos": {"nouns": {"Objects": 1}, "verbs": {"Rotate": 1, "Collision": 1}, "adjectives": {"Regular": 1, "40": 1}}}, {"content": "41 has difficulty in training to urinate.", "word_count": 18, "label": "", "sentence_structure": "Subject鈥搗erb鈥揷omplement", "subjective_objective": {"answer": "Objective", "rationale": "The objective fact that patients experience difficulties in training for urinals."}, "sentence_sentiment": {"answer": "Negative", "rationale": "Because the expression \"the difficulty of training to excrement\" in the entry is negative."}, "language_style": {"answer": "Calm and direct", "rationale": "The language style of the entry is calm and straightforward, and it clearly describes the difficulty of training the subject in urination, without using too much qualifying or emotional expression."}, "time_dimension": {"answer": "Past", "rationale": "The keyword \"difficulties in training urine\" indicates that it is a description of past experience or behaviour, and the temporal dimension of the purpose of the article is therefore judged to be past."}, "sentence_type": "Negative declarative sentence", "pos": {"nouns": {"Training": 1, "Shit": 2, "Difficulties": 1, "Control": 1}, "verbs": {"have": 1, "No, I won't": 1, "In terms of": 1}, "numerals": {"41": 1}}}, {"content": "Only five requests per day in 42 days.", "word_count": 12, "label": "", "sentence_structure": "Subject鈥搗erb鈥揷omplement", "subjective_objective": {"answer": "Objective", "rationale": "The objective facts and rules are the same."}, "sentence_sentiment": {"answer": "Negative", "rationale": "Because the purpose of the article is expressed in a restrictive and depressing way, it may be felt that its needs are ignored or rejected."}, "language_style": {"answer": "Calm and direct", "rationale": "The entry is more concise, plain, and not over-modified in terms of words and emotional colours, and more in the way of sending factual messages, which is a calm and direct language style."}, "time_dimension": {"answer": "Past", "rationale": ""}, "sentence_type": "Imperative sentence", "pos": {"nouns": {"Oh, my God": 1, "Request": 2}, "verbs": {"Present": 1, "Yes": 1, "Only": 1}, "adjectives": {"within": 1, "Five": 1}, "numerals": {"42": 1, "5": 1}}}, {"content": "43 is often frightened or very anxious.", "word_count": 13, "label": "", "sentence_structure": "Subject鈥揷opula鈥損redicative", "subjective_objective": {"answer": "Subjective", "rationale": "Because \"frightened\" and \"anxiety\" are patients' subjective feelings and emotional experiences."}, "sentence_sentiment": {"answer": "Negative", "rationale": "Because \"scared\" and \"very anxious\" are negative emotional experiences in the entries."}, "language_style": {"answer": "Tactful and warm", "rationale": "The purpose of the article is to be judged in a more moderate manner, using the terms \"frequent\" and \"very\" and expressing a sense of concern and concern rather than directly accusing or criticizing the other; and the entry uses the first person's method of asking questions, \"Please analyse below\", which is more acrimony and polite and more acceptable to the person under investigation."}, "time_dimension": {"answer": "Present", "rationale": ""}, "sentence_type": "Affirmative declarative sentence", "pos": {"nouns": {"Surprise": 1, "Anxiety": 2, "Unsettled": 1}, "verbs": {"Copy that": 1}, "adverbs": {"Regular": 1, "Very": 1}, "numerals": {"43": 1}}}, {"content": "44 slashed, closed, frowned under normal light.", "word_count": 12, "label": "", "sentence_structure": "Subject鈥搗erb鈥揷omplement", "subjective_objective": {"answer": "Objective", "rationale": "Because these actions are observed acts that are not part of the patient ' s inner thoughts or feelings."}, "sentence_sentiment": {"answer": "Negative", "rationale": "Because \"slash\" and \"silent\" and \"frown\" are negative behaviours, they usually express uncomfortable or unsatisfactory emotions."}, "language_style": {"answer": "Calm and direct", "rationale": "The language style of the entry is relatively simple, and it is not over-modified or emotionally coloured, and is used to give respondents answers in a direct question."}, "time_dimension": {"answer": "Present", "rationale": "The term \"in normal light\" and \"slash, close eyes, frown\" describe the actions or state of the person being tested, so the time dimension of the entry is now."}, "sentence_type": "Imperative sentence", "pos": {"nouns": {"Light": 1, "Slash your eyes": 1, "Close your eyes": 1, "Wrinkles": 1}, "verbs": {"in": 1, "Slash": 1, "Close": 1, "Wrinkles": 1}, "adjectives": {"Normal": 1}, "numerals": {"44": 1}}}, {"content": "45 is not often helped, and he doesn't wear himself.", "word_count": 18, "label": "", "sentence_structure": "Subject鈥搗erb鈥揷omplement", "subjective_objective": {"answer": "Objective", "rationale": "Since it is an objective ability and fact to dress oneself, the entry to the questionnaire is an objective observation and evaluation of whether the patient can wear clothes without being assisted."}, "sentence_sentiment": {"answer": "Negative", "rationale": "Because the behavior in the entry \"does not dress yourself\" is negative."}, "language_style": {"answer": "Calm and direct", "rationale": "This shows that a person needs to rely on others in his or her daily life and cannot perform some basic life skills independently. Thus, the language style of the article can be described as \"directly practical\"."}, "time_dimension": {"answer": "Present", "rationale": "This is a description of the present."}, "sentence_type": "Affirmative declarative sentence", "pos": {"nouns": {"45": 1, "person": 2, "Word": 1, "self": 1, "Clothes": 1}, "verbs": {"be": 1, "By": 1, "Help": 1, "Here": 2, "Put it on": 1}, "adjectives": {"not": 2, "Regular": 1}, "numerals": {"45": 1}, "pronouns": {"self": 1}}}, {"content": "46 repeats voices or words over and over again.", "word_count": 11, "label": "", "sentence_structure": "Subject鈥搗erb鈥揷omplement", "subjective_objective": {"answer": "Subjective", "rationale": "Because this is a subjective examination of patients' inner feelings and behaviour."}, "sentence_sentiment": {"answer": "Negative", "rationale": "Because \"some voices or words over and over again\" may be the expression of coercion or anxiety, with some negative emotional connotations."}, "language_style": {"answer": "Calm and direct", "rationale": "The entry is in plain and simple language, and the interviewee is asked whether he/she repeated certain sound or words, is not overdoing or making emotional remarks, and is more balanced and objective."}, "time_dimension": {"answer": "Present", "rationale": "Now the keyword \"over and over\" means that it is an act that is now being performed, while the phrase \"some voices or words\" indicates the object and content of such an act. It is therefore judged that the entry is an inquiry as to whether the person being tested is now often repeating certain voices or words."}, "sentence_type": "Imperative sentence", "pos": {"nouns": {"Once": 1, "Some": 1, "Sound": 1, "Word": 1}, "verbs": {"Repeat": 1}, "adverbs": {"Over and over": 1}, "numerals": {"46": 1}}}, {"content": "47 staring at people, like they're gonna look at them.", "word_count": 12, "label": "", "sentence_structure": "Subject鈥搗erb鈥揷omplement", "subjective_objective": {"answer": "Objective", "rationale": "Because \"look at people\" and \"seeing to wear\" are observed behaviours or manifestations, not patients' subjective feelings or ideas. So this is an objective description."}, "sentence_sentiment": {"answer": "Negative", "rationale": "Because the act of staring at people is usually considered a negative act."}, "language_style": {"answer": "Calm and direct", "rationale": "The entry describes the behaviour of the person under observation in a direct and concise manner, without any emotional or subjective assessment, and with a more calm and objective language style."}, "time_dimension": {"answer": "Present", "rationale": "The keywords are \"sighting\" and \"like,\" and the verbs are indicative of the current behavior and state, so the entry describes a current situation."}, "sentence_type": "Affirmative declarative sentence", "pos": {"nouns": {"Eye": 2, "person": 1, "Questionnaire": 1}, "verbs": {"Keep staring": 1, "Look": 2, "I think so": 1, "Yes": 1, "See through": 1}, "adjectives": {"Keep staring": 1, "Like": 1}, "numerals": {"47": 1}, "pronouns": {"he": 1}}}, {"content": "Repeated questions or answers", "word_count": 10, "label": "", "sentence_structure": "Subject鈥搗erb鈥揷omplement", "subjective_objective": {"answer": "Subjective", "rationale": "Because it's the patient's subjective feelings and experiences."}, "sentence_sentiment": {"answer": "Negative", "rationale": "Because \"repeat or answer\" is a passive act, it may indicate that the person lacks the capacity to innovate and express himself."}, "language_style": {"answer": "Calm and direct", "rationale": "The entry is asked in a more objective and direct manner, without too many rhetorical or emotional renderings, and in a more cold and straightforward manner."}, "time_dimension": {"answer": "Present", "rationale": "The entry is for the purpose of asking the testee whether he or she often repeats the words or answers of others, and the time dimension is now judged."}, "sentence_type": "Affirmative declarative sentence", "pos": {"nouns": {"48": 1, "others": 1, "Problem": 1, "Answer": 1}, "verbs": {"Repeat": 1, "Question": 1, "Do it": 1}, "adverbs": {"very": 1}, "numerals": {"48": 1}, "pronouns": {"self": 1}}}, {"content": "49 often unaware of the environment and may not care about the dangerous environment", "word_count": 24, "label": "", "sentence_structure": "Subject鈥搗erb鈥搊bject", "subjective_objective": {"answer": "Objective", "rationale": "Since awareness of the environment and attitudes towards the dangerous environment are objective facts experienced by patients, it is subjective to examine subjective ideas."}, "sentence_sentiment": {"answer": "Negative", "rationale": "Because \"not knowing the environment\" and \"not caring about the dangerous environment\" are unsafe acts."}, "language_style": {"answer": "Calm and direct", "rationale": "The entry uses more direct language to describe the possible existence of the person under investigation, with no excessive adoration or emotional colour, and is more in line with the linguistic style of the factual description."}, "time_dimension": {"answer": "Present", "rationale": "The entry describes the behaviour or state of the person being tested during the current period, namely, \"often unconsciously in an environment\" and \"possibly unattractive to a dangerous environment\". These descriptions are based on an assessment of the current situation and therefore are judged to be \"now\"."}, "sentence_type": "Affirmative declarative sentence", "pos": {"nouns": {"49": 1, "Environment": 2, "Danger": 1}, "verbs": {"Yes": 1, "Awareness": 1, "in": 2, "I don't care": 1}, "adjectives": {"Regular": 1}, "numerals": {"49": 1}, "classifiers": {"individual": 1}}}, {"content": "50 especially likes to play, to play, to play, to play, to play, to play, to play, to play, to play, to play, to play, to play, to play, to play, to play, to play, to play, to play, to play, to play, to play, to play, to play, to play, to play, to play, to play, to play, to play, to play, to play, to play, to play, to play, to play, to play, to play, to play, to play, to play, to play, to play, to play, to play, to play, to play, to play, to play, to play, to play, to run, to run, to run, to jump, to jump, to jump, to tap, to tap, to tap, to tap, to tap, to knock.", "word_count": 36, "label": "", "sentence_structure": "Subject鈥搗erb鈥搊bject", "subjective_objective": {"answer": "Subjective", "rationale": "Because the words \"specially like\" and \"smuggling\" and \"smuggling\" are expressions of the patient's personal feelings and attitudes, it's the patient's subjective thoughts. So the questionnaire entry is based on subjective considerations."}, "sentence_sentiment": {"answer": "Negative", "rationale": "Because the behaviour of \"smuggling, fascinated with monotonous things or games\" in the entries may affect normal life and socialization, it is negative."}, "language_style": {"answer": "Tactful and warm", "rationale": "The entry is more euphemistic, takes the form of a question phrase and uses the words \"specially like\" and \"smuggling\" to express more gentleness and affection, without too direct or commandive a tone, and is more suitable for interaction with the person under investigation."}, "time_dimension": {"answer": "Present", "rationale": "The entry describes a way of acting, i.e., especially in a way that is enchanted by one-way games and so forth. This may be an ongoing act, because it is now done in a state of fashion (e.g. \"specially like\" and \"infindably...\"), which expresses customary or recurrent descriptions over time."}, "sentence_type": "Imperative sentence", "pos": {"nouns": {"50": 1, "Special": 1, "Yeah": 1, "-Playing": 1, "I'm obsessed": 1, "Undo": 1, "Something": 1, "Game": 1, "Activities": 1, "Back and forth": 1, "Run": 1, "It's not over": 1, "Boom": 1, "Jump": 1, "Shoot": 1, "Knock": 1}, "verbs": {"Let's go": 1, "Knock": 1, "Yeah": 1, "-Playing": 1, "I'm obsessed": 1, "Undo": 1, "Something": 1, "Game": 1, "Activities": 1, "Back and forth": 1, "Run": 1, "It's not over": 1, "Boom": 1, "Jump": 1}, "adjectives": {"Special": 1, "Undo": 1}, "adverbs": {"It's not over": 1}, "numerals": {"50": 1}, "classifiers": {"individual": 1}, "pronouns": {"Wait": 1}}}, {"content": "51 for something around.", "word_count": 11, "label": "", "sentence_structure": "Subject鈥搗erb鈥揷omplement", "subjective_objective": {"answer": "Subjective", "rationale": "Because it's the patient's subjective attitude to smell, touch or taste."}, "sentence_sentiment": {"answer": "Neutral", "rationale": "Because the entry is not clearly emotional, it merely describes a person ' s behaviour."}, "language_style": {"answer": "Tactful and warm", "rationale": "The language style of the entry is more euphemistic, and the use of the verb \"sniff, touch, or taste\" to describe people's behaviour, rather than directly to say that they are doing so, makes the whole issue more friendly and inclusive. At the same time, the expression \"close things\" adds more affection and makes the questionnaire more acceptable."}, "time_dimension": {"answer": "Present", "rationale": "The key word in the sentence, \"likes to smell, touch or taste\", can be judged to be asking the subject about the current behaviour or habits, so the answer is now."}, "sentence_type": "Affirmative declarative sentence", "pos": {"nouns": {"Around": 1, "Something": 1}, "verbs": {"Yeah": 1, "Smell": 1, "Touch": 1, "Taste it": 1}, "numerals": {"51": 1}}}, {"content": "52 without visual reaction to living people.", "word_count": 14, "label": "", "sentence_structure": "Subject鈥搗erb鈥揷omplement", "subjective_objective": {"answer": "Objective", "rationale": "Because \"the lack of visual reaction to the living\" is the behaviour and state of the patient, not their subjective feelings or ideas. So, this is an objective look."}, "sentence_sentiment": {"answer": "Negative", "rationale": "Because the description \"no look\" in the entry reflects a lack of social behaviour, it may indicate a certain negative aspect of social incapacity or unwillingness to communicate with others."}, "language_style": {"answer": "Calm and direct", "rationale": "The language style of the entry is relatively calm and straightforward, and it describes a phenomenon in a concise and clear language, without too many qualifiers or emotional expressions. Such expressions make the questionnaire more objective and accurate and help to collect real data."}, "time_dimension": {"answer": "Present", "rationale": "So the time dimension is now judged."}, "sentence_type": "Negative declarative sentence", "pos": {"nouns": {"Livers": 2, "Visual Reaction": 1, "Somebody": 1}, "verbs": {"have": 1, "None": 1, "Look": 1}, "adverbs": {"Regular": 1}, "numerals": {"52": 1}}}, {"content": "The troupe of complex rituals is like a coven of demons (e.g. walking by a certain route, having to place something before dinner or doing something before doing it, or otherwise not sleeping.", "word_count": 65, "label": "", "sentence_structure": "Subject鈥搗erb鈥搊bject", "subjective_objective": {"answer": "Subjective", "rationale": "Because these rituals are subjective ideas and behaviours of the patient."}, "sentence_sentiment": {"answer": "Negative", "rationale": "Because the descriptions of \"entangled in complex rituals\" and \"entangled in the coven\" in the entries give a negative feeling. This suggests that the interviewee may have some kind of psychological problem or coercion, which is a negative emotion or attitude."}, "language_style": {"answer": "Tactful and warm", "rationale": "The language style of the entry is more euphemistic and warm. It guides the interviewer to wonder if he or she has a similar tendency and does not use strong negative terms or tone."}, "time_dimension": {"answer": "Present", "rationale": ""}, "sentence_type": "Affirmative declarative sentence", "pos": {"nouns": {"Ceremonial acts": 1, "The Ring": 1, "Route": 1, "Rice": 1, "Something": 1, "Location": 1, "Actions": 1, "Things": 1, "Sleep": 1, "Eat": 1}, "verbs": {"- A entanglement": 1, "Let's go": 1, "Fire": 1, "Huh": 1, "Do it": 1, "Sleep": 1, "Eat": 1}, "adjectives": {"Complex": 1, "I'm sure": 2, "Otherwise": 1}, "adverbs": {"Yeah": 1, "Otherwise": 1}, "numerals": {"53": 1}, "pronouns": {"it": 1}}}, {"content": "54 destroys things often.", "word_count": 23, "label": "", "sentence_structure": "Subject鈥搗erb鈥揷omplement", "subjective_objective": {"answer": "Subjective", "rationale": "Because it is the subjective behaviour and attitude of the patient that destroys things often."}, "sentence_sentiment": {"answer": "Negative", "rationale": "Because the \"threading\" act is negative."}, "language_style": {"answer": "Calm and direct", "rationale": "This is a direct question of whether the interviewees often destroy things without any aesthetic or euphemism. This question usually requires a clear answer from the interviewees, so the language style is more direct and objective."}, "time_dimension": {"answer": "Present", "rationale": "Thus, judging from the temporal dimension, the article is intended to describe the present situation, that is, the present behaviour of the person being tested."}, "sentence_type": "Affirmative declarative sentence", "pos": {"nouns": {"54": 1, "Something": 2, "Toys": 1, "Home": 1, "Everything": 1, "Use the gear": 1, "Soon": 1, "Broken": 1}, "verbs": {"Destruction": 1, "Here": 1, "Do it": 1}, "adverbs": {"Regular": 1, "Just": 1}, "numerals": {"54": 1, "2": 1}, "classifiers": {"individual": 1}}}, {"content": "55 years before 2 and a half, they found a child with a delay in development.", "word_count": 14, "label": "", "sentence_structure": "Subject鈥搗erb鈥搊bject", "subjective_objective": {"answer": "Objective", "rationale": "Because \"55 found a child delayed in development before the age of two and a half\" is an observed fact, not a subjective feeling or attitude of the patient. This is an objective observation."}, "sentence_sentiment": {"answer": "Negative", "rationale": "Because the description of \"child delayed development\" in the entry is negative."}, "language_style": {"answer": "Tactful and warm", "rationale": "The entry is more personal and interactive when it is spoken in a gentle, inviting tone, rather than in direct statements or orders. At the same time, the entry does not overemphasize the child鈥檚 development, but rather provides a fact that allows the interviewee to judge and think for himself."}, "time_dimension": {"answer": "Past", "rationale": ""}, "sentence_type": "Affirmative declarative sentence", "pos": {"nouns": {"55": 1, "Two and a half": 1, "Son": 1, "Development": 1, "Delay": 1}, "verbs": {"Found": 1}, "numerals": {"in": 1, "Before": 1, "Just": 1, "55": 1, "Two and a half": 1}, "pronouns": {"he": 1}}}, {"content": "At least 15 short sentences, but not more than 30 short sentences, are used in daily life (under 15 also 鈥渟ingle鈥?", "word_count": 26, "label": "", "sentence_structure": "Subject鈥搗erb鈥搊bject", "subjective_objective": {"answer": "Objective", "rationale": "The purpose of this questionnaire is to describe the behaviour of individuals in their daily lives, i.e. the number of sentences used, which is an objective observation and record. The entry is therefore viewed from an objective perspective."}, "sentence_sentiment": {"answer": "Neutral", "rationale": "Because the entry is not emotional, it only describes the number of sentences that are exchanged."}, "language_style": {"answer": "Calm and direct", "rationale": "The language style of the entry is more direct and objective, and there is no excessive amount of embellishment or emotional rendering. It describes the information to be filled in in a simple and clear language, allowing respondents to understand clearly and answer questions as requested."}, "time_dimension": {"answer": "Present", "rationale": "The entry describes the person's current behavior or state, the keywords being \"in everyday life,\" \"communication\" and \"short sentences.\" These words point to what is happening and is now going on. So the temporal dimension is now."}, "sentence_type": "Imperative sentence", "pos": {"nouns": {"Life": 1, "Short sentences": 2, "Communications": 1}, "verbs": {"Use": 1, "Fight": 1, "Communications": 1}, "adjectives": {"Day": 1, "At least": 1, "More than": 1, "Not really": 1}, "numerals": {"56": 1, "15": 2, "30": 1}, "classifiers": {"individual": 2}, "pronouns": {"Yeah": 1}}}, {"content": "57 has been looking at one place for a long time.", "word_count": 15, "label": "", "sentence_structure": "Subject鈥搗erb鈥揷omplement", "subjective_objective": {"answer": "Objective", "rationale": "The fact that a place is a subject of prolonged suspicion is an objective fact."}, "sentence_sentiment": {"answer": "Neutral", "rationale": "Because the entry does not have any emotional color."}, "language_style": {"answer": "Calm and direct", "rationale": "The language style of the entry is more calm and straightforward, describing behaviour characteristics in a concise and clear manner, and does not involve too many emotional renderings or rhetorical techniques."}, "time_dimension": {"answer": "Present", "rationale": "\"Long time\" and \"Scrutinizing a place\" and \"Scrambling a place\" all indicate that this is a state of fact or behaviour that is taking place, so the temporal dimension of this entry is now."}, "sentence_type": "Affirmative declarative sentence", "pos": {"nouns": {"Local": 1, "Stay": 1}, "verbs": {"Doubt": 1, "Long time": 1, "Look": 1}, "adverbs": {"Long time": 1, "Stay still": 1}, "numerals": {"57": 1}}}]}

{"questionnaire_title": "Social evasive and distress tables (SAD)", "questionnaire_category": "Behavioral questionnaire", "item_count": 28, "emotion_shift_overall": "From negative to positive", "language_style_overall": "Tactful and warm", "items": [{"content": "I feel relaxed even in unfamiliar social situations.", "word_count": 18, "label": "", "sentence_structure": "Subject鈥搗erb鈥揷omplement", "subjective_objective": {"answer": "Subjective", "rationale": "Because it is the patient ' s subjective feeling, not the objective situation."}, "sentence_sentiment": {"answer": "Positive", "rationale": "Because even in strange social settings, I feel relaxed is a positive emotional experience."}, "language_style": {"answer": "Tactful and warm", "rationale": "The entry asks the interviewees in a positive and positive way, rather than directly questioning or criticizing them. Moreover, it uses the phrase \"still relaxing\" to express a positive mentality and to try to resonate and identify with the interviewees. This euphemism and comfort can make them more receptive to the problem and more willing to share their views and feelings."}, "time_dimension": {"answer": "Present", "rationale": ""}, "sentence_type": "Imperative sentence", "pos": {"nouns": {"Socially": 1}, "verbs": {"I feel": 1, "Relax": 1, "Even if": 1, "in": 1, "Still": 1}, "adjectives": {"I don't know": 1}, "adverbs": {"Still": 1}, "numerals": {"1": 2}, "pronouns": {"I": 1}}}, {"content": "I tried to avoid forcing me to participate in the pay.", "word_count": 17, "label": "", "sentence_structure": "Subject鈥搗erb鈥搊bject", "subjective_objective": {"answer": "Subjective", "rationale": "Because \"as much as possible\" and \"as much as \"as much as possible\" are the attitudes and behaviours of the patient, it is a subjective examination."}, "sentence_sentiment": {"answer": "Negative", "rationale": "Because \"as much as possible\" in the entry indicates the author's negative attitude towards such social behaviour."}, "language_style": {"answer": "Tactful and warm", "rationale": "The entry expresses a displeasure at being forced to participate in a relationship by using the words \"as much as possible to avoid\" and \"the situational\" and does not directly reject or criticize this way, but rather expresses its views in a euphemism and moderation. This language style is more personal and interactive."}, "time_dimension": {"answer": "Present", "rationale": "So the time dimension of this questionnaire entry is now."}, "sentence_type": "Imperative sentence", "pos": {"nouns": {"Situation": 1, "Professional relations": 1}, "verbs": {"Try": 1, "Avoid": 1, "Participation": 1}, "adjectives": {"I": 1}, "adverbs": {"Try": 1}, "classifiers": {"individual": 1}}}, {"content": "I'm easy to relax when I'm with strangers.", "word_count": 14, "label": "", "sentence_structure": "Subject鈥搗erb鈥揷omplement", "subjective_objective": {"answer": "Subjective", "rationale": "Because \"relax\" is a subjective feeling."}, "sentence_sentiment": {"answer": "Positive", "rationale": "Because the \"easy\" behavior in the entries is positive, and it's good to be with strangers."}, "language_style": {"answer": "Tactful and warm", "rationale": "The entry is more adept and interactive when it is spoken in a gentle and inviting manner, rather than by direct statements or orders."}, "time_dimension": {"answer": "Present", "rationale": "According to the two key words \"I\" and \"now,\" \"I'm with strangers\" describes a state of behaviour, and \"easy to relax\" indicates an emotional reaction in this state. So the entry is to ask the subject about the emotional reaction of his current relationship with strangers, and the temporal dimension is now."}, "sentence_type": "Affirmative declarative sentence", "pos": {"nouns": {"I": 1, "Stranger": 1, "Time": 1}, "verbs": {"in": 1, "Together": 1, "Easy": 1, "Relax": 1}, "adjectives": {"very": 1}, "adverbs": {"Same": 1}, "numerals": {"3": 1}, "classifiers": {"individual": 1}}}, {"content": "I don't really want to leave people.", "word_count": 11, "label": "", "sentence_structure": "Subject鈥揷opula鈥損redicative", "subjective_objective": {"answer": "Subjective", "rationale": "Because \"want to avoid people\" is a subjective attitude and a will."}, "sentence_sentiment": {"answer": "Neutral", "rationale": "The entry is not clearly positive or negative, but rather describes a person ' s attitude or behaviour."}, "language_style": {"answer": "Calm and direct", "rationale": "The entry is more calm and straightforward in language, with no excessive phrasing or emotional colour. It asks if the interviewee is willing to interact with others, with a temperature and without being taken seriously.\""}, "time_dimension": {"answer": "Present", "rationale": "Time dimension is judged now."}, "sentence_type": "Negative declarative sentence", "pos": {"nouns": {"Avoidance": 1, "People": 1}, "verbs": {"Go on": 1, "Yes": 1}, "adverbs": {"not": 1, "Special": 1, "Don't": 1}, "numerals": {"4": 1}, "pronouns": {"I": 1}}}, {"content": "I usually find social scenes disturbing.", "word_count": 15, "label": "", "sentence_structure": "Subject鈥揷opula鈥損redicative", "subjective_objective": {"answer": "Subjective", "rationale": "The entry is about the patient's psychological feelings and subjective views."}, "sentence_sentiment": {"answer": "Negative", "rationale": "Because the description \"frustrated\" in the entry is negative."}, "language_style": {"answer": "Tactful and warm", "rationale": "The language style of the entry is euphemistic, using the words \"normal\" and \"disturbing\" to express views of social settings, is not too direct or emotional, gives a gentle, friendly feeling and makes it easier to resonate and respond to the interviewee."}, "time_dimension": {"answer": "Present", "rationale": "The entry \"I usually find\" indicates that it is a regular, customary act and is in progress, so it points to the present. Moreover, \"discovery\" is a verb and a verb, which requires a guest, and \"disturbed\" social scene is its guest. So, the entry \"I usually find social occasions disturbing\" is asking the testee whether he or she is currently suffering from subjective feelings and experiences, that is, whether he or she is distraught in social settings."}, "sentence_type": "Negative declarative sentence", "pos": {"nouns": {"Questionnaire Entry": 1, "5": 1, "Socially": 1, "I'm upset": 1}, "verbs": {"Usually": 1, "Found": 1, "You're": 1}, "adverbs": {"General": 1}, "numerals": {"5": 1}, "classifiers": {"individual": 1}, "pronouns": {"I": 1}}}, {"content": "Six, I usually feel calm and comfortable in social settings.", "word_count": 15, "label": "", "sentence_structure": "Subject鈥揷opula鈥損redicative", "subjective_objective": {"answer": "Subjective", "rationale": "Because \"feeling calm and comfort\" is the patient's subjective feeling and experience, which reflects how he feels in social settings. This subjectivity includes not only emotional states, but also cognitive, sensory and evaluation aspects, so the questionnaire entry is a subjective one."}, "sentence_sentiment": {"answer": "Positive", "rationale": "Because the attitude and experience of \"feeling calm and comfort\" in the entries is positive."}, "language_style": {"answer": "Tactful and warm", "rationale": "The entry uses a gentle and inviting tone for dialogue and a positive term \"silence and comfort\", which is more adept and interactive."}, "time_dimension": {"answer": "Present", "rationale": "The entry describes the feelings and experiences of the person being tested in the current social setting, and thus the time dimension is now judged."}, "sentence_type": "Affirmative declarative sentence", "pos": {"nouns": {"Socially": 1, "I": 1, "Usually": 1, "Feel": 1, "Calm down": 1, "Comfortable": 1}, "verbs": {"have": 1}, "adverbs": {"in": 1}, "numerals": {"6": 1}}}, {"content": "When I talk to people of the opposite sex, I usually feel relaxed.", "word_count": 14, "label": "", "sentence_structure": "Subject鈥揷opula鈥損redicative", "subjective_objective": {"answer": "Subjective", "rationale": "Because \"relax\" is a personal feeling or emotional experience, and it's a subjective view of the patient."}, "sentence_sentiment": {"answer": "Neutral", "rationale": "Because the entry describes a feeling, it does not address positive or negative factors such as conduct, purpose or quality and is therefore neutral."}, "language_style": {"answer": "Tactful and warm", "rationale": "The entry is more adept and interactive when it is spoken in a gentle and inviting manner, rather than by direct statements or orders."}, "time_dimension": {"answer": "Present", "rationale": "The entry describes the current feelings or state of the testee, the keyword \"usually feeling relaxed\", which means that the testee is often comfortable with the same sex, and is a state of conduct."}, "sentence_type": "Affirmative declarative sentence", "pos": {"nouns": {"Heterosexual": 1, "Talk": 1}, "verbs": {"Usually": 1, "Feel": 1, "Relax": 1}, "adjectives": {"Same": 1}, "numerals": {"7": 1}}}, {"content": "I try not to talk to anyone unless I know them very well.", "word_count": 15, "label": "", "sentence_structure": "Subject鈥搗erb鈥揷omplement", "subjective_objective": {"answer": "Subjective", "rationale": "Because the descriptions \"defended speaking\" and \"unless they are particularly familiar\" are based on patients' subjective views, reflecting their attitudes and perceptions of interpersonal interaction. The questionnaire is therefore mainly about examining patients' subjective attitudes."}, "sentence_sentiment": {"answer": "Negative", "rationale": "Because the \"avoid talking\" act in the entry is negative."}, "language_style": {"answer": "Tactful and warm", "rationale": "The entry is more euphemistic, soft-worded, and not too direct or hard-line language to describe the behaviour of the person interviewed. It uses a more implicit expression that leads the person to think about his behaviour and encourages them to try to change. This expression is more accompanimental and gentle, and helps to create a good atmosphere of interaction."}, "time_dimension": {"answer": "Present", "rationale": "The entry \"I try to avoid\" and \"unless I'm particularly familiar\" suggests that this is the present situation, so the temporal dimension is now."}, "sentence_type": "Affirmative declarative sentence", "pos": {"nouns": {"8": 1, "I": 2, "He's": 1, "Speech": 1, "Yes": 1}, "verbs": {"Avoid": 1, "and": 1, "Speak": 1}, "adverbs": {"Try": 1, "Unless": 1}, "numerals": {"8": 1, "Special": 1}, "pronouns": {"He's": 1}}}, {"content": "If there's a chance to meet a new guy, I'll catch it.", "word_count": 16, "label": "", "sentence_structure": "Subject鈥搗erb鈥搊bject", "subjective_objective": {"answer": "Subjective", "rationale": "This is because the patient ' s attitude and ideas about opportunities are subjective."}, "sentence_sentiment": {"answer": "Positive", "rationale": "Because \"Take the chance\" in the entry expresses positive attitudes and behaviour."}, "language_style": {"answer": "Tactful and warm", "rationale": "The entry is more adept and interactive when it is spoken in a gentle and inviting manner, rather than by direct statements or orders."}, "time_dimension": {"answer": "Future", "rationale": "The keyword \"if...\" and \"capture\" suggests that this is a vision and a vision for the future, so the entry describes future behaviour or mentality."}, "sentence_type": "Affirmative declarative sentence", "pos": {"nouns": {"New guy": 1, "Opportunities": 1}, "verbs": {"have": 1, "Grab him": 1, "Stay": 1, "Yes": 1}, "adverbs": {"If there's a chance to meet a new guy, I'll catch it": 1}, "numerals": {"9": 1}}}, {"content": "I'm usually anxious and nervous about having a different sex at an informal party.", "word_count": 24, "label": "", "sentence_structure": "Subject鈥搗erb鈥揷omplement", "subjective_objective": {"answer": "Subjective", "rationale": "Because \"fear anxiety and stress\" is the patient's subjective feeling."}, "sentence_sentiment": {"answer": "Negative", "rationale": "Because \"anxiety and stress\" in the entry is a sign of negative emotion."}, "language_style": {"answer": "Calm and direct", "rationale": ""}, "time_dimension": {"answer": "Past", "rationale": "Past."}, "sentence_type": "Negative declarative sentence", "pos": {"nouns": {"Party": 1, "Heterosexual": 2, "Anxiety": 1, "Nervous": 1}, "verbs": {"have": 1, "Participation": 1, "I think": 1, "Anxiety": 1, "Nervous": 1}, "adjectives": {"Informal": 1}, "numerals": {"10": 1}, "pronouns": {"I": 1}}}, {"content": "I'm usually anxious to be with people, unless I know them very well.", "word_count": 22, "label": "", "sentence_structure": "Subject鈥揷opula鈥損redicative", "subjective_objective": {"answer": "Subjective", "rationale": "Because anxiety is a subjective emotional experience and requires special familiarity to alleviate it, it is the individual feeling and judgement of the patient."}, "sentence_sentiment": {"answer": "Negative", "rationale": "Because the description of \"fearing\" in the entry is negative."}, "language_style": {"answer": "Tactful and warm", "rationale": ""}, "time_dimension": {"answer": "Present", "rationale": ""}, "sentence_type": "Negative declarative sentence", "pos": {"nouns": {"Questionnaire": 1, "Entry": 1, "I": 2, "People": 1, "Anxiety": 1, "Special": 1, "Yes": 1}, "verbs": {"I feel": 1, "Usually": 1, "Together": 1, "and": 3, "Unless": 1}, "adjectives": {"Special": 1}, "adverbs": {"Together": 1, "Usually": 1}, "numerals": {"11": 1}, "classifiers": {"individual": 1}, "pronouns": {"they": 1}}}, {"content": "I usually feel relaxed when I'm with a group.", "word_count": 15, "label": "", "sentence_structure": "Subject鈥揷opula鈥損redicative", "subjective_objective": {"answer": "Subjective", "rationale": "Because feeling relaxed is a subjective emotional experience and feeling."}, "sentence_sentiment": {"answer": "Positive", "rationale": "Because the \"fright to relax\" attitude in the entry is positive."}, "language_style": {"answer": "Tactful and warm", "rationale": "The language style of the entry is euphemistic. It asks the respondents, in a gentle and inviting tone, whether they will feel relaxed in the group, which is more personal and interactive, and avoids too direct or command-style tone."}, "time_dimension": {"answer": "Present", "rationale": ""}, "sentence_type": "Affirmative declarative sentence", "pos": {"nouns": {"person": 1, "Time": 1, "Relax": 1, "Population": 1}, "verbs": {"in": 1, "Together": 2, "I feel": 1, "and": 1}, "adjectives": {"Usually": 1}, "numerals": {"12": 1}, "classifiers": {"individual": 1}, "pronouns": {"I": 1}}}, {"content": "I've always wanted to leave the crowd.", "word_count": 8, "label": "", "sentence_structure": "Subject鈥搗erb鈥揷omplement", "subjective_objective": {"answer": "Subjective", "rationale": "Because \"want\" is patient's subjective attitude."}, "sentence_sentiment": {"answer": "Negative", "rationale": "The behaviour based on \"leave the crowd\" is usually negative and may indicate, for example, the individual's feelings of isolation or social fear."}, "language_style": {"answer": "Calm and direct", "rationale": "The entry is a direct statement asking the respondents whether they often have the idea of leaving the crowd, without using excessive rhetoric or emotional rendering, and in a more balanced and objective way, as a calm and direct language style."}, "time_dimension": {"answer": "Present", "rationale": "The keyword \"often wants\" indicates the frequency of the act, which is customary, and therefore the temporal dimension is now judged."}, "sentence_type": "Affirmative declarative sentence", "pos": {"nouns": {"Population": 1, "13": 1}, "verbs": {"Yes": 1, "Leave": 1}, "numerals": {"Regular": 1}}}, {"content": "I was uncomfortable with being in a crowd I didn't know.", "word_count": 18, "label": "", "sentence_structure": "Subject鈥揷opula鈥損redicative", "subjective_objective": {"answer": "Subjective", "rationale": "Because it is a manifestation of a personal feeling and subjective attitude."}, "sentence_sentiment": {"answer": "Negative", "rationale": "Because the emotional reaction of \"discomfort\" in the entry is negative, it shows that the interviewee may have social fear, discomfort and negative emotional tendencies in a strange environment."}, "language_style": {"answer": "Tactful and warm", "rationale": "The entry is expressed in descriptive language, emphasizing subjective feelings, rather than direct orders or evaluations. This euphemism makes the interviewees more willing to share their real thoughts and feelings."}, "time_dimension": {"answer": "Present", "rationale": "\"In the midst of unknown people\" describes the situation at hand, and \"I feel uncomfortable\" is the feeling or behaviour at this moment, so the entry reflects the state of things."}, "sentence_type": "Negative declarative sentence", "pos": {"nouns": {"Population": 1, "I": 1}, "verbs": {"Stand up": 1, "I feel": 1, "I'm not comfortable": 1}, "adjectives": {"No, I don't": 1}, "adverbs": {"in": 1}, "numerals": {"14": 1}}}, {"content": "I usually relax when I first meet someone.", "word_count": 16, "label": "", "sentence_structure": "Subject鈥揷opula鈥損redicative", "subjective_objective": {"answer": "Subjective", "rationale": "Because the entry relates to the feelings and attitudes of individuals, it is an examination of patients ' subjective experiences and thoughts."}, "sentence_sentiment": {"answer": "Positive", "rationale": "Because the expression \"usually relaxed\" indicates that the person is usually able to remain relaxed at first sight, a positive attitude or quality."}, "language_style": {"answer": "Tactful and warm", "rationale": "The language style of the entry is more euphemistic, and by using the term \"usually relaxed\" it avoids the use of too direct or strong expressions, while at the same time being able to convey its feelings and views. It is more personal and interactive, and it helps to establish good communication with the respondents."}, "time_dimension": {"answer": "Present", "rationale": "\"usually relaxed\" means \"normal, customary\" behaviour, and \"yes\" adopts the general current, which means that the act not only has occurred in the past, but may still continue. Thus, the entry asks about the person's current behaviour or state, and the temporal dimension is now judged."}, "sentence_type": "Affirmative declarative sentence", "pos": {"nouns": {"Initial": 1, "I met him": 1, "person": 1}, "verbs": {"be": 1, "Usually": 1, "Relax": 1}, "numerals": {"15": 1}, "pronouns": {"I": 1}}}, {"content": "Sixteen people introduced me to anxiety.", "word_count": 15, "label": "", "sentence_structure": "Subject鈥搗erb鈥揷omplement", "subjective_objective": {"answer": "Subjective", "rationale": "As stress and anxiety are the psychological feelings of the patient, they are subjective."}, "sentence_sentiment": {"answer": "Negative", "rationale": "Because the description of \"strained anxiety\" in this entry is negative emotional."}, "language_style": {"answer": "Tactful and warm", "rationale": "This language style makes it easier for the interviewee to share his real thoughts and feelings. It also conveys concern and respect for the interviewee and helps to create a good atmosphere of communication."}, "time_dimension": {"answer": "Past", "rationale": "So the output should be \"the past.\""}, "sentence_type": "Affirmative declarative sentence", "pos": {"nouns": {"16": 1, "others": 1, "Anxiety": 1}, "verbs": {"Introduction": 1, "鈾?Make you feel 鈾?: 1}, "adjectives": {"Nervous": 1}}}, {"content": "17, even though there are people in the room, I might go in.", "word_count": 18, "label": "", "sentence_structure": "Subject鈥搗erb鈥揷omplement", "subjective_objective": {"answer": "Subjective", "rationale": "Although, it may be the patient ' s subjective idea, the view of the questionnaire entry is subjective."}, "sentence_sentiment": {"answer": "Positive", "rationale": "Because despite the description of \"a room full of living people,\" the behavior \"I'll go in\" shows a positive, courageous attitude."}, "language_style": {"answer": "Tactful and warm", "rationale": "The entry is more personal and interactive when it is spoken in a gentle and inviting manner, rather than in direct statements or orders. At the same time, the article is also more objective and has no emotive tone."}, "time_dimension": {"answer": "Present", "rationale": "In the entry, \"may\" indicates that this is a future situation, but \"into the room\" and \"into\" are both occurring at the moment. So, time is now."}, "sentence_type": "Affirmative declarative sentence", "pos": {"nouns": {"Room": 1, "Livers": 1}, "verbs": {"Full": 1, "Yes": 2, "In": 1, "Go on": 1}, "adjectives": {"鈾?Though 鈾?: 1, "Or is it": 1}, "adverbs": {"Maybe": 1}, "numerals": {"17": 1, "Both": 1}, "pronouns": {"I": 1}}}, {"content": "I'll avoid going up and joining a large crowd.", "word_count": 17, "label": "", "sentence_structure": "Subject鈥搗erb鈥搊bject", "subjective_objective": {"answer": "Objective", "rationale": "As this is a choice of behaviour for the patient, it is objective, although it may be influenced by subjective ideas."}, "sentence_sentiment": {"answer": "Negative", "rationale": "Because the \"Run Out\" in the entry is negative."}, "language_style": {"answer": "Calm and direct", "rationale": "The language style of the entry is more direct and concise, with no excessive rhetorical or emotional rendering, but rather directs the question of whether the other person will avoid being included in the crowd."}, "time_dimension": {"answer": "Past", "rationale": "The entry expresses the behaviour and choice of the person who was tested at a point in the past and therefore falls within the category of \"the past\"."}, "sentence_type": "Exclamatory sentence", "pos": {"nouns": {"18": 1, "Population": 1}, "verbs": {"Yes": 1, "Avoid": 1, "Step up": 1, "Add": 1}, "adjectives": {"Large": 1}, "adverbs": {"Go on": 1, "Centre": 1}, "numerals": {"A lot of them": 1}}}, {"content": "When my boss wanted to talk to me, I was happy to talk to him.", "word_count": 17, "label": "", "sentence_structure": "Subject鈥搗erb鈥搊bject", "subjective_objective": {"answer": "Subjective", "rationale": "Because \"happiness\" is an expression of subjective attitude or feelings."}, "sentence_sentiment": {"answer": "Positive", "rationale": "Because the \"happily talking to him\" act in the entry is positive."}, "language_style": {"answer": "Tactful and warm", "rationale": "The entry is more personal and interactive in its expression of a gentle and inviting tone, rather than a direct statement or order. At the same time, the tone of the article gives a positive sense of optimism, which is more acceptable and willing to answer."}, "time_dimension": {"answer": "Present", "rationale": "\"When my supervisor wants to talk to me\" means a normal situation, and \"I'm happy to talk to him\" means an emotional reaction that usually lasts until now. So this entry is mainly about the person's current emotional state."}, "sentence_type": "Affirmative declarative sentence", "pos": {"nouns": {"Sir": 1, "Talk": 2, "Happy": 1}, "verbs": {"Yes": 1, "Same": 1, "Talk": 1, "I'm glad": 1, "With him": 1}, "adverbs": {"very": 2}, "numerals": {"19": 1}, "pronouns": {"I": 1, "he": 1}}}, {"content": "I've always figured out some excuse to avoid socialization.", "word_count": 16, "label": "", "sentence_structure": "Subject鈥搗erb鈥搊bject", "subjective_objective": {"answer": "Subjective", "rationale": "Because \"often think\" is the patient's subjective idea."}, "sentence_sentiment": {"answer": "Negative", "rationale": "Because the \"scrambling social activity\" act is negative."}, "language_style": {"answer": "Calm and direct", "rationale": "The entry is asked in a straightforward manner, is not presented in excessive terms or emotional colours and is more objective."}, "time_dimension": {"answer": "Present", "rationale": "The term \"often\" in this entry indicates a high frequency and habit, and the description \"fortuitive excuse to avoid social activities\" also highlights that the act is present and recurrent. Thus, the temporal dimension of the article is now judged."}, "sentence_type": "Affirmative declarative sentence", "pos": {"nouns": {"I": 1, "- An excuse": 1, "Social events": 1}, "verbs": {"I've got it": 1, "Avoidance": 1}, "adverbs": {"Regular": 1}, "numerals": {"24": 1}}}, {"content": "I'm trying to avoid formal socialization.", "word_count": 12, "label": "", "sentence_structure": "Subject鈥搗erb鈥揷omplement", "subjective_objective": {"answer": "Subjective", "rationale": "Since the purpose of the article contains a subjective attitude and description of conduct \"as far as possible\", \"as far as possible\" and \"shield\", it can be judged as a subjective view."}, "sentence_sentiment": {"answer": "Negative", "rationale": "Because the \"shield\" act in the entry is negative."}, "language_style": {"answer": "Tactful and warm", "rationale": "The language style of the entry is euphemism, because it uses a more euphemistic approach to expressing an attitude of avoiding formal socialization, without being too direct or commandive. At the same time, the expression \"as far as possible\" is used, which also appears to be more moderate and respectful of each other's ideas."}, "time_dimension": {"answer": "Past", "rationale": "If the questionnaire is for a survey over time, it should be judged as the past; if it is for a survey of the current situation, it should be judged as the present."}, "sentence_type": "Affirmative declarative sentence", "pos": {"nouns": {"I": 1, "Socially": 1}, "verbs": {"Avoid": 1, "Try": 1}, "adjectives": {"Formal": 1}, "numerals": {"26": 1}}}, {"content": "22 talking to people on the Internet or at social gatherings is not a problem for me.", "word_count": 20, "label": "", "sentence_structure": "Subject鈥搗erb鈥搊bject", "subjective_objective": {"answer": "Subjective", "rationale": "This is because patients self-evaluate their social abilities and belong to their subjective feelings and perceptions."}, "sentence_sentiment": {"answer": "Positive", "rationale": "It is a positive thing that the entry indicates that communication with people on the Internet or at social gatherings is not a problem for the interviewees."}, "language_style": {"answer": "Tactful and warm", "rationale": "The entry is more adept and interactive when it is spoken in a gentle and inviting manner, rather than by direct statements or orders."}, "time_dimension": {"answer": "Present", "rationale": "Now, \"22 Talk to people on the Internet or social gatherings without a problem\" describes the current capabilities and circumstances, which show that the testees have no difficulty in dealing with this situation. The keyword \"no problem\" also highlights that there are no obstacles to this behaviour, past, present and future. The entry is therefore in the present context."}, "sentence_type": "Affirmative declarative sentence", "pos": {"nouns": {"Online": 1, "Social parties": 1, "person": 1, "Talk": 1}, "verbs": {"and": 1, "not": 1, "Done": 1, "be": 1, "Problem": 1}, "adjectives": {"22": 1}}}, {"content": "I sometimes act as a character for people to introduce themselves to.", "word_count": 15, "label": "", "sentence_structure": "Subject鈥搗erb鈥揷omplement", "subjective_objective": {"answer": "Subjective", "rationale": "Because \"acting as a character for people\" is a behavioral act that reflects the patient鈥檚 behavioural orientation and role identity, and is the patient鈥檚 subjective perception of his role and function in society. So the point of view of this questionnaire is subjective."}, "sentence_sentiment": {"answer": "Neutral", "rationale": "Because the entry does not have any emotional color."}, "language_style": {"answer": "Tactful and warm", "rationale": "The entry uses the term \"sometimes\" to describe its role in human relations, expressing a relatively open and self-deserving attitude. Moreover, the phrase \"act as a role\" is used instead of \"I am...\", and it shows the gentle, friendly language style of the questionnaire."}, "time_dimension": {"answer": "Present", "rationale": "Now, \"sometimes\" means \"sometimes\" means \"sometimes\" is a customary, occasional act, \"bet\" happens in the past, so it's judged now."}, "sentence_type": "Imperative sentence", "pos": {"nouns": {"I": 1, "People": 1, "Role": 1}, "verbs": {"Act as": 1, "Introduction": 1}, "adverbs": {"Sometimes": 1}, "numerals": {"25": 1}}}, {"content": "23 of a large crowd, I'm rarely comfortable.", "word_count": 15, "label": "", "sentence_structure": "Subject鈥揷opula鈥損redicative", "subjective_objective": {"answer": "Subjective", "rationale": "Because feeling comfortable is the experience and feeling inside the patient, it's seen from the patient's subjective perspective."}, "sentence_sentiment": {"answer": "Negative", "rationale": "Because the entry is a negative expression of the emotional experience of individuals who feel uncomfortable in the population."}, "language_style": {"answer": "Calm and direct", "rationale": "The entry directly expresses the subject of questioning the other party ' s social feelings in the crowd, without using excessive rhetorical or emotional rendering, and with a more objective tone."}, "time_dimension": {"answer": "Present", "rationale": "The phrase \"I rarely can\" and \"in a large group\" would make it sound like asking the testee about his current behavior or feelings. So the time dimension would be now."}, "sentence_type": "Negative declarative sentence", "pos": {"nouns": {"Population": 1, "I'm comfortable": 1}, "verbs": {"I feel": 1, "Yes": 1}, "adjectives": {"Large": 1, "A bunch of them": 1, "Very few": 1}, "adverbs": {"in": 1, "Centre": 1, "23": 1}, "numerals": {"23": 1}, "pronouns": {"I": 1}}}, {"content": "I'm usually upset when I'm with a group of people.", "word_count": 17, "label": "", "sentence_structure": "Subject鈥揷opula鈥損redicative", "subjective_objective": {"answer": "Subjective", "rationale": "Because the feeling of unease is the individual's psychological experience and emotional reaction, it is the patient's own subjective feeling."}, "sentence_sentiment": {"answer": "Negative", "rationale": "The entry is a reflection of the negative feelings of a person in a social setting."}, "language_style": {"answer": "Calm and direct", "rationale": "The entry expresses the subject of the question in a direct way, i.e. in situations of tension among the group, without using euphemism or emotional colour to guide the answer."}, "time_dimension": {"answer": "Present", "rationale": ""}, "sentence_type": "Negative declarative sentence", "pos": {"nouns": {"Group": 1, "I'm not sure": 1}, "verbs": {"in": 1, "Together": 1, "Feel": 1, "I'm not sure": 1}, "adverbs": {"Usually": 1}, "numerals": {"20": 1}, "pronouns": {"I": 1}}}, {"content": "I like to avoid crowds.", "word_count": 7, "label": "", "sentence_structure": "Subject鈥搗erb鈥搊bject", "subjective_objective": {"answer": "Subjective", "rationale": "Because \"like\" is a subjective feeling and attitude."}, "sentence_sentiment": {"answer": "Negative", "rationale": "Because the act of \"scrambling the crowd\" in the entry is negative and reflects the possible tendency of the individual to be alone and to escape."}, "language_style": {"answer": "Tactful and warm", "rationale": "The language style of the entry is more euphemistic, with dialogue taking place in an invitation-style rather than direct statements or orders, which is more personal and interactive. At the same time, the purpose of the article is also more moderate, without too much subjectivity, giving people a comfortable sense of nature."}, "time_dimension": {"answer": "Present", "rationale": "It can be inferred from the sentence that it is \"like\" and \"shun\" that it is a description of current habits or tendencies. Although there is no clear time-frame, it is known in context that this is a regular or customary act, so the answer is \"now\"."}, "sentence_type": "Affirmative declarative sentence", "pos": {"nouns": {"Population": 1}, "verbs": {"Yeah": 1, "Get out of the way": 1}, "numerals": {"21": 1}}}, {"content": "I usually get into all the social contacts I can. Whatever social events I can.", "word_count": 34, "label": "", "sentence_structure": "Subject鈥搗erb鈥搊bject", "subjective_objective": {"answer": "Subjective", "rationale": "Since participation in social events is a subjective decision and behaviour of a person, it is the social attitudes and preferences of the individual."}, "sentence_sentiment": {"answer": "Positive", "rationale": "Because the attitudes and behaviours in the entries express the desire and action to actively engage in social activities."}, "language_style": {"answer": "Calm and direct", "rationale": "The language style of the article ' s purpose can therefore be judged as being straightforward."}, "time_dimension": {"answer": "Present", "rationale": ""}, "sentence_type": "Affirmative declarative sentence", "pos": {"nouns": {"I": 3, "- To be with someone": 1, "Various": 1, "Social": 2, "Activities": 2, "General": 1}, "verbs": {"Participation": 2, "Yes": 4, "Go on": 2}, "adjectives": {"Usually": 1, "Whatever it is": 1}, "adverbs": {"Just": 1}, "numerals": {"27": 1}, "classifiers": {"Various": 1, "individual": 1}}}, {"content": "I found it easy to relax when I'm with someone else.", "word_count": 15, "label": "", "sentence_structure": "Subject鈥搗erb鈥揷omplement", "subjective_objective": {"answer": "Subjective", "rationale": "Because \"discovery\" and \"easy\" are the feelings and understandings of the patient, the subjective descriptions."}, "sentence_sentiment": {"answer": "Positive", "rationale": "Because the \"Easy to light when found with others\" behavior and attitude in the entry are positive."}, "language_style": {"answer": "Tactful and warm", "rationale": "The entry uses moderate, encouraging language to guide respondents to share their feelings rather than direct orders or criticism. At the same time, the purpose of the article is more simple and understandable."}, "time_dimension": {"answer": "Present", "rationale": ""}, "sentence_type": "Affirmative declarative sentence", "pos": {"nouns": {"28": 1, "- With someone else": 1, "Together": 1, "Time": 1, "Relax": 1, "Easy": 1}, "verbs": {"Found": 1, "in": 1, "Together": 1}, "adverbs": {"very": 1, "Easy": 1}, "numerals": {"28": 1}, "classifiers": {"individual": 1}}}]}

{"questionnaire_title": "Health status questionnaire (Chinese version of SF-36)", "questionnaire_category": "Mental health", "item_count": 36, "emotion_shift_overall": "From positive to negative", "language_style_overall": "Neutral", "items": [{"content": "1. In general, your state of health is:", "word_count": 11, "label": "", "sentence_structure": "Subject鈥損redicate", "subjective_objective": {"answer": "Subjective", "rationale": "Because the expression \"generally\" and \"you\" shows that this is the subjective view and assessment of the patient's health."}, "sentence_sentiment": {"answer": "Neutral", "rationale": "Because the entry does not have any emotional color."}, "language_style": {"answer": "Calm and direct", "rationale": "The entry is more objective and straightforward in its language style, without using too many rhetorical techniques or highly emotional terms."}, "time_dimension": {"answer": "Present", "rationale": ""}, "sentence_type": "Interrogative sentence", "pos": {"nouns": {"Overall": 1, "Health status": 1}, "verbs": {"be": 1}, "adverbs": {"Speak": 1, "Both": 1}, "numerals": {"1": 2}, "classifiers": {"individual": 1}}}, {"content": "2. Compared to a year ago, you feel that your current state of health is:", "word_count": 18, "label": "", "sentence_structure": "Subject鈥搗erb鈥搊bject", "subjective_objective": {"answer": "Subjective", "rationale": "This is because patients have subjective assessments of themselves, not objective facts."}, "sentence_sentiment": {"answer": "Neutral", "rationale": "Because the entry is concerned with changes in the health status of the individual and does not contain any apparent emotional colour."}, "language_style": {"answer": "Tactful and warm", "rationale": ""}, "time_dimension": {"answer": "Present", "rationale": "The entry asks the testee about his current state of health compared to a year ago, and the temporal dimension is now judged."}, "sentence_type": "Interrogative sentence", "pos": {"nouns": {"Year": 1, "Present": 1, "Health status": 1}, "verbs": {"I think": 1, "- Yeah": 1, "be": 1, "Call": 1}, "adverbs": {"More": 1}, "numerals": {"One": 1}, "pronouns": {"you": 2}}}, {"content": "3. Powerful activity capable of (e.g. running, lifting, intense exercise, etc.)", "word_count": 18, "label": "", "sentence_structure": "Subject鈥搗erb鈥揷omplement", "subjective_objective": {"answer": "Objective", "rationale": "Since it is the patient ' s personal choice and subjective attitude to prefer heavy physical activity, the ability to perform heavy physical activity is judged by his physical condition and the actual situation, and this is an objective entry."}, "sentence_sentiment": {"answer": "Positive", "rationale": "Because heavy physical activity usually brings benefits for physical health."}, "language_style": {"answer": "Calm and direct", "rationale": "This is more in line with the formal and objective requirements of the questionnaire."}, "time_dimension": {"answer": "Present", "rationale": "The entry asks about the current situation or habits of the person being tested, and the keyword \"can\" indicates that this is a capability that can be expressed through the current heavy physical activity. The temporal dimension is therefore now judged."}, "sentence_type": "Interrogative sentence", "pos": {"nouns": {"Physical activity": 1, "Run": 1, "Lifting heavy objects": 1, "Heat": 1}, "verbs": {"Yes": 1, "Like": 1}}}, {"content": "4. Moderate activities (e.g., desk transfer, ground cleaning, exercise, etc.)", "word_count": 13, "label": "", "sentence_structure": "Subject鈥損redicate", "subjective_objective": {"answer": "Objective", "rationale": "Since these activities are specific manifestations of behaviour, they do not require subjective judgement and evaluation by the patient and are therefore examined from an objective perspective."}, "sentence_sentiment": {"answer": "Positive", "rationale": "Because the \"moderate activity\" in the entry is positive, it helps people to stay healthy."}, "language_style": {"answer": "Calm and direct", "rationale": "The entry describes the appropriate physical mode of movement in a concise and clear language, is not over-modified or emotionally rendered and is expressed more directly."}, "time_dimension": {"answer": "Present", "rationale": "The term \"motivation\" in this entry refers to actions that are now under way and therefore is judged to be now."}, "sentence_type": "Interrogative sentence", "pos": {"nouns": {"Moderate": 1, "Activities": 2, "Table": 1, "Sweep": 1, "Fuck": 1}, "verbs": {"Like": 1, "Move": 1, "Do it": 1}, "adverbs": {"Moderate": 1}, "numerals": {"4": 1}, "classifiers": {"Wait": 1}}}, {"content": "5. Hand-held groceries (e.g., buying, shopping, etc.)", "word_count": 12, "label": "", "sentence_structure": "Subject鈥搗erb鈥揷omplement", "subjective_objective": {"answer": "Objective", "rationale": "Because it is an act that can be observed and measured, it is not a subjective attitude or feeling of the patient."}, "sentence_sentiment": {"answer": "Neutral", "rationale": ""}, "language_style": {"answer": "Tactful and warm", "rationale": "The language style of the entry is a warmer type, mainly reflected in a gentle and invited tone of conversation. By using a polite phrase like \"please\" gives people a sense of affection and interaction, and the respondents are more willing to cooperate with the questionnaire. At the same time, the absence of too many professional terms or complex sentences makes the problem easier to understand and increases the participation of the respondents."}, "time_dimension": {"answer": "Present", "rationale": "According to the keywords \"handheld\" and \"shopping items (e.g., buying food, shopping, etc.), this behaviour usually occurs in everyday life and is currently being carried out or has recently been completed. The entry therefore indicates the person being tested for the current act or habit."}, "sentence_type": "Imperative sentence", "pos": {"nouns": {"Handheld": 1, "Miscellaneous general stores": 1, "Like": 1, "Buying food": 1, "Shopping": 1}, "verbs": {"Carry": 1, "Wait": 1}, "numerals": {"5锛?: 1}, "classifiers": {"individual": 1, "Wait": 1}}}, {"content": "6. Upstairs", "word_count": 5, "label": "", "sentence_structure": "Subject鈥搗erb鈥揷omplement", "subjective_objective": {"answer": "Objective", "rationale": "This is because it examines the actual mobility of the patient and does not involve any subjective attitude or emotional colour, and is therefore objective in perspective."}, "sentence_sentiment": {"answer": "Neutral", "rationale": "Because the entry does not have any emotional color."}, "language_style": {"answer": "Tactful and warm", "rationale": "The language style of the entry is euphemism. It asks each other how many floors they are upstairs in a gentle and polite manner, rather than directly ordering or asking questions, which is more acceptable and responsive, and is more friendly."}, "time_dimension": {"answer": "Present", "rationale": "The entry is for questioning the behaviour or frequency of the person being tested, and the time dimension is now judged."}, "sentence_type": "Imperative sentence", "pos": {"nouns": {"Stairs": 1, "Layer": 1}, "verbs": {"Go, go, go": 1}, "numerals": {"How many": 1}}}, {"content": "7. Upper stairs", "word_count": 5, "label": "", "sentence_structure": "Subject鈥搗erb鈥揷omplement", "subjective_objective": {"answer": "Objective", "rationale": "Because the upper stairs are an objective fact, it has nothing to do with the patient ' s subjective feelings."}, "sentence_sentiment": {"answer": "Neutral", "rationale": "Because the entry does not have any emotional color."}, "language_style": {"answer": "Calm and direct", "rationale": "The entry asked if the person surveyed would go up the stairs in the course of his daily activities, with a clear language style, with no excessive adoration and emotional colour, and was a direct expression of calm."}, "time_dimension": {"answer": "Present", "rationale": "The entry describes a specific act - \"upstairs\", with no clear time reference point, and therefore the judgement is now."}, "sentence_type": "Imperative sentence", "pos": {"nouns": {"Stairs": 1}, "verbs": {"Go, go, go": 1}}}, {"content": "8. Bend over, bend your knees, crouch down.", "word_count": 6, "label": "", "sentence_structure": "Subject鈥搗erb鈥揷omplement", "subjective_objective": {"answer": "Objective", "rationale": "Because it is a description of the action, it is in the context of objective observation and documentation, not the subjective feelings or attitudes of the patient."}, "sentence_sentiment": {"answer": "Neutral", "rationale": "Because the entry does not express positive or negative emotional sentiments, it is merely describing an action."}, "language_style": {"answer": "Tactful and warm", "rationale": "The language style of the entry is more moderate, using verbs such as \" bending\", \"claping\" and \"crawling\" to give a soft feeling and not directly ordering or requiring the person interviewed to do these moves, but asking questions and giving tips, which are more personal and interactive."}, "time_dimension": {"answer": "Present", "rationale": "\"Bow, bend, crouch\" is the action that the testee has to perform when completing the investigation, and therefore it is a description of the current behaviour."}, "sentence_type": "Imperative sentence", "pos": {"nouns": {"Bend over": 1, "Crush your knees": 1, "Down": 1}}}, {"content": "9. Travel about 1,500 metres.", "word_count": 8, "label": "", "sentence_structure": "Subject鈥搗erb鈥搊bject", "subjective_objective": {"answer": "Objective", "rationale": "Since 1,500 metres are specific distances, there is no need for subjective judgement or attitude on the part of the patient."}, "sentence_sentiment": {"answer": "Neutral", "rationale": "As the entry merely stated a fact, it did not involve any emotion or attitude."}, "language_style": {"answer": "Tactful and warm", "rationale": "The entry is more euphemistic and friendly, using the word \"please\" to guide respondents to self-assessment, and is not too direct or commandive. At the same time, the description \"walking about 1,500 metres\" is more specific and understandable, allowing respondents to better judge their situation."}, "time_dimension": {"answer": "Past", "rationale": "The entry is an inquiry into the distance and time of the person to walk, and is not clearly time-directed, and therefore cannot be judged as past, present or future."}, "sentence_type": "Imperative sentence", "pos": {"nouns": {"Walk": 1, "1,500 meters": 1, "Road": 1}, "adjectives": {"Around": 1}, "numerals": {"9锛?: 1, "1500": 1}, "classifiers": {"Rice": 1}}}, {"content": "10. Travel about 800 metres.", "word_count": 8, "label": "", "sentence_structure": "Subject鈥搗erb鈥揷omplement", "subjective_objective": {"answer": "Objective", "rationale": "Because this is a specific description of behaviour, it is an objective fact that is being examined."}, "sentence_sentiment": {"answer": "Neutral", "rationale": "Because the entry does not have any emotional color."}, "language_style": {"answer": "Calm and direct", "rationale": "The language style of the entry is more straightforward, with no excessive rhetorical or emotional rendering, and is objective and aimed at communicating clearly the tasks to be performed by the subject of the survey."}, "time_dimension": {"answer": "Past", "rationale": "The entry does not specify the time dimension and therefore does not determine whether it is past, present or future behaviour or mentality."}, "sentence_type": "Imperative sentence", "pos": {"nouns": {"Walk": 1, "Road": 1}, "verbs": {"have": 1}, "adjectives": {"About 800 meters": 1}, "numerals": {"800 meters": 1, "Around": 1}, "classifiers": {"individual": 1}}}, {"content": "11. About 100 metres on foot", "word_count": 7, "label": "", "sentence_structure": "Subject鈥搗erb鈥揷omplement", "subjective_objective": {"answer": "Objective", "rationale": "Since walking about 100 metres is an objective fact, the subjective idea of examining it is subjective."}, "sentence_sentiment": {"answer": "Neutral", "rationale": "Because the entry describes a fact, it does not express a positive or negative attitude, emotion or evaluation."}, "language_style": {"answer": "Tactful and warm", "rationale": "The entry is more euphemistic, friendly and friendly. It does not use hard or command language, but rather directs the respondents to answer questions in an invitation. At the same time, the description \"walking about 100 metres\" is also relatively mild, without too much emotional color, giving people a comfortable feeling."}, "time_dimension": {"answer": "Present", "rationale": "It can be judged by the phrase \"walking\" and \"about 100 metres' distance\" as a description of current behaviour or state, and therefore the temporal dimension is now."}, "sentence_type": "Imperative sentence", "pos": {"nouns": {"Walk": 1, "Road": 1, "Rice": 1}, "verbs": {"About": 1}, "adverbs": {"100": 1}, "numerals": {"100": 1}, "classifiers": {"Rice": 1}}}, {"content": "12. Take a bath, dress yourself", "word_count": 6, "label": "", "sentence_structure": "Subject鈥搗erb鈥揷omplement", "subjective_objective": {"answer": "Objective", "rationale": "Because \"self-washing, dressing\" is a practical act, it does not require the patient ' s subjective will or feelings to explain its occurrence. It is an objective fact, and the answer is therefore objective."}, "sentence_sentiment": {"answer": "Neutral", "rationale": "Because the entry describes only one act and does not contain any emotional colour."}, "language_style": {"answer": "Calm and direct", "rationale": "The language style of the entry is relatively simple and not too much rhetorical or emotional. It simply asks the respondents whether they are capable of accomplishing these basic life skills independently."}, "time_dimension": {"answer": "Present", "rationale": "\"Show yourself, dress\" describes the behaviour or habits that the person tested is doing, and the temporal dimension is now judged."}, "sentence_type": "Imperative sentence", "pos": {"nouns": {"self": 1, "Bath": 1, "Clothes": 1}, "verbs": {"Wash": 1, "Put it on": 1}, "numerals": {"12": 1}}}, {"content": "13. Reduction of time for work or other activities", "word_count": 13, "label": "", "sentence_structure": "Subject鈥搗erb鈥揷omplement", "subjective_objective": {"answer": "Subjective", "rationale": "Because reducing the time spent working or operating is an individual ' s subjective judgement and decision."}, "sentence_sentiment": {"answer": "Negative", "rationale": "The attitude of this entry is negative, as reducing the time spent on work or other activities may lead to reduced work efficiency and quality of life."}, "language_style": {"answer": "Calm and direct", "rationale": "This is a more direct, concise language style, which does not have too many rhetorical or emotional features, but is based on facts and asks whether the other side has reduced the time spent working or other activities. This is a cold and direct language style that helps to obtain more objective and accurate data."}, "time_dimension": {"answer": "Past", "rationale": ""}, "sentence_type": "Imperative sentence", "pos": {"nouns": {"Work": 1, "Time": 1}, "verbs": {"Reduction": 1, "Other": 1}, "numerals": {"13": 1}}}, {"content": "14. The only thing that could have been done was to do something that was a little bit complete.", "word_count": 15, "label": "", "sentence_structure": "Subject鈥搗erb鈥揷omplement", "subjective_objective": {"answer": "Subjective", "rationale": "Because \"want\" and \"can only be done\" are part of the patient's subjective feelings and assessments."}, "sentence_sentiment": {"answer": "Negative", "rationale": "Because the \"only part of the\" act in the entry is negative, it means that the desired goal has not been achieved."}, "language_style": {"answer": "Tactful and warm", "rationale": "The entry uses a more euphemistic and implicit language style, which is not too direct or commandive, but rather a more moderate and inviting approach to dialogue. Such expression is more personal and interactive, which makes the respondents more comfortable and comfortable and helps to obtain more realistic and accurate data and feedback."}, "time_dimension": {"answer": "Present", "rationale": "The keyword \"what was meant to be done was only partially completed\" indicates that it is a description of the current situation and therefore the temporal dimension is now judged."}, "sentence_type": "Imperative sentence", "pos": {"nouns": {"Things": 1, "Part": 1}, "verbs": {"I want to do it": 1, "Only": 1, "Completed": 1}, "adjectives": {"Ben": 1}, "adverbs": {"It was": 1, "Only": 2}, "numerals": {"14": 1}}}, {"content": "15. Limitations on the types of work or activities that are intended to be performed", "word_count": 16, "label": "", "sentence_structure": "Subject鈥搗erb鈥搊bject", "subjective_objective": {"answer": "Subjective", "rationale": "Since the purpose of the questionnaire was to provide information on the patient ' s knowledge and feelings of his or her situation, the patient was asked whether he or she believed that he or she was restricted and therefore was subject to subjective examination."}, "sentence_sentiment": {"answer": "Negative", "rationale": "Because the expression \"restricted\" in the entry is negative, implying that the freedom of the individual is being obstructed."}, "language_style": {"answer": "Calm and direct", "rationale": "The article is more straightforward, with no excessive emotional and rhetorical style. It asks whether the interviewee is restricted in choosing a job or activity and expresses it in a very clear manner. This type of questionnaire language is usually used to obtain specific information or data, rather than to establish emotional connections or in-depth dialogue."}, "time_dimension": {"answer": "Past", "rationale": "The entry \"type of work or activity desired\" refers to past wishes or expectations, and \"restricted\" indicates that these aspirations have not been fulfilled, and therefore the temporal dimension is a matter of the past."}, "sentence_type": "Negative declarative sentence", "pos": {"nouns": {"Work": 1, "Activities": 1, "Category": 1}, "verbs": {"Yes": 1, "Present": 1, "Limit": 1, "Production": 1}, "adverbs": {"Yes": 1, "of": 1, "or": 1}, "numerals": {"15": 1}, "classifiers": {"Plant": 1}, "pronouns": {"Here": 1}}}, {"content": "16. Difficulties in completing work or other activities (e.g., additional requirements)", "word_count": 19, "label": "", "sentence_structure": "Subject鈥搗erb鈥揷omplement", "subjective_objective": {"answer": "Subjective", "rationale": "Since the subject is concerned with the feelings and experiences of patients who experience difficulties in performing their work or activities, it is the patient ' s subjective feeling."}, "sentence_sentiment": {"answer": "Negative", "rationale": "Because the descriptions of \"difficulties\" and \"need additionality\" in the entries are negative."}, "language_style": {"answer": "Tactful and warm", "rationale": ""}, "time_dimension": {"answer": "Present", "rationale": "The entry is for the purpose of asking the testee whether there are difficulties in completing the work or other activities, and the keywords \"complete\", \"difficult\" and \"need additional\" point to the act or state of conduct that is being performed, so the temporal dimension is now judged."}, "sentence_type": "Affirmative declarative sentence", "pos": {"nouns": {"Work": 1, "Activities": 1, "Difficulties": 1}, "verbs": {"Completed": 1, "Yes": 1}, "adjectives": {"Extra": 1}}}, {"content": "17. Reduction in time for work or other activities", "word_count": 13, "label": "", "sentence_structure": "Subject鈥搗erb鈥揷omplement", "subjective_objective": {"answer": "Objective", "rationale": "Since reducing the time spent working or working is an objective fact of the patient ' s experience, it does not involve subjective attitudes or feelings of the patient."}, "sentence_sentiment": {"answer": "Negative", "rationale": "Because it is negative to reduce the time spent on work or other activities."}, "language_style": {"answer": "Calm and direct", "rationale": "The language style of the entry is more calm and straightforward, and does not involve too much rhetorical or emotional rendering, but rather leaves the question to the interviewee to fill out his/her own answers."}, "time_dimension": {"answer": "Past", "rationale": "The entry is for the testee ' s behaviour or state before a certain point in time, and the time dimension is judged to be past."}, "sentence_type": "Negative declarative sentence", "pos": {"nouns": {"Work": 1, "Time": 2, "Activities": 1}, "verbs": {"Reduction": 1}, "numerals": {"17": 1}}}, {"content": "The only thing that would have been done was to do it in part.", "word_count": 15, "label": "", "sentence_structure": "Subject鈥搗erb鈥揷omplement", "subjective_objective": {"answer": "Subjective", "rationale": "Because \"a desire\" implied the patient's will or expectations, which was a subjective perspective."}, "sentence_sentiment": {"answer": "Negative", "rationale": "Because the result of \"only part of the work\" is negative, implying that the desired goal cannot be achieved."}, "language_style": {"answer": "Tactful and warm", "rationale": "The entry is more adept and interactive when it is spoken in a gentle and inviting manner, rather than by direct statements or orders."}, "time_dimension": {"answer": "Present", "rationale": "In this entry, \"already\" indicates a plan or expectation of the past, and \"only part of the completion\" describes the actual results of the present, so the temporal dimension is now."}, "sentence_type": "Interrogative sentence", "pos": {"nouns": {"Things": 1, "Part": 1}, "verbs": {"It was": 1, "Yes": 1, "Do it": 1, "Completed": 1}, "adverbs": {"Only": 1}, "numerals": {"18": 1}, "pronouns": {"you": 1}}}, {"content": "19. Work or other activities can be less careful than normal.", "word_count": 17, "label": "", "sentence_structure": "Subject鈥揷opula鈥損redicative", "subjective_objective": {"answer": "Subjective", "rationale": "Because the examination is about the patient ' s assessment and perception of his performance in the performance of his work or other activities, it involves subjective attitudes and emotional experiences."}, "sentence_sentiment": {"answer": "Negative", "rationale": "Because the \"not as careful as usual\" act in the entry is negative."}, "language_style": {"answer": "Calm and direct", "rationale": "The entry is more calm and straightforward in its language, without too much rhetoric or emotional colour. It is simple and clear to ask whether the person surveyed is as careful as he or she is when doing his or her work or other activities, and this expression is more objective and true."}, "time_dimension": {"answer": "Present", "rationale": ""}, "sentence_type": "Negative declarative sentence", "pos": {"nouns": {"Work": 1, "Activities": 1, "Normal": 1}, "verbs": {"Do it": 1, "Like": 1, "Yes": 1, "not": 1, "Careful": 1}, "adjectives": {"Other": 1}, "numerals": {"19": 1}, "pronouns": {"you": 1}}}, {"content": "20. To what extent has your health or emotional infirmity affected your normal social activities with your family, friends, neighbours or groups over the past four weeks?", "word_count": 46, "label": "", "sentence_structure": "Subject鈥搗erb鈥搊bject", "subjective_objective": {"answer": "Subjective", "rationale": "Because the problem is one of the impact of the patient ' s own health or emotional state on social activities, self-awareness and subjective evaluation of the patient is required."}, "sentence_sentiment": {"answer": "Negative", "rationale": "Because the entries describe the effects of bad health or emotions on social activities, this is usually negative."}, "language_style": {"answer": "Tactful and warm", "rationale": "The language style of the entry is euphemism. It uses some polite and respectful language to ask the interviewee about, for example, \"the extent to which it affects\" rather than \"the effect or not.\" Moreover, this description avoids problematic language that may cause discomfort or resentment."}, "time_dimension": {"answer": "Past", "rationale": "The entry is for the purpose of questioning the behaviour and feelings of the subjects over the past four weeks and therefore for the purposes of judging the past."}, "sentence_type": "Interrogative sentence", "pos": {"nouns": {"Past": 1, "Four weeks": 1, "Health": 1, "Emotion": 1, "Level": 1, "Impact": 1, "Family": 1, "friends": 1, "Neighbors": 1, "Collective": 1, "Social events": 1}, "verbs": {"in": 2, "Lee": 1, "It's affecting": 1, "and": 1}, "adjectives": {"Normal": 1}, "numerals": {"Four": 1}, "pronouns": {"Yours": 1, "or": 1, "of": 3, "you": 1}}}, {"content": "21. Have you had any physical pain in the last four weeks?", "word_count": 17, "label": "", "sentence_structure": "Subject鈥損redicate", "subjective_objective": {"answer": "Objective", "rationale": "Because \"the physical pain of the last four weeks\" is an objective fact, it does not require subjective perceptions of the patient."}, "sentence_sentiment": {"answer": "Negative", "rationale": "Because the entry asks for physical pain, it is a negative experience or state."}, "language_style": {"answer": "Calm and direct", "rationale": "The entry asks the other party in a straightforward manner whether the physical pain has occurred in the past four weeks, is more objective and calm, and does not use excessive rhetorical or emotional colours."}, "time_dimension": {"answer": "Past", "rationale": "The entry is for the testee's physical condition over the last four weeks and can therefore be judged as \"the past\"."}, "sentence_type": "Interrogative sentence", "pos": {"nouns": {"Past": 1, "Four weeks": 1, "Physically": 1, "Pain": 1}, "verbs": {"have": 1, "Come on": 1}, "numerals": {"Four": 1}, "classifiers": {"individual": 1}, "pronouns": {"you": 1}}}, {"content": "22. Has physical pain affected your normal work (including work and domestic activities) during the past four weeks?", "word_count": 34, "label": "", "sentence_structure": "Subject鈥搗erb鈥搊bject", "subjective_objective": {"answer": "Objective", "rationale": "Since the question is whether the patient ' s physical pain has affected his normal work during the last four weeks, it is an objective fact-finding exercise, independent of the patient ' s subjective ideas."}, "sentence_sentiment": {"answer": "Negative", "rationale": "Because the act of \"physical pain affects your normal work\" in the entry is negative."}, "language_style": {"answer": "Calm and direct", "rationale": "The language style of the entry is more calm and straightforward, with no excessive emotional or rhetorical style. It opens the door to questions about whether physical pain has affected normal work and household activity in the last four weeks."}, "time_dimension": {"answer": "Past", "rationale": ""}, "sentence_type": "Interrogative sentence", "pos": {"nouns": {"Past": 1, "Four weeks": 1, "Body": 1, "Pain": 1, "Work": 3, "Including": 1, "Domestic work": 1, "Activities": 1}, "verbs": {"Impact": 1, "Do it": 1}, "adverbs": {"Normal": 1}, "numerals": {"Four": 1}, "classifiers": {"individual": 1}, "pronouns": {"you": 1}}}, {"content": "Do you feel that life is full?", "word_count": 8, "label": "", "sentence_structure": "Subject鈥揷opula鈥損redicative", "subjective_objective": {"answer": "Subjective", "rationale": "Since the subject is asked about the perceived and subjective evaluation of the trial, rather than the objective facts or behaviour, it is subjective in its perspective."}, "sentence_sentiment": {"answer": "Neutral", "rationale": "Because the entry does not have any emotional color."}, "language_style": {"answer": "Tactful and warm", "rationale": "The entry uses a soft and encouraging question-and-answer approach, by asking the other side about how they feel about their lives, and by leading the interviewees to think and share their views, which is more personal and interactive. At the same time, this type of questionnaire makes it easier for the interviewees to feel comfortable and free, and thus more willing to share real ideas."}, "time_dimension": {"answer": "Present", "rationale": ""}, "sentence_type": "Interrogative sentence", "pos": {"nouns": {"Questionnaire": 1, "Entry": 1, "Life": 1, "Fill": 1}, "verbs": {"I think": 1}, "adverbs": {"you": 1}, "numerals": {"23锛?: 1}}}, {"content": "Are you a nervous person?", "word_count": 11, "label": "", "sentence_structure": "Subject鈥揷opula鈥損redicative", "subjective_objective": {"answer": "Subjective", "rationale": "Because stress is a subjective feeling and experience, different people may have different levels and manifestations of stress."}, "sentence_sentiment": {"answer": "Negative", "rationale": "Because the term \"sense\" is usually considered negative quality or state."}, "language_style": {"answer": "Calm and direct", "rationale": "It is more objective and balanced. It asks whether the other party is a nervous person, without using euphemism or a qualifier, with some degree of directness and clarity."}, "time_dimension": {"answer": "Present", "rationale": "The keywords are \"Are you\" and \"A man of stress,\" which describe the person's current state or character, so the temporal dimension of the article is now judged."}, "sentence_type": "Interrogative sentence", "pos": {"nouns": {"person": 1, "Spirit": 1}, "verbs": {"be": 1, "Nervous": 1}, "adjectives": {"He's nervous": 1}, "numerals": {"24锛?: 1}}}, {"content": "25. Are you feeling depressed that nothing will cheer you up?", "word_count": 20, "label": "", "sentence_structure": "Subject鈥損redicate", "subjective_objective": {"answer": "Subjective", "rationale": "Because the problem is examined by the patient ' s own emotional and mental changes, it is the patient ' s subjective experience."}, "sentence_sentiment": {"answer": "Negative", "rationale": "Because both \"sliding\" and \"not cheering you up\" have negative emotional and negative connotations."}, "language_style": {"answer": "Tactful and warm", "rationale": "The words \"showing down\" and \"stammering up\" are used to describe emotional state and behaviour, giving people a sense of affection and caring. And \"Does nothing cheer you up when you feel depressed?\" This way of asking is even more gentle and respectful of the feelings of the interviewees."}, "time_dimension": {"answer": "Present", "rationale": "The time dimension of the article is therefore the present."}, "sentence_type": "Interrogative sentence", "pos": {"nouns": {"you": 1, "I'm so upset": 1, "Yes": 1}, "verbs": {"I feel": 1, "鈾?And I'm gonna be 鈾?: 1, "Come on, get up": 1, "Come on": 1}, "adjectives": {"No, I can't": 1}, "adverbs": {"What": 1, "Both": 1, "- You're not": 1}, "numerals": {"25": 1}}}, {"content": "Do you feel calm?", "word_count": 6, "label": "", "sentence_structure": "Subject鈥損redicate", "subjective_objective": {"answer": "Subjective", "rationale": "Because \"feel\" is the patient's subjective feeling."}, "sentence_sentiment": {"answer": "Neutral", "rationale": "Because the entry does not have any emotional color."}, "language_style": {"answer": "Tactful and warm", "rationale": "The entry expresses the theme \"quiet\" in question, does not use the tone of direct statements or orders, is more cordial and interactive and is suitable for use in psychological questionnaires."}, "time_dimension": {"answer": "Present", "rationale": "The entry asks the person who was tested about his current emotional state, does not address past or future behaviour or mental state, and therefore the judgement is now."}, "sentence_type": "Interrogative sentence", "pos": {"nouns": {"Calm down": 1}, "verbs": {"I think": 1}, "adverbs": {"- You're not": 1}, "numerals": {"26": 1}}}, {"content": "Are you energetic?", "word_count": 6, "label": "", "sentence_structure": "Subject鈥揷opula鈥損redicative", "subjective_objective": {"answer": "Subjective", "rationale": "Because \"absorbed\" is the patient's subjective assessment of his or her physical and mental condition, not an objective fact or result."}, "sentence_sentiment": {"answer": "Positive", "rationale": "Because \"energy\" is a positive term for the term, which means that individuals have enough energy to carry out their daily activities and to meet challenges."}, "language_style": {"answer": "Tactful and warm", "rationale": ""}, "time_dimension": {"answer": "Present", "rationale": "The entry asks the testee's current state and feelings, \"Are you energetic?\" is a current-day question, so it's \"now.\""}, "sentence_type": "Interrogative sentence", "pos": {"nouns": {"- The energy": 1, "鈾?Full of 鈾?: 1}, "verbs": {"you": 1, "I feel": 1}, "adjectives": {"鈾?Full of 鈾?: 1}, "numerals": {"27": 1}, "pronouns": {"you": 1}}}, {"content": "Are you depressed?", "word_count": 7, "label": "", "sentence_structure": "Subject鈥損redicate", "subjective_objective": {"answer": "Subjective", "rationale": "Because \"felter\" is the expression of what the patient feels inside, and it's subjective."}, "sentence_sentiment": {"answer": "Negative", "rationale": "Because \"frightfully low\" is a negative emotional experience."}, "language_style": {"answer": "Calm and direct", "rationale": "The entry directly asks the respondents in a concise language about their current emotional state, without excessive ado or emotional rendering, and with a more calm and direct language style."}, "time_dimension": {"answer": "Present", "rationale": "The entry is for the testee ' s current emotional state, and the time dimension is now judged."}, "sentence_type": "Interrogative sentence", "pos": {"nouns": {"Emotion": 1, "Down": 1, "- You're not": 1}, "verbs": {"you": 1, "be": 1}, "numerals": {"28": 1}}}, {"content": "Are you exhausted?", "word_count": 8, "label": "", "sentence_structure": "Subject鈥搗erb鈥搊bject", "subjective_objective": {"answer": "Subjective", "rationale": "Because fatigue is a subjective feeling."}, "sentence_sentiment": {"answer": "Negative", "rationale": "Because the expression \"tired\" in the entry is negative."}, "language_style": {"answer": "Tactful and warm", "rationale": ""}, "time_dimension": {"answer": "Present", "rationale": "The entry asks if the testee's current state of feeling, i.e., \"tired\" is present, so the time dimension is now judged."}, "sentence_type": "Interrogative sentence", "pos": {"nouns": {"I'm exhausted": 1}, "verbs": {"I think": 1}, "numerals": {"29": 1}}}, {"content": "Are you a happy man?", "word_count": 8, "label": "", "sentence_structure": "Subject鈥揷opula鈥損redicative", "subjective_objective": {"answer": "Subjective", "rationale": "Because \"happy\" is a subjective feeling and attitude."}, "sentence_sentiment": {"answer": "Positive", "rationale": "Because \"happy\" is a positive word, expressing positive emotional attitudes."}, "language_style": {"answer": "Tactful and warm", "rationale": "The entry is spoken in a question-and-answer tone and is more friendly and respectful by \"you\"; and \"Happy person?\" is a more moderate expression, with no direct demand or order. So the language style of the questionnaire is euphemism."}, "time_dimension": {"answer": "Present", "rationale": "The key word is \"Are you a happy person,\" which is an inquiry into the current state of the subject, so the temporal dimension is now judged."}, "sentence_type": "Interrogative sentence", "pos": {"nouns": {"person": 1, "Questionnaire": 1, "Entry": 1, "Happy": 1}, "verbs": {"be": 1, "Here": 1, "Analysis": 1}, "adjectives": {"individual": 1, "Happy": 1}, "numerals": {"30锛?: 1}}}, {"content": "Are you tired?", "word_count": 6, "label": "", "sentence_structure": "Subject鈥損redicate", "subjective_objective": {"answer": "Subjective", "rationale": "Because \"feel fatigue\" is a subjective feeling or experience, a perception and evaluation of the interviewees themselves, not an objective fact."}, "sentence_sentiment": {"answer": "Negative", "rationale": "Because fatigue is a negative physical condition."}, "language_style": {"answer": "Tactful and warm", "rationale": "The entry is more personal and interactive when it is spoken in a gentle, inquisitive manner rather than directly speaking or ordering."}, "time_dimension": {"answer": "Present", "rationale": ""}, "sentence_type": "Interrogative sentence", "pos": {"nouns": {"Tired": 1}, "verbs": {"Feel": 1, "Tired": 1}, "numerals": {"31": 1}}}, {"content": "32. Do your health restrict your social activities (e.g., visiting friends and friends)?", "word_count": 19, "label": "", "sentence_structure": "Subject鈥搗erb鈥搊bject", "subjective_objective": {"answer": "Subjective", "rationale": "Because the subject is primarily concerned with patients鈥?subjective feelings and perceptions that they believe their health condition affects their social activities. Although health status is an objective fact, patients鈥?feelings and assessments of it are subjective."}, "sentence_sentiment": {"answer": "Negative", "rationale": "Because the description \"health limits social activities\" is negative and indicates that individuals are socially hindered and restricted."}, "language_style": {"answer": "Tactful and warm", "rationale": "This expression is more acceptable to the respondents and is willing to answer questions, and more conducive to obtaining real feedback."}, "time_dimension": {"answer": "Past", "rationale": ""}, "sentence_type": "Interrogative sentence", "pos": {"nouns": {"Health": 1, "Limits": 1, "Social events": 1, "Go visit your friends": 1}, "verbs": {"Limits": 1, "Yes": 1, "Impact": 1}, "adjectives": {"Yours": 1, "32锛?: 1}, "numerals": {"32锛?: 1}, "pronouns": {"you": 1}}}, {"content": "I'm more vulnerable to illness than anyone.", "word_count": 9, "label": "", "sentence_structure": "Subject鈥揷opula鈥損redicative", "subjective_objective": {"answer": "Subjective", "rationale": "Because \"predisposed\" is the patient's subjective feeling and perception, not the objective fact or behaviour."}, "sentence_sentiment": {"answer": "Negative", "rationale": "Because the \"predisposition\" status in the entry is negative."}, "language_style": {"answer": "Tactful and warm", "rationale": "The entry uses the first person's pronoun, \"I\", which emphasizes the feelings and experiences of the person and compares them with others in a metaphorical way. At the same time, there is a sense of self-taste and impatience in the expression, which makes the questionnaire more friendly and natural and helps to increase the participation of the respondents."}, "time_dimension": {"answer": "Present", "rationale": "The entry is for the present physical condition and experience of the person being tested, and the time dimension is now judged."}, "sentence_type": "Interrogative sentence", "pos": {"nouns": {"I": 1, "person": 1, "Diseases": 1}, "verbs": {"Elephant": 1, "- Yeah": 1, "Fan": 1}, "adjectives": {"Easy": 1}, "adverbs": {"It looks like": 1}, "numerals": {"33": 1}}}, {"content": "I'm as healthy as anyone I know.", "word_count": 11, "label": "", "sentence_structure": "Subject鈥揷opula鈥損redicative", "subjective_objective": {"answer": "Objective", "rationale": "Because \"I'm as healthy as anyone I know\" is a factual statement, which involves comparisons and observations of individual health status, and therefore an objective view."}, "sentence_sentiment": {"answer": "Neutral", "rationale": "Because the entry does not have any emotional color."}, "language_style": {"answer": "Calm and direct", "rationale": "The entry presents in a more objective way whether the subjects are in the same health condition as the persons they know, and is not too much rhetorical or emotional, so that the language style is straightforward."}, "time_dimension": {"answer": "Present", "rationale": "The keyword \"I\" and \"I'm as healthy as anyone I know\" suggests that it's a description of the state of the moment, and therefore of the time dimension of the moment."}, "sentence_type": "Interrogative sentence", "pos": {"nouns": {"person": 1, "Health": 1}, "verbs": {"Call": 1, "Yes": 1, "Same thing": 1}, "adverbs": {"very": 1}, "numerals": {"34": 1}, "pronouns": {"I": 1}}}, {"content": "I think my health is getting worse.", "word_count": 12, "label": "", "sentence_structure": "Subject鈥揷opula鈥損redicative", "subjective_objective": {"answer": "Subjective", "rationale": "This is seen from the patient鈥檚 subjective perspective."}, "sentence_sentiment": {"answer": "Negative", "rationale": "Because the entry \"Assumpturing your health is bad\" is a negative emotion and attitude."}, "language_style": {"answer": "Calm and direct", "rationale": "The entry uses direct statement language to ask the interviewees about their subjective views of their health, without too much rhetoric or emotional rendering, and with a fair and objective tone."}, "time_dimension": {"answer": "Past", "rationale": ""}, "sentence_type": "Negative declarative sentence", "pos": {"nouns": {"Health status": 1, "Bad": 1}, "verbs": {"I think": 1}, "numerals": {"35": 1}, "pronouns": {"I": 1}}}, {"content": "I'm in a very good health condition.", "word_count": 9, "label": "", "sentence_structure": "Subject鈥揷opula鈥損redicative", "subjective_objective": {"answer": "Subjective", "rationale": "Because \"very good health\" is the patient's subjective opinion and feeling."}, "sentence_sentiment": {"answer": "Positive", "rationale": "Because the expression \"very healthy\" is positive, which means that the individual is in good physical condition."}, "language_style": {"answer": "Tactful and warm", "rationale": "The language style of the entry is more euphemistic, not overly emphatic or exaggerated, but rather a more peaceful way of expressing the concerns and queries of the interviewee. In addition, the sentence uses a positive presentation, giving a positive impression and contributing to a good investigation atmosphere."}, "time_dimension": {"answer": "Present", "rationale": "The words \"very\" and \"good\" in the sentence make it clear that the entry is describing the current state, so the answer is now."}, "sentence_type": "Affirmative declarative sentence", "pos": {"nouns": {"Health status": 1, "Very": 1}, "verbs": {"be": 1}, "adjectives": {"Okay": 1}}}]}

{"questionnaire_title": "Diagnosis of social resilience", "questionnaire_category": "Behavioral questionnaire", "item_count": 20, "emotion_shift_overall": "From negative to positive", "language_style_overall": "Tactful and warm", "items": [{"content": "I will never mention one of my failures, lest someone capture his weakness.", "word_count": 27, "label": "", "sentence_structure": "Subject鈥搗erb鈥搊bject", "subjective_objective": {"answer": "Subjective", "rationale": "Fear of being caught by others is a subjective feeling and attitude."}, "sentence_sentiment": {"answer": "Negative", "rationale": "Because the actions in the entries \"never mention\" and the purpose \"to be caught by others\" are negative."}, "language_style": {"answer": "Calm and direct", "rationale": "The entry is based on the following: 1. The entry is expressed directly through a clear expression of the subject - the failure of the person concerned is avoided, thus preventing others from drawing on their weaknesses. 2. The description is not based on a rhetorical technique or excessive emotional accommodations, which are more objective and show a calm attitude. It is therefore possible to judge the language style of the entry in the questionnaire as being straightforward."}, "time_dimension": {"answer": "Present", "rationale": "The entry describes the person who was tested, in his current behavior or mentality, in his own defence strategy in the face of failure. The keyword \"never\" or \"fear of being caught\" indicates that this is the current behavioral tendency.\""}, "sentence_type": "Negative declarative sentence", "pos": {"nouns": {"self": 2, "One failed": 1, "person": 1, "Weaknesses": 1}, "verbs": {"to": 1, "Absolutely not": 1, "T": 1, "Yes": 1, "They're getting caught": 1}, "adverbs": {"I'm afraid": 1, "Absolutely": 1}, "classifiers": {"Number of times": 1}, "pronouns": {"self": 2, "others": 1}}}, {"content": "2. Every time I go to a new place, I'm easily accessible.", "word_count": 17, "label": "", "sentence_structure": "Subject鈥搗erb鈥搊bject", "subjective_objective": {"answer": "Subjective", "rationale": "Because this is a subjective examination of the patients ' own feelings and perceptions."}, "sentence_sentiment": {"answer": "Positive", "rationale": "Because the \"easy to approach\" act in the entry is positive, indicating that this person is good at communicating and interacting with people."}, "language_style": {"answer": "Tactful and warm", "rationale": "The language style of the entry is more euphemistic and encouraging, and instead of asking questions or orders directly, it is more friendly and intimate to guide the other to share their feelings."}, "time_dimension": {"answer": "Present", "rationale": "Now the entry describes the behaviour and emotional state of the person being tested in the current situation, \"every new place\" indicating that it is a description of the current situation, and \"I am easily accessible\" suggesting that it is a customary, constant expression of emotion. Therefore, the entry should be classified as now."}, "sentence_type": "Affirmative declarative sentence", "pos": {"nouns": {"Local": 1, "person": 1}, "verbs": {"Close": 1}, "adjectives": {"New": 1, "Easy": 1}, "adverbs": {"very": 1}, "pronouns": {"I": 1}}}, {"content": "3. I am often left without words in front of strangers, and I am embarrassed.", "word_count": 18, "label": "", "sentence_structure": "Subject鈥揷opula鈥損redicative", "subjective_objective": {"answer": "Subjective", "rationale": "Because the feeling of \"always saying\" and \"inconvenient\" is the patient's subjective experience."}, "sentence_sentiment": {"answer": "Negative", "rationale": "Because the entry \"I'm embarrassed to say nothing in front of strangers\" is negative."}, "language_style": {"answer": "Tactful and warm", "rationale": ""}, "time_dimension": {"answer": "Present", "rationale": "I've never said anything about \"normal\" being a regular, customary act, \"no words\" and \"unusual\" being present, so this entry describes the person being tested for the act or the mental state."}, "sentence_type": "Affirmative declarative sentence", "pos": {"nouns": {"Stranger": 1, "In front of you": 1, "Awkward": 1}, "verbs": {"Regular": 1, "Nothing to say": 1, "I feel": 1}, "adjectives": {"Awkward": 1}, "adverbs": {"Regular": 1}}}, {"content": "When I choose my clothes, I want to follow the tide and hope it will suit myself.", "word_count": 19, "label": "", "sentence_structure": "Subject鈥搗erb鈥搊bject", "subjective_objective": {"answer": "Subjective", "rationale": "Because \"want to follow the tide\" and \"favorite to fit himself\" are patients' subjective attitudes and expectations."}, "sentence_sentiment": {"answer": "Positive", "rationale": "Because it expresses the desire of people to be fit for their image and suit their clothes, it is a positive quality and attitude."}, "language_style": {"answer": "Tactful and warm", "rationale": "The language style of the entry is more euphemistic and warm. First, the opening phrase \"when I choose my clothes\" creates a comfortable, friendly feeling for subsequent expression. Second, the phrase \"will want to follow the tide, wish to fit myself\" is not a direct order or instruction, but rather a question about the preferences and needs of the other, which makes it more acceptable to fill out and to be respected and understood."}, "time_dimension": {"answer": "Present", "rationale": "The keyword \"will\" indicates that it is a description of the current behavior, that the words \"when choosing clothes\", \"wanting to follow the tide\" and \"want to fit himself\" point to the current state and orientation. So the entry describes the current behaviour and mindset of the subject."}, "sentence_type": "Affirmative declarative sentence", "pos": {"nouns": {"I": 1, "Clothes": 1, "The tide": 1}, "verbs": {"Choose": 1, "Yes": 1, "Call": 1, "Whatever": 1, "Fit": 1}, "adjectives": {"Tide": 1, "self": 1}, "adverbs": {"EMPTY": 2, "When": 1}, "numerals": {"4锛?: 1}}}, {"content": "5. Every time I go to a new place, I always sleep poorly on the first day, at home, and when I have to change the bed, I sometimes get insomnia.", "word_count": 33, "label": "", "sentence_structure": "Subject鈥搗erb鈥搊bject", "subjective_objective": {"answer": "Subjective", "rationale": "Because it involves the patient's own feelings and experiences, i.e., being in a new environment, which leads to sleep problems.\""}, "sentence_sentiment": {"answer": "Negative", "rationale": "Because the \"sleeping bad\" and \"sleep insomnia\" cases described in the entries are negative."}, "language_style": {"answer": "Calm and direct", "rationale": "The entry raises the question in a direct and clear manner, i.e., insomnia in the new environment, with no excessive rhetorical or emotional rendering, and with a more objective tone."}, "time_dimension": {"answer": "Present", "rationale": "Now the entry \"always\" and \"sometimes\" indicates that it is a customary act and may be continuing. So, the time dimension is now."}, "sentence_type": "Affirmative declarative sentence", "pos": {"nouns": {"Local": 2, "New": 1, "The bed": 2, "Home": 1, "Chang": 1, "Insomnia": 1}, "verbs": {"Present": 1, "Sleep": 2, "Switch": 1, "Yes": 1, "Always": 1}, "adjectives": {"New": 1, "Okay": 1, "Sometimes": 1}, "adverbs": {"Every": 1, "Both": 1, "Just": 1}, "numerals": {"5": 1, "One": 2, "Two": 1, "Chang": 1}, "pronouns": {"I": 2}}}, {"content": "6. I have actively participated in social practice to build working experience.", "word_count": 16, "label": "", "sentence_structure": "Subject鈥搗erb鈥揷omplement", "subjective_objective": {"answer": "Subjective", "rationale": "Because motivation, willingness to participate and the accumulation of practical experience are subjective attitudes and behaviour of patients."}, "sentence_sentiment": {"answer": "Positive", "rationale": "The entry \"Enjoy active social practice to build work experience\" expresses a positive attitude and behaviour."}, "language_style": {"answer": "Tactful and warm", "rationale": "The language style of the entry is more euphemistic, with an encouraging tone, with respondents feeling respected and supported, more willing to participate in the questionnaire, and with no direct orders or criticism, which can increase the motivation and cooperation of the interviewees."}, "time_dimension": {"answer": "Present", "rationale": "The words \"active participation\" and \"accumulation of work experience\" in this entry indicate that this is an ongoing act or state and therefore is judged to be the present."}, "sentence_type": "Affirmative declarative sentence", "pos": {"nouns": {"Social practice": 1, "Work experience": 1}, "verbs": {"Participation": 1, "Accumulation": 1}, "adjectives": {"Positive": 1}, "pronouns": {"I": 1}}}, {"content": "The more crowded I am, the more nervous I am.", "word_count": 13, "label": "", "sentence_structure": "Subject鈥搗erb鈥揷omplement", "subjective_objective": {"answer": "Subjective", "rationale": "Because feeling nervous is a subjective feeling."}, "sentence_sentiment": {"answer": "Negative", "rationale": "The basis of judgment. Because the act of \"stretching\" is negative."}, "language_style": {"answer": "Calm and direct", "rationale": "The entry is simple and straightforward in asking the other side how it feels in the crowd, without too many rhetorical or emotional renderings, and is more objective in tone."}, "time_dimension": {"answer": "Present", "rationale": "Based on the phrase \"the more...\" the more the structure and the time-style \"now\" it can be inferred that the entry describes the current feelings and experiences of the person being tested and thus the present."}, "sentence_type": "Affirmative declarative sentence", "pos": {"nouns": {"Local": 1, "Nervous": 1}, "verbs": {"be": 1, "I feel": 1, "There's a lot of people": 1}, "adjectives": {"More": 1}, "adverbs": {"The more you are, the more you are": 1, "Tsk": 2}, "pronouns": {"I": 1}}}, {"content": "8. In formal competitions or examinations, I will do more than I do in practice.", "word_count": 23, "label": "", "sentence_structure": "Subject鈥搗erb鈥揷omplement", "subjective_objective": {"answer": "Subjective", "rationale": "Because this is a subjective assessment and expectation of the patient, not a description based on objective facts. While in some cases it may be better to perform in a formal competition, this is not the rule in all cases, and the view is therefore somewhat subjective."}, "sentence_sentiment": {"answer": "Positive", "rationale": "Because the entries express a positive attitude and self-confidence that can be better demonstrated in important contexts."}, "language_style": {"answer": "Tactful and warm", "rationale": "The entry leads the respondents to share their experiences in a question-and-answer tone and uses a comparative format to make it easier for the respondents to express their views. At the same time, the use of the term \"mostly not\" makes the language more euphemism and moderation, reducing the pressure and discomfort on the respondents."}, "time_dimension": {"answer": "Present", "rationale": "The entry expresses a customary behaviour of the person being tested, i.e., that the performance at the official competition or examination is usually similar to or better than normal practice. This behaviour is now being carried out, and can therefore be judged as being present."}, "sentence_type": "Affirmative declarative sentence", "pos": {"nouns": {"The game": 1, "Examinations": 1, "Achievements": 1, "Practice": 1}, "verbs": {"Yes": 1, "- Yeah": 2, "Bad": 1}, "adjectives": {"Formal": 1, "Normal": 1}, "adverbs": {"I don't think so": 1}}}, {"content": "9. I must speak before the public and I will be overwhelmed and incompetent by fear.", "word_count": 28, "label": "", "sentence_structure": "Subject鈥搗erb鈥揷omplement", "subjective_objective": {"answer": "Subjective", "rationale": "This is a subjective prediction or concern of the patient about the possible reaction of the patient in a given situation."}, "sentence_sentiment": {"answer": "Negative", "rationale": "Because the description of \"weakness, distraught and incomprehensible\" in the entry is negative, it shows that the person in question has a degree of psychological stress and tension in the face of a wide public."}, "language_style": {"answer": "Tactful and warm", "rationale": "The entry is more euphemistic and implicit. It does not directly express the question of whether the other party is speaking publicly about phobia, but rather directs the other party to share its feelings and experiences in a way that is more relaxed and willing to cooperate with the investigation."}, "time_dimension": {"answer": "Present", "rationale": "Now the entry describes the current experience and feelings of the testee, the keywords being \"will\" and \"will become,\" which indicate that this is a state of present existence. Moreover, the situation of \"must speak in public\" also shows that it is a present situation or source of stress. So the time dimension of this questionnaire item is now judged."}, "sentence_type": "Affirmative declarative sentence", "pos": {"nouns": {"I have to": 1, "It's a public place": 1, "Noodles": 1, "Talk": 2, "Frightened": 1, "鈾?And become 鈾?: 1, "I don't know what to do": 1, "You're talking like a fool": 1}, "verbs": {"in": 1, "I have to": 1, "Yes": 1, "Because": 1, "鈾?And become 鈾?: 1, "Say it": 2, "have": 1}, "adjectives": {"Frightened": 1, "I don't know what to do": 1, "I'm not sure I can say it": 1}, "adverbs": {"I have to": 1, "It's a public place": 1}, "numerals": {"9": 1, "One": 2}, "classifiers": {"Noodles": 1, "individual": 1}, "pronouns": {"I": 1}}}, {"content": "10. When I ride a bike to a more distant place for social purposes and no sign of a roadway is found, I will wait patiently for the passing of a passing vehicle or others to ask.", "word_count": 49, "label": "", "sentence_structure": "Subject鈥搗erb鈥搊bject", "subjective_objective": {"answer": "Subjective", "rationale": "Because it is the patient ' s personal behaviour and attitude, i.e. how to respond to a difficult situation, it is subjective thinking and behaviour."}, "sentence_sentiment": {"answer": "Positive", "rationale": "Because the entry describes behaviour that is calm, patient and seeking help from others in the face of problems, which is a positive attitude and quality."}, "language_style": {"answer": "Tactful and warm", "rationale": "The language style of the entry is more euphemistic and warm, and it does not directly ask the other side about the reaction or attitude, but rather describes a situation and gives a possible form of behaviour and, at the end, expresses a desire. This expression is more accommodative and acceptable."}, "time_dimension": {"answer": "Present", "rationale": "The entry describes the reaction of the person who was tested in the event of difficulties, i.e. waiting for help and questioning. The act occurs in the current situation and is therefore judged as \"now\"."}, "sentence_type": "Affirmative declarative sentence", "pos": {"nouns": {"Local": 1, "Social events": 1, "Road signs": 1, "Patience": 1, "A passing car": 1, "person": 2}, "verbs": {"Ride": 1, "Participation": 1, "Wait": 2, "Walking through": 1, "Ask me": 1}, "adjectives": {"Far": 1, "Clear": 1}, "numerals": {"10": 1, "One": 2, "Comparison": 1}, "classifiers": {"individual": 1}, "pronouns": {"I": 1}}}, {"content": "I have never been willing to do it specifically, even if there is a chance, in order to make a good impression in front of others.", "word_count": 31, "label": "", "sentence_structure": "Subject鈥搗erb鈥搊bject", "subjective_objective": {"answer": "Subjective", "rationale": "Because the expression \"no\" and \"no\" are the feelings and attitudes of the patient, it is viewed from the patient's subjective perspective."}, "sentence_sentiment": {"answer": "Negative", "rationale": "Because the expressions \"involved\" and \"not done\" in the entries are all negative and negative."}, "language_style": {"answer": "Calm and direct", "rationale": "The entry is more calm and straightforward in its language, with no excessive emotional or speech modification, but rather a direct inquiry. The entry also uses negative language to express the subject matter and to make it more explicit."}, "time_dimension": {"answer": "Past", "rationale": "The entry is therefore past and present."}, "sentence_type": "Negative declarative sentence", "pos": {"nouns": {"Impression": 1, "Opportunities": 1}, "verbs": {"May I": 1, "Do it": 2, "Stay": 1, "Do it on purpose": 1, "Go on": 1, "have": 1}, "adverbs": {"Never": 1, "- Oh, yeah": 1, "Even if": 1}, "numerals": {"11": 1, "1": 3}, "pronouns": {"I": 1}}}, {"content": "12. I am rarely stubborn and open to other views in my relations with my classmates and family.", "word_count": 23, "label": "", "sentence_structure": "Subject鈥揷opula鈥損redicative", "subjective_objective": {"answer": "Subjective", "rationale": "This is because the patient's description of his/her attitude in interpersonal relations is a reflection of subjective feelings and attitudes."}, "sentence_sentiment": {"answer": "Positive", "rationale": "Because the \"better to take the other\" act in the entry is positive."}, "language_style": {"answer": "Tactful and warm", "rationale": "The language style of the entry is euphemism. It asks in a gentle and inviting manner whether the interviewee is willing to listen to others in his or her dealings with others and to reduce the number of cases in which he or she is holding his or her opinion."}, "time_dimension": {"answer": "Present", "rationale": ""}, "sentence_type": "Affirmative declarative sentence", "pos": {"nouns": {"Students": 1, "Family": 1, "Observations": 1}, "verbs": {"Get along": 1, "Obstinacy": 1, "Accepted": 1}, "adjectives": {"Very few": 1, "I'm happy": 1}, "numerals": {"12锛?: 1}}}, {"content": "13. When I argue with others, I often feel speechless and think later about how to counter each other, unfortunately too late.", "word_count": 32, "label": "", "sentence_structure": "Subject鈥搗erb鈥搊bject", "subjective_objective": {"answer": "Subjective", "rationale": "Because it is the patient who is describing his or her feelings and experiences, it is subjective attitudes and experiences that are examined, not objective facts or results."}, "sentence_sentiment": {"answer": "Negative", "rationale": "Because \"When I argue with someone, I often feel speechless\" and \"how to counter them later, it's too late\" in the entries to express negative emotions and experiences."}, "language_style": {"answer": "Calm and direct", "rationale": "The entry \"Calm down straight\" describes in a more objective and direct manner the problems that the respondents may encounter in their dispute with others, with no excessive rhetorical or emotional rendering, and with a more balanced tone."}, "time_dimension": {"answer": "Present", "rationale": "The keyword \"always\" means that the experience is regular, that \"I often feel speech-tale,\" \"remember\" and \"almost too late\" are present or recent, so it can be judged to be a description of the person being tested."}, "sentence_type": "Affirmative declarative sentence", "pos": {"nouns": {"- I'm not gonna fight with anyone": 1, "Psst": 1, "Yes": 1, "Back": 1, "The": 1, "How do you counter them": 1, "Too late": 1}, "verbs": {"I feel": 1, "Yes": 1, "Countered": 1, "Say it": 1}, "adverbs": {"Often": 1, "That's right": 1, "Already": 1}, "numerals": {"13": 1}, "classifiers": {"individual": 1}, "pronouns": {"I": 1}}}, {"content": "I am not demanding living conditions, and I can enjoy them even if they are difficult.", "word_count": 27, "label": "", "sentence_structure": "Subject鈥損redicate", "subjective_objective": {"answer": "Subjective", "rationale": "The question is examined in the context of patients ' attitudes and perceptions, namely, their satisfaction with the requirements of living conditions and their fulfilment, which are subjective."}, "sentence_sentiment": {"answer": "Neutral", "rationale": "Because the entry does not have any emotional color."}, "language_style": {"answer": "Tactful and warm", "rationale": "The language style of the entry tends to be euphemistic. By using words such as \"not demanding living conditions\" and \"whoever can be happy\", it expresses an optimistic, positive attitude and conveys this in a more soft way, without directly criticizing or negatively evaluating difficult living conditions. At the same time, the entry uses the concession phrase \"even if...\", adding a milder and more euphemism to the phrase."}, "time_dimension": {"answer": "Present", "rationale": "The entry describes the attitude and feelings of the testee with regard to living conditions, and the words \"me\" and \"capable\" indicate that the testee's personal cognitive and emotional reaction. So the time dimension of the entry is now."}, "sentence_type": "Affirmative declarative sentence", "pos": {"nouns": {"Conditions": 2, "Life": 2, "Request": 1, "Hard": 1}, "verbs": {"to": 1, "have": 1, "Request": 1, "Yes": 1}, "adjectives": {"Not high": 1, "very": 2, "Have a nice day": 1}, "adverbs": {"Even if": 1}, "pronouns": {"I": 1}}}, {"content": "15. When I am criticized, I would like to find an opportunity to criticize him.", "word_count": 20, "label": "", "sentence_structure": "Subject鈥搗erb鈥搊bject", "subjective_objective": {"answer": "Subjective", "rationale": "Because \"want\" is patient's subjective attitude."}, "sentence_sentiment": {"answer": "Negative", "rationale": "Because the contents of the entries describe acts of reprisal that are criticized and which are motivated by negative emotional tendencies."}, "language_style": {"answer": "Tactful and warm", "rationale": "The language style of the entry is euphemism. By using the expression \"will think,\" it conveys the response that the other party may have, without being too direct or harsh. At the same time, the entire sentence structure is relatively simple and understandable. So the language style of the questionnaire entry is euphemism."}, "time_dimension": {"answer": "Present", "rationale": "The keywords are \"will want\" and \"see opportunities\", which indicate the psychological orientation and possible action of the testee when the criticism occurs. So the time dimension of this entry is now."}, "sentence_type": "Affirmative declarative sentence", "pos": {"nouns": {"others": 1, "Criticism": 2, "he": 1, "she": 1}, "verbs": {"Yes": 2, "Find": 1, "Go on": 1, "Criticism": 1}, "adverbs": {"in": 1, "Time": 1}, "numerals": {"15": 1}, "classifiers": {"individual": 1}, "pronouns": {"I": 1}}}, {"content": "16. At a critical moment in the decision to win or lose, I was nervous, but I was able to calm myself quickly.", "word_count": 31, "label": "", "sentence_structure": "Subject鈥損redicate", "subjective_objective": {"answer": "Subjective", "rationale": "Because \"I'm nervous\" and \"I'm calm\" are subjective feelings and behaviours of patients. Although the situation is objective (the critical moment for success or failure), the response to this situation is one of individual subjective experience."}, "sentence_sentiment": {"answer": "Positive", "rationale": "Because the entry \"I'm nervous at the critical moment of success or failure, but I can always calm myself quickly\" shows a positive emotional control."}, "language_style": {"answer": "Tactful and warm", "rationale": "This expression is more likely to resonate with the interviewees and to inspire their willingness to participate actively in the questionnaire."}, "time_dimension": {"answer": "Present", "rationale": "The entry describes the behaviour of the testee at a critical moment, which, although tense, can calm himself quickly. The act is being answered, so it is now judged."}, "sentence_type": "Affirmative declarative sentence", "pos": {"nouns": {"Time": 2, "Criticality": 1, "self": 1}, "verbs": {"Decision": 1, "Lose": 1, "Done": 1, "Chile": 1, "Although": 1, "very": 1, "Yes": 1, "Soon": 1, "鈾?And I'm gonna be 鈾?: 1, "Calm down": 1, "Get down": 1}, "adjectives": {"Nervous": 1}, "adverbs": {"But": 1}, "numerals": {"16": 1}, "pronouns": {"I": 1}}}, {"content": "17. I don't like things that I can't learn at all.", "word_count": 16, "label": "", "sentence_structure": "Subject鈥搗erb鈥揷omplement", "subjective_objective": {"answer": "Subjective", "rationale": "Because what you don't like is the expression of the feelings, attitudes and values of the patient himself, and it's subjective."}, "sentence_sentiment": {"answer": "Negative", "rationale": "Because the entry \"What it doesn't like, can't learn at all\" expresses a negative emotion and attitude."}, "language_style": {"answer": "Tactful and warm", "rationale": "The entry asks the other side in a more euphemistic way whether there are difficulties or challenges in certain areas, which are relatively moderate in language style and do not imply direct accusations or criticism."}, "time_dimension": {"answer": "Present", "rationale": "The phrase \"I\" indicates the subjective feeling and state of the person who speaks, the description \"not like\" is a description of the current situation, and \"does not learn at all\" is a prediction of possible future behaviour. Thus, the entry on this questionnaire can be classified as a current behavior or mentality."}, "sentence_type": "Negative declarative sentence", "pos": {"nouns": {"Something": 1, "Learn": 2}, "verbs": {"Yeah": 1, "Learn": 1, "Yes": 1}, "adjectives": {"not": 1, "What": 1}, "adverbs": {"Yeah": 1}, "numerals": {"17": 1}, "classifiers": {"individual": 1}, "pronouns": {"I": 1}}}, {"content": "18. In a chaotic environment, I remain able to focus on learning and to be more efficient.", "word_count": 25, "label": "", "sentence_structure": "Subject鈥搗erb鈥搊bject", "subjective_objective": {"answer": "Objective", "rationale": "Because the sentence describes the behaviour and results of individuals in a given environment, these are objective facts. Although it relates to the individual鈥檚 feelings (i.e., 鈥渉igher efficiency鈥?, they are based on observations of the situation. The questionnaire entry is therefore based on objective observations."}, "sentence_sentiment": {"answer": "Positive", "rationale": "Because the actions and results in the entries are positive."}, "language_style": {"answer": "Calm and direct", "rationale": "The entry describes in an objective and direct way whether the person surveyed is able to maintain attention and efficiency in a noisy environment. The language style is simple and clear, and there are no excessive emotional accoutrements or rhetorical techniques, so that the language style can be judged as calm and straightforward."}, "time_dimension": {"answer": "Present", "rationale": ""}, "sentence_type": "Affirmative declarative sentence", "pos": {"nouns": {"Environment": 1, "Noise": 1, "Loud": 1, "Chaos": 1, "- The energy": 1, "Learning": 2, "Efficiency": 1}, "verbs": {"Yes": 1, "Focus": 1, "Still": 1, "And": 1, "Higher": 1}, "adjectives": {"Loud": 1, "Chaos": 1}, "adverbs": {"Still": 1, "Higher": 1}, "numerals": {"2": 1}, "classifiers": {"1": 6}, "pronouns": {"I": 1}}}, {"content": "I do not like strangers visiting my house, and I do not want to avoid them whenever they do.", "word_count": 24, "label": "", "sentence_structure": "Subject鈥搗erb鈥搊bject", "subjective_objective": {"answer": "Subjective", "rationale": "Because \"not like\" and \"intentionally evasive\" are subjective attitudes and behaviours of the patient, the point of view of this questionnaire is subjective."}, "sentence_sentiment": {"answer": "Negative", "rationale": "Because the entries \"not like strangers to come and visit\" and \"intentionally avoid\" are negative."}, "language_style": {"answer": "Tactful and warm", "rationale": ""}, "time_dimension": {"answer": "Present", "rationale": "The word \"in the event\" in the entry indicates a regular, customary act, and the word \"in the event\" implies that the act is immediate, i.e., that it is acted upon in such a case. The time dimension of the entry is therefore now."}, "sentence_type": "Negative declarative sentence", "pos": {"nouns": {"Situation": 1, "Home": 1, "Guests": 1, "Avoidance": 1}, "verbs": {"Yeah": 1, "Come on": 1, "Do it": 1, "Just": 2, "Intentionally": 1}, "adjectives": {"Strange": 1}, "adverbs": {"Every time": 1}, "pronouns": {"I": 1}}}, {"content": "20. I am very fond of social events, and I feel that this is a good opportunity to make friends.", "word_count": 22, "label": "", "sentence_structure": "Subject鈥揷opula鈥損redicative", "subjective_objective": {"answer": "Subjective", "rationale": "I love social events and the feeling that this is a good opportunity to be friends, and that it's all about the patient's own feelings and feelings."}, "sentence_sentiment": {"answer": "Positive", "rationale": "Because the words \"like social events\" and \"likes to be friends\" are positive."}, "language_style": {"answer": "Tactful and warm", "rationale": "The words \"like\" and \"good opportunity\" are used to make the respondents feel good and respectable by the designers and are more willing to share their true feelings."}, "time_dimension": {"answer": "Present", "rationale": "The entry expresses the current attitude and feelings of the testee, the keywords \"like,\" \"participating,\" \"social activity,\" which point to the behaviour that is being performed. And, \"I feel like this is a good chance to be friends\" is also a narrative description of the current state. So, the time dimension of this entry is now."}, "sentence_type": "Affirmative declarative sentence", "pos": {"nouns": {"Questionnaire Entry": 1, "Social events": 1, "friends": 1}, "verbs": {"Yeah": 1, "Participation": 1, "Feel": 1, "be": 1, "Hand it over": 1, "Okay": 1, "Opportunities": 1}, "adjectives": {"I like it": 1}, "numerals": {"20锛?: 1}, "classifiers": {"individual": 1}, "pronouns": {"I": 1}}}]}

{"questionnaire_title": "Test your personality charisma.", "questionnaire_category": "Self-cognition", "item_count": 25, "emotion_shift_overall": "From negative to positive", "language_style_overall": "Tactful and warm", "items": [{"content": "Did anyone say your eyes speak?", "word_count": 12, "label": "", "sentence_structure": "Subject鈥搗erb鈥搊bject", "subjective_objective": {"answer": "Subjective", "rationale": "The entry asks whether the patient has been commented on in his or her eyes, which is a subjective matter of perception and evaluation. This is therefore viewed from the patient鈥檚 subjective point of view."}, "sentence_sentiment": {"answer": "Neutral", "rationale": "Because the entry does not have any emotional color."}, "language_style": {"answer": "Tactful and warm", "rationale": "The language style of the entry is euphemismal, because it uses a more moderate, emotional expression to ask if the interviewees have ever said that their eyes speak. The question is not to make the interviewee feel challenged or attacked, but to build dialogue and communication in a more friendly way."}, "time_dimension": {"answer": "Present", "rationale": "Now the entry asks about the current status or experience of the subject, the keyword being \"had\" and \"had,\" all pointing to the facts or experience of the present."}, "sentence_type": "Interrogative sentence", "pos": {"nouns": {"person": 1, "Eyes": 1, "Word": 1}, "verbs": {"Say it": 2, "Yes": 1}, "adverbs": {"Pass": 1}, "pronouns": {"you": 1}}}, {"content": "Do you have contact with someone else's body while you're talking to someone?", "word_count": 20, "label": "", "sentence_structure": "Subject鈥搗erb鈥搊bject", "subjective_objective": {"answer": "Subjective", "rationale": "Because physical contact requires the patient ' s subjective will to occur."}, "sentence_sentiment": {"answer": "Neutral", "rationale": "Because the entry asks about behaviour, there is no apparent emotional tendency."}, "language_style": {"answer": "Tactful and warm", "rationale": "The language style of the entry is more moderate and friendly, and it leads the interviewee to think and answer by asking questions, rather than by direct command or instruction. This expression helps to create a climate of ease and friendship that allows the interviewee to share his thoughts and experiences more openly."}, "time_dimension": {"answer": "Present", "rationale": "The entry asks if the person who was tested would be exposed to the other person ' s body during the interview, is in the current state of behaviour or state and therefore judged to be the present."}, "sentence_type": "Interrogative sentence", "pos": {"nouns": {"you": 1, "person": 1, "Body": 1, "Talk": 1}, "verbs": {"Yes": 1, "Exposure": 1, "Present": 1}, "adverbs": {"very": 1}, "numerals": {"2": 1}}}, {"content": "- You think your life is like a party?", "word_count": 13, "label": "", "sentence_structure": "Subject鈥搗erb鈥搊bject", "subjective_objective": {"answer": "Subjective", "rationale": "Because this is the view of others, assessed from the perspective of others, and is subjective."}, "sentence_sentiment": {"answer": "Neutral", "rationale": "Because the entry does not display positive or negative emotional features, it is a description of the views of others."}, "language_style": {"answer": "Tactful and warm", "rationale": "The language style of the entry is relatively euphemistic and has some subjective typologies, rather than direct statements or orders. At the same time, metaphoric rhetoric is used to express a view of the other ' s lifestyle, leaving a more cordial and moderate feeling."}, "time_dimension": {"answer": "Present", "rationale": "The keyword \"others will think\" expresses the views and views of others, and such views and views may exist in the present, so the topic is related to the present situation."}, "sentence_type": "Interrogative sentence", "pos": {"nouns": {"person": 1, "Life": 1, "party": 1}, "verbs": {"Yes": 2, "Recognition": 1, "Yeah": 1, "Look": 1}, "adjectives": {"Don't": 1, "Yours": 1}, "adverbs": {"very": 1, "True": 1, "not": 1}, "numerals": {"3": 1}, "pronouns": {"you": 1}}}, {"content": "- You're all in your face?", "word_count": 9, "label": "", "sentence_structure": "Subject鈥搗erb鈥搊bject", "subjective_objective": {"answer": "Subjective", "rationale": "Because emotions are expressed in a person's subjective feelings and attitudes."}, "sentence_sentiment": {"answer": "Negative", "rationale": "Because too much emotional expression can lead to tension between people, and too direct emotional expression can be considered as premature or uncontrollable. So, the expression \"all written in the face\" has a negative emotional connotation."}, "language_style": {"answer": "Calm and direct", "rationale": "This is a more objective and direct language style."}, "time_dimension": {"answer": "Present", "rationale": "The key words \"all written in the face\" show whether the person's emotions are easily perceived and understood, which is a description of how emotional expression is expressed, and is in the context of the present. So the output is: \"Now.\""}, "sentence_type": "Interrogative sentence", "pos": {"nouns": {"Emotion": 1, "Face": 1}, "verbs": {"Write": 1, "in": 1}, "adverbs": {"Both": 1}, "numerals": {"4": 1}}}, {"content": "You're considered a energetic man?", "word_count": 13, "label": "", "sentence_structure": "Subject鈥搗erb鈥搊bject", "subjective_objective": {"answer": "Subjective", "rationale": "Because \"considered\" is a subjective perception, usually derived from an evaluation by another person, and is not an objective fact."}, "sentence_sentiment": {"answer": "Positive", "rationale": "Because \"absorbed\" is usually considered positive."}, "language_style": {"answer": "Tactful and warm", "rationale": "The entry is a conversation in a positive tone and a more comfortable and confident answer to questions, while also enhancing the interaction and communication between researchers and interviewees to a certain extent, and therefore the language style is more euphemismal."}, "time_dimension": {"answer": "Present", "rationale": "The entry asks about the current state or quality of the testee, and the temporal dimension is now judged. The keywords are \"a perceived\" and \"a energetic person\" , which both express the current view or description."}, "sentence_type": "Interrogative sentence", "pos": {"nouns": {"Questionnaire": 1, "Entry": 1, "- I'm very energetic": 1}, "verbs": {"I think": 1}, "numerals": {"5": 1}, "pronouns": {"you": 1}}}, {"content": "6. Even if someone tries to hide your feelings in front of you, can you read them?", "word_count": 23, "label": "", "sentence_structure": "Subject鈥搗erb鈥搊bject", "subjective_objective": {"answer": "Subjective", "rationale": "This ability is dependent on the ability of individuals to observe and sense and is a subjective experience."}, "sentence_sentiment": {"answer": "Positive", "rationale": "Because the \"read out\" behavior in the entries is perceptive and sensitive, and it is a positive quality."}, "language_style": {"answer": "Tactful and warm", "rationale": "The entry uses words like \"even\" and \"attempted\" to express an understanding, acceptance, not directly to blame or deny the other. At the same time, the question \"You can read?\" is also a peaceful and open dialogue, not an order or a judgment. So it is a soft language."}, "time_dimension": {"answer": "Present", "rationale": "According to the phrases \"read out\" and \"tear your feelings in front of you\", the sentence is a discussion of a frequent act and should therefore be in the present state of affairs."}, "sentence_type": "Interrogative sentence", "pos": {"nouns": {"person": 2, "Emotion": 1, "Questionnaire": 1, "Entry": 1, "self": 1}, "verbs": {"Try": 1, "Cover it up": 1, "Read": 1}, "adverbs": {"Even if": 1, "In front of you": 1}, "numerals": {"6": 1, "2": 1}, "pronouns": {"you": 1}}}, {"content": "7. Do you often feel the feelings that others felt at the time?", "word_count": 20, "label": "", "sentence_structure": "Subject鈥損redicate", "subjective_objective": {"answer": "Subjective", "rationale": "The entry is about whether the patient can feel the emotional feelings and experiences of others."}, "sentence_sentiment": {"answer": "Positive", "rationale": "Because the action of \"can feel the feelings that others felt at the time\" in the entry is positive."}, "language_style": {"answer": "Tactful and warm", "rationale": "The entry uses a more euphemistic and warm language style, such as \"the feeling of the emotion that others felt at the time\", making the question more friendly, friendly, and more accessible to the interviewee. At the same time, the phrase \"can't often\" is a more gentle way of asking the interviewee about his feelings and experiences, without putting them under much stress or discomfort."}, "time_dimension": {"answer": "Present", "rationale": "The entry asks about the present feelings and behaviour of the subject, the key words being \"you\", \"if you can feel it all the time,\" \"the feelings that others felt at the time\" and \"the emotions\" all point to the present situation."}, "sentence_type": "Interrogative sentence", "pos": {"nouns": {"Questionnaire": 1, "Entry": 1, "person": 1, "Emotion": 1}, "verbs": {"Feel": 2, "Yes": 1, "The long run": 1, "Regular": 1, "Feel it": 1, "others": 1, "Time": 1}, "adverbs": {"Regular": 1, "Yes": 1}, "numerals": {"7": 1}, "pronouns": {"you": 1}}}, {"content": "Do your friends come to you when they have emotional problems?", "word_count": 23, "label": "", "sentence_structure": "Subject鈥損redicate", "subjective_objective": {"answer": "Subjective", "rationale": "Because the question is examined by the patient in the eyes of others in terms of his or her perception and evaluation, it is a subjective one."}, "sentence_sentiment": {"answer": "Neutral", "rationale": "Since the entry is factual and does not cover the description of emotional attitudes, behaviour or quality, it is neutral."}, "language_style": {"answer": "Tactful and warm", "rationale": "The language style of the entry is euphemistic, because it uses a gentle, invitation-style tone to engage in dialogue and ask if the respondents are willing to be supporters of their friends on emotional issues. This is more personal and interactive."}, "time_dimension": {"answer": "Present", "rationale": "The verbs \"attend\" and \"seek\" use the general current, which means that it is a regular, customary act, and the temporal dimension of the article is therefore the present."}, "sentence_type": "Interrogative sentence", "pos": {"nouns": {"friends": 2, "Emotion": 1, "Problem": 1, "In terms of": 1}, "verbs": {"I met him": 1, "Find": 1}, "numerals": {"8": 1}, "classifiers": {"individual": 1}, "pronouns": {"they": 1, "you": 2}}}, {"content": "- You're a little sympathetic?", "word_count": 10, "label": "", "sentence_structure": "Subject鈥搗erb鈥搊bject", "subjective_objective": {"answer": "Subjective", "rationale": "This is viewed from the patient's subjective point of view, because it asks if the patient thinks he has compassion. Others think that it is an objective result, not a direct examination of the patient's subjective attitude. The answer is therefore \"subjective.\""}, "sentence_sentiment": {"answer": "Positive", "rationale": "Because the quality of compassion in the entries is positive."}, "language_style": {"answer": "Tactful and warm", "rationale": "The entry is more euphemistic and warm in its language style, using a softer way to ask the other side whether they have been perceived as having had a compassionate experience, without directly passing judgement or evaluation. Moreover, \"compassionate\" is a relatively subjective concept, and this way of asking questions is more respectful of and inclusive of the different views and feelings of the interviewees."}, "time_dimension": {"answer": "Present", "rationale": ""}, "sentence_type": "Interrogative sentence", "pos": {"nouns": {"others": 1, "Compassion": 1}, "verbs": {"I think": 1, "have": 1}, "adjectives": {"very": 1}, "numerals": {"9": 1}, "pronouns": {"you": 1}}}, {"content": "Can you calm people down when they're depressed or excited?", "word_count": 23, "label": "", "sentence_structure": "Subject鈥搗erb鈥搊bject", "subjective_objective": {"answer": "Subjective", "rationale": "According to the description of the subject, the subject of the questionnaire is about whether an individual can help others when their emotions fluctuate. So I think the subject is a subjective one."}, "sentence_sentiment": {"answer": "Positive", "rationale": "Because the entry \"can calm down\" is positive."}, "language_style": {"answer": "Tactful and warm", "rationale": "The language style of the entry is more euphemistic and warm, and is mainly manifested in the following: 1. The use of the question-and-answer form rather than direct statements or orders is more personal and interactive; 2. The expression that describes the behaviour of the other party when the other person is frustrated or excited, rather than directly pointing out the other person's problems, is more gentle and respectful; 3. To guide the other party to think about its ability to calm others, rather than telling the other person directly how to do it, is more open and inclusive."}, "time_dimension": {"answer": "Present", "rationale": "The entry asks if the testee currently has the ability to calm down, so the time dimension is now judged."}, "sentence_type": "Interrogative sentence", "pos": {"nouns": {"person": 2, "Time": 1, "Frustration": 1, "Excited": 1, "Calm down": 1}, "verbs": {"Yes": 1, "Jean": 1, "Get down": 1}, "adjectives": {"Tens": 1}, "numerals": {"10": 1}, "pronouns": {"you": 1}}}, {"content": "If you want, can you easily hide your feelings in front of others?", "word_count": 23, "label": "", "sentence_structure": "Subject鈥搗erb鈥搊bject", "subjective_objective": {"answer": "Subjective", "rationale": "Because the desire to hide feelings is a subjective will or attitude of the patient."}, "sentence_sentiment": {"answer": "Negative", "rationale": "Because \"hidden your feelings\" is usually a negative act."}, "language_style": {"answer": "Tactful and warm", "rationale": "The entry uses a soft, question-style language style rather than a direct statement or order. It talks in an invitation-style tone, more friendly and interactive. At the same time, the issue avoids too direct discussion of the emotional privacy of the other side, but instead leads the other to think and express one鈥檚 views by asking questions. This language style is more appropriate for the psychological questionnaire, which better protects the personal privacy and dignity of the person under investigation."}, "time_dimension": {"answer": "Present", "rationale": "Now."}, "sentence_type": "Interrogative sentence", "pos": {"nouns": {"Questionnaire Entry": 1, "person": 2, "Emotion": 1}, "verbs": {"Yes": 3, "Hide": 1, "Easy": 1, "in": 1}, "adjectives": {"Your own": 1, "others": 1}, "adverbs": {"If": 1, "If you": 1}, "numerals": {"11": 1, "1": 2}, "pronouns": {"you": 1}}}, {"content": "Can you show the appropriate emotion to fit the atmosphere?", "word_count": 17, "label": "", "sentence_structure": "Subject鈥搗erb鈥搊bject", "subjective_objective": {"answer": "Subjective", "rationale": "Because \"the ability and performance of an individual to demonstrate the appropriate emotion in the atmosphere at that time\" requires a patient's self-assessment and is therefore subjective."}, "sentence_sentiment": {"answer": "Positive", "rationale": "Because the action of \"showing the right emotions in the right atmosphere\" in the entry is positive."}, "language_style": {"answer": "Tactful and warm", "rationale": "The language style of the entry is euphemistic. It asks questions in a gentle and inviting manner about whether participants can show their emotions in a given situation, rather than direct orders or demands."}, "time_dimension": {"answer": "Present", "rationale": "The entry asks if the person tested can properly express feelings in the current or near future circumstances, without addressing the past or future time dimension, and therefore determines the present."}, "sentence_type": "Interrogative sentence", "pos": {"nouns": {"Grandpa": 1, "Questionnaire": 1, "Entry": 1, "At the time": 1, "Abet": 1, "Emotion": 1}, "verbs": {"Yes": 2, "Performance": 1, "Match": 1, "Appropriate": 1}, "adjectives": {"At the time": 1, "The atmosphere": 1, "Appropriate emotion": 1}, "numerals": {"12": 1, "1": 2}, "classifiers": {"individual": 1}, "pronouns": {"you": 1}}}, {"content": "13. Can you still stay calm when people around you may be losing control?", "word_count": 24, "label": "", "sentence_structure": "Subject鈥搗erb鈥搊bject", "subjective_objective": {"answer": "Subjective", "rationale": "The subject is to ask patients about subjective reactions and attitudes in specific circumstances."}, "sentence_sentiment": {"answer": "Positive", "rationale": "Because the entry is expressed as being able to remain calm in a stress environment, with positive emotional attitudes and behaviour."}, "language_style": {"answer": "Calm and direct", "rationale": "The language style of the entry is straightforward and is addressed directly to the person under investigation, mainly by means of a question, and there is no excessive emotional modification or acrimony.\""}, "time_dimension": {"answer": "Present", "rationale": "According to the sentence, \"when your people around you may be out of control,\" it can be seen that the question is asking the subject about the conduct or reaction in a given situation. This particular situation is taking place now, and it is now judged."}, "sentence_type": "Interrogative sentence", "pos": {"nouns": {"People around": 1, "Out of control": 1, "Calm down": 1}, "verbs": {"Hold on": 1, "Maybe": 1, "Both": 1, "Out of control": 1}, "adverbs": {"Still": 1}, "numerals": {"13": 1}, "pronouns": {"you": 1}}}, {"content": "You think someone else might really not know what's going on inside you?", "word_count": 22, "label": "", "sentence_structure": "Subject鈥搗erb鈥搊bject", "subjective_objective": {"answer": "Subjective", "rationale": "Because \"you think\" means that it's the patient's subjective belief that people may not know what's going on inside them."}, "sentence_sentiment": {"answer": "Neutral", "rationale": "Because the entry does not have any emotional color."}, "language_style": {"answer": "Tactful and warm", "rationale": "The entry is more euphemistic, using the \"You think\" approach to the question, to direct the interviewee to share his/her views and feelings. At the same time, the word \"occupying\" is very subtle, avoiding direct reference to sensitive topics, and generally being moderate and respectful of the interviewee ' s privacy."}, "time_dimension": {"answer": "Past", "rationale": "The entry is a question of the testee ' s views or experience with the past and therefore judgement is a past."}, "sentence_type": "Interrogative sentence", "pos": {"nouns": {"Questionnaire": 1, "Entry": 1, "person": 2, "Inside": 1, "Come on": 1}, "verbs": {"I think": 1, "Yes": 1, "Maybe": 1, "Really": 1, "Come on": 1}, "adjectives": {"What": 1}, "adverbs": {"What the hell": 1}, "numerals": {"14": 1, "2": 1}, "pronouns": {"you": 1, "others": 1}}}, {"content": "When people are in crisis, they think of you?", "word_count": 16, "label": "", "sentence_structure": "Subject鈥搗erb鈥搊bject", "subjective_objective": {"answer": "Subjective", "rationale": "Because it's the patient who feels subjectively."}, "sentence_sentiment": {"answer": "Neutral", "rationale": "Because the entry does not have any emotional color."}, "language_style": {"answer": "Tactful and warm", "rationale": "The entry is a soft, invited conversation, asking if the person interviewed is reminded of someone else in crisis, which is more personal and interactive. Moreover, the expression \"thinking you\" is vague, does not directly suggest what kind of response or behaviour the person interviewed should be, thus avoiding overly strong or prescriptive tone."}, "time_dimension": {"answer": "Present", "rationale": "The entry asks about the current situation of the subjects, i.e. whether they will be remembered by others for their role in other people's crises and therefore judge as \"now\"."}, "sentence_type": "Interrogative sentence", "pos": {"nouns": {"Crisis": 1, "Time": 1}, "verbs": {"Yes": 3}, "adverbs": {"When": 1, "they": 1}, "numerals": {"15": 1}, "pronouns": {"you": 1}}}, {"content": "You're a great listener?", "word_count": 11, "label": "", "sentence_structure": "Subject鈥損redicate", "subjective_objective": {"answer": "Subjective", "rationale": "Because excellence is an assessment or judgement, it is a subjective feeling or attitude."}, "sentence_sentiment": {"answer": "Positive", "rationale": "Because the word \"excellent\" is a positive adjective."}, "language_style": {"answer": "Tactful and warm", "rationale": "The entry uses a polite inquiry and leads the respondents to think about their abilities through positive evaluations, which are more moderate and positive."}, "time_dimension": {"answer": "Present", "rationale": "According to the entry sentence, \"You're a very good listener?\" The question can be seen in the tester's current state, so it's now."}, "sentence_type": "Interrogative sentence", "pos": {"nouns": {"Listener": 1, "Excellent": 1}, "verbs": {"be": 1, "Give": 1}, "adjectives": {"One": 2, "very": 1}, "adverbs": {"Very": 1, "not": 1}, "pronouns": {"you": 1}}}, {"content": "You're always careful in social settings not to get yourself into a situation?", "word_count": 20, "label": "", "sentence_structure": "Subject鈥搗erb鈥揷omplement", "subjective_objective": {"answer": "Subjective", "rationale": "Because of the subjective feelings and attitudes of the patients, they are examined in their behavioural and psychological state in the social setting."}, "sentence_sentiment": {"answer": "Negative", "rationale": "Because the entry \"Always be careful not to get yourself into a situation\" is negative."}, "language_style": {"answer": "Tactful and warm", "rationale": "This way neither puts pressure on the person interviewed nor improves access to real information."}, "time_dimension": {"answer": "Present", "rationale": ""}, "sentence_type": "Interrogative sentence", "pos": {"nouns": {"Socially": 1, "The dilemma": 1}, "verbs": {"Always": 1, "Jean": 1, "Into the": 1}, "adjectives": {"Watch out": 1}, "adverbs": {"very": 1}, "numerals": {"17": 1}}}, {"content": "You know a lot of social rituals?", "word_count": 9, "label": "", "sentence_structure": "Subject鈥損redicate", "subjective_objective": {"answer": "Subjective", "rationale": "The question is whether the individual knows about social rituals, which is a subjective perception and experience of the individual."}, "sentence_sentiment": {"answer": "Neutral", "rationale": "Because the entry does not have any emotional color."}, "language_style": {"answer": "Tactful and warm", "rationale": "The language style of the entry is more euphemistic and gentle, and it leads the interviewee to answer questions in a question-and-answer manner, and it does not use too direct or hard language. Moreover, the expression \"you know\" is more polite and respectful of each other. So the language style of the questionnaire entry is euphemistic and warm."}, "time_dimension": {"answer": "Present", "rationale": "The entry asks if the testee is aware of many social ceremonies at the moment, and therefore the time dimension is now judged."}, "sentence_type": "Interrogative sentence", "pos": {"nouns": {"you": 1, "Social rituals": 1}, "verbs": {"Yes": 1}, "adjectives": {"A lot": 1}, "numerals": {"18": 1}}}, {"content": "All the words you say are subject to constant scrutiny?", "word_count": 17, "label": "", "sentence_structure": "Subject鈥損redicate", "subjective_objective": {"answer": "Subjective", "rationale": "Because \"the words are being considered\" is the patient's subjective perception and judgment."}, "sentence_sentiment": {"answer": "Neutral", "rationale": "Because the entry does not have any emotional color."}, "language_style": {"answer": "Calm and direct", "rationale": "The language style of the entry is relatively simple, and there is no excessive emotional accommodation or euphemism, but rather a direct question about whether the other person is very thoughtful when he or she speaks."}, "time_dimension": {"answer": "Present", "rationale": ""}, "sentence_type": "Interrogative sentence", "pos": {"nouns": {"Word": 1, "Consideration": 1}, "verbs": {"Say it": 1, "be": 1, "The long run": 1, "Three": 2}, "adverbs": {"Every one": 1, "Both": 1, "Three": 1}, "numerals": {"19": 1}, "pronouns": {"you": 1}}}, {"content": "You'll watch the people around you?", "word_count": 12, "label": "", "sentence_structure": "Subject鈥搗erb鈥搊bject", "subjective_objective": {"answer": "Subjective", "rationale": "Because of the self-awareness and feelings of the patients, the question is whether they will consciously observe the behaviour of the people around them."}, "sentence_sentiment": {"answer": "Neutral", "rationale": "Because the entry does not have any emotional color."}, "language_style": {"answer": "Calm and direct", "rationale": "The entry uses a direct and clear language style to ask the interviewee whether he or she is concerned about the behaviour of others, without any emotional or rhetorical use, and is expressed in a concise manner."}, "time_dimension": {"answer": "Present", "rationale": "The entry asks the subject about his current behaviour, the keywords being \"will\" and \"observation\", which point to the state of the moment, which is happening."}, "sentence_type": "Interrogative sentence", "pos": {"nouns": {"person": 1, "Every move": 1}, "verbs": {"Yes": 1, "Observation": 1}, "numerals": {"20": 1}}}, {"content": "21. Do people think you're a man of confidence?", "word_count": 18, "label": "", "sentence_structure": "Subject鈥損redicate", "subjective_objective": {"answer": "Subjective", "rationale": "Because this is a subjective view of what others think of you and evaluate you."}, "sentence_sentiment": {"answer": "Positive", "rationale": "Because the description of \"good self-confidence\" in the entry is positive."}, "language_style": {"answer": "Tactful and warm", "rationale": "The entry uses a query-based approach and uses positive terminology (such as \"goody\" and \"confident\"), which is more encouraging and positive, and helps to create a positive and friendly atmosphere that guides respondents to answer more readily."}, "time_dimension": {"answer": "Present", "rationale": "The keyword \"considers\" and \"is\" indicates that it is a description of the current state and evaluation of the person being tested, and therefore the judgement is now."}, "sentence_type": "Interrogative sentence", "pos": {"nouns": {"person": 1, "I'm glad": 1, "Confidence": 1}, "verbs": {"I think": 1, "be": 1, "One": 2}, "adjectives": {"Fill": 1, "Self-confident": 1}, "adverbs": {"You're so good": 1}, "numerals": {"21": 1}, "pronouns": {"you": 1}}}, {"content": "22. You rarely panic or be embarrassed in social settings?", "word_count": 16, "label": "", "sentence_structure": "Subject鈥揷opula鈥損redicative", "subjective_objective": {"answer": "Objective", "rationale": "Because the question is to examine the patient's psychological state in a social setting, and \"fright or awkward\" is the patient's subjective feeling and experience, the answer should be objective."}, "sentence_sentiment": {"answer": "Neutral", "rationale": "Because the entry does not have any emotional color."}, "language_style": {"answer": "Calm and direct", "rationale": "The language style of the entry is calm and straightforward. It uses simple and clear language, asking whether the person surveyed is often panicned or embarrassed in social settings. This straightforward approach avoids too many rhetorical or emotional overtones, making the problem clearer."}, "time_dimension": {"answer": "Present", "rationale": "The entry for this questionnaire asks about the psychological state and feelings of the person currently being tested in the social setting, and therefore judges it as \"now\". The keyword \"very rarely\" indicates that it is a regular act or condition, while \"in the social setting\" further clarifies the circumstances and circumstances in which it occurs."}, "sentence_type": "Interrogative sentence", "pos": {"nouns": {"Socially": 1, "I'm scared": 1, "Awkward": 1}, "verbs": {"I feel": 1, "Very few": 1, "In... in": 1}, "adverbs": {"Very few": 1}, "numerals": {"22": 1}}}, {"content": "You can live with different kinds of people?", "word_count": 14, "label": "", "sentence_structure": "Subject鈥搗erb鈥搊bject", "subjective_objective": {"answer": "Subjective", "rationale": "Because the ability to live in harmony with different types of people is the patient ' s subjective attitude and ability."}, "sentence_sentiment": {"answer": "Positive", "rationale": "Because the article is intended to be \"coherent with different types of people\", it is a positive attitude."}, "language_style": {"answer": "Tactful and warm", "rationale": "The language style of the entry is more euphemistic and moderate, and it does not use strong tone or command style expression, but rather leads the respondents to answer in a way that is asked and invited."}, "time_dimension": {"answer": "Present", "rationale": ""}, "sentence_type": "Interrogative sentence", "pos": {"nouns": {"Questionnaire": 1, "Entry": 1, "Type": 1, "person": 1}, "verbs": {"Yes": 1, "and": 1, "Get along": 1}, "adjectives": {"Different": 1, "Harmony": 1}, "numerals": {"23": 1}}}, {"content": "Can you talk to strangers without fear?", "word_count": 13, "label": "", "sentence_structure": "Subject鈥搗erb鈥搊bject", "subjective_objective": {"answer": "Subjective", "rationale": "The subject is considered to be the patient ' s willingness and attitude to communicate with strangers and is subjective."}, "sentence_sentiment": {"answer": "Neutral", "rationale": "Because the entry is concerned with the attitude of individuals towards strangers and does not involve positive or negative emotional aspects."}, "language_style": {"answer": "Calm and direct", "rationale": "This language style is suitable for psychological questionnaires, which allow the person surveyed to express his or her thoughts and feelings more honestly and accurately."}, "time_dimension": {"answer": "Present", "rationale": "The entry \"now\" is about asking the testee about his current state or behaviour, that is, whether they can speak to strangers now. The key word is \"you can\" and \"you can talk\" -- all about the state or behavior that you are doing."}, "sentence_type": "Interrogative sentence", "pos": {"nouns": {"you": 1, "Stranger": 1, "Talk": 1}, "verbs": {"Yes": 1, "Fire": 1, "Heart": 1}, "adjectives": {"Don't worry": 1, "Strange": 1}, "adverbs": {"Land": 1, "Go on": 1}}}, {"content": "25. Do you often get elected as an advocate or leader of a team?", "word_count": 20, "label": "", "sentence_structure": "Subject鈥搗erb鈥搊bject", "subjective_objective": {"answer": "Subjective", "rationale": "Because the person selected to represent or lead the group is the individual鈥檚 perception and evaluation, it is an objective fact. And whether the individual is willing to accept and play such a role reflects his or her subjective attitude."}, "sentence_sentiment": {"answer": "Positive", "rationale": "Because the action of being \"selected as an advocate or leader of a team\" in the entry is positive, it implies that the person has influence and competence in the team."}, "language_style": {"answer": "Calm and direct", "rationale": "This is a quiet, direct language style that allows the respondents to understand the questions more clearly and to provide accurate answers."}, "time_dimension": {"answer": "Present", "rationale": "Now."}, "sentence_type": "Interrogative sentence", "pos": {"nouns": {"Entry": 1, "Panel": 1, "Advocate": 1, "Leadership": 1}, "verbs": {"By": 1, "Selection": 1, "Regular": 1}, "adverbs": {"Regular": 1, "Or": 1}, "numerals": {"25": 1}, "pronouns": {"you": 1}}}]}

{"questionnaire_title": "Baker-Lafanson's Dreary Card", "questionnaire_category": "Behavioral questionnaire", "item_count": 49, "emotion_shift_overall": "From positive to negative", "language_style_overall": "Calm and direct", "items": [{"content": "Baker-Lafanson's Dreary Card", "word_count": 9, "label": "", "sentence_structure": "Subject鈥損redicate", "subjective_objective": {"answer": "Objective", "rationale": "Because the Baker-Lafanson Manic Madness Table is a tool for assessing whether individuals are suffering from manic excesses, and it draws conclusions mainly by observing and quantifying the behaviour, thinking, and emotions of patients. These indicators are objective and can be measured and validated by scientific methods. Thus, the entry \"Beck-Lafanson Manic Madness Sheet\" examines patients' symptoms from an objective perspective."}, "sentence_sentiment": {"answer": "Neutral", "rationale": "Because the entry does not express positive or negative feelings, attitudes, behaviour, purpose or quality."}, "language_style": {"answer": "Calm and direct", "rationale": "The language style of the entry is straightforward and it clearly describes the name of the measurement tool, does not add too much emotional colour or acrimony, but is expressed in an objective and clear manner."}, "time_dimension": {"answer": "Present", "rationale": "The keyword \"Beck-Lafanson's Manic Madness Scale\" refers to a tool that is currently widely used to assess manic symptoms, so the temporal dimension is now judged."}, "sentence_type": "Interrogative sentence", "pos": {"nouns": {"Beck": 1, "Lafanson": 1, "- Manic": 1, "Scale": 1}}}, {"content": "Two, a little bit more.", "word_count": 8, "label": "", "sentence_structure": "Subject鈥搗erb鈥揷omplement", "subjective_objective": {"answer": "Subjective", "rationale": "Because \"slightly motionful\" and \"active expression\" are subjective feelings of patients, they are subjectively viewed."}, "sentence_sentiment": {"answer": "Positive", "rationale": "Because the entry \"slightly more action, active expression\" describes lively, open behavioral features with positive emotional features."}, "language_style": {"answer": "Tactful and warm", "rationale": "The entry is more personal and interactive when it is spoken in a gentle and inviting tone, rather than in direct statements or orders. At the same time, the descriptive expression \"slightly motionful, face-active\" is also more objective and neutral, with no excessive subjective emotional colours, consistent with the \"sweetly\" language style."}, "time_dimension": {"answer": "Present", "rationale": ""}, "sentence_type": "Interrogative sentence", "pos": {"verbs": {"A little bit more": 1, "Performance": 1, "Active": 1}, "adjectives": {"Actions": 1, "Emoticons": 1}, "adverbs": {"A little": 1}}}, {"content": "Three, a lot of action.", "word_count": 7, "label": "", "sentence_structure": "Subject鈥搗erb鈥揷omplement", "subjective_objective": {"answer": "Subjective", "rationale": "Because \"show and pose\" is a self-presentation and observation of the patient, it is viewed from a subjective perspective."}, "sentence_sentiment": {"answer": "Positive", "rationale": "Because \"showful, active\" is usually considered a positive adjective, which means lively and dynamic."}, "language_style": {"answer": "Tactful and warm", "rationale": "The entry uses a more imaged description of the behaviour of the person, rather than a direct statement or order, which is more euphemism and friendly. At the same time, the \"many, active pose\" also gives a positive feeling of a positive development that is more likely to resonate with the person under investigation."}, "time_dimension": {"answer": "Present", "rationale": "According to the sentence entered, \"3\" is a dynamic pose describing the physical characteristics of the person being tested, which does not clearly indicate the point of time and is therefore the current state."}, "sentence_type": "Affirmative declarative sentence", "pos": {"verbs": {"Actions": 1, "Position": 1, "Active": 1}, "adjectives": {"More": 1}}}, {"content": "Four. Very many moves.", "word_count": 12, "label": "", "sentence_structure": "Subject鈥搗erb鈥揷omplement", "subjective_objective": {"answer": "Objective", "rationale": "Because \"very much action\" and \"a rise in the talks\" are the actual manifestations of the patient in the meeting, not his subjective thoughts or feelings. So, this is an objective look."}, "sentence_sentiment": {"answer": "Positive", "rationale": "Because the act of \"stand-up activities\" is positive, it shows that the person under investigation is able to live up to the atmosphere in which he or she communicates with others."}, "language_style": {"answer": "Calm and direct", "rationale": "The entry, in descriptive terms, directly identifies the behaviour of the person who was tried to be in frequent movement during the talks and who has been up and running, without using excessive rhetorical or emotional rendering, and is more objective in tone. Thus, the language style of the entry can be judged as \"cool and direct\"."}, "time_dimension": {"answer": "Past", "rationale": ""}, "sentence_type": "Imperative sentence", "pos": {"nouns": {"Actions": 1, "Yes": 1, "Talk": 1, "Time": 1, "Tsang": 1, "Stand up": 1, "Activities": 1}, "verbs": {"A lot": 1, "Meetings": 1, "Stand up": 1}, "adverbs": {"More": 1, "Tsang": 1}, "numerals": {"4": 1}}}, {"content": "Five, you're moving, you're still in a state of disquiet.", "word_count": 14, "label": "", "sentence_structure": "Subject鈥搗erb鈥揷omplement", "subjective_objective": {"answer": "Subjective", "rationale": "Because of the constant movement, the patient ' s subjective feelings and experiences of the unrest, even though he is persuaded, are described in terms of the patient ' s inner state."}, "sentence_sentiment": {"answer": "Negative", "rationale": "Because the act of \"sitting in disquiet\" is negative."}, "language_style": {"answer": "Tactful and warm", "rationale": "Although it asks the other side whether there is \"continue to move and to persuade him to sit in disquiet and to remain in a state of disquiet or criticism,\" it does not use the term \"no\" directly. Rather, it uses a more gentle and polite way of expressing the issue and trying to communicate with the other side in a more friendly and understanding manner, which reflects the euphemism of the language style of the article."}, "time_dimension": {"answer": "Present", "rationale": ""}, "sentence_type": "Affirmative declarative sentence", "pos": {"nouns": {"Move": 1, "Stop": 1, "Say it": 2, "Peace": 1}, "verbs": {"Move": 1, "Give it to me": 1, "- I'm gonna get you out of here": 1, "Sit up": 1, "Unsettled": 1}, "adverbs": {"鈾?And it's not stopping 鈾?: 1, "Although": 1, "Still": 1}, "numerals": {"5": 1}, "classifiers": {"individual": 1}}}, {"content": "2 More talk.", "word_count": 3, "label": "", "sentence_structure": "Subject鈥搗erb鈥揷omplement", "subjective_objective": {"answer": "Subjective", "rationale": "Because \"speak more\" is a description of the patient's own performance, reflecting his subjective feelings and attitude."}, "sentence_sentiment": {"answer": "Neutral", "rationale": "Because the entry does not have any emotional color."}, "language_style": {"answer": "Tactful and warm", "rationale": "The language style of the entry is euphemism, as it is more intimate and interactive in terms of its tone of conversation in a gentle and inviting manner, rather than direct statements or orders."}, "time_dimension": {"answer": "Present", "rationale": "The entry does not have a specific time frame or future expectation, describing the present behaviour or characteristics of the person being tested and therefore judging as now."}, "sentence_type": "Negative declarative sentence", "pos": {"adjectives": {"More": 1}}}, {"content": "Three, more talk. No automatic pause.", "word_count": 8, "label": "", "sentence_structure": "Subject鈥搗erb鈥揷omplement", "subjective_objective": {"answer": "Subjective", "rationale": "Because of the habit and style of one ' s speech, there is more or less no automatic pause, which is a subjective experience of individuals."}, "sentence_sentiment": {"answer": "Negative", "rationale": "Because of the \"speaks, few automatic stops\" in the entries, a negative behaviour or characteristic is expressed."}, "language_style": {"answer": "Calm and direct", "rationale": "The entry appears in a concise question form, with more direct tone, with no excessive acrimony or emotional colour, and is more suitable for use in psychological questionnaires."}, "time_dimension": {"answer": "Present", "rationale": ""}, "sentence_type": "Affirmative declarative sentence", "pos": {"nouns": {"Word": 1, "Autostop": 1}, "verbs": {"More": 1, "have": 1, "Stop": 1}, "adjectives": {"- A few": 1}, "numerals": {"3": 1, "How many": 1}}}, {"content": "4. It's hard to interrupt", "word_count": 4, "label": "", "sentence_structure": "Subject鈥搗erb鈥揷omplement", "subjective_objective": {"answer": "Subjective", "rationale": "Interruption is a subjective act of a person and can reflect the character characteristics or mental state of the individual."}, "sentence_sentiment": {"answer": "Neutral", "rationale": "Because the entry does not have any emotional color."}, "language_style": {"answer": "Calm and direct", "rationale": "The entry directly expresses the question whether the subject is frequently interrupted in the communication, does not use excessive rhetoric or emotional rendering, and is more objective in tone."}, "time_dimension": {"answer": "Present", "rationale": "The entry describes the person's current state or habitual behaviour, and the keywords are \"hard\" and \"cut off\" and all point to the actions that are being carried out."}, "sentence_type": "Interrogative sentence", "pos": {"nouns": {"Interrupt": 1}, "verbs": {"very": 1, "Interrupt": 1}, "adverbs": {"very": 1}}}, {"content": "5. Unable to interrupt", "word_count": 4, "label": "", "sentence_structure": "Subject鈥搗erb鈥揷omplement", "subjective_objective": {"answer": "Objective", "rationale": "Because \"cannot interrupt\" is an objective expression, which describes the behaviour of the person under investigation in a given situation, rather than their subjective feelings or attitudes. The answer to this questionnaire is therefore objective."}, "sentence_sentiment": {"answer": "Neutral", "rationale": "Because the entry does not have any emotional color."}, "language_style": {"answer": "Calm and direct", "rationale": "The language style of the entry is relatively calm and straightforward, and it does not use too many glossy or emotional expressions, but rather describes the feelings or behaviour of the person questioned in a concise and concise language."}, "time_dimension": {"answer": "Past", "rationale": "However, it is assumed that the problem may be a survey of the current situation of the testees. If there are similar questions in the questionnaire in other parts, then it is assumed that these questions relate to current or recent behaviour or feelings, not just past behaviour."}, "sentence_type": "Negative declarative sentence", "pos": {"nouns": {"Questionnaire": 1, "Entry": 1}, "verbs": {"Interrupt": 1}, "adverbs": {"Cannot": 1}}}, {"content": "2. Too many terms of description, qualifying or explanation", "word_count": 12, "label": "", "sentence_structure": "Subject鈥損redicate", "subjective_objective": {"answer": "Objective", "rationale": "This is an objective description and evaluation of the linguistic characteristics of the patient."}, "sentence_sentiment": {"answer": "Negative", "rationale": "Because the word \"too many\" is generally used to describe negative situations, indicating that the description, qualifying or interpretation is too cumbersome and may create difficulties for understanding."}, "language_style": {"answer": "Calm and direct", "rationale": "The entry uses a more direct and concise language style, does not use too many descriptive or retrofitted terms and focuses more on conveying messages than on emotional rendering."}, "time_dimension": {"answer": "Past", "rationale": "The entry requires the testee to describe and assess himself, without a clear temporal dimension, and therefore cannot be determined whether this is past, present or future behaviour or mentality."}, "sentence_type": "Affirmative declarative sentence", "pos": {"nouns": {"Questionnaire": 1, "Entry": 1}, "verbs": {"Description": 1, "Modifier": 1, "Explanation": 1, "Too much": 1}}}, {"content": "3. Scattered or detached content, intentionally associated, vocal or puntual", "word_count": 17, "label": "", "sentence_structure": "Subject鈥搗erb鈥揷omplement", "subjective_objective": {"answer": "Objective", "rationale": "This is a description of the linguistic characteristics of the patient in answering the questions and is an objective observation."}, "sentence_sentiment": {"answer": "Neutral", "rationale": "Because the entry does not have any emotional color."}, "language_style": {"answer": "Calm and direct", "rationale": "The basis for the determination: - The purpose of this article is expressed in a more concise and clear manner, without the use of excessive rhetorical techniques or emotional rendering. - The type of error (scattered, detached) in the content is clearly indicated in the entry and the use of these errors is requested in a direct manner."}, "time_dimension": {"answer": "Present", "rationale": "The entry describes the current behaviour or behaviour of the person being tested, and the keywords are \"scattered or detached\", \"intentional connection\", \"symphony or puny-word\" which represent an ongoing state. Thus, the temporal dimension is now judged."}, "sentence_type": "Interrogative sentence", "pos": {"nouns": {"Questionnaire": 1, "Entry": 1, "Contents": 1, "Wandering": 1, "Out of the question": 1, "Association": 1, "Audio Union": 1, "Punctuation": 1}, "verbs": {"Analysis": 1}, "adjectives": {"A little": 1}, "numerals": {"3": 1}}}, {"content": "4. Ignorance of thinking", "word_count": 6, "label": "", "sentence_structure": "Subject鈥搗erb鈥揷omplement", "subjective_objective": {"answer": "Subjective", "rationale": "Because \"drainless\" is a subjective description or feeling of the state of your mind."}, "sentence_sentiment": {"answer": "Negative", "rationale": "Because the \"scattered mind\" act is negative."}, "language_style": {"answer": "Calm and direct", "rationale": "The entry \"Calm Direct\" directly expresses the question of whether there is a disorderly mind-blowing, is not based on too many rhetorical techniques or emotional renderings and is more objective in tone."}, "time_dimension": {"answer": "Present", "rationale": "The entry describes the current state and performance of the testee, the keyword \"drained mind\" and they are all described in terms of the present situation."}, "sentence_type": "Negative declarative sentence", "pos": {"nouns": {"Thinking": 1, "Wandering": 1, "Order": 1}, "adjectives": {"Orderless": 1}}}, {"content": "5. Isolation and incoherence, which are not understandable", "word_count": 11, "label": "", "sentence_structure": "Subject鈥搗erb鈥揷omplement", "subjective_objective": {"answer": "Objective", "rationale": "Because \"inconsistent\" and \"incomprehensible\" are the things that describe the patient's state of mind, and are the result of objective observations."}, "sentence_sentiment": {"answer": "Negative", "rationale": "Because the description \"inconsistent thinking, content is not understandable\" in the entry is negative."}, "language_style": {"answer": "Calm and direct", "rationale": "The language style of the entry is more calm and straightforward, and it clearly sets out the objective of observation and evaluation 鈥?whether the person surveyed has a coherent mind and whether the content is easily understood. This description is not based on excessive rhetorical or emotional overtones, but is fact-oriented, with high objectivity and accuracy."}, "time_dimension": {"answer": "Present", "rationale": "The entry is for the current state of the testee's mind, and the time dimension is now judged."}, "sentence_type": "Imperative sentence", "pos": {"nouns": {"Thinking": 1, "Coherence": 1, "Contents": 1, "Understood": 1}, "verbs": {"not": 1, "Yes": 1, "Understood": 1}, "adjectives": {"Coherence": 1, "Cannot": 1}, "numerals": {"5": 1}}}, {"content": "2. High voice", "word_count": 5, "label": "", "sentence_structure": "Subject鈥搗erb鈥揷omplement", "subjective_objective": {"answer": "Subjective", "rationale": "Because the sound is the patient ' s subjective feeling and experience, not the objective fact."}, "sentence_sentiment": {"answer": "Negative", "rationale": "Basis: As \"sounding\" is often seen as a bad social habit, it may be considered a rude or disrespectful expression. Therefore, \"sounding\" is often seen as an expression of negative feelings and attitudes."}, "language_style": {"answer": "Tactful and warm", "rationale": "The language style of the entry is relatively mild. It does not directly criticize or accuse the other of being too loud, but rather asks the other in a balanced and inviting manner. This way better allows the other to accept the question and to answer it, and is more conducive to creating a good atmosphere of communication."}, "time_dimension": {"answer": "Present", "rationale": "The keyword \"speaks loud\" denotes a behavior or habit that is now in progress, so the time dimension of the entry is now judged."}, "sentence_type": "Imperative sentence", "pos": {"verbs": {"Talk": 1, "High": 1}, "adjectives": {"Sound": 1}}}, {"content": "Three, speak loud. You can still hear from a distance.", "word_count": 14, "label": "", "sentence_structure": "Subject鈥搗erb鈥揷omplement", "subjective_objective": {"answer": "Objective", "rationale": "Because it is objective to speak out and to be heard from afar, there is no need for patient subjective attitudes or ideas."}, "sentence_sentiment": {"answer": "Neutral", "rationale": "Because the entry does not have any emotional color."}, "language_style": {"answer": "Tactful and warm", "rationale": "The entry is a soft and warm language style, which leads respondents to answer by asking questions rather than by direct command or evaluation, which is more apt and acceptable. At the same time, the description \" speak loud, and can still be heard at a distance\" is more objective and descriptive, and does not have too much emotional or subjective judgement, and is consistent with the language style."}, "time_dimension": {"answer": "Present", "rationale": ""}, "sentence_type": "Imperative sentence", "pos": {"nouns": {"Sound": 1, "Distance": 1}, "verbs": {"Say it": 1, "Yes": 1}, "adjectives": {"Louder": 1}, "adverbs": {"Still": 1}, "numerals": {"3": 1}}}, {"content": "4 and very high voice with singing or noise", "word_count": 11, "label": "", "sentence_structure": "Subject鈥搗erb鈥揷omplement", "subjective_objective": {"answer": "Subjective", "rationale": "Because \"high\" and \"singing\" and \"crumpy\" are subjective descriptions and feelings of patients."}, "sentence_sentiment": {"answer": "Negative", "rationale": "Because the description \"singing or manic sounds\" in the entry is negative and indicates an inaccurate pronunciation."}, "language_style": {"answer": "Calm and direct", "rationale": "The article is formulated in a more straightforward way, without too much rhetorical or emotional colour. It directly points to the problems that the person under investigation may have with the use of voice, i.e., with a high voice and a voice or noise. 2. In terms of tone, the entry appears relatively objective and calm, and does not have too much emotional orientation or emotional publicity. This language style is more appropriate for use in the questionnaire survey, because it helps researchers to collect information more accurately and objectively."}, "time_dimension": {"answer": "Present", "rationale": "The entry describes the voice of the person being tested during the conduct of an activity (e.g. speech, singing, etc.), and the time dimension is now judged."}, "sentence_type": "Imperative sentence", "pos": {"nouns": {"Voice": 1, "Songs": 1, "Mock Sound": 1}, "verbs": {"Very high": 1, "Tie": 1}, "adjectives": {"High": 1}, "numerals": {"4": 1}}}, {"content": "5. Shout or scream", "word_count": 5, "label": "", "sentence_structure": "Subject鈥損redicate", "subjective_objective": {"answer": "Objective", "rationale": "The part of the questionnaire that reads \"scream or scream\" is to examine whether a patient has committed a particular act or reaction, and therefore it is objective. Such behaviour has already occurred and does not require the patient's subjective feelings to judge. Thus, \"scream or scream\" is an objective part."}, "sentence_sentiment": {"answer": "Negative", "rationale": "Because \"scream or scream\" is usually considered negative."}, "language_style": {"answer": "Tactful and warm", "rationale": "The language style of the entry is relatively euphemistic and warm, using an invitation-based question-and-answer format and not directly addressing overly sensitive or offensive topics, making it easier for the respondents to accept and answer questions."}, "time_dimension": {"answer": "Future", "rationale": "The entry describes a possible action, \"scream\" and \"scream\" are unfinished, so the dimension of time is the future."}, "sentence_type": "Imperative sentence", "pos": {"nouns": {"Shout": 1, "Screaming": 1}, "verbs": {"Shouting or screaming": 1}}}, {"content": "2. A little hasty or irritating, controlable.", "word_count": 10, "label": "", "sentence_structure": "Subject鈥搗erb鈥揷omplement", "subjective_objective": {"answer": "Subjective", "rationale": "Because controlling emotions requires a certain subjective effort."}, "sentence_sentiment": {"answer": "Negative", "rationale": "Because the description of \"a little haste or irritation\" is negative, although the latter part says it can be controlled, it still has a negative emotional connotation."}, "language_style": {"answer": "Calm and direct", "rationale": "The language style of the entry is calm and straightforward. It asks the interviewee whether he is a little bit impatient or impulsive, and emphasizes that this is manageable. It is a relatively simple expression, with no excessive emotional overturning, and a greater focus on gathering information and data."}, "time_dimension": {"answer": "Past", "rationale": "There is no clear past, present or future hint in the time dimension, and it is therefore difficult to judge clearly its temporal properties."}, "sentence_type": "Affirmative declarative sentence", "pos": {"nouns": {"Questionnaire": 1, "Entry": 1}, "verbs": {"Analysis": 1, "A little hasty": 1, "or": 1, "It's a little hot": 1, "It's got control": 1}, "adjectives": {"Routine": 1, "A little": 1, "Easy": 1, "- You're a bad guy": 1, "Yes": 1, "Control": 1}, "adverbs": {"or": 1}, "classifiers": {"individual": 1}}}, {"content": "3. Obviously impatient, irritated or angry", "word_count": 10, "label": "", "sentence_structure": "Subject鈥揷opula鈥損redicative", "subjective_objective": {"answer": "Subjective", "rationale": "Because these emotional responses are often perceived and experienced by patients themselves, irritating, irritating or irritating, they are subjective in nature."}, "sentence_sentiment": {"answer": "Negative", "rationale": "Because \"freaky\" and \"freaky\" are negative emotional attitudes."}, "language_style": {"answer": "Calm and direct", "rationale": "The language style of the entry is more straightforward, with no rhetorical or emotional rendering, but simply describes the possible behaviour characteristics of the person under investigation. This expression makes the question clearer, more concise, easier to understand and answer."}, "time_dimension": {"answer": "Present", "rationale": "Keywords \"manifestly hasty\", \"facilitating\" and \"irregular\" point to the current emotional experience of the subjects."}, "sentence_type": "Imperative sentence", "pos": {"nouns": {"Questionnaire": 1, "Entry": 1, "Obviously": 1, "Routine": 1, "It's a little hot": 1, "Easier than I thought": 1}, "verbs": {"Analysis": 1, "have": 1, "Routine": 1, "It's a little hot": 1, "Easier than I thought": 1}, "adjectives": {"Obviously": 1}}}, {"content": "4. Threatened sex, but plagiarized", "word_count": 11, "label": "", "sentence_structure": "Subject鈥揷opula鈥損redicative", "subjective_objective": {"answer": "Subjective", "rationale": "The reference to the questionnaire examines whether the patient has threatened sex and whether it can be plagued, based on the patient鈥檚 subjective feelings and perceptions. The entry is therefore viewed from a subjective perspective."}, "sentence_sentiment": {"answer": "Positive", "rationale": "According to the information given in the title, I think that the purpose is positive. Because, despite the existence of \"threatous sexual behaviour\", such behaviour can be appeased, indicating the possibility that the problem can be solved and improved and thus have some positive significance."}, "language_style": {"answer": "Tactful and warm", "rationale": "The language style of the entry is more euphemistic, not in direct or cold language, but in a calm and understanding manner, which makes the interviewees more willing to share their own situation. At the same time, the expression \"settled\" is used, with a more problem-solving approach than a fingertip or criticism, reflecting a positive upward attitude."}, "time_dimension": {"answer": "Present", "rationale": "The entry asks if the person tested currently has certain specific acts, which may pose a threat, but can be mitigated or resolved by appeasement."}, "sentence_type": "Interrogative sentence", "pos": {"nouns": {"Threatened sex": 1, "# And calm down #": 1}, "verbs": {"have": 1, "Yes": 1}, "adjectives": {"Threatened": 1}, "pronouns": {"Here": 1}}}, {"content": "5. Riots, impulses or acts of sabotage", "word_count": 9, "label": "", "sentence_structure": "Subject鈥搗erb鈥揷omplement", "subjective_objective": {"answer": "Subjective", "rationale": "For violent behaviour, impulse or sabotage is the subjective attitude and behaviour of the patient."}, "sentence_sentiment": {"answer": "Negative", "rationale": "Because \"absolute\" and \"exult\" and \"destructive\" are all acts with negative emotional connotations, it is suggested that the person may have such problems and may cause harm or adverse effects to others."}, "language_style": {"answer": "Calm and direct", "rationale": "The entry is more directly linguistically, is not used too much rhetorical or emotionally, and is more objective in tone and more focused on the expression of facts and information. It is therefore possible to judge that language style for this purpose is \"cool and direct\"."}, "time_dimension": {"answer": "Past", "rationale": ""}, "sentence_type": "Interrogative sentence", "pos": {"nouns": {"Riot": 1, "Expedition": 1, "Acts of sabotage": 1}, "adjectives": {"- A violent one": 1, "Exult": 1, "It's destructive": 1}}}, {"content": "II. Optimism", "word_count": 2, "label": "", "sentence_structure": "Subject鈥搗erb鈥揷omplement", "subjective_objective": {"answer": "Subjective", "rationale": "Because optimism is an attitude or feeling, it is the patient ' s own creation and is therefore a subjective approach."}, "sentence_sentiment": {"answer": "Positive", "rationale": "Because \"optimistic\" is often seen as a positive personality character, indicating a positive mindset and a positive view of life."}, "language_style": {"answer": "Calm and direct", "rationale": "The language style of the entry is more straightforward, there is no excessive emotional accoutrements or euphemism, but rather a direct question about the optimism of the other party and a more objective tone."}, "time_dimension": {"answer": "Present", "rationale": "The entry asks the person ' s current emotional state and thus judges it as now."}, "sentence_type": "Imperative sentence", "pos": {"adjectives": {"Optimistic": 1}}}, {"content": "Three, high moods, jokers, laughs.", "word_count": 10, "label": "", "sentence_structure": "Subject鈥搗erb鈥揷omplement", "subjective_objective": {"answer": "Subjective", "rationale": "Because \"float\" and \"joke\" and \"fluencing\" are the patient's own psychological state and performance, and are viewed from the patient's subjective point of view."}, "sentence_sentiment": {"answer": "Positive", "rationale": "Because the entries \"fun\" and \"joke\" and \"smuggle\" are all positive features."}, "language_style": {"answer": "Tactful and warm", "rationale": "The entry is more euphemistic and warm, depicting the emotional state of the person under investigation by using adjectives such as \"high\", \"joke-a-fun\" and \"smuggle-a-fun.\" At the same time, the question or order is not directly raised, but is being discussed in a more friendly tone, which better attracts the participation and cooperation of the person under investigation."}, "time_dimension": {"answer": "Present", "rationale": "The entry describes the person's current behaviour or mentality, and the temporal dimension is now judged."}, "sentence_type": "Affirmative declarative sentence", "pos": {"nouns": {"The mood is high": 1, "A joke": 1, "Smile": 1}, "adjectives": {"Love": 1, "Open": 1, "Easy": 1}, "numerals": {"3": 1}}}, {"content": "Four, it's a clear rise in emotions.", "word_count": 10, "label": "", "sentence_structure": "Subject鈥搗erb鈥揷omplement", "subjective_objective": {"answer": "Subjective", "rationale": "Because high emotions and self-esteem are the emotional experiences of patients, and are subjective observations."}, "sentence_sentiment": {"answer": "Positive", "rationale": "Because \"manifestly high\" and \"floatly self-righteous\" are positive expressions."}, "language_style": {"answer": "Tactful and warm", "rationale": "This entry uses a gentle and inviting tone to describe the possible emotional behaviour of the other party, rather than a direct statement or order."}, "time_dimension": {"answer": "Present", "rationale": "The keywords \"manifestly high\" and \"automated self-interest\" suggest that the entry is a description of the current emotional state of the person being tested. So, the temporal dimension is now."}, "sentence_type": "Imperative sentence", "pos": {"nouns": {"Emotion": 1, "You're a good man": 1}, "verbs": {"Obviously": 1, "High": 1}, "numerals": {"4": 1}}}, {"content": "Five, very high emotional and environmentally unharmonic.", "word_count": 11, "label": "", "sentence_structure": "Subject鈥揷opula鈥損redicative", "subjective_objective": {"answer": "Subjective", "rationale": "Because of the high level of emotion and the coordination of the environment, it is a human inner experience and a subjective feeling."}, "sentence_sentiment": {"answer": "Negative", "rationale": "Because the description \"sexually high, unharmonic\" in the entries is negative."}, "language_style": {"answer": "Tactful and warm", "rationale": "The entry is more adept and interactive when it is spoken in a gentle and inviting manner, rather than by direct statements or orders."}, "time_dimension": {"answer": "Past", "rationale": ""}, "sentence_type": "Affirmative declarative sentence", "pos": {"nouns": {"Emotion": 1, "High": 1, "Environment": 1, "Coordination": 1}, "adverbs": {"Extreme": 1, "not": 1}}}, {"content": "4. Unrealistic exaggerations", "word_count": 10, "label": "", "sentence_structure": "Subject鈥搗erb鈥揷omplement", "subjective_objective": {"answer": "Subjective", "rationale": "Because exaggerating is a subjective idea or attitude."}, "sentence_sentiment": {"answer": "Negative", "rationale": "Because the idea of \"exaggeration\" is negative and not true."}, "language_style": {"answer": "Calm and direct", "rationale": "The entry is based on the following criteria: The question of whether the other party is being asked whether there is an \"unreal and exaggerated notion\" is expressed in an objective and direct manner, without excessive rhetorical or emotional rendering, and in a more objective and balanced manner. This expression is more appropriate for scientific research and investigation."}, "time_dimension": {"answer": "Present", "rationale": "The existence of \"yes\" indicates that it is a survey currently under way, and that \"exaggeration\" refers to an act or attitude that the person who was tested may have shown in completing the questionnaire. The temporal dimension of the article should therefore be judged as now."}, "sentence_type": "Exclamatory sentence", "pos": {"nouns": {"Perceptions": 1, "Exaggeration": 1}, "verbs": {"have": 1, "- Yeah": 1, "not": 1, "Exaggeration": 1}, "adjectives": {"Actual": 1, "Not in line with": 1}, "numerals": {"4": 1}}}, {"content": "Five, a pretense of grandeur that can't be corrected.", "word_count": 10, "label": "", "sentence_structure": "Subject鈥搗erb鈥揷omplement", "subjective_objective": {"answer": "Subjective", "rationale": "Because \"exaggeration\" is a mental state or psychological activity that is the subject of subjective experience and judgement."}, "sentence_sentiment": {"answer": "Negative", "rationale": "Because \"exaggerated delusion\" is a mental illness, which has a negative emotional effect."}, "language_style": {"answer": "Calm and direct", "rationale": "The entry expresses in a straightforward language style the question of whether the person under investigation is over-examining his or her imagination, without too much emotional adornment or euphemism."}, "time_dimension": {"answer": "Past", "rationale": ""}, "sentence_type": "Exclamatory sentence", "pos": {"nouns": {"Exaggerating": 1, "Paranoia": 1}, "verbs": {"have": 1, "Correct": 1}, "adjectives": {"Hard": 1}, "numerals": {"5": 1}}}, {"content": "VIII. Contact", "word_count": 3, "label": "", "sentence_structure": "Subject鈥搗erb鈥揷omplement", "subjective_objective": {"answer": "Subjective", "rationale": "Because \"exposure\" is a subjective feeling and experience of a person, different people may have different definitions and understandings of exposure."}, "sentence_sentiment": {"answer": "Neutral", "rationale": "Because the entry does not express any emotional colour, it only describes a phenomenon."}, "language_style": {"answer": "Tactful and warm", "rationale": "The language style of the entry is more moderate, not too direct or too strong, but rather a kind and natural way of directing the interviewee to think and answer questions. Moreover, the title of the entry uses the word \"exposure\" to suggest that the interviewee needs to share some personal experiences or feelings, which are more likely to trigger the echoes of the interviewee and to make them comfortable and relaxed, thereby making it easier to express his real thoughts and feelings. In general, the \"eight, Touch\" entry guides the interviewee to answer questions with a gentle and intimate touch, a typical \"kind\" language style."}, "time_dimension": {"answer": "Present", "rationale": "The keyword is \"exposure\", which indicates the person's current behaviour or experience."}, "sentence_type": "Interrogative sentence", "pos": {"nouns": {"Exposure": 1}}}, {"content": "1. No symptoms or levels comparable to those of normal patients", "word_count": 17, "label": "", "sentence_structure": "Subject鈥搗erb鈥揷omplement", "subjective_objective": {"answer": "Objective", "rationale": "Because \"no symptoms of this or a level comparable to the normal patient's level\" describes the actual situation of the patient, and does not involve the patient's feelings or subjective judgement."}, "sentence_sentiment": {"answer": "Neutral", "rationale": "Because the entry describes the state of a certain symptom, there is no apparent emotional colour."}, "language_style": {"answer": "Calm and direct", "rationale": "The entry describes the results of a medical test in a concise and clear language, without the use of excessive rhetorical or emotional colours and with a more objective tone."}, "time_dimension": {"answer": "Past", "rationale": "Based on the key words in the input sentence, \"nil\" and \"similar to the normal patient's level\", it can be judged that the entry is a question about behaviour at a certain point in the past, and therefore the temporal dimension is the past."}, "sentence_type": "Affirmative declarative sentence", "pos": {"nouns": {"Symptoms": 1, "Patient": 1, "Horizontal": 1}, "verbs": {"have": 1, "and": 1, "Similar": 1}, "adjectives": {"None": 1}, "adverbs": {"or": 1}, "numerals": {"1": 2}}}, {"content": "Two, a little nosy or a little finger-to-mouth.", "word_count": 13, "label": "", "sentence_structure": "Subject鈥搗erb鈥搊bject", "subjective_objective": {"answer": "Subjective", "rationale": "Because this is a subjective aspect of the patient ' s assessment and description of himself."}, "sentence_sentiment": {"answer": "Negative", "rationale": "Because \"minding\" and \"showing\" are derogatory terms that indicate that the interviewee may have engaged in excessive interference in the affairs of others."}, "language_style": {"answer": "Tactful and warm", "rationale": "By using the terms \"slight\" and \"preferance\", the entry presents this characteristic of the subject of the investigation in a more objective and moderate manner, avoiding direct accusations or criticism and being more friendly and friendly."}, "time_dimension": {"answer": "Present", "rationale": "The entry asks about the current behaviour or state of the subject, and the words \"slightly\" and \"preferably\" indicate that the act may have existed in the past, but it still exists or continues. The temporal dimension therefore determines it now."}, "sentence_type": "Imperative sentence", "pos": {"nouns": {"Mind your own business": 1, "Point and point": 1}, "verbs": {"have": 1}, "adjectives": {"A little": 1}, "numerals": {"2": 1}}}, {"content": "Three, nosy. It's so controversial.", "word_count": 7, "label": "", "sentence_structure": "Subject鈥搗erb鈥搊bject", "subjective_objective": {"answer": "Subjective", "rationale": "The individual attitude and perception of the patient, as he prefers to be idle and to argue well, is a subjective one."}, "sentence_sentiment": {"answer": "Negative", "rationale": "Because \"mind\" and \"good argument\" are all negative emotional acts."}, "language_style": {"answer": "Tactful and warm", "rationale": "The more euphemistic and less direct criticism of the language of the entry, which helps the interviewees to be more willing to answer questions, while avoiding situations in which they feel attacked or offended. Moreover, the terms \"mindful\" and \"good argument\" are used to describe the personality of the interviewee and are neutral expressions that are neither too downplayed nor overapproached."}, "time_dimension": {"answer": "Present", "rationale": "The entry describes a customary act of the testee, \"minding\" and \"good argument\" both describing the current state of behaviour. So it's now."}, "sentence_type": "Negative declarative sentence", "pos": {"verbs": {"Love": 1, "Pipe": 1, "Argument": 1}, "adjectives": {"No, no, no": 1}}}, {"content": "Four, command the command, command the others.", "word_count": 9, "label": "", "sentence_structure": "Subject鈥搗erb鈥搊bject", "subjective_objective": {"answer": "Subjective", "rationale": "Because \"Face, commanding others\" reflects a person's behavioural orientation and character, and is the patient's own subjective feeling and perception."}, "sentence_sentiment": {"answer": "Negative", "rationale": "Because the \"order, command\" act in the entry usually has a negative meaning."}, "language_style": {"answer": "Calm and direct", "rationale": "The language style of the entry is more calm and straightforward, and it directly expresses the subject of a desire to ask the other party whether it prefers to dictate or direct others, and does not use too much acrimonymous language or emotional colour, which is more direct and serious."}, "time_dimension": {"answer": "Present", "rationale": "The entry is an inquiry into the current behaviour of the subject, in which the terms \"order\" and \"director\" indicate that this is an ongoing or frequent act, so the temporal dimension is now judged."}, "sentence_type": "Imperative sentence", "pos": {"nouns": {"Love": 1, "No, no, no": 1, "Order": 1, "Command": 1, "Others": 1}, "verbs": {"Let's go": 1, "Here": 1}}}, {"content": "5. Arbitraryity, incoherence with the environment", "word_count": 8, "label": "", "sentence_structure": "Subject鈥搗erb鈥揷omplement", "subjective_objective": {"answer": "Subjective", "rationale": "This is because the patient ' s assessment and perception of himself or of others is subjective."}, "sentence_sentiment": {"answer": "Negative", "rationale": "Because the descriptions of \"arbitrary\" and \"unconformity with the environment\" have negative emotional aspects."}, "language_style": {"answer": "Calm and direct", "rationale": "The entry directly indicates whether the testee has a tendency to be arbitrary and to relate to the surrounding environment, with a more direct and explicit language style, and without excessive rhetorical or emotional colours."}, "time_dimension": {"answer": "Present", "rationale": ""}, "sentence_type": "Negative declarative sentence", "pos": {"nouns": {"Obsessive": 1, "Environment": 1}, "adjectives": {"Obscene": 1, "Coordination": 1}}}, {"content": "2 25% reduction in sleep time", "word_count": 6, "label": "", "sentence_structure": "Subject鈥搗erb鈥揷omplement", "subjective_objective": {"answer": "Objective", "rationale": "Because the reduction in sleep time is an objective fact of the patient's experience."}, "sentence_sentiment": {"answer": "Negative", "rationale": "Because sleep is harmful to health, it is a negative behaviour."}, "language_style": {"answer": "Calm and direct", "rationale": "The entry is a direct indicator of the extent to which the person was able to sleep in a reduced time, with an objective calm tone and without any use of rhetorical or emotional colours."}, "time_dimension": {"answer": "Present", "rationale": "The entry uses \"reduce\" and \"25%\" to describe the current state, indicating that the testee is experiencing a reduction in sleep time when completing the questionnaire. The time dimension of the article is therefore now."}, "sentence_type": "Imperative sentence", "pos": {"nouns": {"Sleep time": 1, "25%": 1}, "verbs": {"Reduction": 1}, "numerals": {"25": 1}, "classifiers": {"individual": 1}}}, {"content": "3. 50% reduction in sleep time", "word_count": 6, "label": "", "sentence_structure": "Subject鈥搗erb鈥揷omplement", "subjective_objective": {"answer": "Subjective", "rationale": "Because the reduction in the time spent sleeping is the result of subjective judgement by the patient himself, not objective facts."}, "sentence_sentiment": {"answer": "Negative", "rationale": "Because the act of \"50% reduction in sleep time\" is negative and may cause physical health problems."}, "language_style": {"answer": "Calm and direct", "rationale": "The language style of the entry is relatively simple, and it directly indicates a 50 per cent reduction in the time spent sleeping by the subject, without excessive rhetoric or emotional colour. This expression makes the transmission of information clearer and more accurate."}, "time_dimension": {"answer": "Past", "rationale": "The entry is a question about the testee's past behaviour or state, and therefore the test is judged to be \"the past\"."}, "sentence_type": "Negative declarative sentence", "pos": {"nouns": {"Sleep time": 1, "Reduction": 1, "50%": 1}, "verbs": {"Increase": 1}, "numerals": {"50%": 1}}}, {"content": "4 75% reduction in sleep time", "word_count": 6, "label": "", "sentence_structure": "Subject鈥搗erb鈥揷omplement", "subjective_objective": {"answer": "Objective", "rationale": "Because the reduction in sleep time is an objective fact of the patient's actual experience."}, "sentence_sentiment": {"answer": "Negative", "rationale": "Because the \"75% reduction in sleep time\" in the entries is negative."}, "language_style": {"answer": "Calm and direct", "rationale": "The entry is presented with a specific data \"75% reduction in sleep time\" and is straightforward and not overly rhetorical or emotional."}, "time_dimension": {"answer": "Present", "rationale": "The entry describes the person's current behaviour or condition, i.e., the reduction of his sleep time by 75 per cent. We can therefore judge it as now."}, "sentence_type": "Imperative sentence", "pos": {"nouns": {"Sleep time": 1, "Reduction": 1, "Percentage": 1}, "verbs": {"Increase": 1, "Impact": 1}, "numerals": {"75": 1}}}, {"content": "5 - Sleepless all night", "word_count": 4, "label": "", "sentence_structure": "Subject鈥搗erb鈥揷omplement", "subjective_objective": {"answer": "Subjective", "rationale": "According to the information provided, \"night insomnia\" is a subjective feeling and experience of the patient and is therefore subjective in its perspective."}, "sentence_sentiment": {"answer": "Negative", "rationale": "Because the \"night insomnia\" behavior in the entry is negative."}, "language_style": {"answer": "Calm and direct", "rationale": "The entry uses simple and clear terms to describe the sleep conditions of the person under investigation, without too many emotional adorations or rhetorical techniques, and is more calm and straightforward in language style.\""}, "time_dimension": {"answer": "Present", "rationale": "The questionnaire entry asked if the person tested was currently insomnia all night, so the time dimension was now judged."}, "sentence_type": "Imperative sentence", "pos": {"verbs": {"All night long": 1, "Not sleeping": 1}}}, {"content": "2. A slight increase in sexual interest, with some minor rhetoric and action", "word_count": 12, "label": "", "sentence_structure": "Subject鈥搗erb鈥揷omplement", "subjective_objective": {"answer": "Subjective", "rationale": "Because sexual interest and frivolous behaviour are feelings and attitudes of the patient himself, they are subjective."}, "sentence_sentiment": {"answer": "Negative", "rationale": "Because the entry describes a light and unserious attitude, it is negative."}, "language_style": {"answer": "Tactful and warm", "rationale": "The entry is neutral, has no overly negative emotional connotations, and does not directly criticize or accuse the other. Such expressions convey the researcher鈥檚 concern and respect for the person under investigation, and can effectively avoid responses that may provoke resentment or resistance."}, "time_dimension": {"answer": "Present", "rationale": "The keywords are \"a slight increase in sexual interest\" and \"a little act of frivolousness\", all of which refer to the situation of the person being tested. The temporal dimension of the article is therefore now."}, "sentence_type": "Affirmative declarative sentence", "pos": {"nouns": {"Sexual interest": 1, "Words and lines": 1}, "verbs": {"A little bit": 1, "Floating": 1}, "adverbs": {"A little": 1, "Some": 1}}}, {"content": "3. Increased sexual interest, with visible light rhetoric", "word_count": 12, "label": "", "sentence_structure": "Subject鈥搗erb鈥揷omplement", "subjective_objective": {"answer": "Subjective", "rationale": "Because \"sex interest increases\" and \"shows and acts\" are the self-perceptions or attitudes of the patient."}, "sentence_sentiment": {"answer": "Negative", "rationale": "Since the purpose of the article was expressed in relation to obvious frivolous statements and acts, this was usually a negative attitude."}, "language_style": {"answer": "Calm and direct", "rationale": "So I think the language style of the article is \"cool and straight.\""}, "time_dimension": {"answer": "Present", "rationale": "The entry describes the current behaviour or behaviour of the testee, with the keywords \"enhanced sexual interest\" and \"manifestly light words and actions\" pointing to what is happening. Thus, the temporal dimension is now."}, "sentence_type": "Imperative sentence", "pos": {"nouns": {"Sexual interest": 1, "Words and lines": 1}, "verbs": {"Enhanced": 1, "have": 1, "Obviously": 1, "Floating": 1}, "adjectives": {"Obviously": 1, "Floating": 1}, "numerals": {"3": 1}}}, {"content": "4. Significant increase in sexual interest, serious flirtation or aggressive behaviour", "word_count": 21, "label": "", "sentence_structure": "Subject鈥搗erb鈥搊bject", "subjective_objective": {"answer": "Subjective", "rationale": "Because \"sexual interest is significantly increased\", \"seriously molested opposite sex\" and \"showing the wrong words and deeds\" are subjective acts and ideas of patients."}, "sentence_sentiment": {"answer": "Negative", "rationale": "Because it describes bad sexual behaviour and attitudes, it has a clear negative emotional connotation."}, "language_style": {"answer": "Calm and direct", "rationale": "The language style in this entry is more direct and offensive, and may be stressful and uncomfortable to the respondents. Using negative terms such as \"serious\" , \"mix\" and \"showing the wind,\" it displays a strong condemnation and lacks elements of comfort and understanding. This cold language style may affect the authenticity of the findings or the real feedback of the interviewees."}, "time_dimension": {"answer": "Past", "rationale": "The entry describes the sexual interests and behaviour of the person tested in the past and therefore judges the past."}, "sentence_type": "Affirmative declarative sentence", "pos": {"nouns": {"Sexual interest": 1, "Smuggle": 1, "Heterosexual": 1, "Wind": 1}, "verbs": {"Significant increase": 1, "Smuggler": 1, "Words and lines": 1}, "adjectives": {"Serious": 1}, "numerals": {"4": 1}}}, {"content": "5. Focusing on sexual activity throughout the day", "word_count": 8, "label": "", "sentence_structure": "Subject鈥搗erb鈥揷omplement", "subjective_objective": {"answer": "Subjective", "rationale": "Because \"focus\" and \"sex\" are personal feelings and behaviours of the patient, and are subjective in terms of the subject."}, "sentence_sentiment": {"answer": "Negative", "rationale": "Because the \"all day sex\" act in the entry is negative."}, "language_style": {"answer": "Calm and direct", "rationale": "The subject is in plain and simple language, asking the respondents whether they are focused on sex all day, without any rhetorical or emotional rendering, and in a more objective and straightforward way. So we can judge that the language style of the questionnaire entry is calm and straightforward."}, "time_dimension": {"answer": "Present", "rationale": "Now the entry describes the current behaviour or state of the testee, \"wholly\" means that it is a long-term, continuous act, and \"whomever sex\" is a specific description of the act. Thus, the temporal dimension of the entry is now judged."}, "sentence_type": "Imperative sentence", "pos": {"nouns": {"Sexual activity": 1, "All day long": 1}, "verbs": {"Focus": 1, "Conduct": 0}, "adverbs": {"Absolutely": 0, "Total": 0, "Not at all": 0, "Just": 0, "But": 0, "Almost": 0, "Maybe": 0, "Often": 0, "General": 0, "Usually": 0, "Regular": 0, "Sometimes": 0, "It's rare": 0, "Very few": 0, "Not much": 0, "Very little": 0}, "classifiers": {"individual": 1}}}, {"content": "2. A slight decline in the quality of work", "word_count": 8, "label": "", "sentence_structure": "Subject鈥搗erb鈥揷omplement", "subjective_objective": {"answer": "Objective", "rationale": "Because the decline is a quantifiable fact and is an objective observation."}, "sentence_sentiment": {"answer": "Negative", "rationale": "Because the description of \"a slight decline in the quality of work\" indicates a lack of work and a problem, it is a negative expression."}, "language_style": {"answer": "Calm and direct", "rationale": "The language style of the entry is calm and straightforward. It simply raises the question whether the quality of the work of the interviewee has declined, is not overly emotional or rhetorical, and is more objective and serious."}, "time_dimension": {"answer": "Past", "rationale": "The entry describes the changes in the behaviour or state of the person tested over a certain period of time, and \"a slight decline in the quality of the work\" is a description of his past performance and therefore judgement is a past."}, "sentence_type": "Negative declarative sentence", "pos": {"nouns": {"Quality of work": 1}, "verbs": {"Down": 1, "Slight": 1}, "adjectives": {"have": 1, "of": 1}}}, {"content": "3. Significant decline in the quality of work", "word_count": 8, "label": "", "sentence_structure": "Subject鈥搗erb鈥揷omplement", "subjective_objective": {"answer": "Objective", "rationale": "Because the apparent decline in the quality of work is an objective fact that patients experience in their work. Even if patients鈥?subjective feelings may influence their judgment, the impact can still be considered to be based on objective evidence."}, "sentence_sentiment": {"answer": "Negative", "rationale": "Because the entry \"manifestly declining quality of work\" represents a negative situation, indicating problems or errors at work. The purpose of the article is therefore negative."}, "language_style": {"answer": "Calm and direct", "rationale": "The language style of the entry is calmer and more direct, with no excessive adoration or emotional colour, but rather is a straightforward question, expressing concern and concern about the quality of the interviewee鈥檚 work. This tone is more objective and serious and can attract attention and reflection from the interviewee."}, "time_dimension": {"answer": "Past", "rationale": "The entry describes changes in the quality of work that take time to become apparent and should therefore be classified as \"the past\"."}, "sentence_type": "Imperative sentence", "pos": {"nouns": {"Quality of work": 1, "Down": 1}, "verbs": {"Obviously": 1, "Down": 1}, "adjectives": {"Obviously": 1}}}, {"content": "4. Impossible to continue working or to attend activities in hospitals for hours", "word_count": 20, "label": "", "sentence_structure": "Subject鈥搗erb鈥揷omplement", "subjective_objective": {"answer": "Subjective", "rationale": "Because \"not working\" and \"not working for hours\" are subjective feelings that are described from the point of view of the patient."}, "sentence_sentiment": {"answer": "Negative", "rationale": "Because the descriptions of \"not working\" and \"not having been able to attend for hours in the hospital\" have some negative mood."}, "language_style": {"answer": "Calm and direct", "rationale": "This entry describes the working and physical state of the person under investigation in a concise language, which does not involve too much emotional rendering or accoutrements."}, "time_dimension": {"answer": "Past", "rationale": ""}, "sentence_type": "Negative declarative sentence", "pos": {"nouns": {"Cannot": 1, "Work": 1, "Hospital": 1, "Activities": 1, "Hours": 1}, "verbs": {"Go on": 1, "Participation": 1, "Yes": 1}, "adverbs": {"Jean": 1}, "numerals": {"4": 1}}}, {"content": "5. No day-to-day activities are self-cared or not available for ward activities", "word_count": 17, "label": "", "sentence_structure": "Subject鈥搗erb鈥揷omplement", "subjective_objective": {"answer": "Objective", "rationale": "Since self-care and participation in activities is a real condition of the patient, a fact that can be observed, it is an objective examination."}, "sentence_sentiment": {"answer": "Negative", "rationale": "Because this entry represents physical limitations and negative situations of exclusion from social activities."}, "language_style": {"answer": "Calm and direct", "rationale": "The entry uses a direct and clear language to describe the subject, i.e. whether the person tested can take care of himself or participate in the activity of the ward in his daily life."}, "time_dimension": {"answer": "Present", "rationale": "The keywords \"day activities\" and \"involvement\" and \"involvement in ward activities\" all indicate the condition of the person being tested, so the entry describes the situation."}, "sentence_type": "Negative declarative sentence", "pos": {"nouns": {"Daily activities": 1, "Ward activities": 1}, "verbs": {"No, I can't": 2, "Self-regulating": 1, "Participation": 1}, "numerals": {"5": 1}}}, {"content": "2. Improper or suspicious", "word_count": 7, "label": "", "sentence_structure": "Subject鈥損redicate", "subjective_objective": {"answer": "Subjective", "rationale": "Because \"false\" is a subjective perception deviation and a false belief, and the extent of \"situated or suspicious\" is also perceived and assessed by the patient himself, this part is examined from the patient's subjective point of view."}, "sentence_sentiment": {"answer": "Negative", "rationale": "Because the idea in the entry is negative."}, "language_style": {"answer": "Calm and direct", "rationale": "The entry uses a direct language style, without too many rhetorical or emotional renderings, and simply raises the question \"Is there a delusion?\" This language style is more objective and cold, and suitable for psychological survey purposes, which requires a critical and precise description."}, "time_dimension": {"answer": "Present", "rationale": "Now the entry describes the current situation of the person being tested, and the keyword \"specified or suspicious\" indicates that it is a description of a state of behaviour or of mind, with no clear time pointing at it. It can therefore be judged that the entry is for the purpose of questioning and evaluating the current state of conduct or state."}, "sentence_type": "Negative declarative sentence", "pos": {"nouns": {"Paranoia": 1, "Suspected": 1}, "verbs": {"I do": 1}, "adverbs": {"or": 1}}}, {"content": "Three, delusional certainty. Emotional interpretation.", "word_count": 10, "label": "", "sentence_structure": "Subject鈥搗erb鈥搊bject", "subjective_objective": {"answer": "Subjective", "rationale": "According to the description given, delusions are certainly the patient's own thoughts and beliefs, and are subjective; they can be interpreted emotionally, and they are based on an objective perspective by observing and understanding the emotional changes of the patient. The answer is therefore: \"subjective/objective\"."}, "sentence_sentiment": {"answer": "Neutral", "rationale": "Because the entry does not have any emotional color."}, "language_style": {"answer": "Tactful and warm", "rationale": "The entry uses more moderate language, such as \"presumptuous positive\" and \"available emotional interpretation,\" which attempts to guide respondents to think openly, rather than directly denying or confirming. This expression is more personal and interactive and helps to create a good interview climate."}, "time_dimension": {"answer": "Present", "rationale": ""}, "sentence_type": "Affirmative declarative sentence", "pos": {"nouns": {"Paranoia": 1, "Sure": 1, "Emotion": 1}, "verbs": {"Available": 1, "Explanation": 1}, "numerals": {"3": 1}}}, {"content": "Four, delusional. It's hard to explain.", "word_count": 11, "label": "", "sentence_structure": "Subject鈥搗erb鈥搊bject", "subjective_objective": {"answer": "Objective", "rationale": "Because delusion is an objective fact that patients experience, emotional experience and interpretation are subjective."}, "sentence_sentiment": {"answer": "Negative", "rationale": "Because the \"false certainty\" attitude in the entries is negative."}, "language_style": {"answer": "Calm and direct", "rationale": "The entry directly expresses a question about the other party's delusions, and points out that they may be difficult to interpret with emotion. This expression is more direct and objective, does not have too many rhetorical techniques or emotional renderings, and therefore can be judged as \"cool and straight\"."}, "time_dimension": {"answer": "Present", "rationale": "According to the key words and sentence structure, the words \"false\", \"yes\", \"unable to interpret\" are all related to the individual's mental, sensory, etc. and express the current state or situation, and therefore the judgement is now."}, "sentence_type": "Interrogative sentence", "pos": {"nouns": {"Paranoia": 1, "Explanation": 1}, "verbs": {"Sure": 1, "Use": 1, "Hard": 1}, "adjectives": {"Hard": 1}, "adverbs": {"Use": 1, "I'm sure": 1}, "numerals": {"4": 1}}}, {"content": "Five, delusional delusions.", "word_count": 7, "label": "", "sentence_structure": "Subject鈥搗erb鈥搊bject", "subjective_objective": {"answer": "Subjective", "rationale": "\"The delusional delusions\" are made of feelings and ideas that patients experience and are therefore subjective."}, "sentence_sentiment": {"answer": "Negative", "rationale": "Because \"the delusional delusion\" is usually considered a psychiatric symptom with negative connotations."}, "language_style": {"answer": "Calm and direct", "rationale": "The entry specifically asked the respondents whether they had been suffering from delusional delusions, without using any accommodative language or emotional colours, and with a speech temperature and directness."}, "time_dimension": {"answer": "Present", "rationale": "The keywords are \"arise\" and \"fantasy delusions\", which describe the current state or situation and can therefore be judged as the present."}, "sentence_type": "Affirmative declarative sentence", "pos": {"nouns": {"Imagination": 1, "Paranoia": 2}, "numerals": {"5": 1}}}]}

{"questionnaire_title": "Hamilton Axiety Sheet (HAMA)", "questionnaire_category": "Mental health", "item_count": 14, "emotion_shift_overall": "No obvious trend", "language_style_overall": "Calm and direct", "items": [{"content": "1. Anxious situations (always worried about what was going to happen)", "word_count": 14, "label": "", "sentence_structure": "Subject鈥損redicate", "subjective_objective": {"answer": "Subjective", "rationale": "Because it's the patient's subjective feeling and the idea."}, "sentence_sentiment": {"answer": "Negative", "rationale": "Because \"anxiety\" and \"awesome of what's going to happen\" are negative emotional expressions in the entries."}, "language_style": {"answer": "Calm and direct", "rationale": "The entry directly asks the other side whether there is anxiety, does not use over-modified or emotional expressions and is more objective and cold."}, "time_dimension": {"answer": "Present", "rationale": "Now the entry asks the subject about the current emotional state, the key words being \"anxiety\" and \"always fear what will happen\", all pointing to the current thinking and feeling. So the time dimension of the questionnaire is now judged."}, "sentence_type": "Interrogative sentence", "pos": {"nouns": {"Anxious state": 1, "I'm worried": 1, "Situation": 1}, "verbs": {"Come on": 1}, "adverbs": {"Total": 1}}}, {"content": "2. Tension (high muscle tension, tremors, unrelaxion)", "word_count": 12, "label": "", "sentence_structure": "Subject鈥搗erb鈥揷omplement", "subjective_objective": {"answer": "Objective", "rationale": "As stress is a physical reaction or behaviour that can be observed, it is the objective state of the patient."}, "sentence_sentiment": {"answer": "Negative", "rationale": "Because the descriptions of \"stretch\" and \"were not relaxed\" in the entries are negative, suggesting that people may be disturbed and discomfort in certain circumstances."}, "language_style": {"answer": "Calm and direct", "rationale": "The entry describes the tense features in an objective and concise manner, with no excessive emotional rendering or rhetorical techniques, and is more straightforward in tone."}, "time_dimension": {"answer": "Present", "rationale": "The entry describes the physiological responses of the person who was tested in reply to the questions, i.e. \"strained\", \"high muscle tension\" and \"shocking\", all of which are related to the current emotional state. Thus, the basis of the judgment is \"now\"."}, "sentence_type": "Affirmative declarative sentence", "pos": {"nouns": {"Questionnaire": 1, "Entry": 1, "Nervous": 1, "Fiber tension": 1, "Wreck": 1, "Relax": 1}, "verbs": {"Analysis": 1, "High muscle tension": 1, "Wreck": 1, "No, I can't": 1, "Relax": 1}, "numerals": {"2": 1}}}, {"content": "3. Fear (dark, solitary, car, crowds)", "word_count": 11, "label": "", "sentence_structure": "Subject鈥搗erb鈥搊bject", "subjective_objective": {"answer": "Subjective", "rationale": "Because fear is a subjective feeling and emotional experience. While these situations may trigger a reaction of fear, fear is itself a subjective internal experience, not an objective fact or result."}, "sentence_sentiment": {"answer": "Negative", "rationale": "Because \"fear\" is a negative emotion."}, "language_style": {"answer": "Tactful and warm", "rationale": "Warm and warm."}, "time_dimension": {"answer": "Present", "rationale": "The entry asks if the person tested has a fear of something, and the time dimension is now judged."}, "sentence_type": "Negative declarative sentence", "pos": {"nouns": {"Fear": 1, "Darkness": 1, "Alone": 1, "- By car": 1, "There's a lot of people": 1}, "verbs": {"Scared": 1}, "numerals": {"3": 1}}}, {"content": "4. Insomnia", "word_count": 2, "label": "", "sentence_structure": "Subject鈥損redicate", "subjective_objective": {"answer": "Subjective", "rationale": "Because insomnia is a subjective experience and feeling, it is the sleep problems of the patient and the subjective feelings of the individual."}, "sentence_sentiment": {"answer": "Negative", "rationale": "Because insomnia is a negative physical discomfort."}, "language_style": {"answer": "Calm and direct", "rationale": "The entry is more directly linguistically, with no excessive rhetorical or emotional rendering, and is more objective in terms of the insomnia."}, "time_dimension": {"answer": "Present", "rationale": "The keyword \"insomnia\" indicates the current problem of the testee, and the temporal dimension is now judged."}, "sentence_type": "Interrogative sentence", "pos": {"nouns": {"Insomnia": 1}}}, {"content": "5. Memory or Discretion", "word_count": 7, "label": "", "sentence_structure": "Subject鈥搗erb鈥揷omplement", "subjective_objective": {"answer": "Subjective", "rationale": "Because memory or attention impairment is the patient ' s subjective experience and feelings."}, "sentence_sentiment": {"answer": "Negative", "rationale": "Because memory or attention impairment is a negative picture, it means that individuals have problems with cognitive abilities."}, "language_style": {"answer": "Tactful and warm", "rationale": "Warm and warm."}, "time_dimension": {"answer": "Present", "rationale": "The entry describes the question of whether the person tested currently has memory or attention impairments and therefore is judged to be \"now\"."}, "sentence_type": "Declarative sentence", "pos": {"nouns": {"Memory": 1, "Attention": 1, "Obstacles": 1}, "verbs": {"Come on": 1, "have": 1}, "numerals": {"5": 1}, "classifiers": {"individual": 1}}}, {"content": "6. Depression", "word_count": 4, "label": "", "sentence_structure": "Subject鈥搗erb鈥搊bject", "subjective_objective": {"answer": "Subjective", "rationale": "Because depression is the patient's subjective emotional experience."}, "sentence_sentiment": {"answer": "Negative", "rationale": "Because \"depressive\" is a negative psychological state."}, "language_style": {"answer": "Calm and direct", "rationale": "This is a more straightforward language style, which explicitly asks the respondents whether they are depressed, and do not use excessive rhetorical techniques or emotional rendering."}, "time_dimension": {"answer": "Present", "rationale": "The entry can be judged by the time term \"now\" in the sentence to be the current depression of the person being tested and therefore the time dimension of the present."}, "sentence_type": "Interrogative sentence", "pos": {"nouns": {"It's a depression": 1}}}, {"content": "7. Symptoms of the muscle system", "word_count": 6, "label": "", "sentence_structure": "Subject鈥搗erb鈥揷omplement", "subjective_objective": {"answer": "Subjective", "rationale": "Because \"muscular system symptoms\" are a subjective experience or feeling, patients may experience and feel differently."}, "sentence_sentiment": {"answer": "Neutral", "rationale": "Because the entry describes symptoms of a physical system and does not involve any emotional colour."}, "language_style": {"answer": "Tactful and warm", "rationale": "The entry, which guides the respondents to answer questions by asking questions, does not use strong tone or command language style, making the respondents more comfortable and natural in their answers, reflecting a caring attitude."}, "time_dimension": {"answer": "Present", "rationale": "The entry asks if the person tested is currently suffering from muscle system symptoms and is an inquiry into the current situation, so the temporal dimension is now judged."}, "sentence_type": "Interrogative sentence", "pos": {"nouns": {"Muscle system": 1, "Symptoms": 1}}}, {"content": "Perceptive system symptoms", "word_count": 6, "label": "", "sentence_structure": "Subject鈥搗erb鈥揷omplement", "subjective_objective": {"answer": "Subjective", "rationale": "Because the symptoms of the sensory system are the subjective experience and feelings of the patient."}, "sentence_sentiment": {"answer": "Neutral", "rationale": "Because the entry does not have any emotional color."}, "language_style": {"answer": "Calm and direct", "rationale": "The entry describes a subject in a concise manner, i.e. the symptoms of the sensory system. It does not use excessive rhetorical or emotional rendering, is more objective in tone, directly addresses the question to be asked, and does not use words such as too qualifier or emotional adjectives to give people a calm, rational sense."}, "time_dimension": {"answer": "Present", "rationale": "The keyword is \"the symptoms of the sensory system\", which describes a current feeling or state, so the temporal dimension is now judged."}, "sentence_type": "Interrogative sentence", "pos": {"nouns": {"I feel the symptoms of the system": 1}}}, {"content": "Symptoms of the cardiovascular system", "word_count": 7, "label": "", "sentence_structure": "Subject鈥搗erb鈥搊bject", "subjective_objective": {"answer": "Objective", "rationale": "Because \"the symptoms of the cardiovascular system\" are a specific physical reaction or a physical indication, they are objective facts and are not influenced by the patient's subjective consciousness."}, "sentence_sentiment": {"answer": "Neutral", "rationale": "This entry describes a physical symptom and does not express positive or negative emotional expressions."}, "language_style": {"answer": "Calm and direct", "rationale": "The entry directly asked the respondents whether they were suffering from cardiovascular system symptoms, were clear in language, did not have too many emotional renderings or rhetorical techniques and met the objectivity and usefulness of the questionnaire."}, "time_dimension": {"answer": "Past", "rationale": "The entry asks if the person tested has symptoms of a cardiovascular system and the temporal dimension is a past."}, "sentence_type": "Interrogative sentence", "pos": {"nouns": {"Cardiovascular system": 1, "Symptoms": 1}}}, {"content": "10. Respiratory symptoms", "word_count": 6, "label": "", "sentence_structure": "Subject鈥搗erb鈥揷omplement", "subjective_objective": {"answer": "Objective", "rationale": "Since respiratory symptoms are a physiological phenomenon, they can be observed and assessed through, for example, medical examinations, and are examined from an objective perspective."}, "sentence_sentiment": {"answer": "Neutral", "rationale": "Because the entry describes only one aspect and does not involve any emotional colour."}, "language_style": {"answer": "Calm and direct", "rationale": "The language style of the entry is more direct and clear, with no excessive adoration or emotional colour, but simply asks the person surveyed whether he or she has respiratory symptoms."}, "time_dimension": {"answer": "Present", "rationale": "The entry can be seen in the keyword \"respiratory symptoms\" and therefore the temporal dimension is now judged by asking the person tested whether he or she is in a state of respiratory discomfort or illness."}, "sentence_type": "Interrogative sentence", "pos": {"nouns": {"Respiratory system": 1, "Symptoms": 1}, "numerals": {"10": 1}}}, {"content": "11: gastrointestinal symptoms", "word_count": 5, "label": "", "sentence_structure": "Subject鈥搗erb鈥揷omplement", "subjective_objective": {"answer": "Subjective", "rationale": "As gastrointestinal symptoms are subjective feelings and experiences of patients, they are not objective facts."}, "sentence_sentiment": {"answer": "Negative", "rationale": "Because \"temperature intestinal symptoms\" are often considered negative health problems."}, "language_style": {"answer": "Tactful and warm", "rationale": ""}, "time_dimension": {"answer": "Present", "rationale": "The entry is not clearly timed, but it describes a current physical condition or experience, namely \"gastrointestinal symptoms\", and therefore the temporal dimension is now judged."}, "sentence_type": "Interrogative sentence", "pos": {"nouns": {"Stomach": 1, "Symptoms": 1}, "numerals": {"11": 1}}}, {"content": "12. Symptoms of the genitourinary system", "word_count": 8, "label": "", "sentence_structure": "Subject鈥搗erb鈥揷omplement", "subjective_objective": {"answer": "Subjective", "rationale": "Since the entry asks whether the patient has specific symptoms of the urinary system, requiring self-reporting and feelings of the patient, it is subjective."}, "sentence_sentiment": {"answer": "Negative", "rationale": "Because symptoms of the genitourinary system are usually associated with health problems, they may involve negative factors such as disease or pain."}, "language_style": {"answer": "Calm and direct", "rationale": "The entry starts with a number \"12\" and clearly indicates that the specific subject of the survey is \"Symptoms of the genitourinary system\". The subsequent description is clear and concise, with no excessive rhetorical or emotional rendering, and the language temperature and objectivity. The language style of the article can therefore be judged as being calm and straightforward."}, "time_dimension": {"answer": "Present", "rationale": "The entry is an inquiry into the testee ' s presence of symptoms of the genitourinary system, and does not address past, present or future temporal dimensions, and therefore cannot be judged."}, "sentence_type": "Interrogative sentence", "pos": {"nouns": {"Reproduction of the urine system": 1, "Symptoms": 1}, "verbs": {"have": 1}, "numerals": {"12": 1}}}, {"content": "13. Vegetative neurotic symptoms", "word_count": 6, "label": "", "sentence_structure": "Subject鈥搗erb鈥揷omplement", "subjective_objective": {"answer": "Objective", "rationale": "Since a plant neurotic condition is a series of symptoms resulting from functional disorders in the autonomous nervous system in a person, it is an objective reaction of the patient鈥檚 body."}, "sentence_sentiment": {"answer": "Neutral", "rationale": "Because the entry is only a description of a phenomenon, it does not express positive or negative emotional tendencies."}, "language_style": {"answer": "Calm and direct", "rationale": "The entry directly expresses the subject of asking the other party whether there are plant neurotic symptoms, without excessive rhetorical or emotional rendering, and is more objective in tone."}, "time_dimension": {"answer": "Future", "rationale": "The term \"vegetative neurotic symptoms\" is used in the entry, indicating that it is a description of the future, not a state of the past or present. The answer is therefore \"the future\"."}, "sentence_type": "Interrogative sentence", "pos": {"nouns": {"Plant neurotic symptoms": 1}, "numerals": {"13": 1}}}, {"content": "14. Unusual behaviour during talks", "word_count": 7, "label": "", "sentence_structure": "Subject鈥搗erb鈥揷omplement", "subjective_objective": {"answer": "Subjective", "rationale": "Because \"behaviour abnormality\" is determined by observation and assessment, this judgement is often based on the subjective experience and judgement of researchers. The perspective of this article is therefore subjective."}, "sentence_sentiment": {"answer": "Negative", "rationale": "Because \"behaviour abnormal\" is a negative description that may imply psychological problems or abnormal behaviour."}, "language_style": {"answer": "Calm and direct", "rationale": "The entry describes the behaviour of the person under investigation during the talks in a concise and clear manner, without too much adoration or emotional colour, and with a more calm and straightforward language style."}, "time_dimension": {"answer": "Past", "rationale": "The time dimension is uncertain whether it is past, present or future, and further information is needed to judge. If a specific time frame is provided in the title (such as \"a recent meeting\" or \"a meeting that will occur every time\"), it can be more accurately judged whether the entry describes past, present or future conduct."}, "sentence_type": "Affirmative declarative sentence", "pos": {"nouns": {"Meetings": 1, "Behaviour": 1, "Unusual": 1}, "adverbs": {"Yes": 1}}}]}

{"questionnaire_title": "Marriage style survey", "questionnaire_category": "Behavioral questionnaire", "item_count": 18, "emotion_shift_overall": "From negative to positive", "language_style_overall": "Tactful and warm", "items": [{"content": "1 To rule the other side for the parties", "word_count": 10, "label": "", "sentence_structure": "Subject鈥搗erb鈥搊bject", "subjective_objective": {"answer": "Subjective", "rationale": "Since domination and decision-making are the views or behaviour of the patient, they are considered subjectively."}, "sentence_sentiment": {"answer": "Negative", "rationale": "The basis for the judgement: the act of \"dominating the other\" in the entry is negative, as it implies an attitude of unequal power and lack of respect."}, "language_style": {"answer": "Calm and direct", "rationale": "This is more in line with the psychological questionnaire."}, "time_dimension": {"answer": "Present", "rationale": ""}, "sentence_type": "Imperative sentence", "pos": {"nouns": {"Rule": 1, "Both sides": 1, "Decision": 1}, "verbs": {"Do it": 1}}}, {"content": "Two, let the other make his own decisions.", "word_count": 9, "label": "", "sentence_structure": "Subject鈥搗erb鈥搊bject", "subjective_objective": {"answer": "Subjective", "rationale": "Because \"leting the other party make a decision for itself\" is a willingness or expectation that needs to be assessed according to the patient's own feelings and thoughts, and is a subjective examination."}, "sentence_sentiment": {"answer": "Negative", "rationale": "Because the act of \"letting the other party make a decision for itself\" shows a dependent and passive attitude, lacking self-advocacy and independent thinking."}, "language_style": {"answer": "Tactful and warm", "rationale": "The entry follows a mild question-and-answer approach and expresses the intention to have the other party make a decision, with a more natural tone, and with no too direct or command-style expression. At the same time, the use of the term \"you\" as the dominant word, which emphasizes the interest and respect of the other party, makes the questionnaire more personal and interactive."}, "time_dimension": {"answer": "Present", "rationale": "\"Letting the other party make a decision for itself\" is a state of present, emerging psychological state or behaviour, since the title does not refer to past or future time references."}, "sentence_type": "Imperative sentence", "pos": {"nouns": {"The other side": 1, "self": 1, "Decision": 1}, "verbs": {"Jean": 1, "Do it": 1}, "adverbs": {"Down": 1}}}, {"content": "The parties discuss and decide on a regular basis, and can disagree and seek compromise", "word_count": 20, "label": "", "sentence_structure": "Subject鈥搗erb鈥搊bject", "subjective_objective": {"answer": "Subjective", "rationale": "Because \"to decide after frequent discussion\", \"to disagree\", \"to seek compromise\" are patients' views and attitudes to the decision-making process, and are therefore subjective in nature."}, "sentence_sentiment": {"answer": "Positive", "rationale": "Because the conduct expressed in the entry is the search for cooperation and compromise, it is a positive attitude and behaviour."}, "language_style": {"answer": "Tactful and warm", "rationale": "The words \"both parties\" and \"decision after discussion\" are used to be more moderate and euphemistic. At the same time, the phrase \"may not agree, seek compromise\" reflects respect for each other's views and opinions and an attitude of equal consultation. This euphemism is more acceptable and cooperative."}, "time_dimension": {"answer": "Present", "rationale": "In conclusion, the entry is a mixture of \"now\" and \"for the future\"."}, "sentence_type": "Imperative sentence", "pos": {"nouns": {"Both sides": 1, "Decision": 1, "I disagree": 1, "Compromise": 1}, "verbs": {"Discussion": 1, "Do it": 1, "Seek": 1}, "adverbs": {"Regular": 1}, "numerals": {"3": 1}}}, {"content": "2 usually makes the needs of the other party the first to be met.", "word_count": 14, "label": "", "sentence_structure": "Subject鈥搗erb鈥揷omplement", "subjective_objective": {"answer": "Subjective", "rationale": "Because \"usually\" and \"first\" both indicate that this is a patient's tendency or attitude, not an objective fact."}, "sentence_sentiment": {"answer": "Positive", "rationale": "Because it expresses a willingness to help and care for others, it is a positive attitude."}, "language_style": {"answer": "Tactful and warm", "rationale": "This entry asks the respondent, in a caring and respectful manner, whether he or she will normally give priority to the needs of others, the temperature of the language and the sensitivity of the person concerned, and reflects a caring and respectful attitude."}, "time_dimension": {"answer": "Present", "rationale": "Now the keyword \"usually\" indicates that it is a customary act, while \"the need to satisfy the other\" emphasizes the effect and importance of such an act on the present. So the entry asks about the testee ' s current behavioral habits, and the temporal dimension is now."}, "sentence_type": "Imperative sentence", "pos": {"nouns": {"Questionnaire": 1, "Entry": 1, "The other side": 1, "Yes": 1}, "verbs": {"鈾?And I'm gonna be 鈾?: 1, "Got it": 1, "Satisfied": 1}, "adjectives": {"Usually": 1}, "pronouns": {"Here": 1}}}, {"content": "3 Efforts to balance the needs of the parties", "word_count": 9, "label": "", "sentence_structure": "Subject鈥搗erb鈥揷omplement", "subjective_objective": {"answer": "Subjective", "rationale": "Because \"Effort to balance the needs of both sides\" is a subjective attitude or behaviour that reflects a strategy or way in which patients deal with relationships. While the results of such behaviour may be objective (e.g., successful demand for both sides), the issue is more concerned with patients' subjective feelings and ideas."}, "sentence_sentiment": {"answer": "Neutral", "rationale": "Because the entry does not have any emotional color."}, "language_style": {"answer": "Tactful and warm", "rationale": "The entry is more euphemistic, using the words \"balanced\" and \"needs of both parties\" to express concern and respect for the needs of both sides in a relationship, rather than directly identifying the needs of one side. At the same time, \"work\" also expresses positive attitudes and willingness to feel caring and supportive. Such language styles are more acceptable and understandable and can create a good atmosphere of communication."}, "time_dimension": {"answer": "Present", "rationale": "The entry asks the testee how to deal with the needs of both parties in the relationship, and therefore concerns the current and future time dimensions. In it, the term \"work\" indicates that this is an ongoing act and is therefore the current state of affairs. And \"balance the needs of both parties\" means considering and planning for the situation that may be encountered in the future, and therefore includes the future time dimension."}, "sentence_type": "Imperative sentence", "pos": {"nouns": {"Try": 1, "Both sides": 1, "Yes": 1}, "verbs": {"Balance": 1}, "adverbs": {"3": 1, "Down there": 1, "Please": 1, "Analysis": 1, "This": 1, "Questionnaire": 1, "Entry": 1}, "numerals": {"3": 1}}}, {"content": "One needs his needs to be met, and he feels he has the right to do it.", "word_count": 20, "label": "", "sentence_structure": "Subject鈥搗erb鈥揷omplement", "subjective_objective": {"answer": "Subjective", "rationale": "This is a perception and assessment of the patient as such, and is a subjective one."}, "sentence_sentiment": {"answer": "Positive", "rationale": "Because \"to satisfy your needs\" and \"to feel empowered to do so\" in the entry are positive expressions, positive emotional expressions."}, "language_style": {"answer": "Tactful and warm", "rationale": "The entry uses positive and encouraging language to express the importance of enabling individual needs to be met and to emphasize the right of the individual to do so, which is more accommodative."}, "time_dimension": {"answer": "Present", "rationale": ""}, "sentence_type": "Imperative sentence", "pos": {"nouns": {"self": 1, "Yes": 1, "Powers": 1}, "verbs": {"Got it": 1, "Satisfied": 1, "Feel": 1, "Do it": 1}, "adverbs": {"Jean": 1}, "pronouns": {"Here": 1}}}, {"content": "Three trying to figure out how both sides feel.", "word_count": 10, "label": "", "sentence_structure": "Subject鈥搗erb鈥揷omplement", "subjective_objective": {"answer": "Subjective", "rationale": "Because \"know your feelings\" is a subjective judgment and behavior that requires the patient to understand and sense."}, "sentence_sentiment": {"answer": "Positive", "rationale": "Because the purpose of the entry is to \"work to find out how they feel\" and it shows the willingness of the interviewees to spend time and energy to understand and resolve their problems with others."}, "language_style": {"answer": "Tactful and warm", "rationale": "The entry is more personal and interactive when it is spoken in a gentle and inviting tone, rather than in direct statements or orders. At the same time, the word \"work\" implies that it is a process that requires both parties to work together, not a unilateral responsibility or requirement. Overall, the questionnaire is more nuanced in its language style."}, "time_dimension": {"answer": "Past", "rationale": "Since these terms do not specify a specific time period or frequency, it is not possible to determine whether they are past, present or future. If more information is provided in the context, then the time frame involved in this entry may be judged. However, based on the information available, it can only be established that this is a neutral description of the behaviour, without any apparent temporal characteristics."}, "sentence_type": "Imperative sentence", "pos": {"nouns": {"Try": 1, "Both sides": 1, "Feel": 1}, "verbs": {"I'm sure": 1}, "adverbs": {"Down there": 1, "Please": 1, "Analysis": 1}, "numerals": {"3": 1}}}, {"content": "One is not sensitive to each other's needs and feelings.", "word_count": 13, "label": "", "sentence_structure": "Subject鈥搗erb鈥揷omplement", "subjective_objective": {"answer": "Subjective", "rationale": "Because it is a subjective judgement that patients feel and feel about themselves."}, "sentence_sentiment": {"answer": "Negative", "rationale": "Because the act of \"less sensitive to the needs and feelings of the other person\" is not sensitive to others' feelings, it can cause alienation."}, "language_style": {"answer": "Calm and direct", "rationale": "The entry is a direct expression of the subject of asking the other party whether it is not sufficiently sensitive to the other party ' s needs and feelings in dealing with human relationships, and is not based on too much rhetorical or emotional presentation, which is more objective and balanced. It uses simple sentences and words, clearly conveys information and avoids emotional expressions."}, "time_dimension": {"answer": "Present", "rationale": "The keyword \"unsensitive\" suggests that the act may have been long-term, not only past or future. Moreover, the word \"the needs and feelings of the other side\" suggests that it is a matter of interpersonal relations, so the temporal dimension is now judged."}, "sentence_type": "Imperative sentence", "pos": {"nouns": {"The other side": 1, "Yes": 1, "Feel": 1}, "verbs": {"to": 1, "not": 1, "Too much": 1, "Sensitive": 1}, "adverbs": {"Not really": 1}, "numerals": {"1": 2}, "pronouns": {"I": 1}}}, {"content": "Two is sensitive to each other's needs and feelings.", "word_count": 12, "label": "", "sentence_structure": "Subject鈥搗erb鈥揷omplement", "subjective_objective": {"answer": "Subjective", "rationale": "Because it involves the patient ' s understanding and concern for the needs and feelings of others, it is the patient ' s subjective attitude and behaviour."}, "sentence_sentiment": {"answer": "Positive", "rationale": "Because the behaviour of \"sensitive to the needs and feelings of the other\" in the entry reflects a caring attitude towards others and is a positive emotional aspect."}, "language_style": {"answer": "Tactful and warm", "rationale": "The term \"sensitive\" is used in this entry, conveying a caring, caring emotional colour, with a less verbish tone, without direct orders or criticism, and therefore a warmer language style."}, "time_dimension": {"answer": "Present", "rationale": "The words \"yes,\" \"very\" and \"sensitive\" in \"sensitive to the needs and feelings of the other\" are all expressions of an ongoing act or state, indicating that the act is a state of conduct. Thus, the temporal dimension of this entry is now judged."}, "sentence_type": "Imperative sentence", "pos": {"nouns": {"Questionnaire": 1, "Entry": 1, "The other side": 1, "Yes": 1, "Feel": 1}, "verbs": {"to": 1, "Sensitive": 1}, "numerals": {"2": 1}}}, {"content": "One usually knows what he wants.", "word_count": 12, "label": "", "sentence_structure": "Subject鈥揷opula鈥損redicative", "subjective_objective": {"answer": "Subjective", "rationale": "Because this is a subjective assessment of self-awareness by patients."}, "sentence_sentiment": {"answer": "Positive", "rationale": "Because the \"know what you want\" attitude is positive."}, "language_style": {"answer": "Tactful and warm", "rationale": "The entry uses a soft, invitation-style tone for dialogue rather than direct statements or orders, which is more personal and interactive. At the same time, the language style of the article is more relaxed and gives a positive sense of direction, which helps to increase the motivation of respondents to participate in the questionnaire."}, "time_dimension": {"answer": "Present", "rationale": "The words \"normally\" and \"more clearly\" in the entry indicate normal, customary behaviour or state, which suggests that this is a situation that is often present in the daily lives of the person being tested, and therefore the temporal dimension is now judged."}, "sentence_type": "Affirmative declarative sentence", "pos": {"nouns": {"self": 1, "To": 1, "I'm thinking": 1}, "verbs": {"Clear": 1}, "adverbs": {"Usually": 1}, "numerals": {"1": 1}}}, {"content": "Two usually knows what the other person wants.", "word_count": 12, "label": "", "sentence_structure": "Subject鈥揷opula鈥損redicative", "subjective_objective": {"answer": "Subjective", "rationale": "Because \"know what the other person wants\" is a subjective feeling or perception, depending on the patient's subjective observation and understanding."}, "sentence_sentiment": {"answer": "Positive", "rationale": "Because \"know what the other side wants\" is a positive attitude or quality that indicates an understanding of the needs and expectations of others."}, "language_style": {"answer": "Tactful and warm", "rationale": "The entry is more personal and interactive when it is presented in a gentle and inviting tone, rather than in direct statements or orders. At the same time, by asking whether the person surveyed is more able to understand the thoughts of others, the person interviewed demonstrates respect for and concern for the feelings and wishes of the person interviewed."}, "time_dimension": {"answer": "Present", "rationale": "The entry describes the current behaviour or state of the subject, \"usually\" indicating that such conduct occurs in most cases and does not specify that it is a past act. Thus, in terms of time dimension, the subject is the present state."}, "sentence_type": "Affirmative declarative sentence", "pos": {"nouns": {"Questionnaire": 1, "Entry": 1, "The other side": 1, "鈾?To think 鈾?: 1}, "verbs": {"Clear": 1, "More": 1, "Yes": 1}, "adverbs": {"Usually": 1}, "numerals": {"2": 1}}}, {"content": "3 usually knows what both sides want.", "word_count": 11, "label": "", "sentence_structure": "Subject鈥搗erb鈥搊bject", "subjective_objective": {"answer": "Subjective", "rationale": "Because \"know what both sides want\" is the patient's perception of his relationship with others, and it's subjective."}, "sentence_sentiment": {"answer": "Positive", "rationale": "Because \"know what the parties want\" means good communication and understanding, it's a positive attitude and behaviour."}, "language_style": {"answer": "Tactful and warm", "rationale": "The entry is more adept and interactive when it is spoken in a gentle and inviting manner, rather than by direct statements or orders."}, "time_dimension": {"answer": "Present", "rationale": "The keywords \"normally\" and \"know\" now indicate that this is a regular, cognitive act, which means that the person tested has such an act at the current point of time. The entry therefore describes the person who was tested for the present act or state."}, "sentence_type": "Affirmative declarative sentence", "pos": {"nouns": {"Questionnaire Entry": 1, "Both sides": 1, "鈾?To think 鈾?: 2}, "verbs": {"Yes": 1, "have": 1, "Analysis": 1}, "numerals": {"3": 1}, "pronouns": {"you": 1, "I": 1}}}, {"content": "Two likes intimate, hoping to be close to each other.", "word_count": 13, "label": "", "sentence_structure": "Subject鈥搗erb鈥揷omplement", "subjective_objective": {"answer": "Subjective", "rationale": "Because love and hope are patients' subjective emotional attitudes."}, "sentence_sentiment": {"answer": "Positive", "rationale": "Because the entries express the desire and desire for intimate relationships.\""}, "language_style": {"answer": "Tactful and warm", "rationale": "Warm and warm."}, "time_dimension": {"answer": "Present", "rationale": ""}, "sentence_type": "Affirmative declarative sentence", "pos": {"nouns": {"Questionnaire Entry": 1, "Intimacy": 2, "Hope": 1, "The other side": 1}, "verbs": {"Yeah": 1, "Hope": 1, "鈾?Intimacy 鈾?: 1}, "adjectives": {"Intimacy": 1, "No space": 1}, "numerals": {"2": 1}, "pronouns": {"The other side": 1}}}, {"content": "Three wants to be close but needs to keep a distance.", "word_count": 14, "label": "", "sentence_structure": "Subject鈥搗erb鈥揷omplement", "subjective_objective": {"answer": "Subjective", "rationale": "Because \"a desire for intimacy\" and \"a need to keep a distance\" are personal feelings and needs of the patient and are subjectively viewed."}, "sentence_sentiment": {"answer": "Neutral", "rationale": "Because the entry describes a demand or preference and does not contain a clear emotional colour, it can be judged neutral."}, "language_style": {"answer": "Tactful and warm", "rationale": "The language style of the entry is more euphemistic, subtle, and soft, and the need to keep a distance is emphasized in the expression of desire for intimacy. This expression is more acceptable and understandable."}, "time_dimension": {"answer": "Present", "rationale": "The key words in the entry are \"a yearning\" and \"need\", which indicate the needs and ideas of the subjects at the current stage and therefore judge them as now."}, "sentence_type": "Imperative sentence", "pos": {"nouns": {"Desire": 1, "Intimacy": 1, "Distance": 2}, "verbs": {"Yes": 1, "Hold on": 1, "鈾?Want to keep 鈾?: 1}, "adverbs": {"I'm sure": 1}, "numerals": {"3": 1}}}, {"content": "One doesn't want to be too close, or it'll feel uncomfortable.", "word_count": 14, "label": "", "sentence_structure": "Subject鈥搗erb鈥揷omplement", "subjective_objective": {"answer": "Subjective", "rationale": "Because \"not too close\" is the individual feeling and attitude of the patient, and it is subjective."}, "sentence_sentiment": {"answer": "Negative", "rationale": "Because the \"uncomfortable\" emotion in the entry is negative."}, "language_style": {"answer": "Calm and direct", "rationale": "The language style of the entry is calm, because it clearly expresses the expectations or feelings of the other side, without too much rhetorical or emotional rendering. It conveys the message in a simple and direct way, allowing readers to quickly understand and answer questions."}, "time_dimension": {"answer": "Past", "rationale": "The entry contains the words \"not wanting\" and \"not feeling comfortable\", which indicates that this is a description of the past and therefore judgemental."}, "sentence_type": "Negative declarative sentence", "pos": {"nouns": {"Intimacy": 1, "I'm not feeling well": 1}, "verbs": {"Hope": 1, "I feel": 1}, "adjectives": {"Too much": 1}, "pronouns": {"I": 1}}}, {"content": "One doesn't like the topic of relations between the two sides.", "word_count": 12, "label": "", "sentence_structure": "Subject鈥搗erb鈥搊bject", "subjective_objective": {"answer": "Subjective", "rationale": "Because \"not like\" is a subjective emotional attitude."}, "sentence_sentiment": {"answer": "Negative", "rationale": "Because the \"not like\" attitude in the entry is negative."}, "language_style": {"answer": "Calm and direct", "rationale": "The language style of the entry is calm and straightforward, because it expresses the subject clearly and succinctly, without excessive rhetorical or emotional colours. It asks respondents in an objective and neutral manner about their preferences and preferences, without attempting to influence or manipulate the respondents' answers. This language style is suitable for official questionnaires or other occasions where objectivity and neutrality are needed."}, "time_dimension": {"answer": "Present", "rationale": "The sentence describes the current attitude and preferences of the testees, namely that they do not like to talk about the relationship between the parties."}, "sentence_type": "Negative declarative sentence", "pos": {"nouns": {"Topic": 1, "Both sides": 1}, "verbs": {"Yeah": 1, "Involving": 1}, "adjectives": {"not": 1}, "numerals": {"1": 1}}}, {"content": "Two likes to talk about relations between the two sides.", "word_count": 11, "label": "", "sentence_structure": "Subject鈥搗erb鈥搊bject", "subjective_objective": {"answer": "Subjective", "rationale": "The topic of preference for relations between the parties is a personal preference or attitude, which is subjective."}, "sentence_sentiment": {"answer": "Positive", "rationale": "Because the purpose of the act \"likes to be a topic of mutual relations\" is to establish and maintain good interpersonal relations, with positive emotional aspects."}, "language_style": {"answer": "Tactful and warm", "rationale": "The entry is more adept and interactive when it is spoken in a gentle and inviting manner, rather than by direct statements or orders."}, "time_dimension": {"answer": "Present", "rationale": "The entry asks the testee ' s current preferences, so the time dimension is now judged."}, "sentence_type": "Declarative sentence", "pos": {"nouns": {"Topic": 1, "Relations": 1, "Both sides": 1}, "verbs": {"Yeah": 1, "Involving": 1}, "numerals": {"2": 1}}}, {"content": "3 Interest in topics related to relations between the parties is consistent with each other", "word_count": 17, "label": "", "sentence_structure": "Subject鈥搗erb鈥揷omplement", "subjective_objective": {"answer": "Subjective", "rationale": "Because the unity of interest is the subjective feeling and attitude of the individual."}, "sentence_sentiment": {"answer": "Positive", "rationale": "Because the entry expresses a common interest in the subject matter of relations between the parties and is positive."}, "language_style": {"answer": "Tactful and warm", "rationale": "The entry is more personal and interactive when it is presented in a gentle and inviting tone, rather than in direct statements or orders. At the same time, the language style of the article is more concise, not too much rhetorical or emotionally retrofitting, and more pragmatic and objective."}, "time_dimension": {"answer": "Future", "rationale": "The entry contains visions and expectations for the future, and \"interest in the subject of relations with each other is a possible future scenario\" and therefore judgement is a future."}, "sentence_type": "Affirmative declarative sentence", "pos": {"nouns": {"Questionnaire": 1, "Entry": 1, "Both sides": 1, "Relations": 2, "Topic": 2, "Interest": 1, "Unanimously": 1}, "verbs": {"Involving": 1, "Analysis": 1}, "adverbs": {"Down there": 1}, "numerals": {"3": 1}, "pronouns": {"Here": 1}}}]}

{"questionnaire_title": "You're just a \"sniff\" test of your self-worth.", "questionnaire_category": "Self-cognition", "item_count": 10, "emotion_shift_overall": "From positive to negative", "language_style_overall": "Tactful and warm", "items": [{"content": "One is generally satisfied with himself.", "word_count": 10, "label": "", "sentence_structure": "Subject鈥揷opula鈥損redicative", "subjective_objective": {"answer": "Subjective", "rationale": "Because \"generally satisfied with yourself\" is a personal feeling and attitude that belongs to the patient's own subjective view."}, "sentence_sentiment": {"answer": "Positive", "rationale": "Because the attitude of \"satisfaction\" in the entry is positive."}, "language_style": {"answer": "Calm and direct", "rationale": "This is a more objective, concise tone, which does not use too many rhetorical techniques or emotional renderings. It asks whether the respondents are satisfied with their feelings as a whole, whether they speak simple language, and are not too subjective or emotional. This style is more professional and practical."}, "time_dimension": {"answer": "Present", "rationale": "鈥? is generally satisfied with himself鈥? expressing the attitude of the testee towards himself at the moment. Thus, the temporal dimension of the article is now judged."}, "sentence_type": "Affirmative declarative sentence", "pos": {"nouns": {"Questionnaire Entry": 1, "self": 1}, "verbs": {"I feel": 1, "Satisfied": 1}, "adjectives": {"In general": 1}, "numerals": {"1": 1}}}, {"content": "Two sometimes feel like they're very incompetent.", "word_count": 10, "label": "", "sentence_structure": "Subject鈥搗erb鈥揷omplement", "subjective_objective": {"answer": "Subjective", "rationale": "Because it is a subjective experience, not an objective fact or description of the conduct that occurs, it is a subjective view."}, "sentence_sentiment": {"answer": "Negative", "rationale": "Because the attitude of \"responsible incompetence\" in the entry is negative."}, "language_style": {"answer": "Tactful and warm", "rationale": "This expression is more conducive to creating a good climate of communication and trust."}, "time_dimension": {"answer": "Present", "rationale": "Based on the description of \"sometimes feeling\" and \"very incompetent\" in the sentence, it can be seen as a statement describing the current feeling, and thus judging as the present state of affairs."}, "sentence_type": "Negative declarative sentence", "pos": {"nouns": {"Feel": 1, "self": 1, "Incompetent": 1}, "verbs": {"have": 1, "I'm not sure": 1, "Okay": 1}, "adjectives": {"Very": 1}, "adverbs": {"Sometimes": 1}, "pronouns": {"2": 1}}}, {"content": "Three thinks they have a lot of merit.", "word_count": 9, "label": "", "sentence_structure": "Subject鈥揷opula鈥損redicative", "subjective_objective": {"answer": "Subjective", "rationale": "Because \"think\" is the patient's subjective feeling and perception, it's from the patient's own perspective and it's subjective."}, "sentence_sentiment": {"answer": "Positive", "rationale": "Because \"there are many advantages\" is a positive, confident expression of self-identification and self-worth, with positive emotional aspects."}, "language_style": {"answer": "Calm and direct", "rationale": "The entry directly asks the respondents about their merits, without using excessive rhetorical or emotional publicity, and with a more calm and objective language style."}, "time_dimension": {"answer": "Present", "rationale": "The entry describes the person's current feelings and perceptions, and \"seeing many advantages\" is a subjective assessment, which indicates the person's current perception and attitude to himself. So the time dimension is now."}, "sentence_type": "Affirmative declarative sentence", "pos": {"nouns": {"Advantages": 1}, "verbs": {"I think": 1}, "numerals": {"More": 1}, "classifiers": {"A lot": 1}, "pronouns": {"self": 1}}}, {"content": "The four average people can do what they can.", "word_count": 15, "label": "", "sentence_structure": "Subject鈥搗erb鈥搊bject", "subjective_objective": {"answer": "Subjective", "rationale": "This is a patient ' s description of his or her self-evaluation and attitude and is a subjective examination."}, "sentence_sentiment": {"answer": "Neutral", "rationale": "Because the entry is not clearly emotional."}, "language_style": {"answer": "Calm and direct", "rationale": "Because it expresses a point in a concise manner, it is not using too many emotional terms or rhetorical techniques, but is presented in an objective and direct manner."}, "time_dimension": {"answer": "Present", "rationale": "And by the word \"capable\" and \"also\" it can be seen as the general state of affairs, describing the state or capacity of the present, and thus judging it as the present."}, "sentence_type": "Affirmative declarative sentence", "pos": {"nouns": {"Yes": 2, "person": 2}, "verbs": {"Yes": 3, "Do it": 2}, "adjectives": {"General": 1}, "adverbs": {"Okay": 2, "self": 1}, "numerals": {"4": 1}, "pronouns": {"Yeah": 1}}}, {"content": "Five thinks they don't have anything to be proud of.", "word_count": 13, "label": "", "sentence_structure": "Subject鈥揷opula鈥損redicative", "subjective_objective": {"answer": "Subjective", "rationale": "Because it is self-evaluation and perception of patients that they feel that they are not proud of themselves, it is a subjective examination."}, "sentence_sentiment": {"answer": "Negative", "rationale": "Because the phrase \"no proud place of yourself\" in the entry is negative."}, "language_style": {"answer": "Tactful and warm", "rationale": "This language style is more personal and interactive than directly asking the interviewee whether he has confidence and merit."}, "time_dimension": {"answer": "Present", "rationale": "The keywords are \"I feel\" and \"I have no place to be proud of\", which imply an assessment and recognition of the current state of self."}, "sentence_type": "Negative declarative sentence", "pos": {"nouns": {"self": 1, "Local": 1}, "verbs": {"I think": 1, "have": 1, "Proud": 1}, "adverbs": {"very": 1}, "numerals": {"5": 1}}}, {"content": "Six sometimes feel like they're doing nothing.", "word_count": 10, "label": "", "sentence_structure": "Subject鈥揷opula鈥損redicative", "subjective_objective": {"answer": "Subjective", "rationale": "Because \"feel\" is usually subjectively felt and understood, not objective facts or data."}, "sentence_sentiment": {"answer": "Negative", "rationale": "Because the expression \"inaction\" in the entry is negative and reflects a self-denial, negative and frustrating emotional attitude."}, "language_style": {"answer": "Calm and direct", "rationale": "When the entry asks the other side whether it feels unable to work in a direct and clear manner, it is more straightforward and does not use excessive rhetoric or emotional colours."}, "time_dimension": {"answer": "Present", "rationale": ""}, "sentence_type": "Negative declarative sentence", "pos": {"nouns": {"self": 1, "As": 1}, "verbs": {"Feel": 1, "Nothing": 1, "be": 1}, "adjectives": {"Sometimes": 1}, "numerals": {"6": 1}}}, {"content": "Seven thinks they have to be worth at least the same amount as others.", "word_count": 18, "label": "", "sentence_structure": "Subject鈥揷opula鈥損redicative", "subjective_objective": {"answer": "Subjective", "rationale": "Because \"I feel at least as valuable as anyone else\" is the patient's inner feeling and self-evaluation, which is subjective."}, "sentence_sentiment": {"answer": "Neutral", "rationale": "Since the entry does not express positive or negative emotional tendencies, it is merely a descriptive description and asks whether the respondents' expectations of their own values are comparable to those of others."}, "language_style": {"answer": "Tactful and warm", "rationale": "The language style of the entry is more moderate and encouraging, using subjective expressions such as \"think\" to allow respondents to think and answer questions in a more euphemistic manner, more personal and interactive."}, "time_dimension": {"answer": "Present", "rationale": "The sentence \"sweet\" and \"yes\" makes it possible to judge that the entry is an inquiry into the current behaviour or feelings of the subject, and therefore the temporal dimension is now."}, "sentence_type": "Affirmative declarative sentence", "pos": {"nouns": {"self": 1, "person": 1, "Value": 2}, "verbs": {"I think": 1, "have": 1, "Equal": 1, "Level": 1}, "adverbs": {"At least": 1}, "numerals": {"7": 1}}}, {"content": "I'd like to think I'd respect myself a little more.", "word_count": 16, "label": "", "sentence_structure": "Subject鈥搗erb鈥揷omplement", "subjective_objective": {"answer": "Subjective", "rationale": "Since respect for one ' s own self is an individual ' s subjective feeling and perception, it is the subjective attitude and psychological state of the patient that is examined."}, "sentence_sentiment": {"answer": "Negative", "rationale": "Because it expresses dissatisfaction and regret at itself, it is a negative emotional colour."}, "language_style": {"answer": "Tactful and warm", "rationale": "The entry expresses a moderate and encouraging expression of hope that the interviewee will be more sensitive to his feelings than to direct orders or accusations. Such language style is more personal and interactive, and more resonant and responsive to the interviewee."}, "time_dimension": {"answer": "Present", "rationale": "The keywords \"thinking you\" and \"have a little respect for yourself\" indicate that it is a description of and expectations for your current state."}, "sentence_type": "Exclamatory sentence", "pos": {"nouns": {"self": 2, "Points": 1}, "verbs": {"I think": 1, "Respect": 1, "Yes": 1, "Good": 1}, "adjectives": {"8": 1}, "adverbs": {"More": 1}}}, {"content": "I feel like I'm not doing anything right.", "word_count": 16, "label": "", "sentence_structure": "Subject鈥揷opula鈥損redicative", "subjective_objective": {"answer": "Subjective", "rationale": "Because \"bad\" is a personal feeling or self-evaluation that reflects patients' subjective perception of their abilities."}, "sentence_sentiment": {"answer": "Negative", "rationale": "Because the entry \"as if you did anything badly\" expresses a self-denial and negative sentiment and is therefore negative."}, "language_style": {"answer": "Tactful and warm", "rationale": ""}, "time_dimension": {"answer": "Present", "rationale": "The entry is asking the testee what he feels and what he thinks, and the words \"like\" and \"like\" indicate that it is a subjective self-evaluation, and \"doing anything bad\" is a negative emotional experience. So, the time dimension of the entry is now."}, "sentence_type": "Negative declarative sentence", "pos": {"nouns": {"self": 1, "Yes": 1}, "verbs": {"Do it": 2}, "adjectives": {"Okay": 1}, "numerals": {"9": 1}}}, {"content": "Everything is going to be fine.", "word_count": 13, "label": "", "sentence_structure": "Subject鈥搗erb鈥揷omplement", "subjective_objective": {"answer": "Subjective", "rationale": "Because \"will look to the good\" is a description of a personal attitude or belief that reflects the patient's mindset and perspective, it is a subjective examination."}, "sentence_sentiment": {"answer": "Positive", "rationale": "Because the \"good-looking\" attitude in the entry is positive."}, "language_style": {"answer": "Tactful and warm", "rationale": "The language style of the entry is mild and encouraging, and by expressing that \"everything will go in the right direction\", it leads the interviewee to be positive and optimistic, rather than to direct statements or orders. This euphemism, inspiring question-and-answer approach makes it easier for the interviewee to answer and share his thoughts."}, "time_dimension": {"answer": "Future", "rationale": "The entry \"everything will be better\" contains visions and expectations for the future, and the keywords are \"everything\" and \"will\". They point to situations that have not yet occurred and that may arise in the future. The temporal dimension of the article therefore determines the future."}, "sentence_type": "Affirmative declarative sentence", "pos": {"nouns": {"Yes": 1, "In terms of": 1}, "verbs": {"Yes": 2, "Go on": 1}, "adjectives": {"Okay": 1}, "adverbs": {"Both": 1, "All": 1}, "numerals": {"10": 1}}}]}
